# Supplementary material for: Pathway-divergent coupling of 1,3-enynes with acrylates through cascade cobalt catalysis
Source: Nat Commun. 2024 Apr 23;15:3427. doi: 10.1038/s41467-024-47719-1 (PMC11039462; doi:10.1038/s41467-024-47719-1)
Supplement: Supplementary file 1 — Supplementary Information [file 41467_2024_47719_MOESM1_ESM.pdf]

Supplementary Information

Pathway-divergent coupling of 1,3-enynes with acrylates through cascade cobalt catalysis

Heng Wang,[1] Xiaofeng Jie,[1] Qinglei Chong,[1]* and Fanke Meng[1,2,3,4]*

[1]State Key Laboratory of Organometallic Chemistry, Center for Excellence in Molecular Synthesis, Shanghai Institute of Organic Chemistry, University of Chinese Academy of Sciences, 345 Lingling Road, Shanghai, 200032, China

[2]State Key Laboratory of Elemento-Organic Chemistry, Nankai University

[3]School of Chemistry and Materials Science, Hangzhou Institute for Advanced Study University of Chinese Academy of Sciences, 1-Sub-lane Xiangshan, Hangzhou, 310024, China

[4]Beijing National Laboratory for Molecular Sciences

*Correspondence: chongql@sioc.ac.cn, mengf@sioc.ac.cn

Table of Contents

1. Supplementary Methods 3
1.1 General Remarks 3
1.2 Reagents and Ligands 4
1.3 General Procedure and Characterization 6
1.3.1 General Procedure for Co-Catalyzed Enantioselective Coupling of Alkyl-substituted 1,3-Enynes and Acrylates 6
1.3.2 General Procedure for Co-Catalyzed Enantioselective Coupling of Aryl-substituted 1,3-Enynes and Acrylates 6
1.3.3 General Procedure for Co-Catalyzed Coupling of 1,3-Enynes and Acrylates to Generate Achiral 1,3-Dienes Bearing a Tetrasubstituted Alkene 7
1.3.4 General Procedure for Co-Catalyzed Coupling of 1,3-Enynes and Acrylates to Generate Achiral 1,3-Dienes Containing Two Trisubstituted Olefins 7
1.4 Experimental Procedure and Characterization for Gram Scale Reaction and Functionalization 8
1.5 Experimental Procedure and Characterization for Mechanistic Studies 20
1.5.1 Deuterium Labeling Experiments 20
1.5.2 EPR Studies 28
1.5.3 SAESI-MS Study 32
2. Supplementary Notes 37
2.1 Additional Optimization of Reaction Conditions 37
2.2 Characterization of Products 48
2.3 Proof of Stereochemistry: X-ray Characterization Data 97
2.4 Copies of NMR Spectra 167
2.4 Copies of HPLC Spectra 293
3. Supplementary References 348

1. Supplementary Methods

1.1 General Remarks

Infrared (IR) spectra were recorded on a BRUKER TENSOR 27 FT-IR spectrometer, λmax in cm^{-1}. Bands are characterized as broad (br), strong (s), medium (m), and weak (w). ^{1}H NMR spectra were recorded on a Bruker 600 MHz, Bruker 400 MHz, Agilent 400 MHz or Varian 400 MHz spectrometers. Chemical shifts are reported in ppm with the solvent resonance as the internal standard ($CDCl_3$: δ 7.26 ppm), or tetramethylsilane as internal standard (TMS: δ 0.00 ppm). Data are reported as follows: chemical shift, integration, multiplicity (s = singlet, d = doublet, t = triplet, q = quartet, m = multiplet), and coupling constant (Hz). ^{13}C NMR spectra were recorded on a Bruker 100 MHz, Agilent 100 MHz or Varian 100 MHz spectrometers with complete proton decoupling. Chemical shifts are reported in ppm from tetramethylsilane with the solvent resonance as the internal standard ($CDCl_3$: δ 77.00 pm). EI-HRMS and ESI-HRMS spectra were obtained on a Waters Micromass G1540N/GCT Premier and a Thermo Fisher Scientific LTQ FT Ultra, respectively. Enantiomer ratios were determined by high-performance liquid chromatography (HPLC) or supercritical fluid chromatography (SFC) from Shimadzu Corporation. (Chiral Technologies Chiralpak IA (4.6 x 250 mm), Chiralpak IB (4.6 x 250 mm), Chiralpak IBN-5 (4.6 x 250 mm), Chiralpak IC (4.6 x 250 mm), Chiralpak ID (4.6 x 250 mm), Chiralpak IE (4.6 x 250 mm), Chiralpak IG (4.6 x 250 mm), Chiralcel OJ-H (4.6 x 250 mm), Chiralcel OZ-H (4.6 x 250 mm), Chiralcel OD-H (4.6 x 250 mm) Chiralpak AD-H (4.6 x 250 mm) Chiralpak AS-H (4.6 x 250 mm) Chiralpak IJ-3 (4.6 x 250 mm)) in comparison with authentic racemic materials. Specific rotations were measured on a Rudolph Research Analytical Autopol VI Polarimeter and Autopol I Polarimeter. Unless otherwise noted, all reactions were carried out with anhydrous solvents under an atmosphere of dry N_2 in oven- or flame-dried glassware with standard dry box or vacuum-line techniques. Anhydrous tetrahydrofuran, acetonitrile, sulfoxide (J&K Scientific and used as received.) were used without further purification. All work-up and purification procedures were carried out with reagent grade solvents (purchased from Adamas Reagent, Ltd.) in air. Manganese (-325 mesh, 99.95%) and indium (-325 mesh, 99.99%) were purchased from Alfa Aesar and used without further activation. Zinc (-100 mesh, 98+%) was purchased from Acros, and was washed successively with 0.1 M HCl, diethyl ether, and acetone, dried under vacuum, and stored under argon.

1.2 Reagents and Ligands

All of the cobalt salts and phosphine ligands: purchased from Strem Chemicals Inc. and used as received.

All of the acrylates: prepared according to previous reports[1-4].

Benzyl (2E,4E)-hexa-2,4-dienoate: prepared according to previous reports[5].

All of the aryl 1,3-enynes: prepared according to previous reports[6-8].

All of the 1º, 2º, 3ºalkyl substituted 1,3-enynes: prepared according to previous reports[6-7,9-10].

All of the 1,3-enynes bearing quaternary centers substituted with two alkyls and one aryl group : prepared followed the procedure bellowed.

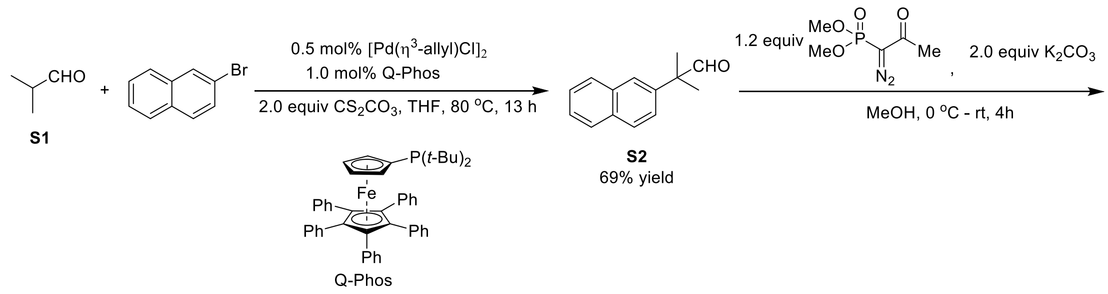

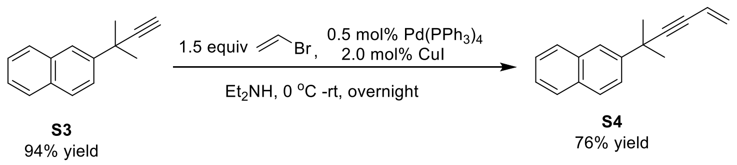

Inside a nitrogen-filled glovebox, $[Pd(\eta^3\text{-allyl})Cl]_2$ (18.3 mg, 0.005 mmol, 0.5 mol%), Q-phos (70.7 mg, 0.050 mmol, 1.0 mol%), and THF (10 mL) were added to a 100 ml thick wall pressure bottle containing a magnetic stir bar. The mixture was allowed to stir for 5 mins at room temperature. Cesium carbonate (6.5164 g, 20.0 mmol, 2.0 equiv), a branched aldehyde **S1** (0.8653g, 12 mmol, 1.2 equiv) and 2-bromonaphthalene (2.0707g, 10.0 mmol, 1.0 equiv) were then added. THF (10 mL) was then added via a gas-tight syringe. The bottle was sealed with a Teflon-lined screwcap, removed from the glovebox, and placed in a 80 ºC oil bath for 13 h. After complete conversion of the aryl halide, the reaction mixture was diluted with ethyl acetate (50 mL) and filtered through a pad of Celite. The filtrate was concentrated in vacuo and the crude product was

purified by flash column chromatography on silical gel to give the α-aryl aldehyde **S2** (1.3712 g, 69% yield) as colorless oil.

At 0 ºC, to a stirred mixture of **S2** (1.3712 g, 6.9158 mmol, 1.0 equiv) and K_2CO_3 (1.9117 g, 13.8316 mmol, 2.0 equiv) in MeOH (14 ml) was added dropwise a solution of dimethyl 1-diazo-2-oxopropylphosphonate (1.5943 g, 8.2990 mmol, 1.2 equiv) in MeOH (14 ml). The mixture was allowed to stir at rt for 4h. Then the reaction was quenched by adding water to the reaction solution and the resulting solution was extracted with Et_2O (50 mL×2). The organic layer was washed with brine, dried over anhydrous $MgSO_4$ and concentrated. The residue was purified by flash chromatography to give the α-aryl alkyne **S3** (1.2630g, 94% yield) as colorless oil.

To a three-necked flask with $Pd(PPh_3)_4$ (0.0.0376 g, 0.0325 mmol, 0.5 mol%) and CuI (0.0248 g, 0.1302 mmol, 2.0 mol%) was added Et_2NH (6.5 mL) under argon. Then alkyne **S3** (1.2644g, 6.5081 mmol, 1.0 equiv) and bromoethane (1M in THF, 9.8 mL, 1.5 equiv) was added to above solution dropwise under ice bath. The resulting solution was allowed to stir at room temperature overnight. After this time, it was quenched by HCl aqueous solution (1.0 M, 15 mL), extracted by Et_2O (50 mL ×3), concentrated and purified by flash column chromatography to provide the product 1,3-conjugated enyne **S4** (1.0900g, 76% yield) as colorless oil.

1.3 General Procedure and Characterization

1.3.1 General Procedure for Co-Catalyzed Enantioselective Coupling of Alkyl-substituted 1,3-Enynes and Acrylates

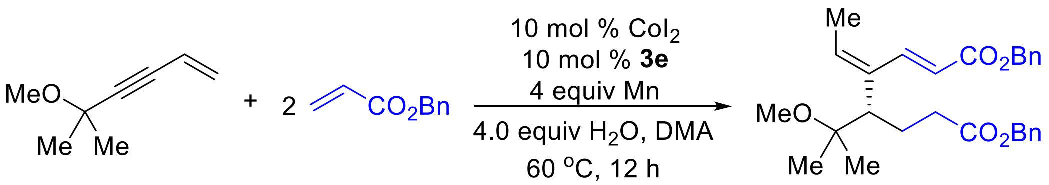

1a **2a** **4a**

In a N_2-filled glove box, an oven-dried 8-mL vial equipped with a stirring bar was charged with CoI_2 (6.3 mg, 0.02 mmol, 10 mol%), **3e** (8.5 mg, 0.02 mmol, 10 mol%), Mn powder (44.0 mg, 0.8 mmol, 4 eq) and DMA (0.8 mL). The vial was sealed with a cap (phenolic cap with red PTFE/white silicone septum) and the solution was allowed to stir at room temperature for 30 min. **1a** (49.7 mg, 0.4 mmol, 2.0 equiv.), **2a** (64.9 mg, 0.4 mmol, 1.0 equiv.), H_2O (14.4 mg, 0.8 mmol, 4.0 equiv.) and DMA (0.2 mL) were added to the solution. Then the vial was sealed with a cap (phenolic open top cap with red PTFE/white silicone septum), removed from the glove box. The mixture was immediately moved to a thermostatic bath and allowed to stir at 60 ºC for 12 h. Upon cooling to room temperature, the reaction mixture was washed with brine (3×15 ml), eluted with Et_2O (20 mL), dried over $MgSO_4$, filtered and concentrated in *vacuo***.** The residue was purified by silica gel column chromatography (PE:EA = 40:1) to afford the **4a** as colorless oil (82.8 mg, 92% yield). The racemic material was prepared followed general procedure for preparing **4a** by replacing **3e** with **3k.**

1.3.2 General Procedure for Co-Catalyzed Enantioselective Coupling of Aryl-substituted 1,3-Enynes and Acrylates

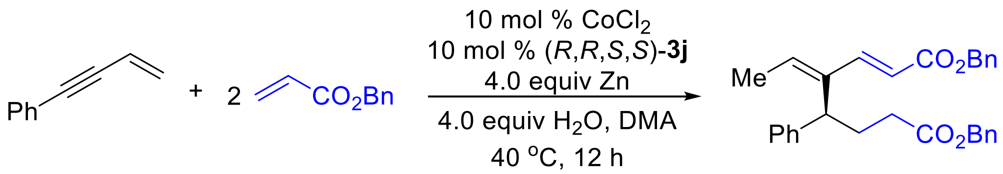

1c **2a** **6a**

In a N_2-filled glove box, an oven-dried 8-mL vial equipped with a stirring bar was charged with $CoCl_2$ (2.6 mg, 0.02 mmol, 10 mol%), (*R,R,S,S*)-**3j** (7.6 mg, 0.02 mmol, 10 mol%), Zn powder (52.3 mg, 0.8 mmol, 4 eq) and DMA (0.8 mL). The vial was sealed with a cap (phenolic cap with

red PTFE/white silicone septum) and the solution was allowed to stir at room temperature for 30 min. **1c** (51.3 mg, 0.4 mmol, 2.0 equiv.), **2a** (64.9 mg, 0.4 mmol, 1.0 equiv.), H_2O (14.4 mg, 0.8 mmol, 4.0 equiv.) and DMA (0.2 mL) were added to the solution. Then the vial was sealed with a cap (phenolic open top cap with red PTFE/white silicone septum), removed from the glove box. The mixture was immediately moved to a thermostatic bath and allowed to stir at 40 ºC for 12 h. Upon cooling to room temperature, the reaction mixture was washed with brine (3×15 mL), eluted with Et_2O (20 mL), dried over $MgSO_4$, filtered and concentrated in *vacuo***.** The residue was purified by silica gel column chromatography (PE:EA = 40:1) to afford the **6a** as colorless oil (48.5 mg, 53% yield). The racemic material was prepared followed general procedure for preparing **6a** by replacing (*R,R,S,S*)-**3j** with rac-**3j.**

1.3.3 General Procedure for Co-Catalyzed Coupling of 1,3-Enynes and Acrylates to Generate Achiral 1,3-Dienes Bearing a Tetrasubstituted Alkene

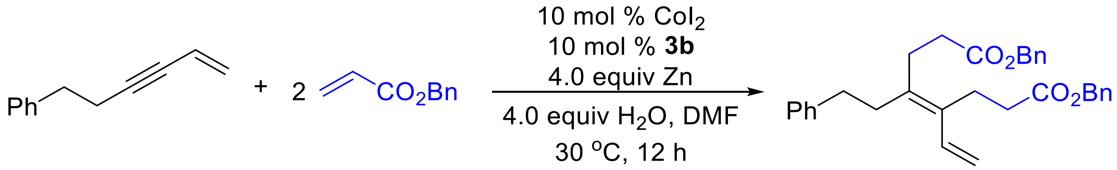

In a N_2-filled glove box, an oven-dried 8-mL vial equipped with a stirring bar was charged with CoI_2 (6.3 mg, 0.02 mmol, 10 mol%), **3b** (12.2 mg, 0.02 mmol, 10 mol%), zinc powder (52.3 mg, 0.8 mmol, 4 equiv) and DMF (0.3 mL). The vial was sealed with a cap (phenolic cap with red PTFE/white silicone septum) and the solution was allowed to stir at room temperature for 30 min. **1b** (62.5 mg, 0.4 mmol, 2.0 equiv.), **2a** (64.9 mg, 0.4 mmol, 1.0 equiv.), H_2O (14.4 mg, 0.8 mmol, 4.0 equiv.) and DMF (0.2 mL) were added to the solution. Then the vial was sealed with a cap (phenolic open top cap with red PTFE/white silicone septum), removed from the glove box. The mixture was immediately moved to a thermostatic bath and allowed to stir at 30 ºC for 12 h. Upon cooling to room temperature, the reaction mixture was washed with brine (3×15 mL), eluted with Et_2O (20 mL), dried over $MgSO_4$, filtered and concentrated in *vacuo***.** The residue was purified by silica gel column chromatography (PE:EA = 20:1) to afford the **8a** as colorless oil (76.4 mg, 79% yield).

1.3.4 General Procedure for Co-Catalyzed Coupling of 1,3-Enynes and Acrylates to Generate Achiral 1,3-Dienes Containing Two Trisubstituted Olefins

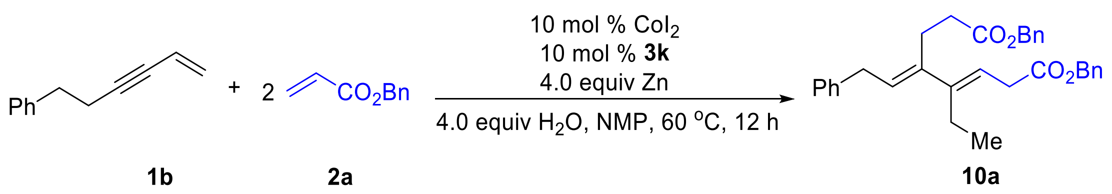

In a N_2-filled glove box, an oven-dried 8-mL vial equipped with a stirring bar was charged with CoI_2 (6.3 mg, 0.02 mmol, 10 mol%), **3k** (8.0 mg, 0.02 mmol, 10 mol%), zinc powder (52.3 mg, 0.8 mmol, 4 eq) and NMP (0.8 mL). The vial was sealed with a cap (phenolic cap with red PTFE/white silicone septum) and the solution was allowed to stir at room temperature for 30 min. **1b** (62.5 mg, 0.4 mmol, 2.0 equiv.), **2a** (64.9 mg, 0.4 mmol, 1.0 equiv.), H_2O (14.4 mg, 0.8 mmol, 4.0 equiv.) and NMP (0.2 mL) were added to the solution. Then the vial was sealed with a cap (phenolic open top cap with red PTFE/white silicone septum), removed from the glove box. The mixture was immediately moved to a thermostatic bath and allowed to stir at 60 ºC for 12 h. Upon cooling to room temperature, the reaction mixture was washed with brine (3×15 ml), eluted with Et_2O (20 mL), dried over $MgSO_4$, filtered and concentrated in vacuo. The residue was purified by silica gel column chromatography (PE:EA = 40:1) to afford the **10a** as colorless oil (76.3 mg, 79% yield).

1.4 Experimental Procedure and Characterization for Gram Scale Reaction and Functionalization

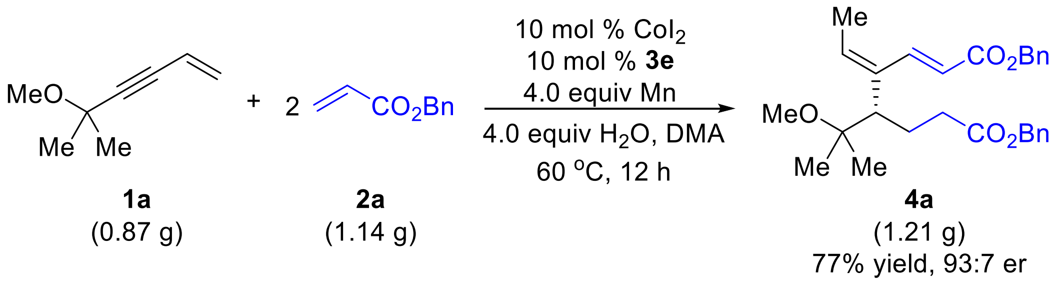

In a N_2-filled glove box, an oven-dried 50-mL round bottom flask equipped with a stirring bar was charged with CoI_2 (109.5 mg, 0.35 mmol, 10 mol%), **3e** (149.3 mg, 0.35 mmol, 10 mol%), Mn powder (0.7692 g, 14 mmol, 4 equiv) and DMA (15 mL). The solution was allowed to stir at room temperature for 30 min. **1a** (0.8693 g, 7 mmol, 2.0 equiv), **2a** (1.1353 g, 7 mmol, 1.0 equiv), H_2O (0.2523 g, 14 mmol, 4.0 equiv) and DMF (2.5 mL) were added to the solution. Then the flask was sealed with a cap, removed from the glove box. The mixture was immediately moved to a thermostatic bath and allowed to stir at 60 ºC for 12 h. Upon cooling to room temperature, the

reaction mixture was washed with brine (3×70 ml), eluted with Et_2O (100 mL), dried over $MgSO_4$, filtered and concentrated in *vacuo*. The residue was purified by silica gel column chromatography (PE:EA = 40:1) to afford the **4a** as colorless oil (1.21 g, 77% yield, 93:7 er).

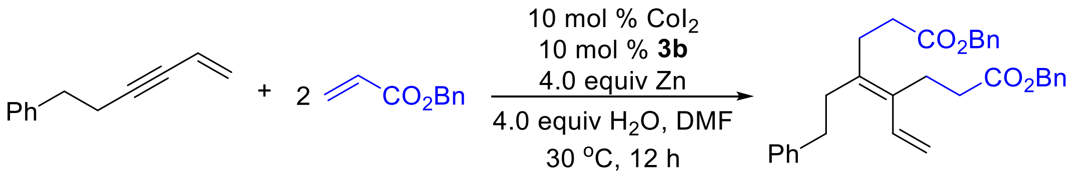

1b (1.25 g) **2a** (1.30 g) **8a** (1.74 g) 90% yield, >98% *E*

In a N_2-filled glove box, an oven-dried 25-mL round bottom flask equipped with a stirring bar was charged with CoI_2 (125.1 mg, 0.4 mmol, 10 mol%), **3b** (244.2 mg, 0.4 mmol, 10 mol%), zinc powder (1.0466 g, 16 mmol, 4.0 equiv) and DMF (8 mL). The solution was allowed to stir at room temperature for 30 min. **1b** (1.2498 g, 8 mmol, 2.0 equiv), **2a** (1.2975 g, 8 mmol, 1.0 equiv), H_2O (0.2885 g, 16 mmol, 4.0 equiv) and DMF (2 mL) were added to the solution. Then the flask was sealed with a cap, removed from the glove box. The mixture was immediately moved to a thermostatic bath and allowed to stir at 30 ºC for 12 h. Upon cooling to room temperature, the reaction mixture was washed with brine (3×50 mL), eluted with Et_2O (70 mL), dried over $MgSO_4$, filtered and concentrated in *vacuo*. The residue was purified by silica gel column chromatography (PE:EA = 20:1) to afford the **8a** as colorless oil (1.7361 g, 90% yield).

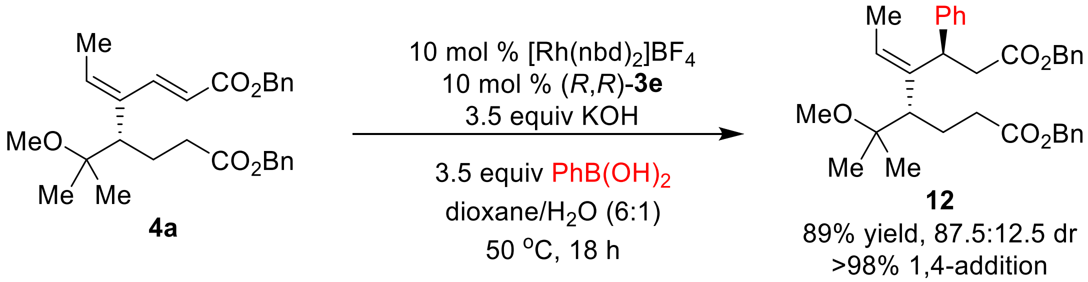

4a

12
89% yield, 87.5:12.5 dr
>98% 1,4-addition

12 was prepared followed previous reported procedure[11]: In a N_2-filled glove-box, an oven-dried vial (8 mL) with a magnetic stir bar was charged with dioxane (1 mL) and H_2O (0.25 mL). Then $[Rh(nbd)_2]BF_4$ (3.7 mg, 0.01 mmol, 10 mol%) and (*R,R*)-Chiraphos (4.3 mg, 0.01 mmol, 10 mol%) was added to the solution. The mixture was allowed to stir at rt for 0.5 h. Then **4a** (45.1 mg, 0.1 mmol, 1.0 equiv), $PhB(OH)_2$ (42.7 mg, 0.35 mmol, 3.5 equiv), KOH (19.6 mg, 0.35 mmol, 3.5 equiv) and dioxane (0.5 mL) were added respectively. The mixture was heated to 50 ºC for 18 h. Upon completion, the reaction was quenched by H_2O (5 mL) and diluted with EA (5 mL). The

layer was separated and the aqueous layer was extracted with EA (5 mL × 2). The combined layers were concentrated, purified by column chromatography (PE:EA = 15:1) to give the product **12** as colorless oil (46.9 mg, 89% yield, 87.5:12.5 dr).

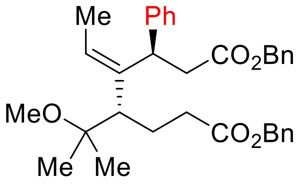

12

Dibenzyl (3*R*,5*S*,*E*)-4-ethylidene-5-(2-methoxypropan-2-yl)-3-phenyloctanedioate (12)

Colorless oil, 89% yield, 87.5:12.5 dr. **IR (neat):** 3030 (w), 2972 (w), 2828 (w), 2361 (w), 1734 (s), 1602 (w), 1496 (w), 1454 (w), 1415 (w), 1380 (w), 1258 (w), 1213 (w), 1151 (s), 1078 (w), 1032 (w), 999 (w), 912 (w), 804 (w), 749 (m), 699 (s), 664 (w), 624 (w) cm^{-1}; **^{1}H NMR (400 MHz, $CDCl_3$)** δ 7.40 – 7.32 (m, 3H), 7.32 – 7.26 (m, 5H), 7.24 – 7.21 (m, 6H), 7.17 – 7.15 (m, 1H), 5.46 (q, *J* = 6.8 Hz, 1H), 5.11 – 5.02 (m, 2H), 5.02 – 4.94 (m, 2H), 4.60 (dd, *J* = 8.8, 6.4 Hz, 1H), 3.11 (s, 3H), 3.08 – 2.99 (m, 1H), 2.93 (dd, *J* = 16.4, 6.4 Hz, 1H), 2.05 – 1.99 (m, 1H), 1.84 (d, *J* = 7.0 Hz, 3H), 1.79 – 1.62 (m, 2H), 1.56 – 1.31 (m, 2H), 1.14 (s, 3H), 1.09 (s, 3H); **^{13}C NMR (100 MHz, $CDCl_3$)** δ 173.9, 172.5, 142.5, 141.1, 136.2, 136.0, 128.5, 128.5, 128.4, 128.4, 128.2, 128.1, 128.0, 127.9, 126.3, 123.4, 77.8, 66.0, 65.7, 48.4, 42.6, 37.9, 31.9, 23.9, 22.5, 20.9, 14.4; **HRMS (ESI) $[M+Na]^+$** Calcd for $C_{34}H_{40}O_5Na$: 551.27680 m/z, Found: 551.27684 m/z; **Specific rotation**: $[\alpha]_D^{27.3}$ -55.6 (*c* 1.00, $CHCl_3$) for an enantiomerically enriched sample of 87.5:12.5 dr.

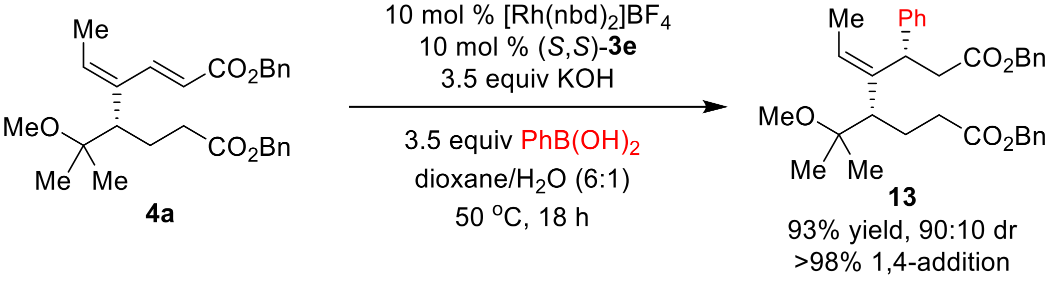

13 was prepared followed previous reported procedure[13]: In a N_2-filled glove-box, an oven-dried vial (8 mL) with a magnetic stir bar was charged with dioxane (1 mL) and H_2O (0.25 mL). Then $[Rh(nbd)_2]BF_4$ (3.7 mg, 0.01 mmol, 10 mol%) and (*S*,*S*)-Chiraphos (4.3 mg, 0.01 mmol, 10 mol%) was added to the solution. The mixture was allowed to stir at rt for 0.5 h. Then 4a (45.1 mg, 0.1 mmol, 1.0 equiv), $PhB(OH)_2$ (42.7 mg, 0.35 mmol, 3.5 equiv), KOH (19.6 mg, 0.35 mmol, 3.5 equiv) and dioxane (0.5 mL) were added respectively. The mixture was heated to 50 °C for 18 h.

Upon completion, the reaction was quenched by H_2O (5 mL) and diluted with EA (5 mL). The layer was separated and the aqueous layer was extracted with EA (5 mL × 2). The combined layers were concentrated, purified by column chromatography (PE:EA = 15:1) to give the product **13** as colorless oil (49.2 mg, 93% yield, 90:10 dr).

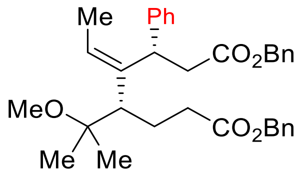

13

Dibenzyl (3*S*,5*S*,*E*)-4-ethylidene-5-(2-methoxypropan-2-yl)-3-phenyloctanedioate (13)

Colorless oil, 93% yield, 90:10 dr. **IR (neat):** 3032 (w), 2972 (w), 2826 (w), 1733 (s), 1602 (w), 1496 (w), 1454 (w), 1380 (w), 1259 (w), 1211 (w), 1153 (m), 1075 (w), 999 (w), 912 (w), 805 (w), 749 (m), 699 (s), 675 (w), 625 (w) cm^{-1}; **^{1}H NMR (400 MHz, $CDCl_3$)** δ 7.40 – 7.32 (m, 5H), 7.32 – 7.26 (m, 4H), 7.25 – 7.19 (m, 6H), 7.19 – 7.14 (m, 1H), 5.51 (q, *J* = 7.2 Hz, 1H), 5.10 – 5.01 (m, 4H), 4.32 (dd, *J* = 10.0, 4.8 Hz, 1H), 3.14 – 3.06 (m, 1H), 3.01 (s, 3H), 2.92 (dd, *J* = 15.2, 4.8 Hz, 1H), 2.34 – 2.22 (m, 2H), 2.08 – 1.98 (m, 1H), 1.97 – 1.90 (m, 1H), 1.86 (d, *J* = 7.2 Hz, 3H), 1.75 – 1.68 (m, 1H), 0.96 (s, 3H), 0.80 (s, 3H). **^{13}C NMR (100 MHz, $CDCl_3$)** δ 173.9, 172.2, 142.1, 141.3, 136.1, 135.8, 128.5, 128.44, 128.40, 128.2, 128.14, 128.09, 128.05, 128.03, 126.5, 123.1, 78.0, 66.2, 65.9, 48.6, 39.3, 32.6, 25.8, 22.6, 22.5, 14.8; **HRMS (ESI) $[M+Na]^+$** Calcd for $C_{34}H_{40}O_5Na$: 551.27680 m/z, Found: 551.27647 m/z; **Specific rotation**: $[\alpha]_D^{26.7}$ -18.5 (*c* 1.00, $CHCl_3$) for an enantiomerically enriched sample of 90:10 dr.

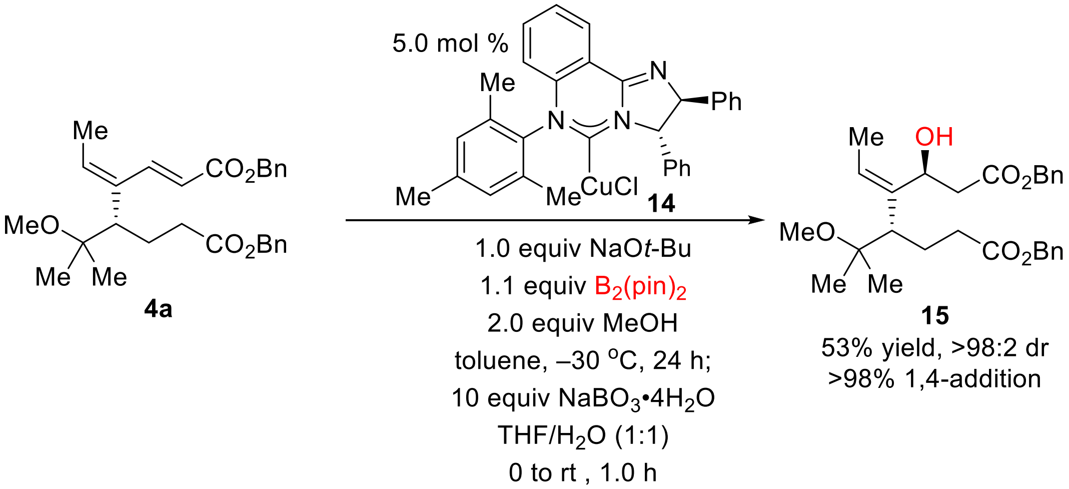

15 was prepared followed previous reported procedure[12]: **4a** (45.1 mg, 0.10 mmol, 1.0 equiv) and bis(pinacolato) diboron (27.9 mg, 0.11 mmol, 1.1 equiv) were dissolved in toluene (1 mL).

NaO*t*Bu (10.6 mg, 0.11 mmol, 1.1 equiv) was added to the reaction mixture. And then the reaction mixture was cooled to -30 °C and MeOH (8 μL, 0.2 mmol) was added. After 5 min, **14** (2.7 mg, 0.005 mmol, 5 mol%) was added. After complete consumption of **4a**, the reaction mixture was filtered through silica gel and washed with toluene. The filtrate was concentrated under rotary evaporator. The resulting residue was dissolved in THF (1 mL) and H_2O (1 mL) and cooled to 0 °C before $NaBO_4 \cdot 4H_2O$ was added. The reaction mixture was allowed to stir vigorous for 1h at rt. The reaction mixture was quenched with H_2O and extracted with EA (3×10 mL). The combined organic layers were washed with brine, dried over $MgSO_4$ and concentrated. The residue was purified by flash chromatography (PE:EA = 10:1) To give the product **15** as cololess oil (24.8 mg, 53% yield, >98:2 dr)

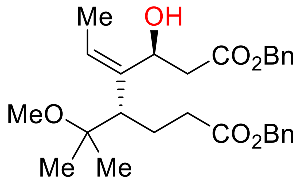

15

Dibenzyl (3S,5S,Z)-4-ethylidene-3-hydroxy-5-(2-methoxypropan-2-yl)octanedioate (15)

Colorless oil, 53% yield, >20:1 dr. **IR (neat):** 3063 (w), 3035 (w), 2923 (w), 2851 (w), 1732 (s), 1497 (w), 1456 (w), 1418 (w), 1381 (w), 1360 (w), 1269 (w), 1236 (w), 1214 (w), 1157 (s), 1072 (m), 998 (w), 972 (w), 914 (w), 827 (w), 748 (m), 698 (s), 652 (w), 625 (w) cm^{-1}; **^{1}H NMR (400 MHz, $CDCl_3$)** δ 7.40 – 7.28 (m, 10H), 5.36 (q, $J = 7.2$ Hz, 1H), 5.17 (d, $J = 1.6$ Hz, 2H), 5.09 (s, 2H), 4.86 (s, 1H), 3.16 (s, 3H), 2.75 (dd, $J = 15.6$, 10.4 Hz, 1H), 2.60 (dd, $J = 15.6$, 3.2 Hz, 1H), 2.45 – 2.38 (m, 1H), 2.30 – 2.21 (m, 2H), 2.02 – 1.94 (m, 1H), 1.82 (s, 1H), 1.76 (d, $J = 7.2$ Hz, 3H), 1.14 (s, 3H) , 1.12 (s, 3H); **^{13}C NMR (100 MHz, $CDCl_3$)** δ 173.9, 172.4, 139.9, 136.0, 135.9, 128.5, 128.2, 128.1, 77.9, 68.9, 48.8, 41.2, 32.7, 29.3, 23.7, 21.5, 14.1; **HRMS (ESI) $[M+Na]^+$** Calcd for $C_{28}H_{36}O_6Na$: 491.24041 m/z, Found: 491.24024 m/z; **Specific rotation**: $[\alpha]_D^{27.2}$ +10.0 (*c* 1.00, $CHCl_3$) for an enantiomerically enriched sample of >98:2 dr.

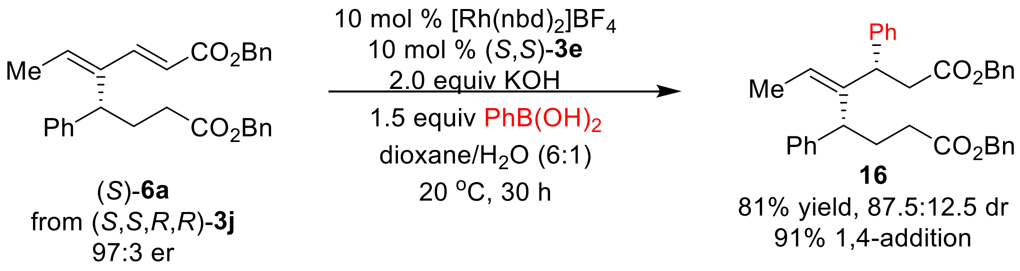

16 was prepared followed previous reported procedure[11]: In a N_2-filled glove-box, an oven-dried vial (8 mL) with a magnetic stir bar was charged with dioxane (1 mL) and H_2O (0.25 mL). Then $[Rh(nbd)_2]BF_4$ (3.7 mg, 0.01 mmol, 10 mol%) and (*S,S*)-**3e** (4.3 mg, 0.01 mmol, 10 mol%) was added to the solution. The mixture was allowed to stir at rt for 0.5 h. Then **(*S*)-6a** (45.5 mg, 0.1 mmol, 1.0 equiv), $PhB(OH)_2$ (42.7 mg, 0.35 mmol, 1.5 equiv), KOH (11.2 mg, 0.2 mmol, 2.0 equiv) and dioxane (0.5 mL) were added respectively. The mixture was heated to 20 ^{o}C for 30 h. Upon completion, the reaction was quenched by H_2O (5 mL) and diluted with EA (5 mL). The layer was separated and the aqueous layer was extracted with EA (5 mL × 2). The combined layers were concentrated, purified by column chromatography (PE:EA = 15:1) to give the addition product **16** as colorless oil (43.2 mg, 81% yield, 87.5:12.5 dr, 91% 1,4-addtion).

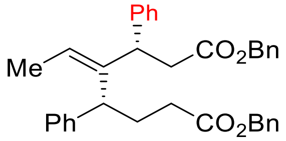

16

Dibenzyl (3*S*,5*R*,*E*)-4-ethylidene-3,5-diphenyloctanedioate (16)

Colorless oil, 74% yield, 87.5:12.5 dr. **IR (neat):** 3061 (w), 3030 (w), 2940 (w), 1733 (m), 1601 (w), 1495 (w), 1452 (w), 1381 (w), 1307 (w), 1247 (w), 1214 (w), 1149 (m), 1081 (w), 1027 (w), 970 (w), 909 (w), 802 (w), 751 (m), 698 (s), 625 (w) cm^{-1}; **^{1}H NMR (400 MHz, CDCl$_3$)** δ 7.41 – 7.27 (m, 10H), 7.23 – 7.15 (m, 4H), 7.08 – 6.90 (m, 7H), 6.70 – 6.69 (m, 2H), 5.65 (q, *J* = 6.8 Hz, 1H), 5.16 – 5.01 (m, 3H), 4.92 (d, *J* = 12.0 Hz, 1H), 3.95 – 3.87 (m, 1H), 3.76 (t, *J* = 8.4 Hz, 1H), 2.80 – 2.67 (m, 2H), 2.38 – 2.28 (m, 3H), 2.24 – 2.13 (m, 1H), 1.78 (d, *J* = 6.8 Hz, 3H); **^{13}C NMR (100 MHz, CDCl$_3$)** δ 173.3, 171.6, 143.1, 142.9, 141.2, 136.0, 135.9, 128.5, 128.41, 128.37, 128.22, 128.16, 128.1, 128.0, 127.9, 127.7, 127.2, 126.0, 125.7, 122.0, 66.1, 66.1, 44.8, 43.6, 41.8, 32.3, 26.5, 13.8; **HRMS (ESI) [M+Na]$^+$** Calcd for $C_{36}H_{36}O_4Na$: 555.25058 m/z, Found: 555.25091 m/z; **Specific rotation**: $[\alpha]_D^{26.3}$ +23.5 (*c* 1.00, $CHCl_3$) for an enantiomerically enriched sample of 87.5:12.5 dr.

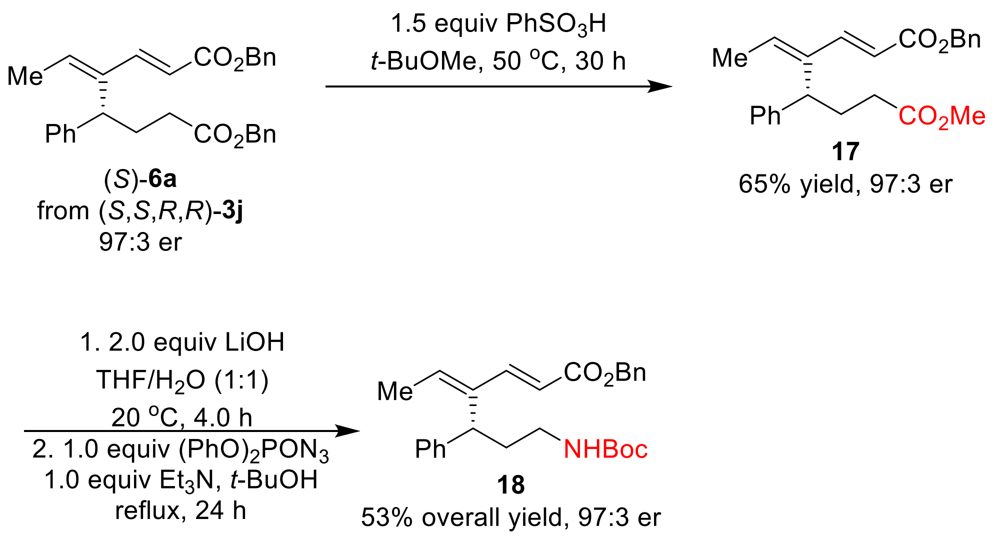

In glovebox, **(*S*)-6a** (0.4786 g, 1.0529 mmol, 1 equiv) and $PhSO_3H$ (0.2498 g, 1.5793 mmol, 1.5 equiv) were dissolved in dry MTBE (0.25M, 4.2ml). The reaction mixture was allowed to stir at 50 °C for 30 h. Then the volatiles were then removed under reduced pressure. The residue was purified by column chromatography (PE:EA = 20:1) to give the product **17** as colorless oil (0.2590 g, 65% yield, 97:3 er).

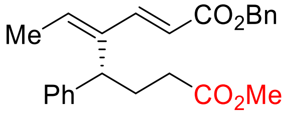

17

1-benzyl 8-methyl (S,2E,4Z)-4-ethylidene-5-phenyloct-2-enedioate (17)

Colorless oil, 65% yield. **IR (neat):** 3030 (w), 2952 (w), 1734 (m), 1713 (m), 1620 (w), 1496 (w), 1440 (w), 1374 (w), 1304 (w), 1253 (w), 1163 (s), 1088 (w), 1013 (w), 984 (w), 912 (w), 863 (w), 823 (w), 740 (m), 698 (m), 648 (w), 615 (w); **^{1}H NMR (400 MHz, $CDCl_3$)** δ 7.36 – 7.26 (m, 6H), 7.25 – 7.13 (m, 5H), 6.19 (q, *J* = 7.2 Hz, 1H), 5.78 (d, *J* = 16.0 Hz, 1H), 5.18 – 5.05 (m, 2H), 4.07 (dd, *J* = 10.4, 5.6 Hz, 1H), 3.65 (s, 3H), 2.49 – 2.36 (m, 1H), 2.36 – 2.26 (m, 2H), 2.26 – 2.10 (m, 1H), 1.84 (d, *J* = 7.2 Hz, 3H); **^{13}C NMR (100 MHz, $CDCl_3$)** δ 173.6, 166.9, 146.7, 141.6, 138.9, 136.0, 135.5, 128.5, 128.4, 128.2, 128.1, 127.1, 126.3, 117.1, 66.0, 51.5, 41.3, 31.9, 26.0, 14.7; **HRMS (ESI) $[M+Na]^+$** Calcd for $C_{24}H_{26}O_4Na$: 401.17233 m/z, Found: 401.17251 m/z; **Specific rotation**: $[\alpha]_D^{24.3}$ +37.3 (*c* 1.00, $CHCl_3$) for an enantiomerically enriched sample of 97:3 e.r.

Enantiomeric purity of **17** was determined by HPLC analysis in comparison with authentic racemic material (97:3 e.r. shown; Chiralpak AD-H column, 95:5 hexane/ *i*-PrOH, 0.7 mL/min, 280 nm).

18 was prepared followed previous reported procedure[13-14]: To a 25 mL round bottle flask was added **17** (37.8 mg, 0.1 mmol, 1.0 equiv), LiOH (4.8 mg, 0.2 mmol, 2.0 equiv), THF (1 mL) and H_2O (1 mL). The mixture was allowed to stir at rt for 4 h. Upon completion, the reaction mixture was diluted with H_2O (2 mL) and acidified with 1M HCl to pH = 1. The aqueous layer was extracted with EA (5 mL × 3), dried over $MgSO_4$, filtered, concentrated and purified by column chromatography (PE:EA = 2:1) to give the Carboxylic acid product as colorless oil (27.4 mg, 75% yield).

A two neck-round bottom flask with cold finger reflux condenser and stir bar was charged with the Carboxylic Acid (36.4 mg, 0.1 mmol, 1 equiv), Et_3N (22 µL, 0.1 mmol, 1 equiv), diphenyl azidophosphate (13 µL, 0.1 mmol, 1 equiv) and 2 mL *t*BuOH under N_2 atmosphere. Then the mixture was allowed to reflux for 24 h. Upon completion, the reaction was concentrated in vacuo. Purification of the residue by flash chromatography (PE:EA = 20:1) gave **18** as colorless oil. (31.1 mg, 71% yield, 97:3 e.r.).

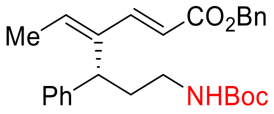

18

Benzyl (S,2E,4Z)-7-((tert-butoxycarbonyl)amino)-4-ethylidene-5-phenylhept-2-enoate (18)

Colorless oil, 71% yield. **IR (neat):** 3388 (br), 3029 (w), 2966 (w), 2930 (w), 1709 (m), 1620 (w), 1514 (w), 1451 (w), 1390 (w), 1366 (w), 1302 (w), 1258 (m), 1163 (s), 1090 (w), 1017 (m), 983 (w), 911 (w), 864 (w), 798 (m), 744 (w), 698 (m), 673 (w), 632 (w) cm^{-1}; **^{1}H NMR (400 MHz, $CDCl_3$)** δ 7.40 – 7.33 (m, 5H), 7.32 – 7.28 (m, 3H), 7.24 – 7.22 (m, 3H), 6.23 (q, *J* = 7.2 Hz, 1H), 5.82 (d, *J* = 16.0 Hz, 1H), 5.21 – 5.09 (m, 2H), 4.57 (s, 1H), 4.12 (dd, *J* = 10.2, 5.6 Hz, 1H), 3.20 – 3.05 (m, 2H), 2.34 – 2.27 (m, 1H), 2.14 – 2.04 (m, 1H), 1.91 (d, *J* = 7.2 Hz, 3H), 1.46 (s, 9H); **^{13}C NMR (100 MHz, $CDCl_3$)** δ 166.9, 155.8, 146.8, 141.7, 139.0, 136.1, 135.3, 128.5, 128.5, 128.2, 128.1, 127.1, 126.3, 117.1, 79.2, 66.1, 39.7, 39.1, 31.3, 28.4, 14.7; **HRMS (ESI) [M+Na]$^+$** Calcd for $C_{27}H_{33}NO_4Na$: 458.23018 m/z, Found: 458.23022 m/z; **Specific rotation**: $[\alpha]_D^{27.1}$ +8.9 (*c* 1.00, $CHCl_3$) for an enantiomerically enriched sample of 97:3 e.r.

Enantiomeric purity of **18** was determined by HPLC analysis in comparison with authentic racemic material (97:3 e.r. shown; Chiralcel OD-H column, 95:5 hexane/ *i*-PrOH, 1.0 mL/min, 208 nm).

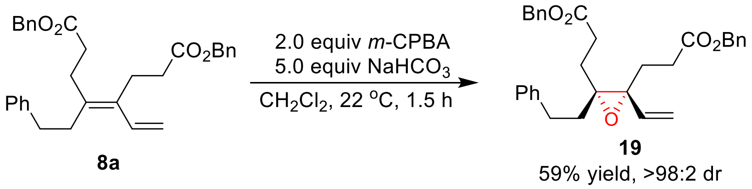

19 was prepared followed previous reported procedure[15]: A 4.0 mL vial with a magnetic stir bar was charged with **8a** (0.10 mmol, 48.3 mg), CH_2Cl_2 (1.0 mL), m-CPBA (0.20 mmol, 34.5 mg) and $NaHCO_3$ (0.50 mmol, 42.0 mg) under 0 °C. Then the reaction mixture was allowed to stir at room temperature until the starting material was consumed. Then the reaction mixture was quenched with NaOH aq. And the aqueous layer was extracted with diethyl ether (3×5 ml), dried over anhydrous Na_2SO_4, concentrated under reduced pressure and purified by flash chromatography on silica gel with petroleum ether and ethyl acetate to afford the colorless oil **19** (29.3 mg, 59% yield).

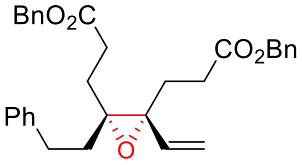

19

Dibenzyl 3,3'-(2-phenethyl-3-vinyloxirane-2,3-diyl)dipropionate (19)

Colorless oil, 59% yield. > 98:2 dr. **IR (neat):** 3030 (w), 2926 (w), 1734 (s), 1603 (w), 1496 (w), 1454 (w), 1421 (w), 1383 (w), 1354 (w), 1283 (w), 1261 (w), 1213 (w), 1163 (s), 1085 (w), 1059 (w), 1026 (w), 995 (w), 929 (w), 908 (w), 747 (m), 698 (s), 676 (w), 624 (w) cm^{-1}; **^{1}H NMR (400 MHz, $CDCl_3$)** δ 7.38 – 7.28 (m, 10H), 7.23 (d, *J* = 7.2 Hz, 2H), 7.19 – 7.13 (m, 1H), 7.11 – 7.07 (m, 2H), 5.63 (dd, *J* = 16.8, 11.2 Hz, 1H), 5.26 (q, *J* = 1.6 Hz, 1H), 5.23 (dd, *J* = 9.2, 1.6 Hz, 1H), 5.13 (s, 2H), 5.10 (s, 2H), 2.68 – 2.56 (m, 2H), 2.55 – 2.49 (m, 2H), 2.47 – 2.42 (m, 2H), 2.14 – 2.02 (m, 3H), 1.96 – 1.83 (m, 2H), 1.72 – 1.64 (m, 1H); **^{13}C NMR (100 MHz, $CDCl_3$)** δ 172.9, 172.7, 141.3, 135.9, 135.8, 134.6, 128.6, 128.5, 128.4, 128.29, 128.25, 128.24, 128.21, 126.0, 118.3, 68.2, 68.1, 66.5, 66.3, 32.7, 31.4, 30.1, 30.1, 26.8, 25.7; **HRMS (ESI) [M+Na]$^+$** Calcd for $C_{32}H_{34}O_5Na$: 521.22985 m/z, Found: 521.23041 m/z.

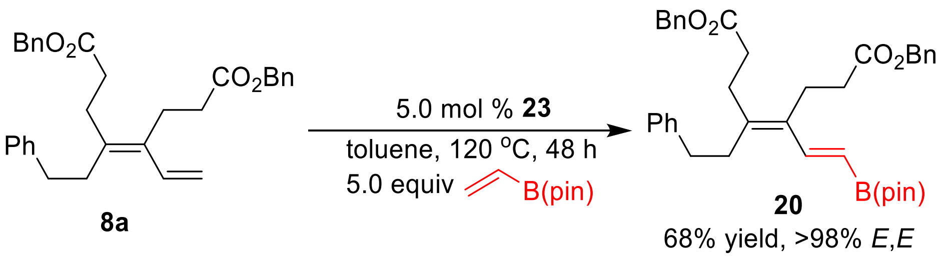

20 was prepared followed previous reported procedure[16]: In a N_2-filled glove-box, an oven-dried vial (8 mL) with a magnetic stir bar was charged with **23** (3.1 mg, 0.005 mmol, 5 mol%) and toluene (1.0 mL). The mixture was allowed to stir at rt for 10 min. Then **8a** (48.3 mg, 0.1 mmol, 1.0 equiv.) and 4,4,5,5-tetramethyl-2-vinyl-1,3,2-dioxaborolane (77.0 mg, 0.5 mmol, 5.0 equiv.) was added to the solution. The vial was sealed with a cap (phenolic open top cap with red PTFE/white silicone septum) and the solution was allowed to stir at 120 °C for 48 h. Upon completion, the reaction was quenched by 0.5 mL saturated aqueous solution of NH_4Cl and extracted with diethyl ether (3×10 mL). The combined organic layer was washed with brine (10 mL) and dried over Na_2SO_4. After the solids were filtered off, the solvent was removed under reduced pressure and the residue was purified by silica-gel column chromatography (eluent: PE/EA = 20 : 1) to afford the **20** as colorless oil (41.1 mg, 68% yield).

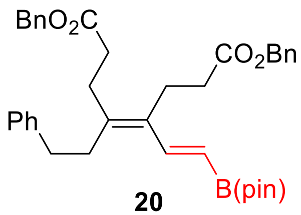

20

Dibenzyl (*E*)-4-phenethyl-5-((*E*)-2-(4,4,5,5-tetramethyl-1,3,2-dioxaborolan-2-yl)vinyl)oct-4-enedioate (20)

Colorless oil, 68% yield. **IR (neat):** 3031 (w), 2976 (w), 1734 (s), 1597 (w), 1496 (w), 1454 (w), 1379 (m), 1345 (s), 1265 (w), 1213 (w), 1145 (s), 1080 (w), 1052 (w), 997 (w), 970 (w), 904 (w), 849 (w), 802 (w), 746 (m), 698 (s), 676 (w), 645 (w) cm^{-1}; **^{1}H NMR (400 MHz, $CDCl_3$)** δ 7.38 – 7.27 (m, 11H), 7.25 – 7.20 (m, 2H), 7.18 – 7.13 (m, 3H), 5.52 (d, J = 18.0 Hz, 1H), 5.08 (s, 4H), 2.70 – 2.57 (m, 4H), 2.52 – 2.45 (m, 4H), 2.45 – 2.33 (m, 4H), 1.27 (s, 12H); **^{13}C NMR (100 MHz, $CDCl_3$)** δ 172.8, 172.5, 145.9, 141.9, 141.4, 136.0, 135.8, 133.2, 128.51, 128.49, 128.4, 128.3, 128.23, 128.21, 128.19, 128.1, 125.9, 115.4, 83.1, 66.4, 66.2, 35.8, 33.8, 33.6, 33.1, 28.8, 24.8, 22.7; **HRMS (ESI) [M+Na]$^+$** Calcd for $C_{38}H_{45}BO_6Na$: 631.32014 m/z, Found: 631.32060 m/z.

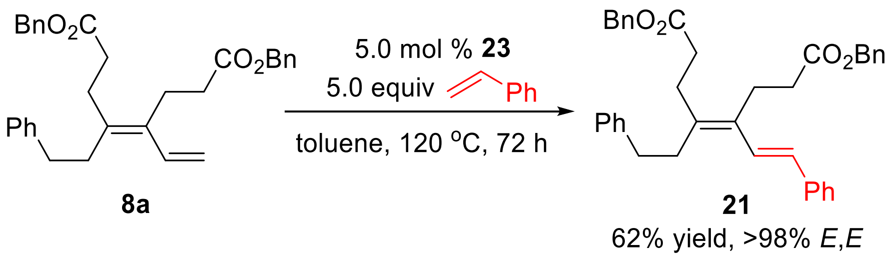

21 was prepared followed previous reported procedure[16]: In a N_2-filled glove-box, an oven-dried vial (8 mL) with a magnetic stir bar was charged with **23** (3.1 mg, 0.005 mmol, 5 mol%) and toluene (1.0 mL). The mixture was allowed to stir at rt for 10 min. Then **8a** (48.3 mg, 0.1 mmol, 1.0 equiv) and styrene (52.1 mg, 0.5 mmol, 5.0 equiv) was added to the solution. The vial was sealed with a cap (phenolic open top cap with red PTFE/white silicone septum) and the solution was allowed to stir at 120 °C for 72 h. Upon completion, the reaction was quenched by 0.5 mL saturated aqueous solution of NH_4Cl and extracted with diethyl ether (3×10 mL). The combined organic layer was washed with brine (10 mL) and dried over Na_2SO_4. After the solids were filtered off, the solvent was removed under reduced pressure and the residue was purified by silica-gel column chromatography (eluent: PE/EA = 20 : 1) to afford the **21** as colorless oil (34.4 mg, 62% yield).

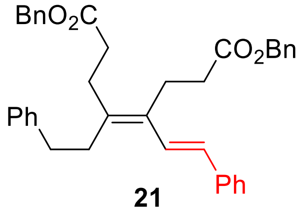

21

Dibenzyl (*E*)-4-phenethyl-5-((*E*)-styryl)oct-4-enedioate (21)

Colorless oil, 62% yield. **IR (neat):** 3029 (w), 2942 (w), 1733 (s), 1597 (w), 1495 (w), 1452 (s), 1380 (w), 1352 (w), 1258 (w), 1231 (w), 1160 (s), 1078 (w), 1053 (w), 1024 (w), 960 (m), 909 (w), 800 (w), 748 (s), 696 (s) cm^{-1}; **^{1}H NMR (400 MHz, $CDCl_3$)** δ 7.38 – 7.33 (m, 8H), 7.33 – 7.26 (m, 7H), 7.25 – 7.23 (m, 1H), 7.23 – 7.18 (m, 1H), 7.18 – 7.13 (m, 3H), 6.92 (d, J = 16.0 Hz, 1H), 6.50 (d, J = 16.0 Hz, 1H), 5.13 (s, 2H), 5.12 (s, 2H), 2.77 – 2.67 (m, 4H), 2.58 – 2.50 (m, 4H), 2.50 – 2.42 (m, 4H); **^{13}C NMR (100 MHz, $CDCl_3$)** δ 173.0, 172.7, 141.5, 138.6, 137.9, 135.9, 135.8, 132.1, 128.6, 128.5, 128.4, 128.3, 128.2, 128.2, 127.7, 127.2, 126.3, 126.1, 125.7, 66.4, 66.3, 35.6, 33.9, 33.8, 33.3, 28.5, 23.4; **HRMS (ESI) [M+Na]$^+$** Calcd for $C_{38}H_{38}O_4Na$: 581.26623 m/z, Found: 581.26692 m/z.

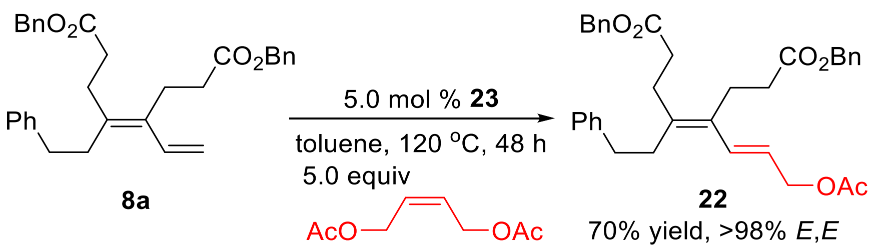

22 was prepared followed previous reported procedure[16]: In a N_2-filled glove-box, an oven-dried vial (8 mL) with a magnetic stir bar was charged with **23** (3.1 mg, 0.005 mmol, 5 mol%) and toluene (1.0 mL). The mixture was allowed to stir at rt for 10 min. Then **8a** (48.3 mg, 0.1 mmol, 1.0 equiv) and (*Z*)-but-2-ene-1,4-diyl diacetate (86.1 mg, 0.5 mmol, 5.0 equiv) was added to the solution. The vial was sealed with a cap (phenolic open top cap with red PTFE/white silicone septum) and the solution was allowed to stir at 120 °C for 48 h. Upon completion, the reaction was quenched by 0.5 mL saturated aqueous solution of NH_4Cl and extracted with diethyl ether (3×10 mL). The combined organic layer was washed with brine (10 mL) and dried over Na_2SO_4. After the solids were filtered off, the solvent was removed under reduced pressure and the residue was purified by silica-gel column chromatography (eluent: PE/EA = 15 : 1) to afford the **22** as colorless oil (38.5 mg, 70% yield).

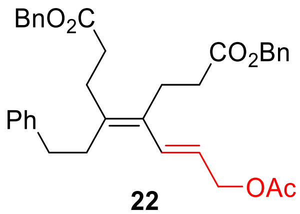

22

Dibenzyl (*E*)-4-((*E*)-3-acetoxyprop-1-en-1-yl)-5-phenethyloct-4-enedioate (22)

Colorless oil, 70% yield. **IR (neat):** 2961 (w), 1734 (s), 1496 (w), 1452 (w), 1380 (w), 1357 (w), 1258 (m), 1230 (m), 1161 (m), 1082 (w), 1022 (m), 962 (w), 799 (m), 748 (m), 698 (m), 677 (w), 628 (w) cm^{-1}; **^{1}H NMR (400 MHz, $CDCl_3$)** δ 7.35 – 7.28 (m, 10H), 7.25 – 7.20 (m, 2H), 7.19 – 7.13 (m, 1H), 7.13 – 7.08 (m, 2H), 6.40 (d, *J* = 15.6 Hz, 1H), 5.66 (dt, *J* = 15.6, 6.4 Hz, 1H), 5.11 (s, 2H), 5.10 (s, 2H), 4.62 – 4.49 (m, 2H), 2.65 – 2.55 (m, 4H), 2.49 – 2.36 (m, 8H), 2.05 (s, 3H); **^{13}C NMR (100 MHz, $CDCl_3$)** δ 172.8, 172.6, 170.7, 141.3, 139.1, 135.9, 135.8, 131.1, 131.0, 128.5, 128.3, 128.3, 128.2, 126.0, 122.1, 66.4, 66.3, 65.5, 35.4, 33.7, 33.6, 33.2, 28.3, 23.5, 21.0; **HRMS (ESI) [M+Na]$^+$** Calcd for $C_{35}H_{38}O_6Na$: 577.25606 m/z, Found: 577.25659 m/z.

1.5 Experimental Procedure and Characterization for Mechanistic Studies

1.5.1 Deuterium Labeling Experiments

All the ratios of deuterium incorporation were determined by comparison of quantitive ^{1}H NMR spectra of non-deuterated compounds and deuterated compounds.

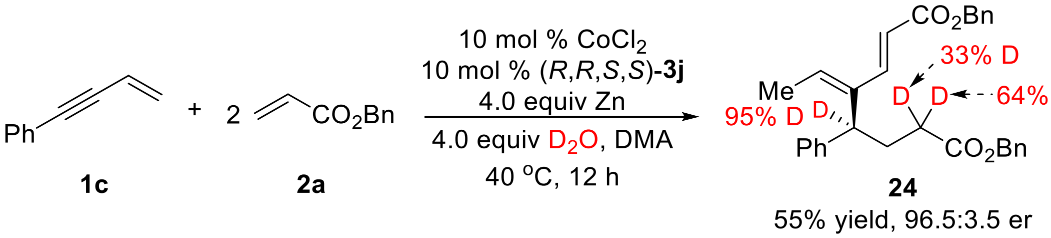

24 was prepared followed general procedure for preparing **6** by replacing H_2O with D_2O. The deuterium-incorporation rate was determined by comparison **24** with **6a.**

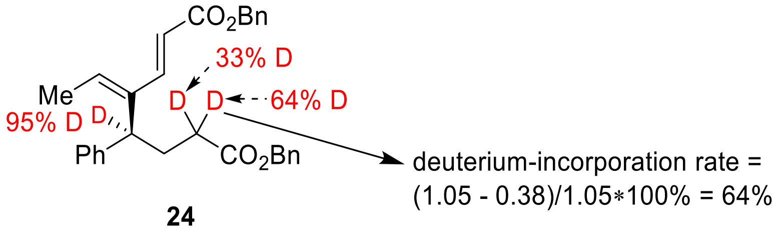

Dibenzyl (2*E*,4*Z*,5*R*)-4-ethylidene-5-phenyloct-2-enedioate-5,7-d_2 (24)

Colorless oil, 55% yield. **IR (neat):** 3032 (w), 2935 (w),1729 (m), 1603 (w), 1496 (w), 1452 (w), 1377 (w), 1283 (w), 1260 (w), 1212 (w), 1164 (m), 1082 (w), 1026 (w), 981 (w), 911 (w), 852 (w), 805 (w), 739 (m), 698 (w), 638 (w); **^{1}H NMR (600 MHz, $CDCl_3$)** δ 7.40 – 7.32 (m, 11H), 7.32 – 7.27 (m, 2H), 7.26 – 7.20 (m, 3H), 6.20 (q, *J* = 7.2 Hz, 1H), 5.82 (d, *J* = 16.2 Hz, 1H), 5.17 – 5.11 (m, 4H), 2.47 (dd, *J* = 13.2, 7.2 Hz, 1H), 2.41 (dd, *J* = 8.4, 5.4 Hz, 0.38H), 2.34 (t, *J* = 7.2 Hz, 0.7H), 2.25 – 2.18 (m, 1H), 1.82 (d, *J* = 7.2 Hz, 3H); **^{13}C NMR (125 MHz, $CDCl_3$)** δ 173.0, 166.9, 146.8, 141.6, 138.8, 136.1, 135.8, 135.6, 128.54, 128.47, 128.3, 128.25, 128.23, 128.1, 127.1, 126.3, 117.1, 66.3, 66.1, 40.9 (t, *J* = 18.9 Hz), 31.8 (t, *J* = 18.9 Hz), 25.8, 14.7; **^{2}H NMR (61 MHz, $CHCl_3$)** δ 7.26, 4.09, 2.40, 2.35 ($CDCl_3$ as internal standard); **HRMS (ESI) [M+Na]$^+$** Calcd for $C_{30}H_{28}O_4NaD_2$: 479.21618 m/z, Found: 479.21545 m/z; **Specific rotation**: $[\alpha]_D^{30.9}$ +17.4 (*c* 1.00, $CHCl_3$) for an enantiomerically enriched sample of 96.5:3.5 e.r.

Enantiomeric purity of **24** was determined by HPLC analysis in comparison with authentic racemic material (96.5:3.5 e.r. shown; Chiralpak IB column, 95:5 hexane/ *i*-PrOH, 1.0 mL/min, 285 nm).

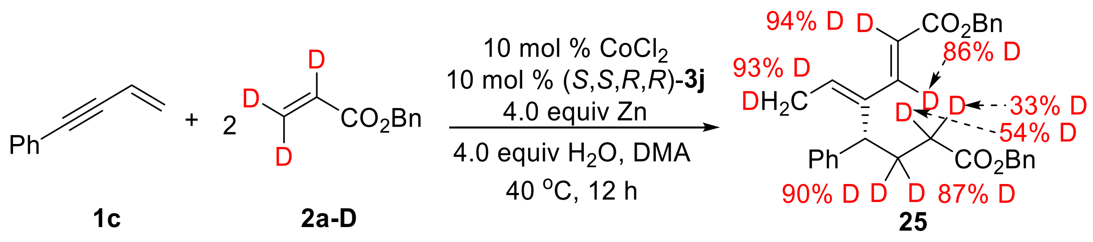

25 was prepared followed general procedure for preparing **6** by replacing **2a** with **2a-D**. The deuterium-incorporation rate was determined by comparison **25** with **6a.**

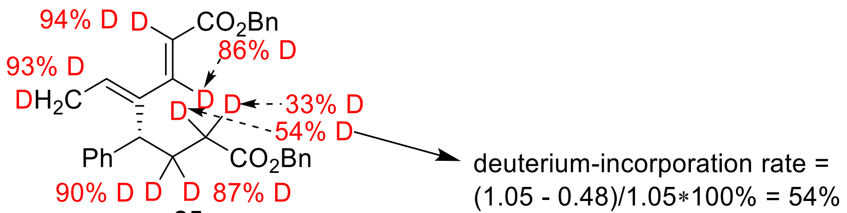

Dibenzyl (2*E*,4*Z*,5*S*)-4-(ethylidene-2-d)-5-phenyloct-2-enedioate-2,3,6,6,7-d$_5$ (25)

Pale yellow oil, 55% yield. **IR (neat):** 3032 (w), 2961 (w), 1732 (m), 1709 (m), 1586 (w), 1495 (w), 1452 (w), 1376 (w), 1257 (m), 1207 (w), 1178 (w), 1086 (m), 1020 (m), 864 (w), 798 (s), 748 (w), 698 (m), 626 (w) cm^{-1}; **^{1}H NMR (600 MHz, CDCl$_3$)** δ 7.40 – 7.34 (m, 10H), 7.31 (m, 3H), 7.33 – 7.29 (m, 2H), 6.21 (t, J = 7.2 Hz, 1H), 5.81 (s, 0.06H), 5.18 – 5.12 (m, 4H), 4.08 (s, 1H), 2.47 (t, J = 7.2 Hz, 0.1H), 2.40 (s, 0.71H), 2.34 (s, 0.48H), 2.21 (dd, J = 10.4, 7.2 Hz, 0.14H), 1.84 – 1.80 (m, 2H); **^{1}H NMR (600 MHz, Acetone-*d*$_6$)** δ 7.39 – 7.34 (m, 8H), 7.33 – 7.28 (m, 6H), 7.21 – 7.18 (m, 1.14H), 6.32 – 6.27 (m, 1H), 5.82 (s, 0.06H), 5.15 – 5.06 (m, 4H), 4.18 (s, 1H), 2.48 – 2.33 (m, 1.21H), 2.28 (t, J = 7.2 Hz, 0.10H), 1.85 – 1.83 (m, 2.07H); **^{13}C NMR (100 MHz, CDCl$_3$)** δ 173.1, 166.9, 146.4 (t, J = 20.0 Hz), 141.6, 138.8, 136.1, 135.9, 135.6, 128.6, 128.5, 128.3, 128.3, 128.2, 128.1, 127.1, 126.3, 116.7 (t, J = 20.0 Hz), 66.3, 66.1, 41.2, 41.1, 31.7 (m), 25.4, 14.4 (m); **^{2}H NMR (61 MHz, CHCl$_3$)** δ 7.26, 5.81, 2.48, 2.41, 2.34, 2.21,1.82 (CDCl$_3$ as internal standard); **^{2}H NMR (61 MHz, CHCl$_3$)** δ 7.21, 5.81, 2.48, 2.40, 2.34, 2.21, 1.81. (no internal standard); **HRMS (ESI) [M+Na]$^+$** Calcd for $C_{30}H_{24}O_4NaD_6$: 483.24283 m/z, Found: 483.24216 m/z; **Specific rotation**: $[\alpha]_D^{27.2}$ -18.0 (*c* 1.00, CHCl$_3$) for an enantiomerically enriched sample of 98:2 e.r.

Enantiomeric purity of **25** was determined by HPLC analysis in comparison with authentic racemic material (98:2 e.r. shown; Chiralpak IG column, 95:5 hexane/ *i*-PrOH, 1.0 mL/min, 254 nm).

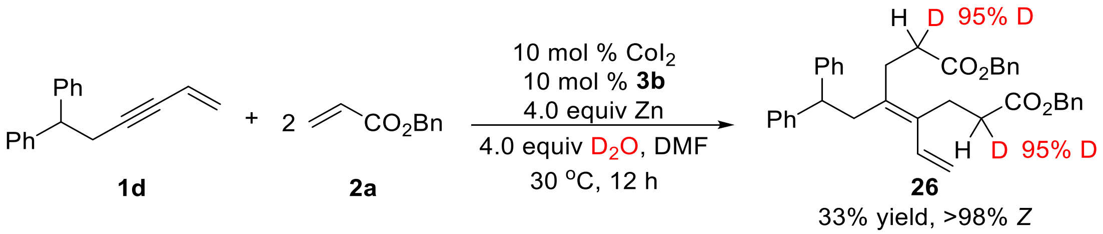

26 was prepared followed general procedure for preparing **8** by replacing **1b** and H_2O with **1d** and D_2O. The deuterium-incorporation rate was determined by comparison **26** with **8p.**

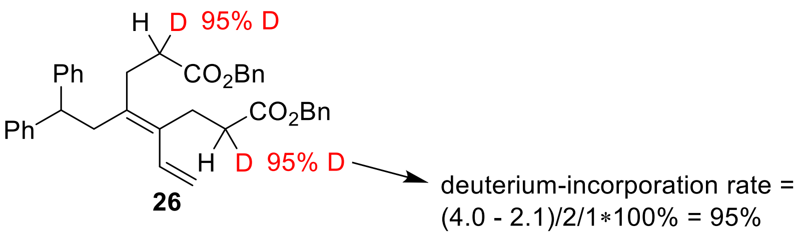

Dibenzyl (*Z*)-4-(2,2-diphenylethyl)-5-vinyloct-4-enedioate-2,7-d$_2$ (26)

Colorless oil, 33% yield. **IR (neat):** 3063 (w), 3029 (w), 2954 (w), 1732 (m), 1599 (w), 1494 (w), 1452 (w), 1377 (w), 1341 (w), 1262 (w), 1228 (w), 1163 (m), 1077 (w), 1028 (w), 988 (w), 905 (w), 788 (w), 747 (m), 698 (s), 639 (w) cm^{-1}; **^{1}H NMR (400 MHz, CDCl$_3$)** δ 7.36 – 7.26 (m, 10H), 7.21 – 7.17 (m, 4H), 7.15 – 7.08 (m, 6H), 6.34 (dd, *J* = 17.2, 11.2 Hz, 1H), 5.14 – 5.01 (m, 5H), 4.89 (dd, *J* = 11.2, 1.2 Hz, 1H), 3.99 (t, *J* = 7.6 Hz, 1H), 2.83 (d, *J* = 7.6 Hz, 2H), 2.49 (d, *J* = 8.4 Hz, 2H), 2.26 (q, *J* = 7.6 Hz, 2.10H), 2.10 (d, *J* = 8.0 Hz, 2H); **^{13}C NMR (100 MHz, CDCl$_3$)** δ 173.0, 172.7, 144.1, 136.7, 136.0, 135.9, 133.3, 133.2, 128.55, 128.52, 128.3, 128.23, 128.20, 128.0, 126.3, 113.1, 66.3, 66.2, 50.6, 37.0, 33.3(t, *J* = 20.2 Hz), 32.8(t, *J* = 20.2 Hz), 28.2, 22.9; **^{2}H NMR (61 MHz, CHCl$_3$)** δ 7.26, 2.27 (CDCl$_3$ as internal standard); **HRMS (ESI) [M+Na]$^+$** Calcd for $C_{38}H_{36}O_4NaD_2$: 583.27878 m/z, Found: 583.27802 m/z.

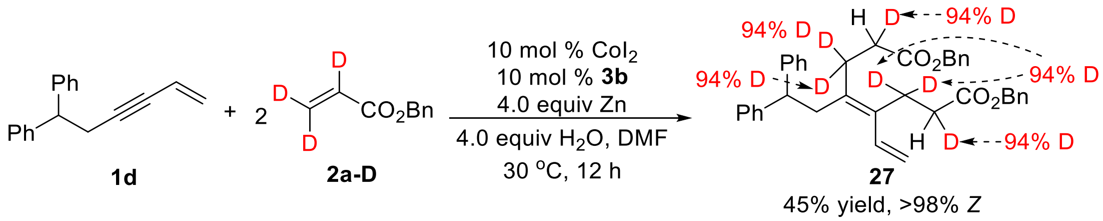

27 was prepared followed general procedure for preparing **8** by replacing **1b** and **2a** with **1d** and **2a-D**. The deuterium-incorporation rate was determined by comparison **27** with **8p.**

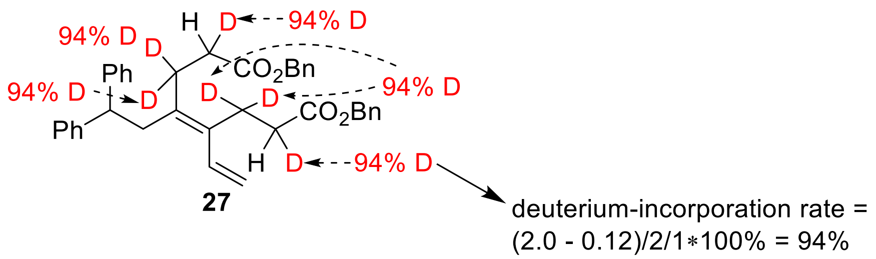

Dibenzyl (*Z*)-4-(2,2-diphenylethyl)-5-vinyloct-4-enedioate-2,3,3,6,6,7-d$_6$ (27)

Colorless oil, 45% yield. **IR (neat):** 3062 (w), 3028 (w), 2931 (w), 1732 (s), 1600 (w), 1494 (w), 1452 (w), 1377 (w), 1318 (w), 1256 (w), 1172 (s), 1082 (w), 1024 (w), 905 (w), 805 (w), 747 (s), 698 (s), 625 (w) cm^{-1}; **^{1}H NMR (400 MHz, CDCl$_3$)** δ 7.38 – 7.30 (m, 10H), 7.25 – 7.21 (m, 4H), 7.19 – 7.14 (m, 6H), 6.39 (dd, *J* = 17.4, 11.2 Hz, 1H), 5.18 – 5.04 (m, 5H), 4.94 (dd, *J* = 11.2, 1.2 Hz, 1H), 4.04 (t, *J* = 7.6 Hz, 1H), 2.88 (d, *J* = 7.6 Hz, 2H), 2.53 – 2.50 (m, 0.12H), 2.30 (dd, *J* = 7.2, 4.8 Hz, 2.12H), 2.14 – 2.12 (m, 0.12H); **^{13}C NMR (100 MHz, CDCl$_3$)** δ 173.0, 172.6, 144.1, 136.6, 135.9, 135.8, 133.2, 133.2, 128.53, 128.50, 128.3, 128.23, 128.18, 128.16, 128.0, 126.3, 113.1, 66.2, 66.2, 50.6, 36.9, 33.2(t, *J* = 20.2 Hz), 32.6(t, *J* = 20.2 Hz), 27.6(m), 22.6(m); **^{2}H NMR (61 MHz, CHCl$_3$)** δ 7.26, 2.51, 2.30, 2.12, 1.96 (CDCl$_3$ as internal standard); **HRMS (ESI) [M+Na]$^+$** Calcd for $C_{38}H_{32}O_4NaD_6$: 587.30388 m/z, Found: 587.30319 m/z.

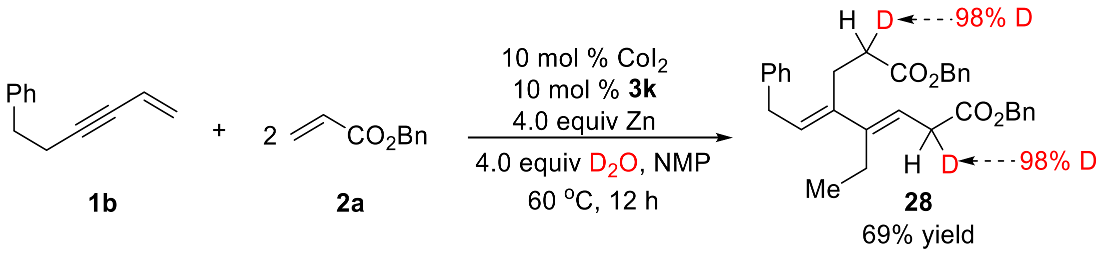

28 was prepared followed general procedure for preparing **10** by replacing H_2O with D_2O. The deuterium-incorporation rate was determined by comparison **28** with **10a.**

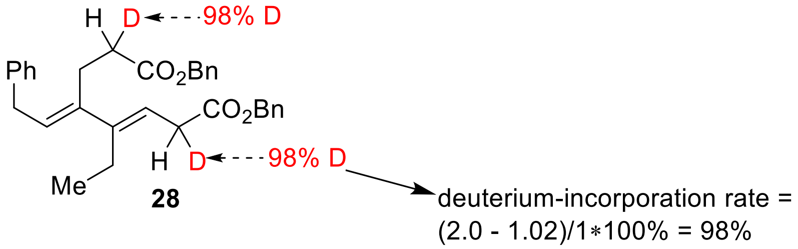

Dibenzyl (3*E*,5*E*)-4-ethyl-5-(2-phenylethylidene)oct-3-enedioate-2,7-d$_2$ (28)

Pale yellow oil, 69% yield. **IR (neat):** 3941 (w), 3861 (w), 3032 (w), 2963 (w), 1733 (s), 1603 (w), 1495 (w), 1454 (w), 1376 (w), 1260 (m), 1215 (w), 1161 (m), 1082 (m), 1018 (m), 864

(w), 797 (s), 740 (m), 698 (s) cm^{-1}; **^{1}H NMR (400 MHz, CDCl$_3$)** δ 7.37 – 7.27 (m, 12H), 7.23 – 7.14 (m, 3H), 5.67 (t, *J* = 7.2 Hz, 1H), 5.58 (d, *J* = 7.2 Hz, 1H), 5.14 (s, 2H), 5.12 (s, 2H), 3.48 (d, *J* = 7.2 Hz, 2H), 3.17 (d, *J* = 7.2 Hz, 1.02H), 2.67 (d, *J* = 8.0 Hz, 2H), 2.39 (t, *J* = 8.0 Hz, 1.02H), 2.21 (q, *J* = 7.6 Hz, 2H), 0.93 (t, *J* = 7.6 Hz, 3H); **^{13}C NMR (100 MHz, CDCl$_3$)** δ 173.0, 171.7, 145.2, 140.9, 139.3, 136.0, 135.9, 128.5, 128.4, 128.3, 128.2, 128.2, 128.1, 126.9, 125.9, 117.2, 66.4, 66.2, 34.3, 33.5 (t, *J* = 20.0 Hz), 33.0 (t, *J* = 20.0 Hz), 23.2, 21.5, 13.2; ^{2}H NMR (61 MHz, CHCl$_3$) δ 7.26, 3.17, 2.38 (CDCl3 as internal standard); **HRMS (ESI) [M+Na]$^+$** Calcd for $C_{32}H_{32}O_4NaD_2$: 507.24748 m/z, Found: 507.24773 m/z;

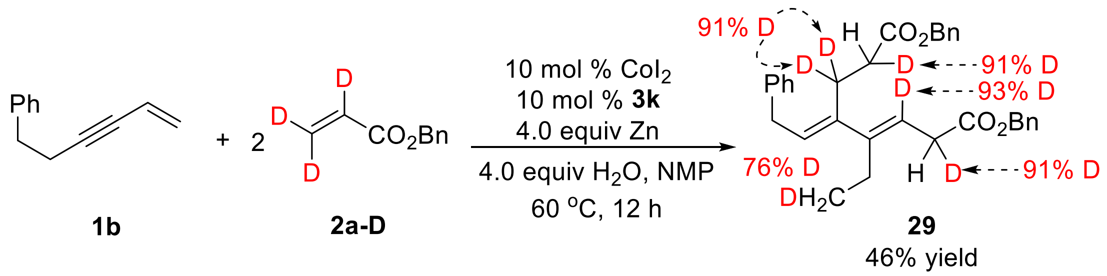

29 was prepared followed general procedure for preparing **10** by replacing **2a** with **2a-D**. The deuterium-incorporation rate was determined by comparison **29** with **10a.**

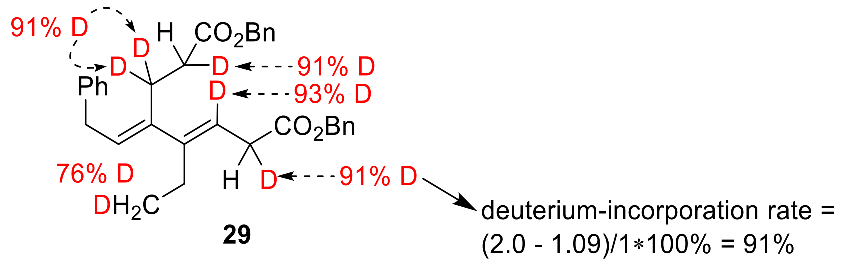

Dibenzyl (3*E*,5*E*)-4-(ethyl-2-d)-5-(2-phenylethylidene)oct-3-enedioate-2,3,6,6,7-d$_5$ (29)

Colorless oil, 46% yield. **IR (neat):** 3064 (w), 3030 (w), 2959 (w), 1733 (s), 1603 (w), 1495 (w),1454 (w), 1376 (w), 1307 (w), 1259 (w), 1214 (w), 1170 (m), 1078 (w), 1012 (w), 907 (w), 804 (w), 740 (m), 698 (s), 648 (w) cm^{-1}; **^{1}H NMR (400 MHz, CDCl$_3$)** δ 7.37 – 7.28 (m, 12H), 7.23 – 7.17 (m, 3H), 5.68 (t, *J* = 7.2 Hz, 1H), 5.59 (d, *J* = 8.0 Hz, 0.07H),5.14 (d, *J* = 1.2 Hz, 2H), 5.12 (s, 2H), 3.48 (d, *J* = 7.2 Hz, 2H), 3.18 (d, *J* = 8.4 Hz, 1.09H), 2.65 (d, *J* = 8.0 Hz, 0.17H), 2.40 (d, *J* = 6.8 Hz, 1.09H), 2.20 (t, *J* = 7.6 Hz, 2H), 0.92 (d, *J* = 7.6 Hz, 2H); **^{13}C NMR (100 MHz, CDCl$_3$)** δ 173.0, 171.7, 145.0, 140.9, 139.1, 135.9, 135.9, 128.5, 128.4, 128.3, 128.22, 128.17, 128.16, 128.1, 126.8, 125.9, 116.8 (t, *J* = 20.2 Hz), 66.4, 66.2, 34.3, 33.4 (t, *J* = 20.2 Hz), 33.0, 32.8 (t, *J* = 20.2 Hz), 32.6, 23.0 –22.4 (m), 21.3, 12.9 (t, *J* = 20.2 Hz).**^{2}H NMR (61 MHz,**

$CHCl_3$) δ 7.26, 5.58, 3.18, 2.64, 2.41, 0.91($CDCl_3$ as internal standard); **HRMS (ESI) $[M+Na]^+$** Calcd for $C_{32}H_{28}O_4NaD_6$: 511.27258 m/z, Found: 511.27255 m/z.

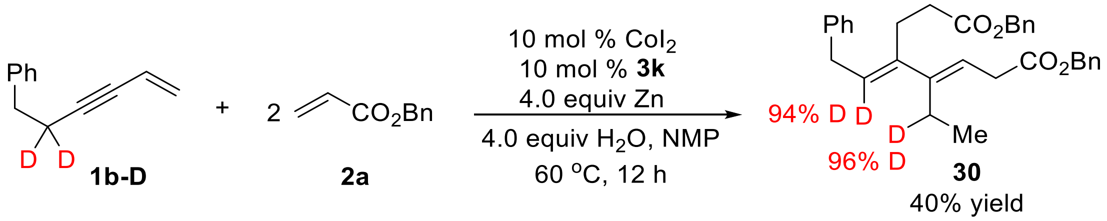

30 was prepared followed general procedure for preparing **10** by replacing **1b** with **1b-D**. The deuterium-incorporation rate was determined by comparison **30** with **10a.**

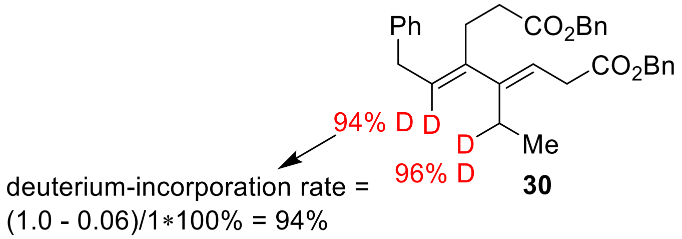

Dibenzyl (3*E*,5*E*)-4-(ethyl-1-d)-5-(2-phenylethylidene-1-d)oct-3-enedioate (30)

Colorless oil, 40% yield. **IR (neat):** 3030 (w), 2961 (w), 2925 (w), 1734 (s), 1603 (w), 1496 (w), 1454 (w), 1379 (w), 1259 (w), 1213 (w), 1156 (m), 1080 (w), 1026 (w), 976 (w), 911 (w), 803 (w), 742 (m), 698 (s), 639 (w) cm^{-1}; **^1H NMR (600 MHz, $CDCl_3$)** δ 7.39 – 7.31 (m, 10H), 7.31 – 7.27 (m, 2H), 7.22 – 7.15 (m, 3H), 5.71 (t, J = 7.2 Hz, 0.06H), 5.59 (t, J = 7.2 Hz, 1H), 5.13 (s, 2H), 5.11 (s, 2H), 3.47 (s, 2H), 3.19 (d, J = 7.2 Hz, 2H), 2.72 – 2.62 (m, 2H), 2.44 – 2.39 (m, 2H), 2.18 (q, J = 7.8 Hz, 1H), 0.91 (d, J = 7.8 Hz, 3H); **^{13}C NMR (100 MHz, $CDCl_3$)** δ 173.0, 171.8, 145.0, 140.9, 139.1, 135.9, 135.8, 128.5, 128.4, 128.3, 128.24, 128.20, 128.18, 128.15, 126.7 (t, J = 20.2 Hz), 117.2, 66.4, 66.2, 34.2, 33.8, 33.3, 23.2, 21.1 (t, J = 20.2 Hz), 13.1; **^2H NMR (61 MHz, $CHCl_3$)** δ 7.26, 5.70, 2.18 ($CDCl_3$ as internal standard); **HRMS (ESI) $[M+Na]^+$** Calcd for $C_{32}H_{32}$ O_4NaD_2: 507.24748 m/z, Found: 507.24805 m/z.

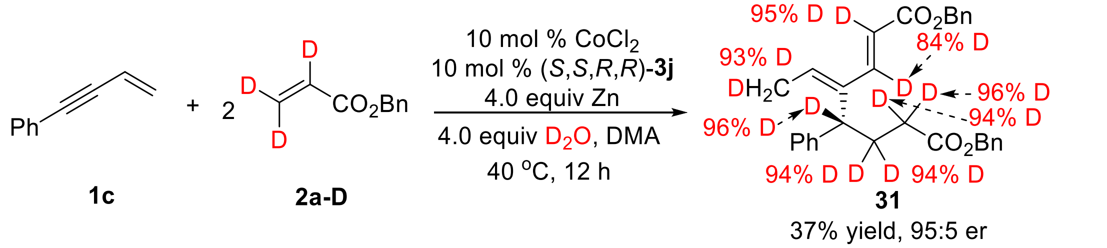

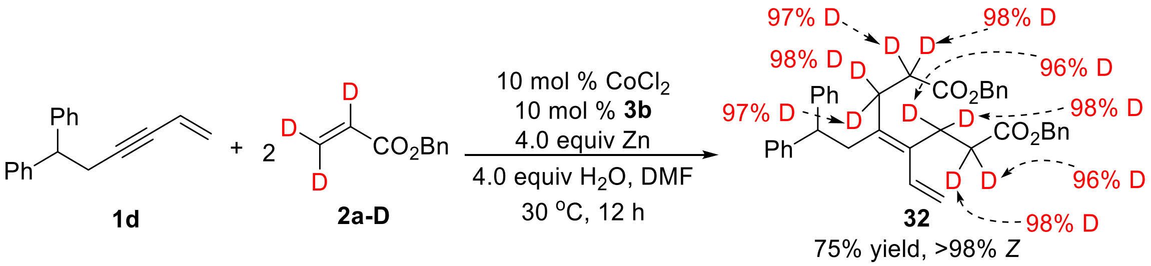

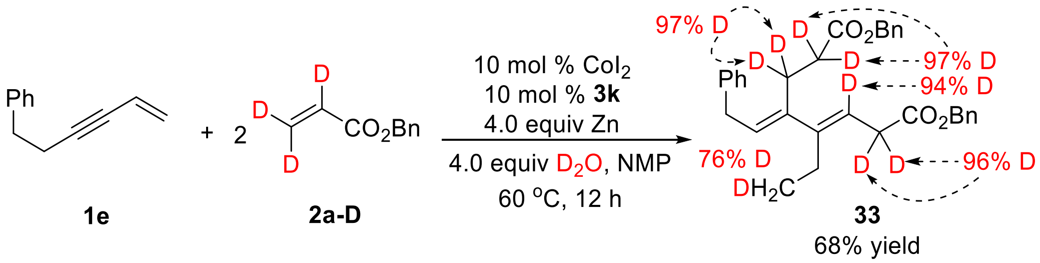

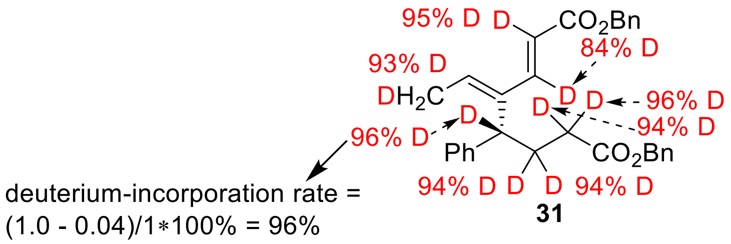

31 was prepared followed general procedure for preparing **6** by replacing **2a** and H_2O with **2a-D** and D_2O. The deuterium-incorporation rate was determined by comparison **31** with **6a.**

Dibenzyl (*S*,2*E*,4*Z*)-4-(ethylidene-2-d)-5-phenyloct-2-enedioate-2,3,5,6,6,7,7-d$_7$ (31)

Colorless oil, 37% yield. **IR (neat):** 3063 (w), 3030 (w), 2932 (w), 1729 (s), 1586 (w), 1495 (w), 1452 (w), 1375 (w), 1260 (s), 1178 (w), 1076 (m), 1027 (w), 911 (w), 803 (w), 742 (m), 699 (s), 632 (w) cm^{-1}; **^{1}H NMR (600 MHz, CDCl3)** δ 7.37 – 7.31 (m, 10H), 7.31 – 7.28 (m, 2H), 7.24 – 7.17 (m, 3H), 6.18 (t, *J* = 4.4 Hz, 1H), 5.79 (s, 0.05H), 5.16 – 5.09 (m, 4H), 4.05 (s, 0.04H), 2.43 (s, 0.06H), 2.37 (s, 0.04H), 2.31 (s, 0.05H), 2.17 (s, 0.06H), 1.82 – 1.77 (m, 2.07H). **^{1}H NMR (600 MHz, Acetone-*d*$_6$)** δ 7.39 – 7.34 (m, 8H), 7.33 – 7.28 (m, 6H), 7.21 – 7.18 (m, 1.14H), 6.29 (t, *J* = 7.6 Hz, 1H), 5.83 (s, 0.05H), 5.17 – 5.04 (m, 4H), 4.18 (s, 0.04H), 2.45 – 2.36 (m, 0.16H), 2.17 (s, 0.06H), 1.86 – 1.83 (m, 2.07H); **^{13}C NMR (100 MHz, CDCl3)** δ 173.1, 166.9, 146.4 (t, *J* =

20.2 Hz), 141.6, 138.7, 136.1, 135.9, 135.5, 128.6, 128.5, 128.3, 128.3, 128.2, 128.1, 127.1, 126.3, 116.7 (t, J = 20.2 Hz), 66.3, 66.1, 40.8 (t, J = 20.2 Hz), 31.5 (m), 25.3, 14.4 (t, J = 20.2 Hz); **^{2}H NMR (61 MHz, $CHCl_3$)** δ 7.26, 5.79, 4.05, 2.44, 2.37, 2.32, 2.18, 1.81 ($CDCl_3$ as internal standard); **^{2}H NMR (61 MHz, CDCl3)** δ 7.19, 5.80, 4.05, 2.44, 2.37, 2.32, 2.18, 1.81 (no internal standard); **HRMS (ESI) [M+Na]$^+$** Calcd for $C_{30}H_{22}O_4NaD_8$: 485.25383 m/z, Found: 485.25288 m/z; **Specific rotation**: $[\alpha]_D^{27.2}$ -17.9 (c 1.00, $CHCl_3$) for an enantiomerically enriched sample of 95:5 e.r.

Enantiomeric purity of **31** was determined by HPLC analysis in comparison with authentic racemic material (95:5 e.r. shown; Chiralpak IBN-5 column, 95:5 hexane/ *i*-PrOH, 1.0 mL/min, 254 nm).

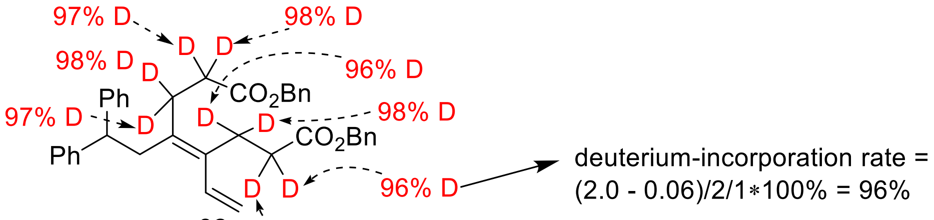

32

32 was prepared followed general procedure for preparing **8** by replacing **1b**, **2a** and H_2O with **2b**, **2a-D** and D_2O. The deuterium-incorporation rate was determined by comparison **32** with **8p.**

Dibenzyl (*Z*)-4-(2,2-diphenylethyl)-5-vinyloct-4-enedioate-2,2,3,3,6,6,7,7-d$_8$ (32)

Colorless oil, 75% yield. **IR (neat):** 3029 (w), 2962 (w), 1731 (m), 600 (w), 1494 (w), 1452 (w), 1375 (w), 1258 (s), 1140 (w), 1085 (w), 1026 (w), 906 (w), 796 (w), 746 (m), 698 (s), 674 (w) cm^{-1}; **^{1}H NMR (400 MHz, $CDCl_3$)** δ 7.45 – 7.32 (m, 11H), 7.29 (d, J = 8.0 Hz, 3H), 7.21 (d, J = 7.2 Hz, 6H), 6.44 (dd, J = 17.2, 11.2 Hz, 1H), 5.18 – 5.11 (m, 5H), 4.98 (d, J = 11.2 Hz, 1H), 4.08 (t, J = 7.6 Hz, 1H), 2.91 (d, J = 7.6 Hz, 2H), 2.55 (s, 0.08H), 2.33 (d, J = 4.0 Hz, 0.08H) , 2.16 (s, 0.06H). **^{13}C NMR (100 MHz, $CDCl_3$)** δ 173.0, 172.6, 144.1, 136.6, 135.9, 135.8, 133.19, 133.16, 128.52, 128.50, 128.3, 128.21, 128.17, 128.1, 128.0, 126.3, 113.1, 66.2, 66.2, 50.6, 36.9, 33.1(m), 32.3(m), 27.6(m), 22.0(m); **^{2}H NMR (61 MHz, $CHCl_3$)** δ 7.26, 2.55, 2.33, 2.16 ($CDCl_3$ as internal standard); **HRMS (ESI) [M+Na]$^+$** Calcd for $C_{38}H_{30}O_4NaD_8$: 589.31643 m/z, Found: 589.31657 m/z.

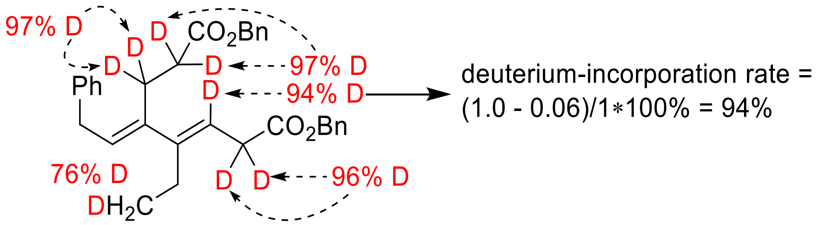

33

33 was prepared followed general procedure for preparing **10** by replacing **2a** and H_2O with **2a-D** and D_2O. The deuterium-incorporation rate was determined by comparison **33** with **10a.**

Dibenzyl (3*E*,5*E*)-4-(ethyl-2-d)-5-(2-phenylethylidene)oct-3-enedioate-2,2,3,6,6,7,7-d$_7$ (33)

Colorless oil, 68% yield. **IR (neat):** 3628 (w), 3031 (w), 2960 (w), 1732 (s), 1602 (w), 1495 (w), 1454 (w), 1374 (w), 1258 (s), 1161 (w), 1109 (w), 1068 (w), 1024 (w), 978 (w), 803 (w), 738 (m), 697 (s), 652 (w) cm^{-1}; **^{1}H NMR (400 MHz, $CDCl_3$)** δ 7.39 – 7.33 (m, 11H), 7.29 – 7.27 (m, 1H), 7.22 – 7.14 (m, 3H), 5.67 (t, *J* = 7.4 Hz, 1H), 5.57 (s, 0.06H), 5.13 (s, 2H), 5.11 (s, 2H), 3.47 (d, *J* = 7.2 Hz, 2H), 3.16 (s, 0.08H), 2.63 (s, 0.06H), 2.37 (s, 0.07H), 2.19 (t, *J* = 7.6 Hz, 2H), 0.93 – 0.87 (m, 2.24H); **^{13}C NMR (100 MHz, $CDCl_3$)** δ 173.0, 171.7, 145.1, 140.9, 139.1, 139.1, 136.0, 135.9, 128.5, 128.4, 128.3, 128.2, 128.2, 128.1, 128.0, 126.8, 125.9, 116.8 (t, *J* = 20.0 Hz), 116.5, 34.3, 33.5 (m), 33.1(m), 23.0 (m), 21.3, 12.9 (t, *J* = 20.2 Hz). **^{2}H NMR (61 MHz, $CHCl_3$)** δ 7.26, 5.58, 3.17, 2.62, 2.38, 0.89 ($CDCl_3$ as internal standard); **HRMS (ESI) $[M+Na]^+$** Calcd for $C_{32}H_{26}O_4NaD_8$: 513.28513 m/z, Found: 513.28450 m/z.

1.5.2 EPR Studies

1.5.2.1 EPR Studies of co-catalyzed coupling of 1,3-enynes and acrylates to afford 1,3-dienes bearing a tetrasubstituted alkene

Electron paramagnetic resonance (EPR) spectra were recorded at room temperature on a Bruker E500-10/12.

(a) Background of co-catalyzed coupling of 1,3-enyne **1e** and acrylate **2a** to afford 1,3-dienes bearing a tetrasubstituted alkene

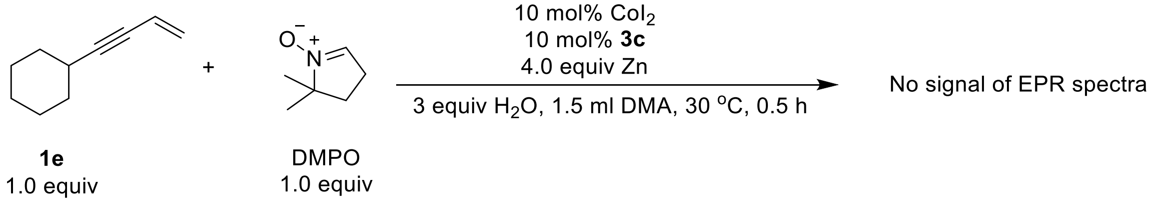

In a N_2-filled glove box, an oven-dried 8-mL vial equipped with a stirring bar was charged with CoI_2 (6.3 mg, 0.02 mmol, 10 mol%), **3c** (11.9 mg, 0.02 mmol, 10 mol%), zinc powder (52.3 mg, 0.8 mmol, 4 eq) and DMA (1.3 mL). The vial was sealed with a cap (phenolic cap with red PTFE/white silicone septum) and the solution was allowed to stir at room temperature for 30 min. **1e** (26.8 mg, 0.2 mmol, 1.0 equiv.), DMPO (22.6 mg, 0.2 mmol, 1.0 equiv.), H_2O (10.8 mg, 0.6 mmol, 3.0 equiv.) and DMA (0.2 mL) were added to the solution. Then the vial was sealed with a cap (phenolic open top cap with red PTFE/white silicone septum), removed from the glove box. The mixture was immediately moved to a thermostatic bath and allowed to stir at 30 ºC for 0.5 h. The reaction mixture was transferred to quartz EPR tube and the spectra was measured at room temperature (**Supplementary Figure 1.**). No signal was observed in the EPR spectra, which indicated that no radical species in the reaction in the absence of **2a**.

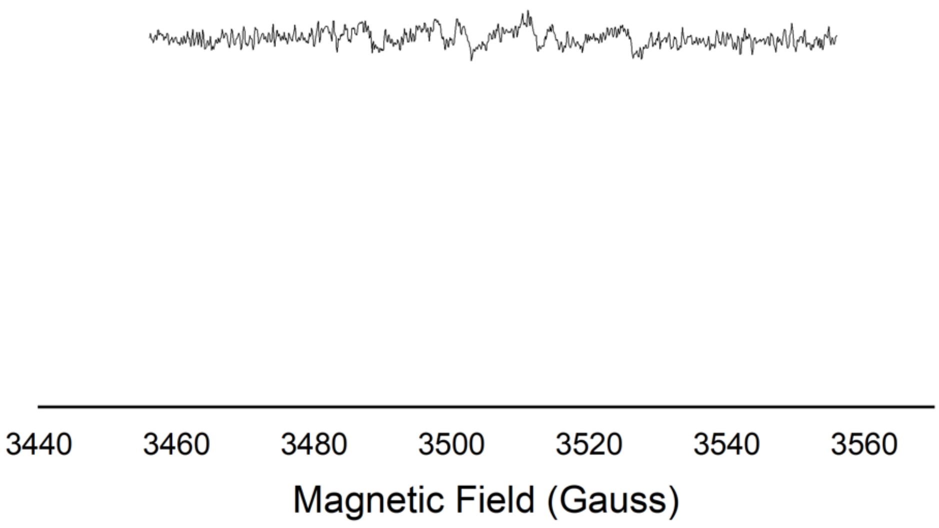

Supplementary Figure 1. EPR spectra in the absence of **2a**

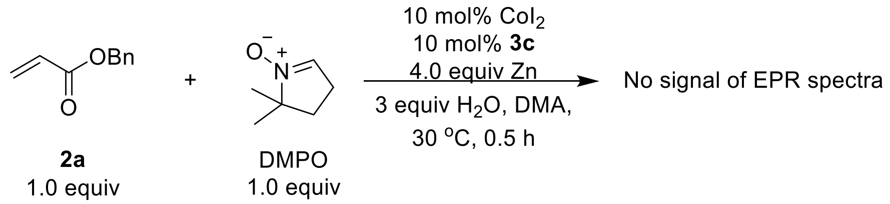

In a N_2-filled glove box, an oven-dried 8-mL vial equipped with a stirring bar was charged with CoI_2 (6.3 mg, 0.02 mmol, 10 mol%), **3c** (11.9 mg, 0.02 mmol, 10 mol%), zinc powder (52.3 mg, 0.8 mmol, 4 eq) and DMA (1.3 mL). The vial was sealed with a cap (phenolic cap with red PTFE/white silicone septum) and the solution was allowed to stir at room temperature for 30 min. **2a** (32.4 mg, 0.2 mmol, 1.0 equiv.), DMPO (22.6 mg, 0.2 mmol, 1.0 equiv.), H_2O (10.8 mg, 0.6 mmol, 3.0 equiv.) and DMA (0.2 mL) were added to the solution. Then the vial was sealed with a cap (phenolic open top cap with red PTFE/white silicone septum), removed from the glove box. The mixture was immediately moved to a thermostatic bath and allowed to stir at 30 ºC for 0.5 h. The reaction mixture was transferred to quartz EPR tube and the spectra was measured at room temperature (**Supplementary Figure 2.**). No signal was observed in the EPR spectra, which indicated that no radical species in the reaction in the absence of **1d**.

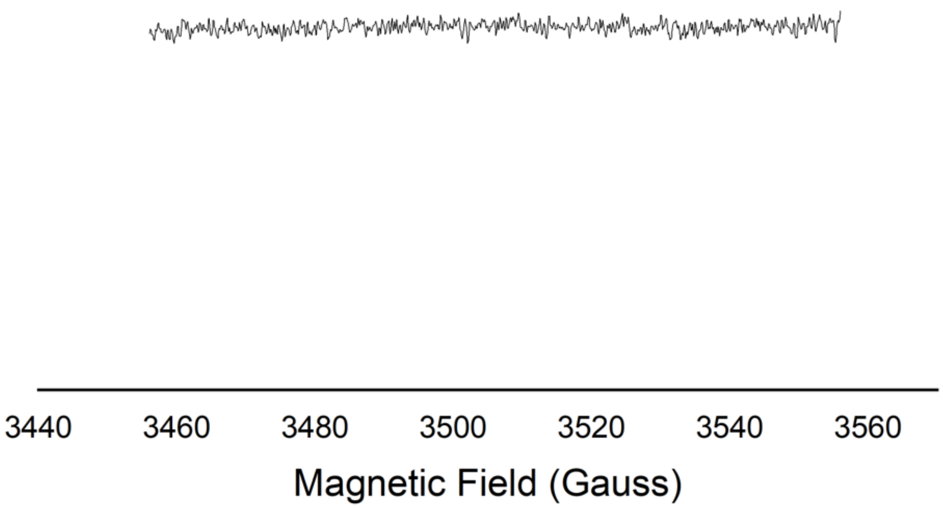

Supplementary Figure 2. EPR spectra in the absence of **1d**.

(b) EPR study of co-catalyzed coupling of 1,3-enyne **1e** and acrylate **2a** to afford 1,3-dienes bearing a tetrasubstituted alkene

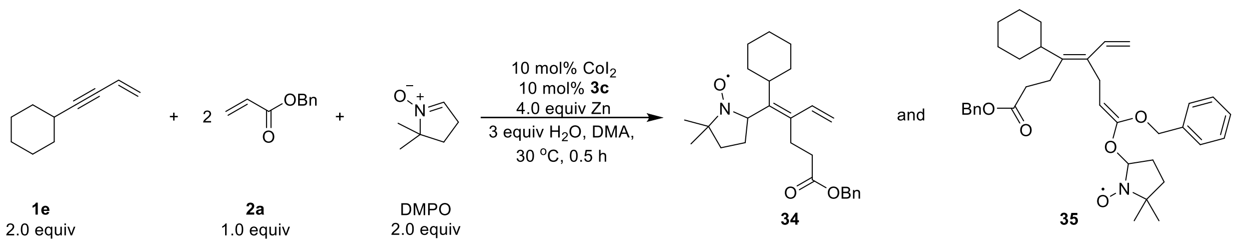

In a N_2-filled glove box, an oven-dried 8-mL vial equipped with a stirring bar was charged with CoI_2 (6.3 mg, 0.02 mmol, 10 mol%), **3c** (11.9 mg, 0.02 mmol, 10 mol%), zinc powder (52.3 mg, 0.8 mmol, 4 eq) and DMA (1.3 mL). The vial was sealed with a cap (phenolic cap with red PTFE/white silicone septum) and the solution was allowed to stir at room temperature for 30 min. **1e** (53.7 mg, 0.4 mmol, 2.0 equiv.), **2a** (64.9 mg, 0.4 mmol, 1.0 equiv.), DMPO (45.3 mg, 0.4 mmol, 2.0 equiv.), H_2O (10.8 mg, 0.6 mmol, 3.0 equiv.) and DMA (0.2 mL) were added to the solution. Then the vial was sealed with a cap (phenolic open top cap with red PTFE/white silicone septum), removed from the glove box. The mixture was immediately moved to a thermostatic bath and allowed to stir at 30 ºC for 0.5 h. The reaction mixture was transferred to quartz EPR tube and the spectra was measured at room temperature (**Supplementary Figure 3.**).

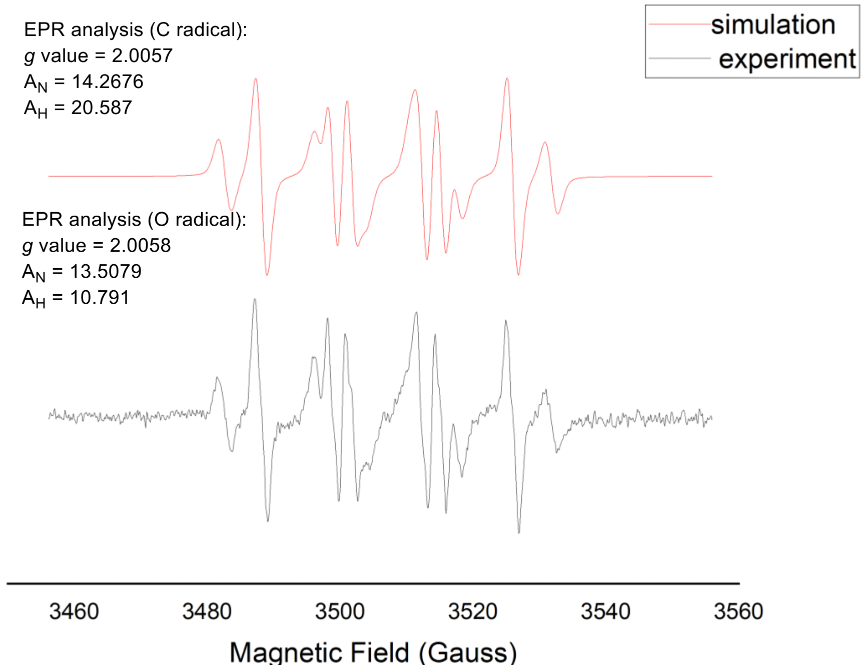

Supplementary Figure 3. EPR spectra for the reaction of **1e**, **2a** and DMPO

1.5.3 SAESI-MS Study

The existence of **34** and **35** was further detected by **SAESI-MS** analysis of reaction mixture of **1e**, **2a** and DMPO.

SAESI-MS conditions[17]

SAESI-MS spectra were recorded on a Thermo TSQ Quantum Access triple-quadrupole mass spectrometer (Thermo Fisher Scientific, Waltham, MA) equipped with a home-made SAESI ion source in positive mode. The basic SAESI conditions were: vacuum, 2.6×10^{-6} torr; spray voltage, 3000 V; capillary temperature, 275°C; sheath gas pressure of two sprayers, 3 arb. units; the collision energy of CID, 10 eV. Data acquisition and analysis were done with the Xcalibur (version 2.0, Thermo Fisher Scientific) software package.

In solvent-assisted electrospray ionization mass spectrometric experiment, the angle (α) between the two sprayers is 50° and the distance (b) between the tip of sprayers and the inlet to the mass is 5 mm. The chemical solutions were injected by a 500-µL air-tight syringe with a speed at 20 µL/min to SAESI-MS. The assisted solvent of methanol was injected by another 500-µL air-tight syringe with a speed at 5 µL/min.

Sample preparation:

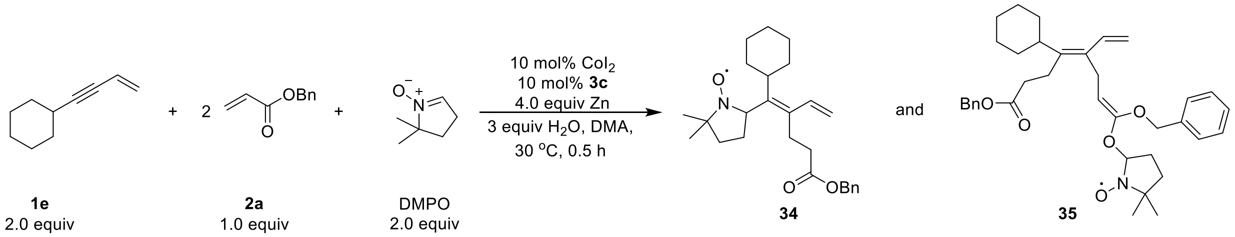

In a N_2-filled glove box, an oven-dried 8-mL vial equipped with a stirring bar was charged with CoI_2 (6.3 mg, 0.02 mmol, 10 mol%), **3c** (11.9 mg, 0.02 mmol, 10 mol%), zinc powder (52.3 mg, 0.8 mmol, 4 eq) and DMA (1.3 mL). The vial was sealed with a cap (phenolic cap with red PTFE/white silicone septum) and the solution was allowed to stir at room temperature for 30 min. **1e** (53.7 mg, 0.4 mmol, 2.0 equiv.), **2a** (64.9 mg, 0.4 mmol, 1.0 equiv.), DMPO (45.3 mg, 0.4 mmol, 2.0 equiv.), H_2O (10.8 mg, 0.6 mmol, 3.0 equiv.) and DMA (0.2 mL) were added to the solution. Then the vial was sealed with a cap (phenolic open top cap with red PTFE/white silicone septum), removed from the glove box. The mixture was immediately moved to a thermostatic bath and allowed to stir at 30 ºC for 0.5 h. Then the reaction mixture was directly used for mass spectrometric experiment.

Mass spectrometric experiment results

Target intermediate:

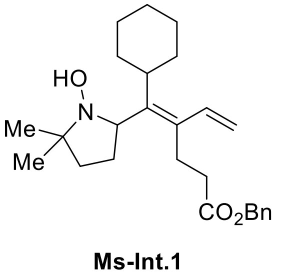

Ms-Int.1

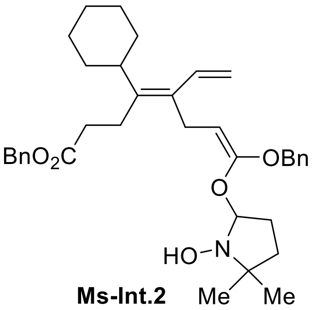

Ms-Int.2

A1) Theoretical isotopic distribution of **Ms-Int.1**

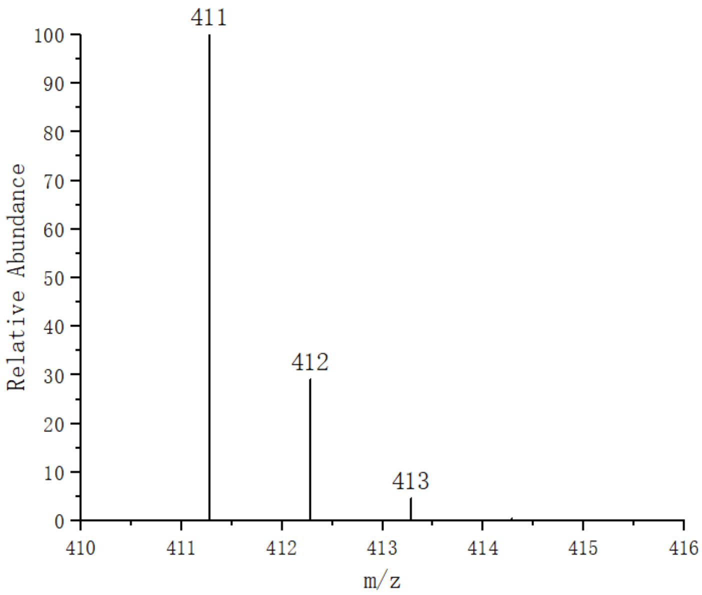

B1) Expanded SAESI-MS spectrum, showing the major signal from m/z 410 to 415.

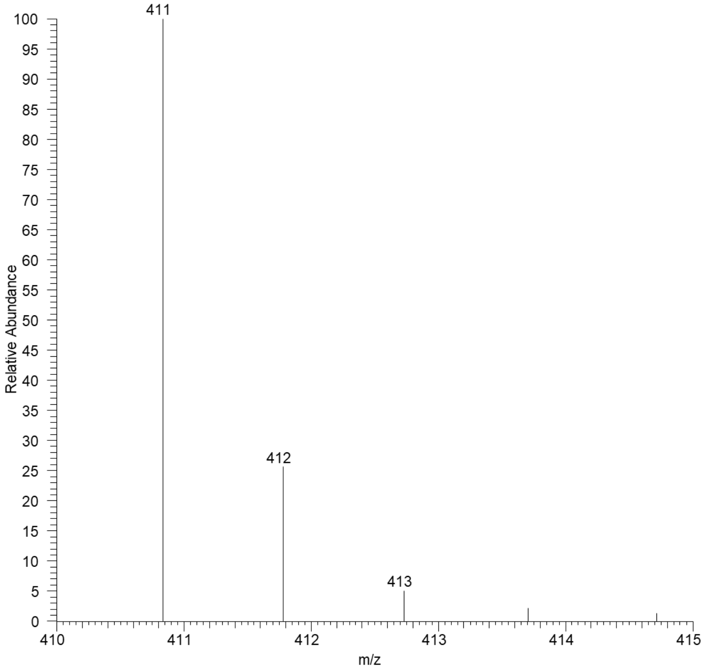

C1) SAESI-MS/MS spectrum of **Ms-Int.1** at m/z 411.

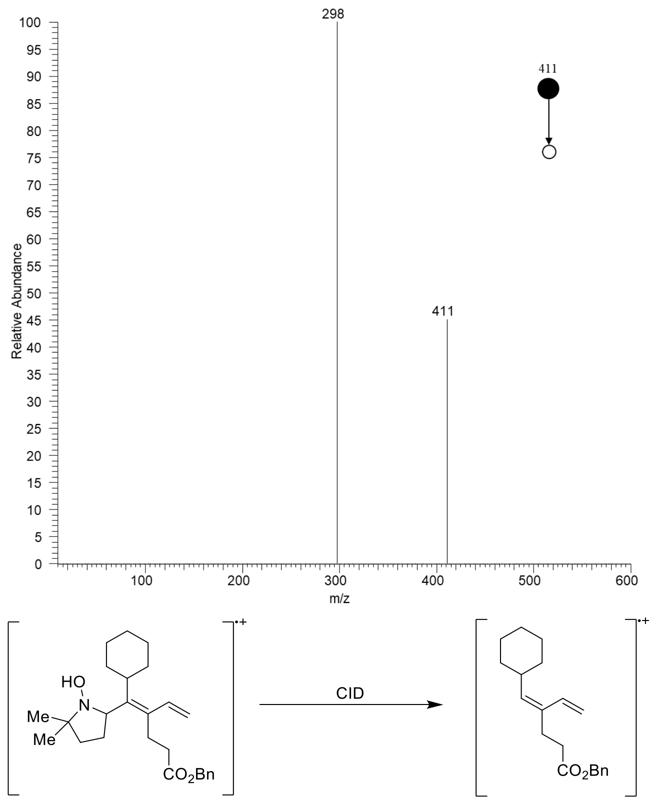

m/z 411 m/z 298

A2) Theoretical isotopic distribution of **Ms-Int.2**

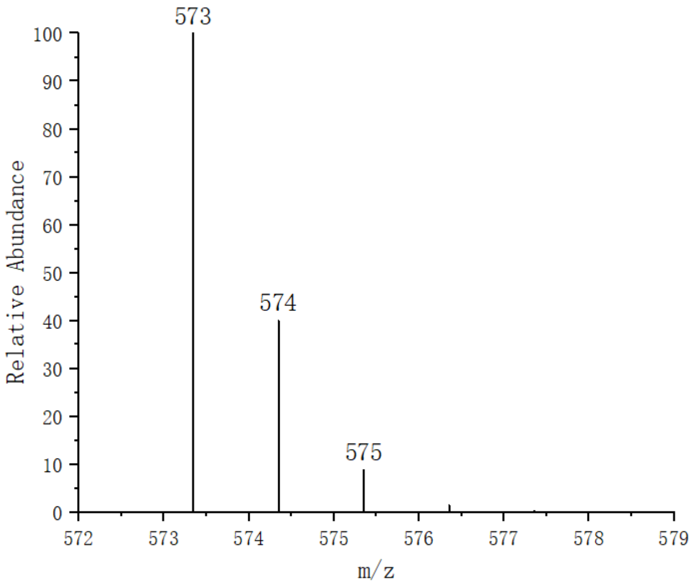

B2）Expanded SAESI-MS spectrum, showing the major signal from m/z 572 to 579.

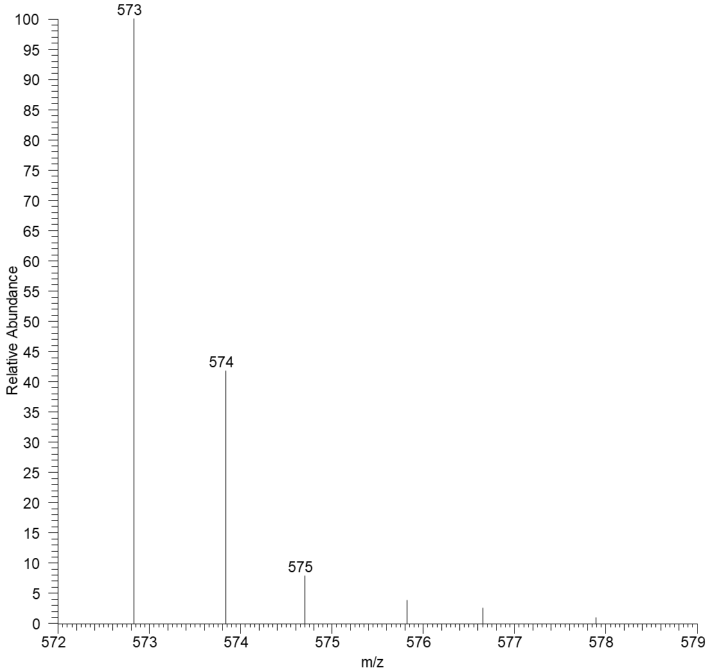

C2）SAESI-MS/MS spectrum of **Ms-Int.2** at m/z 573.

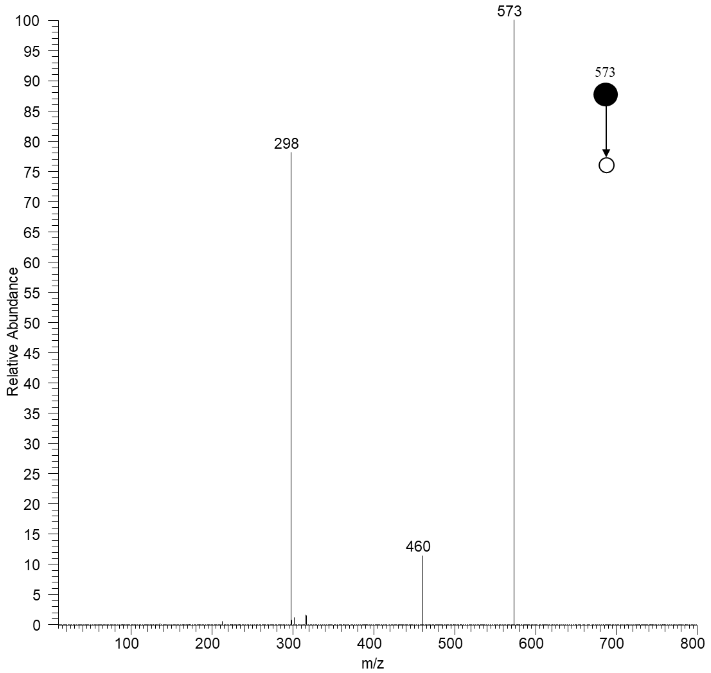

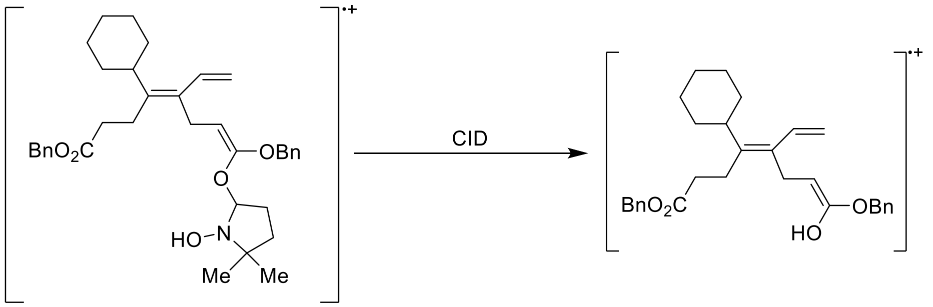

m/z 573 m/z 460

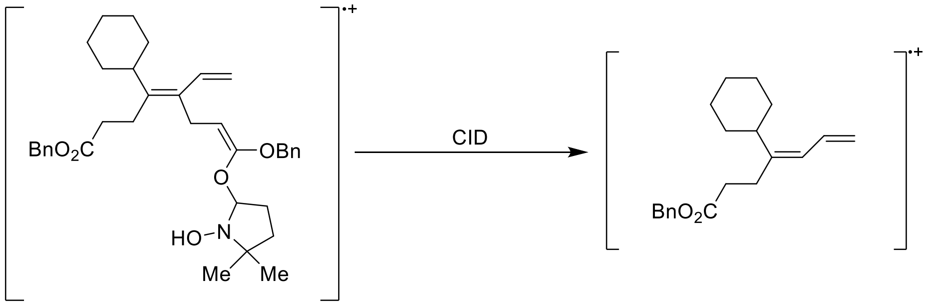

m/z 573 m/z 298

Supplementary Figure 4. SAESI-MS analysis for the reaction of **1e**, **2a** and DMPO

2. Supplementary Notes

2.1 Additional Optimization of Reaction Conditions

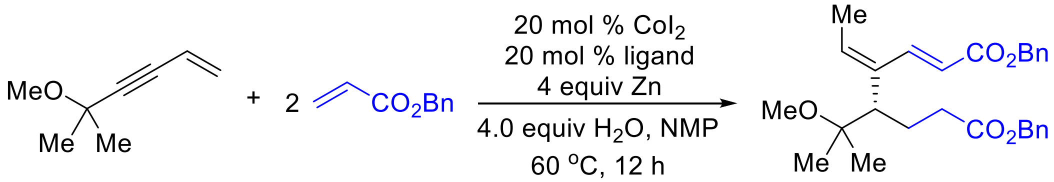

1a **2a** **4a**

entry	ligand	yield (%)[a]	er[b]
1	**3a**	<5	NA[c]
2	**3b**	<5	NA[c]
3	**3c**	<5	NA[c]
4	**3d**	87	93:7
5	**3e**	87	95:5
6	**3f**	85	6:94
7	**3g**	<5	NA[c]
8	**3h**	80	88:12
9	**3i**	74	4:96
10	**3j**	89	5:95

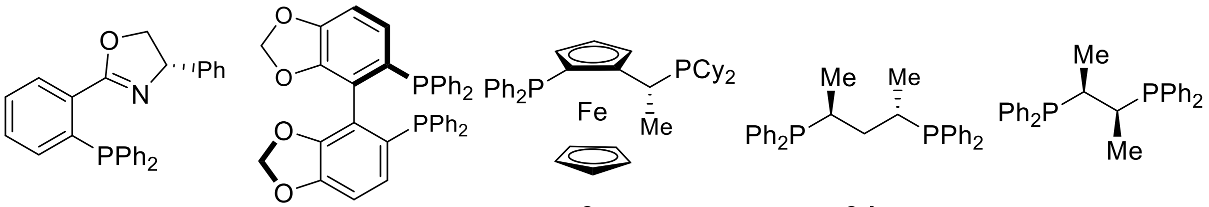

3a **3b** **3c** **3d** **3e**

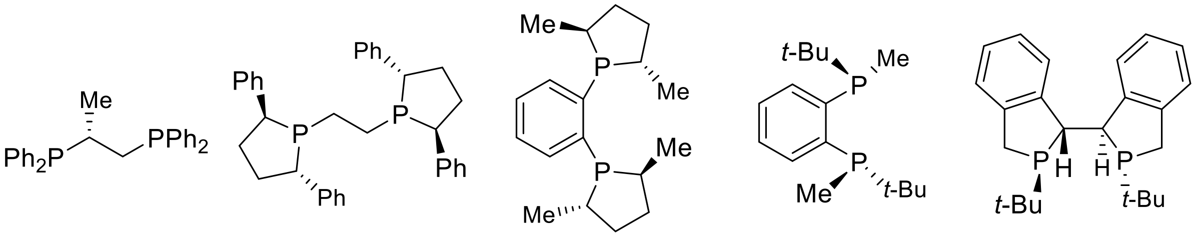

3f **3g** **3h** **3i** **3j**

Supplementary Table 1. Screening of chiral bisphosphine ligands for Co-catalyzed enantioselective coupling of 1,3-enynes and acrylates. [a]Yield of isolated product; [b]Enantiomeric ratios (er) were determined by analysis of HPLC spectra; [c]Not available.

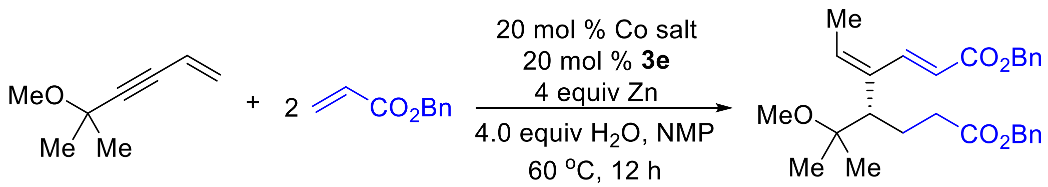

1a **2a** **4a**

entry	Co salt	yield (%)[a]	er[b]
1	$Co(ClO_4)_2•6H_2O$	<5	NA[c]
2	$Co(BF_4)_2•6H_2O$	<5	NA[c]
3	$Co(CF_3SO_3)_2$	<5	NA[c]
4	$Co(OAc)_2$	<5	NA[c]
5	$Co(C_2O_4)$ Co(oxalate)	<5	NA[c]
6	$Co(acac)_2$	<5	NA[c]
7	CoF_2	<5	NA[c]
8	$CoCl_2$	86	95:5
9	$CoBr_2$	68	95:5
10	CoI_2	87	95:5

Supplementary Table 2. Screening of cobalt salts for Co-catalyzed enantioselective coupling of 1,3-enynes and acrylates. [a]Yield of isolated product; [b]Enantiomeric ratios (er) were determined by analysis of HPLC spectra; [c]Not available.

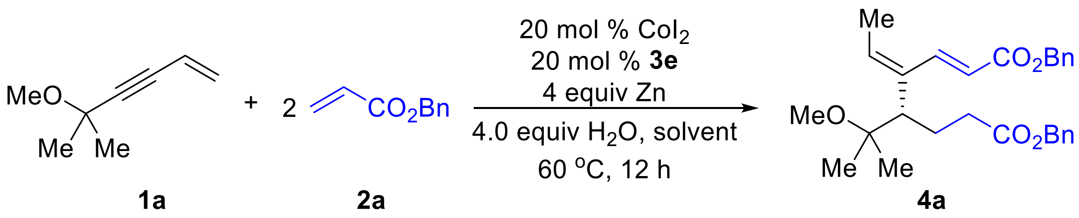

entry	solvent	yield (%)[a]	er[b]
1	toluene	<5	NA[d]
2	cyclohexane	<5	NA[d]
3	DCE	<5	NA[d]
4	CH_3OH	<5	NA[d]
5	THF	14[c]	NA[d]
6	MeCN	12[c]	NA[d]
7	DMSO	54	95:5
8	DMF	74	94:6
9	NMP	87	95:5
10	DMA	90	95:5

Supplementary Table 3. Screening of solvents for Co-catalyzed enantioselective coupling of 1,3-enynes and acrylates. [a]Yield of isolated product; [b]Enantiomeric ratios (er) were determined by analysis of HPLC spectra; [c]NMR yield; [d]Not available.

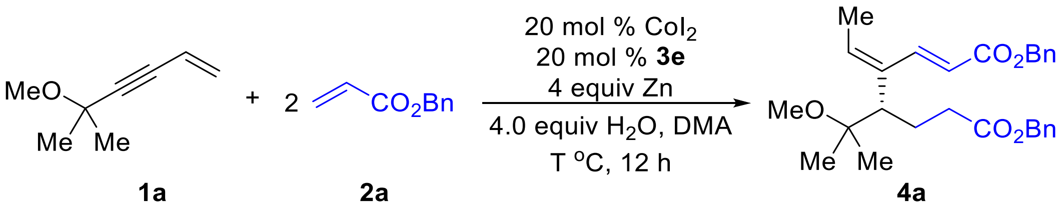

entry	T(°C)	yield (%)[a]	er[b]
1	60	90	95:5
2	50	89	95:5
3	40	86	95:5
4	30	53	95:5
5	20	17	95:5

Supplementary Table 4. Screening of temperatures for Co-catalyzed enantioselective coupling of 1,3-enynes and acrylates. [a]Yield of isolated product; [b]Enantiomeric ratios (er) were determined by analysis of HPLC spectra.

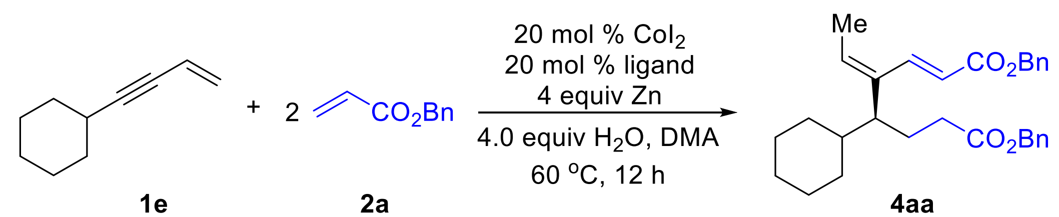

1e **2a** **4aa**

entry	ligand	yield (%)[a]	er[b]
1	**3a**	29	83:17
2	**3b**	<5	NA[c]
3	**3c**	<5	NA[c]
4	**3d**	72	46:54
5	**3e**	40	3:97
6	**3f**	60	96:4
7	**3g**	<5	NA[c]
8	**3h**	22	20.5:79.5
9	**3i**	21	81:19
10	**3j**	<5	NA[c]

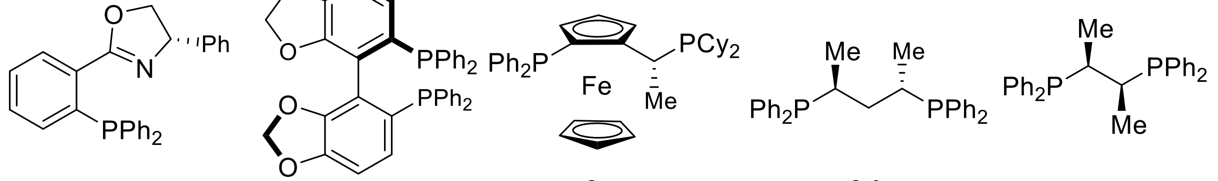

3a **3b** **3c** **3d** **3e**

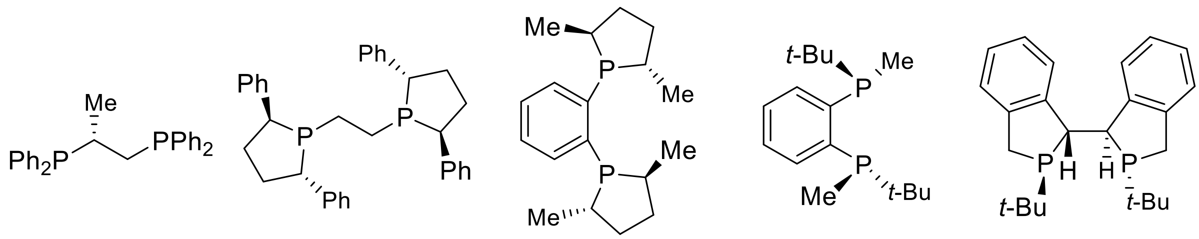

3f **3g** **3h** **3i** **3j**

Supplementary Table 5. Screening of chiral bisphosphine ligands for Co-catalyzed enantioselective coupling of 1,3-enyne **1d** and acrylate **2a**. [a] NMR Yield; [b]Enantiomeric ratios (er) were determined by analysis of HPLC spectra; [c]Not available.

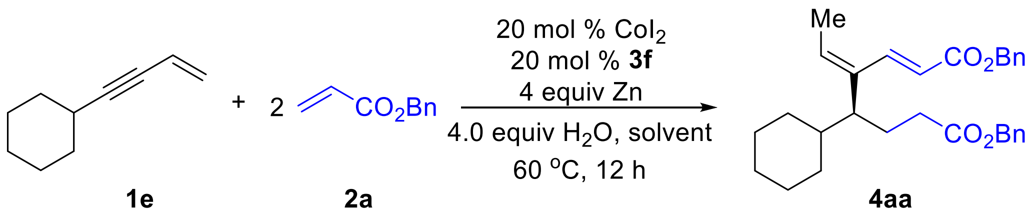

entry	solvent	yield (%)[a]	er[b]
1	toluene	<5	NA[c]
2	cyclohexane	<5	NA[c]
3	DCE	<5	NA[c]
4	CH_3OH	<5	NA[c]
5	MeCN	<5	NA[c]
6	THF	53	92:8
7	DMSO	39	96:4
8	DMF	50	96:4
9	NMP	47	96:4
10	DMA	60	96:4

Supplementary Table 6. Screening of solvents for Co-catalyzed enantioselective coupling of 1,3-enyne **1b** and acrylate **2a**. [a] NMR Yield; [b]Enantiomeric ratios (er) were determined by analysis of HPLC spectra; [c]Not available.

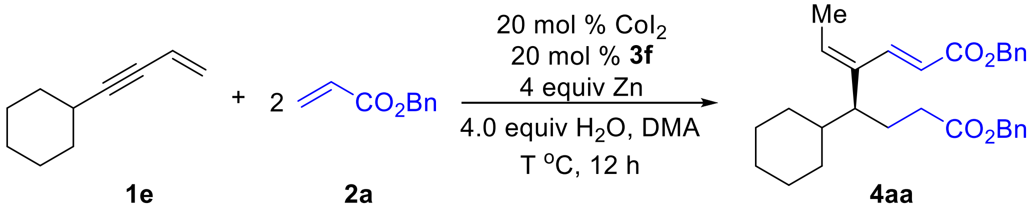

entry	T(°C)	yield (%)[a]	er[b]
1	80	54	95:5
2	70	55	95:5
3	60	59	96:4
4	50	56[c]	97:3
5	40	57	97:3
6	30	63	97:3
7	20	64	97:3

Supplementary Table 7. Screening of temperatures for Co-catalyzed enantioselective coupling of 1,3-enyne **1b** and acrylate **2a**. [a] NMR Yield; [b]Enantiomeric ratios (er) were determined by analysis of HPLC spectra; [c]Yield of isolated product.

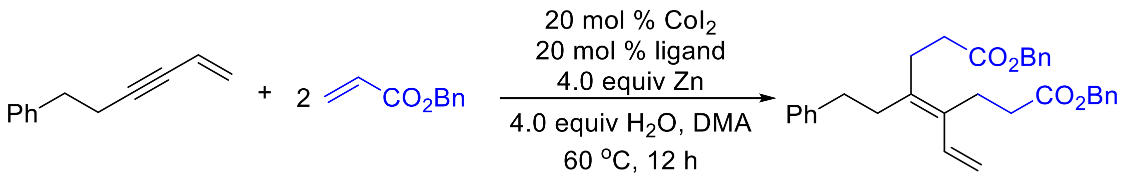

1b **2a** **10a**

entry	ligand	yield (%)[a]	*E/Z*[b]
1	**3a**	27	>98:2
2	**3b**	60	>98:2
3	**3c**	31	>98:2
4	**3d**	10[b]	>98:2
5	**3e**	<5	>98:2
6	**3f**	<5	>98:2
7	**3g**	<5	>98:2
8	**3h**	<5	>98:2
9	**3i**	22	>98:2
10	(*R*,*R*,*S*,*S*)-**3j**	<5	>98:2

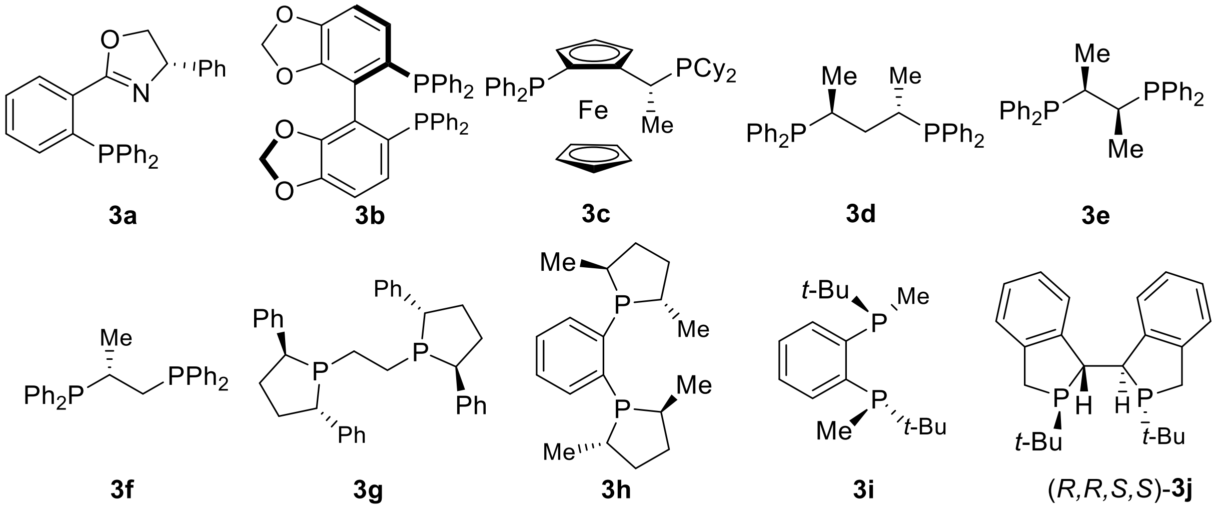

Supplementary Table 8. Screening of chiral bisphosphine ligands for Co-catalyzed coupling of 1,3-enynes and acrylates to afford 1,3-dienes bearing a tetrasubstituted alkene. [a]Yield of isolated product; [b]NMR yield; [c]*E/Z* ratios were determined by analysis of ^{1}H NMR spectra of unpurified mixtures.

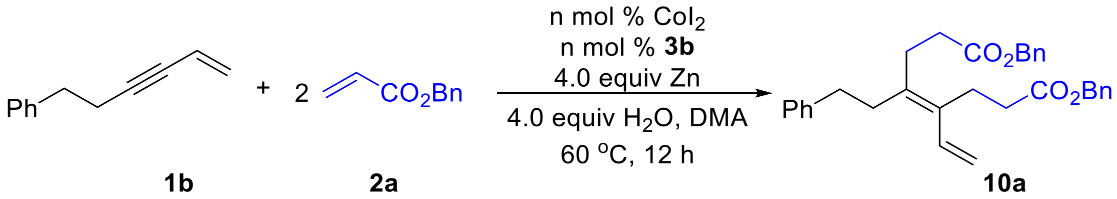

entry	n	yield (%)[a]	*E/Z*[b]
1	20	60	>98:2
2	10	49	>98:2

Supplementary Table 9. Screening of catalyst loadings for Co-catalyzed coupling of 1,3-enynes and acrylates to afford 1,3-dienes bearing a tetrasubstituted alkene. †Yield of isolated product; ‡*E/Z* ratios were determined by analysis of ^{1}H NMR spectra of unpurified mixtures.

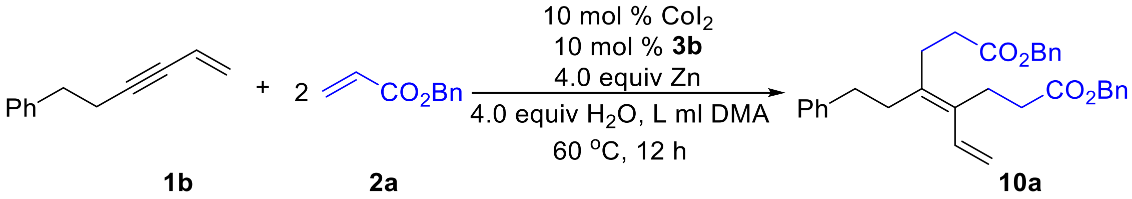

entry	L	yield (%)[a]	*E/Z*[b]
1	1	49	>98:2
2	0.5	63	>98:2

Supplementary Table 10. Screening of concentrations for Co-catalyzed coupling of 1,3-enynes and acrylates to afford 1,3-dienes bearing a tetrasubstituted alkene. [a]Yield of isolated product; [b]*E/Z* ratios were determined by analysis of ^{1}H NMR spectra of unpurified mixtures.

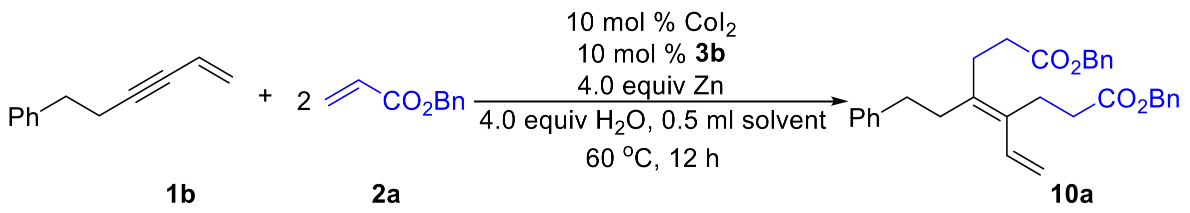

entry	solvent	yield (%)[a]	*E/Z*[b]
1	DCE	9	>98:2
2	MeCN	48	>98:2
3	THF	46	>98:2
4	DMSO	<5	>98:2
5	DMF	64	>98:2
6	NMP	36	>98:2
7	DMA	63	>98:2

Supplementary Table 11. Screening of solvents for Co-catalyzed coupling of 1,3-enynes and acrylates to afford 1,3-dienes bearing a tetrasubstituted alkene. [a]Yield of isolated product; [b]*E/Z* ratios were determined by analysis of ^1H NMR spectra of unpurified mixtures.

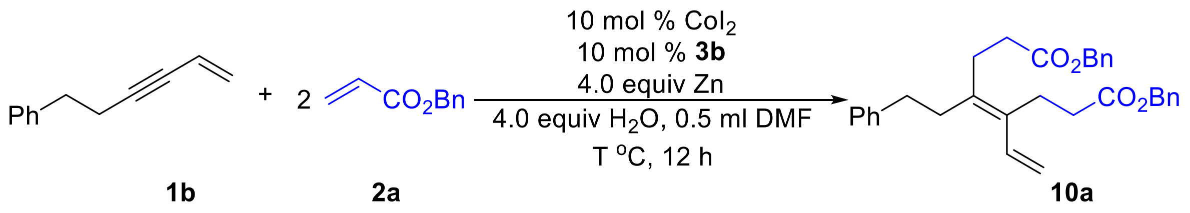

entry	T(°C)	yield (%)[a]	*E/Z*[b]
1	80	26	>98:2
2	70	48	>98:2
3	60	65	>98:2
4	50	70	>98:2
5	40	67	>98:2
6	30	79	>98:2
7	rt(25)	77	>98:2

Supplementary Table 12. Screening of temperatures for Co-catalyzed coupling of 1,3-enynes and acrylates to afford 1,3-dienes bearing a tetrasubstituted alkene. [a]Yield of isolated product; [b]*E/Z* ratios were determined by analysis of ^1H NMR spectra of unpurified mixtures.

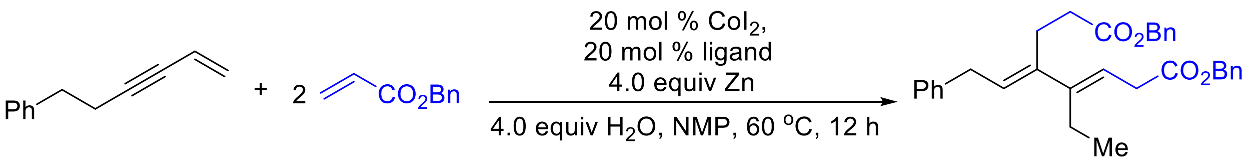

1b **2a** **12a**

entry	ligand	yield (%)[a]
1	**3k**	72
2	**3l**	<5
3	**3m**	<5
4	**3n**	<5
5	**3o**	<5
6	**3p**	<5
7	**3q**	<5
8	**3r**	<5
9	**3s**	<5
10	**3t**	<5

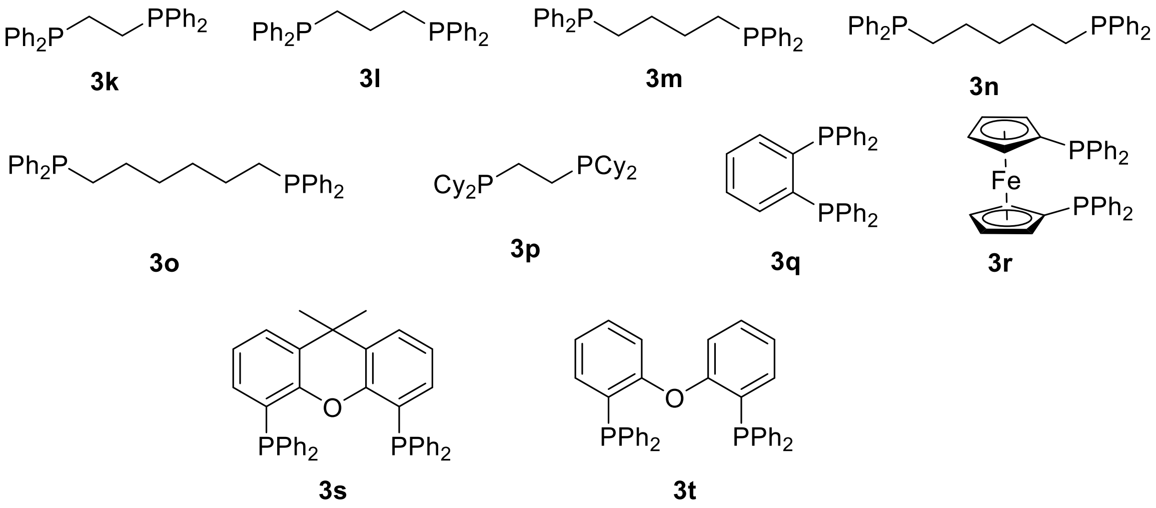

Supplementary Table 13. Screening of bisphosphine ligands for Co-catalyzed coupling of 1,3-enynes and acrylates to form achiral 1,3-dienes containing two trisubstituted olefins. [a]Yield of isolated product.

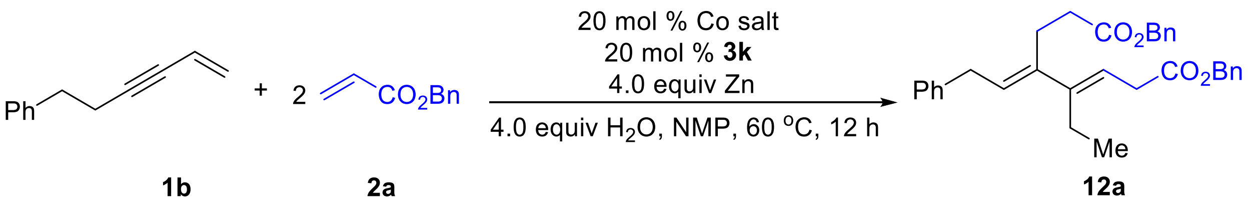

entry	Co salt	yield (%)[a]
1	$Co(ClO_4)_2•6H_2O$	25
2	$Co(BF_4)_2•6H_2O$	15
3	$Co(CF_3SO_3)_2$	27
4	$Co(OAc)_2$	<5
5	$Co(C_2O_4)$ Co(oxalate)	<5
6	$Co(acac)_2$	<5
7	CoF_2	<5
8	$CoCl_2$	72
9	$CoBr_2$	79
10	CoI_2	83

Supplementary Table 14. Screening of cobalt salts for Co-catalyzed coupling of 1,3-enynes and acrylates to form achiral 1,3-dienes containing two trisubstituted olefins. [a]Yield of isolated product.

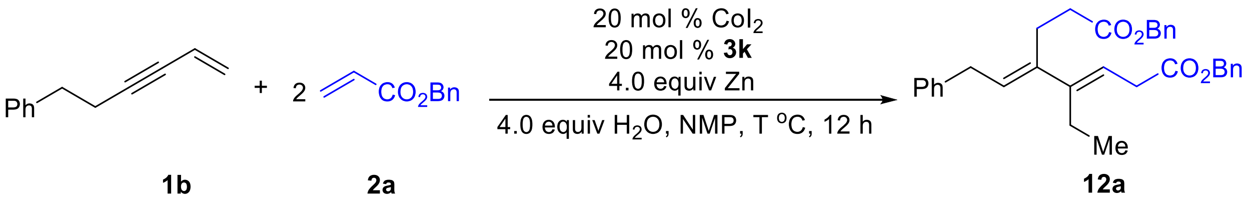

entry	T(°C)	yield (%)[a]
1	80	77
2	70	77
3	60	83
4	50	81
5	40	83
6	30	58
7	rt(25)	47

Supplementary Table 15. Screening of temperatures for Co-catalyzed coupling of 1,3-enynes and acrylates to form achiral 1,3-dienes containing two trisubstituted olefins. [a]Yield of isolated product.

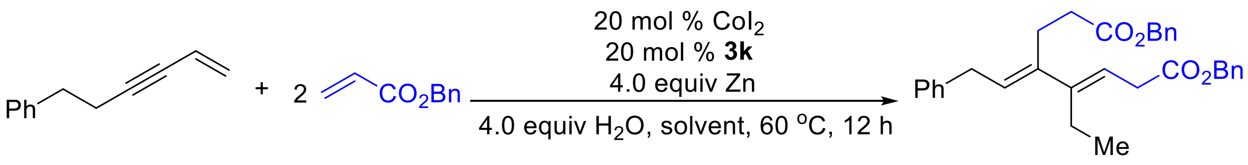

1b **2a** **12a**

entry	solvent	yield (%)[a]
1	toluene	<5
2	cyclohexane	<5
3	DCE	<5
4	CH_3OH	<5
5	THF	<5
6	MeCN	74
7	DMSO	24
8	DMF	79
9	NMP	83
10	DMA	81

Supplementary Table 16. Screening of solvents for Co-catalyzed coupling of 1,3-enynes and acrylates to form achiral 1,3-dienes containing two trisubstituted olefins. [a]Yield of isolated product.

2.2 Characterization of Products

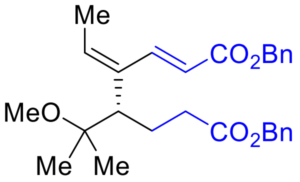

4a

Dibenzyl (*S*,2*E*,4*E*)-4-ethylidene-5-(2-methoxypropan-2-yl)oct-2-enedioate (4a)

Pale yellow oil, 92% yield. **IR (neat):** 3034 (w), 2974 (w), 2825 (w), 1732 (m), 1714 (m), 1620 (w), 1497 (w), 1455 (w), 1379 (w), 1312 (w), 1271 (m), 1244 (w), 1214 (w), 1159 (s), 1074 (w), 1006 (w), 982 (w), 910 (w), 867 (w), 811 (w), 749 (m), 697 (m), 647 (w), 633 (w) cm^{-1}; **^{1}H NMR (400 MHz, $CDCl_3$)** δ 7.84 (d, *J* =16.0 Hz, 1H), 7.41 – 7.32 (m, 10H), 6.01 – 5.97 (m, 2H), 5.20 (s, 2H), 5.08 (d, *J* = 2.8 Hz, 2H), 3.16 (s, 3H), 2.51 (d, *J* = 12.0 Hz, 1H), 2.30 – 2.22 (m, 1H), 2.20 – 2.10 (m, 2H), 1.90 (d, *J* =8.0 Hz, 3H), 1.83 – 1.72 (m, 1H), 1.06 (d, *J* = 2.4 Hz, 6H); **^{13}C NMR (100 MHz, $CDCl_3$)** δ 173.6, 167.4, 143.3, 136.1, 135.9, 135.7, 134.4, 128.52, 128.49, 128.3, 128.19, 128.16, 128.1, 76.9, 66.2, 66.1, 49.0, 32.4, 24.2, 23.6, 21.8, 14.3.; **HRMS (ESI) [M+Na]$^+$** Calcd for $C_{28}H_{34}O_5Na$: 473.22985 m/z, Found: 473.22932 m/z. **Specific rotation**: $[\alpha]_D^{29.6}$ -17.7 (*c* 1.00, $CHCl_3$) for an enantiomerically enriched sample of 94:6 e.r.

Enantiomeric purity of **4a** was determined by HPLC analysis in comparison with authentic racemic material (94:6 e.r. shown; Chiralcel OZ-H column, 95:5 hexane/ *i*-PrOH, 0.7 mL/min, 254 nm).

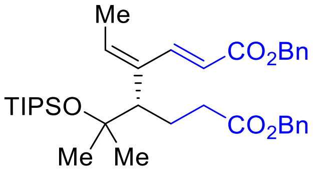

4b

Dibenzyl (*S*,2*E*,4*E*)-4-ethylidene-5-(2-((triisopropylsilyl)oxy)propan-2-yl)oct-2-enedioate (4b)

Colorless oil, 77% yield. **IR (neat):** 3034 (w), 2943 (w), 2866 (w), 2364 (w), 1734 (m), 1716 (m), 1622 (w), 1497 (w), 1458 (w), 1380 (w), 1312 (w), 1270 (m), 1211 (w), 1158 (s), 1032 (m), 982 (w), 882 (w), 805 (w), 747 (w), 696 (m), 674 (m), 646 (m) cm^{-1}; **^{1}H NMR (400 MHz, $CDCl_3$)** δ 7.86 (d, *J* = 15.6 Hz, 1H), 7.43 – 7.29 (m, 10H), 6.04 (d, *J* = 7.6 Hz, 1H), 5.93 (d, *J* = 15.6 Hz, 1H), 5.20 (s, 2H), 5.14 – 4.94 (m, 2H), 2.38 (d, *J* = 12.0 Hz, 1H), 2.29 – 2.21 (m, 2H), 2.14 (t, *J* = 9.6 Hz, 1H), 1.89 (d, *J* = 7.2 Hz, 3H), 1.87 – 1.78 (m, 1H), 1.24 (s, 3H), 1.15 (s, 3H), 1.04 (s, 21H);

^{13}C NMR (100 MHz, CDCl$_3$) δ 173.6, 167.4, 143.6, 136.2, 136.0, 135.9, 134.3, 128.54, 128.50, 128.25, 128.15, 128.13, 128.11, 117.2, 75.5, 66.2, 66.1, 32.6, 29.8, 27.9, 25.1, 18.4, 14.2, 13.5; **HRMS (ESI) [M+Na]$^+$** Calcd for $C_{36}H_{52}O_5NaSi$: 615.34762 m/z, Found: 615.34831 m/z; **Specific rotation**: $[\alpha]_D^{29.5}$ -17.4 (*c* 1.00, $CHCl_3$) for an enantiomerically enriched sample of 92:8 e.r.

Enantiomeric purity of **4b** was determined by HPLC analysis in comparison with authentic racemic material (92:8 e.r. shown; Chiralcel OZ-H column, 95:5 hexane/ *i*-PrOH, 0.5 mL/min, 290 nm).

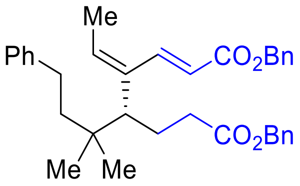

4c

Dibenzyl (R,2E,4E)-4-ethylidene-5-(2-methyl-4-phenylbutan-2-yl)oct-2-enedioate (4c)

Colorless oil, 52% yield. **IR (neat):** 2959 (w), 1715 (m), 1620 (w), 1454 (w), 1270 (m), 1160 (s), 981 (w), 868 (w), 747 (m), 697 (s) cm^{-1}; **^{1}H NMR (400 MHz, CDCl$_3$)** δ 7.90 (s, 1H), 7.44 – 7.27 (m, 10H), 7.26 – 7.21 (m, 2H), 7.18 – 7.09 (m, 3H), 5.96 (d, *J* = 16.0 Hz, 1H), 5.87 (s, 1H), 5.19 (s, 2H), 5.07 (s, 2H), 2.56 – 2.51 (m, 2H), 2.42 – 2.20 (m, 2H), 2.18 – 2.09 (m, 1H), 2.05 – 1.99 (m, 1H), 1.90 (d, *J* = 8.0 Hz, 3H), 1.75 – 1.64 (m, 1H), 1.64 – 1.47 (m, 2H), 0.89 (s, 3H), 0.87 (s, 3H); **^{13}C NMR (100 MHz, CDCl$_3$)** δ 173.6, 167.5, 144.0, 143.2, 136.2, 136.1, 136.0, 133.5, 128.62, 128.59, 128.39, 128.37, 128.26, 128.21, 128.2, 125.7, 116.9, 66.3, 66.2, 46.7, 43.0, 36.9, 32.7, 30.6, 25.5, 24.3, 14.2; **HRMS (ESI) [M+Na]$^+$** Calcd for $C_{35}H_{40}O_4Na$: 547.28188 m/z, Found: 547.28114 m/z; **Specific rotation**: $[\alpha]_D^{28.5}$ -29.1 (*c* 1.00, $CHCl_3$) for an enantiomerically enriched sample of 94:6 e.r.

Enantiomeric purity of **4c** was determined by HPLC analysis in comparison with authentic racemic material (94:6 e.r. shown; Chiralpak IG column, 95:5 hexane/ *i*-PrOH, 1.0 mL/min, 254 nm).

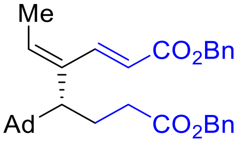

4d

Dibenzyl (*R*,2*E*,4*E*)-5-((3*R*,5*R*,7*R*)-adamantan-1-yl)-4-ethylideneoct-2-enedioate (4d)

Colorless oil, 60% yield. **IR (neat):** 3032 (w), 2902 (w), 2847 (w), 1732 (m), 1713 (m), 1619 (w), 1497 (w), 1452 (w), 1377 (w), 1328 (w), 1272 (m), 1214 (w), 1158 (s), 1103 (w), 1081 (w), 1008 (w), 980 (w), 909 (w), 866 (w), 809 (w), 749 (m), 697 (m), 655 (w), 640 (w), 611 (w) cm^{-1}; **^{1}H NMR (400 MHz, $CDCl_3$)** δ 7.92 (d, *J* = 16.0 Hz, 1H), 7.42 – 7.39 (m, 2H), 7.38 – 7.37 (m, 1H), 7.36 – 7.30 (m, 7H), 5.94 (d, *J* = 16.0 Hz, 1H), 5.82 (s, 1H), 5.20 (d, *J* = 3.2 Hz, 2H), 5.16 – 4.97 (m, 2H), 2.33 – 2.19 (m, 1H), 2.19 – 2.01 (m, 3H), 1.94 – 1.90 (m, 6H), 1.69 – 1.63 (m, 5H), 1.58 – 1.56 (m, 5H), 1.36 (d, *J* = 12.0 Hz, 3H); **^{13}C NMR (100 MHz, $CDCl_3$)** δ 173.7, 167.6, 144.0, 136.1, 136.0, 135.4, 133.2, 128.54, 128.50, 128.3, 128.22, 128.18, 128.1, 116.7, 66.2, 66.1, 49.3, 39.9, 37.0, 35.9, 32.7, 28.6, 14.0; **HRMS (ESI) [M+Na]$^+$** Calcd for $C_{34}H_{40}O_4Na$: 535.28188 m/z, Found: 535.28120 m/z; **Specific rotation**: $[\alpha]_D^{28.7}$ -19.7 (*c* 1.00, $CHCl_3$) for an enantiomerically enriched sample of 94:6 e.r.

Enantiomeric purity of **4d** was determined by HPLC analysis in comparison with authentic racemic material (94:6 e.r. shown; Chiralcel OZ-H column, 95:5 hexane/ *i*-PrOH, 0.7 mL/min, 254 nm).

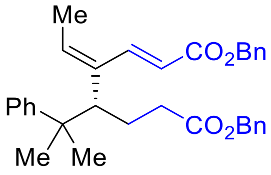

4e

Dibenzyl (*S*,2*E*,4*E*)-4-ethylidene-5-(2-phenylpropan-2-yl)oct-2-enedioate (4e)

Pale yellow oil, 92% yield. **IR (neat):** 3032 (w), 2966 (w), 1731 (m), 1715 (w), 1621 (w), 1496 (w), 1454 (w), 1378 (w), 1271 (m), 1213 (w), 1160 (s), 1083 (w), 1027 (w), 980 (w), 908 (w), 866 (w), 803 (w), 749 (m), 698 (s), 629 (w) cm^{-1}; **^{1}H NMR (400 MHz, $CDCl_3$)** δ 7.81 (d, *J* = 15.6 Hz, 1H), 7.45 – 7.40 (m, 4H), 7.40 – 7.36 (m, 1H), 7.35 – 7.27 (m, 9H), 7.20 – 7.13 (m, 1H), 5.96 (d, *J* = 15.6 Hz, 1H), 5.58 (q, *J* = 7.2 Hz, 1H), 5.22 (s, 2H), 5.02 (s, 2H), 2.66 (d, *J* = 9.2 Hz, 1H), 2.19 – 2.12 (m, 1H), 2.05 – 1.97 (m, 1H), 1.88 (d, *J* = 7.2 Hz, 3H), 1.81 – 1.73 (m, 1H), 1.69 – 1.57 (m, 1H), 1.30 (s, 3H), 1.23 (s, 3H); **^{13}C NMR (100 MHz, $CDCl_3$)** δ 173.3, 167.3, 147.9, 143.6, 136.1, 135.9, 135.2, 134.0, 128.52, 128.46, 128.3, 128.2, 128.13, 128.10, 127.8, 126.6, 125.8, 117.0, 66.2, 66.0, 41.5, 32.5, 28.4, 25.0, 24.2, 14.1; **HRMS (ESI) [M+Na]$^+$** Calcd for $C_{33}H_{36}O_4Na$: 519.25058 m/z, Found: 519.25107 m/z; **Specific rotation**: $[\alpha]_D^{29.6}$ -78.0 (*c* 1.00, $CHCl_3$) for an enantiomerically enriched sample of 95:5 e.r.

Enantiomeric purity of **4e** was determined by HPLC analysis in comparison with authentic racemic material (95:5 e.r. shown; Chiralcel OZ-H column, 95:5 hexane/ *i*-PrOH, 0.7 mL/min, 254 nm).

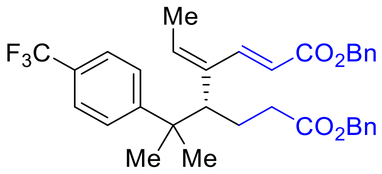

4f

Dibenzyl (*S*,2*E*,4*E*)-4-ethylidene-5-(2-(4-(trifluoromethyl)phenyl)propan-2-yl)oct-2-enedioate (4f)

Pale yellow oil, 75% yield. **IR (neat):** 3034 (w), 2971 (w), 1715 (m), 1618 (w), 1497 (w), 1454 (w), 1409 (w), 1377 (w), 1328 (s), 1271 (m), 1214 (w), 1162 (s), 1119 (s), 1067 (w), 1014 (w), 980 (w), 909 (w), 867 (w), 840 (w), 748 (m), 697 (m), 641 (w), 614 (w) cm^{-1}; **^{1}H NMR (400 MHz, $CDCl_3$)** δ 7.74 (d, *J* = 15.6 Hz, 1H), 7.51 (d, *J* = 8.0 Hz, 2H), 7.39 –7.34 (m, 7H), 7.32 – 7.24 (m, 5H), 5.89 (d, *J* = 15.6 Hz, 1H), 5.59 (q, *J* = 7.2 Hz, 1H), 5.19 (s, 2H), 5.01 (s, 2H), 2.66 (d, *J* = 12.4 Hz, 1H), 2.21 – 2.13 (m, 1H), 2.06 – 1.98 (m, 1H), 1.86 (d, *J* = 7.2 Hz, 3H), 1.81 – 1.69 (m, 1H), 1.65 – 1.57 (m, 1H), 1.31 (s, 3H), 1.22 (s, 3H); **^{13}C NMR (100 MHz, $CDCl_3$)** δ 173.2, 167.2, 152.3, 143.3, 136.1, 135.9, 134.8, 134.3, 128.63, 128.58, 128.4, 128.30, 128.28, 128.27, 128.2 (q, *J* = 32.4 Hz), 127.1, 124.7(q, *J* = 4.0 Hz), 124.4(q, *J* = 270.0 Hz), 117.3, 66.4, 66.2, 41.8, 32.3, 28.0, 24.9, 24.4, 14.2; **^{19}F NMR (376 MHz, $CDCl_3$)** δ -62.24; **HRMS (ESI) $[M+Na]^+$** Calcd for $C_{34}H_{35}O_4F_3Na$: 587.23797 m/z, Found: 587.23734 m/z; **Specific rotation**: $[\alpha]_D^{28.3}$ -64.8 (*c* 1.00, $CHCl_3$) for an enantiomerically enriched sample of 96:4 e.r.

Enantiomeric purity of **4f** was determined by HPLC analysis in comparison with authentic racemic material (96:4 e.r. shown; Chiralcel OZ-H column, 97:3 hexane/ *i*-PrOH, 0.5 mL/min, 254 nm).

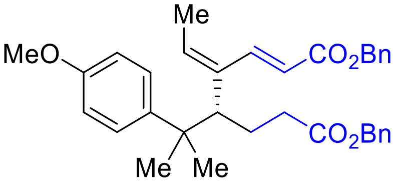

4g

Dibenzyl (*S*,2*E*,4*E*)-4-ethylidene-5-(2-(4-methoxyphenyl)propan-2-yl)oct-2-enedioate (4g)

Colorless oil, 71% yield. **IR (neat):** 3035 (w), 2964 (w), 2836 (w), 1731 (m), 1713 (m), 1611 (w), 1512 (m), 1455 (w), 1379 (w), 1271 (m), 1250 (m), 1159 (s), 1115 (w), 1084 (w), 1034 (w), 1009 (w), 980 (w), 908 (w), 867 (w), 830 (w), 805 (w), 739 (m), 697 (m), 641 (w), 621 (w) cm^{-1}; **^{1}H NMR (400 MHz, $CDCl_3$)** δ 7.81 (d, *J* = 16.0 Hz, 1H), 7.45 – 7.39 (m, 4H), 7.38 – 7.36 (m, 1H), 7.36 – 7.31 (m, 3H), 7.31 – 7.27 (m, 2H), 7.24 – 7.18 (m, 2H), 6.90 – 6.69 (m, 2H), 5.95 (d, *J* = 16.0 Hz, 1H), 5.54 (q, *J* = 8.0 Hz, 1H), 5.22 (s, 2H), 5.03 (s, 2H), 3.78 (s, 3H), 2.61 (dd, *J* = 12.0, 3.2 Hz, 1H), 2.21 – 2.13 (m,1H), 2.09 – 1.96 (m, 1H), 1.87 (d, *J* = 7.2 Hz, 3H), 1.85 – 1.75 (m, 1H), 1.65 – 1.54 (m,1H), 1.26 (s, 3H), 1.22 (s, 3H); **^{13}C NMR (100 MHz, $CDCl_3$)** δ 173.4, 167.4, 157.5, 143.6, 139.8, 136.1, 135.9, 135.3, 134.0, 128.53, 128.47, 128.3, 128.2, 128.14, 128.11, 127.7, 116.9, 113.0, 66.2, 66.0, 55.1, 40.9, 32.5, 28.6, 25.1, 24.6, 14.1. **HRMS (ESI) $[M+Na]^+$** Calcd for $C_{34}H_{38}O_5Na$: 549.26115 m/z, Found: 549.26083 m/z; **Specific rotation**: $[\alpha]_D^{29.5}$ -65.0 (*c* 1.00, $CHCl_3$) for an enantiomerically enriched sample of 94:6 e.r.

Enantiomeric purity of **4g** was determined by HPLC analysis in comparison with authentic racemic material (94:6 e.r. shown; Chiralcel OZ-H column, 95:5 hexane/ *i*-PrOH, 0.5 mL/min, 290 nm).

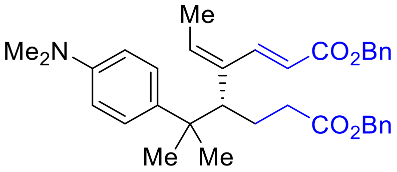

4h

Dibenzyl (*S*,2*E*,4*E*)-5-(2-(4-(dimethylamino)phenyl)propan-2-yl)-4-ethylideneoct-2-enedioate (4h)

Colorless oil, 82% yield. **IR (neat):** 3979 (w), 3922 (w), 3845 (w), 3753 (w), 3718 (w), 3676 (w), 3519 (w), 3034 (w), 2962 (w), 2798 (s), 1732 (m), 1713 (w), 1613 (m), 1521 (m), 1497 (w), 1454 (w), 1349 (w), 1269 (m), 1211 (w), 1159 (s), 1086 (w), 1016 (w), 980 (w), 949 (w), 907 (w), 866 (w), 817 (w),800 (m), 738 (w), 697 (m), 668 (m), 639 (w), 626 (w) cm^{-1}; **^{1}H NMR (400 MHz, $CDCl_3$)** δ 7.85 (d, *J* = 15.6 Hz, 1H), 7.45 – 7.39 (m, 4H), 7.39 – 7.34 (m, 2H), 7.34 – 7.27 (m, 4H), 7.21 – 7.11 (m, 2H), 6.74 – 6.61 (m, 2H), 5.99 (d, *J* =16.0 Hz, 1H), 5.55 (q, *J* =8.0 Hz, 1H), 5.22 (d, *J* = 2.0 Hz, 2H), 5.03 (s, 2H), 2.92 (s, 6H), 2.62 (dd, *J* = 12.4, 2.0 Hz, 1H), 2.20 – 2.13 (m, 1H), 2.07 – 1.99 (m, 1H), 1.88 (d, *J* = 7.2 Hz, 3H), 1.86 – 1.80 (m, 1H), 1.65 – 1.55 (m, 1H), 1.25 (s, 3H), 1.22 (s, 3H); **^{13}C NMR (100 MHz, $CDCl_3$)** δ 173.5, 167.5, 148.7, 143.8, 136.1, 135.9, 135.6, 135.4, 134.0, 128.52, 128.45, 128.3, 128.14, 128.12, 128.07, 127.3, 116.7, 112.1, 66.2, 66.0, 40.6,

32.6, 28.9, 25.2, 24.4, 14.0; **HRMS (ESI) [M+H]$^+$** Calcd for $C_{35}H_{42}NO_4$: 540.31084 m/z, Found: 540.31060 m/z; **Specific rotation**: $[\alpha]_D^{28.5}$ -71.7 (*c* 1.00, $CHCl_3$) for an enantiomerically enriched sample of 94:6 e.r.

Enantiomeric purity of **4h** was determined by HPLC analysis in comparison with authentic racemic material (94:6 e.r. shown; Chiralpak IA column, 95:5 hexane/ *i*-PrOH, 0.7 mL/min, 290 nm).

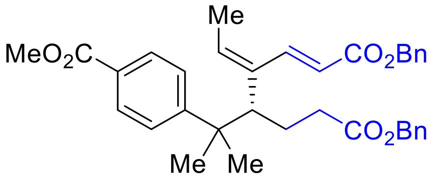

4i

Dibenzyl (S,2E,4E)-4-ethylidene-5-(2-(4-(methoxycarbonyl)phenyl)propan-2-yl)oct-2-enedioate (4i)

Colorless oil, 86% yield. **IR (neat):** 3036 (w), 2954 (w), 1719 (s), 1610 (w), 1498 (w), 1454 (w), 1437 (w), 1408 (w), 1378 (w), 1316 (w), 1276 (s), 1215 (w), 1161 (s), 1114 (m), 1016 (w), 979 (w), 910 (w), 863 (w), 827 (w), 777 (w), 747 (m), 700 (m), 653 (w) cm^{-1}; **^{1}H NMR (400 MHz, $CDCl_3$)** δ 8.00 – 7.88 (d, *J* = 8.0 Hz, 2H), 7.77 (d, *J* = 15.6 Hz, 1H), 7.43 – 7.38 (m, 4H), 7.36 (d, *J* = 1.6 Hz, 1H), 7.36 – 7.33 (m, 2H), 7.31 (d, *J* = 1.6 Hz, 1H), 7.31 – 7.26 (m, 3H), 7.26 – 7.23 (m, 1H), 5.94 (d, *J* = 15.6 Hz, 1H), 5.56 (q, *J* = 7.2 Hz, 1H), 5.19 (d, *J* = 1.6 Hz, 2H), 5.00 (s, 2H), 3.89 (s, 3H), 2.67 (d, *J* = 12.4, 1H), 2.24 – 2.06 (m, 1H), 2.05 – 2.01 (m, 1H), 1.85 (d, *J* = 7.2 Hz, 3H), 1.77 – 1.71 (m, 1H), 1.69 – 1.58 (m, 1H), 1.29 (s, 3H), 1.22 (s, 3H); **^{13}C NMR (100 MHz, $CDCl_3$)** δ 173.1, 167.2, 166.9, 153.4, 143.4, 136.0, 135.8, 134.8, 134.2, 129.0, 128.5, 128.4, 128.3, 128.2, 128.13, 128.11, 127.7, 126.7,117.1, 66.3, 66.1, 51.9, 41.9, 32.3, 28.2, 24.9, 24.1, 14.1; **HRMS (ESI) [M+Na]$^+$** Calcd for $C_{35}H_{38}O_6Na$: 577.25606 m/z, Found: 577.25513 m/z; **Specific rotation**: $[\alpha]_D^{24.6}$ -67.0 (*c* 1.00, $CHCl_3$) for an enantiomerically enriched sample of 96:4 e.r.

Enantiomeric purity of **4i** was determined by HPLC analysis in comparison with authentic racemic material (96:4 e.r. shown; Chiralpak IA column, 95:5 hexane/ *i*-PrOH, 0.7 mL/min, 208 nm).

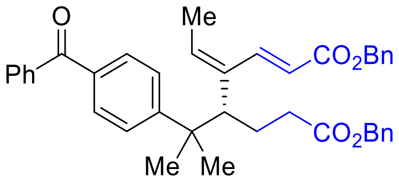

4j

Dibenzyl (*S*,2*E*,4*E*)-5-(2-(4-benzoylphenyl)propan-2-yl)-4-ethylideneoct-2-enedioate (4j)

Colorless oil, 52% yield. **IR (neat):** 3034 (w), 2969 (w), 1731 (m), 1714 (m), 1657 (m), 1604 (m), 1497 (w), 1451 (w), 1405 (w), 1378 (w), 1316 (w), 1274 (s), 1214 (w), 1161 (s), 1096 (w), 1002 (w), 980 (w), 939 (w), 924 (w), 866 (w), 850 (w), 796 (w), 750 (m), 699 (s), 647 (w) cm^{-1}; **^{1}H NMR (400 MHz, $CDCl_3$)** δ 7.78 (d, *J* = 7.6 Hz, 3H), 7.73 (d, *J* = 8.0 Hz, 2H), 7.60 – 7.56 (m, 1H), 7.49 – 7.45 (m, 2H), 7.44 – 7.35 (m, 6H), 7.35 – 7.33 (m, 1H), 7.32 – 7.26 (m, 5H), 5.93 (d, *J* = 15.8 Hz, 1H), 5.63 (q, *J* = 7.2 Hz, 1H), 5.19 (s, 2H), 5.02 (s, 2H), 2.76 – 2.65 (m, 1H), 2.22 – 2.14 (m, 1H), 2.09 – 1.99 (m, 1H), 1.88 (d, *J* = 7.2 Hz, 3H), 1.82 – 1.74 (m, 1H), 1.71 – 1.59 (m, 1H), 1.33 (s, 3H), 1.25 (s, 3H);; **^{13}C NMR (100 MHz, $CDCl_3$)** δ 196.3, 173.2, 167.2, 153.2, 143.4, 137.7, 136.0, 135.8, 135.1, 134.9, 134.2, 132.2, 129.9, 129.8, 128.54, 128.50, 128.3, 128.21, 128.18, 126.6, 117.2, 66.3, 66.1, 42.0, 32.3, 28.1, 24.9, 24.2, 14.1; **HRMS (ESI) $[M+Na]^+$** Calcd for $C_{40}H_{40}O_5Na$: 623.27680 m/z, Found: 623.27625 m/z; **Specific rotation**: $[\alpha]_D^{28.6}$ -48.9 (*c* 1.00, $CHCl_3$) for an enantiomerically enriched sample of 94:6 e.r.

Enantiomeric purity of **4j** was determined by HPLC analysis in comparison with authentic racemic material (94:6 e.r. shown; Chiralpak IB column, 92:8 hexane/ *i*-PrOH, 1.0 mL/min, 290 nm).

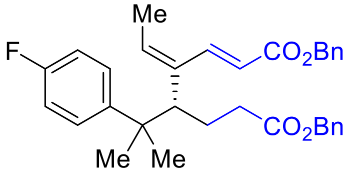

4k

Dibenzyl (*S*,2*E*,4*E*)-4-ethylidene-5-(2-(4-fluorophenyl)propan-2-yl)oct-2-enedioate (4k)

Pale yellow oil, 47% yield. **IR (neat):** 3035 (w), 2965 (w), 1714 (m), 1619 (w), 1509 (w), 1454 (w), 1379 (w), 1312 (w), 1271 (m), 1230 (m), 1161 (s), 1100 (w), 1010 (w), 980 (w), 909 (w), 867 (w), 835 (m), 745 (m), 698 (m), 622 (w) cm^{-1}; **^{1}H NMR (400 MHz, $CDCl_3$)** δ 7.76 (d, *J* = 15.6 Hz, 1H), 7.44 – 7.38 (m, 4H), 7.38 – 7.33 (m, 2H), 7.33 – 7.30 (m, 2H), 7.29 – 7.26 (m,

2H), 7.25 – 7.20 (m, 2H), 6.99 – 6.87 (m, 2H), 5.91 (d, *J* = 15.6 Hz, 1H), 5.55 (q, *J* = 7.2 Hz, 1H), 5.21 (s, 2H), 5.02 (s, 2H), 2.61 (d, *J* = 12.4 Hz, 1H), 2.21 – 2.13 (m, 1H), 2.05 – 2.02 (m, 1H), 1.86 (d, *J* = 7.2 Hz, 3H), 1.79 – 1.75 (m, 1H), 1.70 – 1.60 (m, 1H), 1.27 (s, 3H), 1.21 (s, 3H); **^{13}C NMR (100 MHz, $CDCl_3$)** δ 173.3, 167.3, 161.1 (d, *J* = 244.6 Hz), 143.6 (d, *J* = 3.3 Hz), 143.5, 143.4, 136.0 (d, *J* = 22.0 Hz), 135.1, 134.1, 128.6, 128.5, 128.3, 128.2, 128.2, 128.1, 117.1, 114.4 (d, *J* = 20.7 Hz), 66.3, 66.1, 41.2, 32.4, 28.3, 25.0, 24.7, 14.1; **^{19}F NMR (376 MHz, $CDCl_3$)** δ -117.7; **HRMS (ESI) [M+Na]$^+$** Calcd for $C_{33}H_{35}O_4FNa$: 537.24116 m/z, Found: 537.24052 m/z; **Specific rotation**: $[\alpha]_D^{28.4}$ -71.2 (*c* 1.00, $CHCl_3$) for an enantiomerically enriched sample of 96:4 e.r.

Enantiomeric purity of **4k** was determined by HPLC analysis in comparison with authentic racemic material (96:4 e.r. shown; Chiralcel OZ-H column, 95:5 hexane/ *i*-PrOH, 0.5 mL/min, 254 nm).

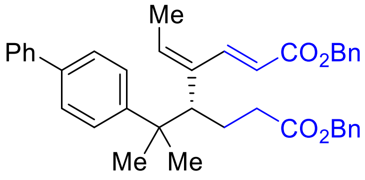

4l

Dibenzyl (*S*,2*E*,4*E*)-5-(2-([1,1'-biphenyl]-4-yl)propan-2-yl)-4-ethylideneoct-2-enedioate (4l)

Colorless oil, 52% yield. **IR (neat):** 3664 (w), 2969 (w), 1731 (m), 1713 (m), 1620 (w), 1486 (w), 1453 (w), 1380 (w), 1312 (w), 1271 (m), 1216 (w), 1160 (s), 1078 (w), 1041 (w), 1007 (w), 980 (w), 909 (w), 867 (w), 838 (w), 766 (w), 735 (m), 697 (s), 655 (w), 631 (w) cm^{-1}; **^{1}H NMR (400 MHz, CDCl3)** δ 7.83 (d, *J* = 16.0 Hz, 1H), 7.61 (d, *J* = 7.2 Hz, 2H), 7.53 (d, *J* = 8.0 Hz, 2H), 7.47 – 7.44 (m, 2H), 7.37 – 7.34 (m, 8H), 7.32 – 7.26 (m, 5H), 5.97 (d, *J* = 15.6 Hz, 1H), 5.64 (q, *J* = 7.2 Hz, 1H), 5.20 (d, *J* = 2.4 Hz, 2H), 5.04 (s, 2H), 2.71 (d, *J* = 12.4 Hz, 1H), 2.24 – 2.17 (m, 1H), 2.11 – 2.03 (m, 1H), 1.91 (d, *J* = 7.2 Hz, 3H), 1.89 – 1.79 (m, 1H), 1.74 – 1.62 (m, 1H), 1.35 (s, 3H), 1.28 (s, 3H); **^{13}C NMR (100 MHz, CDCl3)** δ 173.4, 167.3, 147.1, 143.5, 140.8, 138.6, 136.1, 135.9, 135.1, 134.1, 128.7, 128.53, 128.49, 128.3, 128.2, 128.1, 127.1, 127.04, 126.96, 126.4, 117.0, 66.1, 41.4, 32.5, 28.3, 25.0, 24.4, 14.1; **HRMS (ESI) [M+Na]$^+$** Calcd for $C_{39}H_{40}O_4Na$: 595.28188 m/z, Found: 595.28156 m/z; **Specific rotation**: $[\alpha]_D^{29.4}$ -55.5 (*c* 1.00, $CHCl_3$) for an enantiomerically enriched sample of 96:4 e.r.

Enantiomeric purity of **4l** was determined by HPLC analysis in comparison with authentic racemic material (96:4 e.r. shown; Chiralpak IA column, 95:5 hexane/ *i*-PrOH, 0.7 mL/min, 254 nm).

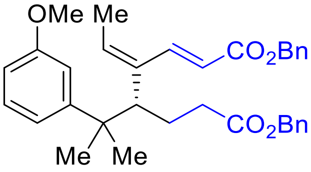

4m

Dibenzyl (*S*,2*E*,4*E*)-4-ethylidene-5-(2-(3-methoxyphenyl)propan-2-yl)oct-2-enedioate (4m)

Pale yellow oil, 89% yield. **IR (neat):** 3032 (w), 2963 (w), 1731 (m), 1713 (m), 1605 (w), 1581 (w), 1490 (w), 1454 (w), 1430 (w), 1378 (w), 1316 (w), 1270 (m), 1212 (w), 1159 (s), 1084 (w), 1047 (w), 1007 (w), 980 (w), 868 (w), 781 (w), 748 (m), 698 (m), 651 (w), 613 (w) cm^{-1}; **^{1}H NMR (400 MHz, CDCl$_3$)** δ 7.79 (d, *J* = 16.0 Hz, 1H), 7.45 – 7.38 (m, 4H), 7.38 – 7.31 (m, 4H), 7.31 – 7.27 (m, 2H), 7.22 – 7.18 (m, 1H), 6.94 – 6.88 (m, 1H), 6.87 – 6.86 (m, 1H), 6.71 (dd, *J* =8.0, 2.4 Hz, 1H), 5.95 (d, *J* = 16.0 Hz, 1H), 5.62 (q, *J* = 7.2 Hz, 1H), 5.21 (d, *J* = 1.2 Hz, 2H), 5.02 (s, 2H), 3.78 (s, 3H), 2.66 (dd, *J* = 12.4, 3.2 Hz, 1H), 2.21 – 2.13 (m, 1H), 2.07 – 1.99 (m, 1H), 1.88 (d, *J* = 7.2 Hz, 3H), 1.80 – 1.76 (m, 1H), 1.67 – 1.62 (m, 1H), 1.28 (s, 3H), 1.20 (s, 3H); **^{13}C NMR (100 MHz, CDCl$_3$)** δ 173.3, 167.3, 159.2, 149.9, 143.6, 136.1, 135.9, 135.1, 134.0, 128.7, 128.5, 128.5, 128.3, 128.2, 128.1, 119.2, 117.0, 113.6, 110.2, 66.2, 66.0, 55.1, 41.6, 32.5, 28.4, 25.0, 24.1, 14.1; **HRMS (ESI) [M+Na]$^+$** Calcd for $C_{34}H_{38}O_5Na$: 549.26115 m/z, Found: 549.26052 m/z; **Specific rotation**: $[\alpha]_D^{29.5}$ -75.3 (*c* 1.00, CHCl$_3$) for an enantiomerically enriched sample of 95:5 e.r.

Enantiomeric purity of **4m** was determined by HPLC analysis in comparison with authentic racemic material (95:5 e.r. shown; Chiralcel OZ-H column, 95:5 hexane/ *i*-PrOH, 0.7 mL/min, 254 nm).

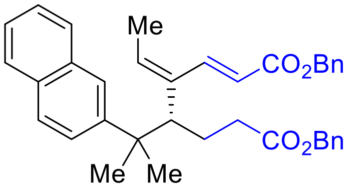

4n

Dibenzyl (*S*,2*E*,4*E*)-4-ethylidene-5-(2-(naphthalen-2-yl)propan-2-yl)oct-2-enedioate (4n)

Pale yellow oil, 89% yield. **IR (neat):** 3891 (w), 3032 (w), 2964 (w), 1730 (m), 1713 (m), 1620 (w), 1500 (w), 1454 (w), 1375 (w), 1313 (w), 1271 (m), 1214 (w), 1160 (s), 1084 (w), 1006 (w), 980 (w), 891 (w), 864 (w), 818 (w), 748 (m), 697 (m), 653 (w), 632 (w) cm^{-1}; **^{1}H NMR (400 MHz, $CDCl_3$)** δ 7.87 – 7.73 (m, 4H), 7.69 (s, 1H), 7.51 – 7.46 (m, 1H), 7.49 – 7.42 (m, 2H), 7.40 – 7.38 (m, 4H), 7.37 – 7.30 (m, 2H), 7.30 – 7.26 (m, 2H), 7.23 – 7.19 (m, 2H), 6.02 (d, *J* = 16.0 Hz, 1H), 5.62 (q, *J* = 7.2 Hz, 1H), 5.18 (d, *J* = 2.4 Hz, 2H), 4.96 (s, 2H), 2.79 (dd, *J* = 12.4, 3.2 Hz, 1H), 2.18 – 2.10 (m, 1H), 2.05 – 1.96 (m, 1H), 1.86 (d, *J* = 7.2 Hz, 3H), 1.80 – 1.72 (m, 1H), 1.72 – 1.54 (m, 1H), 1.39 (s, 3H), 1.29 (s, 3H); **^{13}C NMR (100 MHz, $CDCl_3$)** δ 173.4, 167.4, 145.6, 143.8, 136.2, 135.9, 135.2, 134.3, 133.1, 131.8, 128.6, 128.5, 128.4, 128.3, 128.2, 128.2, 128.1, 127.4, 127.3, 125.9, 125.5, 125.3, 125.2, 117.2, 66.3, 66.1, 41.8, 32.6, 28.8, 25.1, 24.0, 14.2; **HRMS (ESI) $[M+Na]^+$** Calcd for $C_{37}H_{38}O_4Na$: 569.26623 m/z, Found: 569.26686 m/z; **Specific rotation**: $[\alpha]_D^{29.3}$ -79.0 (*c* 1.00, $CHCl_3$) for an enantiomerically enriched sample of 96:4 e.r.

Enantiomeric purity of **4n** was determined by HPLC analysis in comparison with authentic racemic material (96:4 e.r. shown; Chiralpak IA column, 95:5 hexane/ *i*-PrOH, 0.7 mL/min, 254 nm).

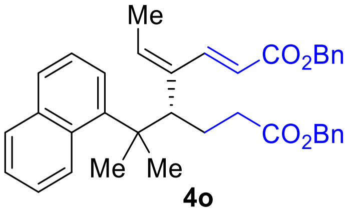

4o

Dibenzyl (*S*,2*E*,4*E*)-4-ethylidene-5-(2-(naphthalen-1-yl)propan-2-yl)oct-2-enedioate (4o)

Colorless oil, 80% yield. **IR (neat):** 3779 (w), 3035 (w), 2962 (w), 1716 (m), 1621 (w), 1497 (w), 1454 (w), 1384 (w), 1311 (w), 1269 (m), 1213 (w), 1160 (s), 1081 (w), 981 (w), 908 (w), 868 (w), 803 (w), 778 (m), 739 (m), 697 (m), 662 (w) cm^{-1}; **^{1}H NMR (400 MHz, $CDCl_3$)** δ 8.49 (d, *J* = 8.8 Hz, 1H), 7.80 (dd, *J* = 8.0, 1.6 Hz, 1H), 7.66 (d, *J* = 8.0 Hz, 2H), 7.46 – 7.30 (m, 11H), 7.30 – 7.27 (m, 1H), 7.19 – 7.16 (m, 2H), 5.88 (s, 2H), 5.17 (s, 2H), 4.93 – 4.80 (m, 2H), 3.59 (dd, *J* = 12.0, 3.2 Hz, 1H), 2.20 – 2.13 (m, 1H), 2.07 – 1.93 (m, 1H), 1.93 – 1.78 (m, 5H), 1.57 (s, 3H), 1.46 (s, 3H); **^{13}C NMR (100 MHz, $CDCl_3$)** δ 173.2, 166.9, 144.2, 143.9, 136.2, 135.8, 135.5, 135.1, 132.9, 131.4, 129.9, 128.5, 128.4, 128.1, 128.1, 128.0, 127.9, 126.2, 125.4, 124.9, 124.7, 124.6, 118.4, 66.0, 65.9, 43.2, 32.8, 27.6, 26.5, 24.8, 14.4; **HRMS (ESI) $[M+Na]^+$** Calcd for $C_{37}H_{38}O_4Na$: 569.26623 m/z, Found: 569.26603 m/z; **Specific rotation**: $[\alpha]_D^{29.6}$ -138.9 (*c* 1.00, $CHCl_3$) for an enantiomerically enriched sample of 98:2 e.r.

Enantiomeric purity of **4o** was determined by HPLC analysis in comparison with authentic racemic material (98:2 e.r. shown; Chiralpak IC column, 95:5 hexane/ *i*-PrOH, 0.5 mL/min, 254 nm).

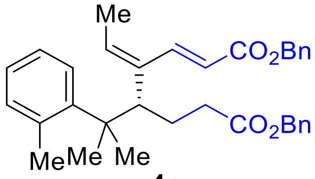

4p

Dibenzyl (*S*,2*E*,4*E*)-4-ethylidene-5-(2-(o-tolyl)propan-2-yl)oct-2-enedioate (4p)

Colorless oil, 92% yield. **IR (neat):** 3033 (w), 2968 (w), 1731 (m), 1715 (m), 1621 (w), 1496 (w), 1454 (w), 1379 (w), 1313 (w), 1268 (m), 1214 (w), 1159 (s), 1084 (w), 1014 (w), 980 (w), 907 (w), 867 (w), 802 (w), 751 (m), 731 (w), 697 (m), 653 (w), 615 (w) cm^{-1}; **^{1}H NMR (400 MHz, $CDCl_3$)** δ 7.60 (d, *J* = 15.6 Hz, 1H), 7.40 –7.37 (m, 4H), 7.36 – 7.27 (m, 4H), 7.26 – 7.19 (m, 3H), 7.10 – 7.04 (m, 1H), 7.04 – 6.99 (m, 2H), 5.84 (d, *J* = 15.6 Hz, 1H), 5.78 (q, *J* = 7.2 Hz, 1H), 5.18 (s, 2H), 5.01 (s, 2H), 3.03 (dd, *J* = 11.6, 4.0 Hz, 1H), 2.53 (s, 3H), 2.24 – 2.17 (m, 1H), 2.09 – 2.01 (m, 1H), 1.86 (d, *J* = 7.2 Hz, 3H), 1.76 – 1.67 (m, 2H), 1.41 (s, 3H), 1.26 (s, 3H); **^{13}C NMR (100 MHz, $CDCl_3$)** δ 173.4, 167.1, 146.0, 143.8, 136.2, 135.9, 135.3, 133.3, 133.1, 128.49, 128.47, 128.2, 128.12, 128.11, 128.07, 126.1, 125.6, 117.6, 66.1, 66.0, 43.0, 32.6, 27.1, 26.3, 24.5, 24.2, 14.4; **HRMS (ESI) $[M+Na]^+$** Calcd for $C_{34}H_{38}O_4Na$: 533.26623 m/z, Found: 533.26552 m/z; **Specific rotation**: $[\alpha]_D^{29.5}$ -94.9 (*c* 1.00, $CHCl_3$) for an enantiomerically enriched sample of 98:2 e.r.

Enantiomeric purity of **4p** was determined by HPLC analysis in comparison with authentic racemic material (98:2 e.r. shown; Chiralcel OZ-H column, 95:5 hexane/ *i*-PrOH, 0.5 mL/min, 300 nm).

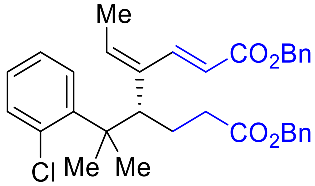

4q

Dibenzyl (*S*,2*E*,4*E*)-5-(2-(2-chlorophenyl)propan-2-yl)-4-ethylideneoct-2-enedioate (4q)

Pale yellow oil, 71% yield. **IR (neat):** 3823 (w), 3672 (w), 2972 (m), 2902 (w), 2358 (w), 1733 (m), 1714 (m), 1621 (w), 1497 (w), 1468 (w), 1454 (w), 1428 (w), 1380 (w), 1315 (w), 1271 (m), 1216 (w), 1160 (s), 1103 (w), 1078 (w), 1034 (m), 981 (w), 903 (w), 869 (w), 803 (w), 751

(m), 697 (m), 674 (w), 648 (w), 632 (w) cm^{-1}; **^{1}H NMR (400 MHz, $CDCl_3$)** δ 7.69 (d, *J* = 16.0 Hz, 1H), 7.41 – 7.37 (m, 4H), 7.36 – 7.26 (m, 7H), 7.25 – 7.24 (m, 1H), 7.18 – 7.13 (m, 1H), 7.09 – 7.05 (m, 1H), 6.14 (d, *J* = 16.0 Hz, 1H), 5.87 (q, *J* = 7.2 Hz, 1H), 5.20 (d, *J* = 2.0 Hz, 2H), 5.01 (d, *J* = 2.0 Hz, 2H), 3.65 (dd, *J* = 12.4, 4.0 Hz, 1H), 2.24 – 2.16 (m, 1H), 2.12 – 2.04 (m, 1H), 1.89 (d, *J* = 7.2 Hz, 3H), 1.80 – 1.69 (m, 1H), 1.56 – 1.54 (m, 1H), 1.41 (s, 3H), 1.32 (s, 3H); **^{13}C NMR (100 MHz, $CDCl_3$)** δ 173.3, 167.3, 144.8, 143.5, 136.3, 135.9, 135.3, 133.9, 133.0, 132.2, 129.5, 128.5, 128.4, 128.1, 128.1, 128.0, 127.5, 126.6, 118.3, 66.0, 66.0, 43.1, 32.7, 25.7, 24.8, 24.6, 14.4; **HRMS (ESI) [M+Na]$^+$** Calcd for $C_{33}H_{35}O_4NaCl$: 553.21161 m/z, Found: 553.21081 m/z; **Specific rotation**: $[\alpha]_D^{28.6}$ -94.3 (*c* 1.00, $CHCl_3$)for an enantiomerically enriched sample of 98:2 e.r.

Enantiomeric purity of **4q** was determined by HPLC analysis in comparison with authentic racemic material (98:2 e.r. shown; Chiralcel OZ-H column, 95:5 hexane/ *i*-PrOH, 0.5 mL/min, 254 nm).

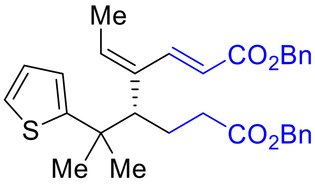

4r

Dibenzyl (*S*,2*E*,4*E*)-4-ethylidene-5-(2-(thiophen-2-yl)propan-2-yl)oct-2-enedioate (4r)

Pale yellow oil, 83% yield. **IR (neat):** 2962 (w), 2926 (w), 2854 (w), 1732 (m), 1714 (m), 1620 (w), 1455 (w), 1379 (w), 1312 (w), 1261 (s), 1215 (w), 1160 (s), 1089 (s), 1019 (s), 982 (w), 865 (w), 799 (s), 749 (w), 696 (s), 633 (w), 614 (w) cm^{-1}; **^{1}H NMR (400 MHz, $CDCl_3$)** δ 7.81 (d, *J* = 15.6 Hz, 1H), 7.44 – 7.39 (m, 3H), 7.39 – 7.34 (m, 3H), 7.34 – 7.28 (m, 4H), 7.11 (dd, *J* = 5.2, 1.2 Hz, 1H), 6.90 (dd, *J* = 5.2, 3.2 Hz, 1H), 6.79 (dd, *J* = 3.2, 1.2 Hz, 1H), 5.95 (d, *J* = 15.6 Hz, 1H), 5.58 (q, *J* = 7.2 Hz, 1H), 5.22 (s, 2H), 5.06 (d, *J* = 1.6 Hz, 2H), 2.64 (dd, *J* = 12.4, 3.2 Hz, 1H), 2.25 – 2.18 (m, 1H), 2.13 – 2.05 (m, 1H), 2.05 – 1.95 (m, 1H), 1.89 (d, *J* = 7.2 Hz, 3H), 1.75 – 1.69 (m, 1H), 1.34 (s, 3H), 1.30 (s, 3H); **^{13}C NMR (100 MHz, $CDCl_3$)** δ173.3, 167.3, 153.7, 143.2, 136.1, 135.9, 135.0, 134.1, 128.50, 128.47, 128.3, 128.15, 128.12, 126.2, 123.6, 122.9, 116.9, 66.2, 66.1, 41.1, 32.5, 30.3, 26.0, 25.2, 14.1; **HRMS (ESI) [M+Na]$^+$** Calcd for $C_{31}H_{34}O_4NaS$: 525.20700 m/z, Found: 525.20659 m/z; **Specific rotation**: $[\alpha]_D^{29.5}$ -61.3 (*c* 1.00, $CHCl_3$) for an enantiomerically enriched sample of 94:6 e.r.

Enantiomeric purity of **4r** was determined by HPLC analysis in comparison with authentic racemic material (94:6 e.r. shown; Chiralcel OZ-H column, 95:5 hexane/ *i*-PrOH, 0.5 mL/min, 254 nm).

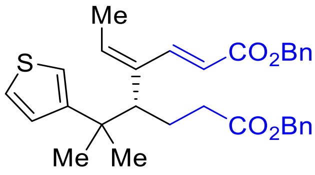

4s

Dibenzyl (*S*,2*E*,4*E*)-4-ethylidene-5-(2-(thiophen-3-yl)propan-2-yl)oct-2-enedioate (4s)

Colorless oil, 72% yield. **IR (neat):** 3034 (w), 2968 (w), 1731 (m), 1714 (m), 1620 (w), 1497 (w), 1454 (w), 1413 (w), 1377 (w), 1311 (w), 1271 (w), 1213 (w), 1160 (s), 1084 (w), 1006 (w), 980 (w), 910 (w), 865 (w), 784 (m), 740 (m), 697 (m), 676 (w), 662 (w), 619 (w) cm^{-1}; **^{1}H NMR (400 MHz, $CDCl_3$)** δ 7.76 (d, *J* = 15.6 Hz, 1H), 7.41 – 7.37 (m, 4H), 7.34 – 7.32 (m, 2H), 7.31 – 7.27 (m, 4H), 7.17 (dd, *J* = 5.2, 2.8 Hz, 1H), 6.96 (dd, *J* = 5.2, 1.6 Hz, 1H), 6.88 (dd, *J* = 3.2, 1.6 Hz, 1H), 5.89 (d, *J* = 16.0 Hz, 1H), 5.48 (q, *J* = 7.2 Hz, 1H), 5.19 (s, 2H), 5.03 (d, *J* = 1.6 Hz, 2H), 2.57 (d, *J* = 12.4 Hz, 1H), 2.22 – 2.14 (m, 1H), 2.09 – 2.01 (m, 1H), 1.94 – 1.89 (m, 1H), 1.85 (d, *J* = 7.2 Hz, 3H), 1.65 – 1.56 (m, 1H), 1.25 (s, 3H), 1.21 (s, 3H); **^{13}C NMR (100 MHz, $CDCl_3$)** δ 173.4, 167.3, 149.5, 143.3, 136.1, 135.9, 135.2, 133.9, 128.52, 128.49, 128.3, 128.2, 128.1, 127.0, 124.6, 120.0, 116.8, 66.2, 66.1, 40.2, 32.4, 28.5, 25.5, 25.2, 14.1; **HRMS (ESI) $[M+Na]^+$** Calcd for $C_{31}H_{34}O_4NaS$: 525.20700 m/z, Found: 525.20642 m/z; **Specific rotation**: $[\alpha]_D^{29.5}$ -37.9 (*c* 1.00, $CHCl_3$) for an enantiomerically enriched sample of 95:5 e.r.

Enantiomeric purity of **4s** was determined by HPLC analysis in comparison with authentic racemic material (95:5 e.r. shown; Chiralcel OZ-H column, 95:5 hexane/ *i*-PrOH, 0.5 mL/min, 290 nm).

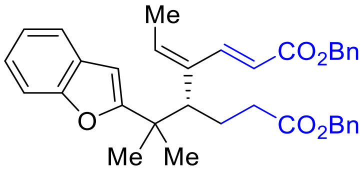

4t

Dibenzyl (S,2E,4E)-5-(2-(benzofuran-2-yl)propan-2-yl)-4-ethylideneoct-2-enedioate (4t)

Colorless oil, 82% yield.Colorless oil. **IR (neat):** 3062 (w), 3028 (w), 2930 (w), 2910 (w), 2869 (w), 2178 (w), 2135 (w), 2015 (w), 1727 (s), 1681 (m), 1608 (s), 1496 (m), 1451 (w), 1433 (m), 1371 (w), 1275 (m), 1236 (w), 1211 (s), 1153 (s), 1126 (m), 1030 (m), 921 (w), 865 (w), 815

(w), 778 (w), 740 (w), 696 (m), 662 (w), 643 (w), 623 (w) cm^{-1}; **^{1}H NMR (400 MHz, CDCl$_3$)** δ 7.76 (d, *J* = 15.6 Hz, 1H), 7.50 – 7.45 (m, 1H), 7.44 – 7.38 (m, 5H), 7.38 – 7.33 (m, 2H), 7.32 – 7.31 (m, 2H), 7.29 – 7.27 (m, 2H), 7.23 – 7.15 (m, 2H), 6.36 (d, *J* = 0.8 Hz, 1H), 6.03 (d, *J* = 15.6 Hz, 1H), 5.70 (q, *J* = 7.2 Hz, 1H), 5.16 (d, *J* = 2.4 Hz, 2H), 5.04 (s, 2H), 2.91 (dd, *J* = 12.4, 3.2 Hz, 1H), 2.28 – 2.20 (m, 1H), 2.16 – 2.08 (m, 1H), 1.99 – 1.91 (m, 1H), 1.87 (d, *J* = 7.2 Hz, 3H), 1.84 – 1.70 (m, 1H), 1.32 (s, 3H), 1.28 (s, 3H); **^{13}C NMR (100 MHz, CDCl$_3$)** δ 173.3, 167.3, 164.6, 154.3, 142.9, 136.1, 135.9, 134.9, 133.9, 128.53, 128.48, 128.3, 128.2, 128.14, 128.12, 123.3, 122.4, 120.4, 117.2, 110.9, 102.0, 66.2, 66.1, 40.0, 32.5, 26.0, 25.1, 23.2, 14.1; **HRMS (ESI) [M+Na]$^+$** Calcd for $C_{35}H_{36}O_5Na$: 559.24550 m/z, Found: 559.24545 m/z; **Specific rotation**: $[\alpha]_D^{28.7}$ -53.2 (*c* 1.00, $CHCl_3$) for an enantiomerically enriched sample of 94:6 e.r.

Enantiomeric purity of **4t** was determined by HPLC analysis in comparison with authentic racemic material (94:6 e.r. shown; Chiralpak IA column, 95:5 hexane/ *i*-PrOH, 0.5 mL/min, 254 nm).

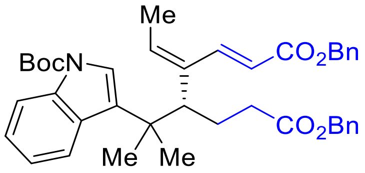

4u

Dibenzyl (*S*,2*E*,4*E*)-5-(2-(1-(tert-butoxycarbonyl)-1H-indol-3-yl)propan-2-yl)-4-ethylideneoct-2-enedioate (4u)

Colorless oil, 72% yield. **IR (neat):** 2973 (w), 1728 (m), 1620 (w), 1497 (w), 1452 (w), 1372 (m), 1308 (w), 1269 (m), 1156 (s), 1117 (w), 1063 (w), 1026 (w), 981 (w), 910 (w), 862 (w), 766 (w), 745 (m), 697 (m), 648 (w) cm^{-1}; **^{1}H NMR (400 MHz, CDCl$_3$)** δ 8.17 (d, *J* = 8.4 Hz, 1H), 7.84 (d, *J* = 15.6 Hz, 1H), 7.75 (d, *J* = 8.0 Hz, 1H), 7.44 – 7.40 (m, 3H), 7.40 – 7.35 (m, 2H), 7.35 – 7.27 (m, 5H), 7.24 – 7.19 (m, 3H), 6.07 (d, *J* = 15.6 Hz, 1H), 5.85 (q, *J* = 7.2 Hz, 1H), 5.23 (s,2H), 4.98 (d, *J* = 12.4 Hz, 1H), 4.90 (d, *J* = 12.4 Hz, 1H), 3.10 (t, *J* = 7.6 Hz, 1H), 2.27 – 2.13 (m, 1H), 2.07 – 1.99 (m, 1H), 1.91 (d, *J* = 7.2 Hz, 3H), 1.80 – 1.74 (m, 2H), 1.69 (s, 9H), 1.38 (s, 3H), 1.31 (s, 3H); **^{13}C NMR (100 MHz, CDCl$_3$)** δ 173.3, 167.2, 149.7, 143.8, 136.1, 135.8, 135.2, 133.9, 128.9, 128.5, 128.4, 128.3, 128.2, 128.13, 128.08, 128.06, 123.8, 122.9, 122.1, 121.3, 117.4, 115.5, 83.5, 66.2, 66.0, 38.9, 32.7, 28.2, 27.4, 25.1, 23.9, 14.3; **HRMS (ESI) [M+Na]$^+$** Calcd for $C_{40}H_{45}NO_6Na$: 658.31391 m/z, Found: 658.31374 m/z; **Specific rotation**: $[\alpha]_D^{28.8}$ -99.4 (*c* 1.00, $CHCl_3$) for an enantiomerically enriched sample of 97:3 e.r.

Enantiomeric purity of **4u** was determined by HPLC analysis in comparison with authentic racemic material (97:3 e.r. shown; Chiralcel IF column, 95:5 hexane/ *i*-PrOH, 0.5 mL/min, 290 nm).

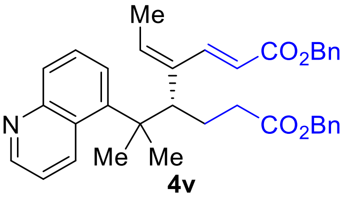

4v

Dibenzyl (*S*,2*E*,4*E*)-4-ethylidene-5-(2-(quinolin-6-yl)propan-2-yl)oct-2-enedioate (4v)

Colorless oil, 60% yield. **IR (neat):** 3418 (br), 2922 (w), 2852 (w), 1727 (m), 1622 (w), 1572 (w), 1500 (w), 1455 (w), 1379 (w), 1312 (w), 1270 (m), 1212 (w), 1162 (s), 1083 (w), 982 (w), 867 (w), 802 (m), 745 (m), 697 (s), 638 (w) cm^{-1}; **^{1}H NMR (400 MHz, $CDCl_3$)** δ 8.86 – 8.78 (m, 2H), 7.97 (d, *J* = 8.4 Hz, 1H), 7.61 – 7.57 (m, 1H), 7.46 (d, *J* = 7.6 Hz, 1H), 7.44 – 7.35 (m, 5H), 7.35 – 7.27 (m, 5H), 7.21 – 7.19 (m, 2H), 5.83 (s, 2H), 5.17 (s, 2H), 4.95 (d, *J* = 12.4 Hz, 1H), 4.86 (d, *J* = 12.4 Hz, 1H), 3.46 – 3.30 (m, 1H), 2.22 – 2.16 (m, 1H), 2.03 – 1.99 (m, 1H), 1.84 (d, *J* = 7.2 Hz, 4H), 1.73 – 1.65 (m, 1H), 1.58 (s, 3H), 1.45 (s, 3H); **^{13}C NMR (100 MHz, $CDCl_3$)** δ 173.1, 166.7, 149.8, 149.0, 144.9, 136.0, 135.7, 135.1, 134.2, 133.2, 129.2, 128.53, 128.46, 128.3, 128.2, 128.13, 128.06, 126.6, 125.8, 119.4, 66.2, 66.1, 43.3, 32.5, 27.7, 26.8, 24.6, 14.5; **HRMS (ESI) $[M+H]^+$** Calcd for $C_{36}H_{38}NO_4$: 548.27954 m/z, Found: 548.27854 m/z; **Specific rotation**: $[\alpha]_D^{29.5}$ -84.9 (*c* 1.00, $CHCl_3$) for an enantiomerically enriched sample of 99:1 e.r.

Enantiomeric purity of **4v** was determined by HPLC analysis in comparison with authentic racemic material (99:1 e.r. shown; Chiralcel OZ-H column, 90:10 hexane/ *i*-PrOH, 1.0 mL/min, 254 nm).

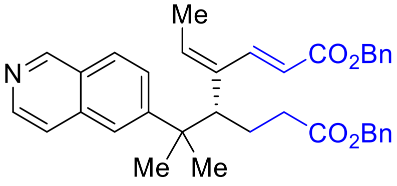

4w

Dibenzyl (*S*,2*E*,4*E*)-4-ethylidene-5-(2-(isoquinolin-6-yl)propan-2-yl)oct-2-enedioate (4w)

Colorless oil, 61% yield. **IR (neat):** 3424 (br), 3062 (w), 2963 (w), 1713 (m), 1627 (w), 1495 (w), 1455 (w), 1439 (w), 1375 (w), 1313 (w), 1272 (m), 1214 (w), 1162 (s), 1117 (w), 1093 (w), 1002 (w), 980 (w), 952 (w), 891 (w), 868 (w), 831 (w), 749 (w), 724 (m), 697 (s), 643 (w), 621 (w) cm^{-1}; **^{1}H NMR (400 MHz, $CDCl_3$)** δ 9.18 (s, 1H), 8.50 (d, *J* = 5.6 Hz, 1H), 7.87 (d, *J* = 8.8

Hz, 1H), 7.77 (d, J = 16.0 Hz, 1H), 7.68 – 7.65 (m, 1H), 7.64 – 7.56 (m, 2H), 7.40 – 7.36 (m, 5H), 7.29 – 7.26 (m, 3H), 7.22 (dd, J = 6.8, 3.2 Hz, 2H), 5.97 (d, J = 16.0 Hz, 1H), 5.66 (q, J = 7.2 Hz, 1H), 5.17 (s, 2H), 4.98 (s, 2H), 2.91 – 2.72 (m, 1H), 2.21 – 2.13 (m, 1H), 2.05 – 1.97 (m, 1H), 1.87 (d, J = 7.2 Hz, 3H), 1.75 – 1.64 (m, 2H), 1.41 (s, 3H), 1.29 (s, 3H); **^{13}C NMR (100 MHz, $CDCl_3$)** δ 173.1, 167.1, 151.8, 150.8, 143.4, 143.0, 136.0, 135.7, 135.5, 134.7, 134.3, 128.6, 128.5, 128.3, 128.2, 128.2, 128.1, 127.1, 127.0, 126.7, 123.6, 120.6, 117.2, 66.3, 66.1, 42.2, 32.3, 28.2, 24.9, 23.9, 14.2; **HRMS (ESI) [M+H]$^+$** Calcd for $C_{36}H_{38}NO_4$: 548.27954 m/z, Found: 548.27961 m/z; **Specific rotation**: $[\alpha]_D^{28.5}$ -78.5 (c 1.00, $CHCl_3$) for an enantiomerically enriched sample of 96:4 e.r.

Enantiomeric purity of **4w** was determined by HPLC analysis in comparison with authentic racemic material (96:4 e.r. shown; Chiralcel IF column, 92:8 hexane/ i-PrOH, 1.0 mL/min, 254 nm).

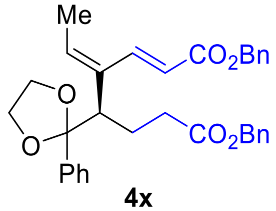

4x

Dibenzyl (R,2E,4E)-4-ethylidene-5-(2-phenyl-1,3-dioxolan-2-yl)oct-2-enedioate (4x)

Colorless oil, 50% yield. **IR (neat):** 3062 (w), 3031 (w), 2950 (w), 2886 (w), 1729 (m), 1710 (m), 1622 (w), 1495 (w), 1450 (w), 1376 (w), 1313 (w), 1269 (m), 1213 (w), 1154 (s), 1077 (w), 1026 (m), 976 (m), 914 (w), 863 (w), 823 (w), 739 (m), 696 (s) 658 (w), 579 (w), 505 (w), 455 (w); **^{1}H NMR (400 MHz, $CDCl_3$)** δ 7.62 (d, J = 15.8 Hz, 1H), 7.43 – 7.27 (m, 11H), 7.27 – 7.14 (m, 4H), 5.92 (q, J = 7.2 Hz, 1H), 5.83 (d, J = 15.6 Hz, 1H), 5.16 (s, 2H), 5.04 (s, 2H), 3.93 (s, 2H), 3.70 (d, J = 5.2 Hz, 2H), 2.92 (dd, J = 11.6, 3.2 Hz, 1H), 2.28 – 2.20 (m, 1H), 2.18 – 2.08 (m, 2H), 1.92 – 1.83 (m, 1H), 1.80 (d, J = 7.2 Hz, 3H); **^{13}C NMR (100 MHz, $CDCl_3$)** δ 173.3, 167.3, 142.1, 141.2, 136.2, 136.0, 135.7, 133.5, 128.5, 128.2, 128.1, 128.1, 127.7, 127.6, 126.5, 117.1, 111.1, 66.0, 66.0, 64.8, 64.3, 32.0, 24.5, 14.2; **HRMS (ESI) [M+Na]$^+$** Calcd for $C_{33}H_{34}O_6Na$: 549.22476 m/z, Found: 549.22498 m/z; **Specific rotation**: $[\alpha]_D^{28.6}$ +12.1 (c 1.00, $CHCl_3$) for an enantiomerically enriched sample of 94:6 e.r.

Enantiomeric purity of **4x** was determined by HPLC analysis in comparison with authentic racemic material (94:6 e.r. shown; Chiralpak IB column, 95:5 hexane/ *i*-PrOH, 0.7 mL/min, 210 nm).

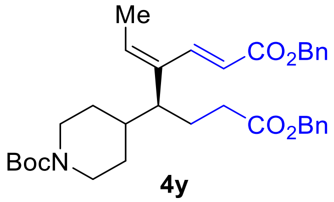

4y

Dibenzyl (*S*,2*E*,4*E*)-5-(1-(tert-butoxycarbonyl)piperidin-4-yl)-4-ethylideneoct-2-enedioate (4y)

Colorless oil, 50% yield. **IR (neat):** 3224 (w), 2963 (w), 2919 (w), 2850 (w), 1731 (w), 1690 (w), 1622 (w), 1453 (w), 1422 (w), 1365 (w), 1260 (m), 1160 (m), 1091 (m), 1018 (s), 867 (w), 798 (s), 748 (w), 698 (w), 659 (w), 632 (w) cm^{-1}; **^{1}H NMR (400 MHz, $CDCl_3$)** δ 7.74 (d, *J* = 16.0 Hz, 1H), 7.42 – 7.37 (m, 4H), 7.37 – 7.34 (m, 2H), 7.33 – 7.32 (m, 3H), 7.31 – 7.30 (m, 1H), 5.93 (d, *J* = 16.0 Hz, 1H), 5.78 (q, *J* = 7.2 Hz, 1H), 5.20 (s, 2H), 5.06 (d, *J* = 2.4 Hz, 2H), 4.07 (s, 2H), 2.71 – 2.48 (m, 2H), 2.32 – 2.19 (m, 1H), 2.19 – 1.96 (m, 3H), 1.88 (d, *J* = 7.2 Hz, 3H), 1.77 (d, *J* = 13.2 Hz, 1H), 1.70 – 1.64 (m, 1H), 1.47 (d, *J* = 3.2 Hz, 2H), 1.44 (s, 9H), 1.07 – 1.00 (m, 2H); **^{13}C NMR (100 MHz, $CDCl_3$)** δ 173.3, 167.2, 154.7, 141.5, 136.5, 136.0, 135.9, 133.6, 128.6, 128.5, 128.4, 128.3, 128.3, 128.2, 117.5, 79.3, 66.4, 66.2, 43.9, 43.1, 40.3, 32.1, 30.4, 28.4, 25.8, 14.1; **HRMS (ESI) $[M+Na]^+$** Calcd for $C_{34}H_{43}NO_6Na$: 584.29826 m/z, Found: 584.29798 m/z; **Specific rotation**: $[\alpha]_D^{28.5}$ +5.1 (*c* 1.00, $CHCl_3$) for an enantiomerically enriched sample of 94:6 e.r.

Enantiomeric purity of **4y** was determined by HPLC analysis in comparison with authentic racemic material (94:6 e.r. shown; Chiralcel OD-H column, 95:5 hexane/ *i*-PrOH, 0.3 mL/min, 254 nm).

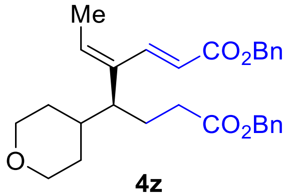

4z

Dibenzyl (*S*,2*E*,4*E*)-4-ethylidene-5-(tetrahydro-2H-pyran-4-yl)oct-2-enedioate (4z)

Colorless oil, 52% yield. **IR (neat):** 3031 (w), 2947 (w), 2844 (w), 1731 (m), 1714 (m), 1622 (w), 1497 (w), 1454 (w), 1379 (w), 1308 (w), 1270 (m), 1213 (w), 1160 (s), 1122 (w), 1092 (w),

1015 (w), 982 (w), 911 (w), 867 (w), 816 (w), 749 (m), 698 (m), 656 (w), 636 (w), 617 (w) cm^{-1}; **^{1}H NMR (400 MHz, $CDCl_3$)** δ 7.74 (d, *J* = 16.0 Hz, 1H), 7.43 – 7.37 (m, 4H), 7.36 – 7.34 (m, 2H), 7.33 (d, *J* = 3.6 Hz, 3H), 7.31 (d, *J* = 2.8 Hz, 1H), 5.95 (d, *J* = 16.0 Hz, 1H), 5.79 (q, *J* = 7.2 Hz, 1H), 5.20 (s, 2H), 5.07 (s, 2H), 3.97 (dd, *J* = 12.0, 4.0 Hz, 1H), 3.89 (dd, *J* = 11.6, 2.8 Hz, 1H), 3.35 – 3.23 (m, 2H), 2.27 – 2.18 (m, 1H), 2.21 – 1.98 (m, 3H), 1.88 (d, *J* = 7.2 Hz, 3H), 1.75 – 1.70 (m, 1H), 1.67 – 1.61 (m, 1H), 1.59 – 1.49 (m, 1H), 1.42 – 1.37 (m, 1H), 1.25 – 1.10 (m, 2H). **^{13}C NMR (100 MHz, $CDCl_3$)** δ 173.3, 167.2, 141.5, 136.3, 135.9, 135.9, 133.6, 128.6, 128.5, 128.4, 128.3, 128.2, 117.5, 68.1, 68.0, 66.4, 66.2, 39.2, 32.0, 31.3, 31.2, 25.6, 14.1; **HRMS (ESI) $[M+Na]^+$** Calcd for $C_{29}H_{34}O_5Na$: 485.22985 m/z, Found: 485.22942 m/z; **Specific rotation**: $[\alpha]_D^{29.6}$ -2.4 (*c* 1.00, $CHCl_3$) for an enantiomerically enriched sample of 95:5 e.r.

Enantiomeric purity of **4z** was determined by HPLC analysis in comparison with authentic racemic material (95:5 e.r. shown; Chiralpak IB column, 97:3 hexane/ *i*-PrOH, 0.3 mL/min, 254 nm).

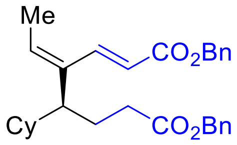

4aa

Dibenzyl (*S*,2*E*,4*E*)-5-cyclohexyl-4-ethylideneoct-2-enedioate (4aa)

Colorless oil, 55% yield. **IR (neat):** 2924 (w), 2851 (w), 1733 (m), 1714 (m), 1621 (w), 1497 (w), 1452 (w), 1377 (w), 1270 (m), 1213 (w), 1159 (s), 1083 (w), 1014 (w), 981 (w), 907 (w), 866 (w), 800 (w), 739 (m), 697 (m), 632 (w) cm^{-1}; **^{1}H NMR (400 MHz, $CDCl_3$)** δ 7.76 (d, *J* = 16.0 Hz, 1H), 7.43 – 7.37 (m, 4H), 7.36 – 7.26 (m, 6H), 5.93 (d, *J* = 16.0 Hz, 1H), 5.76 (q, *J* = 7.2 Hz, 1H), 5.20 (s, 2H), 5.07 (d, *J* = 1.6 Hz, 2H), 2.27 – 2.21 (m, 1H), 2.16 – 2.12 (m, 1H), 2.10 – 1.98 (m, 2H), 1.88 (d, *J* = 7.2 Hz, 3H), 1.83 (d, *J* = 12.8 Hz, 1H), 1.75 – 1.68 (m, 1H), 1.68 – 1.62 (m, 2H), 1.54 (d, *J* = 13.2 Hz, 1H), 1.33 – 1.27 (m, 1H), 1.19 (d, *J* = 3.2 Hz, 1H), 1.17 – 1.04 (m, 3H), 0.87 – 0.81 (m, 2H); **^{13}C NMR (100 MHz, $CDCl_3$)** δ 173.6, 167.5, 142.0, 137.2, 136.1, 136.0, 133.0, 128.6, 128.5, 128.3, 128.2, 128.2, 117.1, 66.1, 41.7, 32.3, 31.4, 30.7, 26.5, 26.4, 25.9, 14.1; **HRMS (ESI) $[M+Na]^+$** Calcd for $C_{30}H_{36}O_4Na$: 483.25058 m/z, Found: 483.24980 m/z; **Specific rotation**: $[\alpha]_D^{29.6}$ -2.1 (*c* 1.00, $CHCl_3$) for an enantiomerically enriched sample of 95:5 e.r.

Enantiomeric purity of **4aa** was determined by HPLC analysis in comparison with authentic racemic material (95:5 e.r. shown; Chiralcel OD-H column, 95:5 hexane/ *i*-PrOH, 0.5 mL/min, 290 nm).

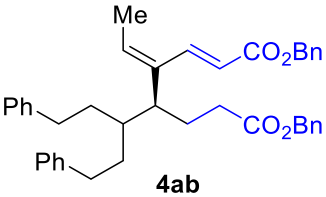

4ab

Dibenzyl (*S*,2*E*,4*E*)-5-(1,5-diphenylpentan-3-yl)-4-ethylideneoct-2-enedioate (4ab)

Colorless oil, 70% yield. **IR (neat):** 3062 (w), 3027 (w), 2928 (w), 2861 (w), 1731 (m), 1714 (m), 1620 (w), 1496 (w), 1453 (w), 1377 (w), 1270 (w), 1213 (w), 1159 (m), 1082 (w), 1026 (w), 982 (w), 909 (w), 866 (w), 804 (w), 746 (m), 697 (s), 647 (w), 619 (w) cm^{-1}; **^{1}H NMR (400 MHz, $CDCl_3$)** δ 7.70 (d, *J* = 16.0 Hz, 1H), 7.41 – 7.35 (m, 4H), 7.33 (dd, *J* = 4.4, 2.2 Hz, 1H), 7.32 – 7.27 (m, 5H), 7.25 – 7.19 (m, 4H), 7.17 – 7.08 (m, 6H), 5.90 (d, *J* = 16.0 Hz, 1H), 5.73 (q, *J* = 7.2 Hz, 1H), 5.19 (s, 2H), 5.06 (s, 2H), 2.68 – 2.52 (m, 3H), 2.50 – 2.39 (m, 2H), 2.30 – 2.22 (m, 1H), 2.16 – 2.06 (m, 1H), 2.04 – 1.98 (m, 1H), 1.85 (d, *J* = 7.2 Hz, 3H), 1.79 – 1.70 (m, 1H), 1.70 – 1.58 (m, 3H), 1.53 (d, *J* = 5.2 Hz, 2H). **^{13}C NMR (100 MHz, $CDCl_3$)** δ 173.4, 167.2, 142.6, 142.3, 141.7, 136.6, 136.1, 135.9, 133.3, 128.6, 128.5, 128.35, 128.33, 128.31, 128.24, 128.21, 128.20, 125.7, 117.4, 66.3, 66.2, 43.6, 40.2, 33.0, 32.9, 32.8, 32.1, 31.9, 25.1, 14.2; **HRMS (ESI) $[M+Na]^+$** Calcd for $C_{41}H_{44}O_4Na$: 623.31318 m/z, Found: 623.31397 m/z; **Specific rotation**: $[\alpha]_D^{28.4}$ -1.3 (*c* 1.00, $CHCl_3$) for an enantiomerically enriched sample of 96:4 e.r.

Enantiomeric purity of **4ab** was determined by HPLC analysis in comparison with authentic racemic material (96:4 e.r. shown; Chiralpak AD-H column, 95:5 hexane/ *i*-PrOH, 0.5 mL/min, 254 nm).

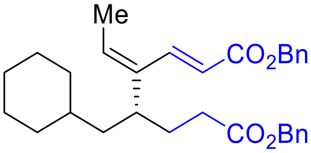

4ac

Dibenzyl (*R*,2*E*,4*Z*)-5-(cyclohexylmethyl)-4-ethylideneoct-2-enedioate (4ac)

Colorless oil, 43% yield. The product was afforded by column chromatography using silver nitrate on silica gel as the stationary phase (PE:EA = 20:1). **IR (neat):** 2961 (w), 2922 (w), 2849 (w), 1732 (m), 1715 (m), 1621 (w), 1497 (w), 1450 (w), 1378 (w), 1261 (m), 1212 (w), 1159 (m),

1087 (w), 1018 (s), 866 (w), 797 (s), 746 (w), 697 (w), 633 (w) cm^{-1}; **^{1}H NMR (400 MHz, $CDCl_3$)** δ 7.74 (d, *J* = 16.0 Hz, 1H), 7.38 (d, *J* = 6.8 Hz, 4H), 7.35 – 7.31 (m, 6H), 5.97 (d, *J* = 15.6 Hz, 1H), 5.79 (q, *J* = 7.2 Hz, 1H), 5.20 (d, *J* = 2.0 Hz, 2H), 5.07 (d, *J* = 2.4 Hz, 2H), 2.45 (s, 1H), 2.38 – 2.15 (m, 2H), 1.87 (d, *J* = 7.6 Hz, 3H), 1.82 (d, *J* = 6.8 Hz, 1H), 1.72 – 1.62 (m, 5H), 1.41 – 1.30 (m, 1H), 1.28 – 1.21 (m, 2H), 1.18 – 1.12 (m, 4H), 0.87 – 0.76 (m, 2H); **^{13}C NMR (100 MHz, $CDCl_3$)** δ 173.5, 167.5, 141.4, 138.3, 136.1, 135.9, 132.6, 128.6, 128.5, 128.3, 128.3, 128.2, 116.9, 66.3, 66.2, 42.7, 37.7, 34.9, 33.7, 33.3, 32.0, 30.0, 26.6, 26.2, 26.2, 14.1; **HRMS (ESI) $[M+Na]^+$** Calcd for $C_{31}H_{38}O_4Na$: 497.26623 m/z, Found: 497.26613 m/z; **Specific rotation**: $[\alpha]_D^{27.2}$ -1.8 (*c* 1.00, $CHCl_3$) for an enantiomerically enriched sample of 96:4 e.r.

Enantiomeric purity of **4ac** was determined by HPLC analysis in comparison with authentic racemic material (96:4 e.r. shown; Chiralpak IG+IF3 column, 95:5 hexane/ *i*-PrOH, 0.7 mL/min, 214 nm).

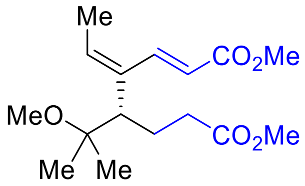

5a

Dimethyl (*S*,2*E*,4*E*)-4-ethylidene-5-(2-methoxypropan-2-yl)oct-2-enedioate (5a)

Pale yellow oil, 90% yield. **IR (neat):** 2976 (w), 2950 (w), 2829 (w), 1735 (m), 1720 (m), 1621 (w), 1436 (w), 1364 (w), 1328 (w), 1274 (m), 1168 (s), 1073 (w), 1039 (w), 983 (w), 921 (w), 868 (w), 813 (w), 722 (w), 632 (w) cm^{-1}; **^{1}H NMR (400 MHz, $CDCl_3$)** δ 7.76 (d, *J* = 15.6 Hz, 1H), 5.98 (t, *J* = 7.2 Hz, 1H), 5.93 (d, *J* = 15.6 Hz, 1H), 3.74 (s, 3H), 3.61 (s, 3H), 3.15 (s, 3H), 2.49 (dd, *J* = 12.4, 2.4 Hz, 1H), 2.24 – 2.15 (m, 1H), 2.14 – 2.03 (m, 2H), 1.89 (d, *J* = 7.2 Hz, 3H), 1.78 – 1.67 (m, 1H), 1.05 (s, 3H), 1.04 (s, 3H); **^{13}C NMR (100 MHz, $CDCl_3$)** δ 174.2, 167.9, 142.9, 135.7, 134.1, 117.5, 76.9, 51.5, 51.4, 49.0, 32.2, 24.3, 23.6, 21.8, 14.3; **HRMS (ESI) $[M+Na]^+$** Calcd for $C_{16}H_{26}O_5Na$: 321.16725 m/z, Found: 321.16662 m/z; **Specific rotation**: $[\alpha]_D^{25.0}$ -15.3 (*c* 1.00, $CHCl_3$) for an enantiomerically enriched sample of 94:6 e.r.

Enantiomeric purity of **5a** was determined by HPLC analysis in comparison with authentic racemic material (94:6 e.r. shown; Chiralcel OZ-H column, 99:1 hexane/ *i*-PrOH, 0.5 mL/min, 254 nm).

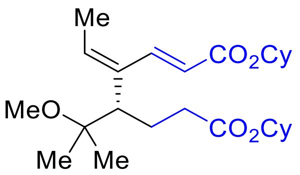

5b

Dicyclohexyl (*S*,2*E*,4*E*)-4-ethylidene-5-(2-methoxypropan-2-yl)oct-2-enedioate (5b)

Colorless oil, 81% yield. **IR (neat):** 2936 (m), 2859 (w), 1727 (m), 1621 (w), 1451 (w), 1380 (w), 1362 (w), 1321 (w), 1272 (m), 1171 (s), 1125 (w), 1075 (m), 1039 (w), 1016 (w), 982 (m), 913 (w), 892 (w), 866 (w), 843 (w), 803 (w), 658 (w) cm^{-1}; **^{1}H NMR (400 MHz, $CDCl_3$)** δ 7.77 (d, *J* = 15.6 Hz, 1H), 5.97 (q, *J* = 7.2 Hz, 1H), 5.91 (d, *J* = 15.6 Hz, 1H), 4.81 (m, 1H), 4.70 (m,1H), 3.18 (s, 3H), 2.59 – 2.47 (m, 1H), 2.26 – 2.13 (m, 1H), 2.13 – 2.00 (m, 2H), 1.90 (d, *J* = 7.2 Hz, 3H), 1.88 – 1.86 (m, 1H), 1.81 (d, *J* = 7.6 Hz, 2H), 1.77 – 1.63 (m, 5H), 1.56 – 1.51 (m, 2H), 1.47 – 1.15 (m, 11H), 1.07 (s, 3H), 1.06 (s, 3H); **^{13}C NMR (100 MHz, $CDCl_3$)** δ 173.3, 167.1, 142.6, 135.8, 133.7, 119.2, 76.9, 72.5, 72.5, 49.1, 32.6, 31.7, 31.6, 31.6, 25.4, 25.3, 24.3, 23.8, 23.6, 21.9, 14.3; **HRMS (ESI) [M+Na]$^+$** Calcd for $C_{26}H_{42}O_5Na$: 457.29245 m/z, Found: 457.29225 m/z; **Specific rotation**: $[\alpha]_D^{24.9}$ +11.5 (*c* 1.00, $CHCl_3$)for an enantiomerically enriched sample of 95:5 e.r.

Enantiomeric purity of **5b** was determined by HPLC analysis in comparison with authentic racemic material (95:5 e.r. shown; Chiralpak IE column, 95:5 hexane/ *i*-PrOH, 0.5 mL/min, 262 nm).

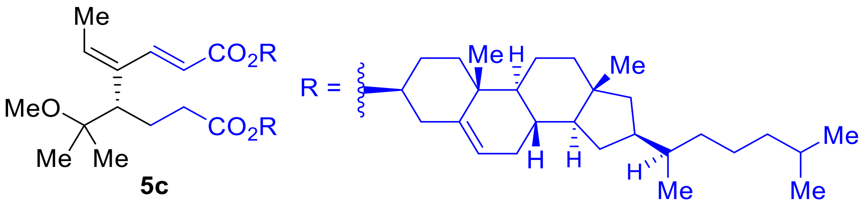

5c

Bis((3*S*,8*S*,9*S*,10*R*,13*R*,14*S*,17*R*)-10,13-dimethyl-17-((*R*)-6-methylheptan-2-yl)-2,3,4,7,8,9,10,11,12,13,14,15,16,17-tetradecahydro-1H-cyclopenta[a]phenanthren-3-yl) (*S*,2*E*,4*E*)-4-ethylidene-5-(2-methoxypropan-2-yl)oct-2-enedioate (5c)

Colorless oil, 71% yield, >95:5 dr. **IR (neat):** 2944 (m), 2868 (w), 2850 (w), 2360 (s), 2338 (m), 1730 (w), 1713 (w), 1622 (w), 1465 (w), 1377 (w), 1329 (w), 1270 (m), 1167 (m), 1139 (w), 1076 (w), 1013 (w), 982 (w), 948 (w), 866 (w), 800 (w), 762 (w), 679 (w), 646 (w), 625 (w) cm^{-1}; **^{1}H NMR (400 MHz, $CDCl_3$)** δ 7.78 (d, *J* = 15.6 Hz, 1H), 5.98 (q, *J* = 7.2 Hz, 1H), 5.91 (d, *J* = 15.8 Hz, 1H), 5.38 (d, *J* = 5.2 Hz, 2H), 4.68 (tt, *J* = 10.8, 5.2 Hz, 1H), 4.56 (tt, *J* = 11.2, 4.8 Hz,

1H), 3.18 (s, 3H), 2.55 (d, J = 11.6 Hz, 1H), 2.31 – 2.27 (m, 4H), 2.21 – 2.07 (m, 2H), 2.07 – 1.73 (m, 13H), 1.70 – 1.65 (m, 2H), 1.63 – 1.41 (m, 13H), 1.41 – 1.23 (m, 11H), 1.17 – 1.10 (m, 11H), 1.07 (d, J = 7.2 Hz, 7H), 1.01 (d, J = 11.2 Hz, 11H), 0.91 (dd, J = 6.4, 2.4 Hz, 7H), 0.86 (d, J = 6.4 Hz, 13H), 0.68 (s, 3H), 0.67 (s, 3H); **^{13}C NMR (100 MHz, $CDCl_3$)** δ 173.2, 167.1, 142.8, 139.7, 139.6, 135.8, 133.9, 122.7, 122.6, 118.0, 77.3, 77.0, 76.9, 76.7, 73.9, 73.8, 56.7, 56.6, 56.1, 50.0, 49.1, 42.3, 42.3, 39.7, 39.5, 38.2, 38.2, 37.0, 36.60, 36.57, 36.2, 35.8, 32.5, 31.90, 31.88, 31.86, 31.8, 28.2, 28.0, 27.8, 27.7, 24.3, 23.8, 23.6, 22.8, 22.5, 22.0, 21.0, 19.35, 19.30, 18.7, 14.3, 11.82, 11.80; **HRMS (ESI) [M+Na]$^+$** Calcd for $C_{68}H_{110}O_5Na$: 1029.82455 m/z, Found: 1029.82620 m/z; **Specific rotation**: $[\alpha]_D^{28.7}$ -17.4 (c 1.00, $CHCl_3$) for an enantiomerically enriched sample of >95:5 dr.

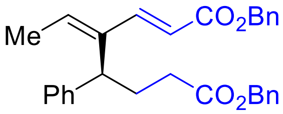

6a

Dibenzyl (*R*,2*E*,4*Z*)-4-ethylidene-5-phenyloct-2-enedioate (6a)

Pale yellow oil, 53% yield. **IR (neat):** 3062 (w), 3032 (w), 2925 (w), 2853 (w), 2359 (w), 1731 (m), 1620 (w), 1496 (w), 1453 (w), 1380 (w), 1302 (w), 1257 (w), 1211 (w), 1161 (m), 1080 (w), 983 (w), 910 (w), 866 (w), 824 (w), 743 (m), 698 (s), 654 (w) cm^{-1}; **^{1}H NMR (600 MHz, $CDCl_3$)** δ 7.39 – 7.33 (m, 10H), 7.33 – 7.28 (m, 3H), 7.25 – 7.20 (m, 3H), 6.21 (q, J = 7.2 Hz, 1H), 5.82 (d, J = 16.2 Hz, 1H), 5.18 – 5.12 (m, 4H), 4.10 (dd, J = 10.4, 5.6 Hz, 1H), 2.53 – 2.46 (m, 1H), 2.46 – 2.41 (m, 1H), 2.39 – 2.33 (m, 1H), 2.26 – 2.20 (m,1H), 1.83 (d, J = 7.2 Hz, 3H); **^{1}H NMR (600 MHz, Acetone-d_6)** δ 7.39 – 7.36 (m, 2H), 7.36 – 7.34 (m, 5H), 7.34 – 7.28 (m, 7H), 7.22 – 7.18 (m, 2H), 6.33 – 6.26 (m, 1H), 5.83 (d, J = 16.2 Hz, 1H), 5.16 – 5.02 (m, 4H), 4.20 (dd, J = 10.2, 5.4 Hz, 1H), 2.55 – 2.33 (m, 3H), 2.25 – 2.17 (m, 1H), 1.86 (d, J = 7.2 Hz, 3H); **^{13}C NMR (100 MHz, $CDCl_3$)** δ 173.0, 166.9, 146.8, 141.6, 138.8, 136.1, 135.8, 135.5, 128.53, 128. 47, 128.3, 128.24, 128.21, 128.1, 127.1, 126.3, 117.1, 66.3, 66.0, 41.3, 32.1, 26.0, 14.6; **HRMS (ESI) [M+Na]$^+$** Calcd for $C_{30}H_{30}O_4Na$: 477.20363 m/z, Found: 477.20322 m/z; **Specific rotation**: $[\alpha]_D^{30.7}$ +7.3 (c 1.00, $CHCl_3$) for an enantiomerically enriched sample of 97:3 e.r.

Enantiomeric purity of **6a** was determined by HPLC analysis in comparison with authentic racemic material (97:3 e.r. shown; Chiralpak IG column, 95:5 hexane/ *i*-PrOH, 1.0 mL/min, 254 nm).

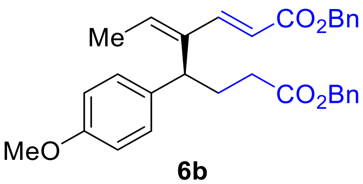

6b

Dibenzyl (*R*,2*E*,4*Z*)-4-ethylidene-5-(4-methoxyphenyl)oct-2-enedioate (6b)

Pale yellow oil, 40% yield. **IR (neat):** 3033 (w), 2959 (w), 2838 (w), 1731 (m), 1611 (w), 1583 (w), 1511 (m), 1455 (w), 1418 (w), 1380 (w), 1301 (w), 1250 (s), 1212 (w), 1161 (s), 1083 (w), 1032 (m), 985 (w), 908 (w), 803 (w), 750 (w), 698 (m), 652 (w), 635 (w) cm^{-1}; **^{1}H NMR (400 MHz, $CDCl_3$)** δ 7.38 – 7.29 (m, 10H), 7.21 (d, *J* = 16.0 Hz, 1H), 7.17 – 7.10 (m, 2H), 6.83 (d, *J* = 8.8 Hz, 2H), 6.22 – 6.13 (m, 1H), 5.79 (d, *J* = 16.0 Hz, 1H), 5.17 – 5.07 (m, 4H), 4.01 (dd, *J* = 10.4, 5.2 Hz, 1H), 3.78 (s, 3H), 2.52 – 2.26 (m, 3H), 2.26 – 2.18 (m, 1H), 1.80 (d, *J* = 7.2 Hz, 3H); **^{13}C NMR (100 MHz, $CDCl_3$)** δ 173.1, 167.0, 158.0, 146.8, 139.0, 136.1, 135.9, 135.1, 133.6, 128.5, 128.5, 128.3, 128.2, 128.1, 128.1, 117.1, 113.9, 66.3, 66.1, 55.2, 40.6, 32.2, 26.2, 14.6; **HRMS (ESI) $[M+Na]^+$** Calcd for $C_{31}H_{32}O_5Na$: 507.21420 m/z, Found: 507.21355 m/z; **Specific rotation**: $[\alpha]_D^{30.7}$ +8.7 (*c* 1.00, $CHCl_3$) for an enantiomerically enriched sample of 95.5:4.5 e.r.

Enantiomeric purity of **6b** was determined by HPLC analysis in comparison with authentic racemic material (95.5:4.5 e.r. shown; Chiralcel OD-H column, 93:7 hexane/ *i*-PrOH, 1.0 mL/min, 208 nm).

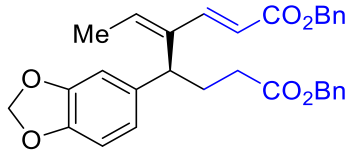

6c

Dibenzyl (*R*,2*E*,4*Z*)-5-(benzo[d][1,3]dioxol-5-yl)-4-ethylideneoct-2-enedioate (6c)

Pale yellow oil, 43% yield. **IR (neat):** 3031 (w), 2927 (w), 1731 (m), 1712 (m), 1620 (w), 1501 (m), 1488 (w), 1454 (w), 1439 (w), 1379 (w), 1303 (w), 1234 (m), 1162 (s), 1099 (w), 1038 (m), 983 (w), 932 (w), 865 (w), 814 (w), 749 (w), 698 (w), 633 (m), 617 (w) cm^{-1}; **^{1}H NMR (400 MHz, $CDCl_3$)** δ 7.41 – 7.29 (m, 10H), 7.20 (d, *J* = 16.0 Hz, 1H), 6.78 – 6.66 (m, 3H), 6.21 – 6.11 (m, 1H), 5.92 (s, 2H), 5.80 (d, *J* = 16.0 Hz, 1H), 5.18 – 5.07 (m, 4H), 3.98 (dd, *J* = 10.4, 4.8 Hz, 1H), 2.45 – 2.25 (m, 3H), 2.20 – 2.07 (m, 1H), 1.80 (d, *J* = 7.2 Hz, 3H); **^{13}C NMR (100 MHz, $CDCl_3$)** δ 173.0, 166.9, 147.8, 146.6, 146.0, 138.8, 136.1, 135.8, 135.5, 135.2, 128.5, 128.5, 128.3, 128.2, 128.1, 119.8, 117.2, 108.1, 107.8, 100.9, 66.3, 66.1, 41.0, 32.1, 26.2, 14.6; **HRMS (ESI)**

[M+Na]$^+$ Calcd for $C_{31}H_{30}O_6Na$: 521.19346 m/z, Found: 521.19316 m/z; **Specific rotation**: $[\alpha]_D^{27.4}$ -23.6 (*c* 1.00, $CHCl_3$) for an enantiomerically enriched sample of 94:6 e.r.

Enantiomeric purity of **6c** was determined by HPLC analysis in comparison with authentic racemic material (94:6 e.r. shown; Chiralpak IA column, 95:5 hexane/ *i*-PrOH, 1.0 mL/min, 254 nm).

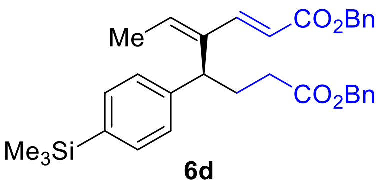

6d

Dibenzyl (*R*,2*E*,4*Z*)-4-ethylidene-5-(4-(trimethylsilyl)phenyl)oct-2-enedioate (6d)

Colorless oil, 40% yield. **IR (neat):** 3065 (w), 3034 (w), 2955 (w), 1734 (m), 1714 (m), 1621 (w), 1498 (w), 1454 (w), 1381 (w), 1302 (w), 1248 (m), 1212 (w), 1161 (s), 1109 (w), 1083 (w), 1016 (w), 983 (w), 908 (w), 839 (s), 751 (m), 697 (m), 634 (w) cm^{-1}; **^{1}H NMR (400 MHz, $CDCl_3$)** δ 7.55 – 7.48 (m, 2H), 7.44 – 7.40 (m, 10H), 7.33 (d, *J* = 1.6 Hz, 1H), 7.32 – 7.26 (m, 2H), 6.27 (q, *J* = 7.2 Hz, 1H), 5.89 (d, *J* = 16.0 Hz, 1H), 5.28 – 5.14 (m, 4H), 4.13 (dd, *J* = 10.4, 5.2 Hz, 1H), 2.60 – 2.48 (m, 1H), 2.48 – 2.35 (m, 2H), 2.35 – 2.22 (m, 1H), 1.88 (d, *J* = 7.2 Hz, 3H), 0.33 (s, 9H); **^{13}C NMR (100 MHz, $CDCl_3$)** δ 173.0, 166.9, 146.7, 142.2, 138.7, 138.2, 136.1, 135.8, 135.4, 133.6, 128.55, 128.48, 128.30, 128.25, 128.2, 128.1, 126.5, 117.2, 66.3, 66.1, 41.4, 32.1, 26.0, 14.7, -1.1; **HRMS (ESI) [M+Na]**$^+$ Calcd for $C_{33}H_{38}O_4NaSi$: 549.24316 m/z, Found: 549.24236 m/z; **Specific rotation**: $[\alpha]_D^{30.7}$ -9.9 (*c* 1.00, $CHCl_3$) for an enantiomerically enriched sample of 96:4 e.r.

Enantiomeric purity of **6d** was determined by HPLC analysis in comparison with authentic racemic material (96:4 e.r. shown; Chiralpak IA column, 95:5 hexane/ *i*-PrOH, 0.5 mL/min, 254 nm).

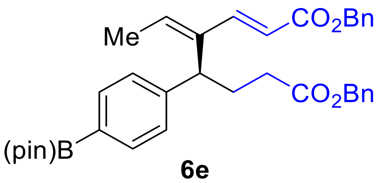

6e

Dibenzyl (R,2E,4Z)-4-ethylidene-5-(4-(4,4,5,5-tetramethyl-1,3,2-dioxaborolan-2-yl)phenyl)oct-2-enedioate (6e)

Colorless oil, 40% yield. **IR (neat):** 3036 (w), 2962 (w), 2923 (w), 2851 (w), 1733 (m), 1714 (m), 1610 (m), 1514 (w), 1498 (w), 1455 (w), 1399 (w), 1360 (s), 1320 (w), 1304 (w), 1261 (m), 1213 (w), 1162 (s), 1144 (s), 1090 (s), 1019 (m), 984 (w), 963 (w), 908 (w), 859 (w), 821 (w), 800 (m), 740 (w), 697 (m), 677 (m), 659 (w), 636 (w) cm^{-1}; **^{1}H NMR (400 MHz, $CDCl_3$)** δ 7.81 (d, *J* = 8.0 Hz, 2H), 7.47 – 7.35 (m, 10H), 7.34 – 7.28 (m, 3H), 6.25 (q, *J* = 7.2 Hz, 1H), 5.84 (d, *J* = 16.0 Hz, 1H), 5.25 – 5.14 (m, 4H), 4.15 (dd, *J* = 10.4, 5.2 Hz, 1H), 2.60 – 2.49 (m, 1H), 2.49 – 2.35 (m, 2H), 2.33 – 2.23 (m, 1H), 1.86 (d, *J* = 7.2 Hz, 3H), 1.41 (s, 12H); **^{13}C NMR (100 MHz, $CDCl_3$)** δ 173.0, 166.9, 146.8, 145.0, 138.7, 136.1, 135.9, 135.0, 128.6, 128.5, 128.3, 128.3, 128.2, 128.1, 126.6, 117.2, 83.7, 66.3, 66.1, 41.5, 32.1, 25.9, 24.9, 24.8, 14.7; **HRMS (ESI) $[M+Na]^+$** Calcd for $C_{36}H_{41}BO_6Na$: 603.28884 m/z, Found: 603.28884 m/z; **Specific rotation**: $[\alpha]_D^{30.9}$ -19.6 (*c* 1.00, $CHCl_3$) for an enantiomerically enriched sample of 98:2 e.r.

Enantiomeric purity of **6e** was determined by HPLC analysis in comparison with authentic racemic material (98:2 e.r. shown; Chiralcel OD-H column, 95:5 hexane/ *i*-PrOH, 0.8 mL/min, 254 nm).

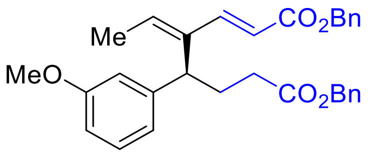

6f

Dibenzyl (*R*,2*E*,4*Z*)-4-ethylidene-5-(3-methoxyphenyl)oct-2-enedioate (6f)

Pale yellow oil, 54% yield. **IR (neat):** 3032 (w), 2942 (w), 2835 (w), 1732 (m), 1712 (m), 1605 (w), 1583 (w), 1491 (w), 1454 (w), 1435 (w), 1380 (w), 1259 (m), 1161 (s), 1082 (w), 1044 (w), 983 (w), 910 (w), 870 (w), 826 (w), 788 (w), 750 (m), 697 (m), 659 (w), 612 (w) cm^{-1}; **^{1}H NMR (400 MHz, $CDCl_3$)** δ 7.39 – 7.26 (m, 10H), 7.24 – 7.18 (m, 1H), 7.22 – 7.16 (m, 1H), 6.83 (d, *J* = 7.6 Hz, 1H), 6.77 – 6.70 (m, 2H), 6.17 (q, *J* = 7.2 Hz, 1H), 5.79 (d, *J* = 16.0 Hz, 1H), 5.18 – 5.01 (m, 4H), 4.03 (dd, *J* = 10.4, 5.2 Hz, 1H), 3.76 (s, 3H), 2.52 – 2.39 (m, 1H), 2.39 – 2.23 (m, 2H), 2.23 – 2.08 (m, 1H), 1.79 (d, *J* = 7.2 Hz, 3H); **^{13}C NMR (100 MHz, $CDCl_3$)** δ 173.0, 166.9, 159.7, 146.7, 143.3, 138.7, 136.0, 135.8, 135.6, 129.4, 128.53, 128.46 128.3, 128.24, 128.22, 128.1, 119.4, 117.1, 113.5, 111.2, 66.3, 66.0, 55.1, 41.2, 32.1, 25.9, 14.7; **HRMS (ESI) $[M+Na]^+$** Calcd for $C_{31}H_{32}O_5Na$: 507.21420 m/z, Found: 507.21333 m/z; **Specific rotation**: $[\alpha]_D^{30.8}$ -30.6 (*c* 1.00, $CHCl_3$) for an enantiomerically enriched sample of 97:3 e.r.

Enantiomeric purity of **6f** was determined by HPLC analysis in comparison with authentic racemic material (97:3 e.r. shown; Chiralpak IA column, 95:5 hexane/ *i*-PrOH, 1.0 mL/min, 254 nm).

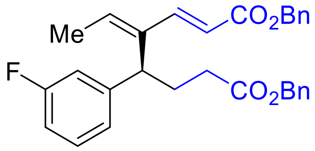

6g

Dibenzyl (*R*,2*E*,4*Z*)-4-ethylidene-5-(3-fluorophenyl)oct-2-enedioate (6g)

Pale yellow oil, 40% yield. **IR (neat):** 3032 (w), 2925 (w), 2854 (w), 1732 (m), 1713 (m), 1615 (w), 1587 (w), 1488 (w), 1453 (w), 1380 (w), 1304 (w), 1246 (m), 1162 (s), 1080 (w), 982 (w), 909 (w), 870 (w), 828 (w), 786 (w), 749 (m), 697 (m), 620 (w) cm^{-1}; **^{1}H NMR (400 MHz, $CDCl_3$)** δ 7.43 – 7.32 (m, 10H), 7.32 – 7.27 (m, 1H), 7.23 (d, *J* = 16.0 Hz, 1H), 7.07 – 7.04 (m, 1H), 6.98 – 6.89 (m, 2H), 6.30 – 6.20 (m, 1H), 5.79 (d, *J* = 16.0 Hz, 1H), 5.20 – 5.12 (m, 4H), 4.10 (dd, *J* = 10.4, 5.2 Hz, 1H), 2.54 – 2.39 (m, 2H), 2.39 – 2.30 (m, 1H), 2.27 – 2.15 (m, 1H), 1.82 (d, *J* = 7.2 Hz, 3H); **^{13}C NMR (100 MHz, $CDCl_3$)** δ 172.8, 166.8, 163.0 (d, *J* = 242.4 Hz), 146.4, 144.4 (d, *J* = 6.7 Hz), 138.2, 136.3, 136.0, 135.8, 129.9 (d, *J* = 8.2 Hz), 128.6, 128.5, 128.32, 128.29, 128.25, 128.1, 122.6 (d, *J* = 2.7 Hz), 117.3, 114.2 (d, *J* = 21.8 Hz), 113.3 (d, *J* = 21.1 Hz), 66.3, 66.1, 40.9, 40.9, 31.9, 25.7, 14.7; **^{19}F NMR (376 MHz, $CDCl_3$)** δ -112.9; **HRMS (ESI) $[M+Na]^+$** Calcd for $C_{30}H_{29}O_4FNa$: 495.19421 m/z, Found: 495.19322 m/z; **Specific rotation**: $[\alpha]_D^{30.7}$ -34.0 (*c* 1.00, $CHCl_3$) for an enantiomerically enriched sample of 98:2 e.r.

Enantiomeric purity of **6g** was determined by HPLC analysis in comparison with authentic racemic material (98:2 e.r. shown; Chiralcel OD-H column, 95:5 hexane/ *i*-PrOH, 1.0 mL/min, 208 nm).

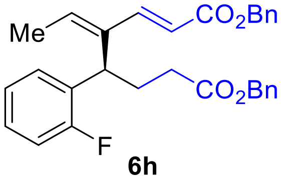

6h

Dibenzyl (*R*,2*E*,4*Z*)-4-ethylidene-5-(2-fluorophenyl)oct-2-enedioate (6h)

Pale yellow oil, 52% yield. **IR (neat):** 3034 (w), 2955 (w), 1732 (m), 1713 (m), 1619 (w), 1584 (w), 1489 (w), 1454 (w), 1380 (w), 1301 (w), 1253 (w), 1228 (w), 1162 (s), 1105 (w), 982 (w), 909 (w), 824 (w), 803 (w), 754 (s), 697 (s), 626 (w) cm^{-1}; **^{1}H NMR (400 MHz, $CDCl_3$)** δ 7.40

– 7.29 (m, 11H), 7.27 – 7.20 (m, 1H), 7.20 – 7.16 (m, 1H), 7.12 – 7.08 (m, 1H), 7.00 – 6.95 (m, 1H), 6.12 (q, *J* = 7.2 Hz, 1H), 5.95 (d, *J* = 15.6 Hz, 1H), 5.13 (s, 2H), 5.10 (s, 2H), 4.23 (dd, *J* = 9.6, 6.0 Hz, 1H), 2.47 – 2.33 (m, 2H), 2.33 – 2.18 (m, 2H), 1.81 (d, *J* = 7.2 Hz, 3H); **^{13}C NMR (100 MHz, CDCl$_3$)** δ 172.9, 166.8, 161.0 (d, *J* = 246.3 Hz), 146.3, 137.0, 136.1, 135.8, 134.0, 128.8, 128.6, 128.5 (d, *J* = 6.3 Hz), 128.28, 128.26, 128.24, 128.21, 128.15, 128.08, 124.0 (d, *J* = 3.5 Hz), 117.0, 115.4 (d, *J* = 22.6 Hz), 66.3, 66.0, 36.0, 36.0, 31.9, 26.3, 14.4; **^{19}F NMR (376 MHz, CDCl$_3$)** δ -115.35; **HRMS (ESI) [M+Na]$^+$** Calcd for $C_{30}H_{29}O_4FNa$: 495.19421 m/z, Found: 495.19414 m/z; **Specific rotation**: $[\alpha]_D^{30.7}$ -28.1 (*c* 1.00, CHCl$_3$) for an enantiomerically enriched sample of >99.5:0.5 e.r.

Enantiomeric purity of **6h** was determined by HPLC analysis in comparison with authentic racemic material (>99.5:0.5 e.r. shown; Chiralcel OD-H column, 95:5 hexane/ *i*-PrOH, 1.0 mL/min, 254 nm).

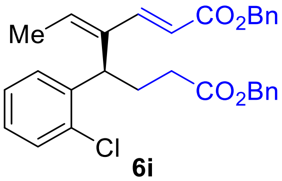

6i

Dibenzyl (*R*,2*E*,4*Z*)-5-(2-chlorophenyl)-4-ethylideneoct-2-enedioate (6i)

Pale yellow oil, 51% yield. **IR (neat):** 3690 (w), 3674 (w), 3647 (w), 3627 (w), 3566 (w), 3066 (w), 3035 (w), 2925 (w), 2851 (w), 2360 (m), 2339 (w), 1731 (m), 1651 (w), 1557 (w), 1540 (w), 1520 (w), 1497 (w), 1473 (w), 1455 (w), 1418 (w), 1381 (w), 1261 (w), 1211 (m), 1158 (w), 1035 (w), 1002 (w), 982 (w), 906 (w), 831 (w), 750 (m), 697 (m), 668 (w), 636 (w) cm^{-1}; **^{1}H NMR (400 MHz, CDCl$_3$)** δ 7.41 – 7.31 (m, 12H), 7.26 – 7.22 (m, 1H), 7.21 – 7.14 (m, 2H), 6.16 (q, *J* = 7.6 Hz, 1H), 5.98 (d, *J* = 15.6 Hz, 1H), 5.14 (s, 2H), 5.11 (s, 2H), 4.28 (dd, *J* = 9.2, 6.4 Hz, 1H), 2.43 – 2.39 (m, 2H), 2.36 – 2.11 (m, 2H), 1.83 (d, *J* = 7.2 Hz, 3H); **^{13}C NMR (101 MHz, CDCl$_3$)** δ 172.9, 166.8, 146.3, 139.0, 136.5, 136.1, 135.8, 134.5, 133.3, 129.9, 128.6, 128.5, 128.33, 128.26, 128.13, 128.10, 127.9, 126.9, 117.1, 66.4, 66.0, 40.1, 32.0, 27.5, 14.8; **HRMS (ESI) [M+Na]$^+$** Calcd for $C_{30}H_{29}O_4NaCl$: 511.16466 m/z, Found: 511.16397 m/z; **Specific rotation**: $[\alpha]_D^{27.6}$ +35.0 (*c* 1.00, CHCl$_3$) for an enantiomerically enriched sample of 98:2 e.r.

Enantiomeric purity of **6i** was determined by HPLC analysis in comparison with authentic racemic material (98:2 e.r. shown; Chiralpak IC column, 93:7 hexane/ *i*-PrOH, 1.0 mL/min, 208 nm).

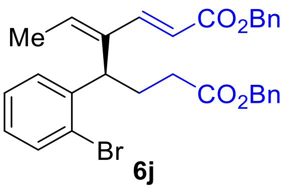

6j

Dibenzyl (*R*,2*E*,4*Z*)-5-(2-bromophenyl)-4-ethylideneoct-2-enedioate (6j)

Pale yellow oil, 49% yield. **IR (neat):** 3065 (w), 3033 (w), 2947 (w), 1732 (m), 1618 (w), 1497 (w), 1454 (w), 1380 (w), 1302 (w), 1249 (w), 1211 (w), 1162 (s), 1081 (w), 1022 (w), 981v (w), 909 (w), 822 (w), 750 (s), 697 (s), 677 (w) cm^{-1}; **^{1}H NMR (400 MHz, $CDCl_3$)** δ 7.54 (dd, *J* = 8.0, 1.2 Hz, 1H), 7.38 – 7.28 (m, 12H), 7.22 (s, 1H), 7.11 – 7.07 (m, 1H), 6.16 (q, *J* = 7.2 Hz, 1H), 6.00 (d, *J* = 15.6 Hz, 1H), 5.15 (s, 2H), 5.12 (s, 2H), 4.24 (dd, *J* = 8.8, 6.8 Hz, 1H), 2.43 – 2.39 (m, 2H), 2.35 – 2.25 (m, 1H), 2.25 – 2.14 (m, 1H), 1.85 (d, *J* = 7.2 Hz, 3H); **^{13}C NMR (100 MHz, $CDCl_3$)** δ 172.8, 166.8, 146.3, 140.6, 136.5, 136.1, 135.8, 133.3, 133.0, 128.8, 128.54, 128.48, 128.3, 128.25, 128.17, 128.10, 128.08, 127.5, 125.1, 117.3, 66.4, 66.0, 42.7, 32.0, 27.9, 15.1; **HRMS (ESI) $[M+Na]^+$** Calcd for $C_{30}H_{29}O_4NaBr$: 555.11414 m/z, Found: 555.11324 m/z; **Specific rotation**: $[\alpha]_D^{30.8}$ -1.8 (*c* 1.00, $CHCl_3$) for an enantiomerically enriched sample of >99.5:0.5 e.r.

Enantiomeric purity of **6j** was determined by HPLC analysis in comparison with authentic racemic material (>99.5:0.5 e.r. shown; Chiralcel OD-H column, 95:5 hexane/ *i*-PrOH, 1.0 mL/min, 208 nm).

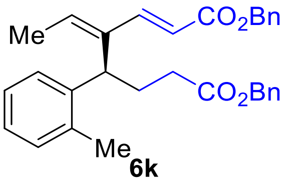

6k

Dibenzyl (*R*,2*E*,4*Z*)-4-ethylidene-5-(o-tolyl)oct-2-enedioate (6k)

Pale yellow oil, 64% yield. **IR (neat):** 3031 (w), 2951 (w), 1731 (m), 1713 (m), 1618 (w), 1495 (w), 1455 (w), 1379 (w), 1303 (w), 1252 (w), 1213 (w), 1161 (s), 1114 (w), 1088 (w), 983 (w), 908 (w), 823 (w), 747 (m), 697 (m), 657 (w) cm^{-1}; **^{1}H NMR (400 MHz, $CDCl_3$)** δ 7.36 – 7.27 (m, 11H), 7.21 (d, *J* = 7.6 Hz, 1H), 7.19 – 7.11 (m, 3H), 6.12 (q, *J* = 7.2 Hz, 1H), 5.98 (d, *J* = 15.6 Hz, 1H), 5.14 (s, 2H), 5.12 (s, 2H), 4.03 (dd, *J* = 8.8, 6.4 Hz, 1H), 2.43 (t, *J* = 7.2 Hz, 2H), 2.38 – 2.31 (m, 1H), 2.23 – 2.16 (m, 4H), 1.79 (d, *J* = 7.2 Hz, 3H); **^{13}C NMR (100 MHz, $CDCl_3$)** δ 173.1, 166.8, 146.7, 139.2, 137.5, 136.9, 136.1, 135.8, 132.4, 130.7, 128.5, 128.5, 128.3, 128.2, 128.1,

128.0, 126.8, 126.5, 126.0, 116.8, 66.3, 66.0, 39.6, 32.0, 27.4, 19.7, 14.6; **HRMS (ESI) [M+Na]$^+$** Calcd for $C_{31}H_{32}O_4Na$: 491.21928 m/z, Found: 491.21830 m/z; **Specific rotation**: $[\alpha]_D^{30.3}$ -73.8 (*c* 1.00, $CHCl_3$) for an enantiomerically enriched sample of >99.5:0.5 e.r.

Enantiomeric purity of **6k** was determined by HPLC analysis in comparison with authentic racemic material (>99.5:0.5 e.r. shown; Chiralcel OD-H column, 95:5 hexane/ *i*-PrOH, 1.0 mL/min, 208 nm).

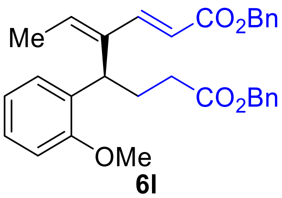

6l

Dibenzyl (*R*,2*E*,4*Z*)-4-ethylidene-5-(2-methoxyphenyl)oct-2-enedioate (6l)

Pale yellow oil, 61% yield. **IR (neat):** 3032 (w), 2938 (w), 2836 (w), 1732 (m), 1617 (w), 1491 (w), 1457 (w), 1380 (w), 1292 (w), 1244 (s), 1160 (w), 1116 (s), 1052 (w), 1027 (w), 984 (w), 908 (w), 864 (w), 800 (w), 752 (s), 698 (m), 640 (w), 621 (w) cm^{-1}; **^{1}H NMR (400 MHz, $CDCl_3$)** δ 7.34 – 7.27 (m, 11H), 7.23 – 7.22 (m, 1H), 7.20 – 7.15 (m, 1H), 6.94 – 6.90 (m, 1H), 6.80 (dd, *J* = 8.0, 1.2 Hz, 1H), 6.11 – 6.03 (m, 1H), 5.96 (d, *J* = 15.6 Hz, 1H), 5.12 (s, 2H), 5.08 (d, *J* = 1.0 Hz, 2H), 4.23 (dd, *J* = 9.2, 6.4 Hz, 1H), 3.72 (s, 3H), 2.38 – 2.33 (m, 2H), 2.31 – 2.14 (m, 2H), 1.81 (d, *J* = 7.2 Hz, 3H); **^{13}C NMR (100 MHz, $CDCl_3$)** δ 173.2, 167.1, 157.3, 147.3, 137.9, 136.2, 135.9, 133.3, 130.1, 128.51, 128.46, 128.23, 128.17, 128.1, 128.0, 127.7, 127.5, 120.4, 116.4, 110.4, 66.2, 65.9, 55.2, 36.6, 32.3, 26.9, 14.6; **HRMS (ESI) [M+Na]$^+$** Calcd for $C_{31}H_{32}O_5Na$: 507.21420 m/z, Found: 507.21371 m/z; **Specific rotation**: $[\alpha]_D^{30.7}$ +5.3 (*c* 1.00, $CHCl_3$) for an enantiomerically enriched sample of >99.5:0.5 e.r.

Enantiomeric purity of **6l** was determined by HPLC analysis in comparison with authentic racemic material (>99.5:0.5 e.r. shown; Chiralpak IC column, 95:5 hexane/ *i*-PrOH, 1.0 mL/min, 254 nm).

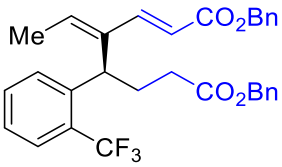

6m

Dibenzyl (*R*,2*E*,4*Z*)-4-ethylidene-5-(2-(trifluoromethyl)phenyl)oct-2-enedioate (6m)

Pale yellow oil, 58% yield. **IR (neat):** 3034 (w), 2938 (w), 1734 (m), 1714 (m), 1610 (w), 1495 (w), 1453 (w), 1379 (w), 1350 (w), 1310 (s), 1257 (w), 1212 (w), 1160 (s), 1120 (s), 1064 (w),1036 (w), 981 (w), 909 (w), 802 (w), 769 (w), 750 (w), 697 (m), 656 (w) cm^{-1}; **^{1}H NMR (400 MHz, $CDCl_3$)** δ 7.65 (d, J = 7.6 Hz, 1H), 7.55 – 7.49 (m, 2H), 7.37 – 7.30 (m, 12H), 6.08 (q, J = 7.2 Hz, 1H), 6.00 (d, J = 15.6 Hz, 1H), 5.15 (d, J = 2.0 Hz, 2H), 5.10 (s, 2H), 4.43 (t, J = 7.8 Hz, 1H), 2.37 – 2.33 (m, 2H), 2.29 – 2.14 (m, 2H), 1.81 (d, J = 7.2 Hz, 3H); **^{13}C NMR (100 MHz, $CDCl_3$)** δ 172.7, 166.6, 145.8, 140.1, 137.4, 136.1, 135.7, 131.9, 130.3, 129.2, 129.0 (q, J = 30.3 Hz), 128.54, 128.50, 128.4, 128.3, 128.12, 128.08, 126.8, 126.6 (q, J = 6.2 Hz), 124.4 (q, J = 274.2 Hz), 118.1, 66.4, 66.0, 39.0, 32.0, 29.1, 14.3; **^{19}F NMR (376 MHz, CDCl3)** δ -58.97; **HRMS (ESI) [M+Na]$^+$** Calcd for $C_{31}H_{29}O_4F_3Na$: 545.19102 m/z, Found: 545.19031 m/z; **Specific rotation**: $[\alpha]_D^{30.9}$ -13.7 (*c* 1.00, $CHCl_3$) for an enantiomerically enriched sample of 98:2 e.r.

Enantiomeric purity of **6m** was determined by HPLC analysis in comparison with authentic racemic material (98:2 e.r. shown; Chiralcel OZ-H column, 95:5 hexane/ *i*-PrOH, 1.0 mL/min, 254 nm).

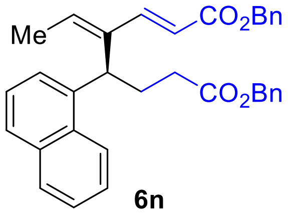

6n

Dibenzyl (*R*,2*E*,4*Z*)-4-ethylidene-5-(naphthalen-1-yl)oct-2-enedioate (6n)

Pale yellow oil, 47% yield. **IR (neat):** 3032 (w), 2929 (w), 2362 (w), 1730 (m), 1712 (m), 1617 (w), 1497 (w), 1454 (w), 1379 (w), 1354 (w), 1302 (w), 1255 (w), 1211 (w), 1162 (s), 1090 (w), 982 (w), 909 (w), 862 (w), 825 (w), 797 (w), 780 (m), 738 (m), 697 (w), 650 (w) cm^{-1}; **^{1}H NMR (400 MHz, $CDCl_3$)** δ 7.89 – 7.80 (m, 2H), 7.74 (d, J = 8.0 Hz, 1H), 7.52 (d, J = 7.2 Hz, 1H), 7.47 – 7.43 (m, 3H), 7.37 – 7.29 (m, 10H), 7.23 (d, J = 7.2 Hz, 1H), 6.20 – 6.11 (m, 1H), 6.00 (d, J = 15.6 Hz, 1H), 5.12 (s, 2H), 5.11 (s, 2H), 4.63 (dd, J = 8.8, 5.6 Hz, 1H), 2.53 – 2.49 (m, 3H), 2.38 – 2.25 (m, 1H), 1.83 (d, J = 7.2 Hz, 3H); **^{13}C NMR (100 MHz, $CDCl_3$)** δ 173.1, 166.9, 147.1, 138.7, 137.3, 136.1, 135.8, 134.0, 133.5, 132.1, 128.9, 128.5, 128.5, 128.3, 128.2, 128.1, 128.1, 127.5, 126.0, 125.5, 125.2, 124.5, 123.3, 116.7, 66.3, 66.0, 39.0, 32.1, 27.6, 14.9; **HRMS (ESI) [M+Na]$^+$** Calcd for $C_{34}H_{32}O_4Na$: 527.21928 m/z, Found: 527.21829 m/z; **Specific rotation**: $[\alpha]_D^{26.7}$ -43.0 (*c* 1.00, $CHCl_3$) for an enantiomerically enriched sample of >99.5:0.5 e.r.

Enantiomeric purity of **6n** was determined by HPLC analysis in comparison with authentic racemic material (>99.5:0.5 e.r. shown; Chiralcel OD-H column, 95:5 hexane/ *i*-PrOH, 1.0 mL/min, 208 nm).

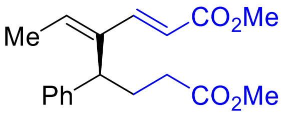

7a

Dimethyl (*R*,2*E*,4*Z*)-4-ethylidene-5-phenyloct-2-enedioate (7a)

Pale yellow oil, 44% yield. **IR (neat):** 2953 (w), 1733 (s), 1655 (w), 1600 (w), 1494 (w), 1437 (m), 1371 (w), 1308 (m), 1268 (m), 1200 (m), 1168 (m), 1020 (w), 986 (w), 901 (w), 863 (w), 762 (w), 730 (w), 702 (m), 639 (w), 616 (w) cm^{-1}; **^{1}H NMR (400 MHz, $CDCl_3$)** δ 7.37 – 7.27 (m, 4H), 7.25 – 7.16 (m, 2H), 6.23 (q, *J* = 7.2 Hz, 1H), 5.76 (d, *J* = 16.0 Hz, 1H), 4.11 (dd, *J* = 10.4, 5.6 Hz, 1H), 3.70 (d, *J* = 0.8 Hz, 6H), 2.56 – 2.40 (m, 1H), 2.40 – 2.31 (m, 2H), 2.29 – 2.16 (m, 1H), 1.89 (d, *J* = 7.2 Hz, 3H); **^{13}C NMR (100 MHz, $CDCl_3$)** δ 173.7, 167.5, 146.3, 141.7, 138.8, 135.4, 128.5, 127.1, 126.3, 117.2, 51.6, 51.4, 41.2, 31.9, 25.9, 14.7; **HRMS (ESI) $[M+Na]^+$** Calcd for $C_{18}H_{22}O_4Na$: 325.14103 m/z, Found: 325.14021 m/z; **Specific rotation**: $[\alpha]_D^{27.9}$ +25.1 (*c* 1.00, $CHCl_3$) for an enantiomerically enriched sample of 96:4 e.r.

Enantiomeric purity of **7a** was determined by HPLC analysis in comparison with authentic racemic material (96:4 e.r. shown; Chiralpak IC column, 95:5 hexane/ *i*-PrOH, 1.0 mL/min, 254 nm).

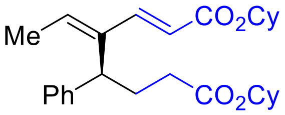

7b

Dicyclohexyl (*R*,2*E*,4*Z*)-4-ethylidene-5-phenyloct-2-enedioate (7b)

Pale yellow oil, 44% yield. **IR (neat):** 2935 (m), 2858 (m), 1725 (s), 1653 (w), 1494 (w), 1451 (m), 1381 (w), 1359 (w), 1258 (w), 1176 (m), 1122 (w), 1037 (m), 1014 (w), 982 (w), 910 (w), 893 (w), 799 (w), 754 (w), 727 (w), 701 (m), 633 (w) cm^{-1}; **^{1}H NMR (400 MHz, $CDCl_3$)** δ 7.24 – 7.18 (m, 4H), 7.17 – 7.06 (m, 2H), 6.13 (q, *J* = 7.2 Hz, 1H), 5.70 (d, *J* = 15.6 Hz, 1H), 4.76 – 4.63 (m, 2H), 4.01 (dd, *J* = 10.4, 5.6 Hz, 1H), 2.43 – 2.29 (m, 1H), 2.29 – 2.19 (m, 1H), 2.19 – 2.06 (m, 1H), 1.83 – 1.73 (m, 7H), 1.69 – 1.61 (m, 4H), 1.51 – 1.45 (m, 2H), 1.38 – 1.25 (m, 8H), 1.23 – 1.09 (m, 3H); **^{13}C NMR (100 MHz, $CDCl_3$)** δ 172.8, 166.6, 146.0, 141.9, 139.0, 134.7,

128.4, 127.2, 126.2, 118.1, 72.7, 72.4, 41.5, 32.5, 31.7, 26.2, 25.4, 25.3, 23.8, 14.7; **HRMS (ESI) $[M+Na]^+$** Calcd for $C_{28}H_{38}O_4Na$: 461.26623 m/z, Found: 461.26540 m/z; **Specific rotation**: $[\alpha]_D^{30.8}$ +26.5 (*c* 1.00, $CHCl_3$) for an enantiomerically enriched sample of 97:3 e.r.

Enantiomeric purity of **7b** was determined by HPLC analysis in comparison with authentic racemic material (97:3 e.r. shown; Chiralpak IC column, 95:5 hexane/ *i*-PrOH, 0.8 mL/min, 254 nm).

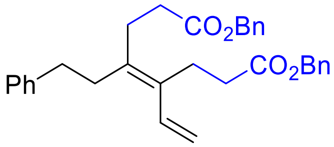

8a

Dibenzyl (*E*)-4-phenethyl-5-vinyloct-4-enedioate (8a)

Colorless oil, 79% yield. **IR (neat):** 3851 (w), 3030 (w), 2944 (w), 2361 (w), 1733 (s), 1625 (w), 1602 (w), 1496 (w), 1453 (w), 1417 (w), 1380 (w), 1353 (w), 1259 (w), 1231 (w), 1161 (m), 1080 (w), 1059 (w), 1029 (w), 989 (w), 904 (w), 747 (m), 697 (s), 653 (w), 613 (w) cm^{-1}; **^{1}H NMR (400 MHz, $CDCl_3$)** δ 7.40 – 7.33 (m, 10H), 7.31 – 7.26 (m, 2H), 7.24 – 7.14 (m, 3H), 6.60 (dd, *J* = 17.2, 11.2 Hz, 1H), 5.22 (d, *J* = 17.2 Hz, 1H), 5.15 (d, *J* = 2.8 Hz, 4H), 5.08 (d, *J* = 11.2 Hz, 1H), 2.73 – 2.55 (m, 4H), 2.52 – 2.42 (m, 8H); **^{13}C NMR (100 MHz, $CDCl_3$)** δ 173.0, 172.7, 141.5, 137.9, 135.9, 135.8, 133.1, 132.0, 128.5, 128.32, 128.27, 128.23, 128.18, 125.9, 113.2, 66.3, 66.2, 35.4, 33.7, 33.5, 33.2, 28.3, 22.8; **HRMS (ESI) $[M+Na]^+$** Calcd for $C_{32}H_{34}O_4Na$: 505.23493 m/z, Found: 505.23439 m/z.

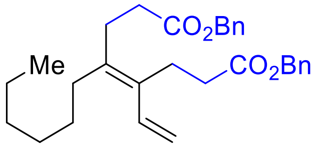

8b

Dibenzyl (*E*)-4-hexyl-5-vinyloct-4-enedioate (8b)

Colorless oil, 87% yield. **IR (neat):** 3663 (w), 2956 (m), 2929 (m), 1734 (s), 1497 (w), 1455 (w), 1415 (w), 1380 (w), 1354 (w), 1260 (w), 1230 (w), 1159 (s), 1103 (w), 1052 (w), 1001 (w), 904 (w), 737 (m), 697 (s), 660 (w), 628 (w) cm^{-1}; **^{1}H NMR (400 MHz, $CDCl_3$)** δ 7.37 – 7.35 (m, 10H), 6.62 (dd, *J* = 17.2, 11.2 Hz, 1H), 5.19 (dd, *J* = 17.6, 1.2 Hz, 1H), 5.12 (d, *J* = 2.4 Hz, 4H), 5.06 (dd, *J* = 11.2, 1.2 Hz, 1H), 2.64 – 2.60 (m, 2H), 2.53 – 2.37 (m, 6H), 2.14 – 2.10 (m, 2H), 1.40 – 1.24 (m, 8H), 0.95 – 0.83 (m, 3H); **^{13}C NMR (100 MHz, $CDCl_3$)** δ 173.1, 172.8, 139.3,

136.0, 135.9, 133.5, 131.2, 128.52, 128.49, 128.22, 128.18, 128.13, 128.11, 112.6, 66.3, 66.2, 33.8, 33.3, 31.7, 31.5, 29.4, 29.3, 28.3, 22.8, 22.6, 14.0; **HRMS (ESI) [M+Na]⁺** Calcd for $C_{30}H_{38}O_4Na$: 485.26623 m/z, Found: 485.26574 m/z.

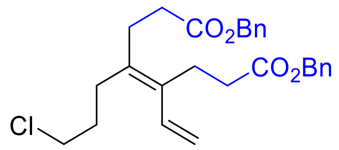

8c

Dibenzyl (*Z*)-4-(3-chloropropyl)-5-vinyloct-4-enedioate (8c)

Colorless oil, 90% yield. **IR (neat):** 3735 (w), 3586 (w), 3033 (w), 2955 (w), 2871 (w), 2362 (w), 1732 (s), 1626 (w), 1497 (w), 1454 (w), 1381 (w), 1353 (w), 1262 (w), 1233 (w), 1212 (w), 1162 (w), 1080 (s), 990 (w), 907 (w), 846 (w), 739 (m), 698 (s), 648 (w), 632 (w), 612 (w) cm^{-1}; **^{1}H NMR (400 MHz, $CDCl_3$)** δ 7.37 – 7.34 (m, 10H), 6.62 (dd, *J* = 17.6, 11.2 Hz, 1H), 5.22 (dd, *J* = 17.6, 1.2 Hz, 1H), 5.14 – 5.08 (m, 5H), 3.49 (t, *J* = 6.4 Hz, 2H), 2.62 (dd, *J* = 9.6, 6.4 Hz, 2H), 2.51 – 2.40 (m, 6H), 2.31 – 2.26 (m, 2H), 1.86 – 1.77 (m, 2H); **^{13}C NMR (100 MHz, $CDCl_3$)** δ 172.9, 172.6, 137.1, 135.9, 135.8, 133.1, 132.6, 128.53, 128.50, 128.3, 128.22, 128.18, 128.16, 113.6, 66.4, 66.2, 44.6, 33.7, 33.2, 31.9, 28.6, 28.2, 22.9; **HRMS (ESI) [M+Na]⁺** Calcd for $C_{27}H_{31}O_4$ NaCl: 477.18031 m/z, Found: 477.18022 m/z.

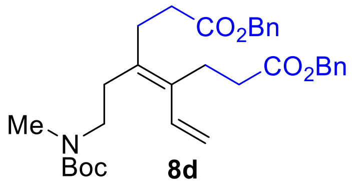

8d

Dibenzyl (*Z*)-4-(2-((tert-butoxycarbonyl)(methyl)amino)ethyl)-5-vinyloct-4-enedioate (8d)

Colorless oil, 86% yield. **IR (neat):** 3862 (w), 3629 (w), 2977 (w), 2360 (w), 1734 (m), 1692 (m), 1481 (w), 1455 (w), 1424 (w), 1392 (w), 1365 (w), 1258 (w), 1218 (w), 1159 (s), 1072 (w), 1048 (w), 993 (w), 906 (w), 878 (w), 750 (m), 698 (m), 651 (w), 612 (w) cm^{-1}; **^{1}H NMR (400 MHz, $CDCl_3$)** δ 7.42 – 7.28 (m, 10H), 6.63 (dd, *J* = 17.6, 11.2 Hz, 1H), 5.22 (d, *J* = 17.2 Hz, 1H), 5.11 – 5.08 (m, 5H), 3.27 – 3.04 (m, 2H), 2.82 (d, *J* = 13.2 Hz, 3H), 2.68 – 2.55 (m, 2H), 2.55 – 2.39 (m, 6H), 2.39 – 2.28 (m, 2H), 1.46 (s, 9H).**^{13}C NMR (100 MHz, $CDCl_3$)** δ 172.9, 172.5, 155.4, 135.9, 135.8, 135.0, 133.4, 133.0, 128.50, 128.48, 128.25, 128.20, 128.1, 113.6, 79.5, 66.3,

66.2, 48.3, 35.1, 34.5, 33.6, 33.1, 30.2, 28.4, 22.8; **HRMS (ESI) [M+Na]$^+$** Calcd for $C_{32}H_{41}NO_6Na$: 558.28261 m/z, Found: 558.28264 m/z.

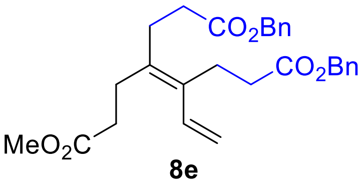

8e

Dibenzyl (*E*)-4-(3-methoxy-3-oxopropyl)-5-vinyloct-4-enedioate (8e)

Colorless oil, 80% yield. **IR (neat):** 3882 (w), 3673 (w), 2972 (m), 2902 (m), 2362 (w), 1733 (s), 1497 (w), 1453 (w), 1409 (w), 1381 (w), 1257 (w), 1164 (m), 1069 (m), 1052 (w), 902 (w),801 (w), 749 (w), 698 (m), 634 (w) cm^{-1}; **^{1}H NMR (400 MHz, $CDCl_3$)** δ 7.36 – 7.33 (m, 10H), 6.58 (dd, *J* = 17.6, 11.2 Hz, 1H), 5.21 (dd, *J* = 17.6, 1.2 Hz, 1H), 5.16 – 5.04 (m, 5H), 3.64 (s, 3H), 2.62 – 2.58 (m, 2H), 2.50 – 2.37 (m, 8H), 2.37 – 2.29 (m, 2H); **^{13}C NMR (100 MHz, $CDCl_3$)** δ 173.1, 172.8, 172.5, 136.5, 135.9, 135.8, 132.9, 132.9, 128.52, 128.50, 128.25, 128.20, 128.17, 128.1, 113.9, 66.4, 66.2, 51.6, 33.7, 33.4, 33.1, 27.9, 26.7, 22.9; **HRMS (ESI) [M+Na]$^+$** Calcd for $C_{28}H_{32}O_6Na$: 487.20911 m/z, Found: 487.20988 m/z.

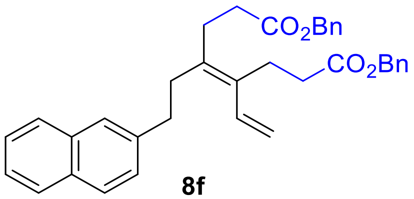

8f

Dibenzyl (*E*)-4-(2-(naphthalen-2-yl)ethyl)-5-vinyloct-4-enedioate (8f)

Pale yellow oil, 90% yield. **IR (neat):** 3032 (w), 2955 (w), 1733 (s), 1627 (w), 1599 (w), 1501 (w), 1454 (w), 1417 (w), 1380 (w), 1352 (w), 1260 (w), 1233 (w), 1161 (s), 1065 (w), 990 (w), 903 (w), 854 (w), 817 (w), 746 (m), 697 (m), 642 (w), 620 (w) cm^{-1}; **^{1}H NMR (400 MHz, $CDCl_3$)** δ 7.87 – 7.81 (m, 3H), 7.65 (s, 1H), 7.54 – 7.45 (m, 2H), 7.45 – 7.32 (m, 11H), 6.70 (dd, *J* = 17.2, 11.2 Hz, 1H), 5.27 (dd, *J* = 17.6, 1.2 Hz, 1H), 5.20 (s, 2H), 5.19 (s, 2H), 5.13 (dd, *J* = 11.2, 1.2 Hz, 1H), 2.91 – 2.84 (m, 2H), 2.73 – 2.69 (m, 2H), 2.61 – 2.49 (m, 8H); **^{13}C NMR (100 MHz, $CDCl_3$)** δ 173.0, 172.7, 139.0, 137.9, 135.9, 135.8, 133.5, 133.1, 132.2, 132.0, 128.50, 128.49, 128.21, 128.17, 128.14, 128.13, 127.9, 127.6, 127.3, 127.1, 126.3, 125.9, 125.1, 113.3, 66.3, 66.2, 35.6, 33.7, 33.4, 33.2, 28.4, 22.8; **HRMS (ESI) [M+Na]$^+$** Calcd for $C_{36}H_{36}O_4Na$: 555.25058 m/z, Found: 555.25130 m/z.

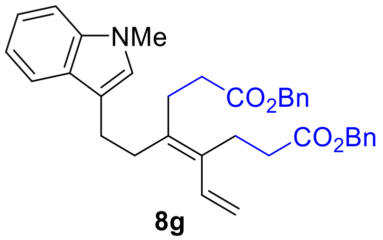

8g

Dibenzyl (*E*)-4-(2-(1-methyl-1H-indol-3-yl)ethyl)-5-vinyloct-4-enedioate (8g)

Colorless oil, 85% yield. **IR (neat):** 3929 (br), 3034 (w), 2941 (w), 1732 (s), 1615 (w), 1470 (w), 1455 (w), 1422 (w), 1378 (w), 1353 (w), 1324 (w), 1259 (w), 1231 (w), 1160 (s), 1069 (w), 89 (w), 905 (w), 803 (w), 738 (w), 697 (s), 616 (w) cm^{-1}; **^{1}H NMR (400 MHz, $CDCl_3$)** δ 7.55 – 7.53 (m, 1H), 7.35 – 7.26 (m, 11H), 7.24 – 7.17 (m, 2H), 7.09 – 7.05 (m, 1H), 6.63 (dd, *J* = 17.6, 11.2 Hz, 1H), 5.17 (dd, *J* = 17.6, 1.2 Hz, 1H), 5.10 (s, 2H), 5.09 (s, 2H), 5.03 (dd, *J* = 11.2, 1.2 Hz, 1H), 3.69 (s, 3H), 2.79 – 2.75 (m, 2H), 2.64 – 2.60 (m, 2H), 2.54 – 2.48 (m, 4H), 2.47 – 2.39 (m, 4H); **^{13}C NMR (100 MHz, $CDCl_3$)** δ 173.0, 172.8, 138.7, 136.9, 136.0, 135.9, 133.4, 131.9, 128.5, 128.2, 128.2, 128.1, 127.7, 126.0, 121.5, 118.8, 118.6, 114.5, 113.0, 109.1, 66.3, 66.2, 33.8, 33.3, 32.6, 32.5, 28.5, 24.8, 22.8; **HRMS (ESI) $[M+Na]^+$** Calcd for $C_{35}H_{37}NO_4Na$: 558.26148 m/z, Found: 558.26170 m/z.

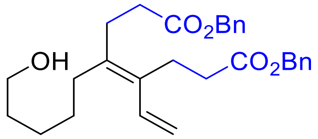

8h

Dibenzyl (*E*)-4-(5-hydroxypentyl)-5-vinyloct-4-enedioate (8h)

Colorless oil, 88% yield. **IR (neat):** 3894 (w), 3845 (w), 3826 (w), 3738 (w), 3525 (w), 3035 (w), 2936 (w), 2861 (w), 1732 (s), 1667 (w), 1497 (w), 1455 (w), 1418 (w), 1382 (w), 1354 (w), 1262 (w), 1213 (w), 1162 (s), 1048 (w), 1027 (w), 906 (w), 802 (w), 748 (m), 698 (s), 657 (w) cm^{-1}; **^{1}H NMR (400 MHz, $CDCl_3$)** δ 7.37 – 7.33 (m, 10H), 6.60 (dd, *J* = 17.6, 11.2 Hz, 1H), 5.18 (dd, *J* = 17.6, 1.2 Hz, 1H), 5.12 (s, 2H), 5.11 (s, 2H), 5.06 (dd, *J* = 11.2, 1.2 Hz, 1H), 3.62 (t, *J* = 6.8 Hz, 2H), 2.69 – 2.58 (m, 2H), 2.48 – 2.40 (m, 6H), 2.23 – 2.03 (m, 2H), 1.64 – 1.50 (m, 3H), 1.40 – 1.34 (m, 4H). **^{13}C NMR (100 MHz, $CDCl_3$)** δ 173.0, 172.8, 138.9, 135.9, 135.8, 133.4, 131.4, 128.5, 128.5, 128.2, 128.2, 128.1, 112.8, 66.3, 66.2, 62.7, 33.8, 33.2, 32.5, 31.4, 29.1, 28.2, 25.8, 22.8; **HRMS (ESI) $[M+Na]^+$** Calcd for $C_{29}H_{36}O_5Na$: 487.24550 m/z, Found: 487.24610 m/z.

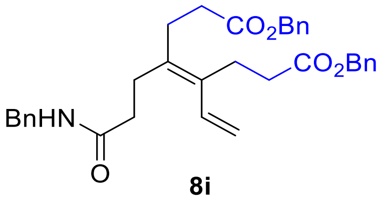

8i

Dibenzyl (*E*)-4-(3-(benzylamino)-3-oxopropyl)-5-vinyloct-4-enedioate (8i)

White solid, m.p. 82.6-83.6 °C, 87% yield. **IR (neat):** 3297 (w), 3031 (w), 2921 (w), 2851 (w), 1729 (s), 1641 (m), 1543 (w), 1497 (w), 1455 (w), 1420 (w), 1381 (w), 1352 (w), 1305 (w), 1280 (w), 1265 (w), 1243 (w), 1218 (w), 1173 (m), 1132 (w), 1074 (w), 1020 (w), 989 (w), 967 (w), 929 (w), 903 (w), 746 (m), 736 (m), 694 (s), 635 (w), 617 (w) cm^{-1}; **^{1}H NMR (400 MHz, $CDCl_3$)** δ 7.31 – 7.26 (m, 11H), 7.23 – 7.21 (m, 4H), 6.56 (dd, *J* = 17.6, 11.2 Hz, 1H), 5.73 (t, *J* = 5.6 Hz, 1H), 5.15 (d, *J* = 17.6 Hz, 1H), 5.07 – 4.99 (m, 5H), 4.35 (d, *J* = 5.6 Hz, 2H), 2.59 – 2.50 (m, 2H), 2.46 (t, *J* = 7.6 Hz, 2H), 2.40 (s, 4H), 2.36 – 2.26 (m, 2H), 2.20 – 2.12 (m, 2H); **^{13}C NMR (100 MHz, $CDCl_3$)** δ 173.0, 172.7, 171.8, 138.2, 137.3, 135.9, 135.8, 133.1, 132.7, 128.7, 128.6, 128.3, 128.3, 128.2, 128.0, 127.5, 113.8, 66.4, 66.3, 43.8, 36.0, 33.7, 33.1, 28.1, 27.2, 22.9; **HRMS (ESI) $[M+Na]^+$** Calcd for $C_{34}H_{37}NO_5Na$: 487.24550 m/z, Found: 487.24610 m/z.

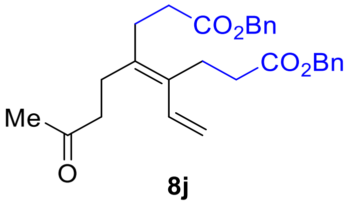

8j

Dibenzyl (*E*)-4-(3-oxobutyl)-5-vinyloct-4-enedioate (8j)

Pale yellow oil, 73% yield. **IR (neat):** 3829 (w), 3034 (w), 2957 (w), 1732 (s), 1625 (w), 1497 (w), 1454 (w), 1416 (w), 1380 (w), 1356 (w), 1260 (w), 1231 (w), 1162 (s), 1079 (w), 991 (w), 907 (w), 799 (w), 740 (m), 698 (w), 646 (m), 631 (w), 614 (w) cm^{-1}; **^{1}H NMR (400 MHz, $CDCl_3$)** δ 7.40 – 7.27 (m, 10H), 6.53 (dd, *J* = 17.6, 11.2 Hz, 1H), 5.20 (dd, *J* = 17.6, 1.2 Hz, 1H), 5.16 – 5.06 (m, 5H), 2.62 – 2.58 (m, 2H), 2.50 – 2.32 (m, 10H), 2.10 (s, 3H); **^{13}C NMR (100 MHz, $CDCl_3$)** δ 207.8, 172.9, 172.6, 137.2, 135.9, 135.8, 132.9, 132.3, 128.52, 128.50, 128.3, 128.21, 128.15, 113.8, 66.4, 66.2, 42.9, 33.7, 33.1, 29.8, 28.1, 25.2, 22.8; **HRMS (ESI) $[M+Na]^+$** Calcd for $C_{28}H_{32}O_5Na$: 471.21420m/z, Found: 471.21425 m/z.

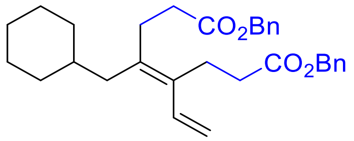

8k

Dibenzyl (*Z*)-4-(cyclohexylmethyl)-5-vinyloct-4-enedioate (8k)

Colorless oil, 90% yield. **IR (neat):** 3747 (w), 3063 (w), 3034 (w), 2923 (w), 2850 (w), 1734 (s), 1623 (w), 1497 (w), 1452 (w), 1418 (w), 1380 (w), 1352 (w), 1260 (w), 1231 (w), 1213 (w), 1157 (s), 1078 (w), 990 (w), 903 (w), 748 (m), 697 (s), 645 (w), 620 (w) cm^{-1}; **^{1}H NMR (400 MHz, $CDCl_3$)** δ 7.37 – 7.34 (m, 10H), 6.66 (dd, *J* = 17.6, 11.2 Hz, 1H), 5.18 (dd, *J* = 17.6, 1.2 Hz, 1H), 5.13 (s, 2H), 5.12 (s, 2H), 5.03 (dd, *J* = 11.2, 1.2 Hz, 1H), 2.65 – 2.61 (m, 2H), 2.52 – 2.35 (m, 6H), 2.05 (d, *J* = 7.2 Hz, 2H), 1.73 – 1.60 (m, 5H), 1.43 – 1.37 (m, 1H), 1.27 – 1.07 (m, 3H), 0.94 – 0.88 (m, 2H); **^{13}C NMR (100 MHz, $CDCl_3$)** δ 173.0, 172.9, 138.0, 135.9, 135.9, 133.9, 132.4, 128.52, 128.50, 128.22, 128.17, 128.14, 128.13, 112.3, 66.3, 66.2, 38.5, 37.8, 33.9, 33.35, 33.26, 28.4, 26.4, 26.4, 22.9; **HRMS (ESI) $[M+Na]^+$** Calcd for $C_{31}H_{38}O_4Na$: 497.26623 m/z, Found: 497.26658 m/z.

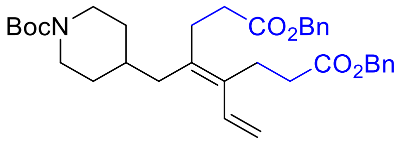

8l

Dibenzyl (*Z*)-4-((1-(tert-butoxycarbonyl)piperidin-4-yl)methyl)-5-vinyloct-4-enedioate (8l)

Colorless oil, 51% yield. **IR (neat):** 2928 (w), 2853 (w), 2362 (w), 1734 (m), 1689 (m), 1453 (w), 1422 (w), 1384 (w), 1363 (w), 1244 (w), 1159 (s), 1113 (w), 1055 (w), 1023 (w), 967 (w), 904 (w), 865 (w), 800 (w), 747 (w), 698 (m), 659 (w) cm^{-1}; **^{1}H NMR (400 MHz, $CDCl_3$)** δ 7.44 – 7.32 (m, 10H), 6.62 (dd, *J* = 17.2, 11.2 Hz, 1H), 5.26 – 5.18 (m, 1H), 5.14 (s, 2H), 5.14 (s, 2H), 5.08 (d, *J* = 11.2 Hz, 1H), 4.10 (s, 2H), 2.71 – 2.55 (m, 4H), 2.51 – 2.43 (m, 6H), 2.11 (d, *J* = 6.8 Hz, 2H), 1.61 – 1.52 (m, 3H), 1.48 (s, 9H), 1.11 (d, *J* = 12.8 Hz, 2H); **^{13}C NMR (100 MHz, $CDCl_3$)** δ 172.9, 172.7, 154.7, 136.8, 135.8, 135.7, 133.4, 133.0, 128.5, 128.25, 128.22, 128.18, 113.1, 79.2, 66.4, 66.2, 37.5, 36.1, 33.8, 33.2, 32.1, 28.4, 28.4, 22.9; **HRMS (ESI) $[M+Na]^+$** Calcd for $C_{35}H_{45}NO_6Na$: 598.31391 m/z, Found: 598.31462 m/z.

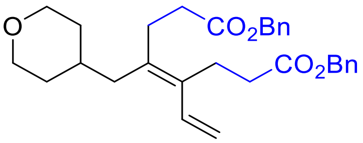

8m

Dibenzyl (*Z*)-4-((tetrahydro-2H-pyran-4-yl)methyl)-5-vinyloct-4-enedioate (8m)

Colorless oil, 77% yield. **IR (neat):** 3033 (w), 2928 (w), 2841 (w), 1733 (s), 1623 (w), 1496 (w), 1455 (w), 1381 (w), 1353 (w), 1297 (w), 1261 (w), 1233 (w), 1158 (m), 1091 (w), 1053 (w), 1013 (w), 984 (w), 903 (w), 848 (w), 803 (w), 746 (w), 698 (w), 677 (m), 655 (w) cm^{-1}; **^{1}H NMR (400 MHz, CDCl$_3$)** δ 7.36 – 7.33 (m, 10H), 6.61 (dd, *J* = 17.6, 11.2 Hz, 1H), 5.19 (d, *J* = 17.6 Hz, 1H), 5.11 (s, 2H), 5.10 (s, 2H), 5.05 (d, *J* = 11.2 Hz, 1H), 3.90 (dd, *J* = 11.2, 4.4 Hz, 2H), 3.29 (td, *J* = 11.6, 2.0 Hz, 2H), 2.64 – 2.60 (m, 2H), 2.49 – 2.39 (m, 6H), 2.09 (d, *J* = 7.2 Hz, 2H), 1.64 – 1.59 (m, 1H), 1.48 (d, *J* = 13.2 Hz, 2H), 1.32 – 1.25 (m, 2H); **^{13}C NMR (100 MHz, CDCl$_3$)** δ 172.9, 172.7, 136.7, 135.8, 135.8, 133.5, 133.1, 128.53, 128.51, 128.25, 128.22, 128.17, 128.1, 113.0, 68.0, 66.4, 66.2, 37.9, 35.1, 33.9, 33.2, 33.1, 28.4, 22.9; **HRMS (ESI) [M+Na]$^+$** Calcd for $C_{30}H_{36}O_5Na$: 499.24550 m/z, Found: 499.24523 m/z.

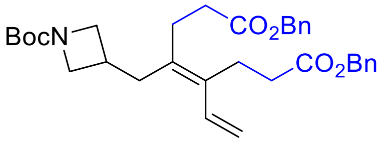

8n

Dibenzyl (*Z*)-4-((1-(tert-butoxycarbonyl)azetidin-3-yl)methyl)-5-vinyloct-4-enedioate (8n)

Colorless oil, 87% yield. **IR (neat):** 2970 (w), 2879 (w), 1734 (m), 1698 (m), 1625 (w), 1479 (w), 1454 (m), 1399 (w), 1363 (w), 1294 (w), 1257 (w), 1160 (s), 1064 (w), 991 (w), 907 (w), 860 (w), 748 (m), 698 (m), 632 (w) cm^{-1}; **^{1}H NMR (400 MHz, CDCl$_3$)** δ 7.35 (s, 10H), 6.66 (dd, *J* = 17.2, 11.2 Hz, 1H), 5.23 (d, *J* = 17.2 Hz, 1H), 5.18 – 5.00 (m, 5H), 3.91 (t, *J* = 8.4 Hz, 2H), 3.53 (dd, *J* = 8.4, 5.4 Hz, 2H), 2.61 (t, *J* = 8.0 Hz, 3H), 2.45 – 2.39 (m, 8H), 1.43 (s, 9H); **^{13}C NMR (100 MHz, CDCl$_3$)** δ 172.8, 172.4, 156.3, 135.8, 135.7, 135.7, 133.4, 133.1, 128.50, 128.47, 128.24, 128.22, 128.1, 114.1, 79.2, 66.4, 66.2, 54.0, 35.0, 33.7, 33.2, 28.3, 28.3, 27.7, 23.0; **HRMS (ESI) [M+Na]$^+$** Calcd for $C_{33}H_{41}NO_6Na$: 570.28261 m/z, Found: 570.28264 m/z.

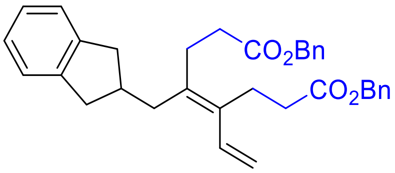

8o

Dibenzyl (*Z*)-4-((2,3-dihydro-1H-inden-2-yl)methyl)-5-vinyloct-4-enedioate (8o)

Colorless oil, 96% yield. **IR (neat):** 3653 (w), 2947 (br), 1734 (s), 1623 (w), 1455 (w), 1380 (w), 1352 (w), 1259 (m), 1231 (w), 1162 (s), 1069 (w), 1025 (w), 993 (w), 903 (w), 743 (s), 698 (s), 626 (w) cm^{-1}; **^{1}H NMR (400 MHz, $CDCl_3$)** δ 7.38 – 7.32 (m, 10H), 7.21 – 7.09 (m, 4H), 6.58 (dd, *J* = 17.6, 11.2 Hz, 1H), 5.21 (d, *J* = 17.6 Hz, 1H), 5.15 (s, 2H), 5.14 (s, 2H), 5.03 (d, *J* = 11.2 Hz, 1H), 2.98 – 2.91 (m, 2H), 2.69 – 2.65 (m, 2H), 2.62 – 2.54 (m, 5H), 2.52 – 2.45 (m, 4H), 2.33 (d, *J* = 6.4 Hz, 2H); **^{13}C NMR (100 MHz, $CDCl_3$)** δ 173.0, 172.7, 143.0, 137.8, 135.9, 135.8, 133.5, 132.7, 128.5, 128.24, 128.19, 128.17, 128.15, 126.1, 124.4, 113.0, 66.3, 66.2, 39.5, 38.6, 35.8, 33.9, 33.3, 28.0, 23.0;**HRMS (ESI) [M+Na]$^+$** Calcd for $C_{34}H_{36}O_4Na$: 531.25058 m/z, Found: 531.24971 m/z.

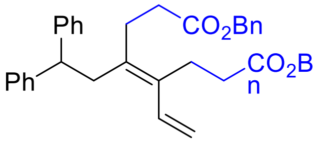

8p

Dibenzyl (*Z*)-4-(2,2-diphenylethyl)-5-vinyloct-4-enedioate (8p)

Colorless oil, 83% yield. **IR (neat):** 3029 (w), 2956 (w), 1733 (m), 1599 (w), 1494 (w), 1452 (w), 1417 (w), 1380 (w), 1353 (w), 1260 (w), 1232 (w), 1161 (m), 1081 (w), 1061 (w), 1029 (w), 989 (w), 905 (w), 789 (w), 749 (m), 698 (s), 641 (w) cm^{-1}; **^{1}H NMR (400 MHz, $CDCl_3$)** δ 7.41 – 7.29 (m, 10H), 7.25 – 7.21 (m, 4H), 7.18 – 7.16 (m, 6H), 6.39 (dd, *J* = 17.2, 11.2 Hz, 1H), 5.17 – 5.06 (m, 5H), 4.94 (d, *J* = 11.2 Hz, 1H), 4.05 (t, *J* = 7.6 Hz, 1H), 2.88 (d, *J* = 7.6 Hz, 2H), 2.58 – 2.51 (m, 2H), 2.36 – 2.29 (m, 4H), 2.19 – 2.15 (m, 2H); **^{13}C NMR (100 MHz, $CDCl_3$)** δ 172.9, 172.6, 144.1, 136.7, 135.9, 135.8, 133.3, 133.2, 128.52, 128.49, 128.3, 128.20, 128.16, 128.0, 126.3, 113.1, 66.2, 66.2, 50.6, 36.9, 33.6, 33.1, 28.2, 23.0; **HRMS (ESI) [M+Na]$^+$** Calcd for $C_{38}H_{38}O_4Na$: 581.26623 m/z, Found: 581.26711 m/z.

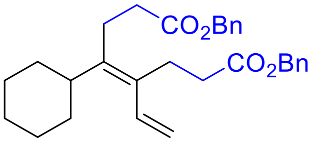

8q

Dibenzyl (*Z*)-4-cyclohexyl-5-vinyloct-4-enedioate (8q)

Colorless oil, 53% yield, *Z*:*E* = 87:13. **IR (neat):** 3440 (w), 3034 (m), 2928 (w), 2852 (w), 1734 (s), 1618 (w), 1497 (w), 1452 (w), 1419 (w), 1380 (w), 1353 (w), 1261 (m), 1228 (w), 1157 (s), 1059 (w), 1027 (w), 987 (w), 903 (w), 802 (w), 737 (m), 697 (w), 646 (s), 615 (w) cm^{-1}; **^{1}H NMR (400 MHz, $CDCl_3$)** δ 7.38 – 7.31 (m, 10H), 6.71 (dd, *J* = 17.6, 11.2 Hz, 1H), 5.19 (d, *J* = 17.2 Hz, 1H), 5.13 (s, 2H), 5.11 (s, 2H), 5.07 (d, *J* = 11.2 Hz, 1H), 2.71 – 2.64 (m, 1H), 2.62 – 2.57 (m, 2H), 2.48 – 2.39 (m, 6H), 1.79 – 1.72 (m, 2H), 1.68 (d, *J* = 13.2 Hz, 2H), 1.50 (d, *J* = 9.2 Hz, 2H), 1.36 – 1.27 (m, 5H), 1.21 – 1.09 (m, 1H); **^{13}C NMR (100 MHz, $CDCl_3$)** δ 173.1, 172.7, 143.1, 136.0, 135.9, 133.1, 130.9, 128.53, 128.50, 128.19, 128.18, 128.14, 128.12, 113.1, 66.3, 66.2, 41.0, 34.6, 33.7, 31.4, 26.6, 26.0, 24.3, 23.5; **HRMS (ESI) $[M+Na]^+$** Calcd for $C_{30}H_{36}O_4Na$: 483.25058 m/z, Found: 483.25013 m/z.

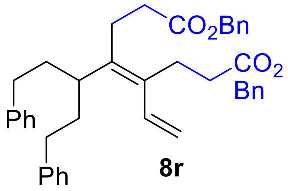

8r

Dibenzyl (Z)-4-(1,5-diphenylpentan-3-yl)-5-vinyloct-4-enedioate (8r)

Colorless oil, 61% yield, *Z*:*E* = 83:17. **IR (neat):** 3061 (w), 3028 (w), 2934 (w), 2857 (w), 1734 (m), 1603 (w), 1495 (w), 1454 (w), 1380 (w), 1353 (w), 1260 (w), 1231 (w), 1158 (m), 1082 (w), 1028 (w), 986 (w), 905 (w), 802 (w), 746 (m), 697 (s), 652 (w), 633 (w), 618 (w) cm^{-1}; **^{1}H NMR (400 MHz, $CDCl_3$)** δ 7.45 – 7.33 (m, 10H), 7.33 – 7.27 (m, 3H), 7.24 – 7.17 (m, 2H), 7.17 – 7.08 (m, 5H), 6.54 (dd, *J* = 17.6, 11.2 Hz, 1H), 5.23 (d, *J* = 17.2 Hz, 1H), 5.16 (s, 2H), 5.15 (s, 2H), 5.05 (d, *J* = 11.2 Hz, 1H), 2.92 (s, 1H), 2.75 – 2.70 (m, 2H), 2.58 – 2.43 (m, 10H), 1.85 – 1.69 (m, 4H); **^{13}C NMR (100 MHz, $CDCl_3$)** δ 173.0, 172.6, 142.1, 139.8, 135.9, 135.8, 134.5, 133.3, 128.6, 128.5, 128.33, 128.31, 128.21, 128.21, 128.18, 125.7, 113.5, 66.4, 66.3, 40.3, 35.7, 34.7, 33.9, 33.7, 23.9, 22.9; **HRMS (ESI) $[M+Na]^+$** Calcd for $C_{41}H_{44}O_4Na$: 623.31318 m/z, Found: 623.31398 m/z.

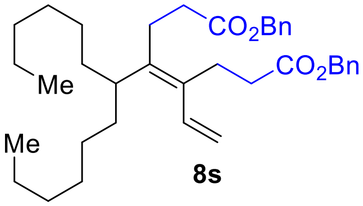

8s

Dibenzyl (*Z*)-4-(tridecan-7-yl)-5-vinyloct-4-enedioate (8s)

Colorless oil, 54% yield, *Z:E* = 92:8. **IR (neat):** 3068 (w), 3037 (w), 2955 (m), 2926 (w), 2855 (w), 1736 (s), 1610 (w), 1497 (w), 1456 (w), 1419 (w), 1379 (w), 1354 (w), 1260 (w), 1214 (w), 1158 (s), 1026 (w), 905 (w), 803 (w), 748 (m), 697 (s), 659 (w), 644 (w), 614 (w) cm^{-1}; **^{1}H NMR (400 MHz, $CDCl_3$)** δ 7.44 – 7.27 (m, 10H), 6.72 (dd, *J* = 17.6, 11.2 Hz, 1H), 5.18 (d, *J* = 17.2 Hz, 1H), 5.13 (s, 2H), 5.12 (s, 2H), 5.04 (d, *J* = 11.2 Hz, 1H), 2.77 (s, 1H), 2.67 – 2.60 (m, 2H), 2.49 – 2.35 (m, 5H), 1.41 – 1.17 (m, 17H), 1.15 – 1.10 (m, 4H), 0.86 (t, *J* = 6.8 Hz, 6H); **^{13}C NMR (100 MHz, $CDCl_3$)** δ 173.1, 172.9, 141.1, 135.9, 135.9, 133.4, 128.5, 128.2, 128.2, 128.0, 112.9, 66.3, 66.2, 40.9, 34.6, 34.0, 33.7, 31.8, 29.5, 27.7, 23.8, 22.6, 14.1; **HRMS (ESI) $[M+Na]^+$** Calcd for $C_{37}H_{52}O_4Na$: 583.37578 m/z, Found: 583.37568 m/z.

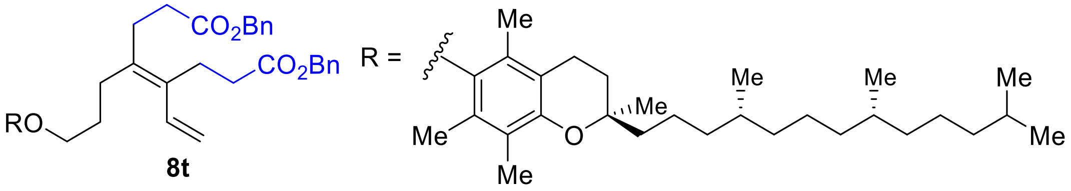

8t

Dibenzyl (*E*)-4-(3-(((*R*)-2,5,7,8-tetramethyl-2-((4*R*,8*R*)-4,8,12-trimethyltridecyl)chroman-6-yl)oxy)propyl)-5-vinyloct-4-enedioate (8t)

Colorless oil, 90% yield. **IR (neat):** 3851 (br), 3326 (w), 3220 (w), 3033 (w), 2925 (m), 2866 (w), 2359 (w), 1736 (s), 1625 (w), 1497 (w), 1457 (m), 1415 (w), 1378 (w), 1353 (w), 1257 (m), 1158 (s), 1088 (m), 1047 (w), 990 (w), 942 (w), 903 (w), 846 (w), 804 (w), 737 (m), 697 (s), 637 (w), 620 (w) cm^{-1}; **^{1}H NMR (400 MHz, $CDCl_3$)** δ 7.41 – 7.28 (m, 10H), 6.71 (dd, *J* = 17.2, 11.2 Hz, 1H), 5.23 (dd, *J* = 17.6, 1.2 Hz, 1H), 5.17 – 5.04 (m, 5H), 3.64 (t, *J* = 6.4 Hz, 2H), 2.70 – 2.62 (m, 2H), 2.62 – 2.53 (m, 4H), 2.53 – 2.44 (m, 4H), 2.44 – 2.35 (m, 2H), 2.17 (s, 3H), 2.12 (s, 3H), 2.10 (s, 3H), 1.90 – 1.73 (m, 4H), 1.58 – 1.51 (m, 3H), 1.45 – 1.35 (m, 4H), 1.35 – 1.20 (m, 11H), 1.20 – 1.14 (m, 2H), 1.14 – 1.02 (m, 4H), 0.90 – 0.86 (m, 12H); **^{13}C NMR (100 MHz, $CDCl_3$)** δ 173.0, 172.7, 148.3, 147.7, 138.4, 136.0, 135.9, 133.4, 131.8, 128.53, 128.51, 128.3, 128.19, 128.17, 128.1, 127.7, 125.7, 122.8, 117.5, 113.1, 74.7, 72.5, 66.3, 66.2, 40.03, 39.98, 39.3, 37.6, 37.53, 37.46, 37.44, 37.42, 37.38, 37.35, 37.33, 37.30, 37.25, 33.8, 33.3, 32.8, 32.7, 32.7, 32.6, 31.3, 31.2, 30.0, 28.3, 28.2, 27.9, 24.8, 24.8, 24.4, 23.8, 22.9, 22.7, 22.6, 21.0, 21.0, 20.6, 19.73, 19.66, 19.63, 19.60, 19.57, 12.8, 11.9, 11.8; **HRMS (ESI) $[M+Na]^+$** Calcd for $C_{56}H_{80}O_6Na$: 871.58471m/z, Found: 871.58356 m/z.

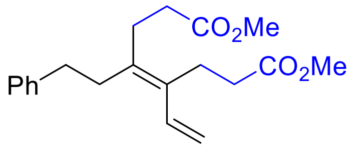

9a

Dimethyl (*E*)-4-phenethyl-5-vinyloct-4-enedioate (9a)

Colorless oil, 75% yield. **IR (neat):** 3024 (w), 2950 (w), 1736 (s), 1625 (w), 1601 (w), 1494 (w), 1437 (m), 1362 (w), 1301 (w), 1256 (w), 1194 (w), 1169 (m), 1061 (w), 987 (w), 901 (w), 747 (w), 700 (m) cm^{-1}; **^{1}H NMR (400 MHz, $CDCl_3$)** δ 7.31 – 7.25 (m, 2H), 7.22 – 7.15 (m, 3H), 6.58 (dd, *J* = 17.4, 11.2 Hz, 1H), 5.19 (dd, *J* = 17.4, 1.2 Hz, 1H), 5.06 (dd, *J* = 11.2, 1.2 Hz, 1H), 3.69 (s, 3H),3.68 (s, 3H), 2.73 – 2.63 (m, 2H), 2.63 – 2.55 (m, 2H), 2.50 – 2.33 (m, 8H). **^{13}C NMR (100 MHz, $CDCl_3$)** δ 173.7, 173.4, 141.5, 137.9, 133.1, 132.0, 128.3, 128.3, 126.0, 113.1, 51.7, 51.6, 35.4, 33.6, 33.5, 33.0, 28.3, 22.8; **HRMS (ESI) [M+Na]$^+$** Calcd for $C_{20}H_{26}O_4Na$: 353.17233 m/z, Found: 353.17179m/z.

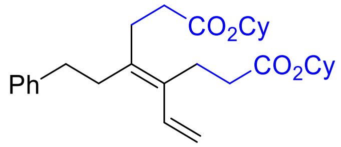

9b

Dicyclohexyl (*E*)-4-phenethyl-5-vinyloct-4-enedioate (9b)

Colorless oil, 49% yield. **IR (neat):** 3026 (w), 2935 (m), 2858 (w), 1728 (s), 1624 (w), 1600 (w), 1494 (m), 1450 (w), 1358 (w), 1258 (w), 1173 (s), 1123 (w), 1040 (w), 1016 (w), 987 (w), 965 (w), 905 (w), 803 (w), 746 (m), 699 (w) cm^{-1}; **^{1}H NMR (400 MHz, $CDCl_3$)** δ 7.33 – 7.23 (m, 2H), 7.18 – 7.16 (m, 3H), 6.58 (dd, *J* = 17.6, 11.2 Hz, 1H), 5.20 (d, *J* = 17.6 Hz, 1H), 5.04 (d, *J* = 11.2 Hz, 1H), 4.77 – 4.72 (m, 2H), 2.68 – 2.60 (m, 2H), 2.60 – 2.56 (m, 2H), 2.43 (dd, *J* = 9.6, 6.4 Hz, 4H), 2.35 (q, *J* = 7.6 Hz, 4H), 1.86 – 1.80 (m, 4H), 1.75 – 1.68 (m, 4H), 1.58 – 1.48 (m, 2H), 1.38 – 1.33 (m, 8H), 1.24 (d, *J* = 9.6 Hz, 2H); **^{13}C NMR (100 MHz, $CDCl_3$)** δ 172.7, 172.4, 141.6, 138.0, 133.2, 132.1, 128.3, 128.3, 125.9, 113.0, 72.7, 72.5, 35.5, 34.2, 33.7, 33.6, 31.6, 31.6, 28.5, 25.4, 25.3, 23.7, 23.0; **HRMS (ESI) [M+Na]$^+$** Calcd for $C_{30}H_{42}O_4Na$: 489.29753 m/z, Found: 489.29674 m/z.

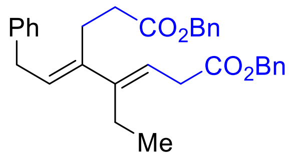

10a

Dibenzyl (3*E*,5*E*)-4-ethyl-5-(2-phenylethylidene)oct-3-enedioate (10a)

Colorless oil, 79% yield. **IR (neat):** 3030 (w), 2965 (w), 1734 (s), 1603 (w), 1495 (w), 1454 (w), 1379 (w), 1325 (w), 1259 (w), 1155 (s), 1057 (w), 1025 (w), 974 (w), 909 (w), 798 (w), 740 (m), 697 (s), 674 (w) cm^{-1};**^{1}H NMR (400 MHz, $CDCl_3$)** δ 7.40 – 7.27 (m, 12H), 7.23 – 7.15 (m, 3H), 5.68 (t, *J* = 7.2 Hz, 1H), 5.60 (t, *J* = 7.2 Hz, 1H), 5.14 (s, 2H), 5.12 (s, 2H), 3.48 (d, *J* = 7.4 Hz, 2H), 3.19 (d, *J* = 7.2 Hz, 2H), 2.68 (t, *J* = 8.0 Hz, 2H), 2.42 (t, *J* = 8.0 Hz, 2H), 2.21 (q, *J* = 7.6 Hz, 2H), 0.93 (t, *J* = 7.6 Hz, 3H). **^{13}C NMR (100 MHz, $CDCl_3$)** δ 172.9, 171.7, 145.1, 140.8, 139.3, 135.9, 135.8, 128.5, 128.4, 128.3, 128.21, 128.16, 128.15, 128.12, 126.9, 125.9, 117.2, 66.4, 66.2, 34.2, 33.7, 33.2, 23.3, 21.4, 13.2; **HRMS (ESI) [M+Na]$^+$** Calcd for $C_{32}H_{34}O_4Na$: 505.23493 m/z, Found: 505.23479 m/z.

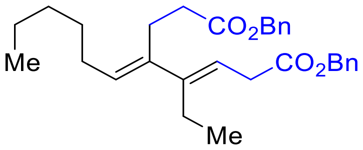

10b

dibenzyl (3*E*,5*E*)-4-ethyl-5-hexylideneoct-3-enedioate (10b)

Colorless oil, 85% yield. **IR (neat):** 3908 (w), 3034 (w), 2958 (w), 2928 (w), 2856 (w), 1735 (s), 1612 (w), 1497 (w), 1456 (w),1378 (w), 1325 (w), 1260 (w), 1214 (w), 1156 (s), 1080 (w), 1058 (w), 974 (w), 909 (w), 803 (w), 738 (m), 697 (m), 639 (w), 616 (w) cm^{-1}; **^{1}H NMR (400 MHz, $CDCl_3$)** δ 7.36 – 7.26 (m, 10H), 5.51 (t, *J* = 7.2 Hz, 1H), 5.46 (t, *J* = 7.2 Hz, 1H), 5.11 (d, *J* = 8.0 Hz, 4H), 3.17 (d, *J* = 7.2 Hz, 2H), 2.54 (t, *J* = 4.0 Hz, 2H), 2.39 – 2.28 (m, 2H), 2.17 (q, *J* = 7.2 Hz, 2H), 2.08 (q, *J* = 7.2 Hz, 2H), 1.41 – 1.32 (m, 2H), 1.32 – 1.19 (m, 4H), 0.91 – 0.87 (m, 6H)..**^{13}C NMR (100 MHz, $CDCl_3$)** δ 173.2, 171.9, 145.4, 138.0, 136.0, 135.9, 129.0, 128.53, 128.52, 128.19, 128.15, 116.4, 66.4, 66.2, 33.8, 33.4, 31.6, 29.5, 28.1, 23.3, 22.6, 21.4, 14.0, 13.2. **HRMS (ESI) [M+Na]$^+$** Calcd for $C_{30}H_{38}O_4Na$: 485.26623 m/z, Found: 485.26714 m/z.

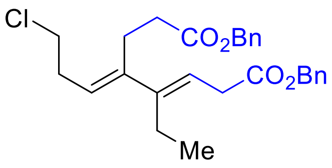

10c

Dibenzyl (3*E*,5*E*)-5-(3-chloropropylidene)-4-ethyloct-3-enedioate (10c)

Colorless oil, 69% yield. **IR (neat):** 3820 (w), 3033 (w), 2962 (w), 2364 (w), 1733 (s), 1497 (w), 1454 (w), 1379 (w), 1349 (w), 1259 (w), 1213 (w), 1155 (s), 1061 (w), 974 (w), 910 (w), 805 (w), 741 (m), 698 (m), 650 (w) cm^{-1}; **^{1}H NMR (400 MHz, $CDCl_3$)** δ 7.36 – 7.35 (m, 10H), 5.55 (t, *J* = 7.2 Hz, 1H), 5.45 (t, *J* = 7.2 Hz, 1H), 5.13 (s, 2H), 5.11 (s, 2H), 3.53 (t, *J* = 6.8 Hz, 2H), 3.18 (d, *J* = 7.2 Hz, 2H), 2.62 – 2.51 (m, 4H), 2.38 (t, *J* = 8.0 Hz, 2H), 2.18 (q, *J* = 7.2 Hz, 2H), 0.91 (t, *J* = 7.6 Hz, 3H); **^{13}C NMR (100 MHz, $CDCl_3$)** δ 172.9, 171.7, 145.0, 141.3, 135.9, 135.8, 128.5, 128.24, 128.20, 128.19, 128.16, 128.1, 123.9, 117.6, 66.4, 66.2, 44.2, 33.7, 33.1, 31.4, 23.3, 21.4, 13.0; **HRMS (ESI) [M+Na]$^+$** Calcd for $C_{27}H_{31}O_4NaCl$: 477.18031 m/z, Found: 477.18034 m/z.

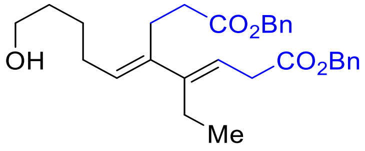

10d

Dibenzyl (3*E*,5*E*)-4-ethyl-5-(5-hydroxypentylidene)oct-3-enedioate (10d)

Colorless oil, 62% yield. **IR (neat):** 3032 (w), 2962 (w), 2934 (w), 2874 (w), 1733 (s), 1497 (w), 1455 (w), 1379 (w), 1325 (w), 1260 (w), 1213 (w), 1156 (s), 1060 (w), 1021 (m), 910 (w), 799 (w), 746 (w), 698 (s), 661 (w), 624 (w) cm^{-1}; **^{1}H NMR (400 MHz, $CDCl_3$)** δ 7.36 – 7.34 (m, 6H), 7.33 – 7.30 (m, 4H), 5.50 (t, *J* = 7.2 Hz, 1H), 5.45 (t, *J* = 7.2 Hz, 1H), 5.12 (s, 2H), 5.10 (s, 2H), 3.63 (t, *J* = 6.4 Hz, 2H), 3.16 (d, *J* = 7.2 Hz, 2H), 2.54 (t, *J* = 8.0 Hz, 2H), 2.35 (t, *J* = 8.0 Hz, 2H), 2.19 – 2.09 (m, 4H), 1.61 – 1.50 (m, 2H), 1.50 – 1.37 (m, 2H), 1.31 (s, 1H), 0.89 (t, *J* = 7.2 Hz, 3H); **^{13}C NMR (100 MHz, $CDCl_3$)** δ 173.1, 171.8, 145.3, 138.5, 135.95, 135.88, 128.5, 128.4, 128.18, 128.16, 128.14, 128.13, 116.7, 66.4, 66.2, 62.7, 33.7, 33.3, 32.3, 27.8, 25.9, 23.2, 21.4, 13.1. **HRMS (ESI) [M+Na]$^+$** Calcd for $C_{29}H_{36}O_5Na$: 487.24550 m/z, Found: 487.24511 m/z.

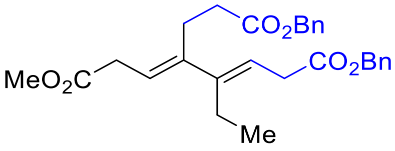

10e

1-benzyl-8-methyl (3*E*,5*E*)-5-(3-(benzyloxy)-3-oxopropyl)-4-ethylocta-3,5-dienedioate (10e)

Colorless oil, 75% yield. **IR (neat):** 2960 (w), 1734 (s), 1497 (w), 1455 (w), 1379 (w), 1259 (w), 1158 (s), 997 (w), 909 (w), 822 (w), 747 (m), 698 (m) cm^{-1}; **^{1}H NMR (400 MHz, $CDCl_3$)** δ 7.35 – 7.33 (m, 10H), 5.65 (t, *J* = 7.2 Hz, 1H), 5.57 (t, *J* = 7.2 Hz, 1H), 5.12 (s, 2H), 5.09 (s, 2H), 3.68 (s, 3H), 3.17 (dd, *J* = 7.2, 2.0 Hz, 4H), 2.54 (t, *J* = 8.0 Hz, 2H), 2.37 (t, *J* = 8.0 Hz, 2H), 2.19 (q, *J* = 7.6 Hz, 2H), 0.90 (t, *J* = 7.2 Hz, 3H); **^{13}C NMR (100 MHz, $CDCl_3$)** δ 172.8, 172.2, 171.6, 144.6, 141.5, 135.9, 135.8, 128.50, 128.49, 128.22, 128.18, 128.16, 128.1, 119.5, 117.9, 66.4, 66.2, 51.8, 33.7, 33.6, 32.9, 23.4, 21.5, 13.0. **HRMS (ESI) [M+Na]$^+$** Calcd for $C_{28}H_{32}O_6Na$: 487.20911 m/z, Found: 487.20853 m/z.

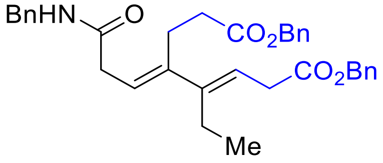

10f

Dibenzyl (3*E*,5*E*)-5-(3-(benzylamino)-3-oxopropylidene)-4-ethyloct-3-enedioate (10f)

Colorless oil, 71% yield. **IR (neat):** 3798 (w), 3295 (w), 3032 (w), 2962 (w), 1733 (s), 1648 (m), 1541 (w), 1498 (w), 1454 (w), 1379 (w), 1258 (w), 1158 (s), 1081 (w), 1026 (w), 994 (w), 910 (w), 804 (w), 737 (m), 698 (s), 634 (w) cm^{-1}; **^{1}H NMR (400 MHz, $CDCl_3$)** δ 7.35 – 7.29 (m, 10H), 7.29 – 7.26 (m, 3H), 7.25 – 7.23 (m, 2H), 6.39 (t, *J* = 5.8 Hz, 1H), 5.65 (t, *J* = 7.6 Hz, 1H), 5.51 (t, *J* = 7.2 Hz, 1H), 5.11 (s, 2H), 4.91 (s, 2H), 4.42 (d, *J* = 5.6 Hz, 2H), 3.15 (dd, *J* = 7.4, 2.4 Hz, 4H), 2.54 (t, *J* = 7.2 Hz, 2H), 2.40 (t, *J* = 6.8 Hz, 2H), 2.16 (q, *J* = 7.6 Hz, 2H), 0.85 (t, *J* = 7.6 Hz, 3H); **^{13}C NMR (100 MHz, $CDCl_3$)** δ 173.1, 171.5, 170.9, 144.6, 141.9, 138.3, 135.8, 135.7, 128.6, 128.54, 128.51, 128.3, 128.2, 127.7, 127.4, 121.2, 118.2, 66.5, 66.3, 43.6, 36.6, 33.6, 32.3, 22.8, 21.5, 13.0; **HRMS (ESI) [M+Na]$^+$** Calcd for $C_{34}H_{37}NO_5Na$: 562.25639 m/z, Found: 562.25604 m/z.

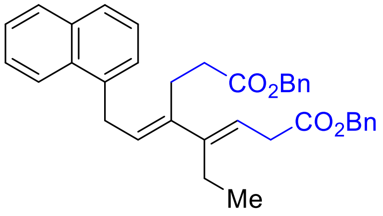

10g

Dibenzyl (3*E*,5*E*)-4-ethyl-5-(2-(naphthalen-2-yl)ethylidene)oct-3-enedioate (10g)

Colorless oil, 63% yield. **IR (neat):** 2964 (w), 2360 (w), 1733 (s),1601 (w), 1502 (w), 1455 (w), 1379 (w), 1328 (w),1260 (m),1213 (w), 1158 (s), 1087 (w), 1019 (m), 854 (w), 799 (m), 746 (m), 698 (m), 622 (w) cm^{-1}; **^{1}H NMR (400 MHz, CDCl$_3$)** δ 7.84 – 7.74 (m, 3H), 7.62 (s, 1H), 7.47 – 7.43 (m, 2H), 7.39 – 7.33 (m, 9H), 7.32 – 7.29 (m, 2H), 5.76 (t, *J* = 7.2 Hz, 1H), 5.63 (t, *J* = 7.6 Hz, 1H), 5.13 (d, *J* = 7.2 Hz, 4H), 3.65 (d, *J* = 7.2 Hz, 2H), 3.21 (d, *J* = 7.2 Hz, 2H), 2.74 (t, *J* = 8.0 Hz, 2H), 2.45 (t, *J* = 8.0 Hz, 2H), 2.23 (q, *J* = 7.6 Hz, 2H), 0.95 (t, *J* = 7.6 Hz, 3H); **^{13}C NMR (100 MHz, CDCl$_3$)** δ 173.0, 171.7, 145.1, 139.5, 138.4, 135.9, 135.9, 133.6, 132.0, 128.5, 128.2, 128.2, 128.1, 128.0, 127.6, 127.4, 127.2, 126.8, 126.3, 125.9, 125.2, 117.4, 66.4, 66.2, 34.5, 33.8, 33.3, 23.3, 21.5, 13.2. **HRMS (ESI) [M+Na]$^+$** Calcd for $C_{36}H_{36}O_4Na$: 555.25058 m/z, Found: 555.25043 m/z.

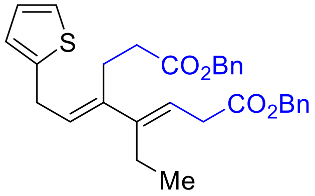

10h

Dibenzyl (3*E*,5*E*)-4-ethyl-5-(2-(thiophen-2-yl)ethylidene)oct-3-enedioate (10h)

Pale yellow oil, 85% yield. **IR (neat):** 3033 (w), 2966 (w), 2360 (w), 1733 (s), 1497 (w),1455 (w), 1380 (w), 1260 (w), 1212 (w), 1158 (s), 973 (w), 909 (w), 848 (w), 823 (w), 746 (m), 697 (s), 622 (w) cm^{-1}; **^{1}H NMR (400 MHz, CDCl$_3$)** δ 7.36 – 7.33 (m, 5H), 7.33 – 7.29 (m, 5H), 7.11 (dd, *J* = 5.2, 1.2 Hz, 1H), 6.91 (dd, *J* = 5.2, 3.2 Hz, 1H), 6.78 – 6.76 (m, 1H), 5.69 (t, *J* = 7.2 Hz, 1H), 5.58 (t, *J* = 7.2 Hz, 1H), 5.12 (s, 2H), 5.09 (s, 2H), 3.63 (d, *J* = 8.0 Hz, 2H), 3.17 (d, *J* = 7.2 Hz, 2H), 2.63 (t, *J* =8.0 Hz, 2H), 2.39 (t, *J* = 8.0 Hz, 2H), 2.19 (q, *J* = 7.6 Hz, 2H), 0.92 (t, *J* = 7.6 Hz, 3H); **^{13}C NMR (100 MHz, CDCl$_3$)** δ 172.9, 171.6, 145.0, 143.9, 139.9, 135.9, 135.9, 128.5, 128.23, 128.18, 128.17, 128.1, 126.8, 126.0, 124.1, 123.4, 117.7, 66.4, 66.2, 33.7, 33.2, 28.5, 23.2, 21.5, 13.1; **HRMS (ESI) [M+Na]$^+$** Calcd for $C_{30}H_{32}O_4NaS$: 511.19135 m/z, Found: 511.19164 m/z.

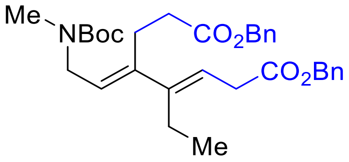

10i

Dibenzyl (3*E*,5*E*)-5-(2-((tert-butoxycarbonyl)(methyl)amino)ethylidene)-4-ethyloct-3-enedioate (10i)

Colorless oil, 71% yield. **IR (neat):** 3031 (w), 2970 (w), 2931 (w), 1736 (m), 1693 (s), 1479 (w), 1454 (w), 1421 (w), 1391 (w), 1366 (w), 1251 (w), 1151 (s), 996 (w), 881 (w), 800 (w), 748 (m), 698 (m), 617 (w) cm^{-1}; **^{1}H NMR (400 MHz, CDCl$_3$)** δ 7.34 – 7.29 (m, 10H), 5.55 (t, *J* = 7.2 Hz, 1H), 5.41 (t, *J* = 6.8 Hz, 1H), 5.12 (s, 2H), 5.09 (s, 2H), 3.91 (s, 2H), 3.17 (d, *J* = 7.2 Hz, 2H), 2.79 (s, 3H), 2.57 (t, *J* = 8.0 Hz, 2H), 2.36 (t *J* = 8.0 Hz, 2H), 2.17 (q, *J* = 7.6 Hz, 2H), 1.45 (s, 9H), 0.89 (t, *J* = 7.6 Hz, 3H); **^{13}C NMR (100 MHz, CDCl$_3$)** δ 172.7, 171.6, 155.6, 144.8, 135.9, 135.8, 128.52, 128.51, 128.23, 128.20, 128.16, 124.4, 117.8, 79.4, 66.4, 66.3, 33.7, 33.1, 28.4, 23.2, 21.4, 13.0; **HRMS (ESI) [M+Na]$^+$** Calcd for $C_{32}H_{41}NO_6Na$: 558.28261 m/z, Found: 558.28254 m/z.

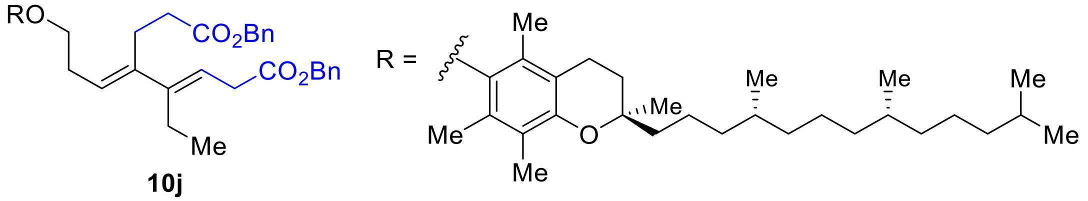

10j

Dibenzyl (3*E*,5*E*)-4-ethyl-5-(3-(((*R*)-2,5,7,8-tetramethyl-2-((4*R*,8*R*)-4,8,12-trimethyltridecyl)chroman-6-yl)oxy)propylidene)oct-3-enedioate (10j)

Colorless oil, 78% yield. **IR (neat):** 3031 (w), 2926 (m), 2866 (w), 1737 (s), 1496 (w), 1458 (m), 1415 (w), 1376 (w), 1328 (w), 1257 (m), 1156 (s), 1088 (m), 1062 (w),1020 (w), 918 (w), 802 (w), 739 (w), 697 (w), 669 (w),646 (w), 614 (w) cm^{-1}; **^{1}H NMR (400 MHz, CDCl$_3$)** δ 7.38 – 7.26 (m, 10H), 5.65 (t, *J* = 7.2 Hz, 1H), 5.56 (t, *J* = 7.2 Hz, 1H), 5.12 (s, 2H), 5.09 (s, 2H), 3.65 (t, *J* = 6.4 Hz, 2H), 3.18 (d, *J* = 7.2 Hz, 2H), 2.65 – 2.53 (m, 5H), 2.42 – 2.39 (m, 2H), 2.19 (q, *J* = 7.6 Hz, 2H), 2.15 (s, 3H), 2.10 (s, 3H), 2.07 (s, 3H), 1.85 – 1.70 (m, 2H), 1.56 – 1.47 (m, 3H), 1.43 – 1.34 (m, 4H), 1.32 – 1.22 (m, 7H), 1.22 (s, 4H), 1.16 – 1.12 (m, 2H), 1.11 – 1.02 (m, 4H), 0.91 (t, *J* = 7.6 Hz, 3H), 0.87 – 0.84 (m, 13H); **^{13}C NMR (100 MHz, CDCl$_3$)** δ 173.0, 171.7, 148.1, 147.7, 145.2, 140.3, 136.0, 135.9, 128.49, 128.47, 128.18, 128.15, 128.13, 128.10, 127.8, 125.7, 124.5, 122.8, 117.4, 117.0, 74.7, 72.1, 66.4, 66.1, 40.1, 40.0, 39.3, 37.55, 37.53, 37.46, 37.44, 37.41, 37.36, 37.34, 37.33, 37.29, 37.2, 33.8, 33.3, 32.7, 32.7, 32.7, 32.6, 31.3, 31.2, 29.3, 27.9, 24.8, 24.8, 24.4, 23.8, 23.3, 22.7, 22.6, 21.4, 21.01, 20.99, 20.6, 19.72, 19.65, 19.63, 19.59, 19.56,

13.2, 12.7, 11.9, 11.7; **HRMS (ESI) [M+Na]$^+$** Calcd for $C_{56}H_{80}O_6Na$: 871.58471 m/z, Found: 871.58235 m/z.

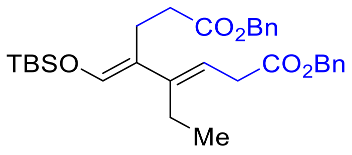

10k

Dibenzyl (3*E*,5*E*)-5-(((tert-butyldimethylsilyl)oxy)methylene)-4-ethyloct-3-enedioate (10k)

Colorless oil, 69% yield. **IR (neat):** 3034 (w), 2958 (w), 2931 (w), 2888 (w), 2857 (w), 1736 (s), 1630 (w), 1497 (w), 1457 (w), 1377 (w), 1329 (w), 1258 (s), 1154 (s), 1091 (m), 1016 (m), 938 (w), 837 (s), 793 (m), 748 (w), 697 (m), 674 (w), 583 (w), 501.22 (w); **^{1}H NMR (400 MHz, CDCl$_3$)** δ 7.41 – 7.27 (m, 10H), 6.38 (s, 1H), 5.42 (t, J = 7.2 Hz, 1H), 5.12 (s, 2H), 5.11 (s, 2H), 3.16 (d, J = 7.2 Hz, 2H), 2.65 – 2.54 (m, 2H), 2.40 – 2.32 (m, 2H), 2.13 (q, J = 7.6 Hz, 2H), 0.92 (s, 9H), 0.14 (s, 6H); **^{13}C NMR (100 MHz, CDCl$_3$)** δ 173.4, 172.0, 142.0, 137.6, 136.2, 135.9, 128.5, 128.5, 128.2, 128.1, 128.0, 128.0, 121.7, 114.9, 66.4, 66.0, 33.6, 33.0, 25.6, 21.2, 20.7, 18.2, 13.3, -5.3; **HRMS (ESI) [M+Na]$^+$** Calcd for $C_{31}H_{42}O_5NaSi$: 545.26937 m/z, Found: 545.26891 m/z.

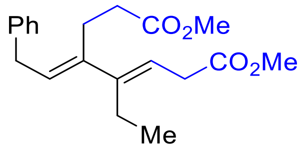

11a

Dimethyl (3*E*,5*E*)-4-ethyl-5-(2-phenylethylidene)oct-3-enedioate (11a)

Colorless oil, 66% yield. **IR (neat):** 3901 (w), 2956 (w),1737 (s), 1602 (w), 1494 (w), 1436 (w), 1329 (w), 1258 (w), 1195 (w), 1167 (m), 1058 (w), 1022 (w), 989 (w), 838 (w), 795 (w), 744 (w), 700 (m) cm^{-1}; **^{1}H NMR (400 MHz, CDCl$_3$)** δ 7.31 – 7.26 (m, 2H), 7.22 – 7.18 (m, 3H), 5.68 (t, J = 7.4 Hz, 1H), 5.57 (t, J = 7.2 Hz, 1H), 3.69 (s, 3H), 3.66 (s, 3H), 3.50 (d, J = 7.6 Hz, 2H), 3.15 (d, J = 7.2 Hz, 2H), 2.66 (t, J = 8.2 Hz, 2H), 2.43 – 2.32 (m, 2H), 2.21 (q, J = 7.6 Hz, 2H), 0.94 (t, J = 7.6 Hz, 3H); **^{13}C NMR (100 MHz, CDCl$_3$)** δ 173.6, 172.5, 144.9, 140.8, 139.2, 128.4, 128.3, 126.8, 125.9, 117.3, 51.8, 51.6, 34.3, 33.5, 33.1, 23.3, 21.4, 13.2; **HRMS (ESI) [M+Na]$^+$** Calcd for $C_{20}H_{26}O_4Na$: 353.17233 m/z, Found: 353.17254 m/z.

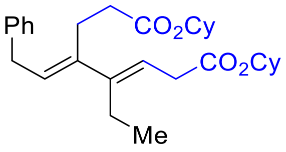

11b

Dicyclohexyl (3*E*,5*E*)-4-ethyl-5-(2-phenylethylidene)oct-3-enedioate (11b)

Colorless oil, 89% yield. **IR (neat):** 2935 (s), 2858 (w), 2362 (w), 1728 (s), 1451 (m), 1380 (w), 1357 (w), 1261 (m), 1177 (s), 1122 (w), 1038 (w), 1014 (m), 970 (w), 911 (w), 838 (w), 801 (w), 747 (w), 699 (m), 646 (w), 628 (w) cm^{-1}; **^{1}H NMR (400 MHz, $CDCl_3$)** δ 7.36 – 7.22 (m, 2H), 7.19 – 7.16 (m, 3H), 5.65 (t, *J* = 7.2 Hz, 1H), 5.55 (t, *J* = 7.2 Hz, 1H), 4.75 (s, 2H), 3.49 (d, *J* = 7.6 Hz, 2H), 3.10 (d, *J* = 7.2 Hz, 2H), 2.62 (t, *J* = 8.0 Hz, 2H), 2.32 (t, *J* = 8.4 Hz, 2H), 2.20 (q, *J* = 7.6 Hz, 2H), 1.83 – 1.81 (m, 4H), 1.70 (s, 3H), 1.52 (s, 2H), 1.45 – 1.31 (m, 8H), 1.24 (s, 3H), 0.92 (t, *J* = 7.6 Hz, 3H). **^{13}C NMR (100 MHz, $CDCl_3$)** δ 172.7, 171.5, 144.8, 141.0, 139.6, 128.4, 128.3, 126.5, 125.9, 117.7, 72.8, 72.6, 34.3, 33.7, 31.7, 31.6, 25.4, 25.3, 23.8, 23.7, 23.4, 21.4, 13.3; **HRMS (ESI) [M+Na]$^+$** Calcd for $C_{30}H_{42}O_4Na$: 489.29753 m/z, Found: 489.29795 m/z.

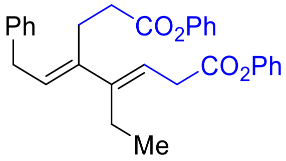

11c

Diphenyl (3*E*,5*E*)-4-ethyl-5-(2-phenylethylidene)oct-3-enedioate (11c)

Colorless oil, 52% yield. **IR (neat):** 3026 (w), 2966 (w), 2929 (w), 2320 (w), 2116 (w), 1753 (s), 1592 (w), 1491 (m), 1453 (w), 1357 (w), 1326 (w), 1230 (w), 1192 (s), 1161 (w), 1127 (s), 1069 (w), 1024 (w), 1005 (w), 927 (w), 897 (w), 813 (w), 745 (m), 690.16 (m), 497 (w); **^{1}H NMR (400 MHz, $CDCl_3$)** δ 7.40 – 7.35 (m, 4H), 7.33 – 7.27 (m, 2H), 7.26 – 7.20 (m, 5H), 7.12 – 7.05 (m, 4H), 5.79 (t, *J* = 7.2 Hz, 1H), 5.75 (t, *J* = 7.2 Hz, 1H), 3.57 (d, *J* = 7.6 Hz, 2H), 3.43 (d, *J* = 7.2 Hz, 2H), 2.85 – 2.76 (m, 2H), 2.72 – 2.62 (m, 2H), 2.34 (q, *J* = 7.6 Hz, 2H), 1.04 (t, *J* = 7.6 Hz, 3H); **^{13}C NMR (101 MHz, $CDCl_3$)** δ 171.6, 170.5, 150.7, 150.6, 145.7, 140.8, 139.0, 129.4, 129.4, 128.5, 128.3, 127.4, 126.0, 125.8, 125.7, 121.5, 121.5, 116.9, 34.4, 33.9, 33.3, 23.3, 21.6, 13.3; **HRMS (ESI) [M+Na]$^+$** Calcd for $C_{30}H_{30}O_4Na$: 477.20363 m/z, Found: 477.20494 m/z.

2.3 Proof of Stereochemistry: X-ray Characterization Data

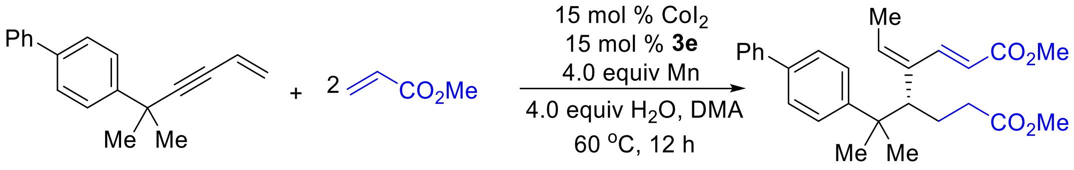

36
61%, 96:4 er

In a N_2-filled glove box, an oven-dried 8-mL vial equipped with a stirring bar was charged with CoI_2 (9.4 mg, 0.03 mmol, 15 mol%), **3e** (12.8 mg, 0.03 mmol, 15 mol%), Mn powder (44.0 mg, 0.8 mmol, 4 eq) and DMA (0.8 mL). The vial was sealed with a cap (phenolic cap with red PTFE/white silicone septum) and the solution was allowed to stir at room temperature for 30 min. 4-(2-methylhex-5-en-3-yn-2-yl)-1,1'-biphenyl (98.5 mg, 0.4 mmol, 2.0 equiv.), methyl acrylate (34.4 mg, 0.4 mmol, 1.0 equiv.), H_2O (14.4 mg, 0.8 mmol, 4.0 equiv.) and DMA (0.2 mL) were added to the solution. Then the vial was sealed with a cap (phenolic open top cap with red PTFE/white silicone septum), removed from the glove box. The mixture was immediately moved to a thermostatic bath and allowed to stir at 30 °C for 12 h. Upon cooling to room temperature, the reaction mixture was washed with brine (3×15 ml), eluted with Et_2O (20 mL), dried over $MgSO_4$, filtered and concentrated in *vacuo*. The residue was purified by silica gel column chromatography (PE:EA = 20:1) to afford the **36** as white solid (51.0 mg, 61% yield).

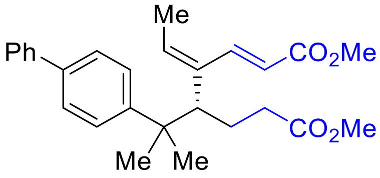

36

Dimethyl (*S*,2*E*,4*E*)-5-(2-([1,1'-biphenyl]-4-yl)propan-2-yl)-4-ethylideneoct-2-enedioate (36)

White solid, m.p. 79.6 - 81.0 °C, 61% yield. **IR (neat):** 3671 (w), 2970 (m), 2922 (w), 2361 (w), 1732 (m), 1712 (m), 1615 (w), 1487 (w), 1433 (w), 1381 (w), 1317 (w), 1277 (m), 1261 (w), 1229 (w), 1191 (m), 1168 (s), 1145 (w), 1076 (m), 1055 (m), 981 (w), 922 (w), 892 (w), 868 (w), 842 (w), 802 (w), 771 (m), 737 (w), 701 (m), 634 (w), 614 (w) cm^{-1}; **^{1}H NMR (400 MHz, $CDCl_3$)** δ 7.76 (d, J = 15.6 Hz, 1H), 7.63 – 7.57 (m, 2H), 7.56 – 7.50 (m, 2H), 7.47 – 7.41 (m, 2H), 7.41 – 7.37 (m, 2H), 7.37 – 7.30 (m, 1H), 5.93 (d, J = 15.6 Hz, 1H), 5.63 (q, J = 7.2 Hz, 1H), 3.75 (s, 3H),

3.58 (s, 3H), 2.69 (d, J = 12.0 Hz, 1H), 2.17 – 2.09 (m, 1H),, 2.06 – 1.94 (m, 1H), 1.90 (d, J = 7.2 Hz, 3H), 1.83 – 1.76 (m, 1H), 1.69 – 1.61 (m, 1H), 1.33 (s, 3H), 1.26 (s, 3H); **^{13}C NMR (100 MHz, CDCl$_3$)** δ 174.0, 167.9, 147.2, 143.2, 140.7, 138.6, 135.1, 133.9, 128.7, 127.1, 126.9, 126.5, 116.6, 51.6, 51.4, 41.4, 32.3, 28.5, 25.1, 24.0, 14.1; **HRMS (ESI) [M+Na]$^+$** Calcd for $C_{27}H_{32}O_4Na$: 443.21928 m/z, Found: 443.21894 m/z; **Specific rotation**: $[\alpha]_D^{29.3}$ -61.4 (*c* 1.00, CHCl$_3$)for an enantiomerically enriched sample of 96:4 e.r.

Enantiomeric purity of **36** was determined by HPLC analysis in comparison with authentic racemic material (96:4 e.r. shown; Chiralpak IBN-5 column, 95:5 hexane/ *i*-PrOH, 0.5 mL/min, 290 nm).

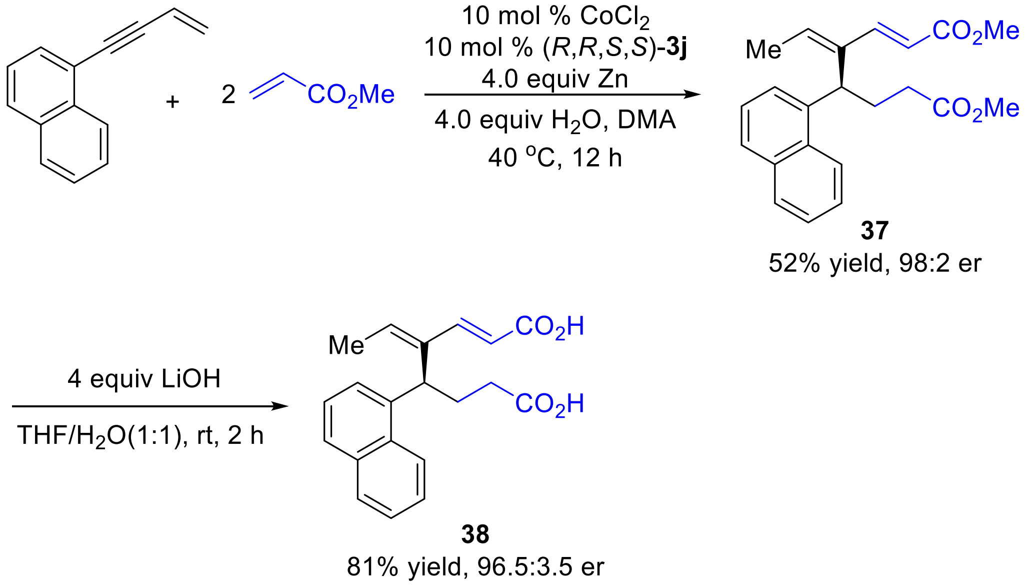

37 was prepared followed general procedure for preparing **6** by replacing **1c** and **2a** with 1-(but-3-en-1-yn-1-yl)naphthalene and methyl acrylate.

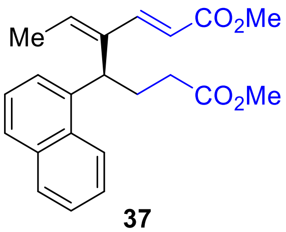

37

Dimethyl (*S*,2*E*,4*Z*)-4-ethylidene-5-(naphthalen-1-yl)oct-2-enedioate (37)

Pale yellow oil, 52% yield. **IR (neat):** 3661 (w), 2972 (w), 2904 (w), 1733 (m), 1717 (m), 1618 (w), 1509 (w), 1435 (w), 1395 (w), 1374 (w), 1309 (w), 1257 (w), 1193 (m), 1170 (s), 1075 (m), 1044 (m), 984 (w), 896 (w), 865 (w), 826 (w), 797 (m), 781 (m), 733 (w), 650 (w), 617 (w)

cm^{-1}; **^{1}H NMR (400 MHz, $CDCl_3$)** δ 8.01 – 7.88 (m, 2H), 7.82 (d, *J* = 8.0 Hz, 1H), 7.60 (d, *J* = 7.2 Hz, 1H), 7.56 – 7.51 (m, 3H), 7.31 (d, *J* = 8.0 Hz, 1H), 6.28 – 6.13 (m, 1H), 6.05 (d, *J* = 15.8 Hz, 1H), 4.75 – 4.64 (m, 1H), 3.76 (s, 3H), 3.74 (s, 3H), 2.65 – 2.52 (m, 3H), 2.43 – 2.33 (m, 1H), 1.93 (d, *J* = 7.2 Hz, 3H). **^{13}C NMR (100 MHz, $CDCl_3$)** δ 173.8, 167.6, 147.0, 138.9, 137.4, 134.0, 133.7, 132.1, 129.0, 127.6, 126.0, 125.6, 125.3, 124.5, 123.4, 116.7, 51.7, 51.5, 39.0, 31.9, 27.7, 14.9; **HRMS (ESI) [M+Na]$^+$** Calcd for $C_{22}H_{24}O_4Na$: 375.15668 m/z, Found: 375.15611 m/z; **Specific rotation**: $[\alpha]_D^{23.7}$ +73.3 (*c* 1.00, $CHCl_3$) for an enantiomerically enriched sample of 98:2 e.r.

Enantiomeric purity of **37** was determined by HPLC analysis in comparison with authentic racemic material (98:2 e.r. shown; Chiralpak IBN-5 column, 95:5 hexane/ *i*-PrOH, 1.0 mL/min, 254nm).

38 was prepared followed previous reported procedure[17]: To a 25 ml round bottle flask was added **37** (64.6 mg, 0.1833 mmol, 1.0 equiv), LiOH (17.6 mg, 0.7332 mmol, 4.0 equiv), THF (2 ml) and H_2O (2 ml). The mixture was allowed to stir at rt for 2h. Upon completion, the reaction mixture was diluted with H_2O (2 ml) and acidified with 1M HCl to pH = 1. The aqueous layer was extracted with EA (15 ml × 2),dried over $MgSO_4$, filtered, concentrated and purified by column chromatography (CH_2Cl_2:MeOH = 20:1) to give the product **38** as white solid (48.2 mg, 81% yield).

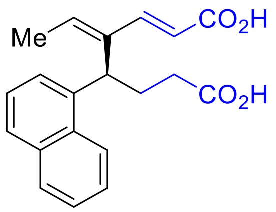

38

(*S*,2*E*,4*Z*)-4-ethylidene-5-(naphthalen-1-yl)oct-2-enedioic acid (38)

White solid, m.p. 100.2 – 101.2 °C, 81% yield. **IR (neat):** 2958 (w), 2922 (m), 2852 (w), 1692 (m), 1644 (w), 1604 (w), 1464 (w), 1423 (w), 1321 (w), 1260 (m), 1225 (w), 1091 (m), 1019 (m), 869 (w), 797 (s), 696 (w), 665 (w), 645 (w) cm^{-1}; **^{1}H NMR (400 MHz, THF-*d*$_8$)** δ 8.07 – 7.96 (m, 1H), 7.90 – 7.79 (m, 1H), 7.73 (d, *J* = 8.2 Hz, 1H), 7.58 (d, *J* = 7.2 Hz, 1H), 7.53 – 7.36 (m, 3H), 7.15 (d, *J* = 15.6 Hz, 1H), 6.15 (q, *J* = 7.2 Hz, 1H), 5.97 (d, *J* = 15.6 Hz, 1H), 4.76 (dd, *J* = 8.8, 6.0 Hz, 1H), 2.48 – 2.40 (m, 3H), 2.33 – 2.19 (m, 1H), 1.88 (d, *J* = 7.2 Hz, 3H); **^{13}C NMR (100 MHz, THF-*d*$_8$)** δ 174.4, 167.6, 146.7, 140.2, 138.8, 135.0, 133.2, 132.6, 129.4, 127.8, 126.3,

125.9, 125.8, 125.2, 124.3, 118.4, 39.7, 31.8, 28.5, 14.7; **HRMS (ESI) [M+Na]$^+$** Calcd for $C_{20}H_{20}O_4Na$: 347.12538 m/z, Found: 347.12491 m/z; **Specific rotation**: $[\alpha]_D^{28.4}$ +23.9 (*c* 1.00, $CHCl_3$) for an enantiomerically enriched sample of 96.5:3.5 e.r.

Enantiomeric purity of **38** was determined by SFC analysis in comparison with authentic racemic material (96.5:3.5 e.r. shown; Chiralpak AD-H column, 90:10 CO_2/ *i*-PrOH, 3.0 mL/min, 200 nm).

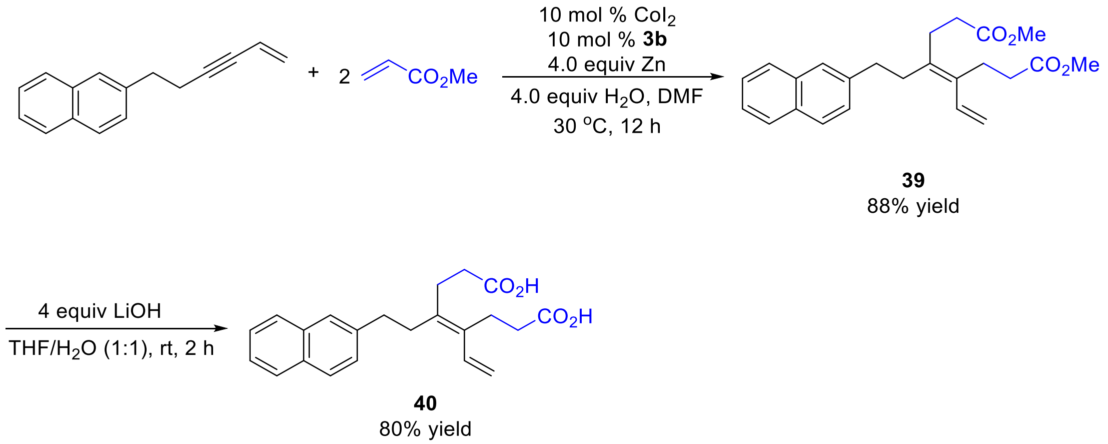

39 was prepared followed general procedure for preparing **8** by replacing **1b** and **2a** with 2-(hex-5-en-3-yn-1-yl)naphthalene and methyl acrylate.

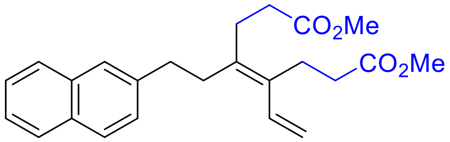

39

Dimethyl (*E*)-4-(2-(naphthalen-2-yl)ethyl)-5-vinyloct-4-enedioate (39)

Colorless oil, 88% yield. **IR (neat):** 3832 (w), 2954 (w), 1735 (s), 1627 (w), 1598 (w), 1508 (w), 1436 (w), 1363 (w), 1259 (m), 1194 (w), 1169 (w), 1069 (m), 1018 (w), 988 (w), 898 (w), 855 (w), 817 (m), 747 (m), 648 (w), 615 (w) cm^{-1}; **^{1}H NMR (400 MHz, CDCl$_3$)** δ 7.82 – 7.77 (m, 3H), 7.62 (s, 1H), 7.49 – 7.41 (m, 2H), 7.34 (dd, *J* = 8.4, 1.8 Hz, 1H), 6.65 (dd, *J* = 17.6, 11.2 Hz, 1H), 5.22 (d, *J* = 17.6 Hz, 1H), 5.08 (d, *J* = 11.2 Hz, 1H), 3.70 (s, 6H), 2.90 – 2.79 (m, 2H), 2.63 (t, *J* = 5.4 Hz, 2H), 2.57 – 2.47 (m, 4H), 2.46 – 2.40 (m, 4H). **^{13}C NMR (100 MHz, CDCl$_3$)** δ 173.6, 173.3, 139.0, 137.9, 133.5, 133.1, 132.1, 132.0, 127.9, 127.6, 127.3, 127.1, 126.3, 125.9, 125.2, 113.2, 51.7, 51.6, 35.6, 33.5, 33.5, 33.1, 28.4, 22.8; **HRMS (ESI) [M+Na]$^+$** Calcd for $C_{24}H_{28}O_4Na$: 403.18798 m/z, Found: 403.18818 m/z.

40 was prepared followed previous reported procedure[17]: To a 25 ml round bottle flask was added **39** (67.3 mg, 0.1769 mmol, 1.0 equiv), LiOH (16.9 mg, 0.7075 mmol, 4.0 equiv), THF (2 ml) and H_2O (2 ml). The mixture was allowed to stir at rt for 2 h. Upon completion, the reaction mixture was diluted with H_2O (2 ml) and acidified with 1M HCl to pH = 1. The aqueous layer was extracted with EA (15 ml × 2),dried over $MgSO_4$, filtered, concentrated and purified by column chromatography (CH_2Cl_2:MeOH = 20:1) to give the product **40** as white solid (49.9 mg, 80% yield).

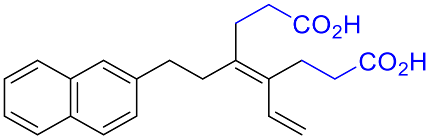

40

(*E*)-4-(2-(naphthalen-2-yl)ethyl)-5-vinyloct-4-enedioic acid (40)

White solid, m.p. 125.5-126.5 °C, 80% yield. **IR (neat):** 2961 (w), 2925 (w), 1705 (s), 1630 (w), 1600 (w), 1508 (w), 1413 (w), 1262 (w), 1212 (w), 1064 (w), 1018 (w), 988 (w), 904 (w), 854 (w), 816 (m), 747 (m), 662 (w), 621 (w) cm^{-1}; **^{1}H NMR (400 MHz, THF-*d*$_8$)** δ 7.83 – 7.70 (m, 3H), 7.65 (s, 1H), 7.42 – 7.34 (m, 3H), 6.73 (dd, *J* = 17.4, 11.2 Hz, 1H), 5.21 (dd, *J* = 17.4, 1.4 Hz, 1H), 5.01 (dd, *J* = 11.0, 1.2 Hz, 1H), 2.89 – 2.79 (m, 2H), 2.65 – 2.48 (m, 6H), 2.39 (t, *J* = 8.0 Hz, 2H), 2.35 – 2.28 (m, 2H). **^{13}C NMR (100 MHz, THF-*d*$_8$)** δ 174.1, 174.0, 140.2, 139.4, 134.6, 134.4, 133.1, 133.0, 128.4, 128.1, 128.0, 127.8, 127.0, 126.3, 125.6, 112.7, 36.4, 34.3, 33.8, 33.3, 29.1, 23.6; **HRMS (ESI) [M+Na]$^+$** Calcd for $C_{22}H_{24}O_4Na$: 375.15668 m/z, Found: 375.15633 m/z.

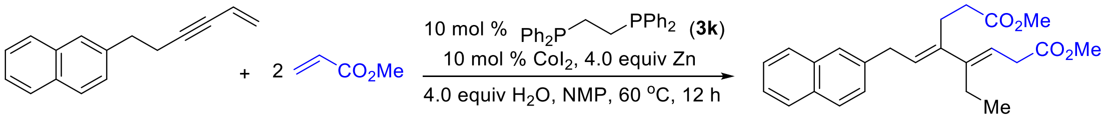

41
48% yield

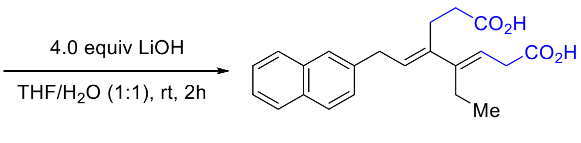

42
48% yield

41 was prepared followed general procedure for preparing **10** by replacing **1b** and **2a** with 2-(hex-5-en-3-yn-1-yl)naphthalene and methyl acrylate.

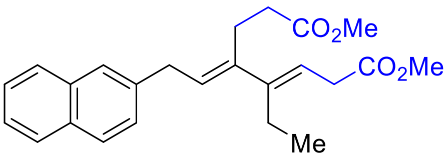

41

Dimethyl (3*E*,5*E*)-4-ethyl-5-(2-(naphthalen-2-yl)ethylidene)oct-3-enedioate (41)

Colorless oil, 48% yield. **IR (neat):** 3538 (w), 3467 (w), 2951 (w), 1735 (s), 1628 (w), 1601 (w), 1508 (w), 1437 (m), 1269 (m), 1197 (m), 1171 (m), 1018 (w), 987 (w), 894 (w), 858 (w), 818 (m), 750 (m), 654 (w), 631 (w), 616 (w) cm^{-1}; **^{1}H NMR (400 MHz, $CDCl_3$)** δ 7.82 – 7.76 (m, 3H), 7.63 – 7.59 (m, 1H), 7.48 – 7.40 (m, 2H), 7.33 (dd, J = 8.4, 1.6 Hz, 1H), 5.76 (t, J = 7.4 Hz, 1H), 5.60 (t, J = 7.2 Hz, 1H), 3.70 (s, 3H), 3.66 (d, J = 7.6 Hz, 5H), 3.16 (d, J = 7.2 Hz, 2H), 2.73 – 2.69 (m, 2H), 2.44 – 2.36 (m, 2H), 2.23 (q, J = 7.6 Hz, 2H), 0.96 (t, J = 7.6 Hz, 3H); **^{13}C NMR (100 MHz, $CDCl_3$)** δ 173.6, 172.4, 144.9, 139.5, 138.4, 133.6, 132.0, 128.0, 127.6, 127.4, 127.2, 126.7, 126.3, 125.9, 125.2, 117.4, 51.8, 51.6, 34.5, 33.6, 33.1, 23.4, 21.5, 13.2; **HRMS (ESI) $[M+Na]^+$** Calcd for $C_{24}H_{28}O_4Na$: 403.18798 m/z, Found: 403.18726 m/z.

42 was prepared followed previous reported procedure[17]: To a 25 ml round bottle flask was added **41** (36.2 mg, 0.0951 mmol, 1.0 equiv), LiOH (9.1 mg, 0.3806 mmol, 4.0 equiv), THF (1 ml) and H_2O (1 mL). The mixture was allowed to stir at rt for 2 h. Upon completion, the reaction mixture was diluted with H_2O (2 ml) and acidified with 1M HCl to pH = 1. The aqueous layer was extracted with EA (10 ml × 2),dried over $MgSO_4$, filtered, concentrated and purified by column chromatography (CH_2Cl_2:MeOH = 20:1) to give the product **42** as white solid (30.2 mg, 90% yield).

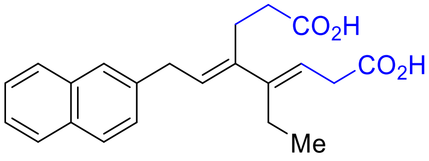

42

(3*E*,5*E*)-4-ethyl-5-(2-(naphthalen-2-yl)ethylidene)oct-3-enedioic acid (42)

White solid, m.p. 127.1-128.1 °C, 90% yield. **IR (neat):** 3048 (w), 2961 (w), 2926 (w), 1717 (w), 1696 (s), 1600 (w), 1508 (w), 1475 (w),1429 (w), 1382 (w), 1345 (w), 1294 (w), 1270 (w), 1238 (w), 1214 (s), 1168 (w), 1104 (w), 1056 (w), 1020 (w), 964 (w), 927 (m),859 (w), 816 (s), 758 (m), 699 (w), 667 (w), 648 (w), 626 (w) cm^{-1}; **^{1}H NMR (400 MHz, THF-d_8)** δ 10.00 (s, 2H),

7.77 – 7.73 (m, 3H), 7.64 (s, 1H), 7.43 – 7.28 (m, 3H), 5.73 (t, *J* = 7.4 Hz, 1H), 5.67 (t, *J* = 7.2 Hz, 1H), 3.66 (d, *J* = 7.2 Hz, 2H), 3.09 (d, *J* = 7.2 Hz, 2H), 2.70 (t, *J* = 8.0 Hz, 2H), 2.35 (t, *J* = 8.0 Hz, 2H), 2.29 – 2.13 (m, 2H), 0.94 (t, *J* = 7.6 Hz, 3H); **^{13}C NMR (100 MHz, THF-*d*$_8$)** δ 174.4, 173.2, 144.9, 140.9, 139.7, 134.8, 133.2, 128.7, 128.3, 128.2, 128.1, 127.1, 127.0, 126.6, 125.8, 119.6, 35.3, 33.8, 33.6, 24.2, 22.1, 13.7; **HRMS (ESI) [M+Na]$^+$** Calcd for $C_{22}H_{24}O_4Na$: 375.15668 m/z, Found: 375.15594 m/z.

The absolute configurations of 1,3-dienes **4**, **5** are determined by the X-ray crystallography of product **dimethyl (*S*,2*E*,4*E*)-5-(2-([1,1'-biphenyl]-4-yl)propan-2-yl)-4-ethylideneoct-2-enedioate (36)**, CCDC number: 2240339.

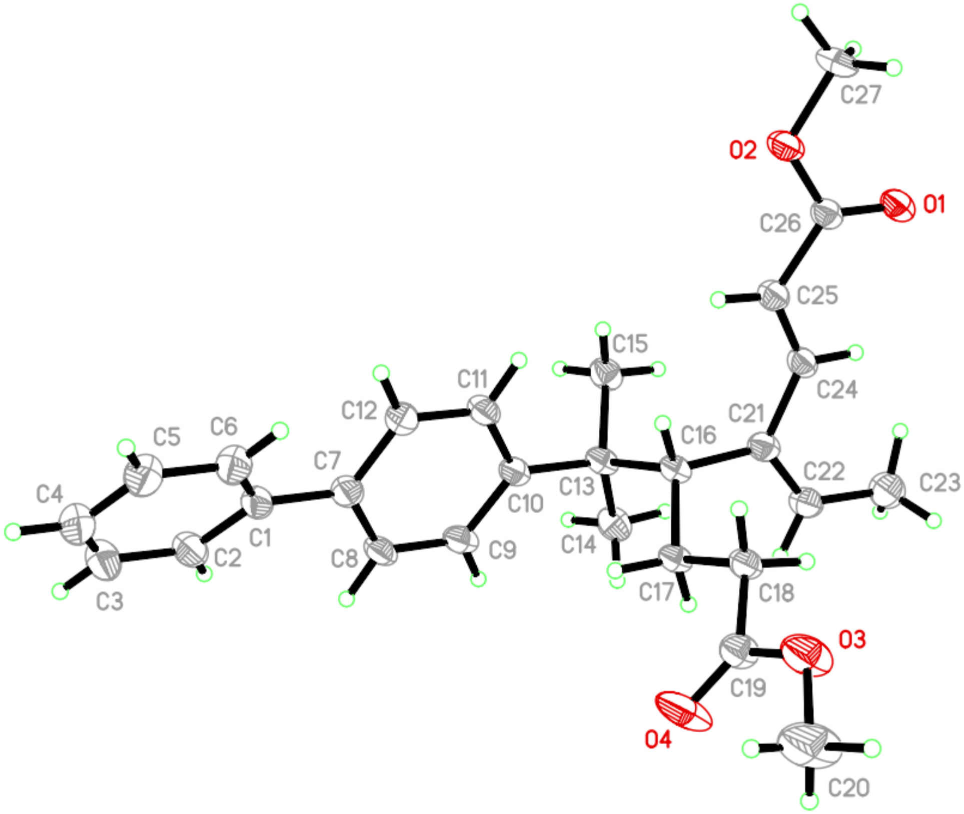

Supplementary Table 17. Crystal data and structure refinement for a_a.

Identification code	a_a
Empirical formula	C27 H32 O4

Formula weight	420.52
Temperature	173(2) K
Wavelength	1.54178 A
Crystal system, space group	Monoclinic, P2(1)
Unit cell dimensions	a = 9.4919(3) A alpha = 90 deg.
	b = 11.4771(3) A beta = 109.520(2) deg.
	c = 11.6927(3) A gamma = 90 deg.
Volume	1200.58(6) A^3
Z, Calculated density	2, 1.163 Mg/m^3
Absorption coefficient	0.610 mm^-1
F(000)	452
Crystal size	0.160 x 0.140 x 0.120 mm
Theta range for data collection	4.011 to 68.328 deg.
Limiting indices	-11<=h<=11, -13<=k<=13, -14<=l<=14
Reflections collected / unique	13965 / 4374 [R(int) = 0.0446]
Completeness to theta = 67.679	100.0 %
Absorption correction	Semi-empirical from equivalents

Max. and min. transmission	0.7531 and 0.6291
Refinement method	Full-matrix least-squares on F^2
Data / restraints / parameters	4374 / 1 / 285
Goodness-of-fit on F^2	1.027
Final R indices [I>2sigma(I)]	R1 = 0.0373, wR2 = 0.0971
R indices (all data)	R1 = 0.0429, wR2 = 0.1023
Absolute structure parameter	-0.04(12)
Extinction coefficient	n/a
Largest diff. peak and hole	0.155 and -0.156 e.A^-3

Supplementary Table 18. Atomic coordinates (x 10^4) and equivalent isotropic displacement parameters (A^2 x 10^3) for a_a.
U(eq) is defined as one third of the trace of the orthogonalized
Uij tensor.

	x	y	z	U(eq)
C(6)	5792(4)	7862(3)	9501(3)	45(1)
C(7)	7391(3)	7864(2)	8168(2)	36(1)
C(8)	8022(3)	8536(2)	7477(3)	36(1)
C(9)	8422(3)	8056(2)	6538(3)	35(1)
C(10)	8182(3)	6884(2)	6222(2)	30(1)
C(11)	7575(3)	6210(2)	6931(2)	34(1)
C(12)	7189(3)	6678(2)	7872(2)	36(1)
C(13)	8544(3)	6338(2)	5157(2)	31(1)
C(14)	9413(3)	7185(3)	4622(3)	38(1)
C(15)	9553(3)	5269(2)	5624(3)	36(1)
C(16)	7034(3)	5934(2)	4172(2)	28(1)
C(17)	5927(3)	6942(2)	3687(2)	30(1)
C(18)	4425(3)	6509(2)	2844(2)	33(1)
C(19)	3242(3)	7435(3)	2510(3)	37(1)
C(20)	734(4)	7859(4)	1367(5)	79(1)
C(21)	7278(3)	5224(2)	3148(2)	31(1)
C(22)	7608(3)	5736(2)	2239(2)	36(1)
C(23)	7933(4)	5175(3)	1197(3)	49(1)
C(24)	7106(3)	3956(2)	3148(2)	31(1)
C(25)	6714(3)	3276(2)	3920(2)	32(1)

C(26)	6622(3)	2008(2)	3708(2)	30(1)
C(27)	6244(3)	174(2)	4438(3)	41(1)
O(1)	6791(2)	1525(2)	2847(2)	41(1)
C(1)	6932(3)	8363(2)	9159(2)	38(1)
O(2)	6352(2)	1424(2)	4609(2)	37(1)
C(2)	7627(4)	9362(3)	9793(3)	46(1)
O(3)	1944(2)	7018(2)	1776(3)	63(1)
C(3)	7197(5)	9817(3)	10721(3)	58(1)
O(4)	3392(3)	8421(2)	2841(3)	67(1)
C(4)	6072(5)	9307(3)	11040(3)	59(1)
C(5)	5368(4)	8327(3)	10425(3)	54(1)

Supplementary Table 19. Bond lengths [A] and angles [deg] for a_a.

C(6)-C(5)	1.381(4)
C(6)-C(1)	1.397(5)
C(7)-C(8)	1.389(4)
C(7)-C(12)	1.402(4)
C(7)-C(1)	1.483(4)
C(8)-C(9)	1.390(4)
C(9)-C(10)	1.394(4)
C(10)-C(11)	1.391(4)
C(10)-C(13)	1.533(4)
C(11)-C(12)	1.379(4)
C(13)-C(14)	1.536(4)
C(13)-C(15)	1.540(4)
C(13)-C(16)	1.579(3)
C(16)-C(21)	1.528(3)
C(16)-C(17)	1.538(3)
C(17)-C(18)	1.521(3)
C(18)-C(19)	1.501(4)
C(19)-O(4)	1.189(4)
C(19)-O(3)	1.332(4)
C(20)-O(3)	1.453(4)
C(21)-C(22)	1.341(4)
C(21)-C(24)	1.463(4)
C(22)-C(23)	1.498(4)
C(24)-C(25)	1.337(4)
C(25)-C(26)	1.474(4)
C(26)-O(1)	1.206(3)
C(26)-O(2)	1.343(3)
C(27)-O(2)	1.448(3)

C(1)-C(2)	1.404(4)
C(2)-C(3)	1.383(5)
C(3)-C(4)	1.374(6)
C(4)-C(5)	1.381(5)
C(5)-C(6)-C(1)	121.4(3)
C(8)-C(7)-C(12)	116.4(2)
C(8)-C(7)-C(1)	122.5(2)
C(12)-C(7)-C(1)	121.1(3)
C(7)-C(8)-C(9)	121.6(2)
C(8)-C(9)-C(10)	121.9(3)
C(11)-C(10)-C(9)	116.2(2)
C(11)-C(10)-C(13)	120.5(2)
C(9)-C(10)-C(13)	123.3(2)
C(12)-C(11)-C(10)	122.1(2)
C(11)-C(12)-C(7)	121.7(3)
C(10)-C(13)-C(14)	111.6(2)
C(10)-C(13)-C(15)	108.6(2)
C(14)-C(13)-C(15)	107.1(2)
C(10)-C(13)-C(16)	108.51(19)
C(14)-C(13)-C(16)	111.7(2)
C(15)-C(13)-C(16)	109.3(2)
C(21)-C(16)-C(17)	112.1(2)
C(21)-C(16)-C(13)	112.94(19)
C(17)-C(16)-C(13)	112.96(19)
C(18)-C(17)-C(16)	111.7(2)
C(19)-C(18)-C(17)	113.3(2)
O(4)-C(19)-O(3)	122.4(3)
O(4)-C(19)-C(18)	126.3(3)
O(3)-C(19)-C(18)	111.3(2)
C(22)-C(21)-C(24)	119.4(2)

C(22)-C(21)-C(16)	121.6(2)
C(24)-C(21)-C(16)	119.0(2)
C(21)-C(22)-C(23)	128.5(3)
C(25)-C(24)-C(21)	129.5(2)
C(24)-C(25)-C(26)	118.5(2)
O(1)-C(26)-O(2)	122.6(2)
O(1)-C(26)-C(25)	125.3(2)
O(2)-C(26)-C(25)	112.1(2)
C(6)-C(1)-C(2)	117.3(3)
C(6)-C(1)-C(7)	121.5(3)
C(2)-C(1)-C(7)	121.3(3)
C(26)-O(2)-C(27)	114.1(2)
C(3)-C(2)-C(1)	120.8(3)
C(19)-O(3)-C(20)	115.6(3)
C(4)-C(3)-C(2)	120.8(3)
C(3)-C(4)-C(5)	119.4(3)
C(6)-C(5)-C(4)	120.4(4)

Symmetry transformations used to generate equivalent atoms:

Supplementary Table 20. Anisotropic displacement parameters (A^2 x 10^3) for a_a. The anisotropic displacement factor exponent takes the form:
-2 pi^2 [h^2 a*^2 U11 + ... + 2 h k a* b* U12]

	U11	U22	U33	U23	U13	U12
C(6)	53(2)	45(2)	35(1)	-1(1)	11(1)	7(1)
C(7)	37(1)	31(1)	32(1)	-2(1)	2(1)	5(1)
C(8)	41(1)	21(1)	40(1)	-5(1)	6(1)	0(1)
C(9)	37(1)	23(1)	43(1)	1(1)	10(1)	1(1)
C(10)	28(1)	24(1)	34(1)	-1(1)	5(1)	1(1)
C(11)	40(1)	23(1)	38(1)	-4(1)	10(1)	-2(1)
C(12)	43(1)	31(1)	34(1)	-1(1)	12(1)	-3(1)
C(13)	28(1)	23(1)	40(1)	-2(1)	10(1)	-1(1)
C(14)	34(1)	35(2)	48(2)	-5(1)	16(1)	-8(1)
C(15)	30(1)	32(1)	43(1)	-4(1)	7(1)	4(1)
C(16)	27(1)	22(1)	36(1)	-1(1)	10(1)	-1(1)
C(17)	30(1)	21(1)	38(1)	-1(1)	10(1)	-1(1)
C(18)	33(1)	24(1)	38(1)	-1(1)	6(1)	1(1)
C(19)	31(1)	32(1)	45(2)	-1(1)	8(1)	1(1)
C(20)	38(2)	56(2)	116(3)	-1(2)	-12(2)	15(2)
C(21)	29(1)	26(1)	36(1)	0(1)	8(1)	2(1)
C(22)	40(1)	28(1)	40(2)	1(1)	15(1)	1(1)
C(23)	64(2)	43(2)	44(2)	0(1)	27(2)	-2(2)
C(24)	32(1)	26(1)	33(1)	-4(1)	9(1)	3(1)
C(25)	34(1)	26(1)	34(1)	-2(1)	10(1)	2(1)
C(26)	28(1)	25(1)	35(1)	-1(1)	7(1)	1(1)

C(27)	50(2)	20(1)	58(2)	1(1)	23(1)	-1(1)
O(1)	57(1)	26(1)	42(1)	-5(1)	20(1)	1(1)
C(1)	47(1)	32(1)	30(1)	-2(1)	4(1)	9(1)
O(2)	48(1)	22(1)	44(1)	-1(1)	19(1)	0(1)
C(2)	54(2)	35(2)	41(2)	-6(1)	6(1)	9(1)
O(3)	38(1)	39(1)	87(2)	-9(1)	-12(1)	7(1)
C(3)	79(2)	44(2)	43(2)	-11(1)	10(2)	10(2)
O(4)	42(1)	27(1)	110(2)	-14(1)	-1(1)	7(1)
C(4)	86(3)	52(2)	39(2)	-2(2)	21(2)	20(2)
C(5)	70(2)	54(2)	42(2)	4(2)	22(2)	12(2)

Supplementary Table 21. Hydrogen coordinates (x 10^4) and isotropic displacement parameters (A^2 x 10^3) for a_a.

	x	y	z	U(eq)
H(6)	5298	7186	9089	54
H(8)	8184	9343	7651	43
H(9)	8873	8542	6099	42
H(11)	7420	5402	6761	41
H(12)	6777	6184	8331	43
H(14A)	9723	6782	4007	58
H(14B)	10299	7467	5270	58
H(14C)	8772	7847	4249	58
H(15A)	9848	4942	4963	55
H(15B)	9006	4681	5915	55
H(15C)	10449	5505	6291	55
H(16)	6547	5395	4598	34
H(17A)	5780	7361	4378	36
H(17B)	6353	7498	3243	36
H(18A)	4553	6202	2093	40
H(18B)	4086	5857	3242	40
H(20A)	-112	7506	733	119
H(20B)	1079	8547	1040	119
H(20C)	420	8090	2052	119
H(22)	7640	6563	2257	43
H(23A)	8804	5552	1083	73
H(23B)	7065	5262	457	73
H(23C)	8143	4346	1369	73
H(24)	7302	3559	2505	37
H(25)	6499	3603	4590	38

H(27A)	6150	-197	5164	62
H(27B)	7145	-115	4299	62
H(27C)	5365	-12	3734	62
H(2)	8401	9731	9581	56
H(3)	7686	10490	11144	70
H(4)	5782	9625	11678	71
H(5)	4587	7972	10639	65

Supplementary Table 22. Torsion angles [deg] for a_a.

C(12)-C(7)-C(8)-C(9)	0.4(4)
C(1)-C(7)-C(8)-C(9)	-178.9(2)
C(7)-C(8)-C(9)-C(10)	1.5(4)
C(8)-C(9)-C(10)-C(11)	-2.5(4)
C(8)-C(9)-C(10)-C(13)	177.2(2)
C(9)-C(10)-C(11)-C(12)	1.8(4)
C(13)-C(10)-C(11)-C(12)	-177.9(2)
C(10)-C(11)-C(12)-C(7)	-0.1(4)
C(8)-C(7)-C(12)-C(11)	-1.1(4)
C(1)-C(7)-C(12)-C(11)	178.2(2)
C(11)-C(10)-C(13)-C(14)	-171.5(2)
C(9)-C(10)-C(13)-C(14)	8.8(3)
C(11)-C(10)-C(13)-C(15)	-53.7(3)
C(9)-C(10)-C(13)-C(15)	126.6(3)
C(11)-C(10)-C(13)-C(16)	65.0(3)
C(9)-C(10)-C(13)-C(16)	-114.7(3)
C(10)-C(13)-C(16)-C(21)	-171.9(2)
C(14)-C(13)-C(16)-C(21)	64.6(3)
C(15)-C(13)-C(16)-C(21)	-53.6(3)
C(10)-C(13)-C(16)-C(17)	59.5(3)
C(14)-C(13)-C(16)-C(17)	-63.9(3)
C(15)-C(13)-C(16)-C(17)	177.8(2)
C(21)-C(16)-C(17)-C(18)	57.2(3)
C(13)-C(16)-C(17)-C(18)	-173.9(2)
C(16)-C(17)-C(18)-C(19)	170.8(2)
C(17)-C(18)-C(19)-O(4)	-0.1(4)
C(17)-C(18)-C(19)-O(3)	-179.7(3)

C(17)-C(16)-C(21)-C(22)	50.0(3)
C(13)-C(16)-C(21)-C(22)	-79.0(3)
C(17)-C(16)-C(21)-C(24)	-128.3(2)
C(13)-C(16)-C(21)-C(24)	102.7(3)
C(24)-C(21)-C(22)-C(23)	-4.1(4)
C(16)-C(21)-C(22)-C(23)	177.6(3)
C(22)-C(21)-C(24)-C(25)	-176.5(3)
C(16)-C(21)-C(24)-C(25)	1.9(4)
C(21)-C(24)-C(25)-C(26)	179.9(3)
C(24)-C(25)-C(26)-O(1)	-4.4(4)
C(24)-C(25)-C(26)-O(2)	174.3(2)
C(5)-C(6)-C(1)-C(2)	-0.1(4)
C(5)-C(6)-C(1)-C(7)	179.9(3)
C(8)-C(7)-C(1)-C(6)	152.9(3)
C(12)-C(7)-C(1)-C(6)	-26.3(4)
C(8)-C(7)-C(1)-C(2)	-27.0(4)
C(12)-C(7)-C(1)-C(2)	153.8(3)
O(1)-C(26)-O(2)-C(27)	-1.6(3)
C(25)-C(26)-O(2)-C(27)	179.7(2)
C(6)-C(1)-C(2)-C(3)	0.5(4)
C(7)-C(1)-C(2)-C(3)	-179.6(3)
O(4)-C(19)-O(3)-C(20)	2.3(5)
C(18)-C(19)-O(3)-C(20)	-178.1(3)
C(1)-C(2)-C(3)-C(4)	-0.5(5)
C(2)-C(3)-C(4)-C(5)	0.1(5)
C(1)-C(6)-C(5)-C(4)	-0.2(5)
C(3)-C(4)-C(5)-C(6)	0.2(5)

Symmetry transformations used to generate equivalent atoms:

Supplementary Table 23. Hydrogen bonds for a_a [A and deg.].

D-H...A	d(D-H)	d(H...A)	d(D...A)	<(DHA)

The absolute configurations of 1,3-dienes **6, 7** are determined by the X-ray crystallography of product **(*S*,2*E*,4*Z*)-4-ethylidene-5-(naphthalen-1-yl)oct-2-enedioic acid (38)**, CCDC number: 2250748.

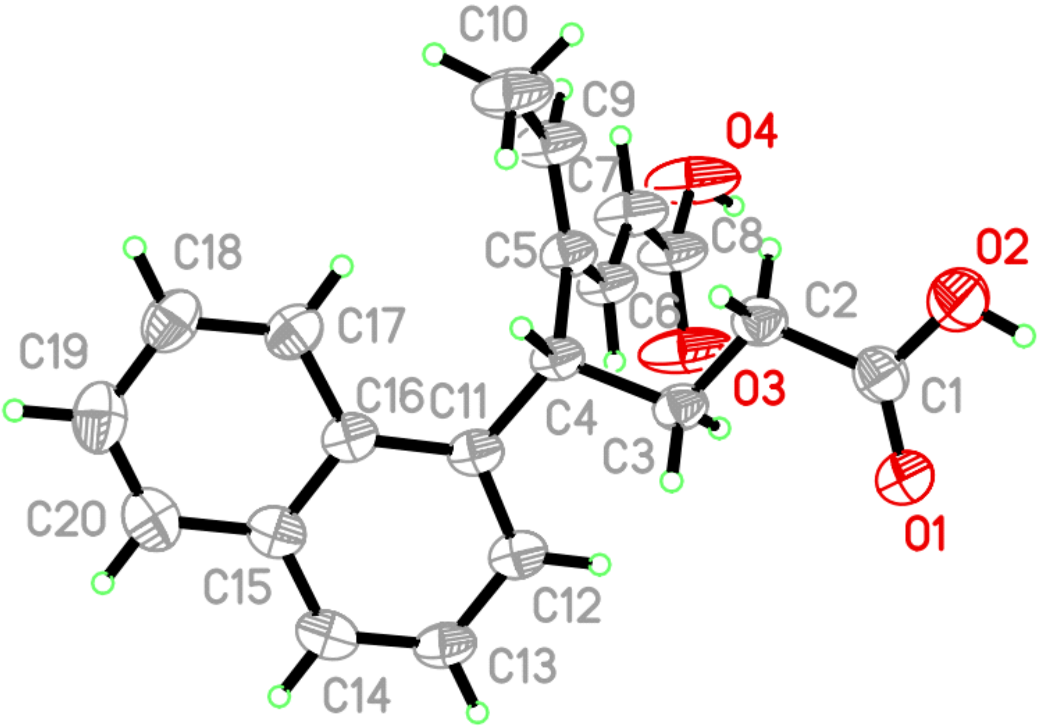

Supplementary Table 24. Crystal data and structure refinement for a.

Identification code	a
Empirical formula	C20 H20 O4
Formula weight	324.36
Temperature	173(2) K
Wavelength	1.54178 A
Crystal system, space group	Monoclinic, P2(1)

Unit cell dimensions a = 15.7730(4) A alpha = 90 deg.
b = 7.7076(2) A beta = 96.0590(10) deg.
c = 19.1694(5) A gamma = 90 deg.

Volume 2317.44(10) A^3

Z, Calculated density 4, 0.930 Mg/m^3

Absorption coefficient 0.522 mm^-1

F(000) 688

Crystal size 0.160 x 0.140 x 0.120 mm

Theta range for data collection 5.680 to 68.245 deg.

Limiting indices -18<=h<=19, -8<=k<=9, -22<=l<=23

Reflections collected / unique 44860 / 8383 [R(int) = 0.0540]

Completeness to theta = 67.679 99.7 %

Absorption correction Semi-empirical from equivalents

Max. and min. transmission 0.7531 and 0.5445

Refinement method Full-matrix least-squares on F^2

Data / restraints / parameters 8383 / 1 / 439

Goodness-of-fit on F^2 0.994

Final R indices [I>2sigma(I)] R1 = 0.0437, wR2 = 0.1265

R indices (all data) R1 = 0.0481, wR2 = 0.1320

Absolute structure parameter -0.15(9)

Extinction coefficient n/a

Largest diff. peak and hole 0.156 and -0.222 e.A^-3

Supplementary Table 25. Atomic coordinates (x 10^4) and equivalent isotropic displacement parameters (A^2 x 10^3) for a.
U(eq) is defined as one third of the trace of the orthogonalized
Uij tensor.

	x	y	z	U(eq)
C(1)	8302(2)	6471(4)	6683(1)	39(1)
C(4)	7542(1)	7148(3)	8527(1)	29(1)
O(5)	6948(1)	6850(2)	2591(1)	43(1)
C(5)	6603(1)	7605(3)	8330(1)	33(1)
O(6)	5869(1)	5235(3)	2129(2)	86(1)
C(6)	6023(1)	6111(3)	8252(1)	35(1)
O(7)	8908(2)	5430(3)	5118(1)	60(1)
C(7)	5196(2)	6123(4)	8031(2)	47(1)
O(8)	9063(2)	2995(3)	5740(1)	65(1)
C(8)	4697(2)	4530(3)	7935(2)	44(1)
C(9)	6321(2)	9219(3)	8201(2)	43(1)
C(10)	6817(2)	10869(4)	8229(2)	52(1)
O(1)	8633(2)	5058(3)	6747(1)	56(1)
C(11)	7703(1)	6101(3)	9203(1)	30(1)
C(12)	7912(1)	4377(3)	9195(1)	35(1)
C(13)	8139(2)	3422(3)	9816(1)	39(1)
C(14)	8166(2)	4216(4)	10452(1)	41(1)
C(15)	7949(1)	5986(3)	10498(1)	36(1)
C(16)	7697(1)	6943(3)	9872(1)	32(1)
C(17)	7461(2)	8706(3)	9946(1)	38(1)

C(18)	7482(2)	9480(4)	10594(1)	46(1)
C(19)	7749(2)	8527(4)	11205(1)	49(1)
C(20)	7980(2)	6841(4)	11155(1)	46(1)
C(21)	9054(2)	3888(4)	5147(1)	42(1)
C(22)	9227(2)	2791(4)	4535(1)	46(1)
C(23)	9520(2)	3871(3)	3935(1)	36(1)
C(24)	9439(1)	2892(3)	3227(1)	30(1)
C(25)	8504(1)	2400(3)	3030(1)	30(1)
C(26)	7935(1)	3863(3)	2834(1)	32(1)
C(27)	7132(2)	3815(4)	2541(2)	49(1)
C(28)	6631(2)	5406(3)	2424(2)	45(1)
C(29)	8200(2)	785(3)	3083(1)	37(1)
C(30)	8684(2)	-827(3)	3300(2)	46(1)
C(31)	9805(1)	3940(3)	2648(1)	29(1)
C(32)	10073(1)	5625(3)	2756(1)	35(1)
C(33)	10425(2)	6607(3)	2232(1)	41(1)
C(34)	10519(2)	5877(3)	1598(1)	38(1)
C(35)	10265(1)	4142(3)	1456(1)	32(1)
C(36)	9899(1)	3145(3)	1978(1)	29(1)
C(37)	9661(1)	1412(3)	1814(1)	31(1)
C(38)	9786(1)	688(3)	1173(1)	36(1)
C(39)	10161(1)	1664(4)	669(1)	37(1)
C(40)	10389(1)	3347(3)	807(1)	34(1)
O(2)	8274(2)	7368(3)	6099(1)	60(1)
C(2)	7921(2)	7403(3)	7266(1)	36(1)
O(3)	5024(1)	3082(3)	8066(2)	72(1)
C(3)	7913(1)	6255(3)	7908(1)	33(1)
O(4)	3906(1)	4706(3)	7710(2)	71(1)

Supplementary Table 26. Bond lengths [A] and angles [deg] for a.

C(1)-O(1)	1.209(4)
C(1)-O(2)	1.312(3)
C(1)-C(2)	1.508(3)
C(4)-C(11)	1.526(3)
C(4)-C(5)	1.531(3)
C(4)-C(3)	1.539(3)
O(5)-C(28)	1.247(3)
C(5)-C(9)	1.336(4)
C(5)-C(6)	1.468(3)
O(6)-C(28)	1.280(3)
C(6)-C(7)	1.328(3)
O(7)-C(21)	1.211(4)
C(7)-C(8)	1.460(4)
O(8)-C(21)	1.328(3)
C(8)-O(3)	1.244(3)
C(8)-O(4)	1.283(3)
C(9)-C(10)	1.492(4)
C(11)-C(12)	1.369(3)
C(11)-C(16)	1.439(3)
C(12)-C(13)	1.413(3)
C(13)-C(14)	1.361(4)
C(14)-C(15)	1.412(4)
C(15)-C(20)	1.418(4)
C(15)-C(16)	1.427(3)
C(16)-C(17)	1.420(4)
C(17)-C(18)	1.374(4)
C(18)-C(19)	1.410(4)

C(19)-C(20)	1.356(5)
C(21)-C(22)	1.495(4)
C(22)-C(23)	1.529(4)
C(23)-C(24)	1.547(3)
C(24)-C(25)	1.531(3)
C(24)-C(31)	1.533(3)
C(25)-C(29)	1.341(3)
C(25)-C(26)	1.466(3)
C(26)-C(27)	1.331(4)
C(27)-C(28)	1.463(4)
C(29)-C(30)	1.494(3)
C(31)-C(32)	1.375(3)
C(31)-C(36)	1.446(3)
C(32)-C(33)	1.418(4)
C(33)-C(34)	1.362(4)
C(34)-C(35)	1.414(3)
C(35)-C(40)	1.419(3)
C(35)-C(36)	1.430(3)
C(36)-C(37)	1.414(3)
C(37)-C(38)	1.383(3)
C(38)-C(39)	1.403(4)
C(39)-C(40)	1.364(4)
C(2)-C(3)	1.516(3)

O(1)-C(1)-O(2)	122.5(2)
O(1)-C(1)-C(2)	123.7(2)
O(2)-C(1)-C(2)	113.7(2)
C(11)-C(4)-C(5)	113.55(17)
C(11)-C(4)-C(3)	112.08(18)
C(5)-C(4)-C(3)	110.48(17)
C(9)-C(5)-C(6)	121.4(2)

C(9)-C(5)-C(4)	123.6(2)
C(6)-C(5)-C(4)	114.9(2)
C(7)-C(6)-C(5)	127.4(2)
C(6)-C(7)-C(8)	122.2(2)
O(3)-C(8)-O(4)	122.0(2)
O(3)-C(8)-C(7)	121.5(2)
O(4)-C(8)-C(7)	116.5(2)
C(5)-C(9)-C(10)	128.7(2)
C(12)-C(11)-C(16)	118.2(2)
C(12)-C(11)-C(4)	121.4(2)
C(16)-C(11)-C(4)	120.2(2)
C(11)-C(12)-C(13)	122.3(2)
C(14)-C(13)-C(12)	120.1(2)
C(13)-C(14)-C(15)	120.4(2)
C(14)-C(15)-C(20)	121.2(2)
C(14)-C(15)-C(16)	119.6(2)
C(20)-C(15)-C(16)	119.1(2)
C(17)-C(16)-C(15)	117.6(2)
C(17)-C(16)-C(11)	123.2(2)
C(15)-C(16)-C(11)	119.2(2)
C(18)-C(17)-C(16)	121.6(2)
C(17)-C(18)-C(19)	120.1(3)
C(20)-C(19)-C(18)	120.0(3)
C(19)-C(20)-C(15)	121.6(3)
O(7)-C(21)-O(8)	122.2(2)
O(7)-C(21)-C(22)	124.6(2)
O(8)-C(21)-C(22)	113.2(2)
C(21)-C(22)-C(23)	112.2(2)
C(22)-C(23)-C(24)	113.0(2)
C(25)-C(24)-C(31)	112.24(18)
C(25)-C(24)-C(23)	108.95(18)

C(31)-C(24)-C(23)	112.07(18)
C(29)-C(25)-C(26)	121.29(19)
C(29)-C(25)-C(24)	123.6(2)
C(26)-C(25)-C(24)	114.87(19)
C(27)-C(26)-C(25)	128.1(2)
C(26)-C(27)-C(28)	121.0(2)
O(5)-C(28)-O(6)	122.5(2)
O(5)-C(28)-C(27)	120.9(2)
O(6)-C(28)-C(27)	116.6(2)
C(25)-C(29)-C(30)	128.1(2)
C(32)-C(31)-C(36)	118.4(2)
C(32)-C(31)-C(24)	121.5(2)
C(36)-C(31)-C(24)	120.14(19)
C(31)-C(32)-C(33)	122.3(2)
C(34)-C(33)-C(32)	120.0(2)
C(33)-C(34)-C(35)	120.5(2)
C(34)-C(35)-C(40)	121.0(2)
C(34)-C(35)-C(36)	120.0(2)
C(40)-C(35)-C(36)	119.0(2)
C(37)-C(36)-C(35)	118.0(2)
C(37)-C(36)-C(31)	123.2(2)
C(35)-C(36)-C(31)	118.8(2)
C(38)-C(37)-C(36)	121.2(2)
C(37)-C(38)-C(39)	120.4(2)
C(40)-C(39)-C(38)	119.9(2)
C(39)-C(40)-C(35)	121.5(2)
C(1)-C(2)-C(3)	111.2(2)
C(2)-C(3)-C(4)	113.59(19)

Symmetry transformations used to generate equivalent atoms:

Supplementary Table 27. Anisotropic displacement parameters (A^2 x 10^3) for a.
The anisotropic displacement factor exponent takes the form:
-2 pi^2 [h^2 a*^2 U11 + ... + 2 h k a* b* U12]

	U11	U22	U33	U23	U13	U12
C(1)	36(1)	44(2)	38(1)	0(1)	-1(1)	-2(1)
C(4)	20(1)	26(1)	41(1)	-2(1)	-1(1)	-2(1)
O(5)	28(1)	28(1)	71(1)	0(1)	-2(1)	1(1)
C(5)	22(1)	31(1)	45(1)	-2(1)	0(1)	-1(1)
O(6)	35(1)	33(1)	181(3)	3(2)	-35(1)	3(1)
C(6)	24(1)	26(1)	55(1)	-2(1)	-1(1)	-1(1)
O(7)	88(2)	41(1)	54(1)	-2(1)	22(1)	6(1)
C(7)	28(1)	28(1)	83(2)	1(1)	-6(1)	-2(1)
O(8)	97(2)	57(1)	42(1)	8(1)	11(1)	29(1)
C(8)	22(1)	30(1)	79(2)	-2(1)	-8(1)	0(1)
C(9)	25(1)	29(1)	72(2)	-1(1)	-6(1)	-1(1)
C(10)	38(1)	28(1)	89(2)	3(1)	-5(1)	-1(1)
O(1)	72(1)	53(1)	44(1)	1(1)	8(1)	26(1)
C(11)	19(1)	28(1)	44(1)	-1(1)	1(1)	-4(1)
C(12)	27(1)	28(1)	50(1)	-1(1)	2(1)	-3(1)
C(13)	36(1)	26(1)	55(1)	4(1)	3(1)	2(1)
C(14)	34(1)	40(1)	47(1)	11(1)	0(1)	-1(1)
C(15)	26(1)	38(1)	44(1)	5(1)	2(1)	-6(1)
C(16)	24(1)	29(1)	44(1)	0(1)	4(1)	-4(1)
C(17)	38(1)	31(1)	47(1)	-2(1)	9(1)	-4(1)
C(18)	52(2)	35(1)	53(2)	-6(1)	15(1)	-5(1)

C(19)	58(2)	49(2)	42(1)	-7(1)	11(1)	-14(1)
C(20)	44(1)	51(2)	42(1)	5(1)	4(1)	-9(1)
C(21)	40(1)	45(2)	39(1)	-2(1)	-1(1)	3(1)
C(22)	56(2)	37(2)	44(1)	-1(1)	3(1)	3(1)
C(23)	34(1)	32(1)	41(1)	-4(1)	-1(1)	0(1)
C(24)	24(1)	25(1)	40(1)	-2(1)	-1(1)	2(1)
C(25)	22(1)	29(1)	38(1)	-1(1)	1(1)	3(1)
C(26)	27(1)	25(1)	45(1)	1(1)	5(1)	1(1)
C(27)	31(1)	26(1)	85(2)	2(1)	-9(1)	2(1)
C(28)	25(1)	30(1)	77(2)	4(1)	-4(1)	0(1)
C(29)	27(1)	33(1)	49(1)	2(1)	0(1)	1(1)
C(30)	40(1)	30(1)	67(2)	4(1)	-4(1)	1(1)
C(31)	19(1)	28(1)	40(1)	-1(1)	0(1)	3(1)
C(32)	29(1)	28(1)	47(1)	-6(1)	1(1)	1(1)
C(33)	35(1)	25(1)	62(2)	-2(1)	2(1)	-2(1)
C(34)	29(1)	34(1)	52(1)	4(1)	2(1)	-2(1)
C(35)	20(1)	30(1)	45(1)	1(1)	-1(1)	3(1)
C(36)	17(1)	27(1)	41(1)	-1(1)	-2(1)	4(1)
C(37)	25(1)	26(1)	43(1)	-2(1)	1(1)	2(1)
C(38)	29(1)	32(1)	47(1)	-8(1)	-2(1)	2(1)
C(39)	28(1)	42(1)	41(1)	-7(1)	-2(1)	6(1)
C(40)	24(1)	39(1)	38(1)	1(1)	0(1)	4(1)
O(2)	83(2)	49(1)	48(1)	5(1)	17(1)	13(1)
C(2)	31(1)	37(1)	41(1)	-1(1)	0(1)	3(1)
O(3)	31(1)	31(1)	148(2)	2(1)	-18(1)	-1(1)
C(3)	25(1)	31(1)	42(1)	-2(1)	1(1)	1(1)
O(4)	27(1)	34(1)	146(2)	6(1)	-23(1)	-6(1)

Supplementary Table 28. Hydrogen coordinates (x 10^4) and isotropic displacement parameters (A^2 x 10^3) for a.

	x	y	z	U(eq)
H(4)	7855	8271	8609	35
H(6)	5627	6207	2104	129
H(6A)	6267	5011	8373	42
H(7)	4921	7205	7932	57
H(8)	8911	3643	6057	97
H(9)	5725	9331	8074	52
H(10A)	6726	11455	7774	79
H(10B)	7425	10612	8339	79
H(10C)	6627	11625	8593	79
H(12)	7905	3803	8755	42
H(13)	8273	2224	9789	47
H(14)	8332	3576	10867	49
H(17)	7285	9366	9538	46
H(18)	7317	10660	10629	55
H(19)	7768	9066	11652	59
H(20)	8167	6216	11570	55
H(22A)	8702	2151	4362	55
H(22B)	9672	1928	4689	55
H(23A)	10123	4211	4058	43
H(23B)	9175	4945	3883	43
H(24)	9775	1793	3295	36
H(26)	8168	4986	2929	39

H(27)	6880	2730	2406	58
H(29)	7603	650	2969	44
H(30A)	8610	-1679	2919	70
H(30B)	8468	-1310	3720	70
H(30C)	9290	-549	3403	70
H(32)	10021	6150	3198	42
H(33)	10595	7775	2323	49
H(34)	10757	6537	1248	46
H(37)	9410	732	2151	38
H(38)	9617	-476	1073	43
H(39)	10256	1154	233	45
H(40)	10636	4001	460	41
H(2)	8515	6807	5800	89
H(2A)	7330	7760	7102	44
H(2B)	8256	8464	7394	44
H(3A)	8504	5881	8061	39
H(3B)	7573	5202	7777	39
H(4A)	3718	3766	7534	107

Supplementary Table 29. Torsion angles [deg] for a.

C(11)-C(4)-C(5)-C(9)	126.3(3)
C(3)-C(4)-C(5)-C(9)	-106.8(3)
C(11)-C(4)-C(5)-C(6)	-57.2(3)
C(3)-C(4)-C(5)-C(6)	69.7(3)
C(9)-C(5)-C(6)-C(7)	2.6(4)
C(4)-C(5)-C(6)-C(7)	-174.0(3)
C(5)-C(6)-C(7)-C(8)	176.2(3)
C(6)-C(7)-C(8)-O(3)	1.0(5)
C(6)-C(7)-C(8)-O(4)	-179.2(3)
C(6)-C(5)-C(9)-C(10)	-177.7(3)
C(4)-C(5)-C(9)-C(10)	-1.4(5)
C(5)-C(4)-C(11)-C(12)	107.8(2)
C(3)-C(4)-C(11)-C(12)	-18.2(3)
C(5)-C(4)-C(11)-C(16)	-76.6(2)
C(3)-C(4)-C(11)-C(16)	157.30(18)
C(16)-C(11)-C(12)-C(13)	-1.5(3)
C(4)-C(11)-C(12)-C(13)	174.1(2)
C(11)-C(12)-C(13)-C(14)	-0.9(4)
C(12)-C(13)-C(14)-C(15)	1.5(4)
C(13)-C(14)-C(15)-C(20)	-179.5(2)
C(13)-C(14)-C(15)-C(16)	0.2(3)
C(14)-C(15)-C(16)-C(17)	178.3(2)
C(20)-C(15)-C(16)-C(17)	-2.0(3)
C(14)-C(15)-C(16)-C(11)	-2.6(3)
C(20)-C(15)-C(16)-C(11)	177.1(2)
C(12)-C(11)-C(16)-C(17)	-177.7(2)
C(4)-C(11)-C(16)-C(17)	6.6(3)

C(12)-C(11)-C(16)-C(15)	3.2(3)
C(4)-C(11)-C(16)-C(15)	-172.51(18)
C(15)-C(16)-C(17)-C(18)	0.7(3)
C(11)-C(16)-C(17)-C(18)	-178.4(2)
C(16)-C(17)-C(18)-C(19)	0.5(4)
C(17)-C(18)-C(19)-C(20)	-0.4(4)
C(18)-C(19)-C(20)-C(15)	-0.9(4)
C(14)-C(15)-C(20)-C(19)	-178.1(3)
C(16)-C(15)-C(20)-C(19)	2.2(4)
O(7)-C(21)-C(22)-C(23)	20.8(4)
O(8)-C(21)-C(22)-C(23)	-160.0(2)
C(21)-C(22)-C(23)-C(24)	-162.4(2)
C(22)-C(23)-C(24)-C(25)	59.9(3)
C(22)-C(23)-C(24)-C(31)	-175.3(2)
C(31)-C(24)-C(25)-C(29)	130.3(2)
C(23)-C(24)-C(25)-C(29)	-104.9(3)
C(31)-C(24)-C(25)-C(26)	-54.9(3)
C(23)-C(24)-C(25)-C(26)	69.9(2)
C(29)-C(25)-C(26)-C(27)	-16.3(4)
C(24)-C(25)-C(26)-C(27)	168.8(3)
C(25)-C(26)-C(27)-C(28)	175.6(3)
C(26)-C(27)-C(28)-O(5)	0.7(5)
C(26)-C(27)-C(28)-O(6)	178.9(3)
C(26)-C(25)-C(29)-C(30)	-177.6(3)
C(24)-C(25)-C(29)-C(30)	-3.1(4)
C(25)-C(24)-C(31)-C(32)	116.0(2)
C(23)-C(24)-C(31)-C(32)	-6.9(3)
C(25)-C(24)-C(31)-C(36)	-65.9(2)
C(23)-C(24)-C(31)-C(36)	171.12(18)
C(36)-C(31)-C(32)-C(33)	0.9(3)
C(24)-C(31)-C(32)-C(33)	179.0(2)

C(31)-C(32)-C(33)-C(34)	-1.0(4)
C(32)-C(33)-C(34)-C(35)	0.2(3)
C(33)-C(34)-C(35)-C(40)	-177.7(2)
C(33)-C(34)-C(35)-C(36)	0.6(3)
C(34)-C(35)-C(36)-C(37)	-179.54(19)
C(40)-C(35)-C(36)-C(37)	-1.2(3)
C(34)-C(35)-C(36)-C(31)	-0.7(3)
C(40)-C(35)-C(36)-C(31)	177.61(18)
C(32)-C(31)-C(36)-C(37)	178.73(19)
C(24)-C(31)-C(36)-C(37)	0.6(3)
C(32)-C(31)-C(36)-C(35)	-0.1(3)
C(24)-C(31)-C(36)-C(35)	-178.18(17)
C(35)-C(36)-C(37)-C(38)	0.8(3)
C(31)-C(36)-C(37)-C(38)	-178.0(2)
C(36)-C(37)-C(38)-C(39)	0.5(3)
C(37)-C(38)-C(39)-C(40)	-1.3(3)
C(38)-C(39)-C(40)-C(35)	0.8(3)
C(34)-C(35)-C(40)-C(39)	178.8(2)
C(36)-C(35)-C(40)-C(39)	0.5(3)
O(1)-C(1)-C(2)-C(3)	-6.8(3)
O(2)-C(1)-C(2)-C(3)	175.8(2)
C(1)-C(2)-C(3)-C(4)	179.12(18)
C(11)-C(4)-C(3)-C(2)	-168.75(17)
C(5)-C(4)-C(3)-C(2)	63.5(2)

Symmetry transformations used to generate equivalent atoms:

Supplementary Table 30. Hydrogen bonds for a [A and deg.].

D-H...A	d(D-H)	d(H...A)	d(D...A)	<(DHA)

The configurations of 1,3-dienes **8, 9** are determined by the X-ray crystallography of product **(*E*)-4-(2-(naphthalen-2-yl)ethyl)-5-vinyloct-4-enedioic acid (40)**, CCDC number: 2251343

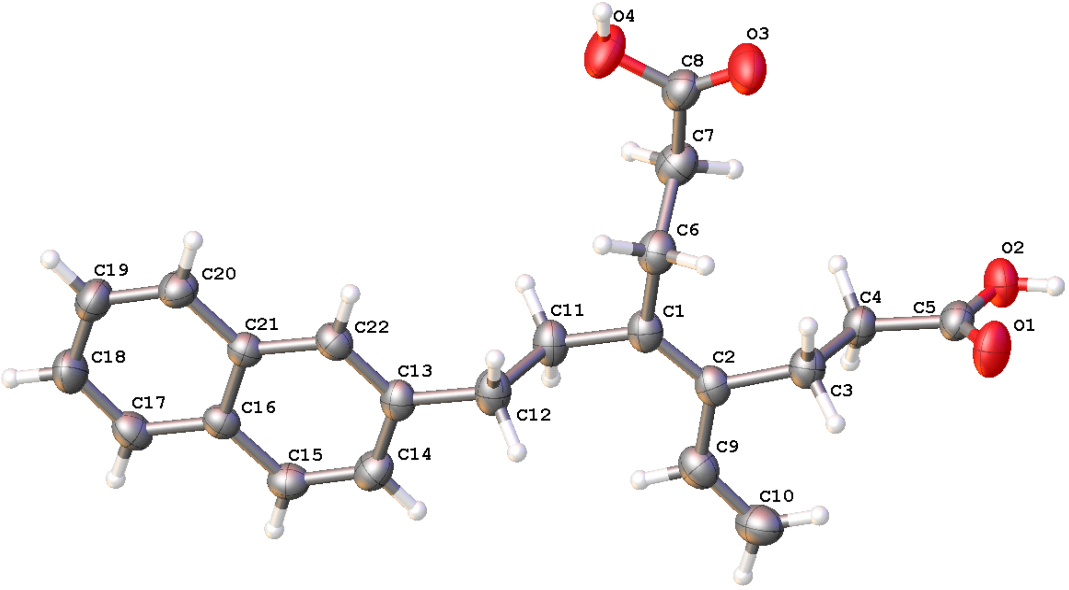

Supplementary Table 31. Crystal data and structure refinement for mj23174.

Identification code	mj23174	
Empirical formula	C22 H24 O4	
Formula weight	352.41	
Temperature	213.00 K	
Wavelength	1.34139 Å	
Crystal system	Monoclinic	
Space group	P 1 21/c 1	
Unit cell dimensions	a = 19.1510(6) Å	a= 90°.
	b = 6.0224(2) Å	b= 98.481(2)°.
	c = 16.6604(5) Å	g = 90°.
Volume	1900.51(10) Å3	
Z	4	
Density (calculated)	1.232 Mg/m3	
Absorption coefficient	0.430 mm-1	
F(000)	752	
Crystal size	0.07 x 0.07 x 0.05 mm3	

Theta range for data collection 4.669 to 55.010°.

Index ranges -20<=h<=23, -7<=k<=7, -20<=l<=20

Reflections collected 19259

Independent reflections 3599 [R(int) = 0.0474]

Completeness to theta = 53.594° 99.0 %

Absorption correction Semi-empirical from equivalents

Max. and min. transmission 0.7508 and 0.6203

Refinement method Full-matrix least-squares on F2

Data / restraints / parameters 3599 / 1 / 240

Goodness-of-fit on F2 0.940

Final R indices [I>2sigma(I)] R1 = 0.0458, wR2 = 0.1222

R indices (all 7data) R1 = 0.0759, wR2 = 0.1435

Extinction coefficient n/a

Largest diff. peak and hole 0.161 and -0.122 e.Å-3

Supplementary Table 32. Atomic coordinates (x 104) and equivalent isotropic displacement parameters (Å2x 103)

for mj23174. U(eq) is defined as one third of the trace of the orthogonalized Uij tensor.

	x	y	z	U(eq)
O(1)	4184(1)	9156(3)	8403(1)	89(1)
O(2)	5070(1)	6933(2)	8239(1)	79(1)
O(3)	4381(1)	13023(3)	5241(1)	95(1)
O(4)	3983(1)	11844(3)	4009(1)	97(1)
C(1)	2892(1)	7191(3)	5490(1)	58(1)
C(2)	2939(1)	6516(3)	6268(1)	56(1)
C(3)	3344(1)	7798(3)	6965(1)	61(1)
C(4)	4070(1)	6805(3)	7244(1)	58(1)
C(5)	4435(1)	7775(3)	8012(1)	60(1)
C(6)	3172(1)	9401(3)	5253(1)	67(1)
C(7)	3927(1)	9311(3)	5073(1)	73(1)
C(8)	4133(1)	11548(4)	4796(1)	70(1)
C(9)	2604(1)	4473(3)	6494(1)	68(1)
C(10)	2621(1)	3646(4)	7231(1)	85(1)
C(11)	2545(1)	5857(3)	4772(1)	66(1)
C(12)	1784(1)	6585(3)	4478(1)	68(1)
C(13)	1450(1)	5325(3)	3740(1)	56(1)
C(14)	1154(1)	3219(3)	3834(1)	64(1)
C(15)	837(1)	2032(3)	3187(1)	61(1)
C(16)	801(1)	2870(3)	2392(1)	51(1)
C(17)	474(1)	1712(3)	1702(1)	64(1)
C(18)	457(1)	2572(4)	948(1)	73(1)
C(19)	765(1)	4633(3)	842(1)	72(1)

C(20)	1082(1)	5795(3)	1486(1)	62(1)
C(21)	1107(1)	4972(3)	2282(1)	49(1)
C(22)	1426(1)	6151(3)	2976(1)	55(1)

Supplementary Table 33. Bond lengths [Å] and angles [°] for mj23174.

O(1)-C(5)	1.199(2)
O(2)-H(2)	0.8300
O(2)-C(5)	1.321(2)
O(3)-C(8)	1.208(2)
O(4)-C(8)	1.313(2)
O(4)-H(4)	0.880(18)
C(1)-C(2)	1.349(2)
C(1)-C(6)	1.509(3)
C(1)-C(11)	1.511(2)
C(2)-C(3)	1.509(2)
C(2)-C(9)	1.463(3)
C(3)-H(3A)	0.9800
C(3)-H(3B)	0.9800
C(3)-C(4)	1.523(2)
C(4)-H(4A)	0.9800
C(4)-H(4B)	0.9800
C(4)-C(5)	1.485(2)
C(6)-H(6A)	0.9800
C(6)-H(6B)	0.9800
C(6)-C(7)	1.521(3)
C(7)-H(7A)	0.9800
C(7)-H(7B)	0.9800
C(7)-C(8)	1.495(3)
C(9)-H(9)	0.9400
C(9)-C(10)	1.321(3)
C(10)-H(10A)	0.9400
C(10)-H(10B)	0.9400
C(11)-H(11A)	0.9800
C(11)-H(11B)	0.9800

C(11)-C(12)	1.531(2)
C(12)-H(12A)	0.9800
C(12)-H(12B)	0.9800
C(12)-C(13)	1.504(2)
C(13)-C(14)	1.407(3)
C(13)-C(22)	1.361(2)
C(14)-H(14)	0.9400
C(14)-C(15)	1.359(2)
C(15)-H(15)	0.9400
C(15)-C(16)	1.409(2)
C(16)-C(17)	1.410(2)
C(16)-C(21)	1.418(2)
C(17)-H(17)	0.9400
C(17)-C(18)	1.355(3)
C(18)-H(18)	0.9400
C(18)-C(19)	1.396(3)
C(19)-H(19)	0.9400
C(19)-C(20)	1.348(2)
C(20)-H(20)	0.9400
C(20)-C(21)	1.411(2)
C(21)-C(22)	1.417(2)
C(22)-H(22)	0.9400

C(5)-O(2)-H(2)	109.5
C(8)-O(4)-H(4)	117(2)
C(2)-C(1)-C(6)	122.74(16)
C(2)-C(1)-C(11)	124.09(17)
C(11)-C(1)-C(6)	113.16(15)
C(1)-C(2)-C(3)	122.65(16)
C(1)-C(2)-C(9)	122.07(16)
C(9)-C(2)-C(3)	115.28(15)

C(2)-C(3)-H(3A)	109.2
C(2)-C(3)-H(3B)	109.2
C(2)-C(3)-C(4)	112.07(14)
H(3A)-C(3)-H(3B)	107.9
C(4)-C(3)-H(3A)	109.2
C(4)-C(3)-H(3B)	109.2
C(3)-C(4)-H(4A)	108.8
C(3)-C(4)-H(4B)	108.8
H(4A)-C(4)-H(4B)	107.7
C(5)-C(4)-C(3)	113.63(15)
C(5)-C(4)-H(4A)	108.8
C(5)-C(4)-H(4B)	108.8
O(1)-C(5)-O(2)	122.41(16)
O(1)-C(5)-C(4)	124.50(17)
O(2)-C(5)-C(4)	113.08(16)
C(1)-C(6)-H(6A)	108.8
C(1)-C(6)-H(6B)	108.8
C(1)-C(6)-C(7)	113.91(15)
H(6A)-C(6)-H(6B)	107.7
C(7)-C(6)-H(6A)	108.8
C(7)-C(6)-H(6B)	108.8
C(6)-C(7)-H(7A)	109.8
C(6)-C(7)-H(7B)	109.8
H(7A)-C(7)-H(7B)	108.3
C(8)-C(7)-C(6)	109.30(15)
C(8)-C(7)-H(7A)	109.8
C(8)-C(7)-H(7B)	109.8
O(3)-C(8)-O(4)	121.37(19)
O(3)-C(8)-C(7)	124.84(17)
O(4)-C(8)-C(7)	113.68(19)
C(2)-C(9)-H(9)	116.4

C(10)-C(9)-C(2)	127.21(19)
C(10)-C(9)-H(9)	116.4
C(9)-C(10)-H(10A)	120.0
C(9)-C(10)-H(10B)	120.0
H(10A)-C(10)-H(10B)	120.0
C(1)-C(11)-H(11A)	109.0
C(1)-C(11)-H(11B)	109.0
C(1)-C(11)-C(12)	112.77(14)
H(11A)-C(11)-H(11B)	107.8
C(12)-C(11)-H(11A)	109.0
C(12)-C(11)-H(11B)	109.0
C(11)-C(12)-H(12A)	109.0
C(11)-C(12)-H(12B)	109.0
H(12A)-C(12)-H(12B)	107.8
C(13)-C(12)-C(11)	112.72(14)
C(13)-C(12)-H(12A)	109.0
C(13)-C(12)-H(12B)	109.0
C(14)-C(13)-C(12)	119.61(16)
C(22)-C(13)-C(12)	122.08(17)
C(22)-C(13)-C(14)	118.32(16)
C(13)-C(14)-H(14)	119.1
C(15)-C(14)-C(13)	121.78(16)
C(15)-C(14)-H(14)	119.1
C(14)-C(15)-H(15)	119.6
C(14)-C(15)-C(16)	120.72(16)
C(16)-C(15)-H(15)	119.6
C(15)-C(16)-C(17)	122.88(16)
C(15)-C(16)-C(21)	118.52(15)
C(17)-C(16)-C(21)	118.59(15)
C(16)-C(17)-H(17)	119.6
C(18)-C(17)-C(16)	120.87(17)

C(18)-C(17)-H(17)	119.6
C(17)-C(18)-H(18)	119.8
C(17)-C(18)-C(19)	120.36(17)
C(19)-C(18)-H(18)	119.8
C(18)-C(19)-H(19)	119.7
C(20)-C(19)-C(18)	120.62(17)
C(20)-C(19)-H(19)	119.7
C(19)-C(20)-H(20)	119.5
C(19)-C(20)-C(21)	121.01(17)
C(21)-C(20)-H(20)	119.5
C(20)-C(21)-C(16)	118.54(15)
C(20)-C(21)-C(22)	122.89(15)
C(22)-C(21)-C(16)	118.57(15)
C(13)-C(22)-C(21)	122.08(16)
C(13)-C(22)-H(22)	119.0
C(21)-C(22)-H(22)	119.0

Symmetry transformations used to generate equivalent atoms:

Supplementary Table 34. Anisotropic displacement parameters (Å2x 103) for mj23174. The anisotropic

displacement factor exponent takes the form: -2p2[h2 a*2U11 + ... + 2 h k a* b* U12]

	U11	U22	U33	U23	U13	U12
O(1)	109(1)	90(1)	64(1)	-25(1)	-1(1)	15(1)
O(2)	75(1)	91(1)	64(1)	-5(1)	-12(1)	1(1)
O(3)	111(1)	95(1)	69(1)	12(1)	-16(1)	-20(1)
O(4)	147(2)	84(1)	56(1)	9(1)	4(1)	-11(1)
C(1)	56(1)	56(1)	58(1)	-4(1)	0(1)	10(1)
C(2)	50(1)	53(1)	62(1)	-4(1)	1(1)	7(1)
C(3)	66(1)	58(1)	57(1)	-4(1)	4(1)	7(1)
C(4)	60(1)	65(1)	49(1)	-3(1)	4(1)	2(1)
C(5)	70(1)	60(1)	48(1)	4(1)	3(1)	-2(1)
C(6)	72(1)	64(1)	63(1)	0(1)	3(1)	10(1)
C(7)	79(1)	73(1)	67(1)	9(1)	11(1)	13(1)
C(8)	71(1)	79(1)	57(1)	9(1)	6(1)	4(1)
C(9)	59(1)	63(1)	80(1)	-1(1)	2(1)	3(1)
C(10)	72(1)	85(1)	98(2)	18(1)	14(1)	-7(1)
C(11)	68(1)	66(1)	60(1)	-8(1)	-3(1)	10(1)
C(12)	66(1)	74(1)	62(1)	-10(1)	-1(1)	15(1)
C(13)	53(1)	59(1)	56(1)	-2(1)	1(1)	11(1)
C(14)	71(1)	67(1)	56(1)	11(1)	9(1)	10(1)
C(15)	62(1)	51(1)	71(1)	9(1)	15(1)	0(1)
C(16)	45(1)	48(1)	61(1)	-2(1)	11(1)	1(1)
C(17)	59(1)	54(1)	78(1)	-10(1)	11(1)	-7(1)
C(18)	79(1)	79(1)	61(1)	-18(1)	7(1)	-10(1)
C(19)	86(1)	81(1)	51(1)	-3(1)	11(1)	-3(1)

C(20)	71(1)	58(1)	60(1)	4(1)	15(1)	-5(1)
C(21)	47(1)	47(1)	54(1)	0(1)	9(1)	3(1)
C(22)	54(1)	49(1)	63(1)	-1(1)	6(1)	-1(1)

Supplementary Table 35. Hydrogen coordinates (x 104) and isotropic displacement parameters (Å2x 10 3)

for mj23174.

	x	y	z	U(eq)
H(2)	5235	7432	8692	118
H(3A)	3073	7806	7420	73
H(3B)	3398	9340	6796	73
H(4A)	4020	5201	7317	70
H(4B)	4365	7029	6817	70
H(6A)	3148	10463	5694	81
H(6B)	2866	9955	4771	81
H(7A)	4246	8873	5563	88
H(7B)	3965	8206	4650	88
H(9)	2348	3658	6067	82
H(10A)	2870	4393	7680	102
H(10B)	2385	2311	7306	102
H(11A)	2547	4284	4922	79
H(11B)	2822	6015	4325	79
H(12A)	1502	6363	4917	82
H(12B)	1779	8174	4351	82
H(14)	1175	2617	4358	77
H(15)	640	638	3271	73
H(17)	263	325	1767	77
H(18)	238	1778	495	88
H(19)	751	5215	316	87
H(20)	1289	7176	1402	75

H(22)	1628	7549	2907	66
H(4)	4044(16)	13180(30)	3823(19)	152(12)

Supplementary Table 36. Torsion angles [°] for mj23174.

C(1)-C(2)-C(3)-C(4)	-100.22(19)
C(1)-C(2)-C(9)-C(10)	179.33(19)
C(1)-C(6)-C(7)-C(8)	176.49(16)
C(1)-C(11)-C(12)-C(13)	177.45(16)
C(2)-C(1)-C(6)-C(7)	92.1(2)
C(2)-C(1)-C(11)-C(12)	97.7(2)
C(2)-C(3)-C(4)-C(5)	-170.18(15)
C(3)-C(2)-C(9)-C(10)	-0.3(3)
C(3)-C(4)-C(5)-O(1)	2.4(3)
C(3)-C(4)-C(5)-O(2)	-178.69(15)
C(6)-C(1)-C(2)-C(3)	-6.6(3)
C(6)-C(1)-C(2)-C(9)	173.76(15)
C(6)-C(1)-C(11)-C(12)	-81.2(2)
C(6)-C(7)-C(8)-O(3)	87.3(3)
C(6)-C(7)-C(8)-O(4)	-89.0(2)
C(9)-C(2)-C(3)-C(4)	79.46(19)
C(11)-C(1)-C(2)-C(3)	174.61(15)
C(11)-C(1)-C(2)-C(9)	-5.0(3)
C(11)-C(1)-C(6)-C(7)	-89.01(19)
C(11)-C(12)-C(13)-C(14)	82.6(2)
C(11)-C(12)-C(13)-C(22)	-97.1(2)
C(12)-C(13)-C(14)-C(15)	179.00(16)
C(12)-C(13)-C(22)-C(21)	-179.52(15)
C(13)-C(14)-C(15)-C(16)	0.7(3)
C(14)-C(13)-C(22)-C(21)	0.8(2)
C(14)-C(15)-C(16)-C(17)	-179.45(16)
C(14)-C(15)-C(16)-C(21)	0.4(2)
C(15)-C(16)-C(17)-C(18)	-179.38(17)
C(15)-C(16)-C(21)-C(20)	178.98(15)

C(15)-C(16)-C(21)-C(22)	-0.9(2)
C(16)-C(17)-C(18)-C(19)	-0.2(3)
C(16)-C(21)-C(22)-C(13)	0.3(2)
C(17)-C(16)-C(21)-C(20)	-1.2(2)
C(17)-C(16)-C(21)-C(22)	178.97(14)
C(17)-C(18)-C(19)-C(20)	0.0(3)
C(18)-C(19)-C(20)-C(21)	-0.4(3)
C(19)-C(20)-C(21)-C(16)	1.0(3)
C(19)-C(20)-C(21)-C(22)	-179.15(17)
C(20)-C(21)-C(22)-C(13)	-179.56(15)
C(21)-C(16)-C(17)-C(18)	0.8(3)
C(22)-C(13)-C(14)-C(15)	-1.3(3)

Symmetry transformations used to generate equivalent atoms:

Supplementary Table 37. Hydrogen bonds for mj23174 [Å and °].

D-H...A	d(D-H)	d(H...A)	d(D...A)	<(DHA)

The configurations of 1,3-internal dienes **10, 11** are determined by the X-ray crystallography of product **(3*E*,5*E*)-4-ethyl-5-(2-(naphthalen-2-yl)ethylidene)oct-3-enedioic acid (42)**, CCDC number: 2240328

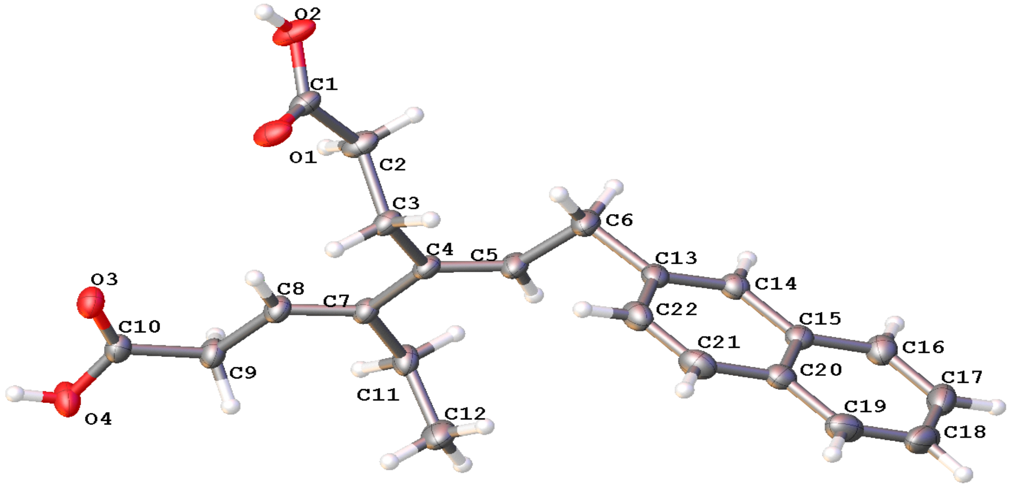

Supplementary Table 38. Crystal data and structure refinement for mj22415_0m.

Identification code	mj22415_0m
Empirical formula	C22 H24 O4
Formula weight	352.41
Temperature	216.00 K
Wavelength	1.34139 Å
Crystal system	Triclinic
Space group	P-1
Unit cell dimensions	a = 5.45890(10) Å a= 94.2480(10)°.
	b = 12.0283(2) Å b= 93.5610(10)°.
	c = 14.6334(2) Å g = 100.3810(10)°.
Volume	939.73(3) Å3
Z	2
Density (calculated)	1.245 Mg/m3
Absorption coefficient	0.435 mm-1
F(000)	376
Crystal size	0.07 x 0.07 x 0.05 mm3
Theta range for data collection	3.262 to 54.946°.

Index ranges -6<=h<=6, -14<=k<=14, -17<=l<=17

Reflections collected 11151

Independent reflections 3545 [R(int) = 0.0388]

Completeness to theta = 53.594° 99.1 %

Absorption correction Semi-empirical from equivalents

Max. and min. transmission 0.7508 and 0.6381

Refinement method Full-matrix least-squares on F2

Data / restraints / parameters 3545 / 0 / 238

Goodness-of-fit on F2 1.104

Final R indices [I>2sigma(I)] R1 = 0.0407, wR2 = 0.1092

R indices (all data) R1 = 0.0549, wR2 = 0.1175

Extinction coefficient n/a

Largest diff. peak and hole 0.168 and -0.226 e.Å-3

Supplementary Table 39. Atomic coordinates (x 104) and equivalent isotropic displacement parameters (Å2x 103)

for mj22415_0m. U(eq) is defined as one third of the trace of the orthogonalized Uij tensor.

	x	y	z	U(eq)
O(1)	3964(2)	9370(1)	898(1)	50(1)
O(2)	7137(2)	9019(1)	107(1)	56(1)
O(3)	-2180(2)	5662(1)	411(1)	45(1)
O(4)	-4220(2)	4008(1)	799(1)	56(1)
C(1)	5678(3)	8864(1)	783(1)	40(1)
C(2)	6317(3)	7999(1)	1390(1)	46(1)
C(3)	4964(3)	7945(1)	2266(1)	37(1)
C(4)	5272(3)	6912(1)	2765(1)	36(1)
C(5)	7180(3)	6958(1)	3398(1)	42(1)
C(6)	9248(3)	7942(1)	3714(1)	50(1)
C(7)	3444(3)	5841(1)	2520(1)	36(1)
C(8)	1628(3)	5753(1)	1847(1)	40(1)
C(9)	-312(3)	4710(1)	1579(1)	44(1)
C(10)	-2303(3)	4856(1)	876(1)	39(1)
C(11)	3620(3)	4832(1)	3067(1)	45(1)
C(12)	2431(4)	4901(2)	3973(1)	62(1)
C(13)	9266(3)	8249(1)	4738(1)	39(1)
C(14)	10902(3)	7908(1)	5350(1)	40(1)
C(15)	10885(3)	8168(1)	6312(1)	38(1)
C(16)	12530(3)	7808(1)	6959(1)	50(1)
C(17)	12448(4)	8073(2)	7878(1)	61(1)
C(18)	10699(4)	8702(2)	8195(1)	67(1)
C(19)	9108(3)	9072(2)	7589(1)	60(1)

C(20)	9150(3)	8813(1)	6634(1)	43(1)
C(21)	7484(3)	9163(1)	5981(1)	53(1)
C(22)	7536(3)	8884(1)	5070(1)	50(1)

Supplementary Table 40. Bond lengths [Å] and angles [°] for mj22415_0m.

O(1)-C(1)	1.2188(17)
O(2)-H(2)	0.8300
O(2)-C(1)	1.3114(17)
O(3)-C(10)	1.2190(17)
O(4)-H(4)	0.8300
O(4)-C(10)	1.3159(18)
C(1)-C(2)	1.4902(18)
C(2)-H(2A)	0.9800
C(2)-H(2B)	0.9800
C(2)-C(3)	1.5187(19)
C(3)-H(3A)	0.9800
C(3)-H(3B)	0.9800
C(3)-C(4)	1.5172(18)
C(4)-C(5)	1.3401(19)
C(4)-C(7)	1.486(2)
C(5)-H(5)	0.9400
C(5)-C(6)	1.504(2)
C(6)-H(6A)	0.9800
C(6)-H(6B)	0.9800
C(6)-C(13)	1.515(2)
C(7)-C(8)	1.337(2)
C(7)-C(11)	1.5172(19)
C(8)-H(8)	0.9400
C(8)-C(9)	1.498(2)
C(9)-H(9A)	0.9800
C(9)-H(9B)	0.9800
C(9)-C(10)	1.493(2)
C(11)-H(11A)	0.9800
C(11)-H(11B)	0.9800

C(11)-C(12)	1.515(2)
C(12)-H(12A)	0.9700
C(12)-H(12B)	0.9700
C(12)-H(12C)	0.9700
C(13)-C(14)	1.3624(19)
C(13)-C(22)	1.404(2)
C(14)-H(14)	0.9400
C(14)-C(15)	1.420(2)
C(15)-C(16)	1.409(2)
C(15)-C(20)	1.410(2)
C(16)-H(16)	0.9400
C(16)-C(17)	1.364(2)
C(17)-H(17)	0.9400
C(17)-C(18)	1.401(3)
C(18)-H(18)	0.9400
C(18)-C(19)	1.357(3)
C(19)-H(19)	0.9400
C(19)-C(20)	1.412(2)
C(20)-C(21)	1.416(2)
C(21)-H(21)	0.9400
C(21)-C(22)	1.354(2)
C(22)-H(22)	0.9400
C(1)-O(2)-H(2)	109.5
C(10)-O(4)-H(4)	109.5
O(1)-C(1)-O(2)	123.31(12)
O(1)-C(1)-C(2)	123.50(13)
O(2)-C(1)-C(2)	113.19(12)
C(1)-C(2)-H(2A)	108.7
C(1)-C(2)-H(2B)	108.7
C(1)-C(2)-C(3)	114.31(12)

H(2A)-C(2)-H(2B)	107.6
C(3)-C(2)-H(2A)	108.7
C(3)-C(2)-H(2B)	108.7
C(2)-C(3)-H(3A)	109.2
C(2)-C(3)-H(3B)	109.2
H(3A)-C(3)-H(3B)	107.9
C(4)-C(3)-C(2)	112.23(11)
C(4)-C(3)-H(3A)	109.2
C(4)-C(3)-H(3B)	109.2
C(5)-C(4)-C(3)	120.71(13)
C(5)-C(4)-C(7)	120.89(12)
C(7)-C(4)-C(3)	118.38(12)
C(4)-C(5)-H(5)	115.7
C(4)-C(5)-C(6)	128.66(13)
C(6)-C(5)-H(5)	115.7
C(5)-C(6)-H(6A)	109.4
C(5)-C(6)-H(6B)	109.4
C(5)-C(6)-C(13)	110.96(12)
H(6A)-C(6)-H(6B)	108.0
C(13)-C(6)-H(6A)	109.4
C(13)-C(6)-H(6B)	109.4
C(4)-C(7)-C(11)	118.61(12)
C(8)-C(7)-C(4)	121.92(12)
C(8)-C(7)-C(11)	119.45(13)
C(7)-C(8)-H(8)	117.7
C(7)-C(8)-C(9)	124.69(13)
C(9)-C(8)-H(8)	117.7
C(8)-C(9)-H(9A)	108.6
C(8)-C(9)-H(9B)	108.6
H(9A)-C(9)-H(9B)	107.5
C(10)-C(9)-C(8)	114.78(12)

C(10)-C(9)-H(9A)	108.6
C(10)-C(9)-H(9B)	108.6
O(3)-C(10)-O(4)	123.04(13)
O(3)-C(10)-C(9)	124.31(13)
O(4)-C(10)-C(9)	112.65(12)
C(7)-C(11)-H(11A)	109.0
C(7)-C(11)-H(11B)	109.0
H(11A)-C(11)-H(11B)	107.8
C(12)-C(11)-C(7)	112.86(13)
C(12)-C(11)-H(11A)	109.0
C(12)-C(11)-H(11B)	109.0
C(11)-C(12)-H(12A)	109.5
C(11)-C(12)-H(12B)	109.5
C(11)-C(12)-H(12C)	109.5
H(12A)-C(12)-H(12B)	109.5
H(12A)-C(12)-H(12C)	109.5
H(12B)-C(12)-H(12C)	109.5
C(14)-C(13)-C(6)	121.47(14)
C(14)-C(13)-C(22)	118.88(14)
C(22)-C(13)-C(6)	119.64(13)
C(13)-C(14)-H(14)	119.1
C(13)-C(14)-C(15)	121.73(14)
C(15)-C(14)-H(14)	119.1
C(16)-C(15)-C(14)	122.76(14)
C(16)-C(15)-C(20)	118.54(14)
C(20)-C(15)-C(14)	118.71(13)
C(15)-C(16)-H(16)	119.5
C(17)-C(16)-C(15)	120.92(17)
C(17)-C(16)-H(16)	119.5
C(16)-C(17)-H(17)	119.8
C(16)-C(17)-C(18)	120.33(17)

C(18)-C(17)-H(17)	119.8
C(17)-C(18)-H(18)	119.9
C(19)-C(18)-C(17)	120.17(16)
C(19)-C(18)-H(18)	119.9
C(18)-C(19)-H(19)	119.6
C(18)-C(19)-C(20)	120.83(17)
C(20)-C(19)-H(19)	119.6
C(15)-C(20)-C(19)	119.19(15)
C(15)-C(20)-C(21)	118.29(14)
C(19)-C(20)-C(21)	122.51(15)
C(20)-C(21)-H(21)	119.4
C(22)-C(21)-C(20)	121.22(15)
C(22)-C(21)-H(21)	119.4
C(13)-C(22)-H(22)	119.4
C(21)-C(22)-C(13)	121.16(14)
C(21)-C(22)-H(22)	119.4

Symmetry transformations used to generate equivalent atoms:

Supplementary Table 41. Anisotropic displacement parameters (Å2x 103) for mj22415_0m. The anisotropic

displacement factor exponent takes the form: -2p2[h2 a*2U11 + ... + 2 h k a* b* U12]

	U11	U22	U33	U23	U13	U12
O(1)	61(1)	58(1)	42(1)	24(1)	13(1)	26(1)
O(2)	77(1)	64(1)	41(1)	27(1)	22(1)	34(1)
O(3)	49(1)	44(1)	41(1)	6(1)	-12(1)	8(1)
O(4)	50(1)	51(1)	60(1)	13(1)	-22(1)	-1(1)
C(1)	54(1)	40(1)	28(1)	8(1)	4(1)	11(1)
C(2)	62(1)	44(1)	39(1)	17(1)	10(1)	21(1)
C(3)	49(1)	34(1)	28(1)	7(1)	0(1)	7(1)
C(4)	44(1)	37(1)	28(1)	10(1)	0(1)	6(1)
C(5)	47(1)	42(1)	35(1)	14(1)	-5(1)	-1(1)
C(6)	49(1)	54(1)	41(1)	15(1)	-5(1)	-8(1)
C(7)	41(1)	36(1)	30(1)	7(1)	-3(1)	8(1)
C(8)	46(1)	38(1)	34(1)	10(1)	-6(1)	5(1)
C(9)	48(1)	44(1)	38(1)	9(1)	-11(1)	5(1)
C(10)	43(1)	40(1)	34(1)	0(1)	-5(1)	8(1)
C(11)	48(1)	37(1)	45(1)	10(1)	-15(1)	2(1)
C(12)	68(1)	65(1)	50(1)	28(1)	-4(1)	-2(1)
C(13)	34(1)	36(1)	41(1)	10(1)	-2(1)	-6(1)
C(14)	37(1)	35(1)	45(1)	7(1)	0(1)	2(1)
C(15)	38(1)	34(1)	40(1)	7(1)	-1(1)	-4(1)
C(16)	51(1)	45(1)	52(1)	12(1)	-5(1)	2(1)
C(17)	62(1)	63(1)	50(1)	20(1)	-16(1)	-12(1)
C(18)	71(1)	78(1)	39(1)	-2(1)	4(1)	-20(1)
C(19)	55(1)	64(1)	56(1)	-7(1)	13(1)	-3(1)

C(20)	39(1)	40(1)	46(1)	5(1)	3(1)	-4(1)
C(21)	42(1)	50(1)	70(1)	8(1)	8(1)	11(1)
C(22)	39(1)	52(1)	60(1)	19(1)	-6(1)	5(1)

Supplementary Table 42. Hydrogen coordinates (x 104) and isotropic displacement parameters (Å2x 10 3)

for mj22415_0m.

	x	y	z	U(eq)
H(2)	6724	9514	-205	84
H(4)	-5262	4115	393	83
H(2A)	8120	8166	1555	55
H(2B)	5920	7252	1044	55
H(3A)	5613	8632	2675	45
H(3B)	3182	7926	2114	45
H(5)	7232	6286	3682	51
H(6A)	9024	8599	3381	60
H(6B)	10856	7744	3574	60
H(8)	1565	6391	1518	48
H(9A)	520	4104	1342	53
H(9B)	-1103	4464	2132	53
H(11A)	2798	4136	2698	54
H(11B)	5383	4785	3188	54
H(12A)	681	4942	3858	93
H(12B)	3277	5573	4351	93
H(12C)	2572	4232	4289	93
H(14)	12072	7489	5130	47
H(16)	13700	7378	6754	60
H(17)	13570	7833	8300	74
H(18)	10628	8868	8830	80
H(19)	7964	9506	7808	72

H(21)	6320	9596	6184	64
H(22)	6394	9119	4653	61

Supplementary Table 43. Torsion angles [°] for mj22415_0m.

O(1)-C(1)-C(2)-C(3)	-10.4(2)
O(2)-C(1)-C(2)-C(3)	170.05(13)
C(1)-C(2)-C(3)-C(4)	168.73(13)
C(2)-C(3)-C(4)-C(5)	89.97(16)
C(2)-C(3)-C(4)-C(7)	-88.58(16)
C(3)-C(4)-C(5)-C(6)	-0.7(2)
C(3)-C(4)-C(7)-C(8)	3.2(2)
C(3)-C(4)-C(7)-C(11)	-175.28(12)
C(4)-C(5)-C(6)-C(13)	121.12(17)
C(4)-C(7)-C(8)-C(9)	-178.40(14)
C(4)-C(7)-C(11)-C(12)	80.69(17)
C(5)-C(4)-C(7)-C(8)	-175.31(14)
C(5)-C(4)-C(7)-C(11)	6.2(2)
C(5)-C(6)-C(13)-C(14)	100.39(17)
C(5)-C(6)-C(13)-C(22)	-78.51(18)
C(6)-C(13)-C(14)-C(15)	-178.36(13)
C(6)-C(13)-C(22)-C(21)	179.31(14)
C(7)-C(4)-C(5)-C(6)	177.81(15)
C(7)-C(8)-C(9)-C(10)	173.79(14)
C(8)-C(7)-C(11)-C(12)	-97.86(17)
C(8)-C(9)-C(10)-O(3)	13.8(2)
C(8)-C(9)-C(10)-O(4)	-166.91(13)
C(11)-C(7)-C(8)-C(9)	0.1(2)
C(13)-C(14)-C(15)-C(16)	178.88(13)
C(13)-C(14)-C(15)-C(20)	-1.0(2)
C(14)-C(13)-C(22)-C(21)	0.4(2)
C(14)-C(15)-C(16)-C(17)	-179.78(15)
C(14)-C(15)-C(20)-C(19)	179.66(14)
C(14)-C(15)-C(20)-C(21)	0.6(2)

C(15)-C(16)-C(17)-C(18)	0.7(3)
C(15)-C(20)-C(21)-C(22)	0.3(2)
C(16)-C(15)-C(20)-C(19)	-0.3(2)
C(16)-C(15)-C(20)-C(21)	-179.32(14)
C(16)-C(17)-C(18)-C(19)	-1.4(3)
C(17)-C(18)-C(19)-C(20)	1.3(3)
C(18)-C(19)-C(20)-C(15)	-0.5(2)
C(18)-C(19)-C(20)-C(21)	178.57(16)
C(19)-C(20)-C(21)-C(22)	-178.74(15)
C(20)-C(15)-C(16)-C(17)	0.1(2)
C(20)-C(21)-C(22)-C(13)	-0.8(2)
C(22)-C(13)-C(14)-C(15)	0.5(2)

Symmetry transformations used to generate equivalent atoms:

Supplementary Table 44. Hydrogen bonds for mj22415_0m [Å and °].

D-H...A	d(D-H)	d(H...A)	d(D...A)	<(DHA)

2.4 Copies of NMR Spectra

Supplementary Figure 5. ^{1}H NMR and ^{13}C NMR spectrum of compound of **4a**

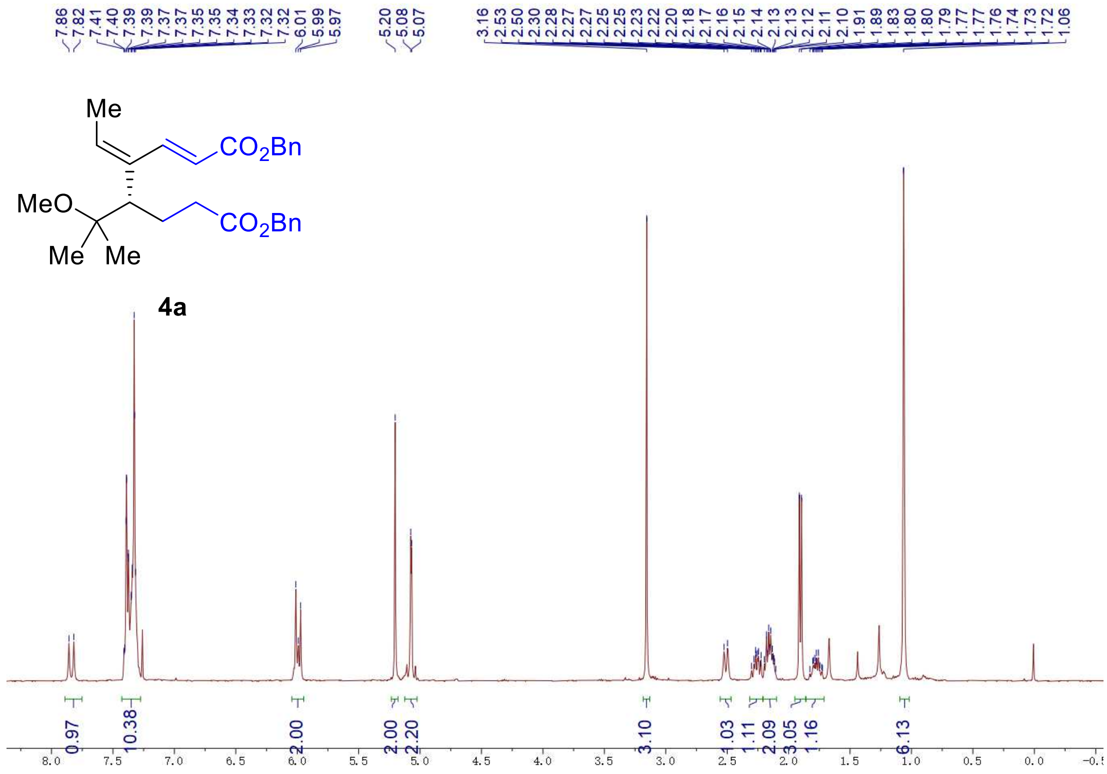

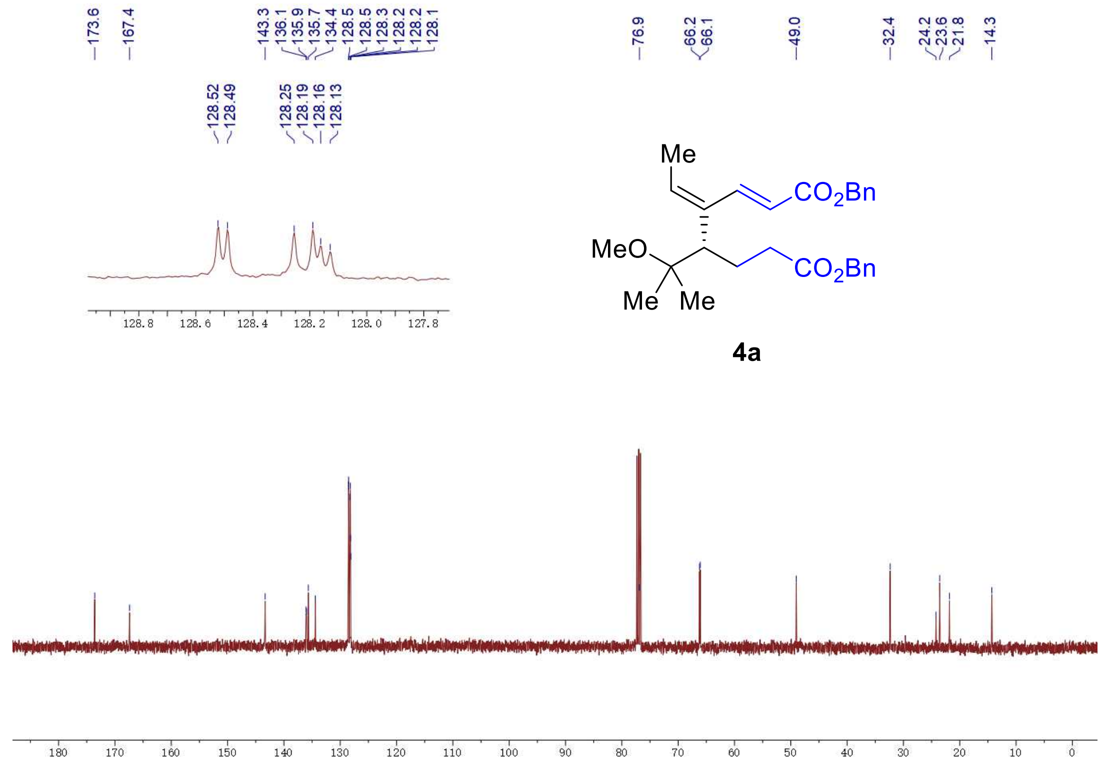

Supplementary Figure 6. ^{1}H NMR and ^{13}C NMR spectrum of compound of **4b**

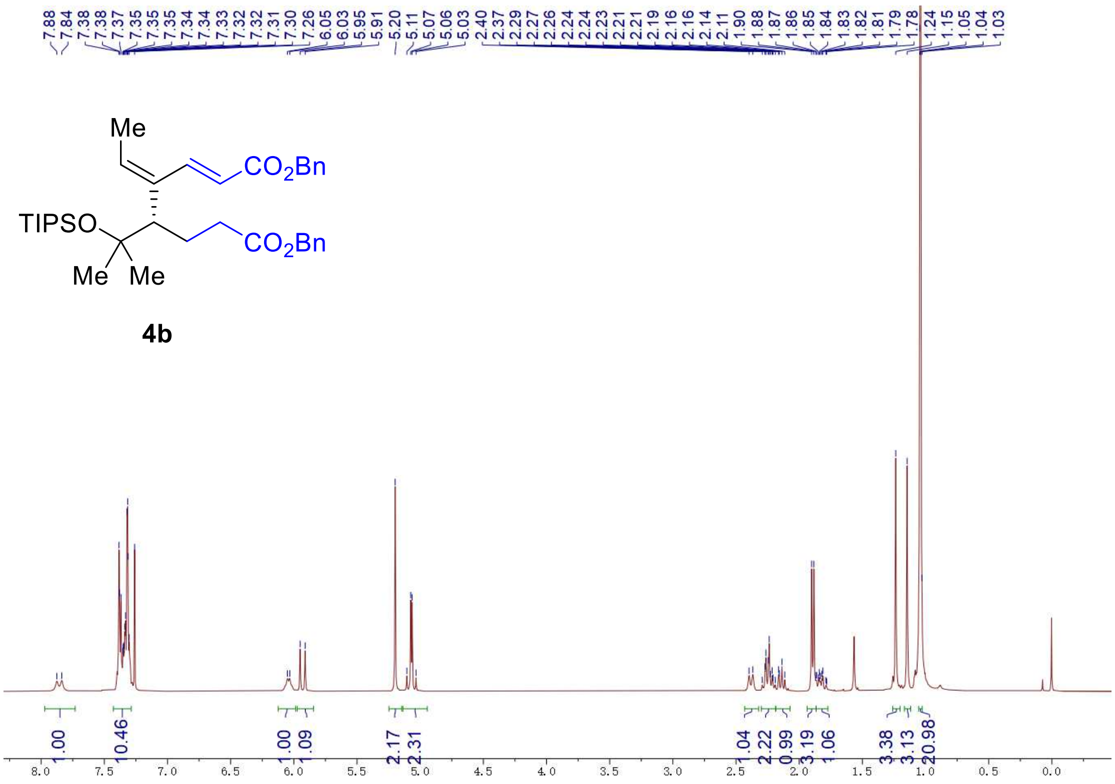

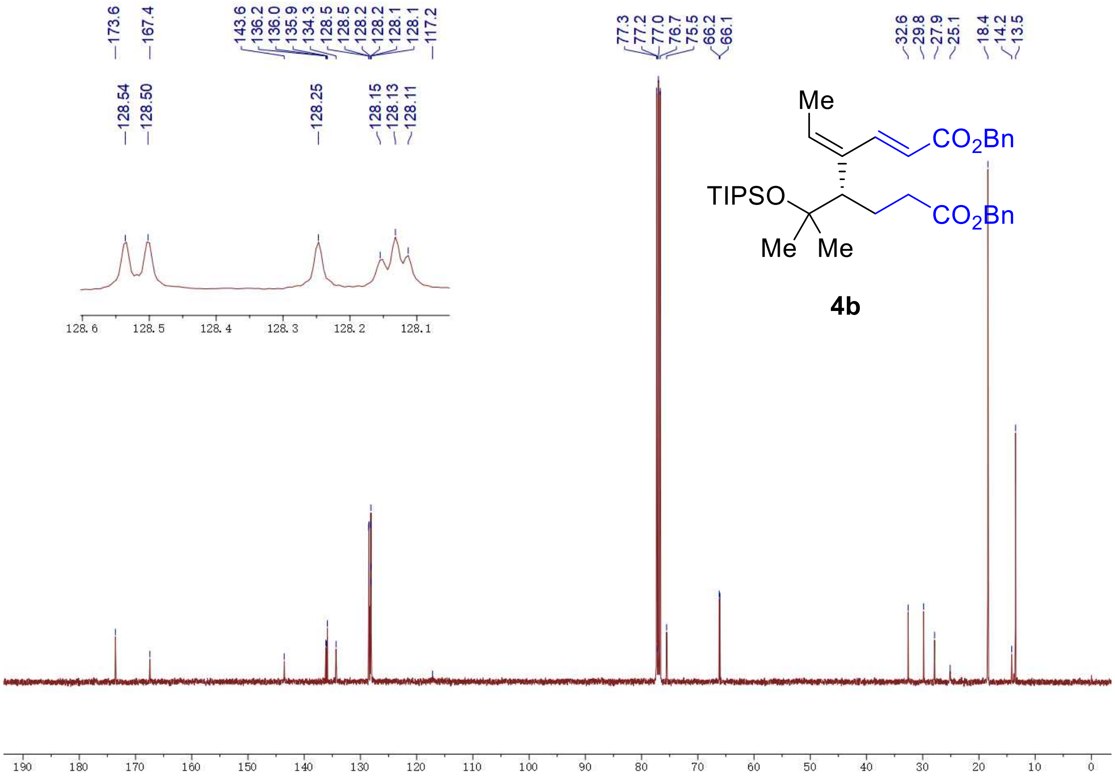

Supplementary Figure 7. ^{1}H NMR and ^{13}C NMR spectrum of compound of **4c**

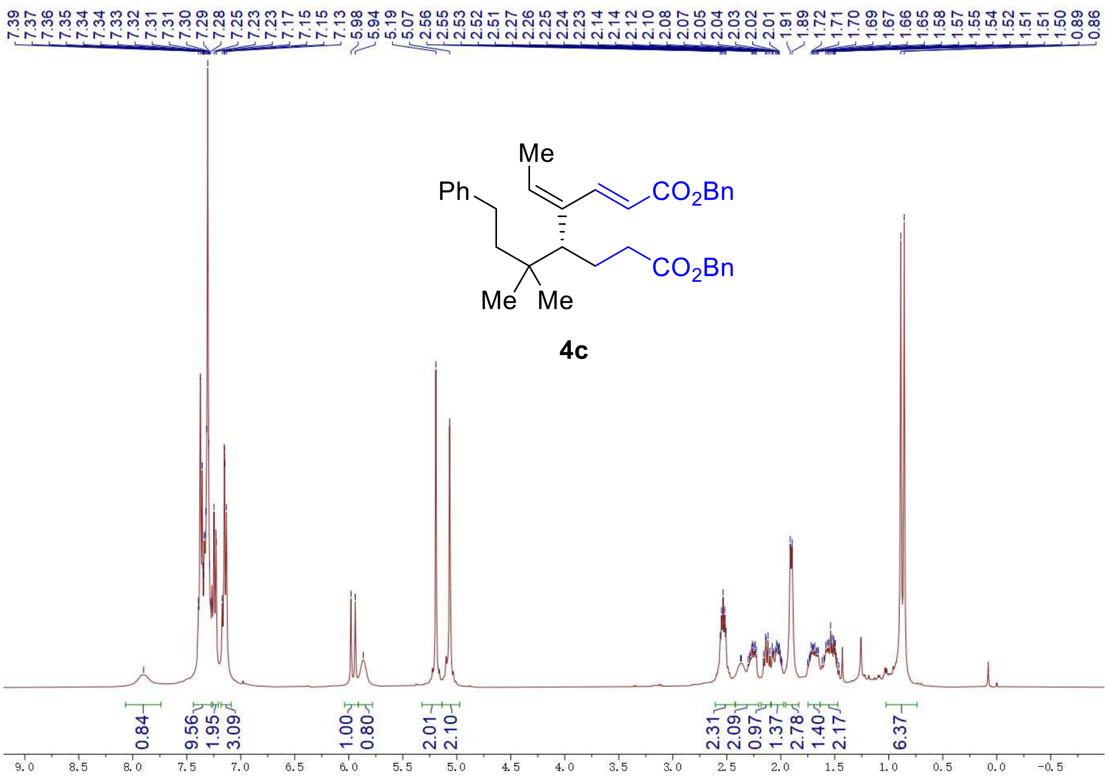

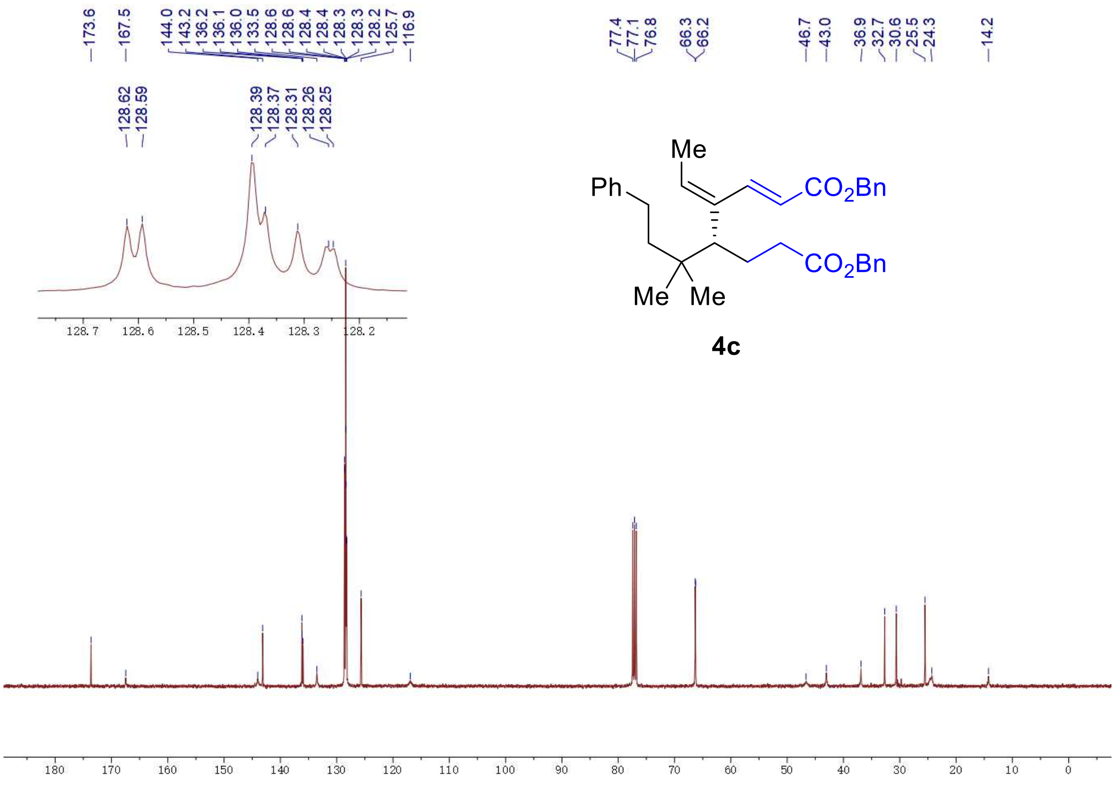

Supplementary Figure 8. ^{1}H NMR and ^{13}C NMR spectrum of compound of **4d**

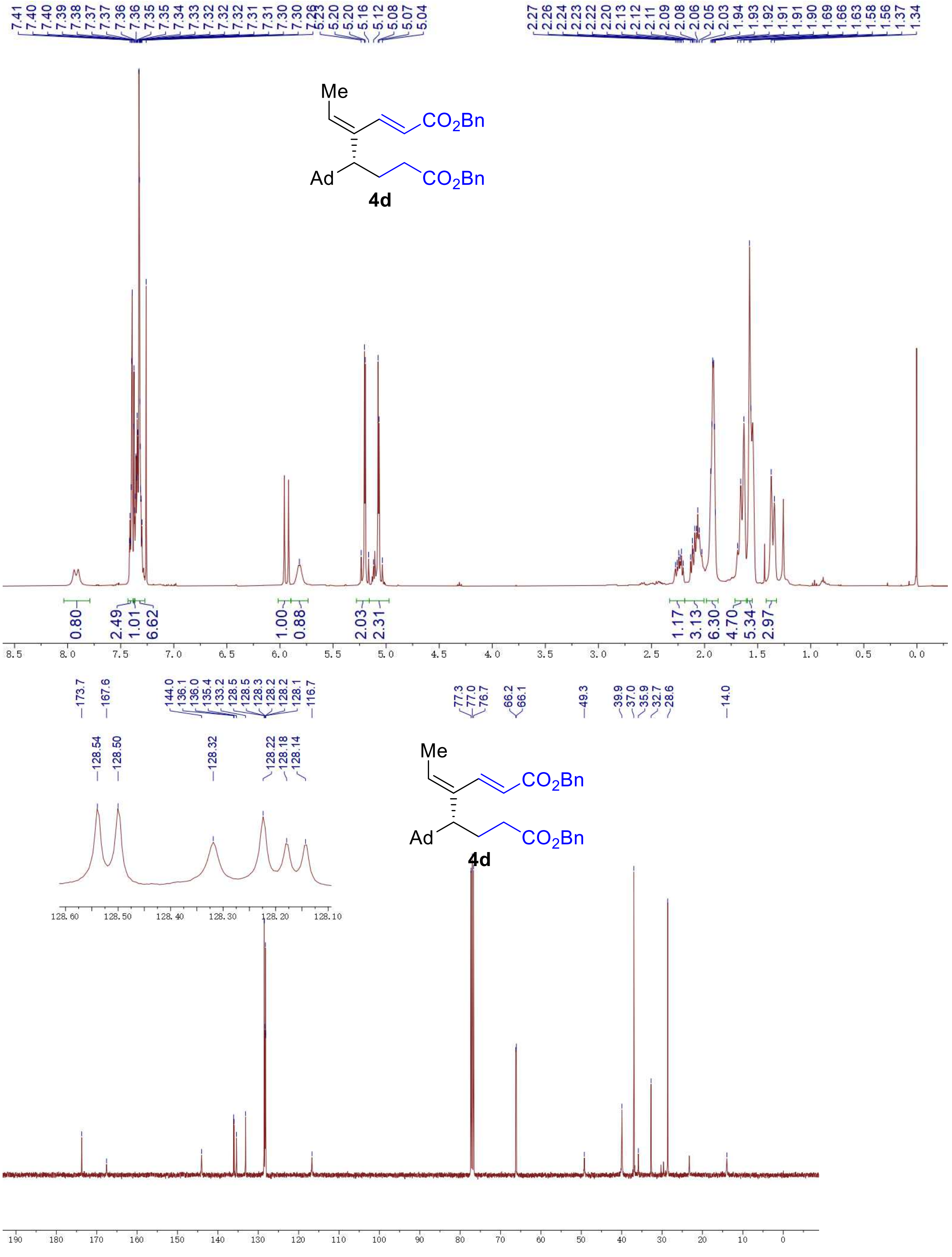

Supplementary Figure 9. ^{1}H NMR and ^{13}C NMR spectrum of compound of **4e**

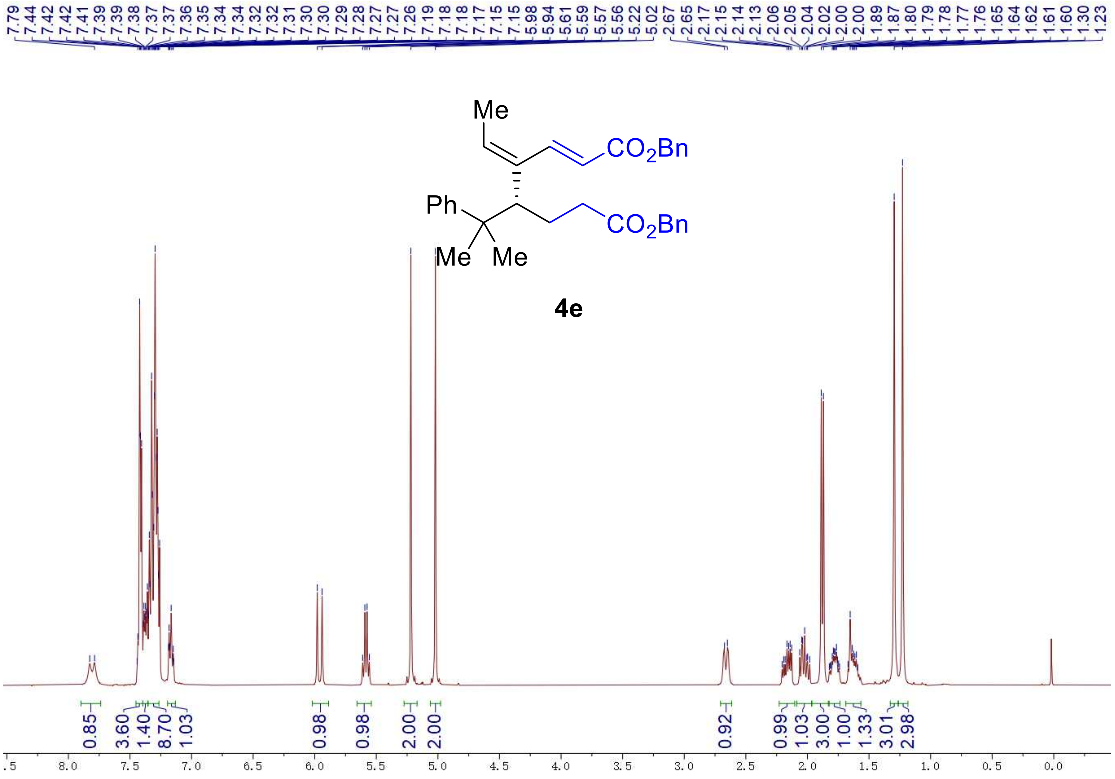

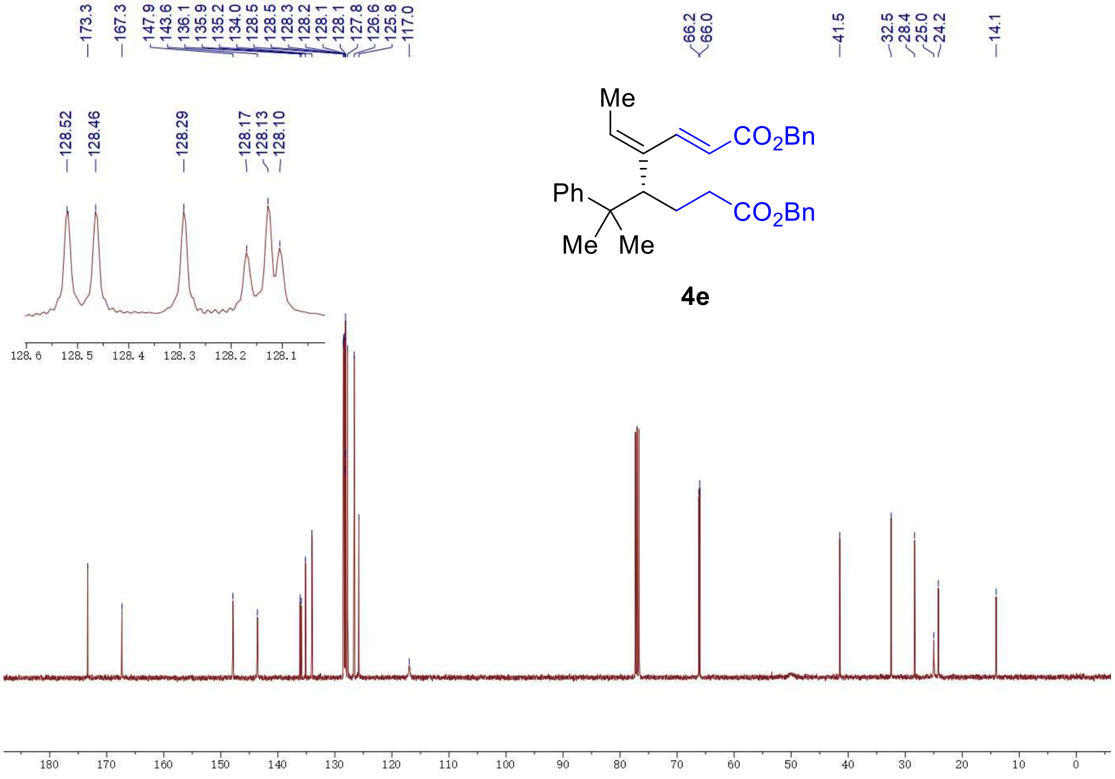

Supplementary Figure 10. ^{1}H NMR and ^{13}C NMR spectrum of compound of **4f**

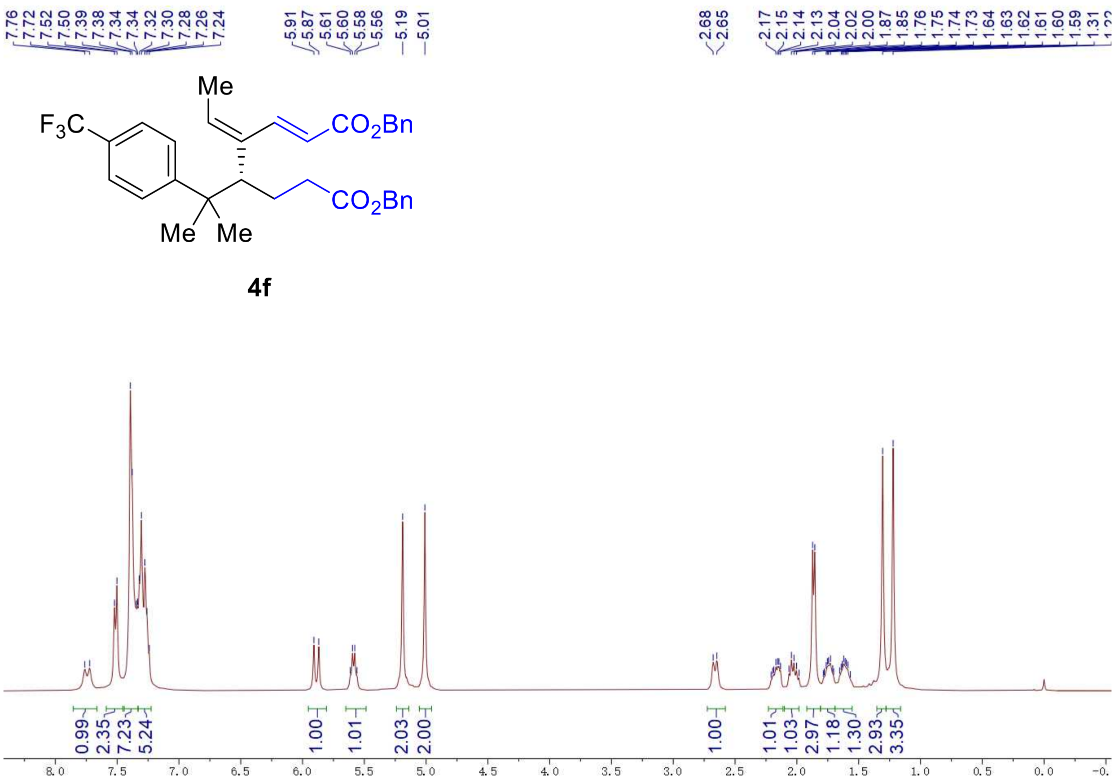

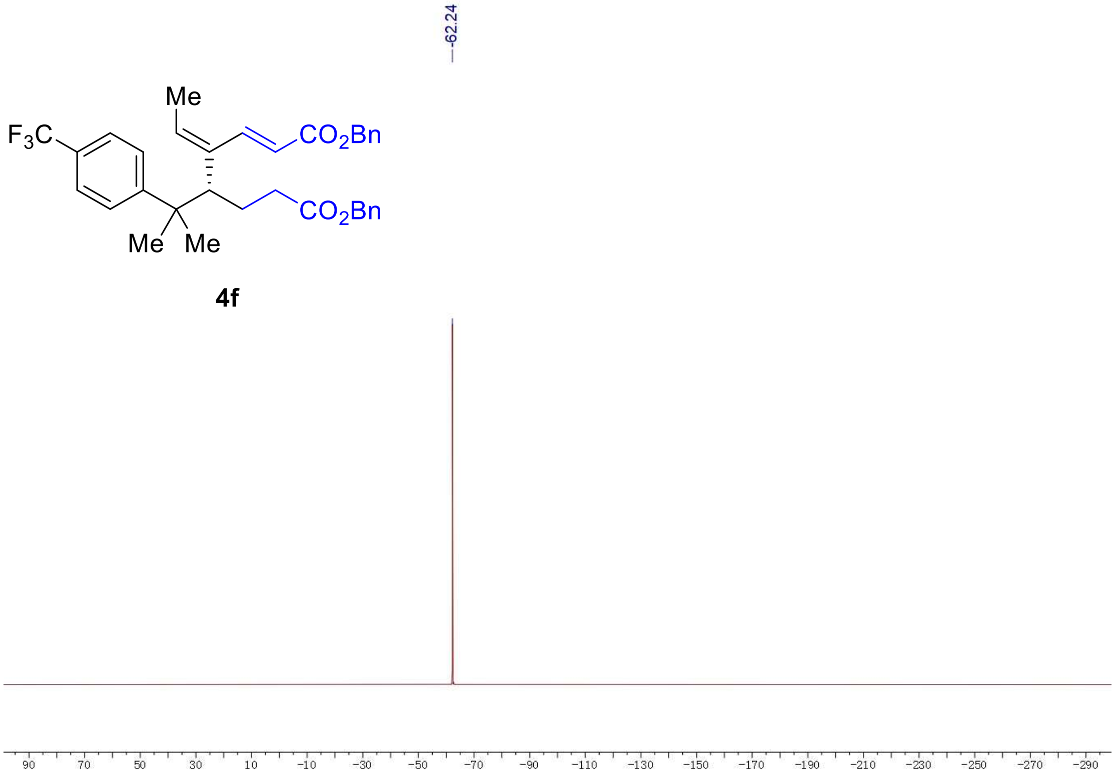

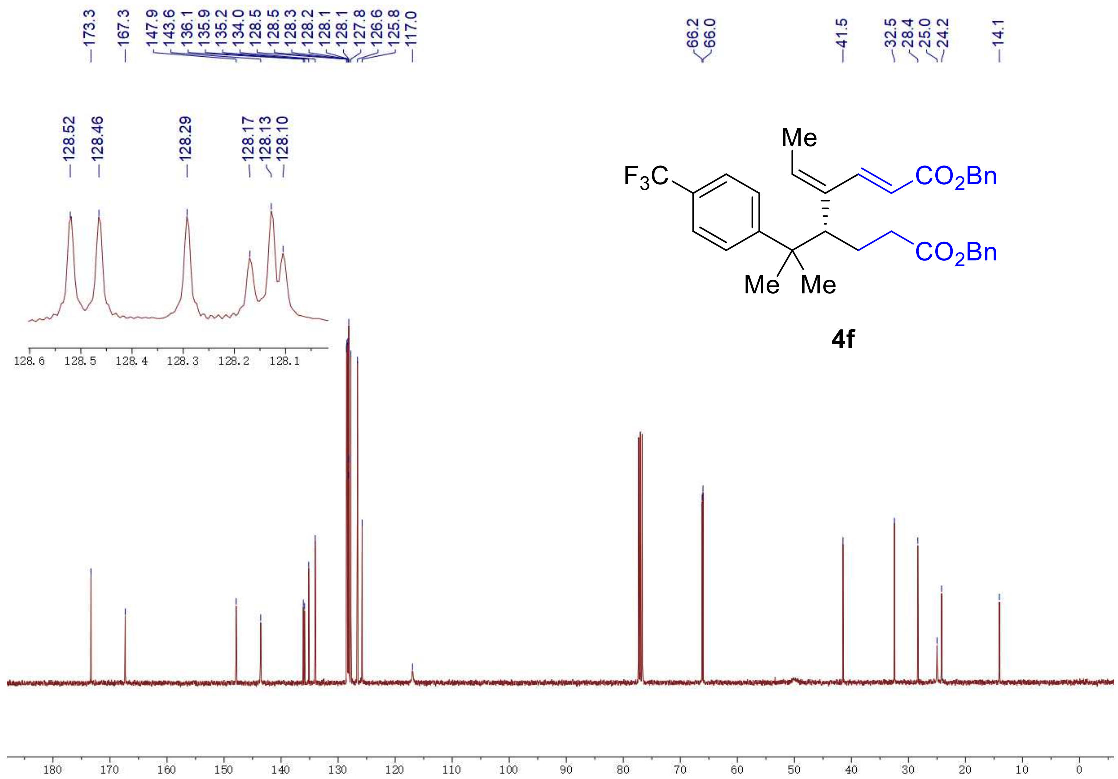

Supplementary Figure 11. ^{1}H NMR and ^{13}C NMR spectrum of compound of **4g**

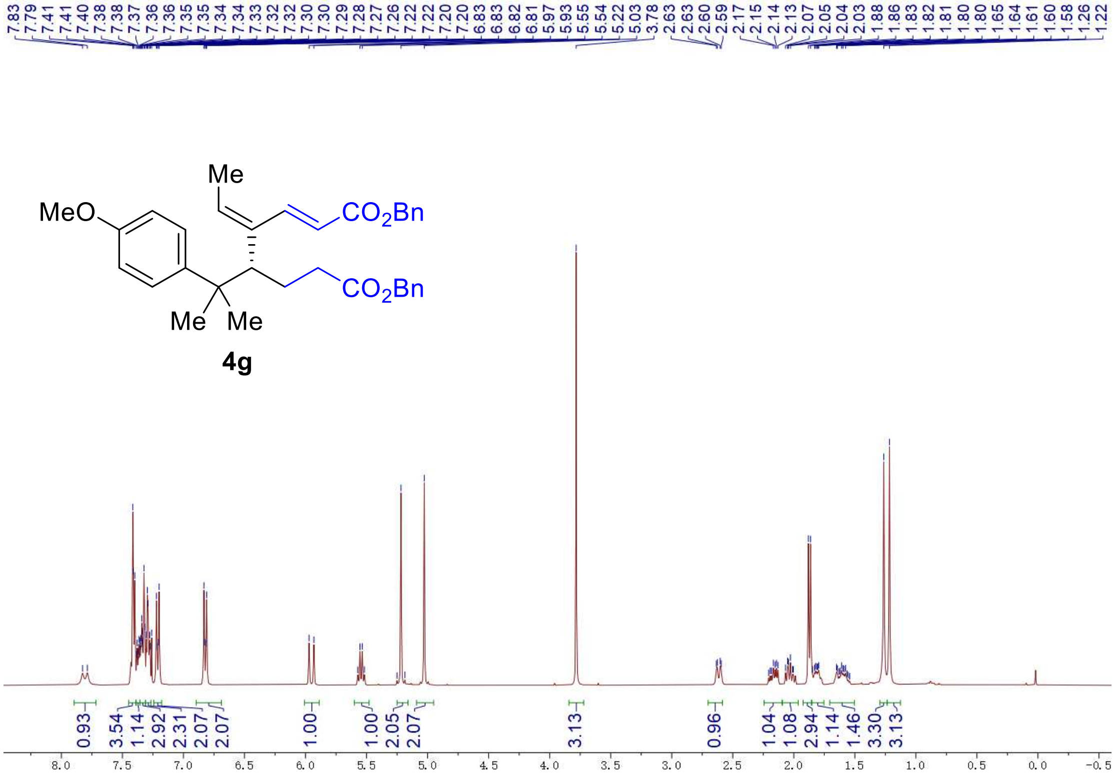

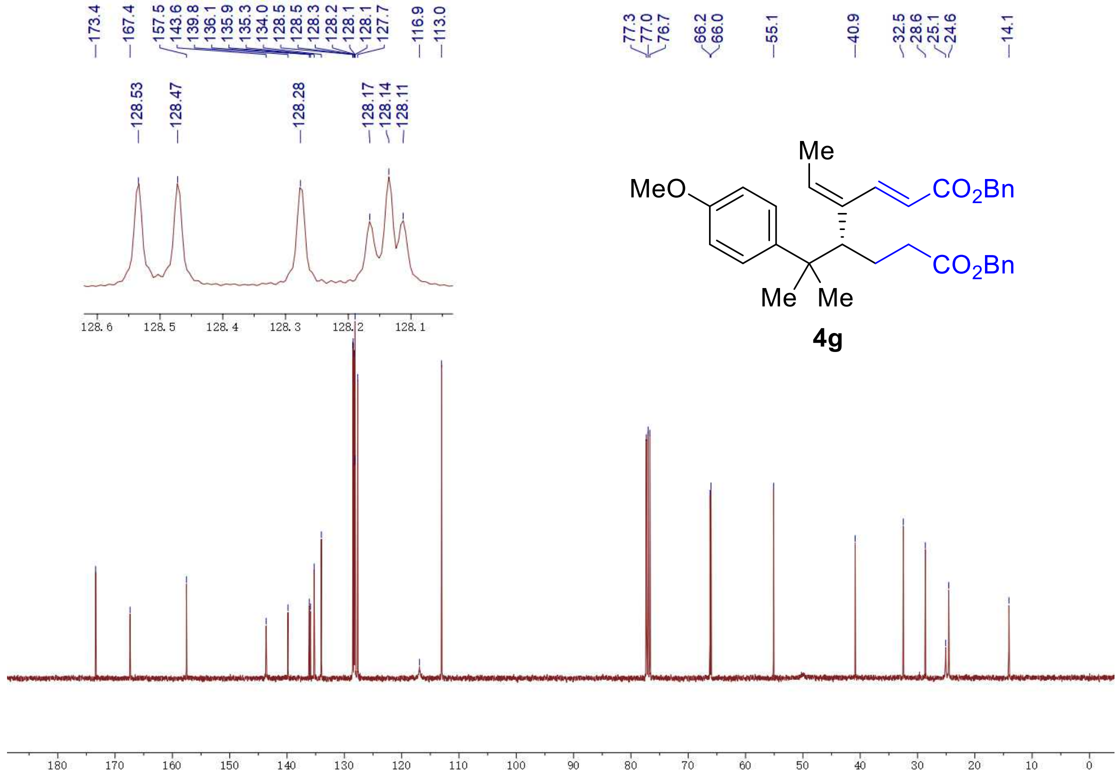

Supplementary Figure 12. ^{1}H NMR and ^{13}C NMR spectrum of compound of **4h**

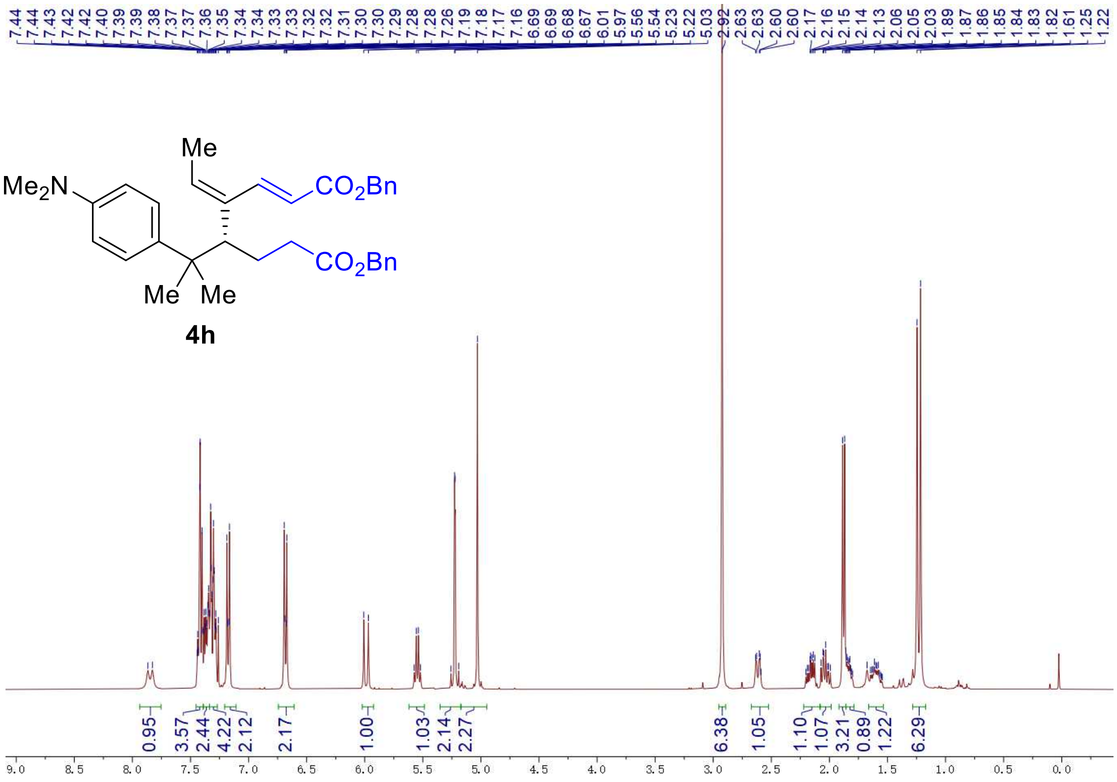

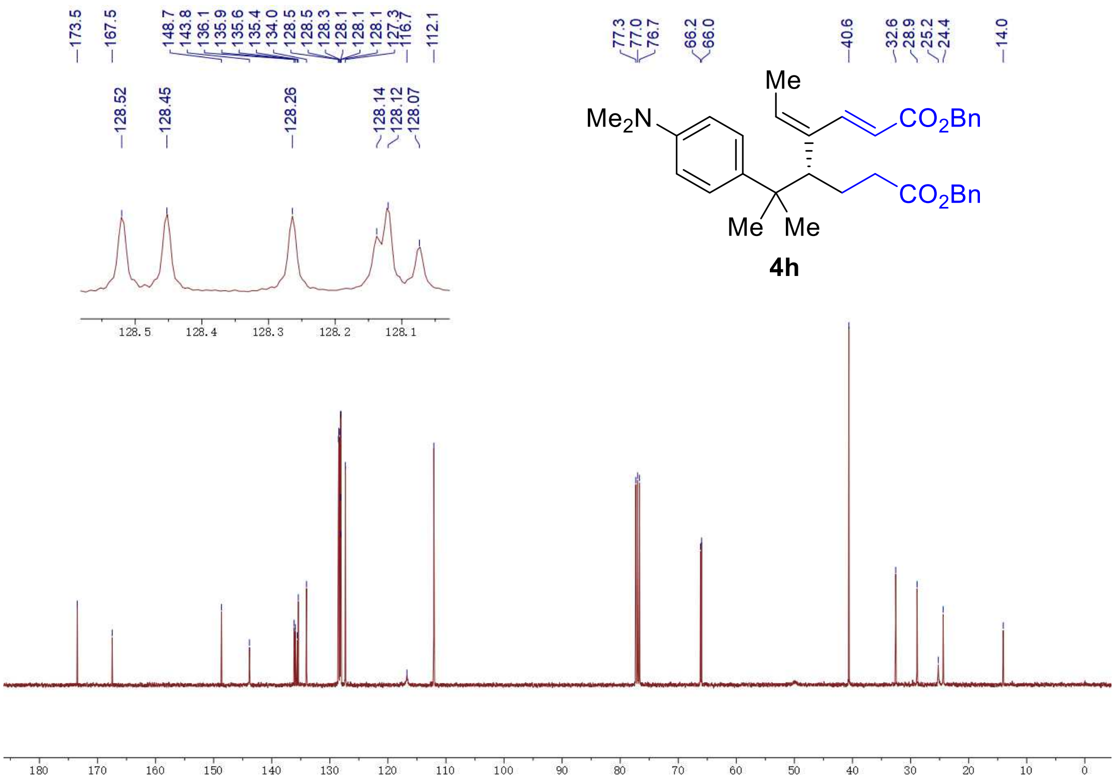

Supplementary Figure 13. ^{1}H NMR and ^{13}C NMR spectrum of compound of **4i**

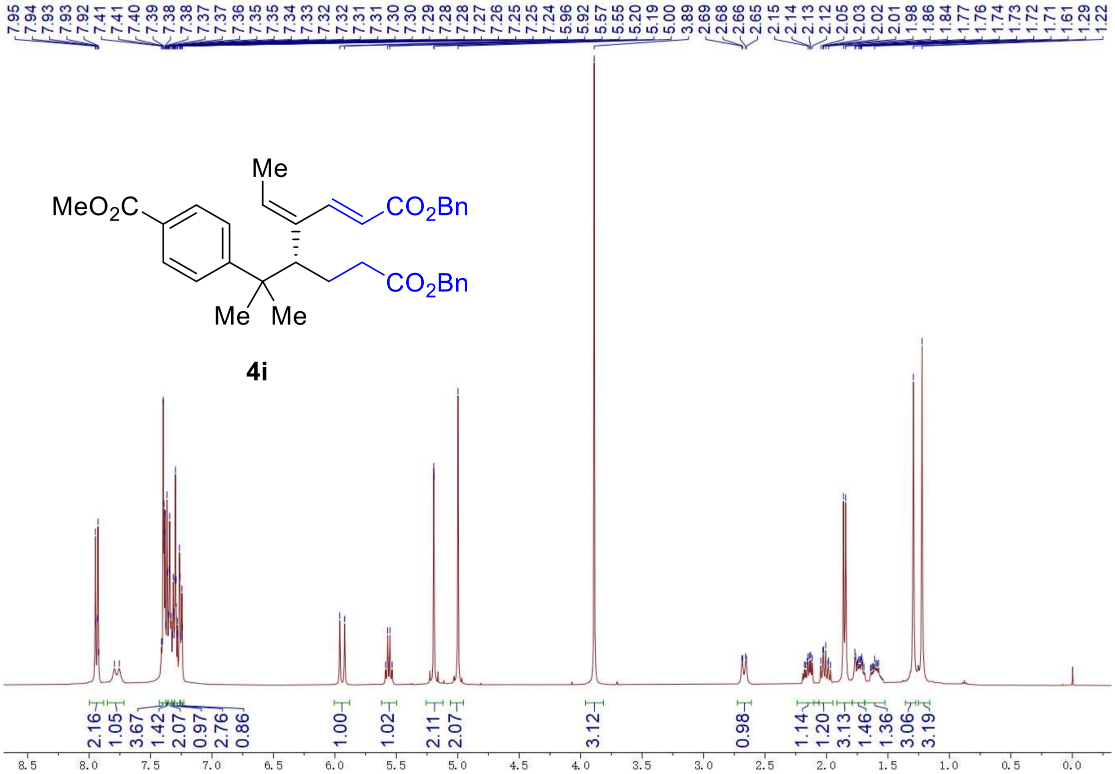

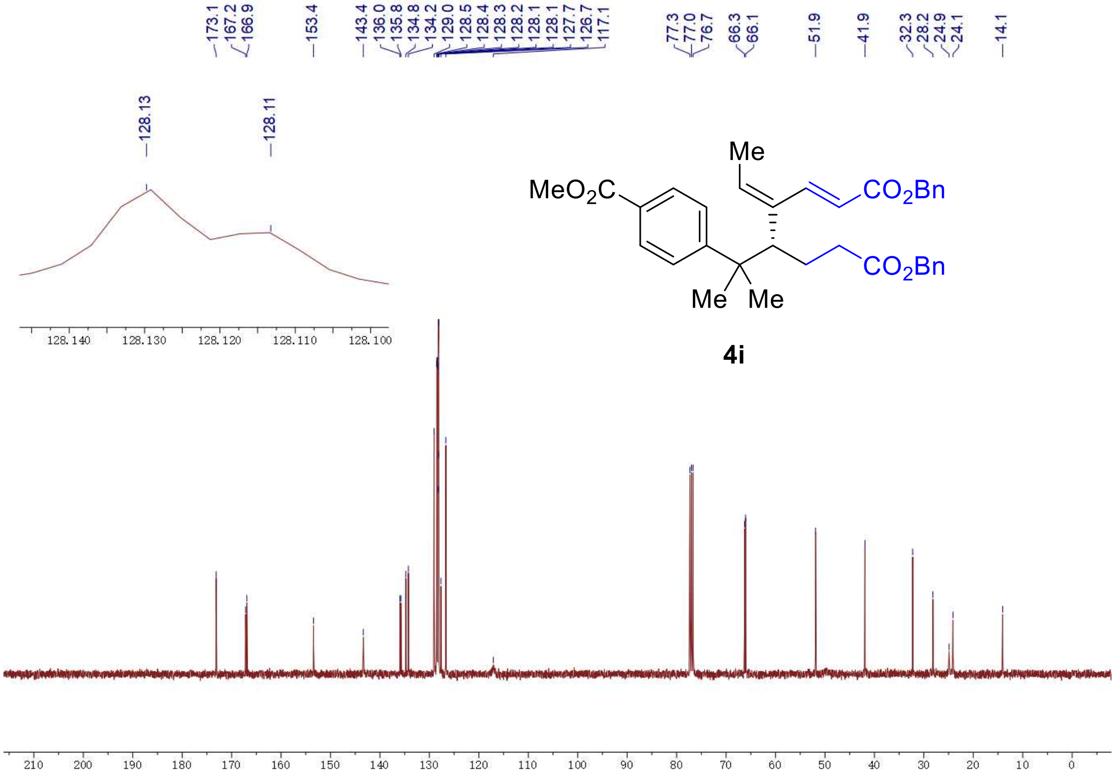

Supplementary Figure 14. ^{1}H NMR and ^{13}C NMR spectrum of compound of **4j**

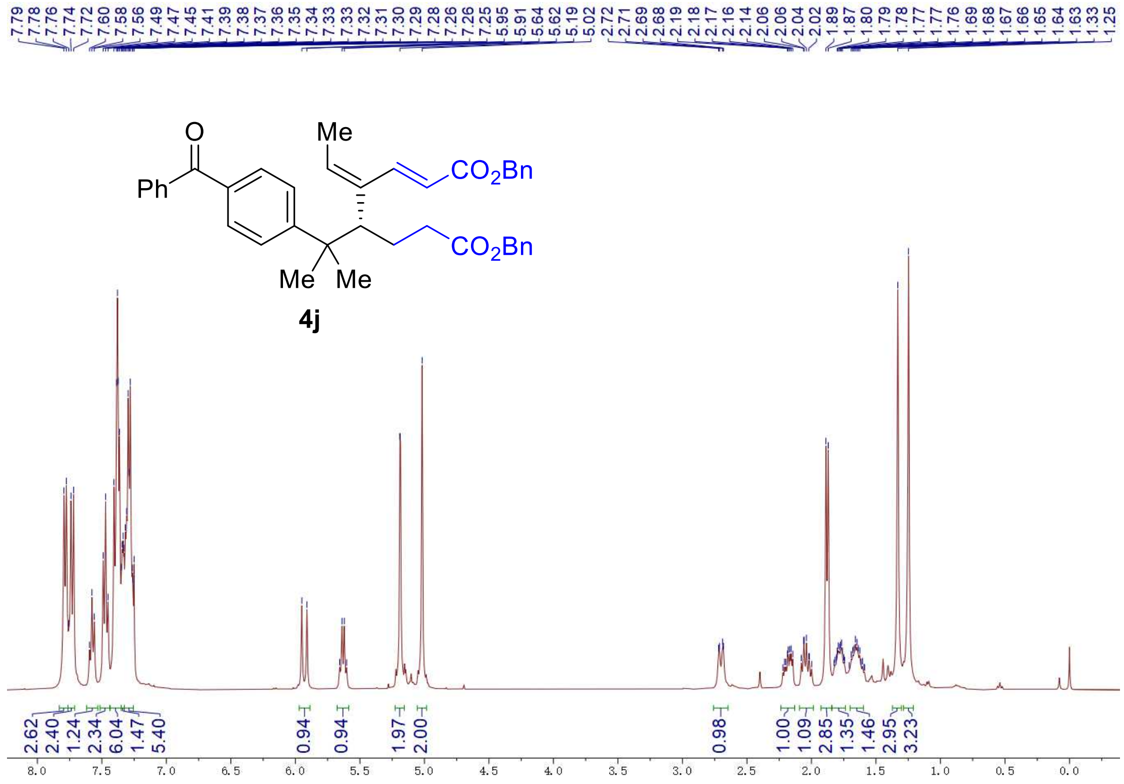

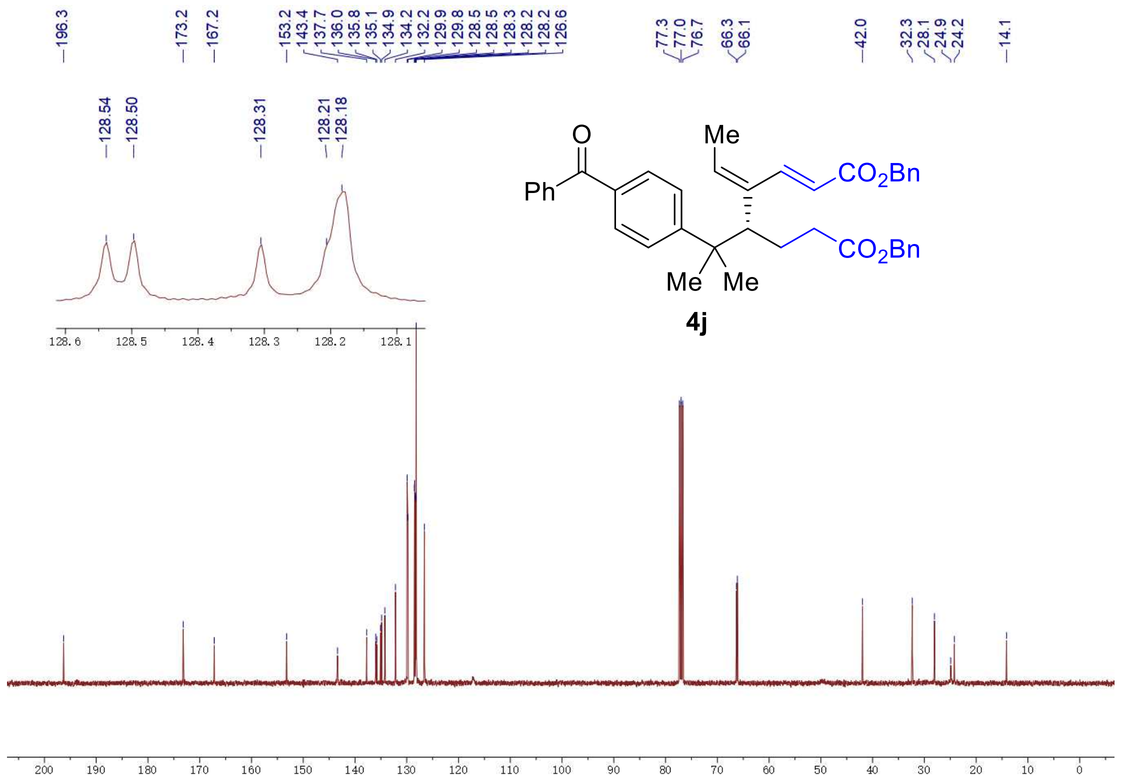

Supplementary Figure 15. ^{1}H NMR and ^{13}C NMR spectrum of compound of **4k**

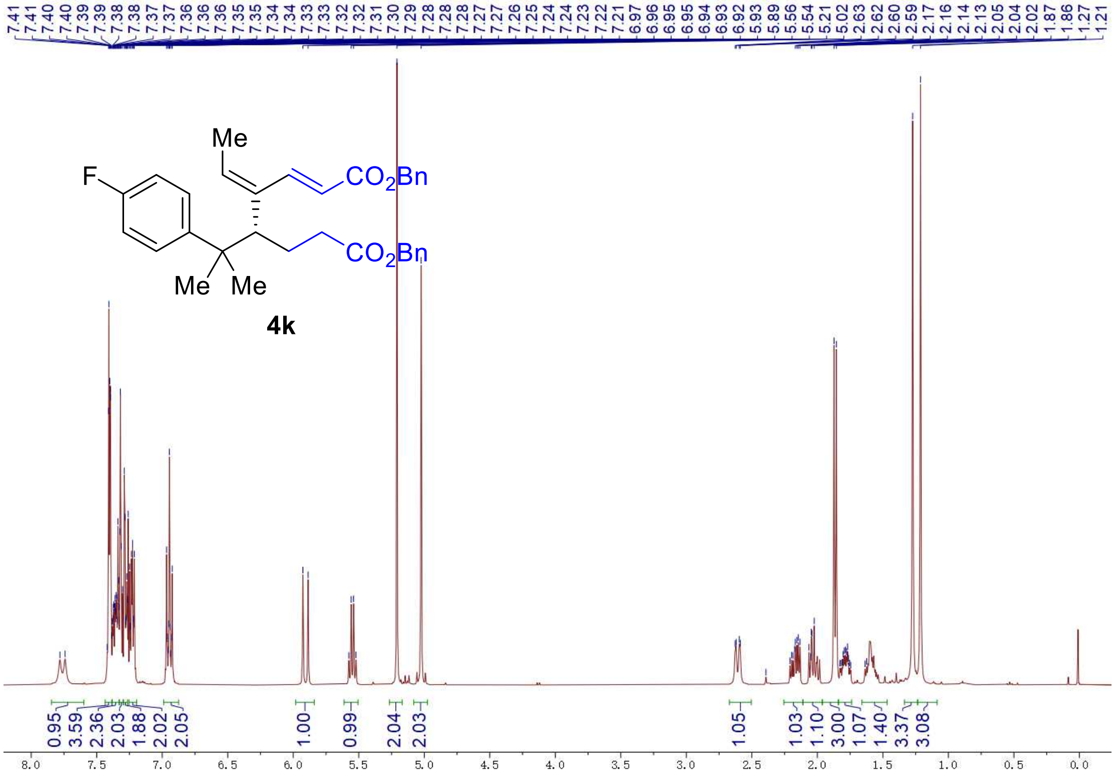

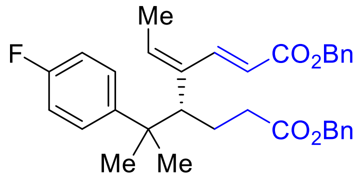

4k

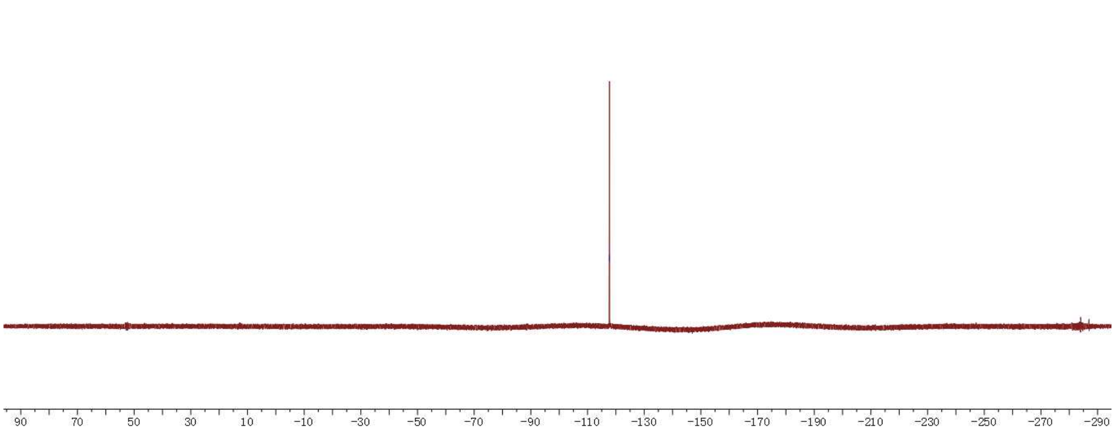
-117.7
90
70
50
30
10
−10
−30
−50
−70
−90
−110
−130
−150
−170
−190
−210
−230
−250
−270
−290

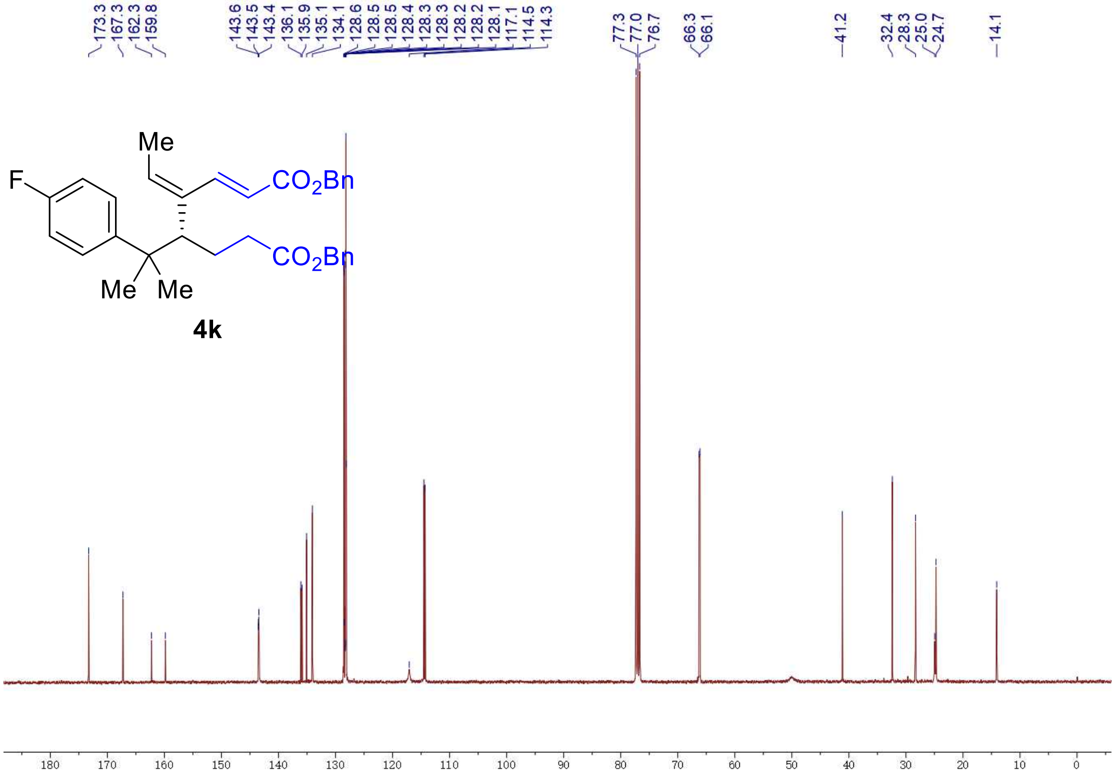
173.3
167.3
162.3
159.8
143.6
143.5
143.4
136.1
135.9
135.1
134.1
128.6
128.5
128.5
128.4
128.3
128.3
128.2
128.2
128.1
117.1
114.5
114.3
77.3
77.0
76.7
66.3
66.1
41.2
32.4
28.3
25.0
24.7
14.1
Me
F
CO2Bn
CO2Bn
Me
Me
4k
180
170
160
150
140
130
120
110
100
90
80
70
60
50
40
30
20
10
0

Supplementary Figure 16. ^{1}H NMR and ^{13}C NMR spectrum of compound of **4l**

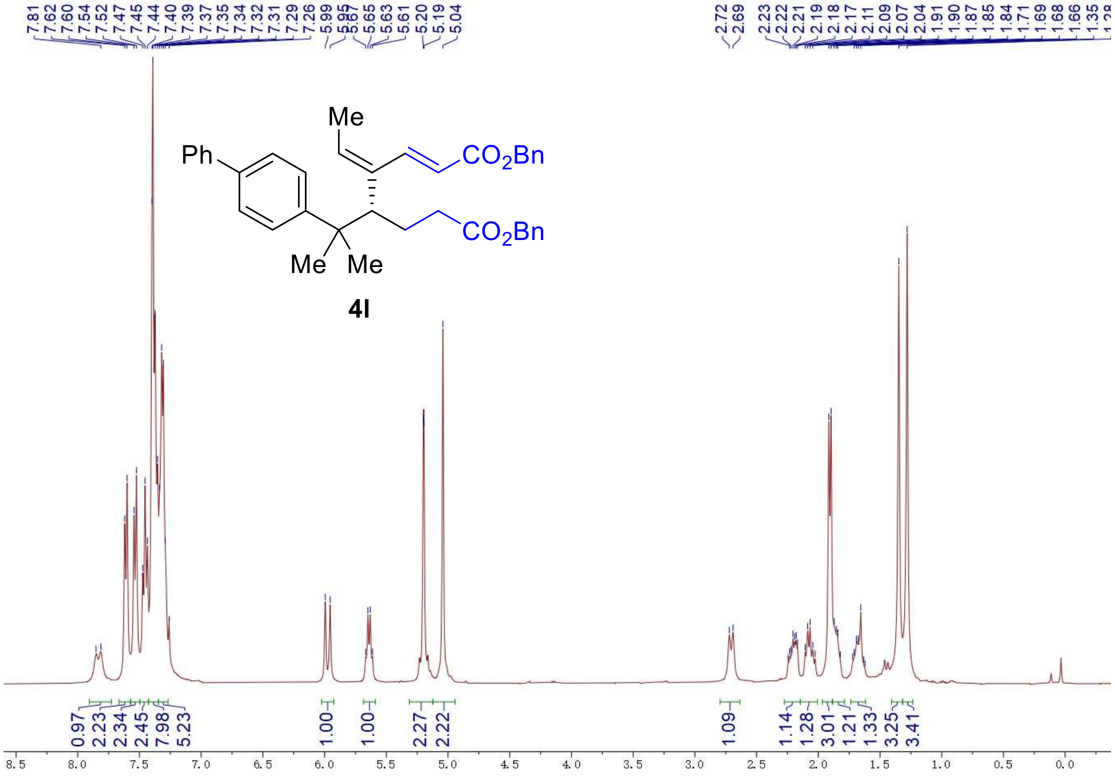

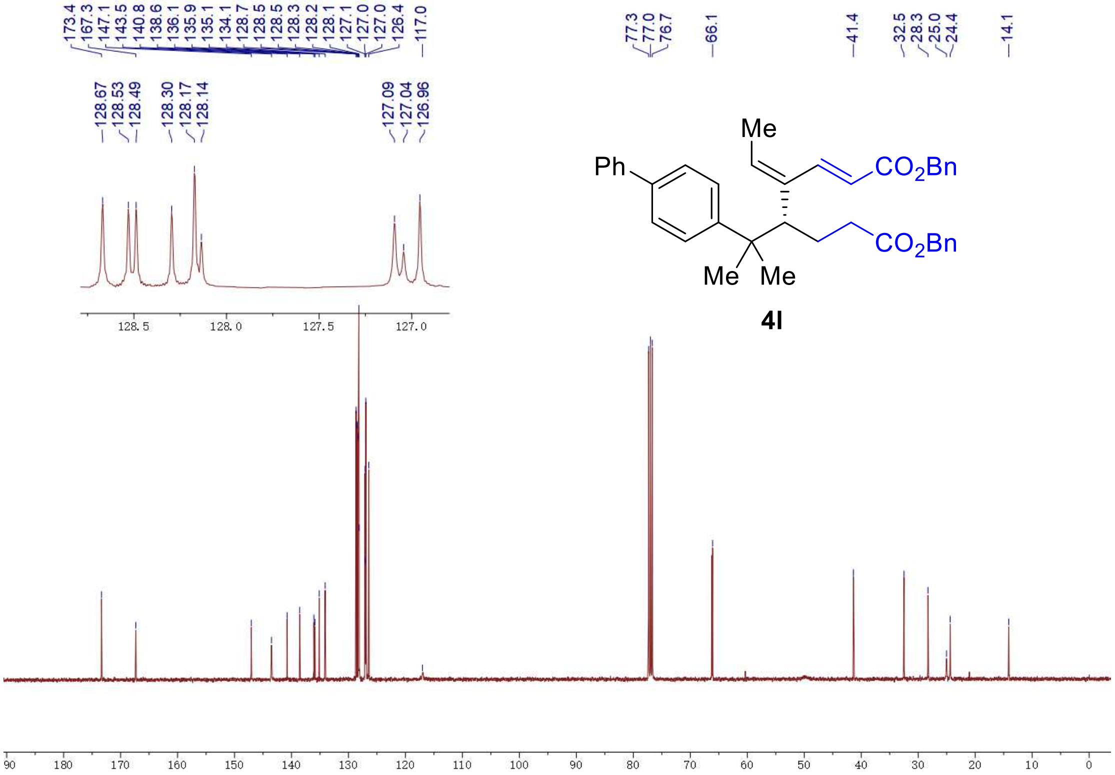

Supplementary Figure 17. ^{1}H NMR and ^{13}C NMR spectrum of compound of **4m**

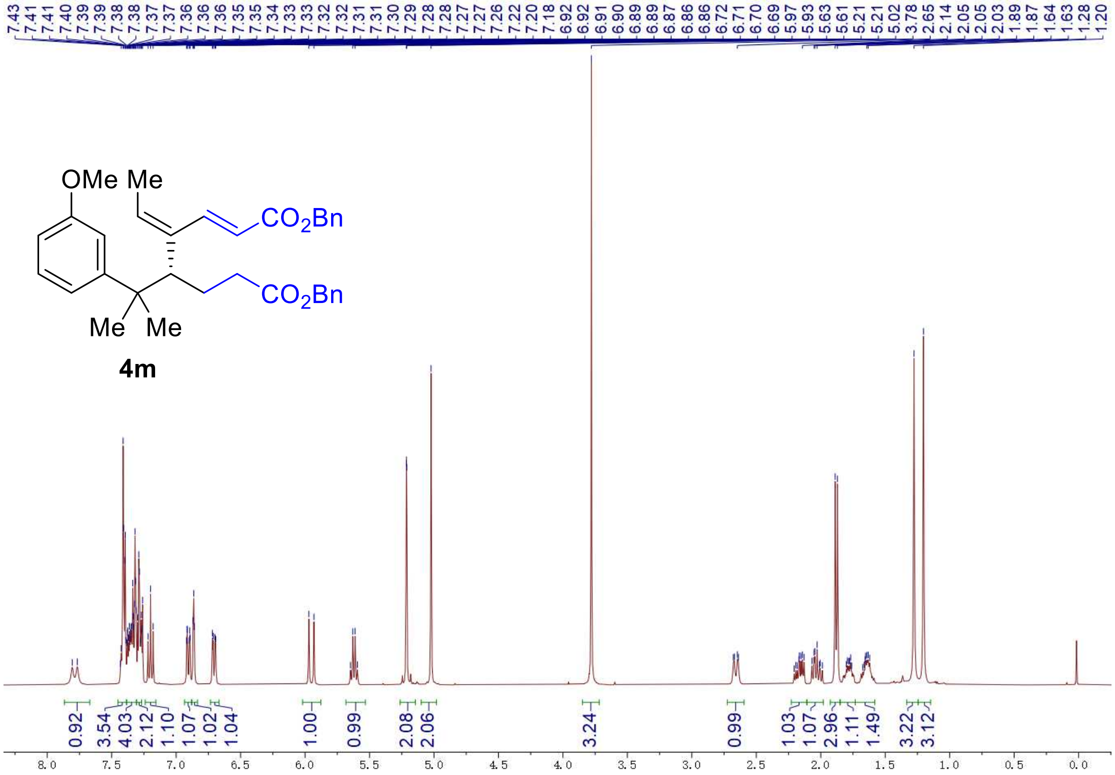

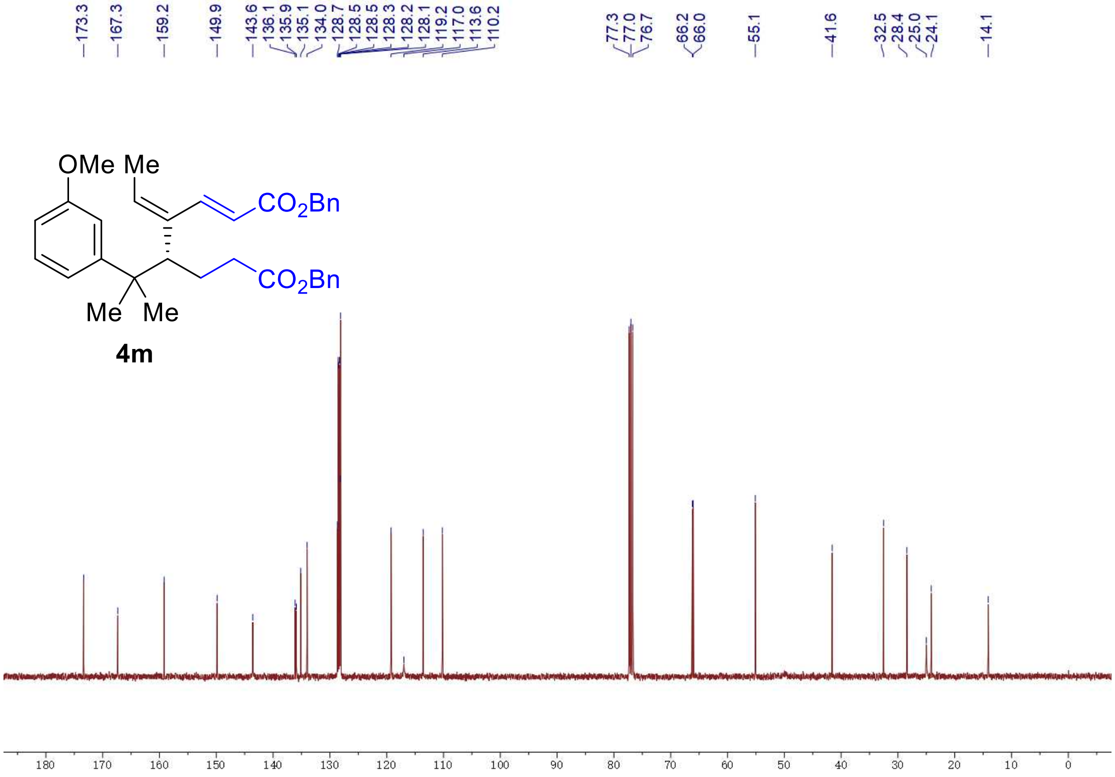

Supplementary Figure 18. ^{1}H NMR and ^{13}C NMR spectrum of compound of **4n**

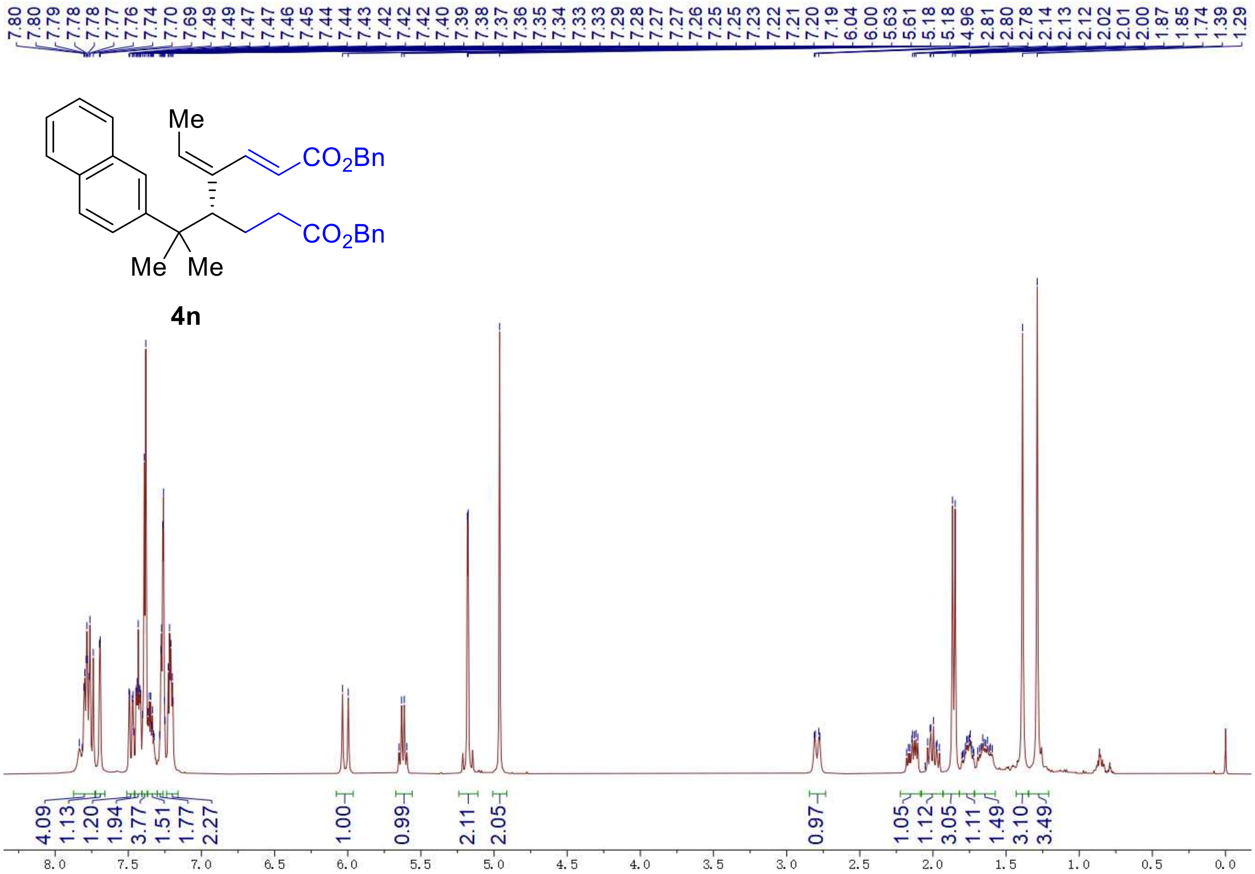

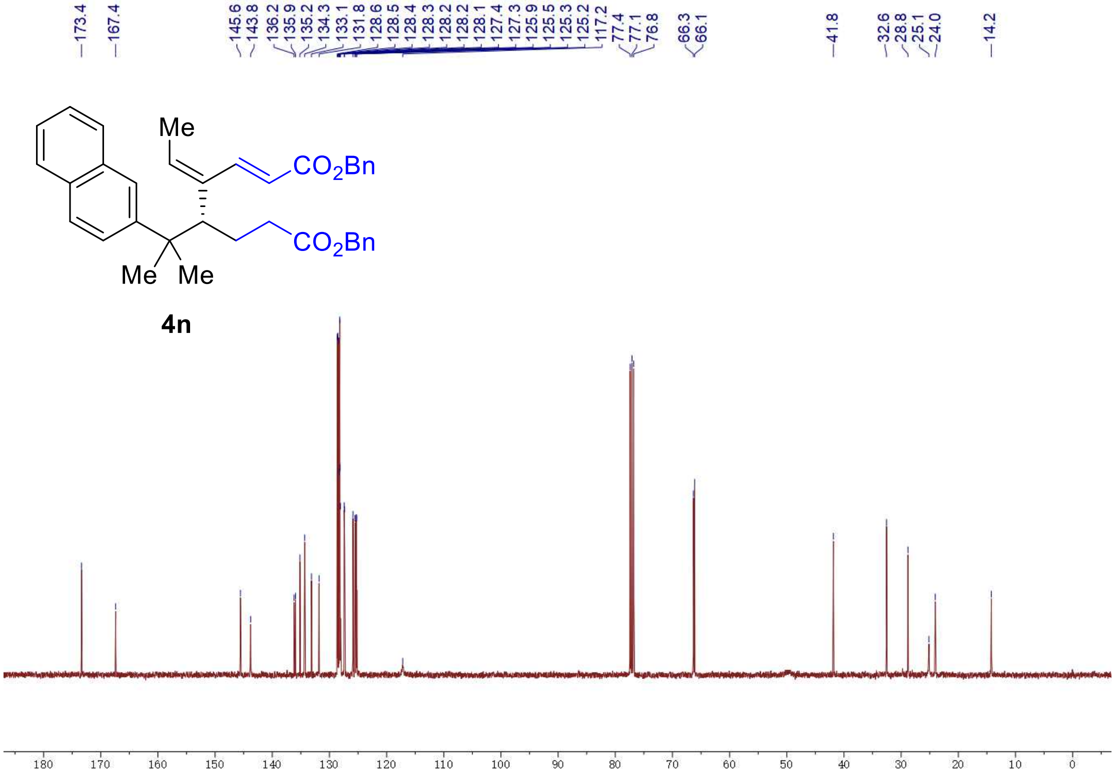

Supplementary Figure 19. ^{1}H NMR and ^{13}C NMR spectrum of compound of **4o**

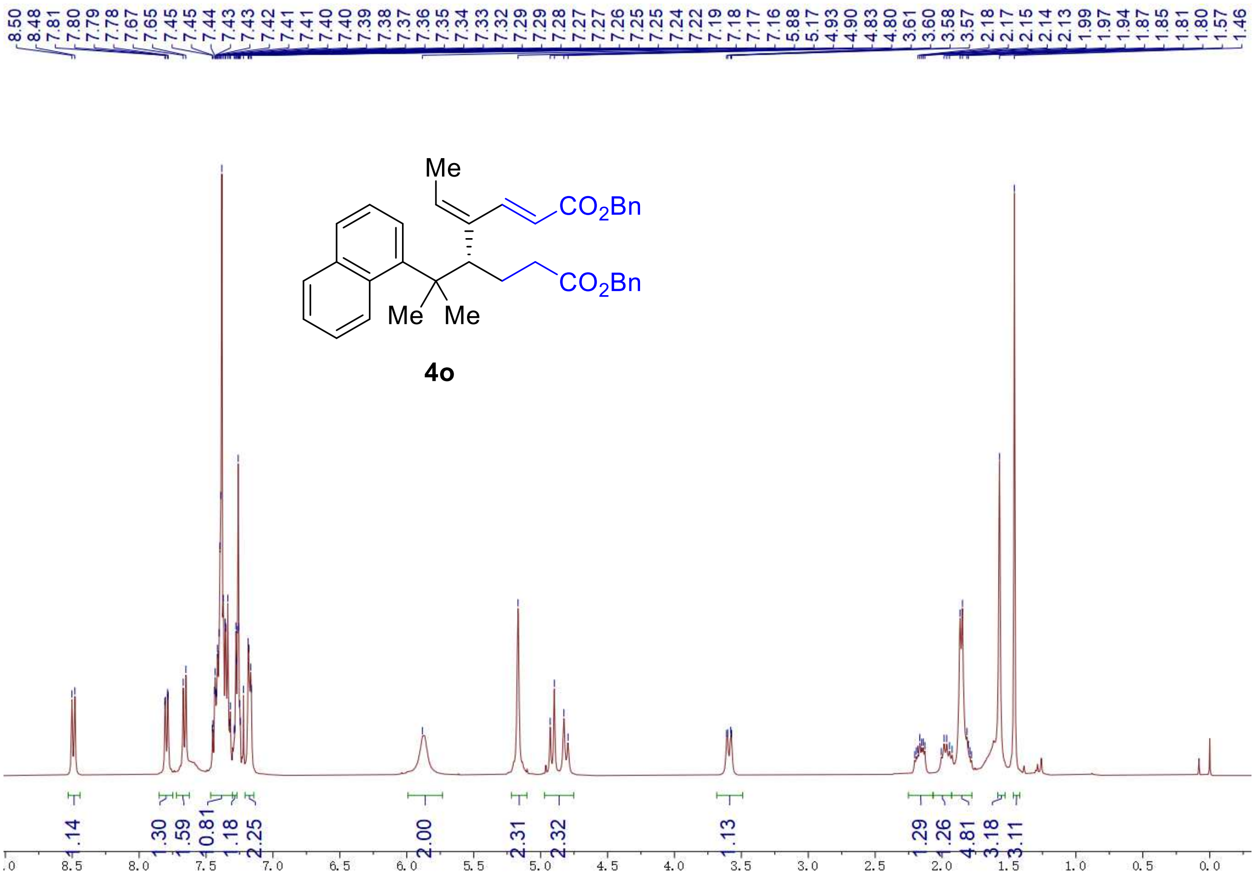

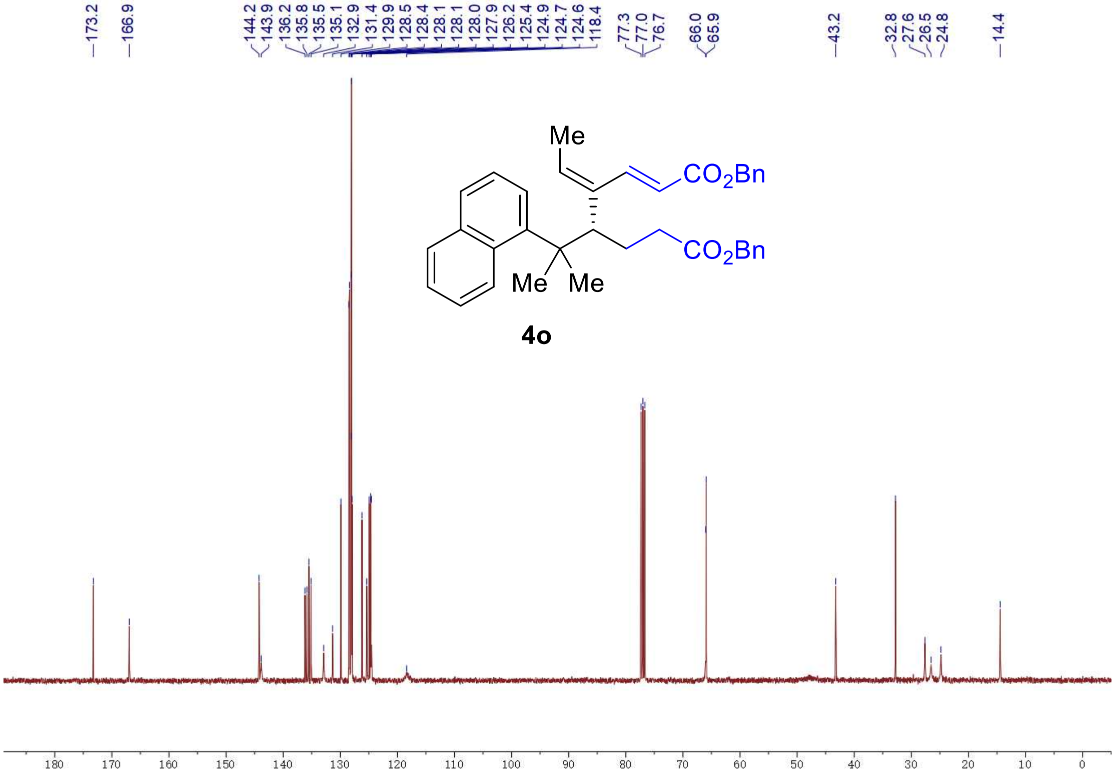

Supplementary Figure 20. ^{1}**H NMR and** ^{13}**C NMR** spectrum of compound of **4p**

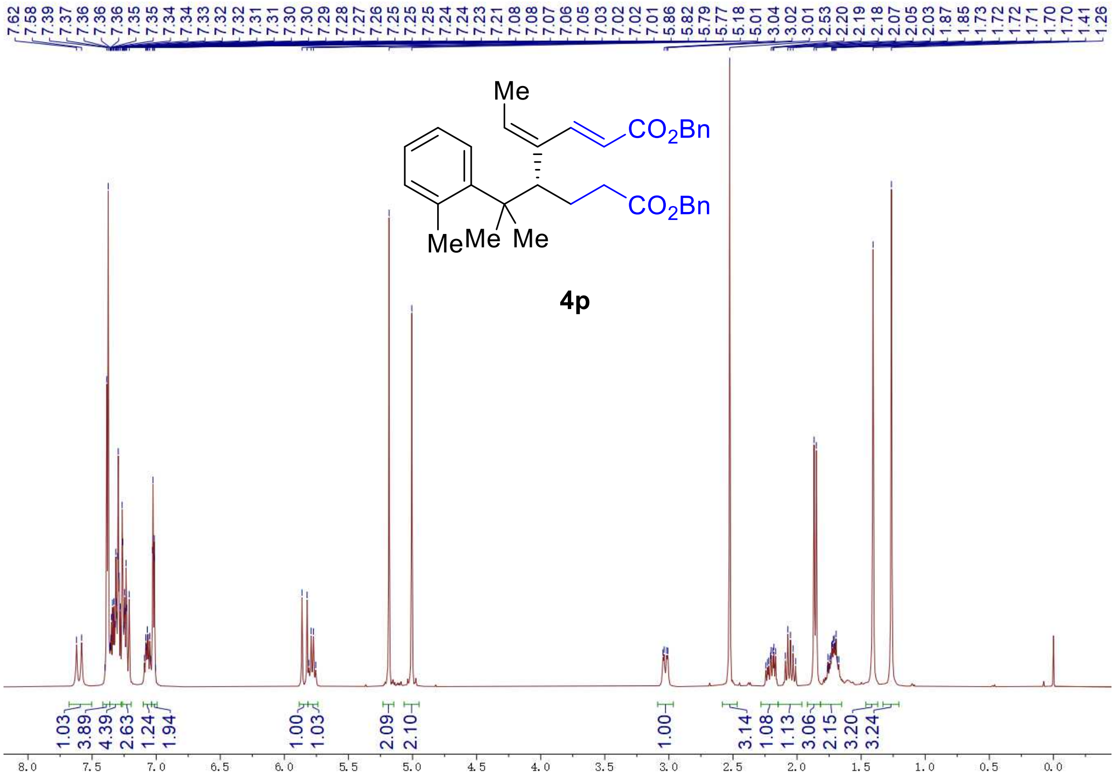

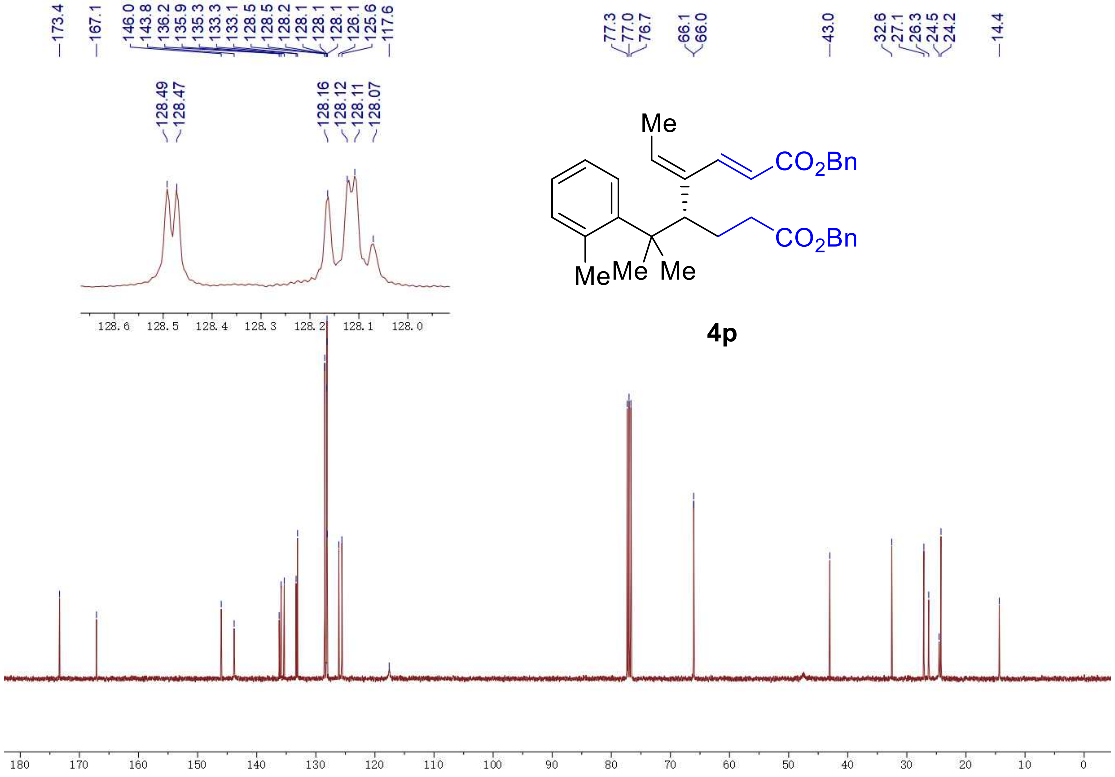

Supplementary Figure 21. ^{1}**H NMR and** ^{13}**C NMR** spectrum of compound of **4q**

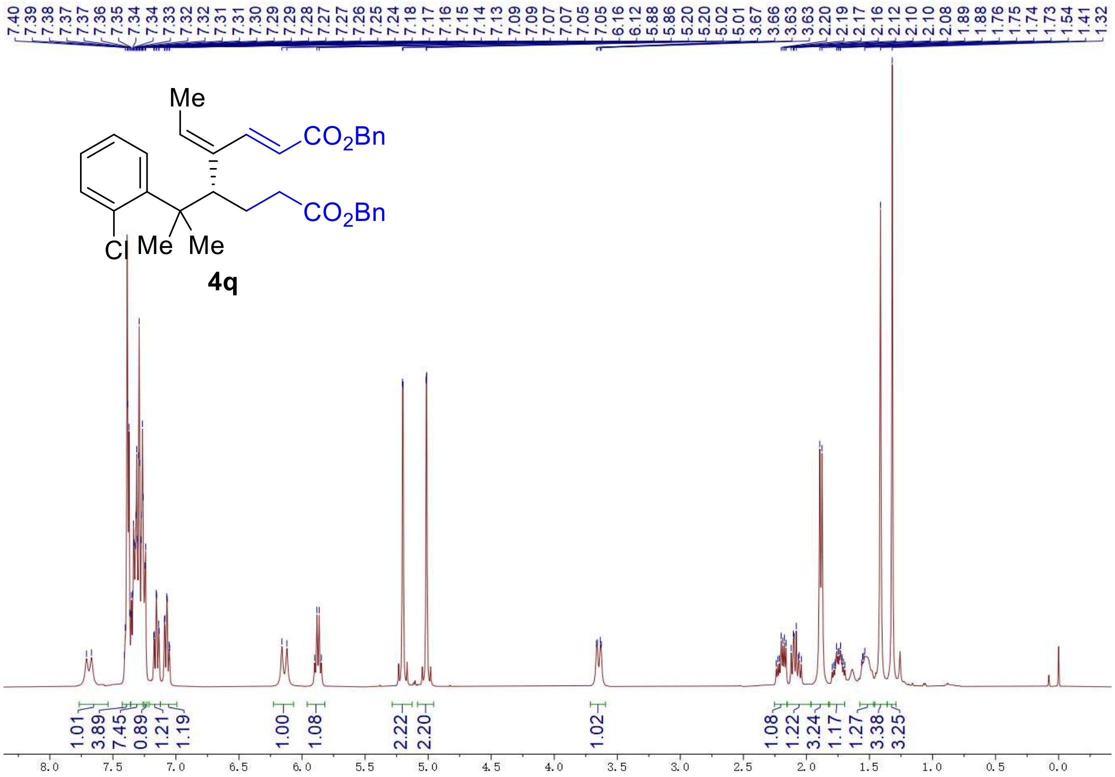

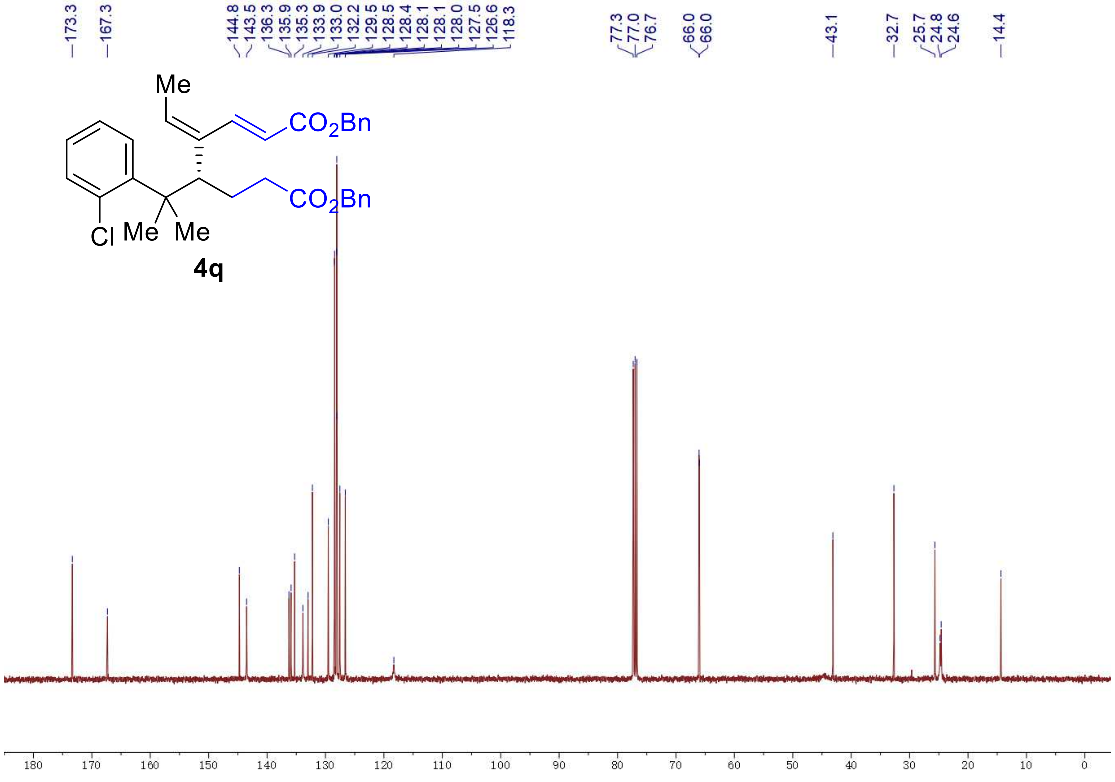

Supplementary Figure 22. ^{1}H NMR and ^{13}C NMR spectrum of compound of **4r**

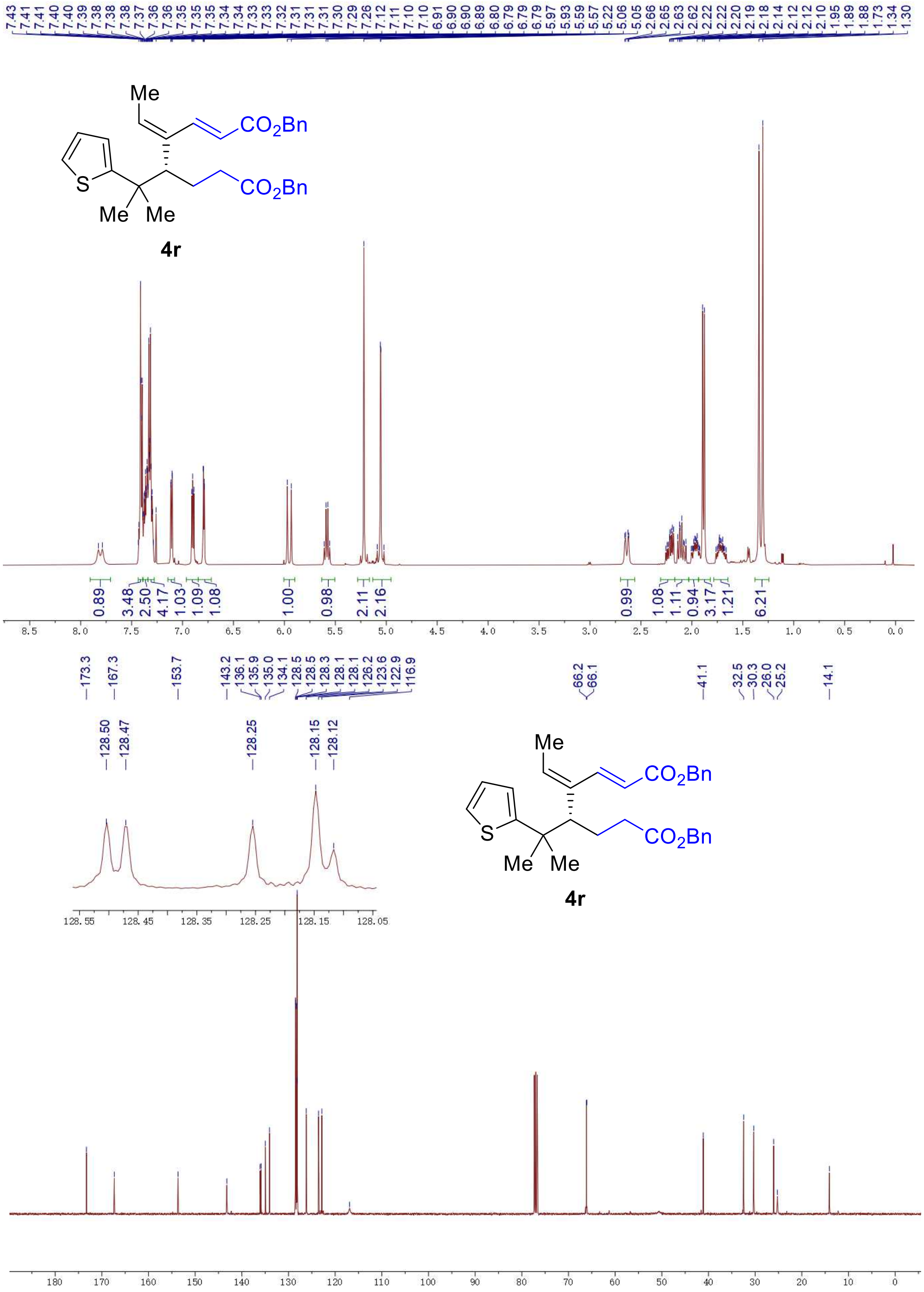

Supplementary Figure 23. ^{1}**H NMR and** ^{13}**C NMR** spectrum of compound of **4s**

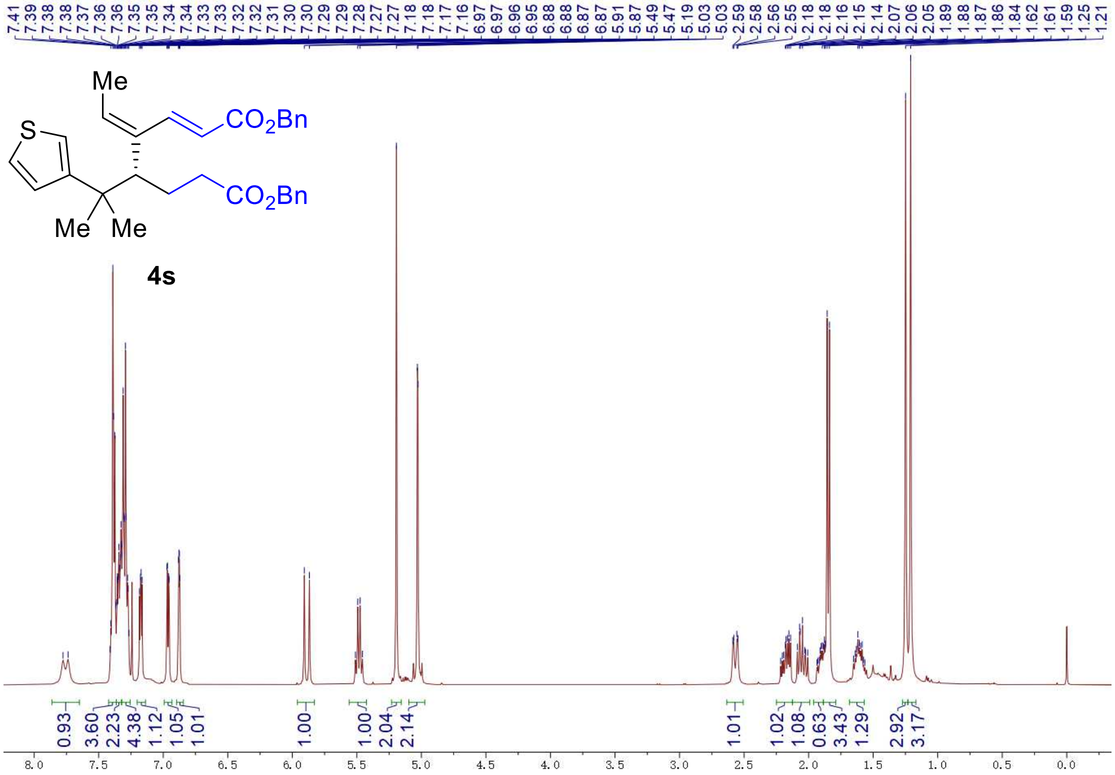

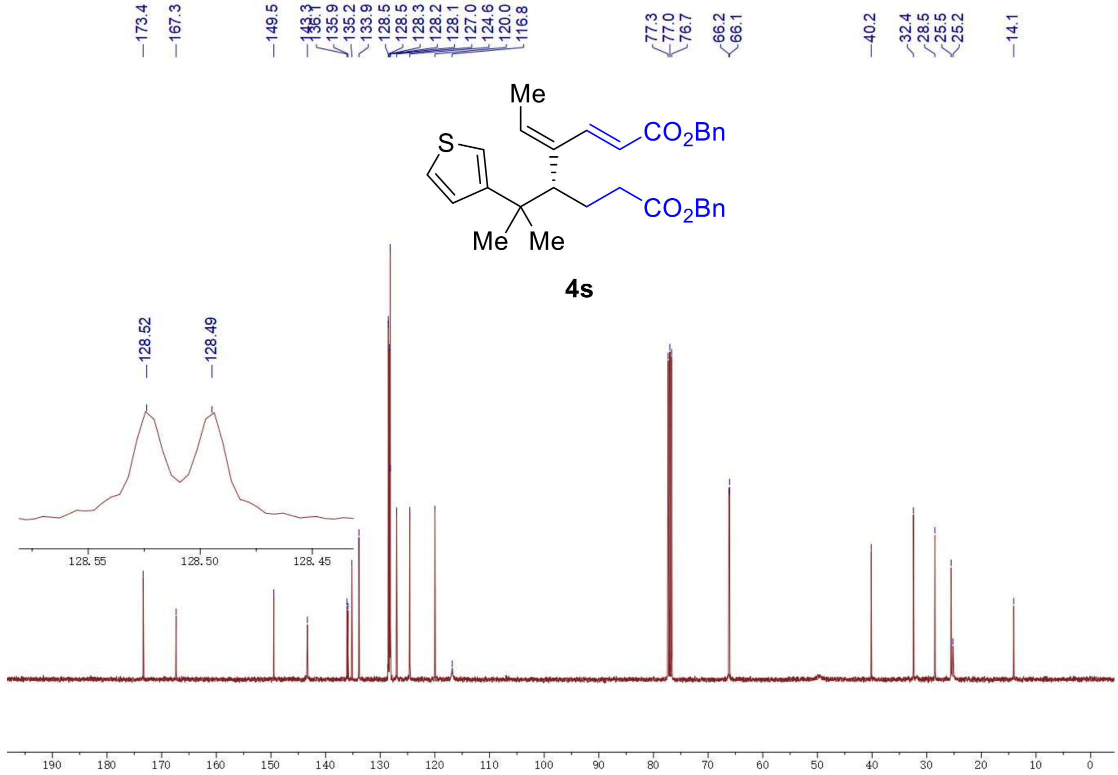

Supplementary Figure 24. ^{1}H NMR and ^{13}C NMR spectrum of compound of **4t**

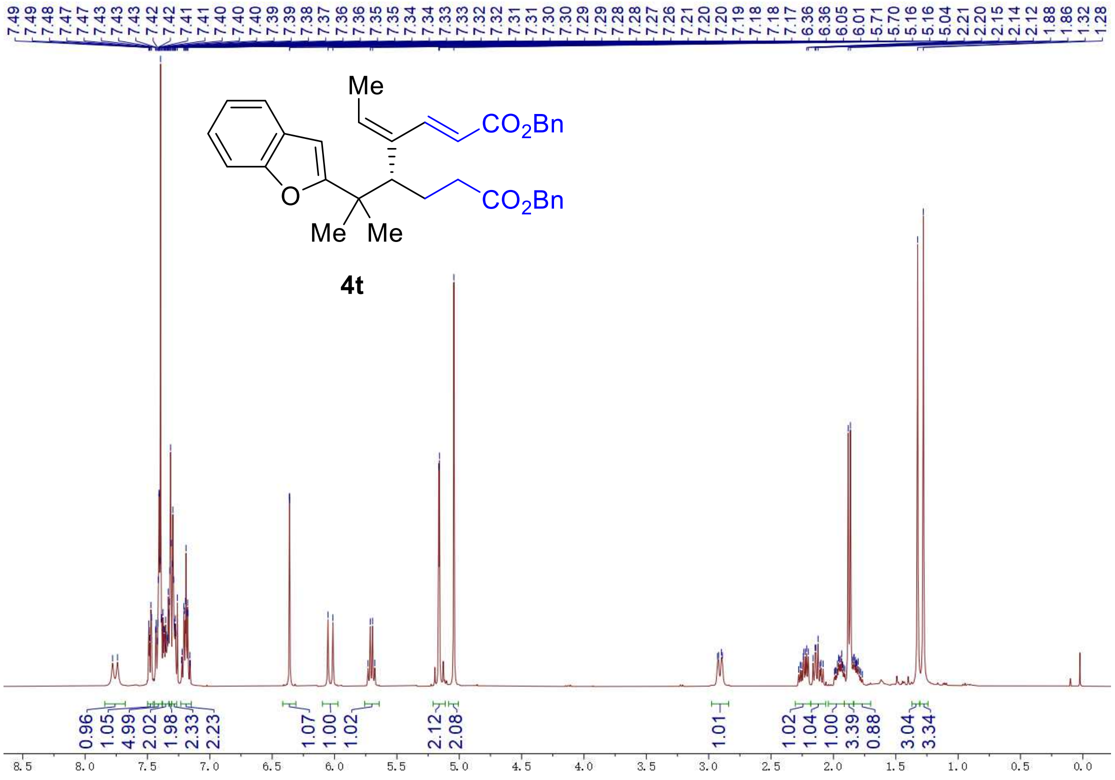

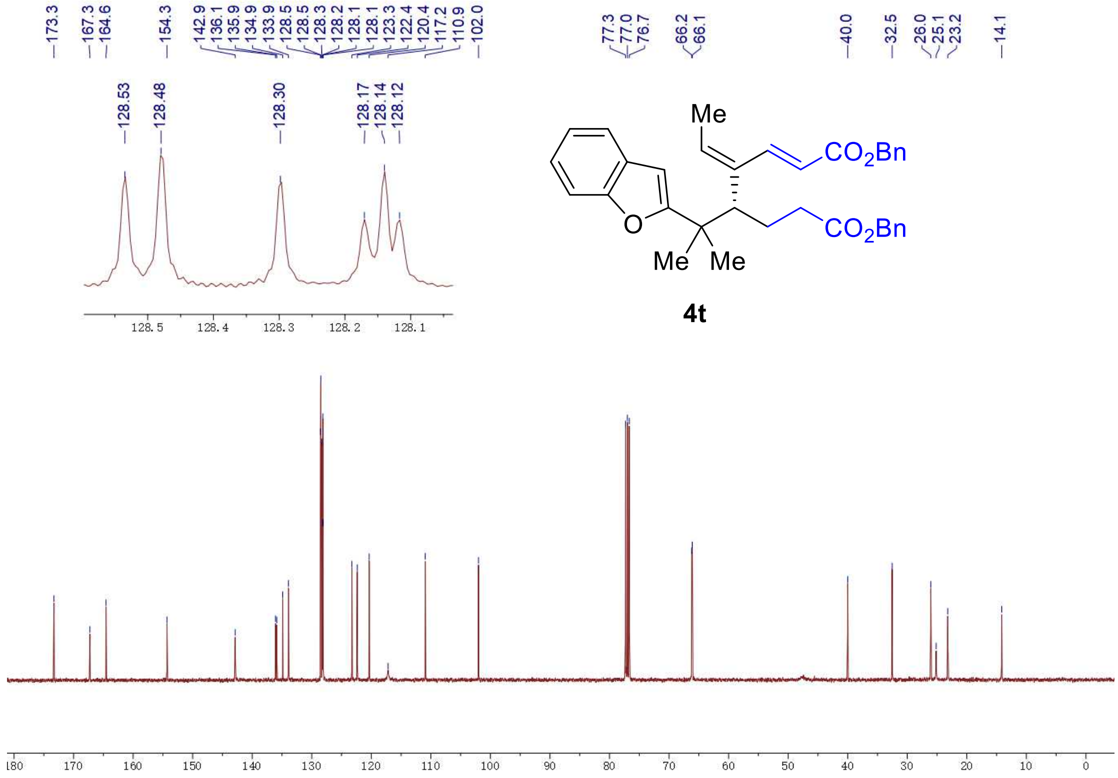

Supplementary Figure 25. ^{1}H NMR and ^{13}C NMR spectrum of compound of **4u**

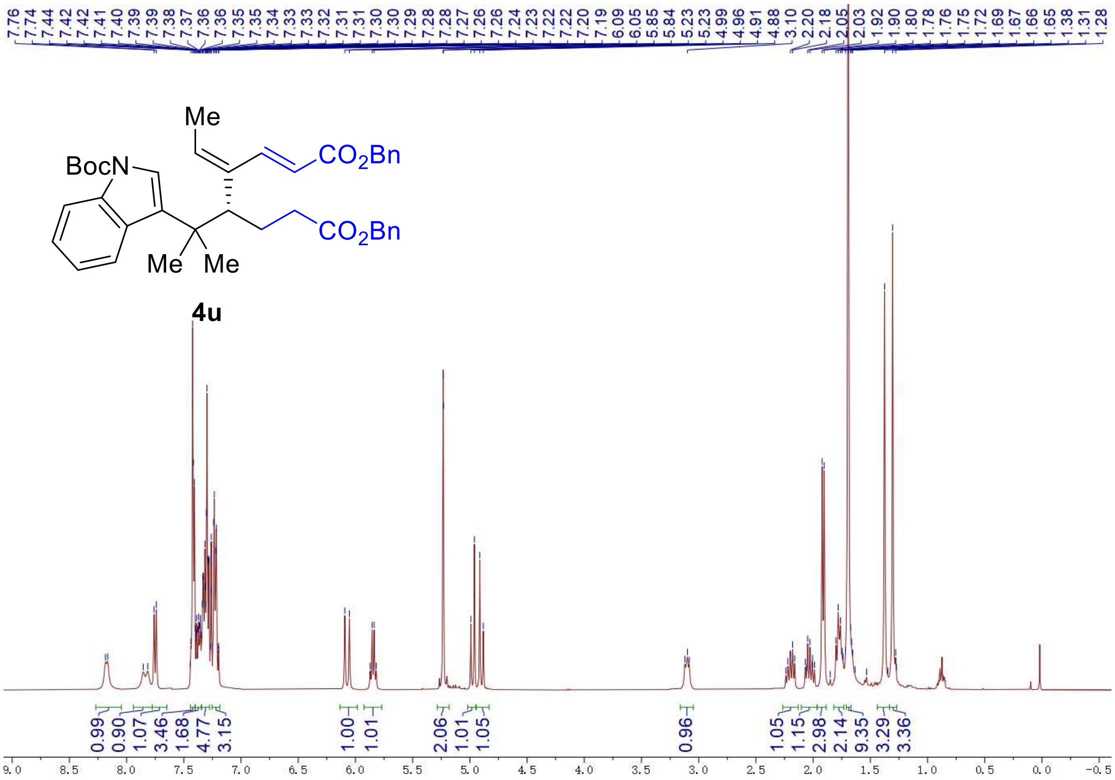

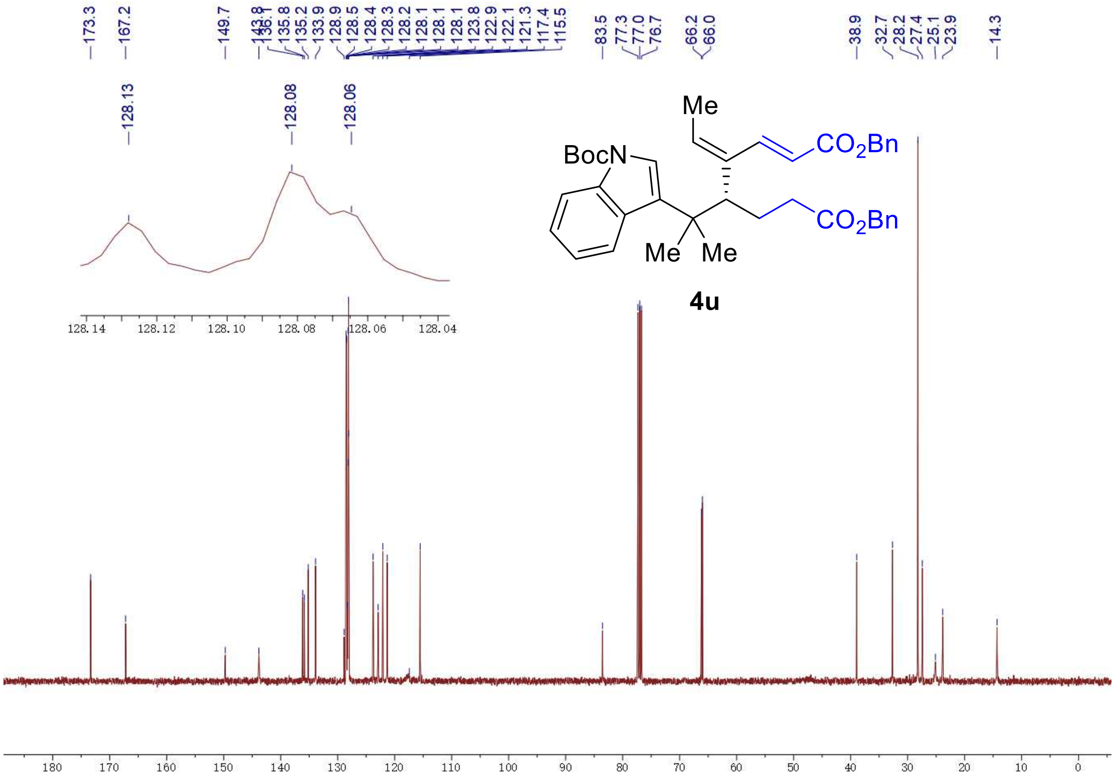

Supplementary Figure 26. ^{1}H NMR and ^{13}C NMR spectrum of compound of **4v**

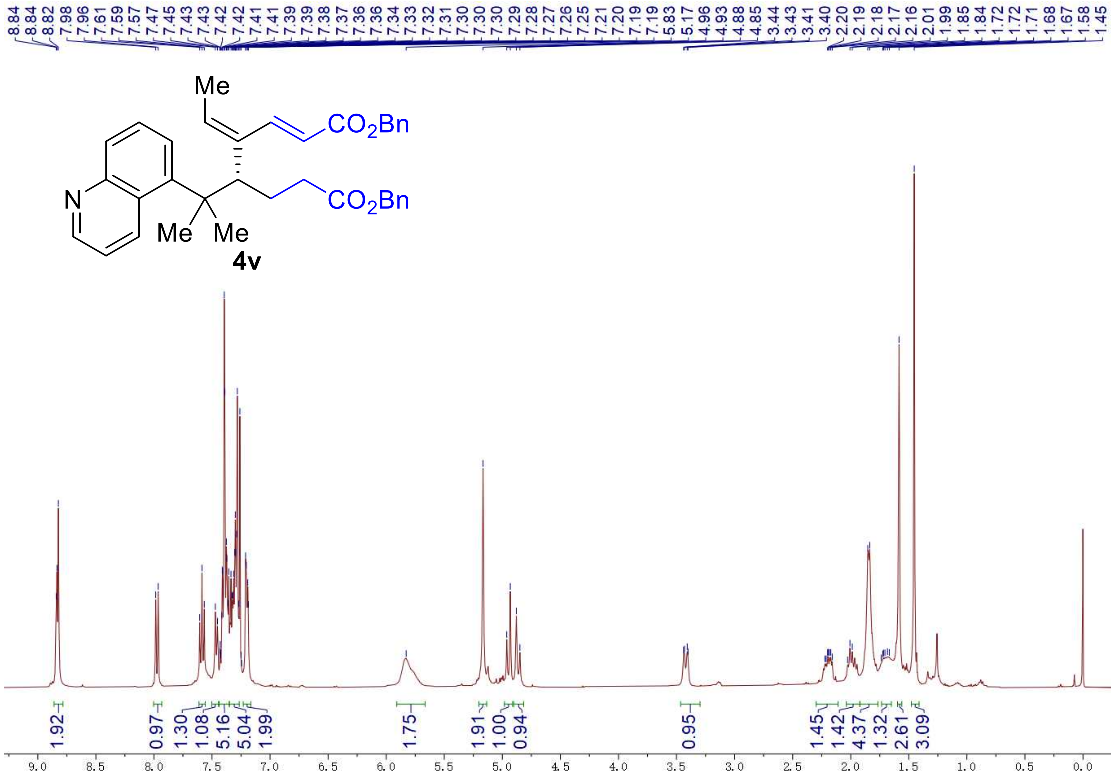

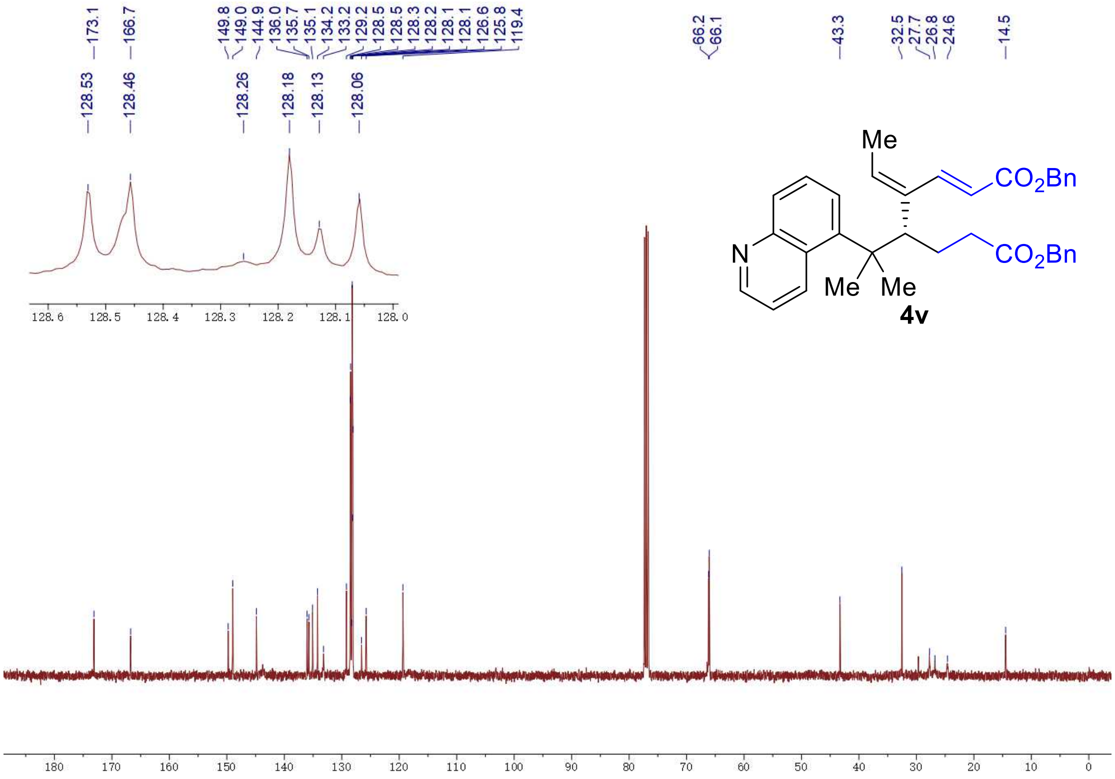

Supplementary Figure 27. ^{1}H NMR and ^{13}C NMR spectrum of compound of **4w**

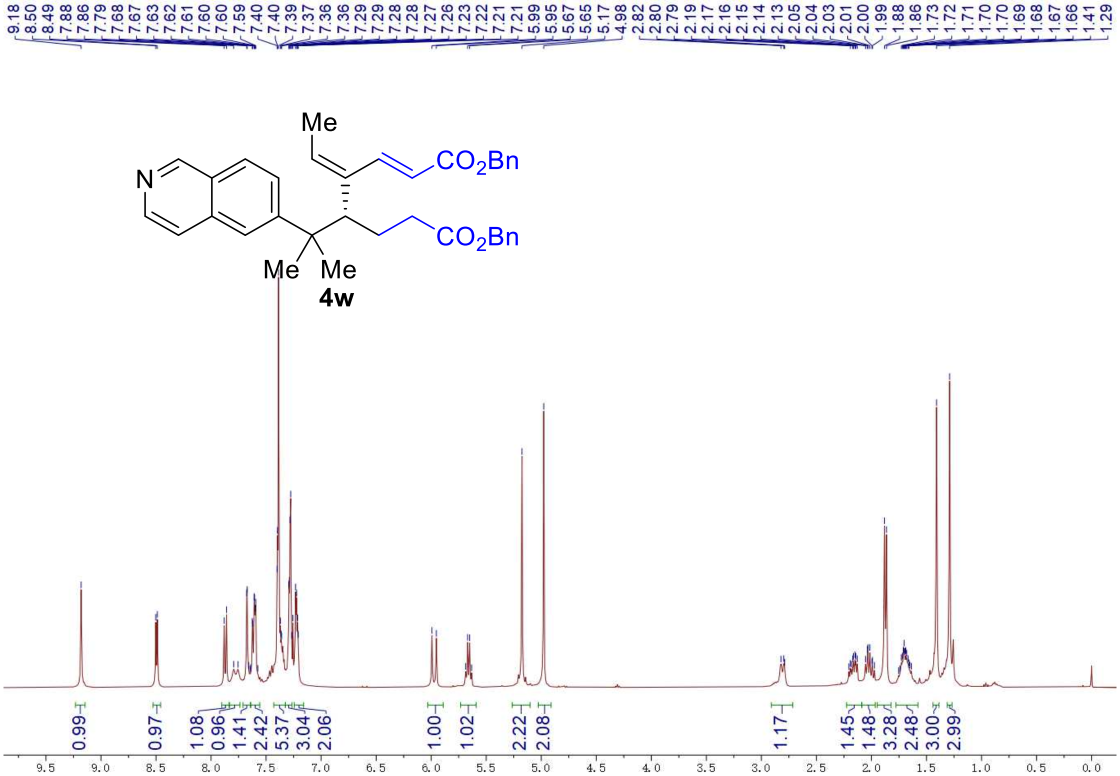

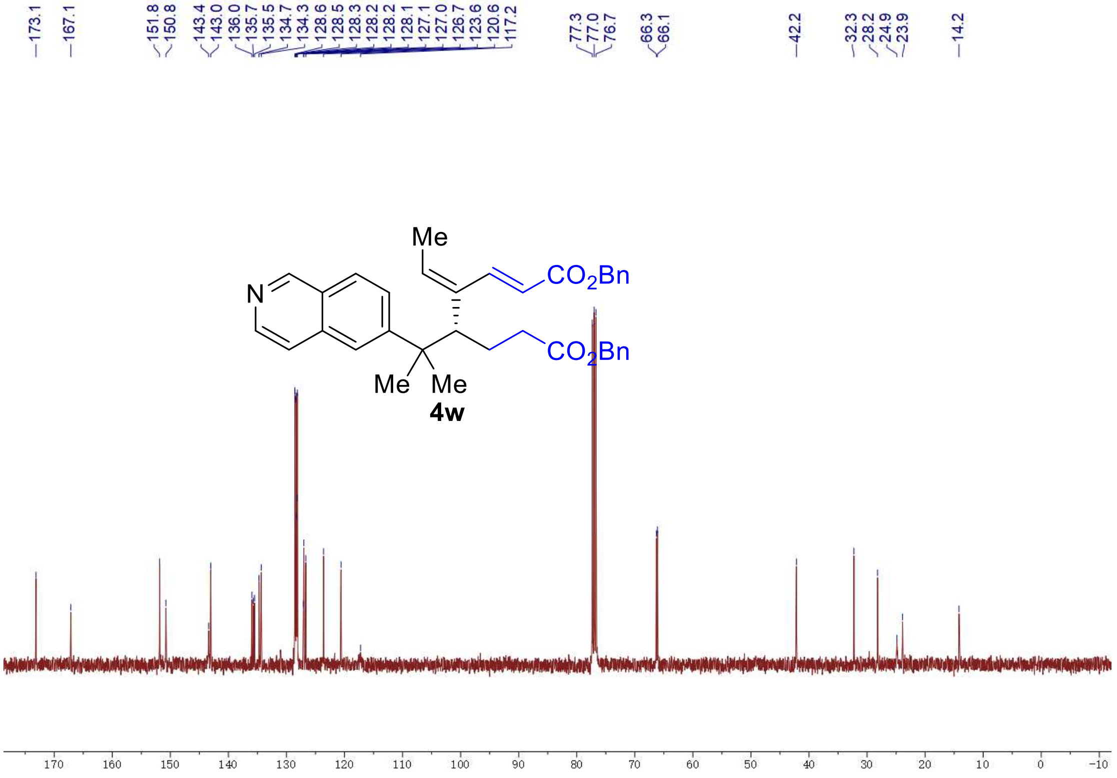

Supplementary Figure 28. ^{1}H NMR and ^{13}C NMR spectrum of compound of **4x**

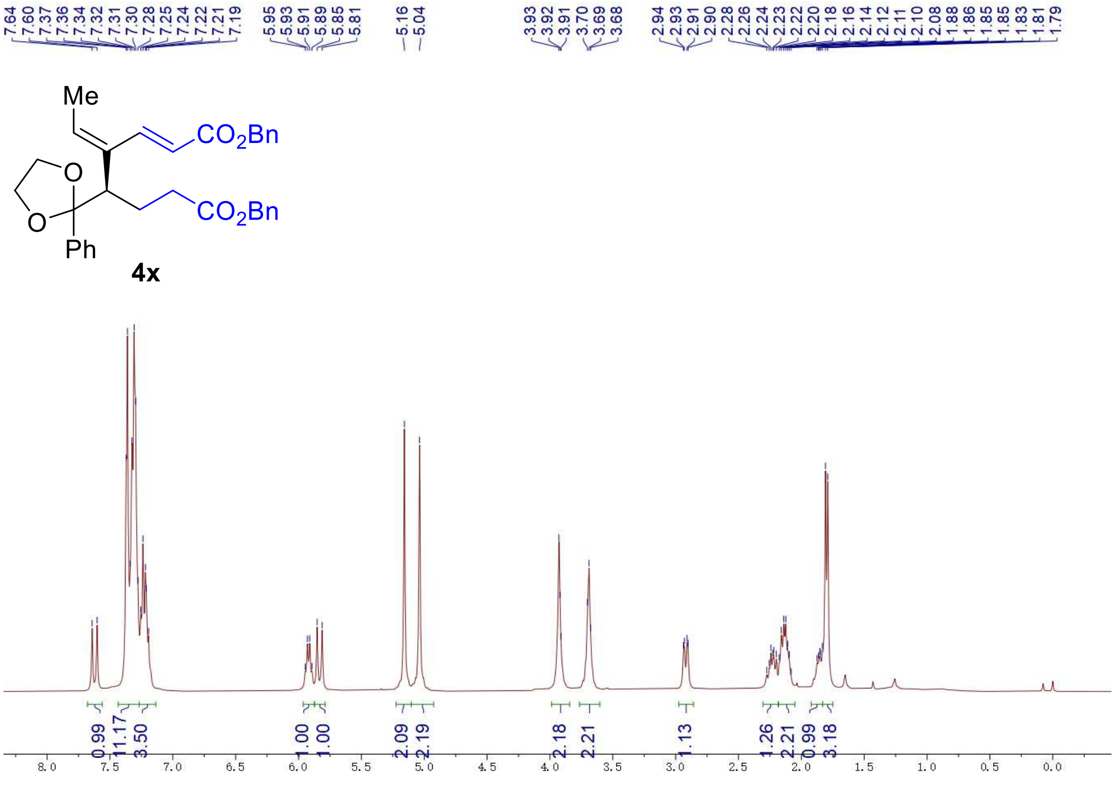

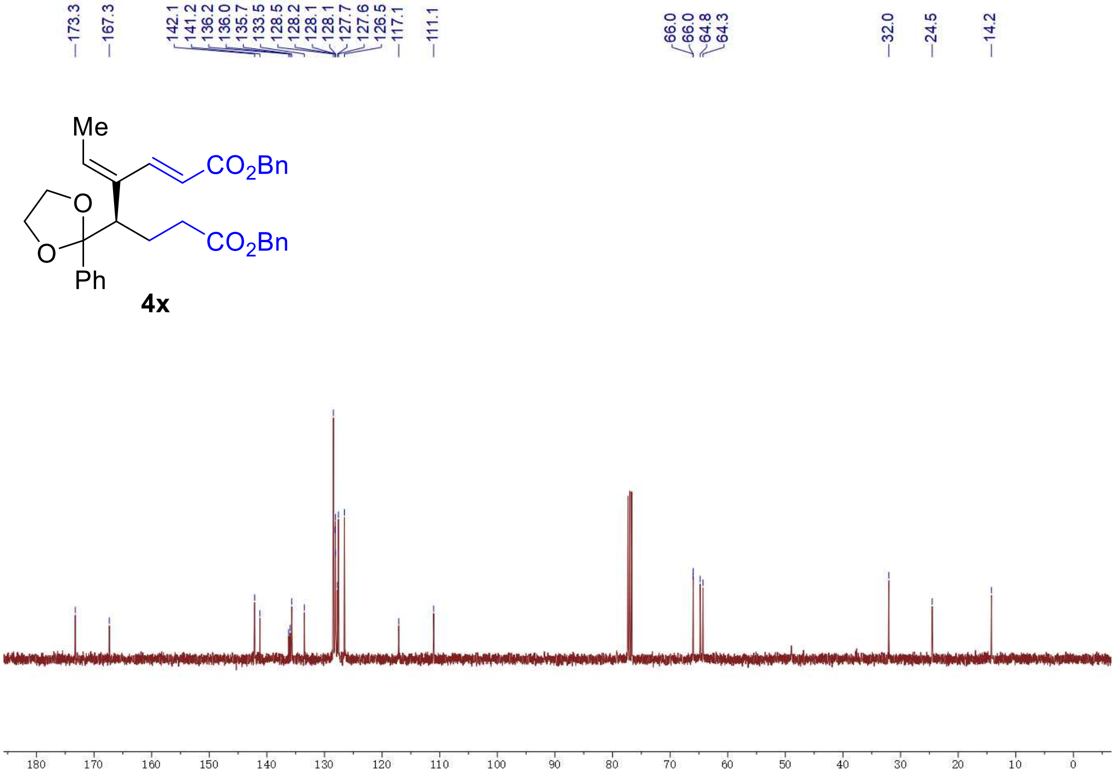

Supplementary Figure 29. ^{1}**H NMR and** ^{13}**C NMR** spectrum of compound of **4y**

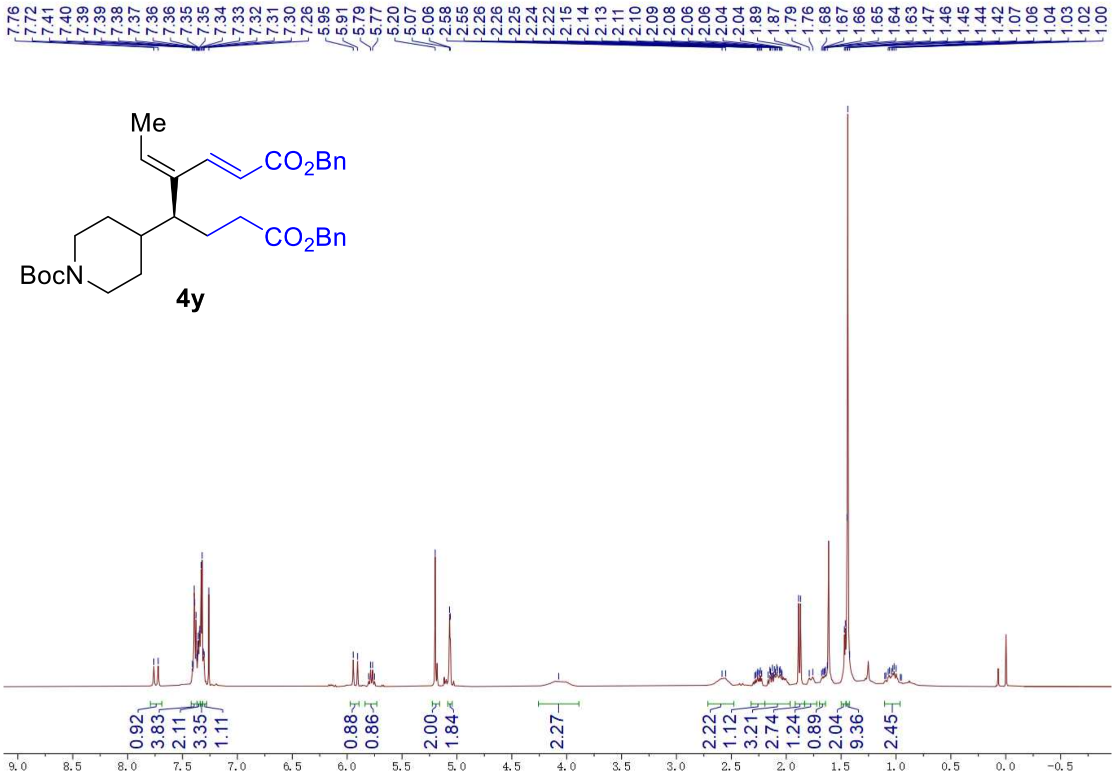

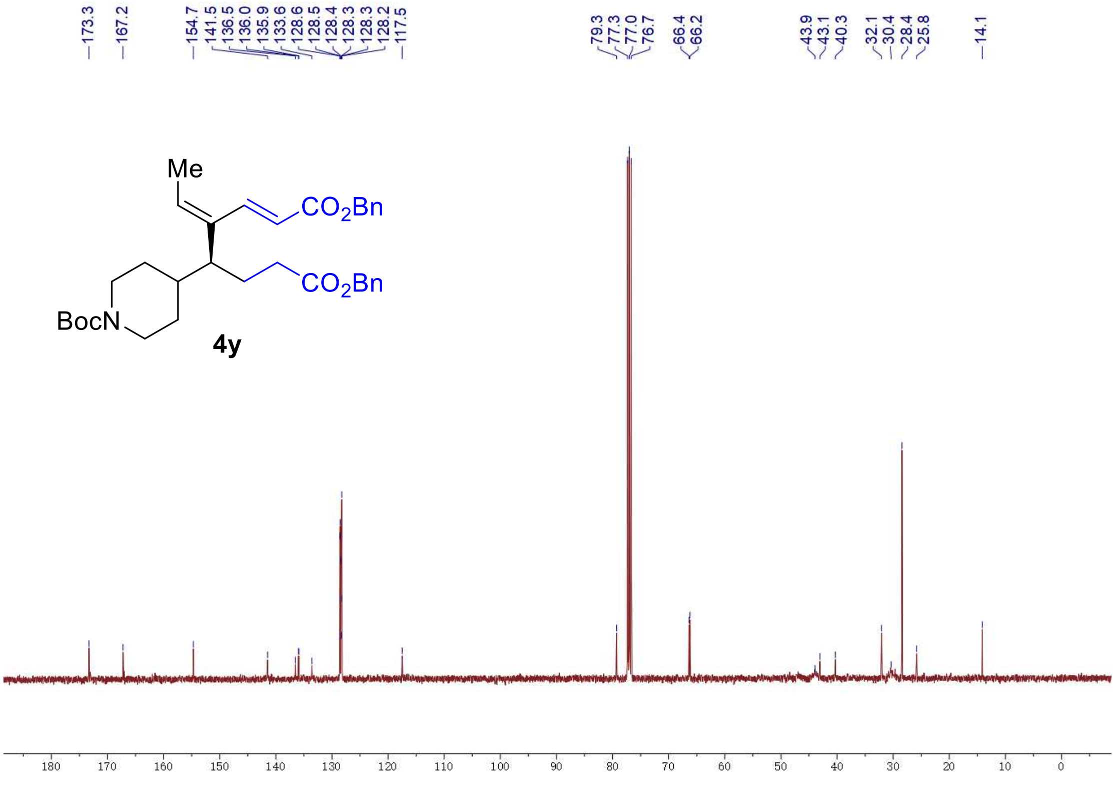

Supplementary Figure 30. ^{1}H NMR and ^{13}C NMR spectrum of compound of **4z**

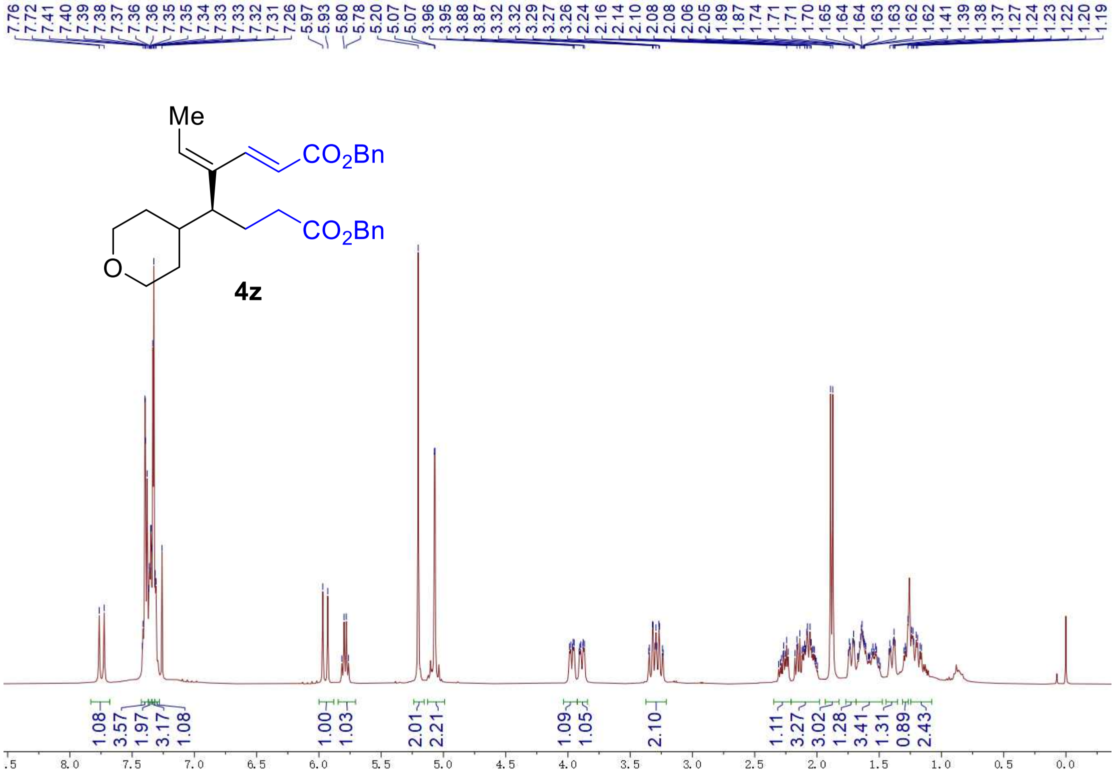

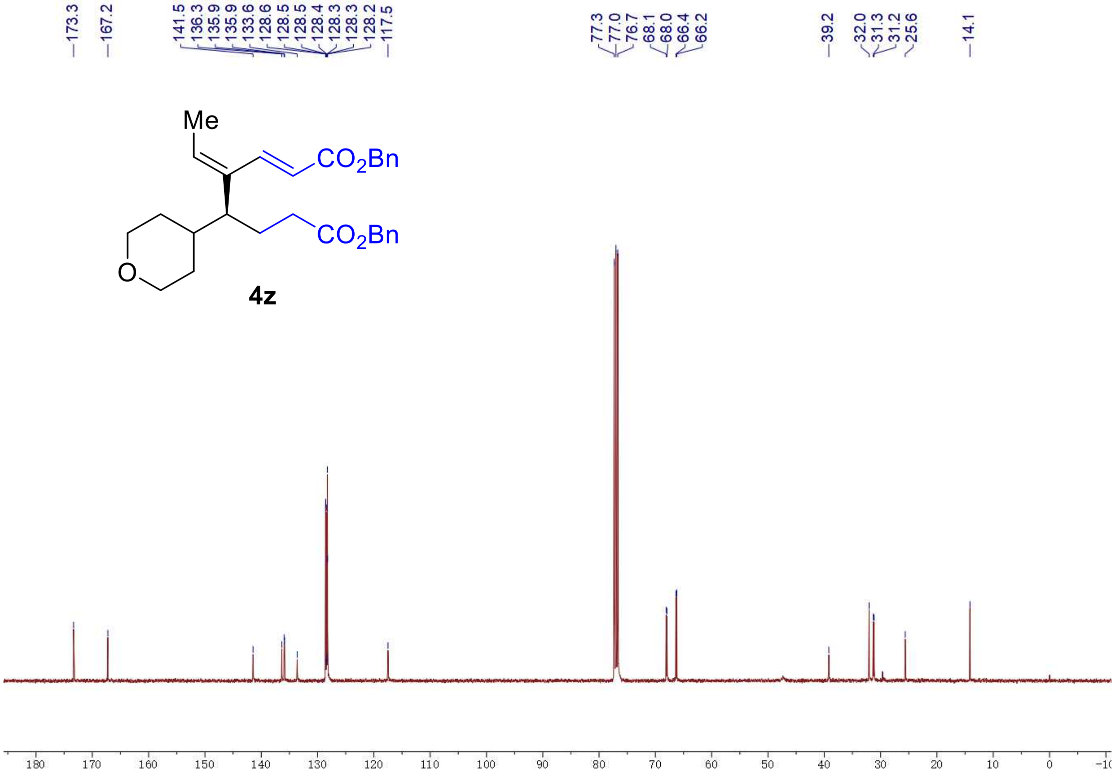

Supplementary Figure 31. **^{1}H NMR and ^{13}C NMR** spectrum of compound of **4aa**

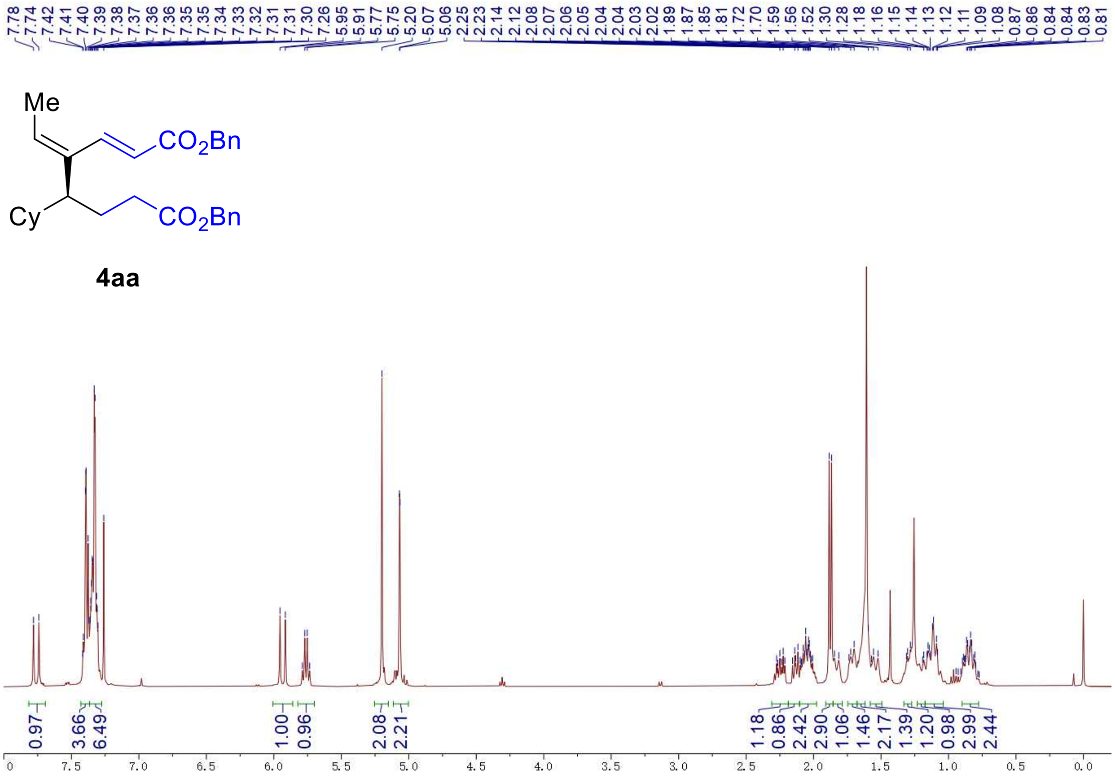

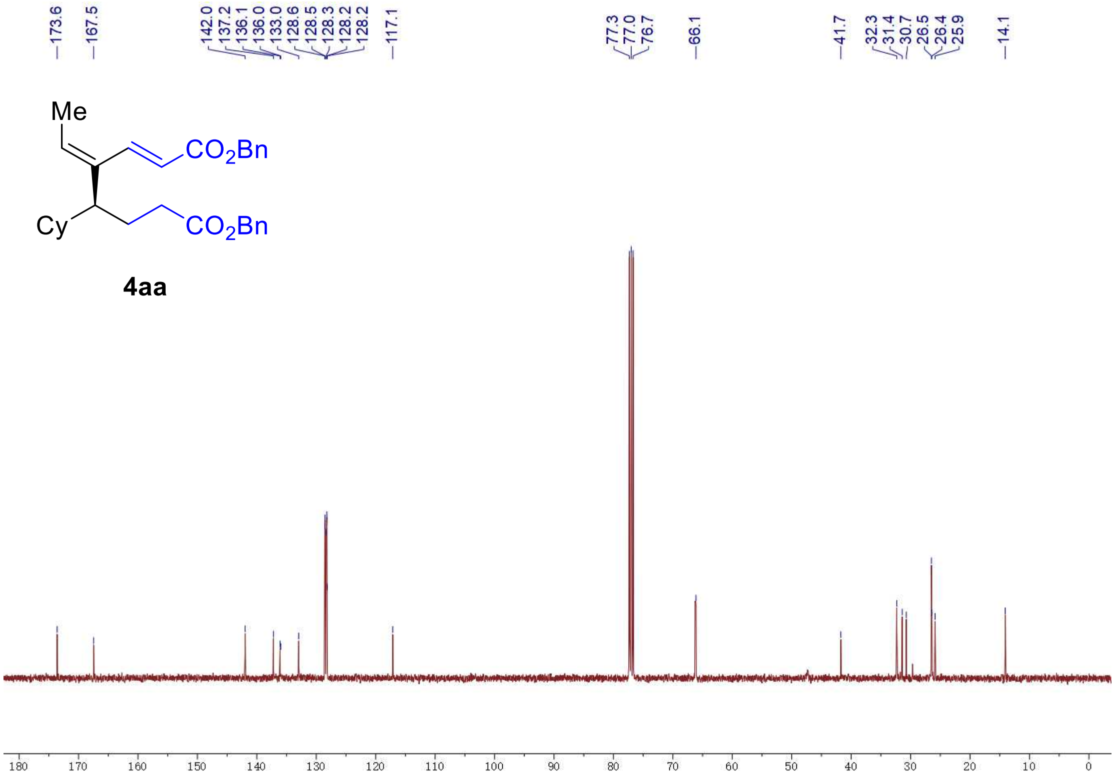

Supplementary Figure 32. **^{1}H NMR and ^{13}C NMR** spectrum of compound of **4ab**

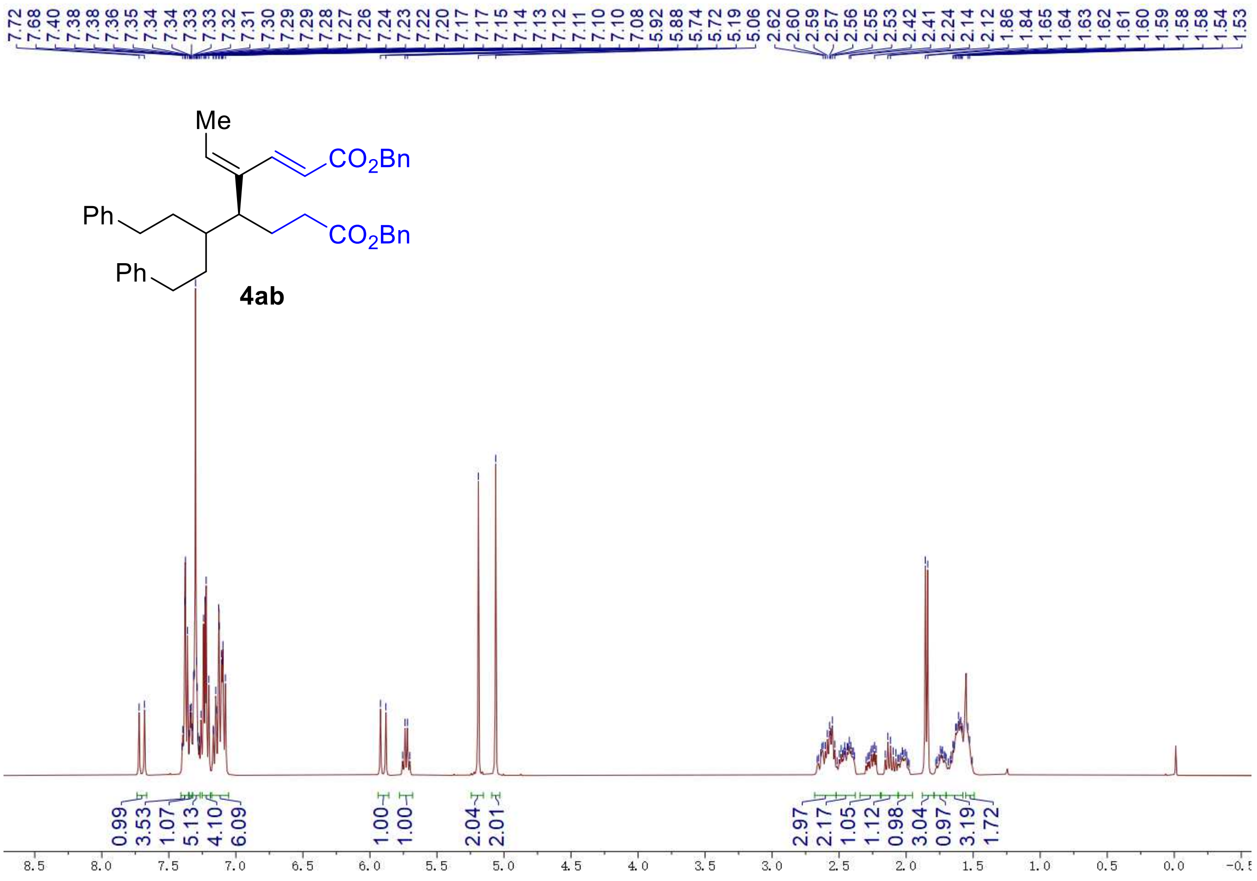

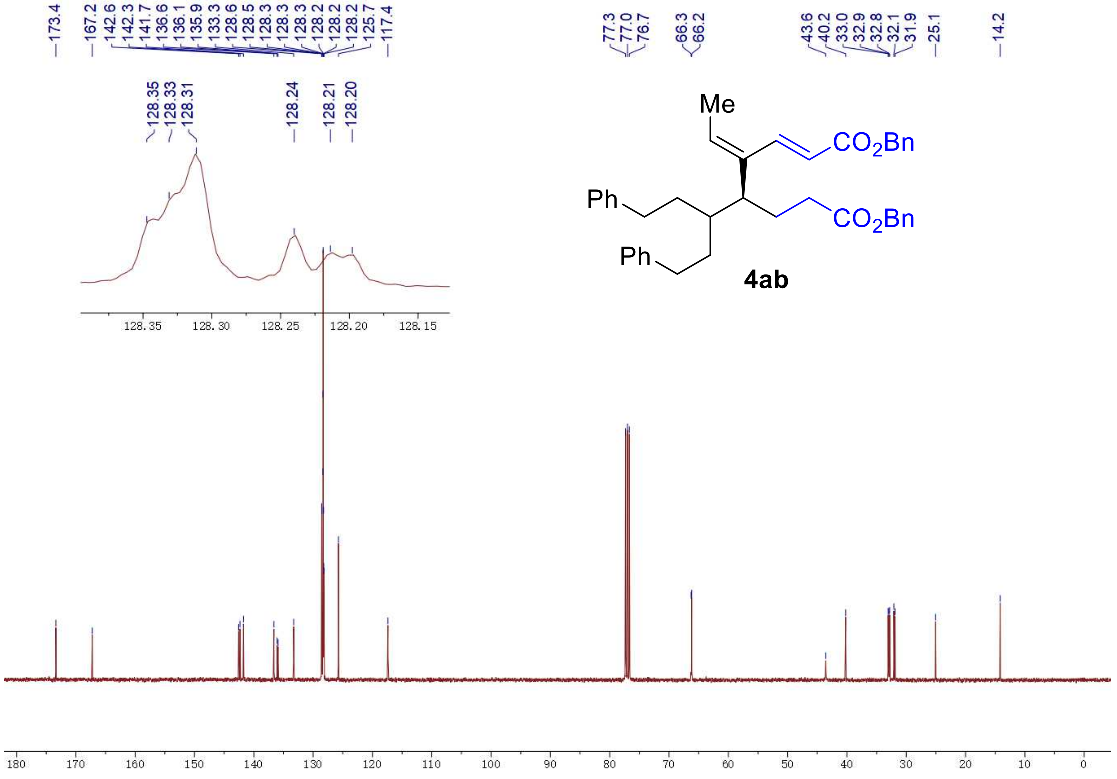

Supplementary Figure 33. ^{1}H NMR and ^{13}C NMR spectrum of compound of **4ac**

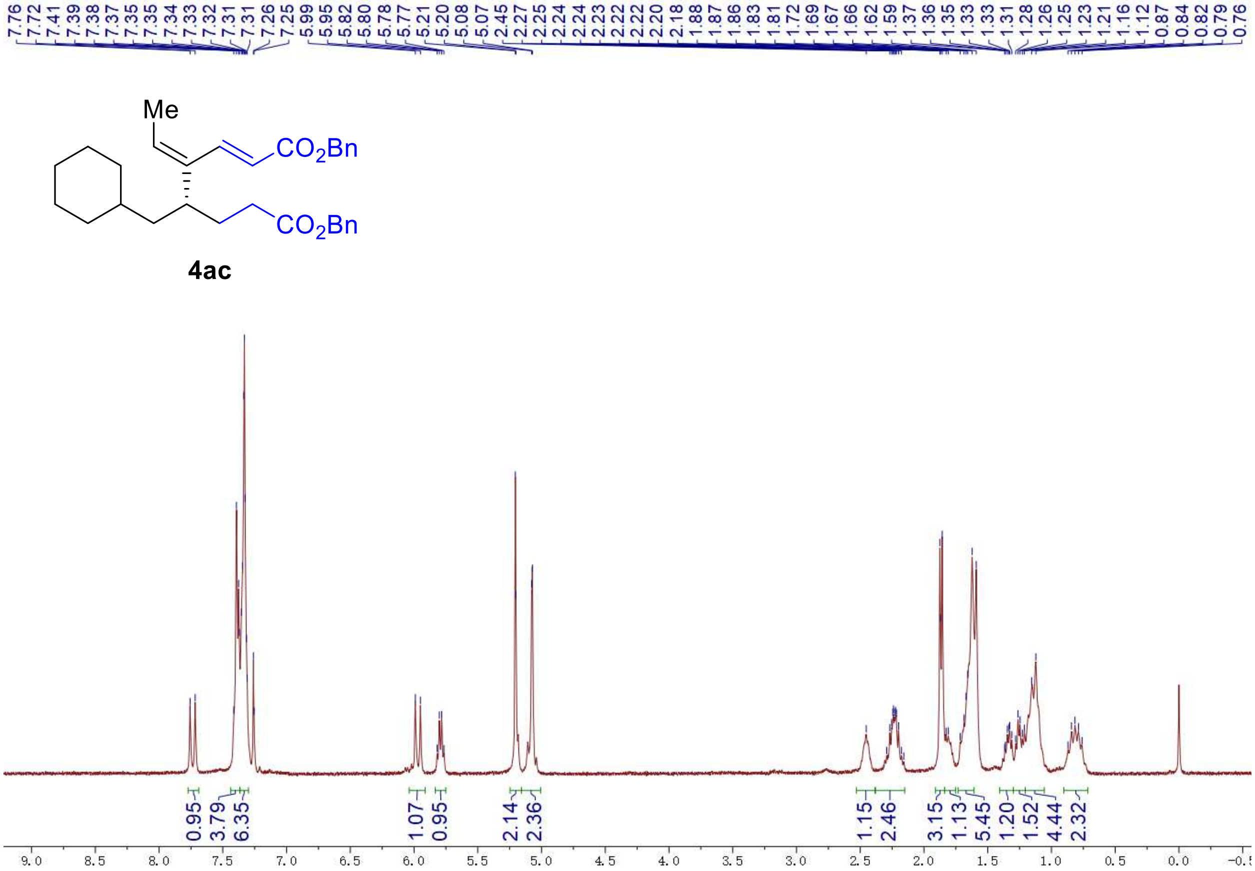

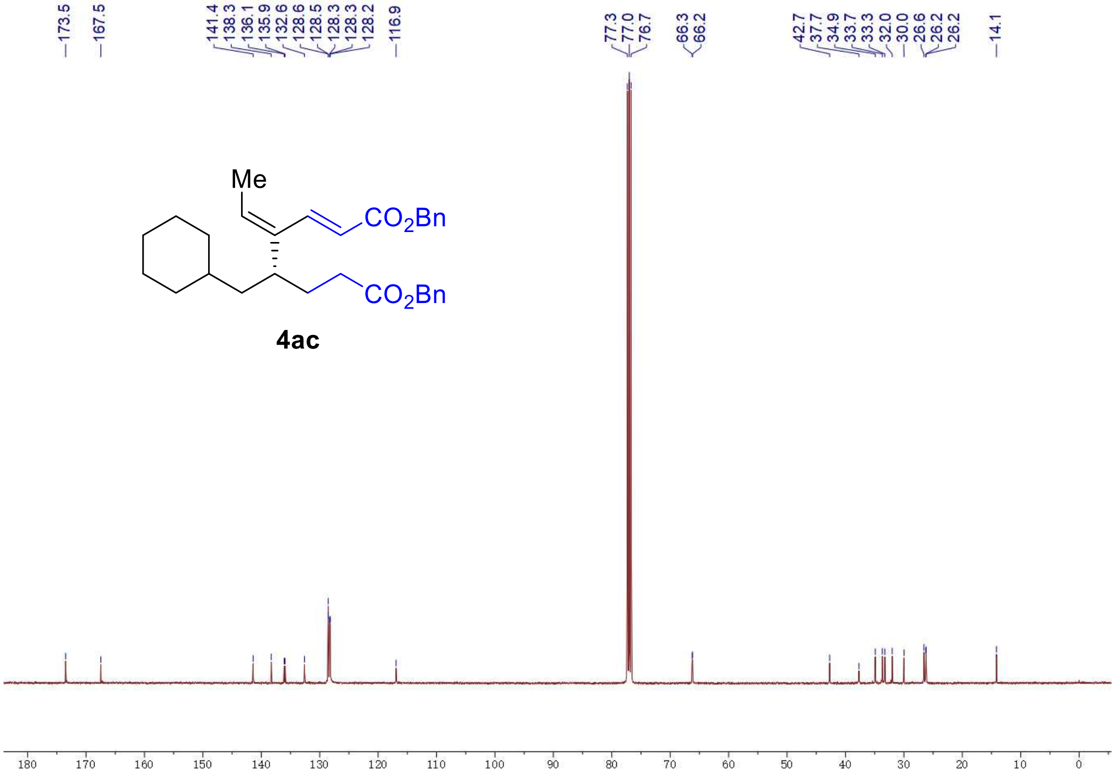

Supplementary Figure 34. ^{1}H NMR and ^{13}C NMR spectrum of compound of **5a**

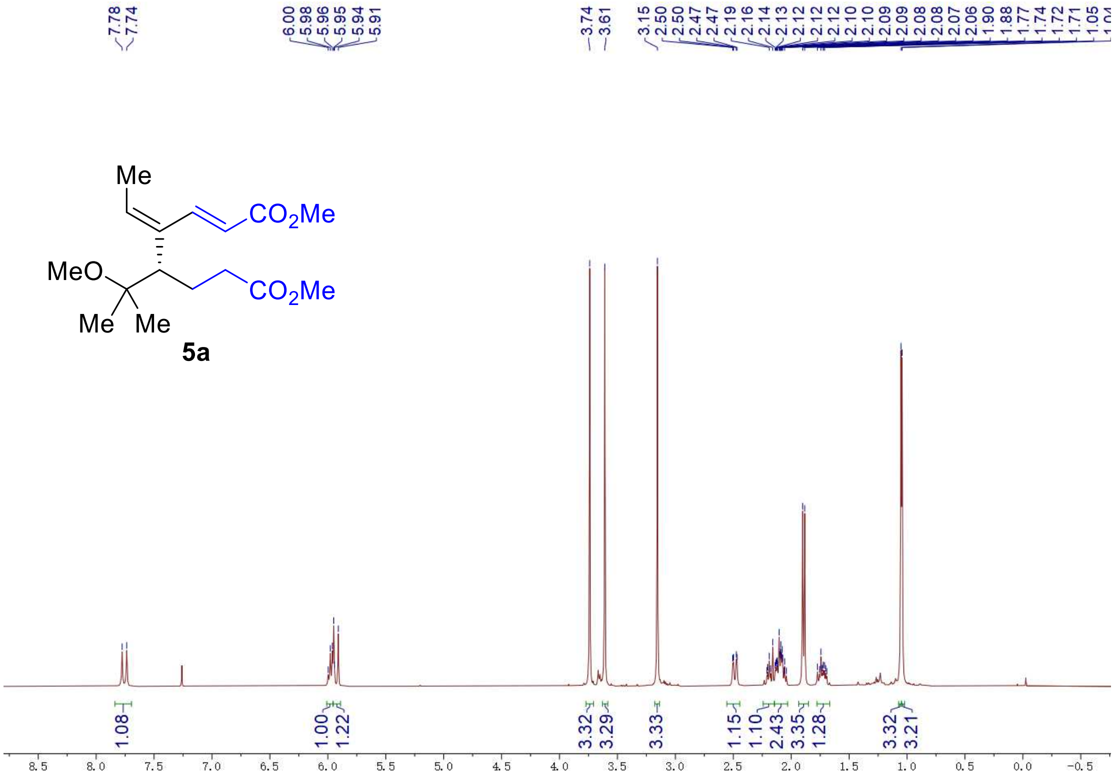

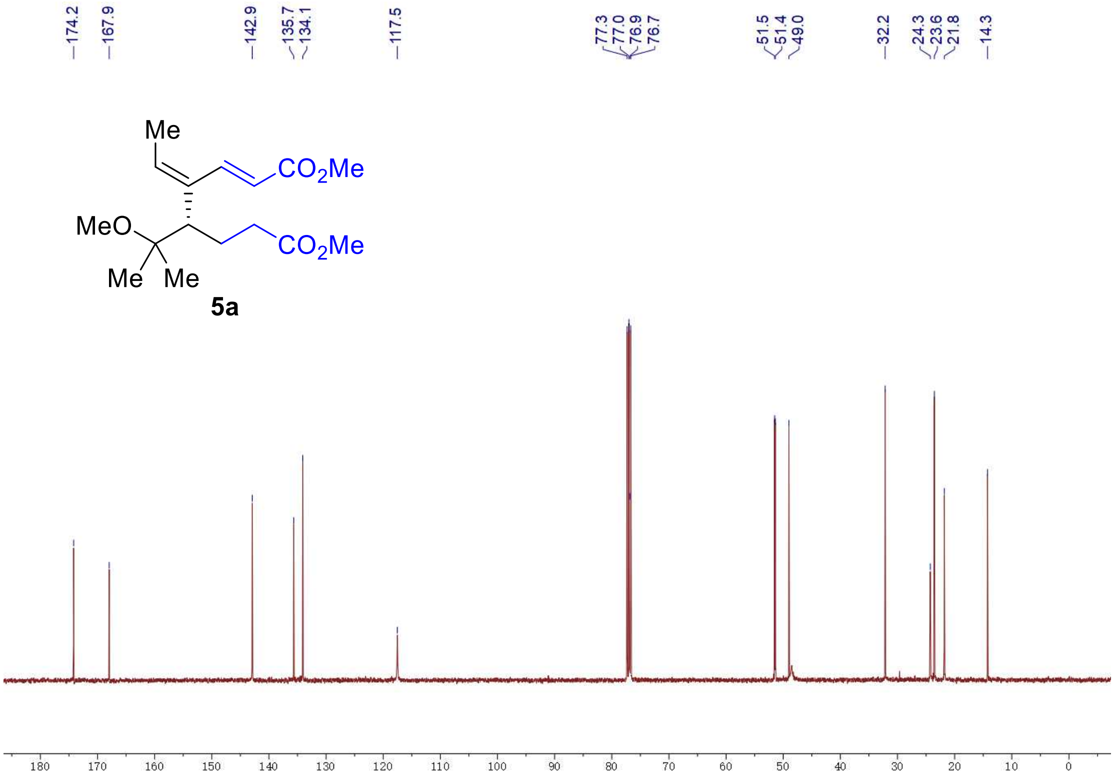

Supplementary Figure 35. ^{1}H NMR and ^{13}C NMR spectrum of compound of **5b**

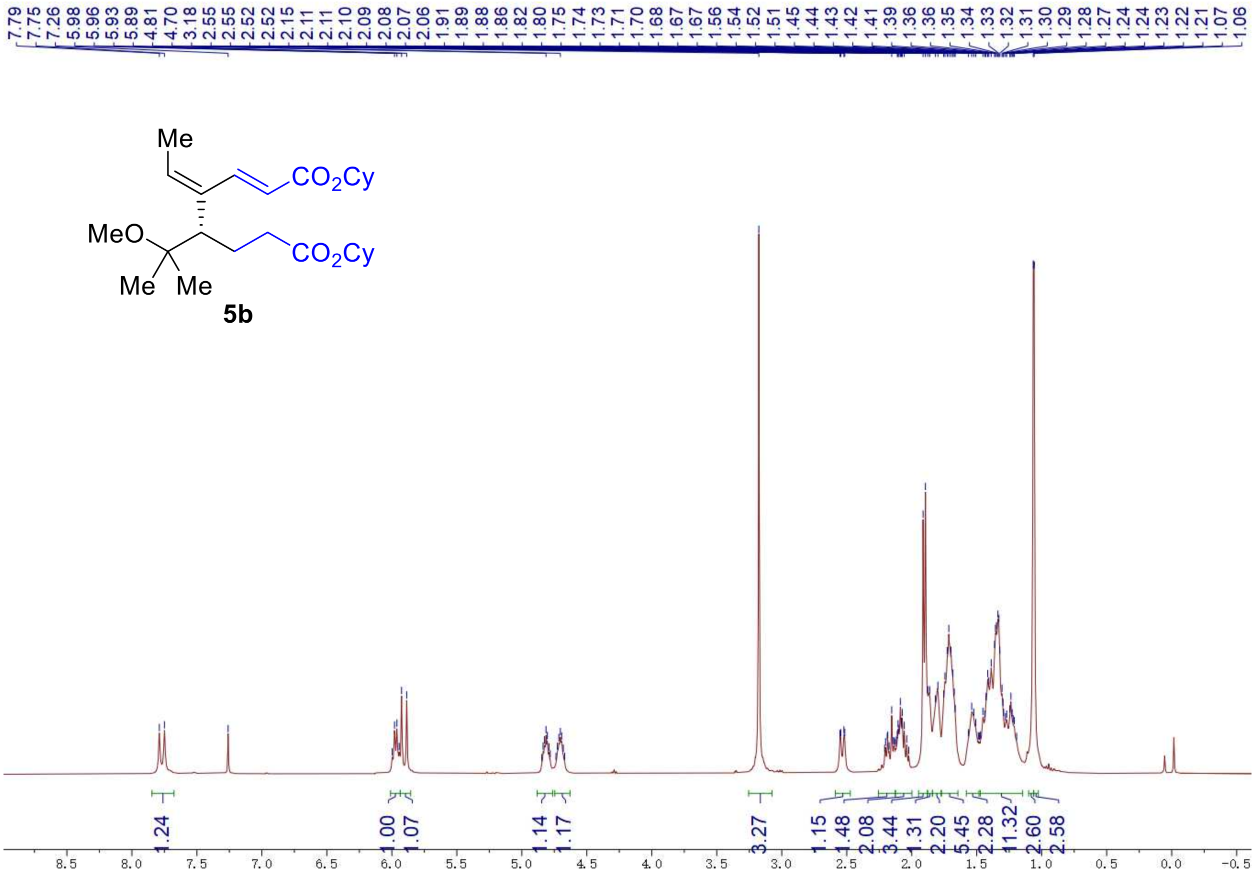

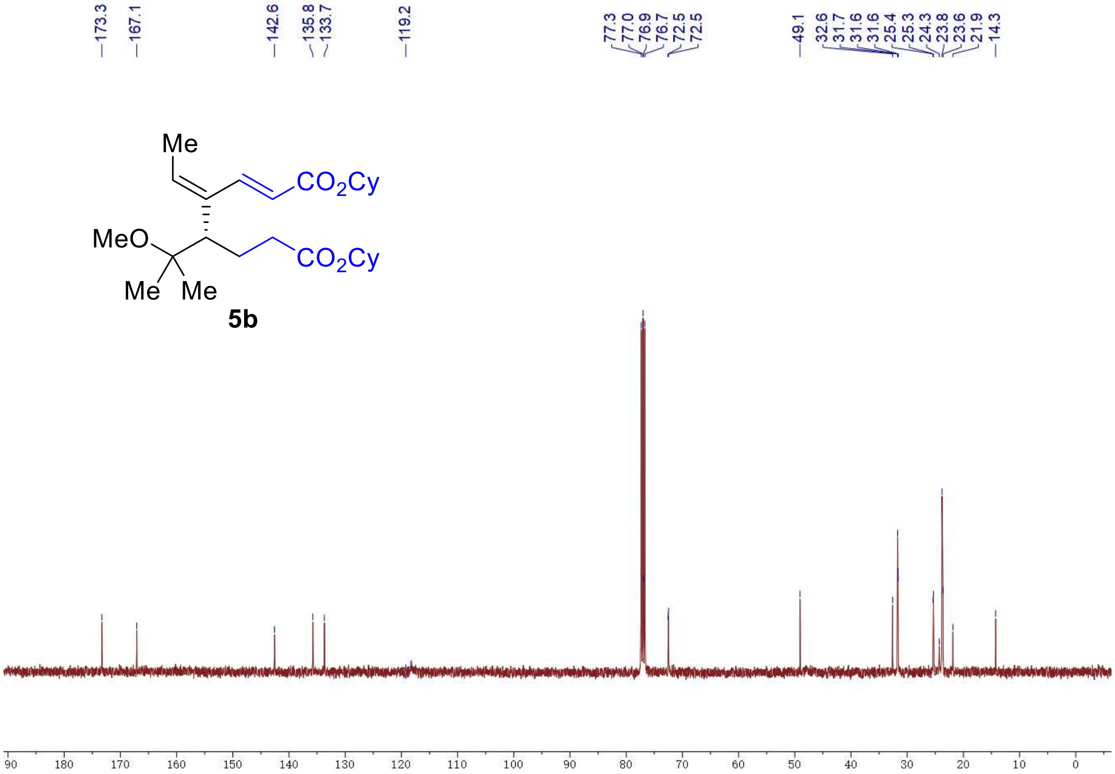

Supplementary Figure 36. ¹H NMR and ¹³C NMR spectrum of compound of **5c**

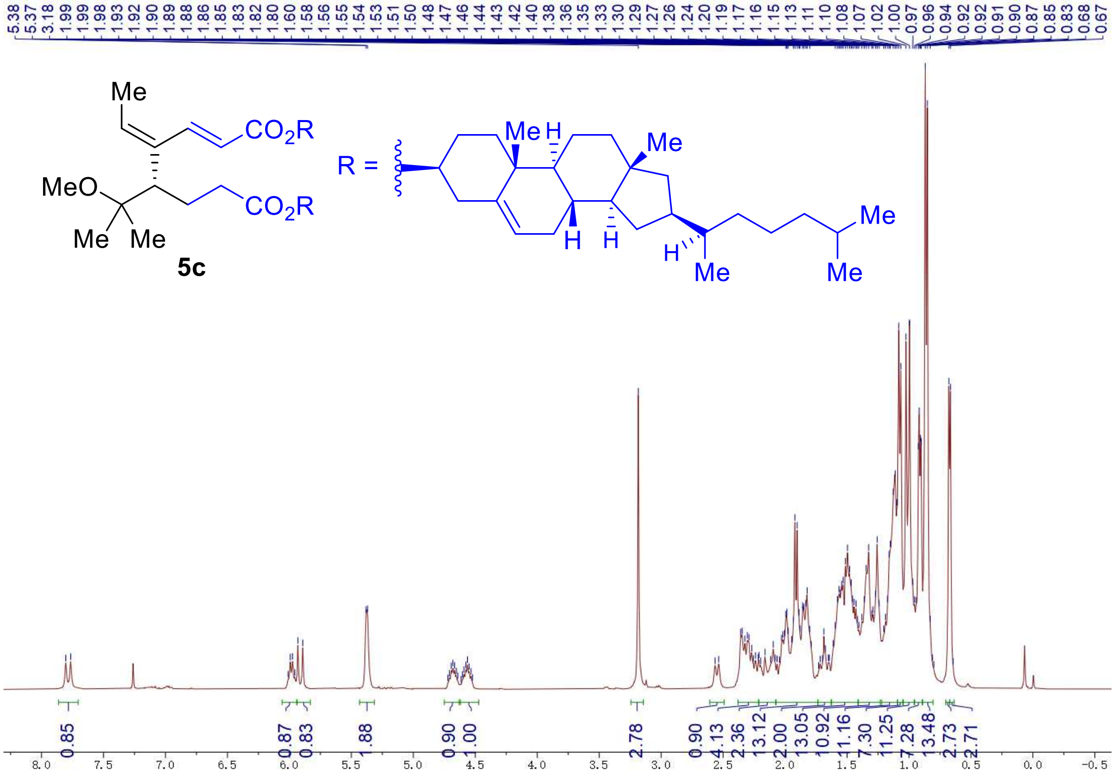

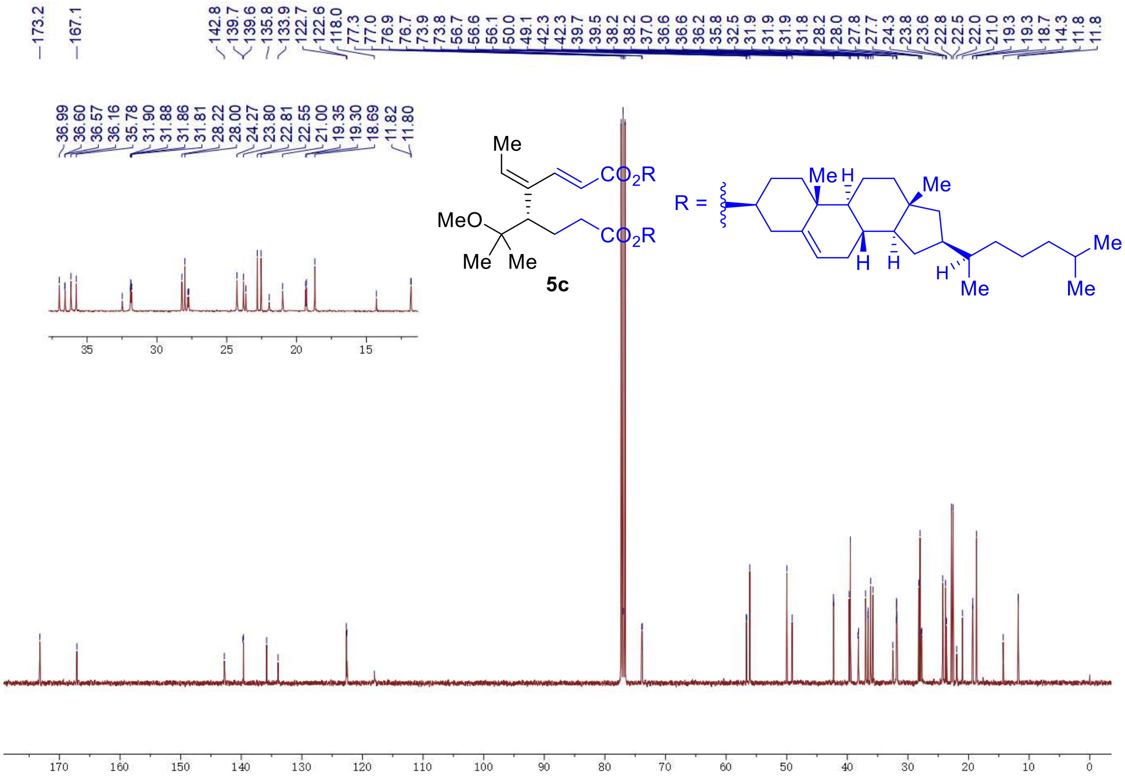

Supplementary Figure 37. **^{1}H NMR** spectrum of compound of **6a** in $CDCl_3$

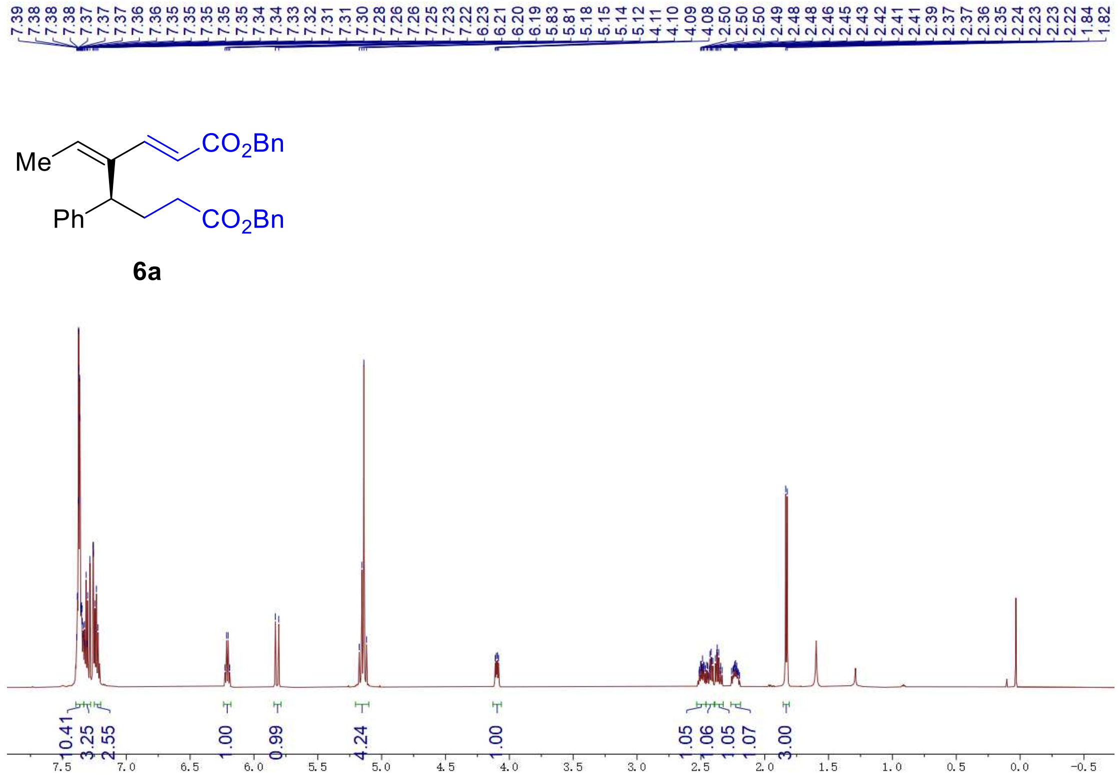

Supplementary Figure 38. **^{1}H NMR** spectrum of compound of **6a** in acetone-d_6

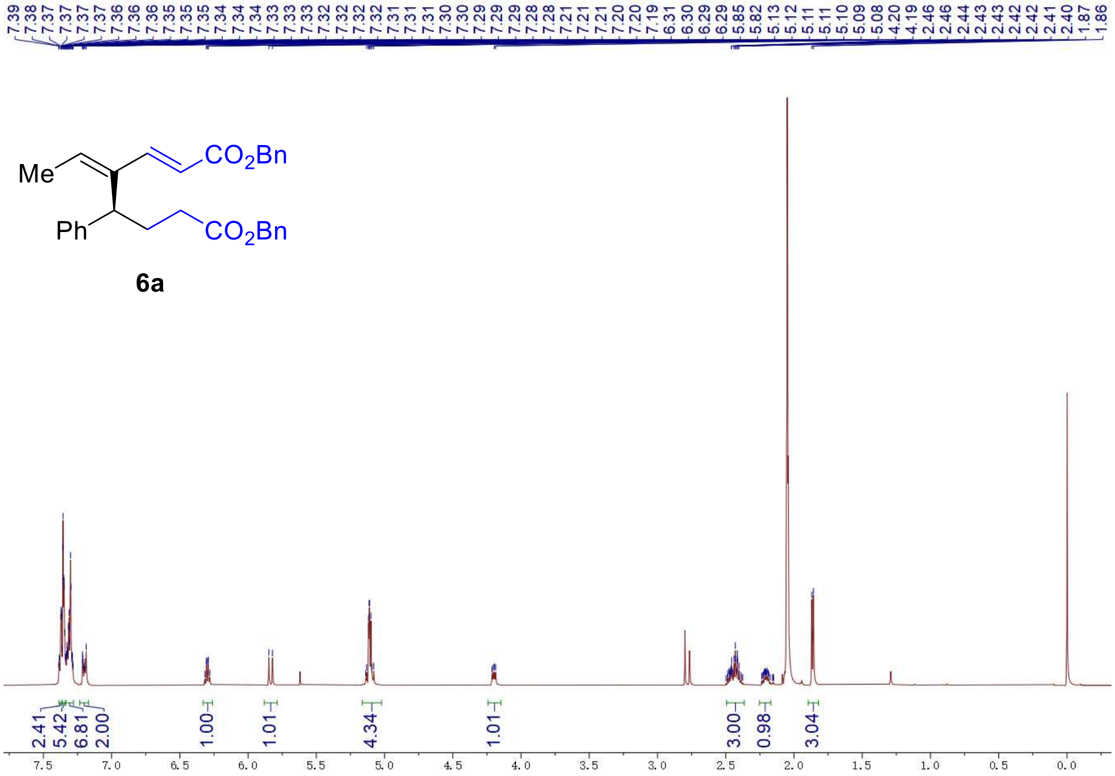

Supplementary Figure 39. H-H cosy spectrum of compound of **6a** in acetone-d_6

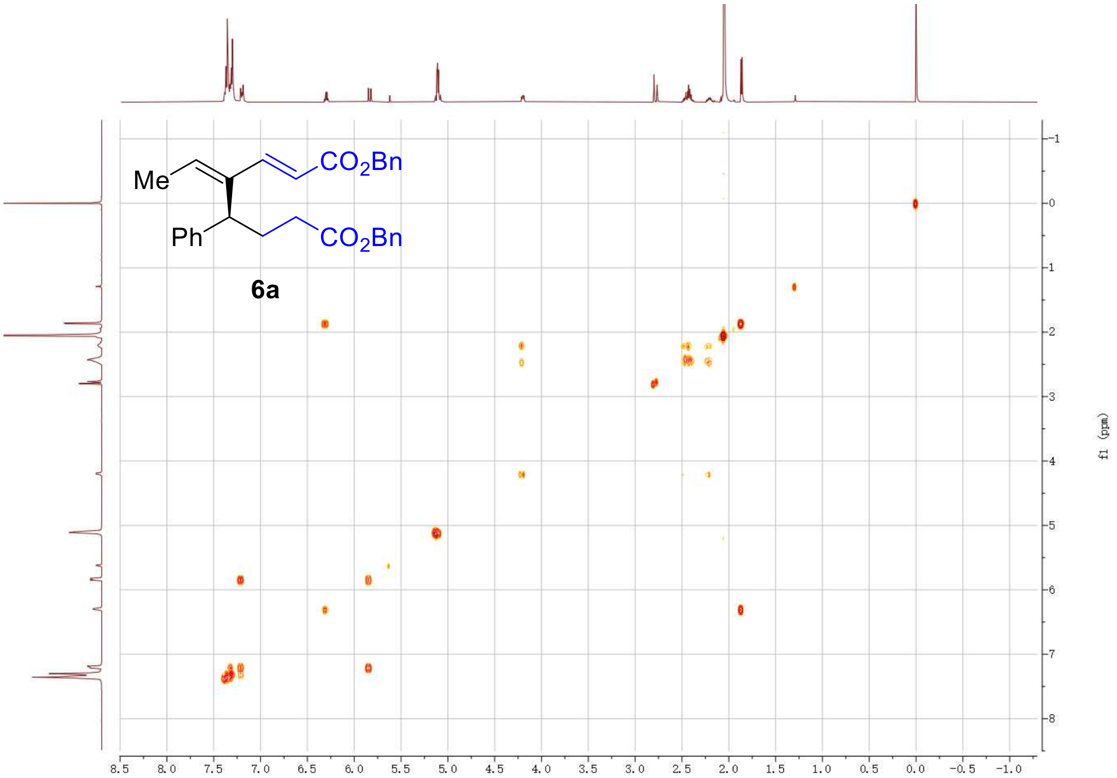

Supplementary Figure 40. H-H cosy spectrum of compound of **6a** in $CDCl_3$

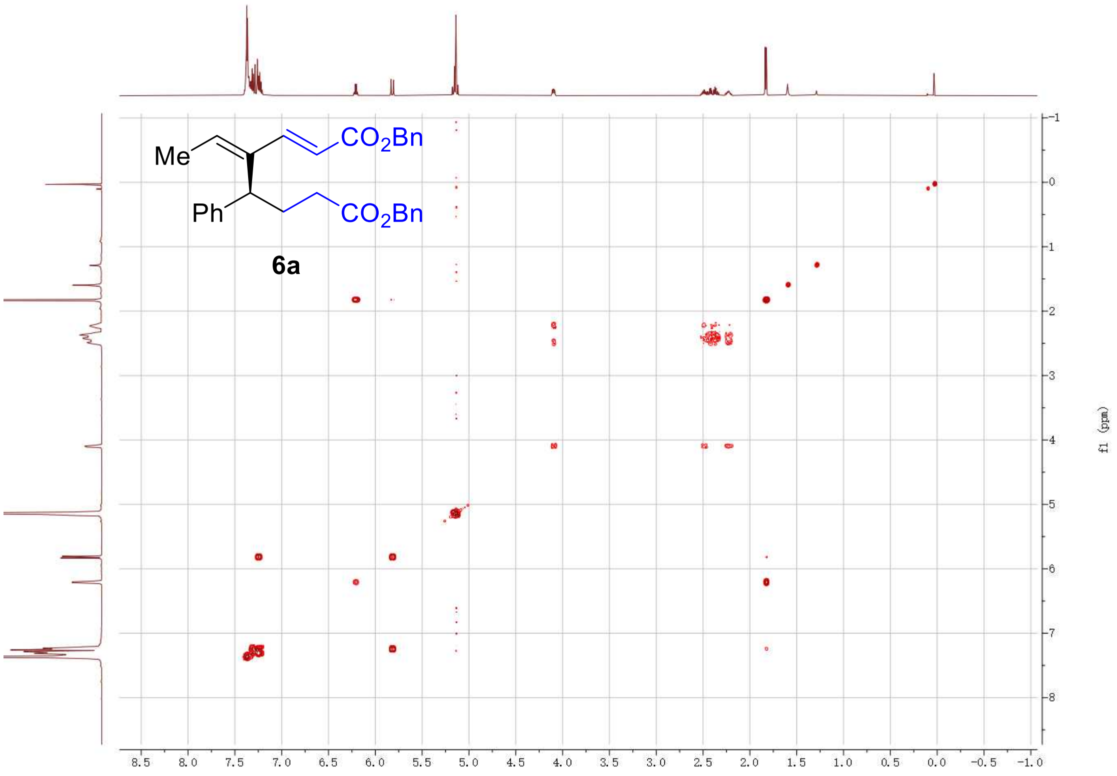

Supplementary Figure 41. **^{13}C NMR** spectrum of compound of **6a**

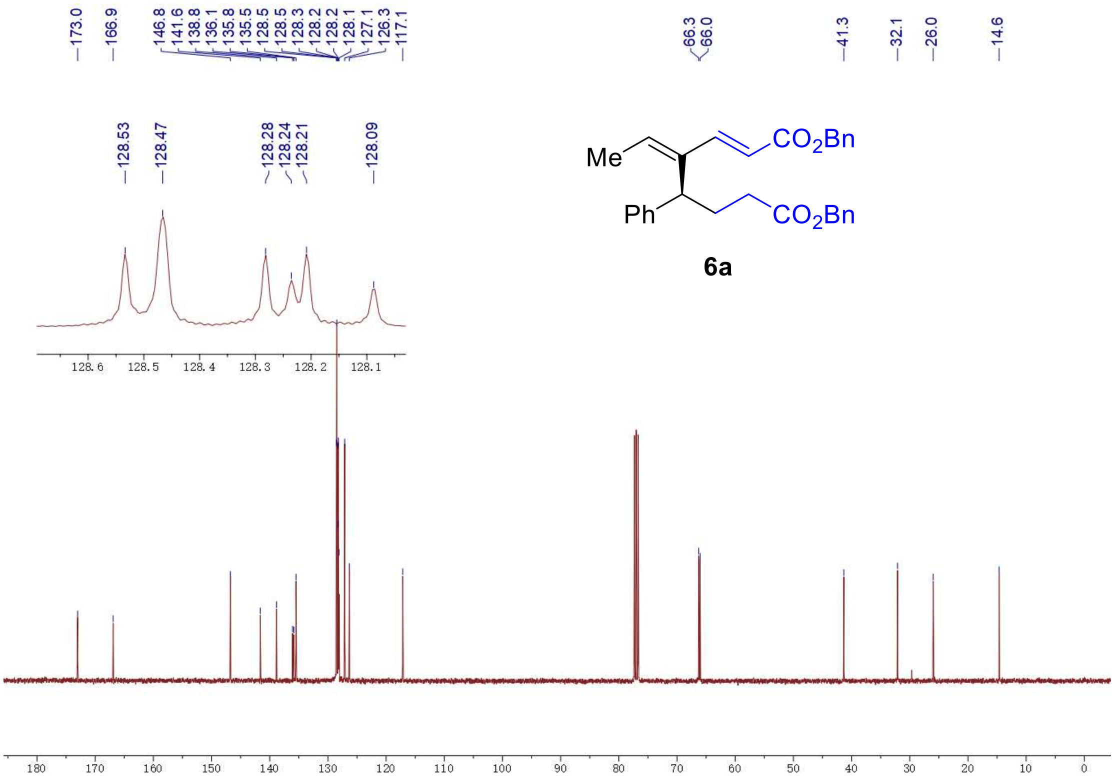

Supplementary Figure 42. **^{1}H NMR and ^{13}C NMR** spectrum of compound of **6b**

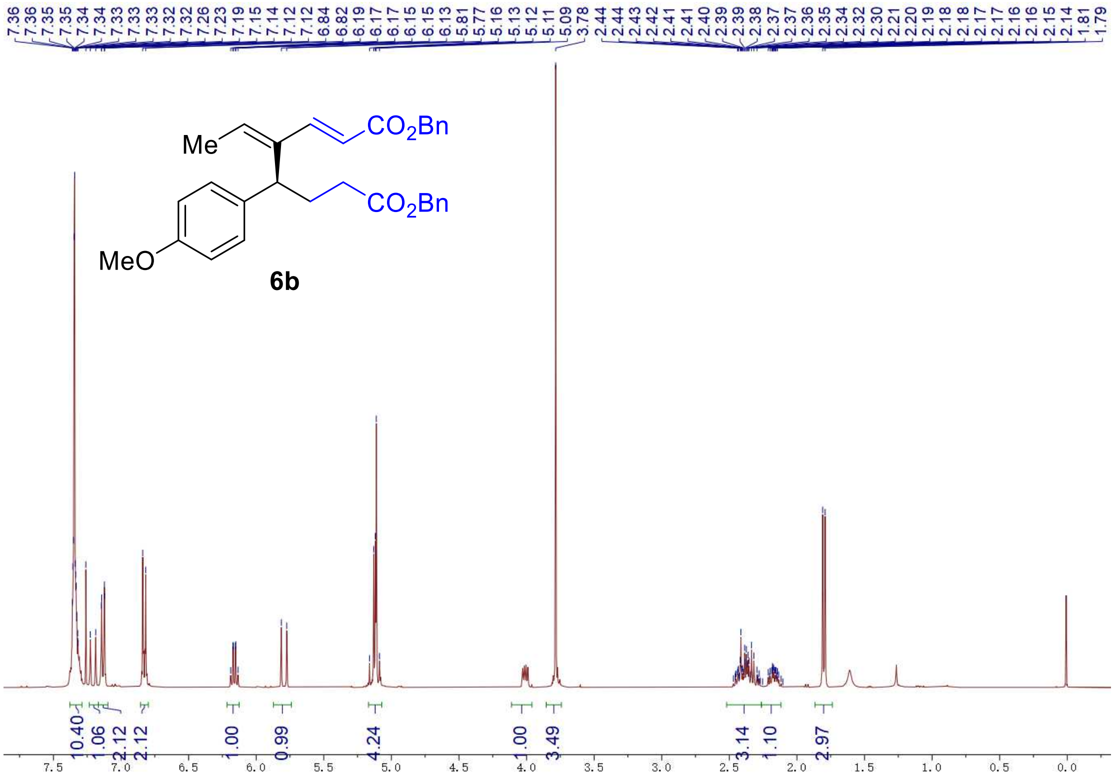

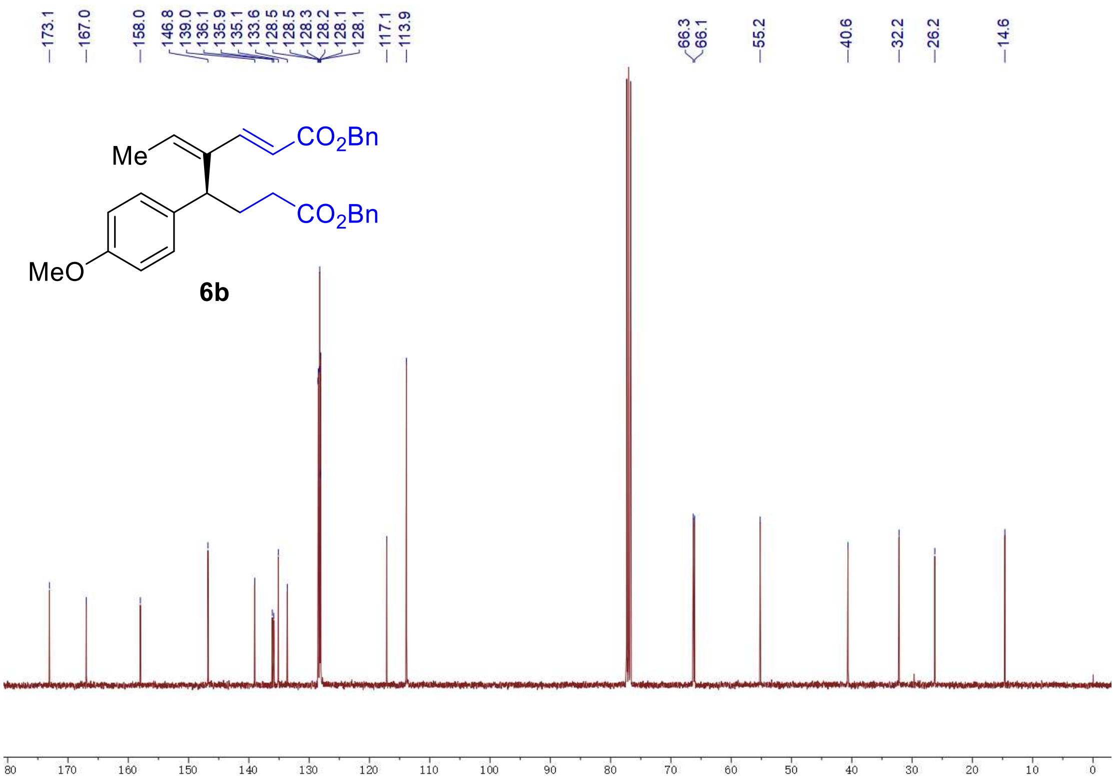

Supplementary Figure 43. ^{1}H NMR and ^{13}C NMR spectrum of compound of **6c**

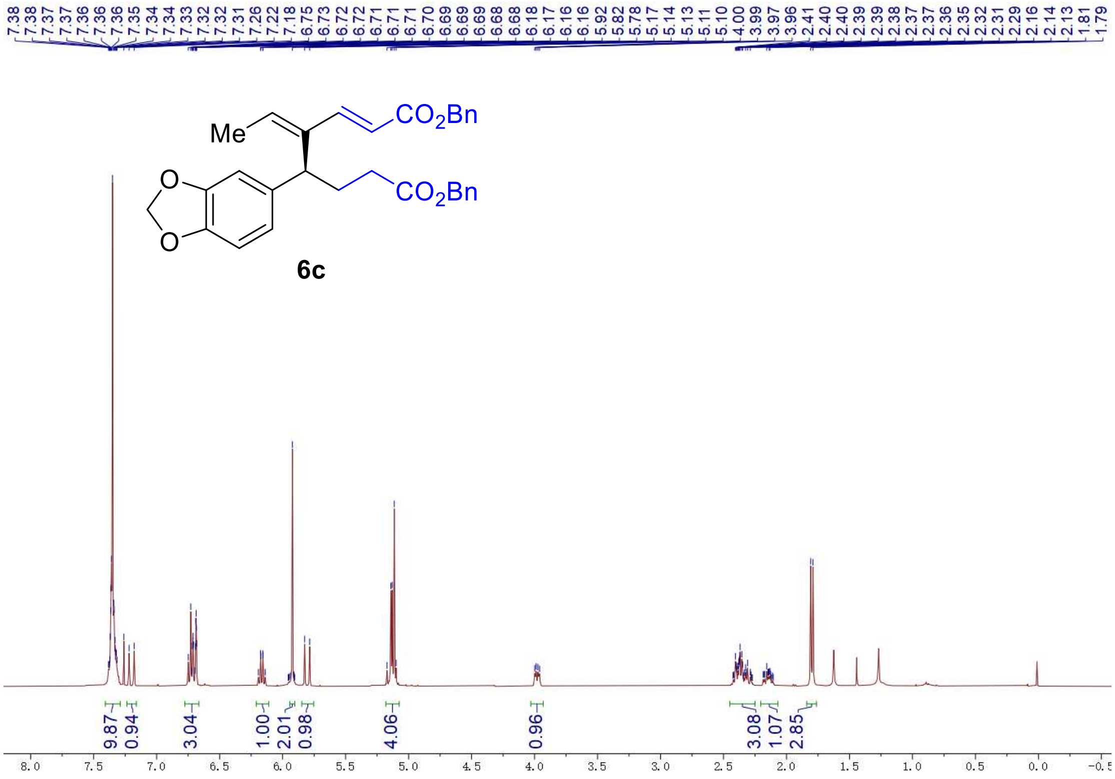

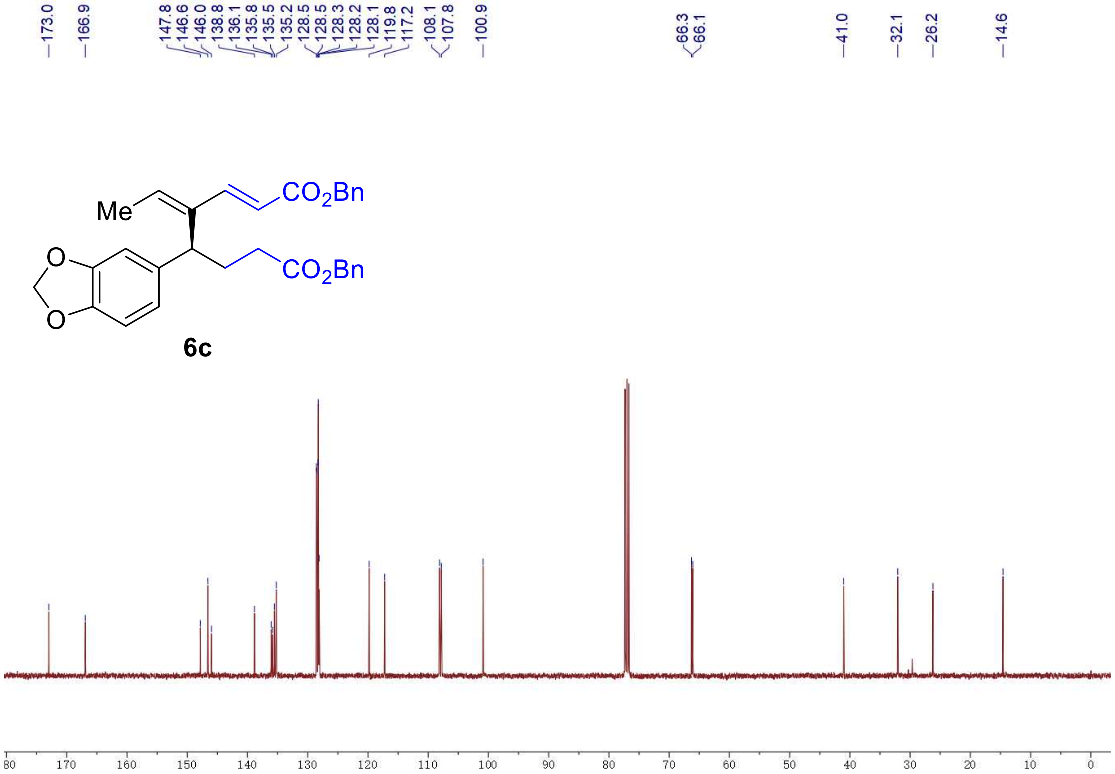

Supplementary Figure 44. ^{1}H NMR and ^{13}C NMR spectrum of compound of **6d**

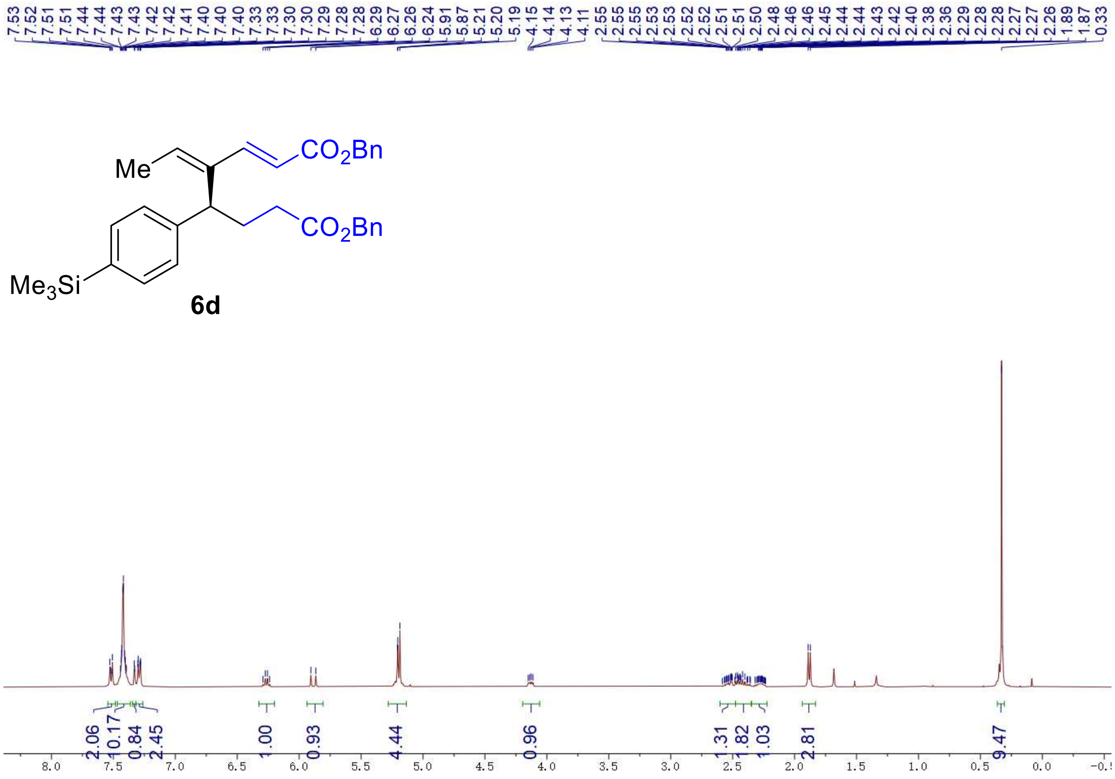

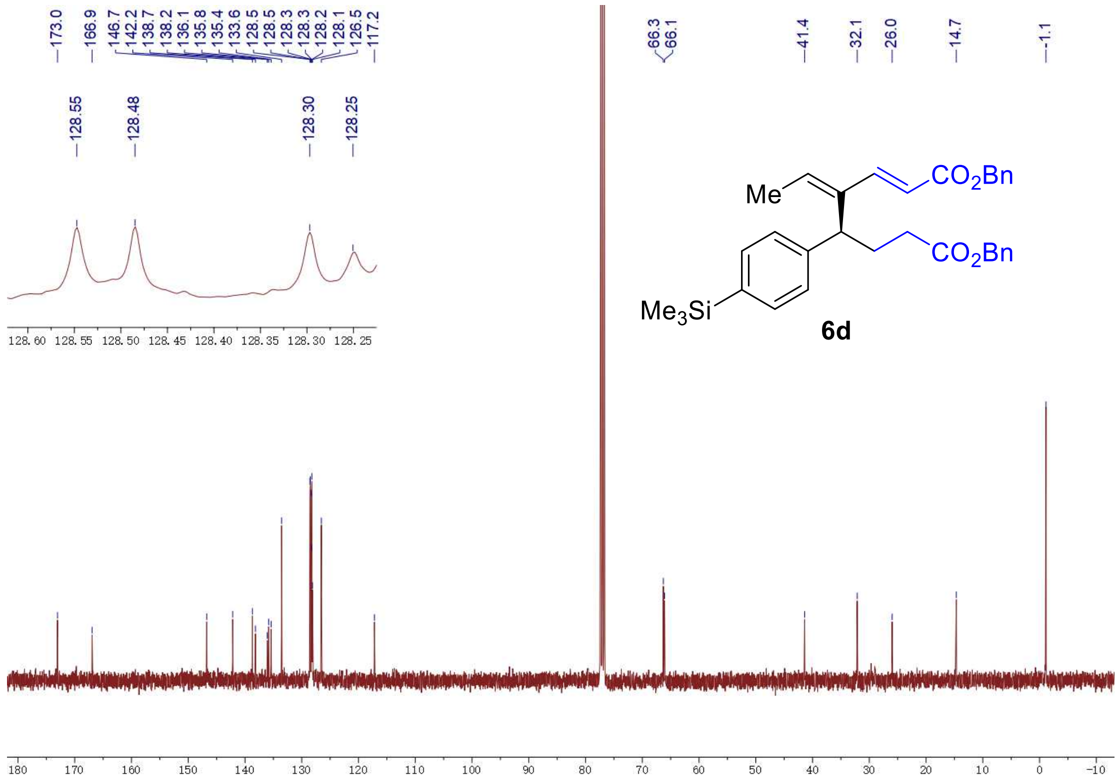

Supplementary Figure 45. ^{1}H NMR and ^{13}C NMR spectrum of compound of **6e**

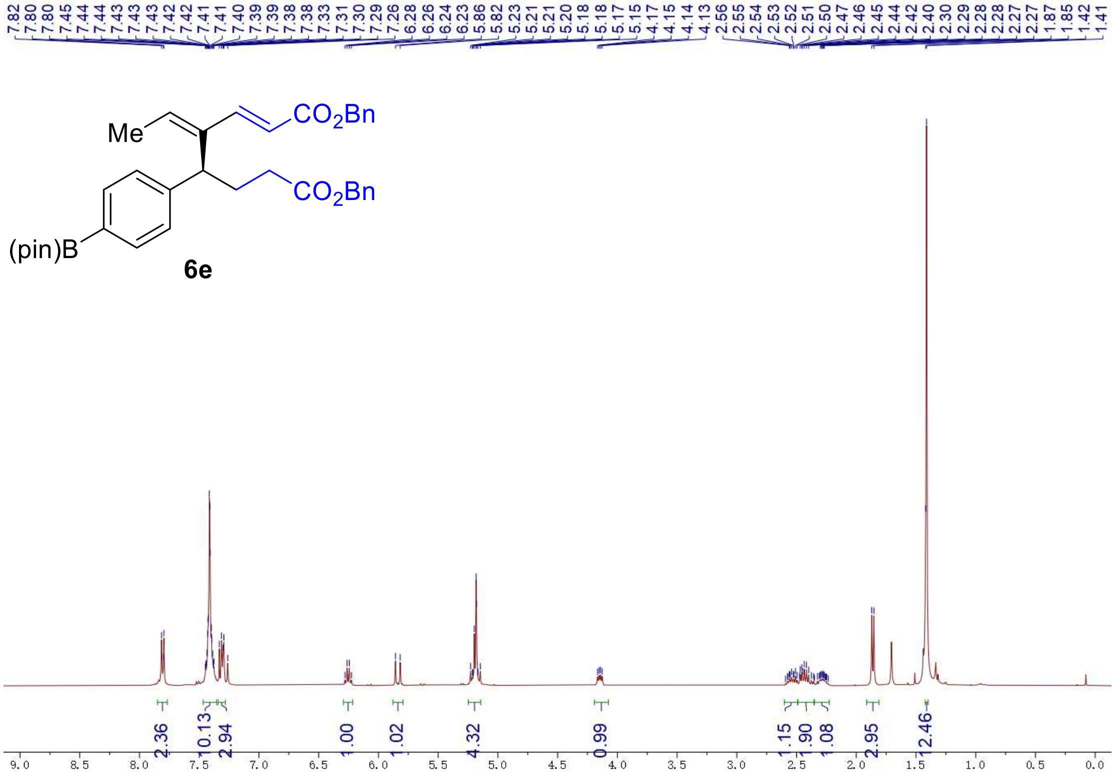

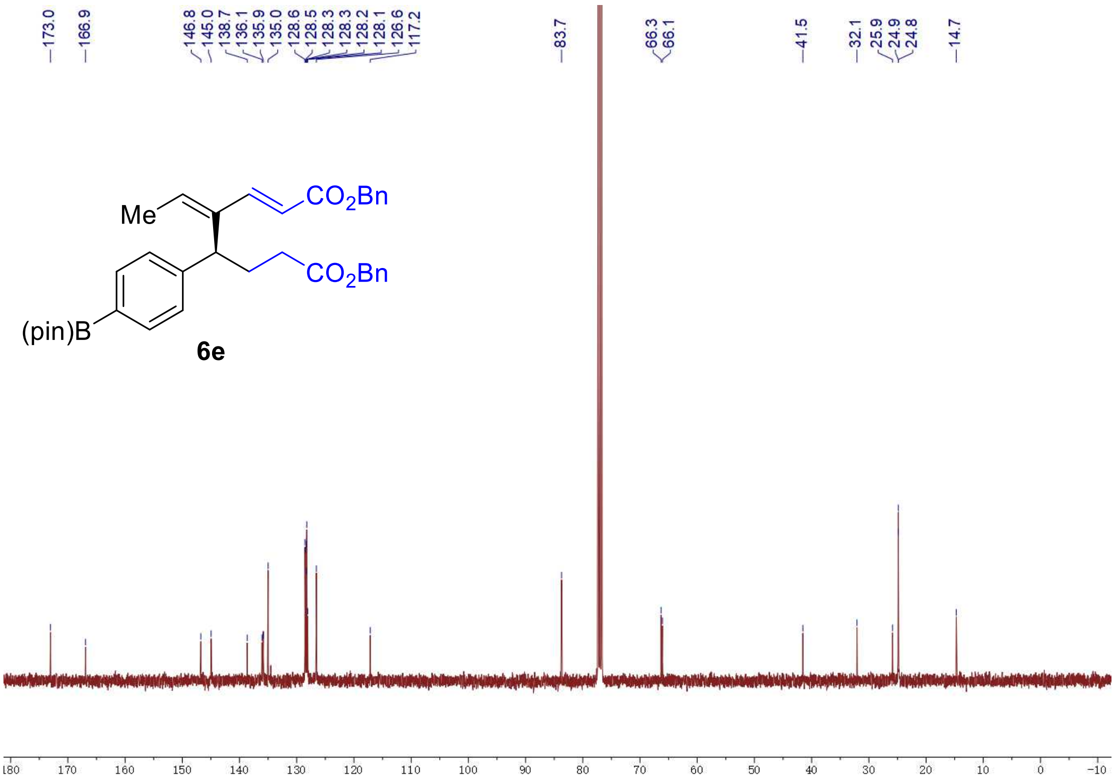

Supplementary Figure 46. ^{1}H NMR and ^{13}C NMR spectrum of compound of **6f**

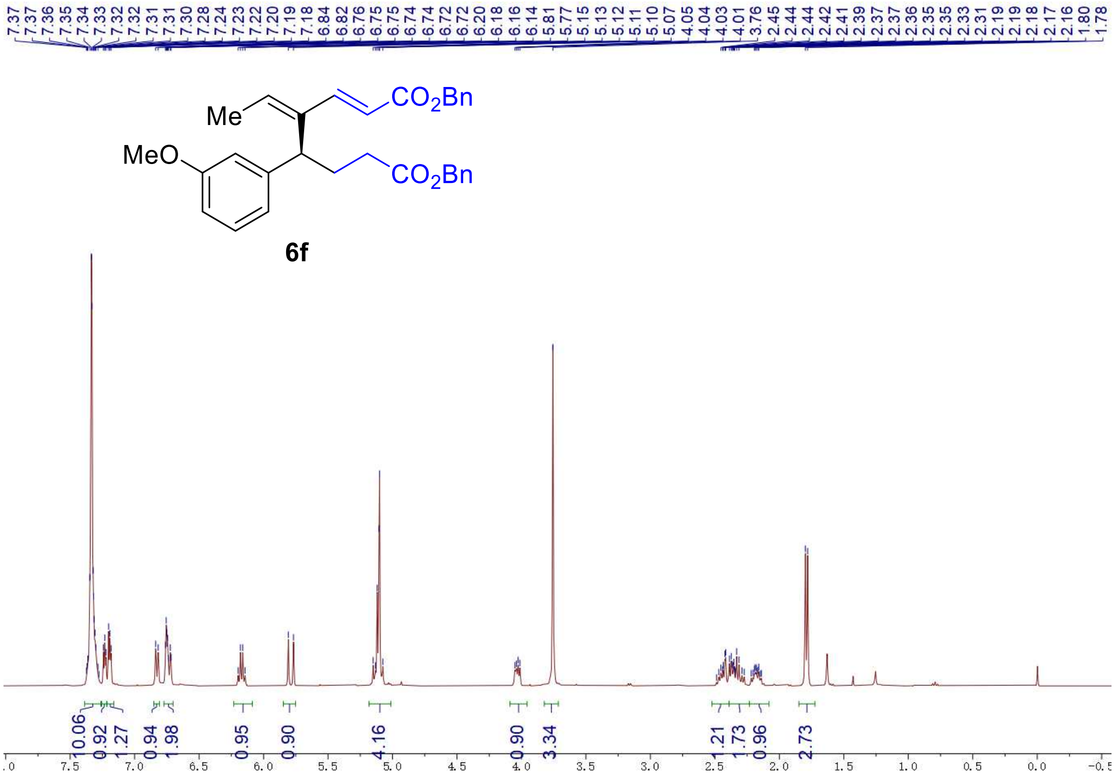

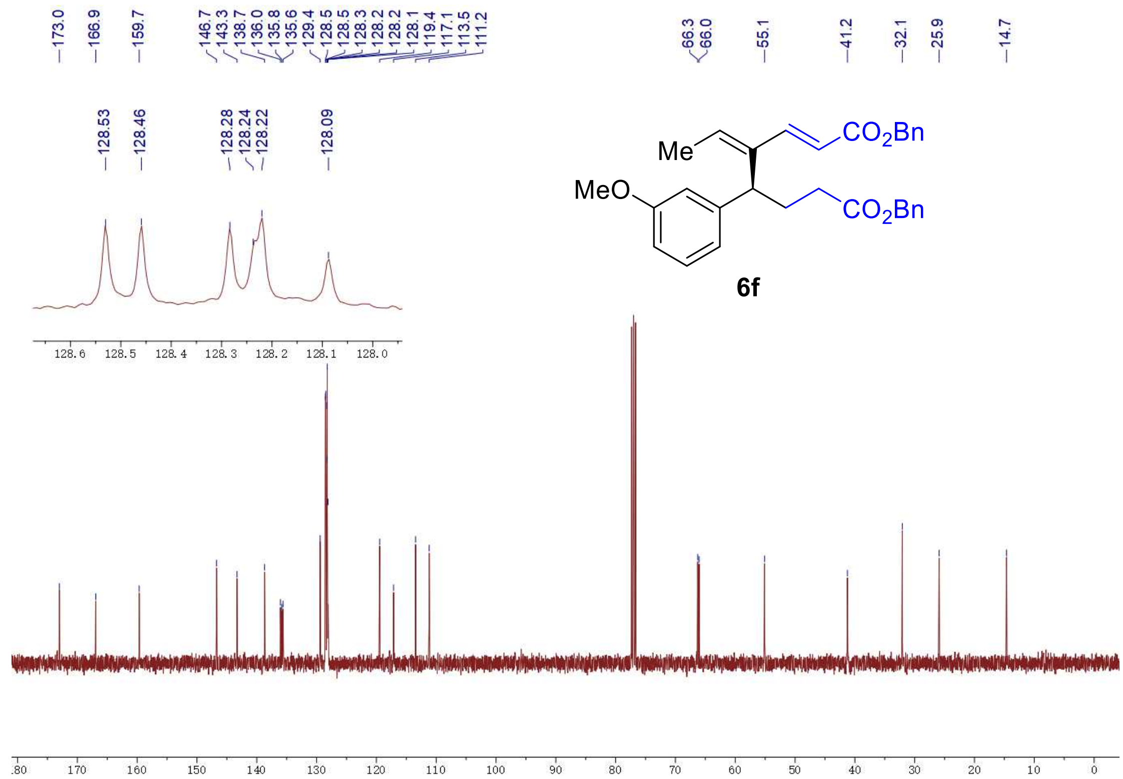

Supplementary Figure 47. ^{1}H NMR and ^{13}C NMR spectrum of compound of **6g**

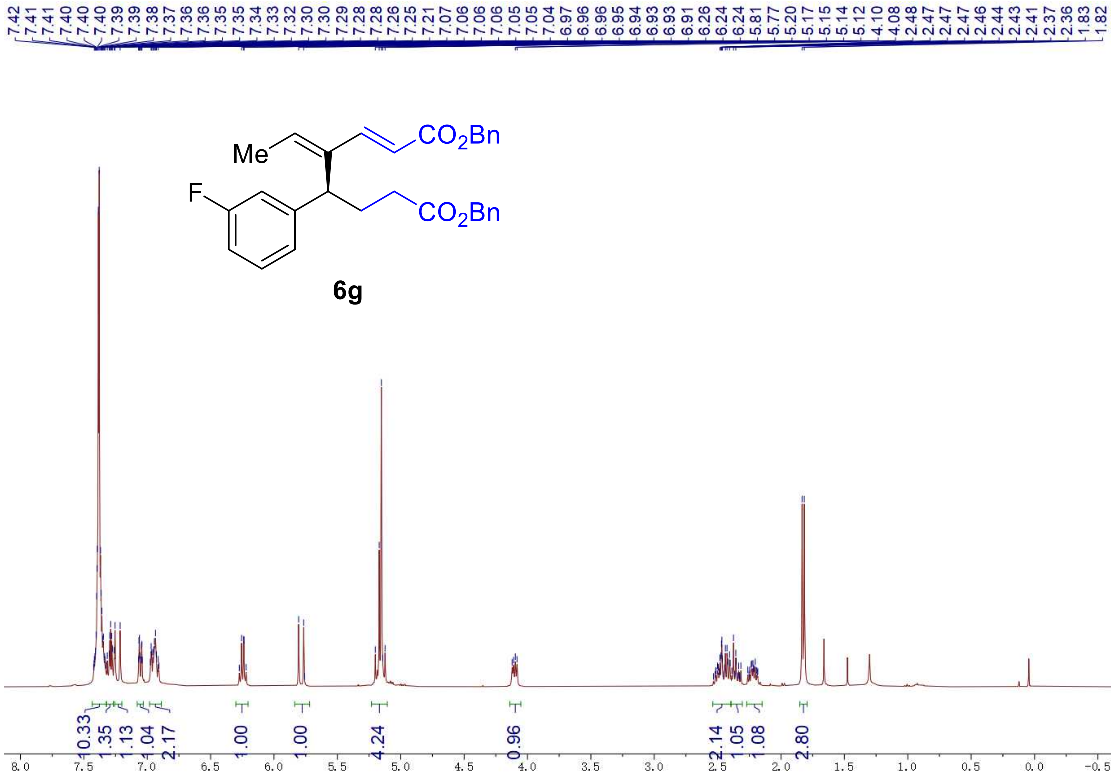

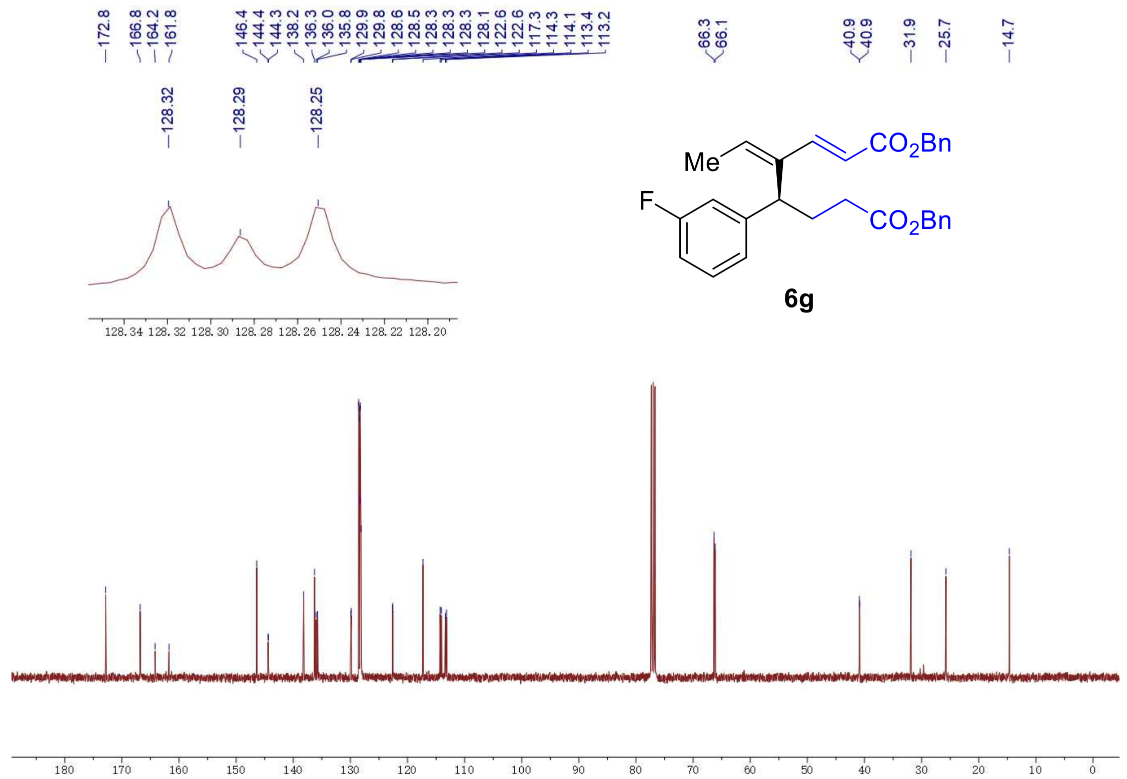
172.8
166.8
164.2
161.8
146.4
144.4
144.3
138.2
136.3
136.0
135.8
129.9
129.8
128.6
128.5
128.3
128.3
128.3
128.1
122.6
122.6
117.3
114.3
114.1
113.4
113.2
66.3
66.1
40.9
40.9
31.9
25.7
14.7
128.32
128.29
128.25
Me
F
CO_2Bn
CO_2Bn
6g

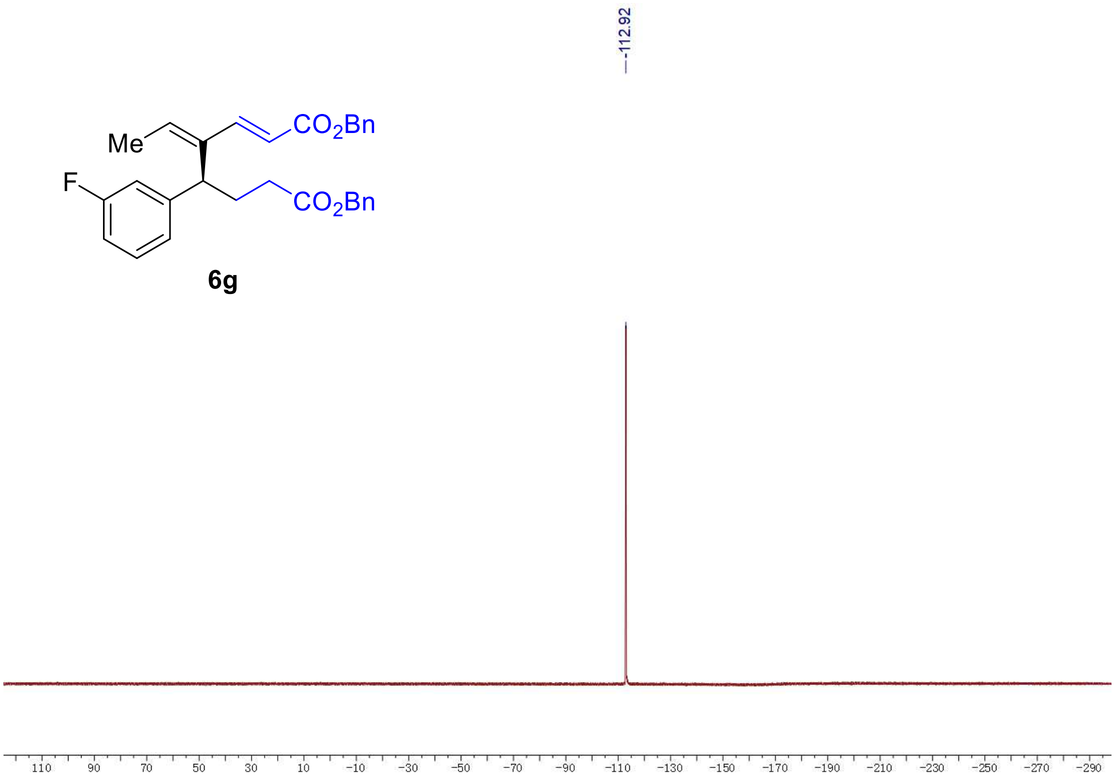
-112.92
Me
F
CO_2Bn
CO_2Bn
6g

Supplementary Figure 48. ^{1}H NMR and ^{13}C NMR spectrum of compound of **6h**

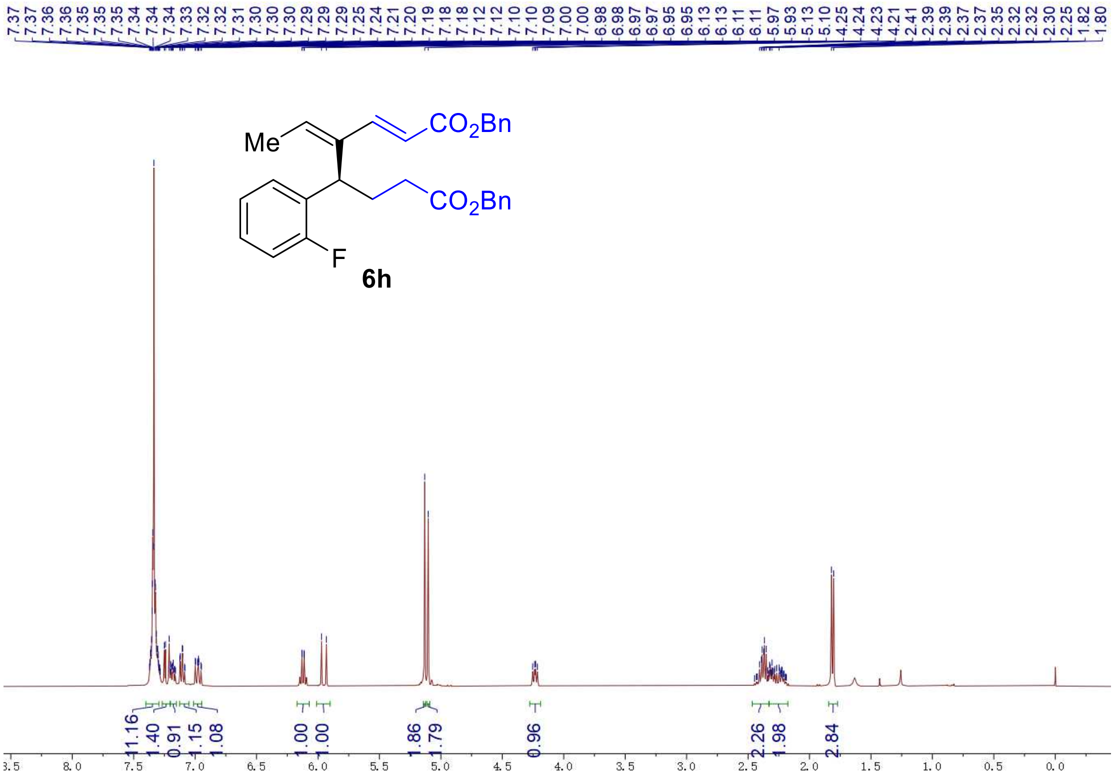

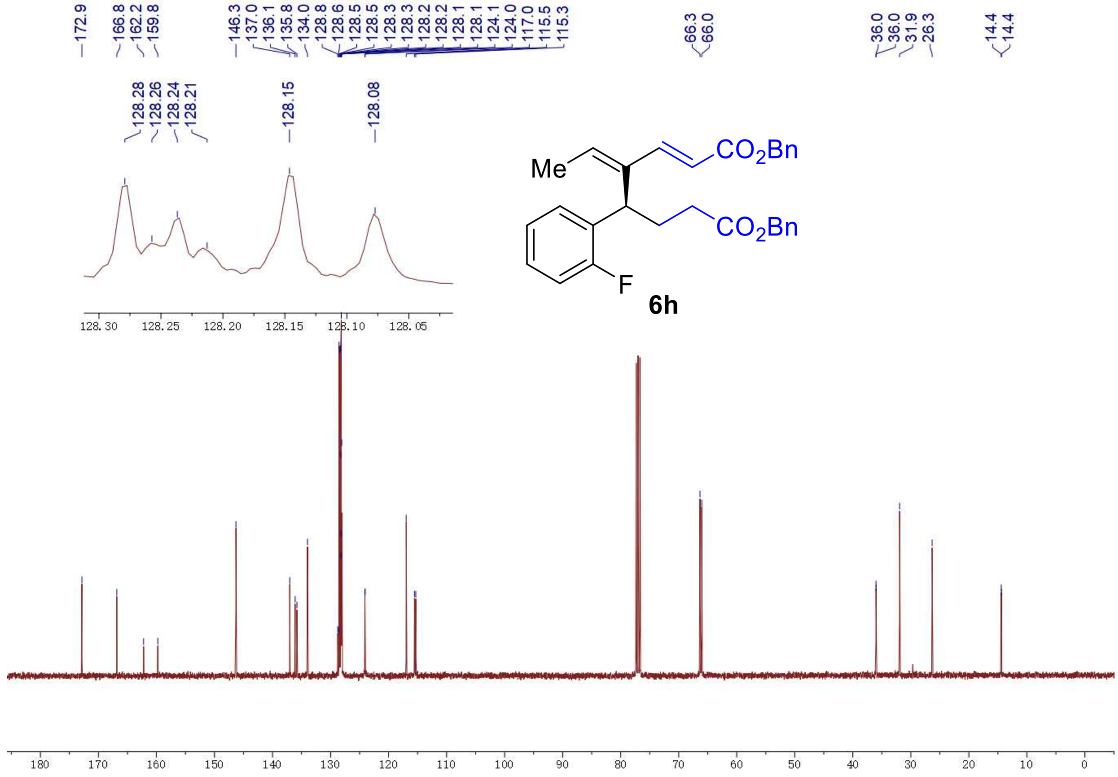

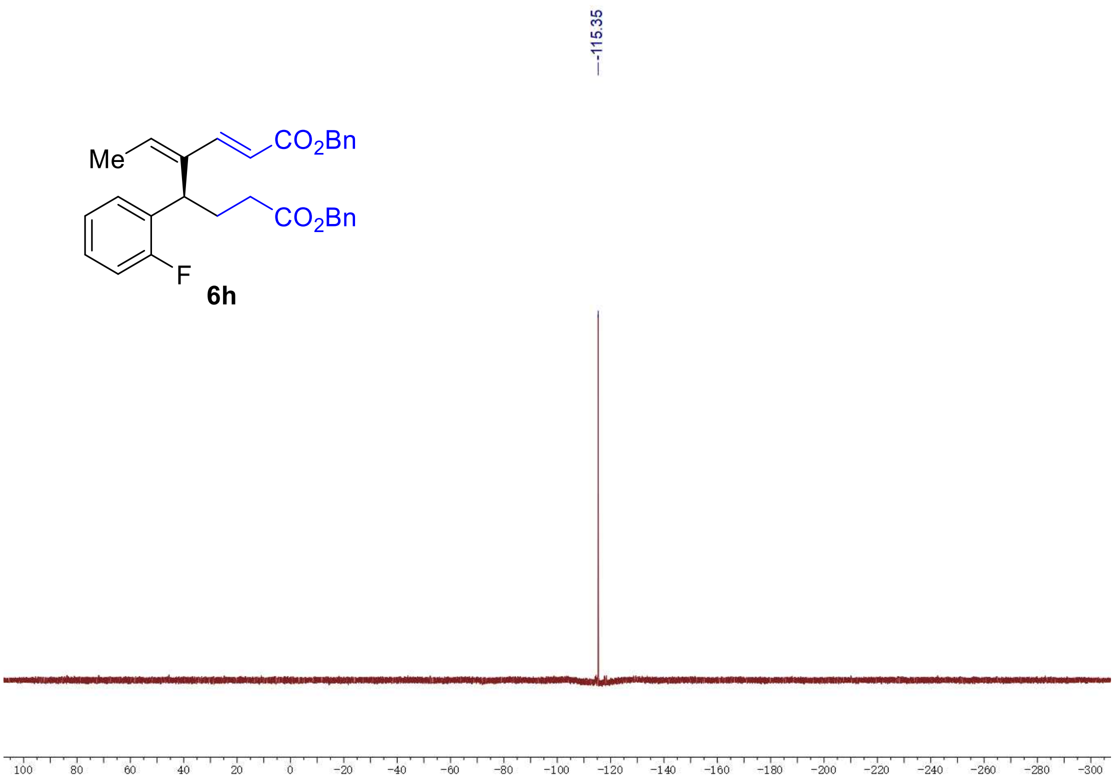

Supplementary Figure 49. ^{1}H NMR and ^{13}C NMR spectrum of compound of **6i**

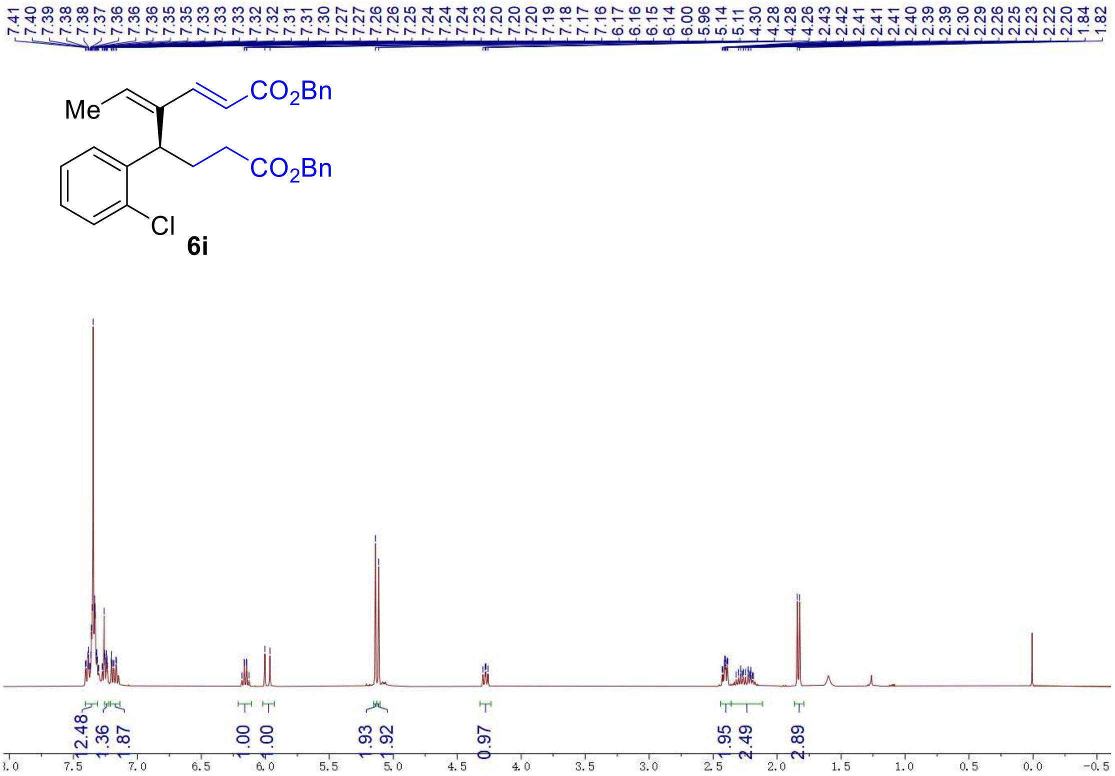

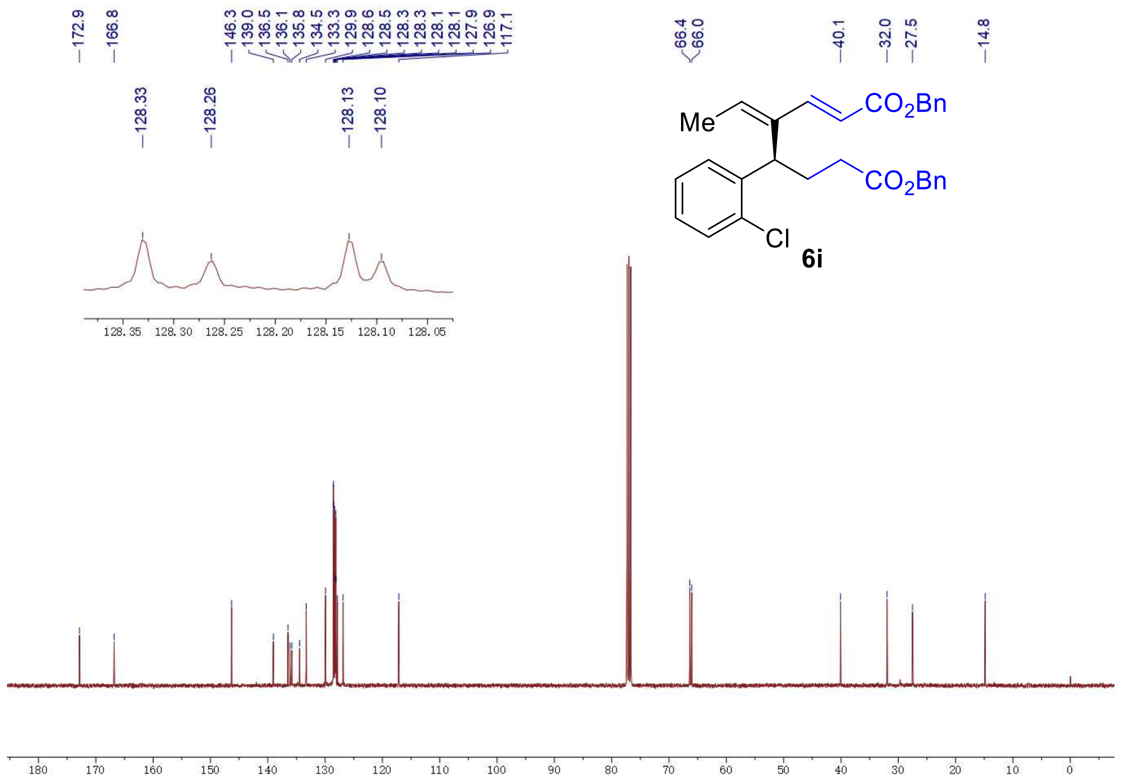

Supplementary Figure 50. ^{1}H NMR and ^{13}C NMR spectrum of compound of **6j**

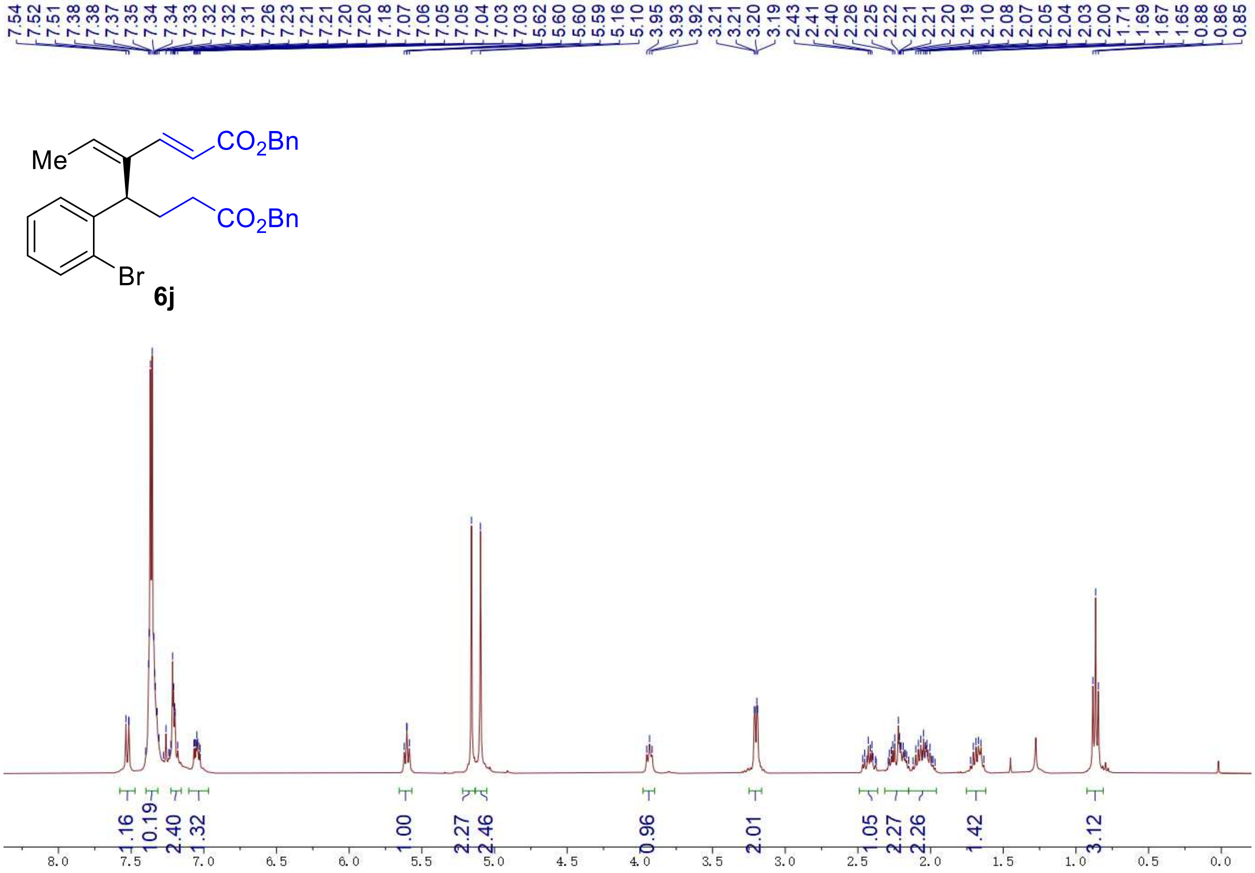

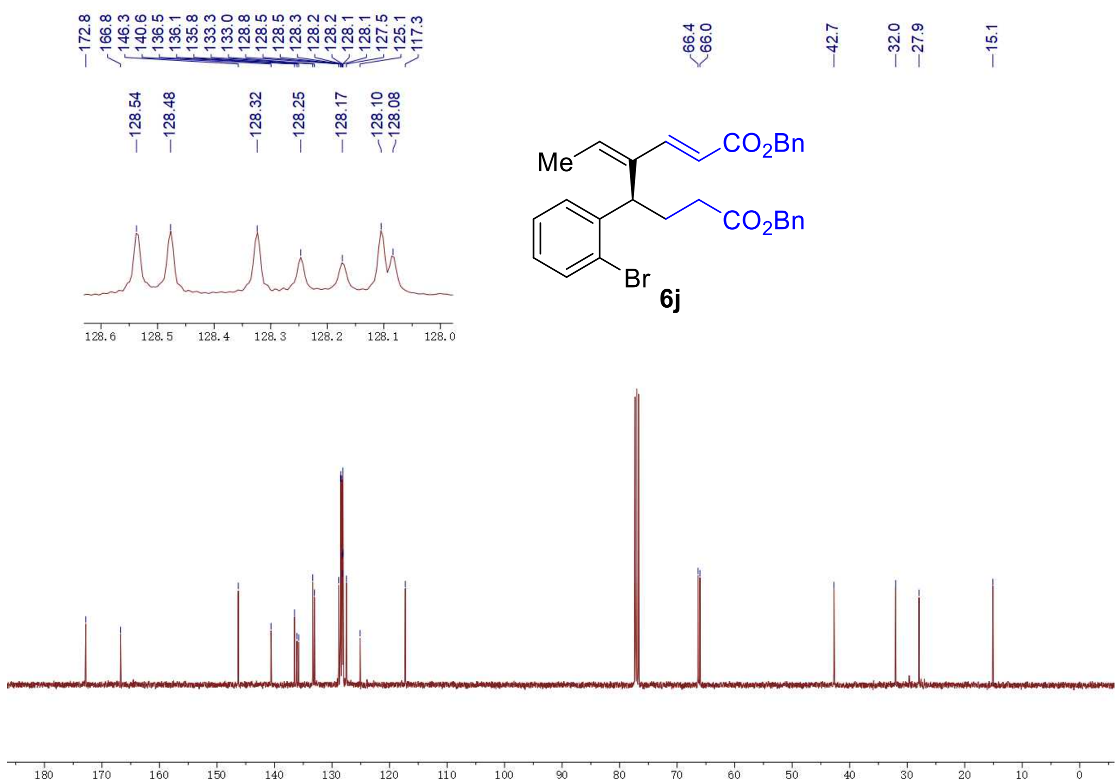

Supplementary Figure 51. ^{1}H NMR and ^{13}C NMR spectrum of compound of **6k**

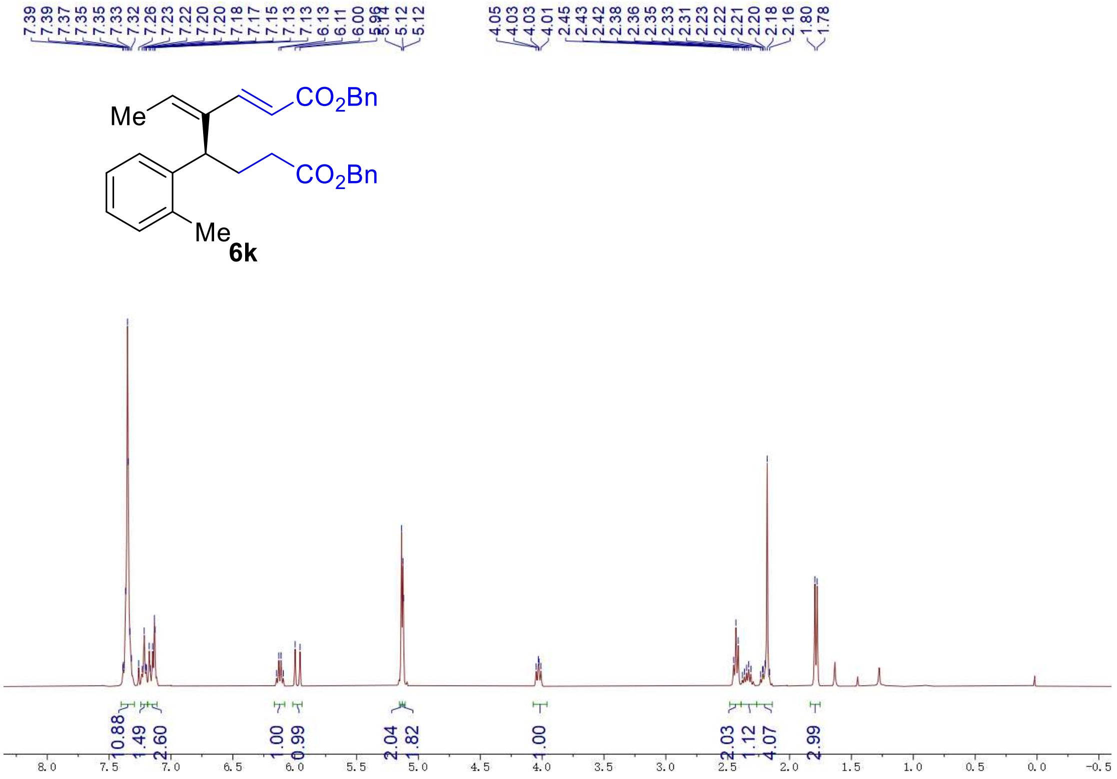

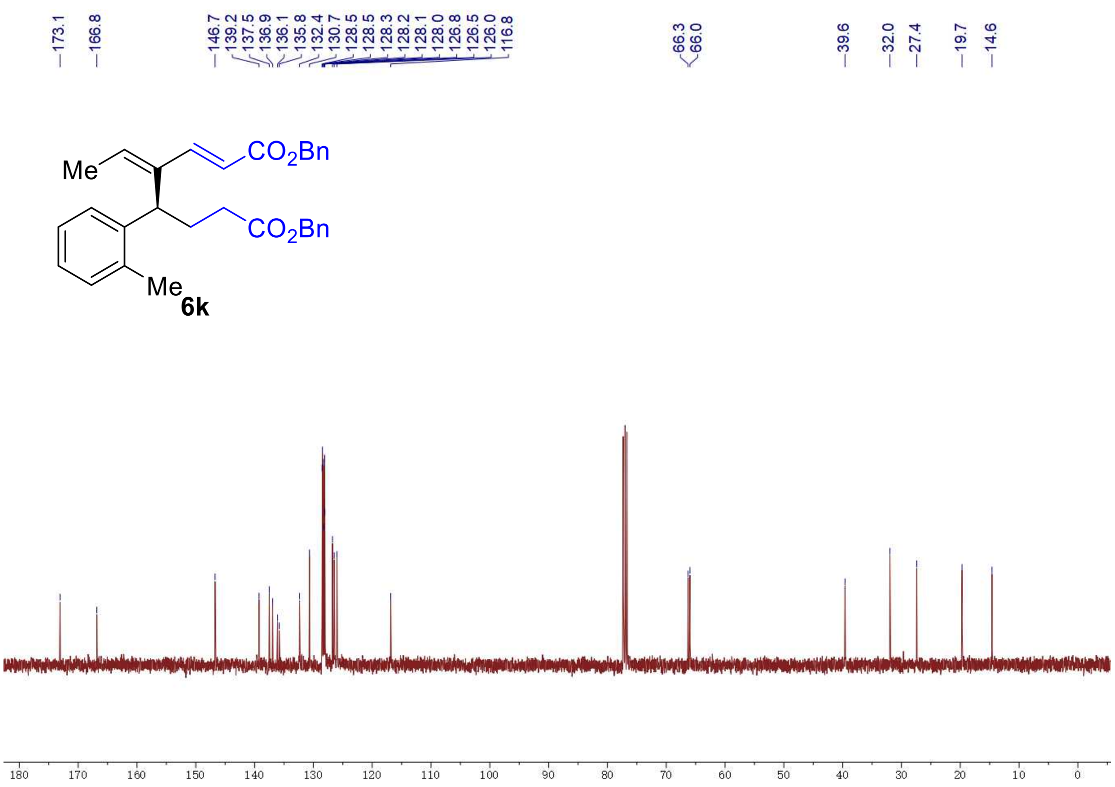

Supplementary Figure 52. ¹H NMR and ¹³C NMR spectrum of compound of **6l**

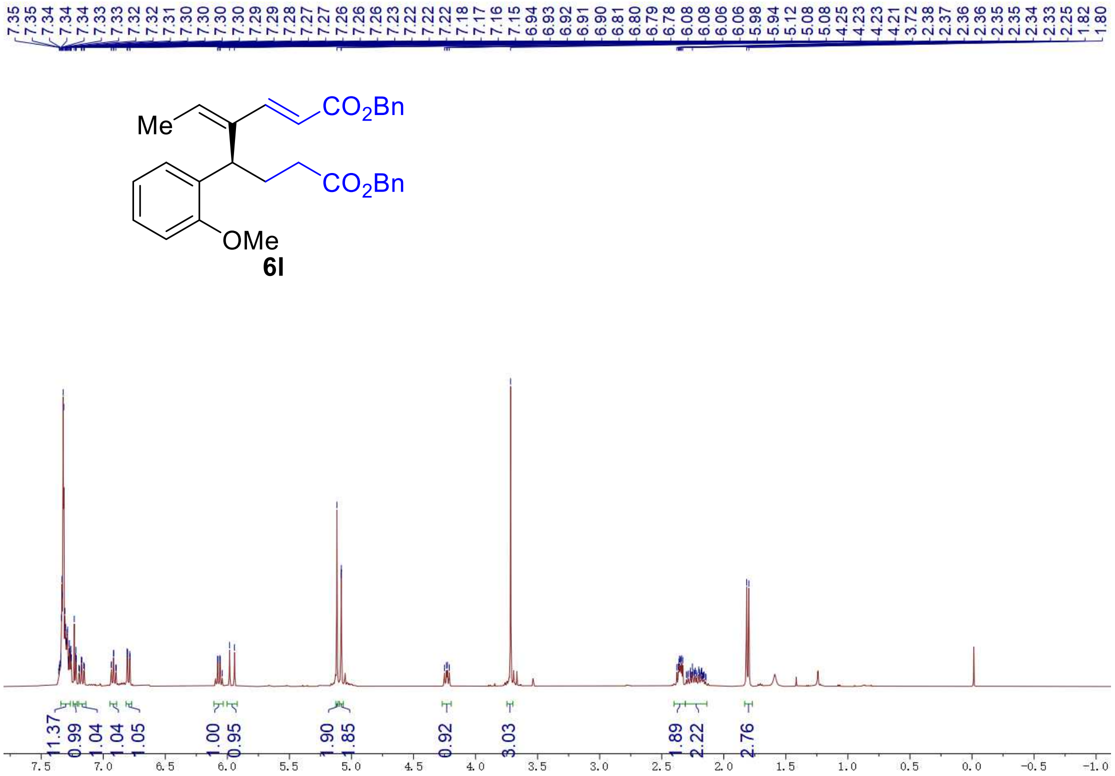

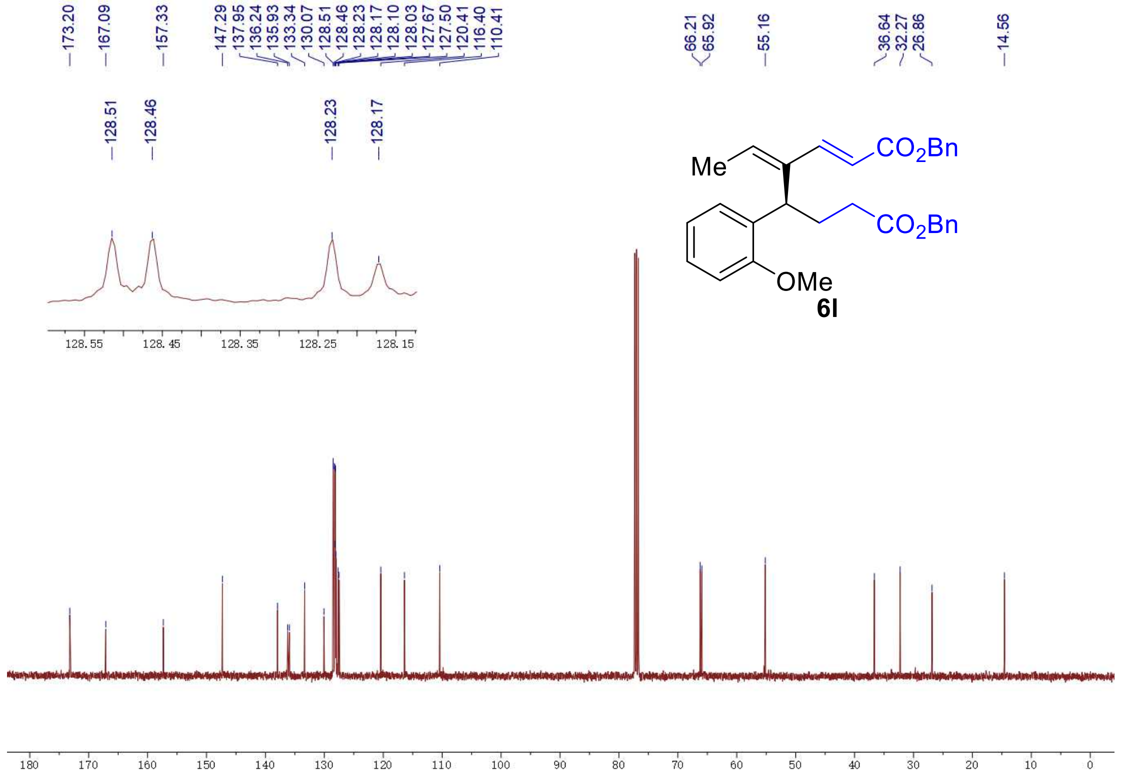

Supplementary Figure 53. ^{1}H NMR and ^{13}C NMR spectrum of compound of **6m**

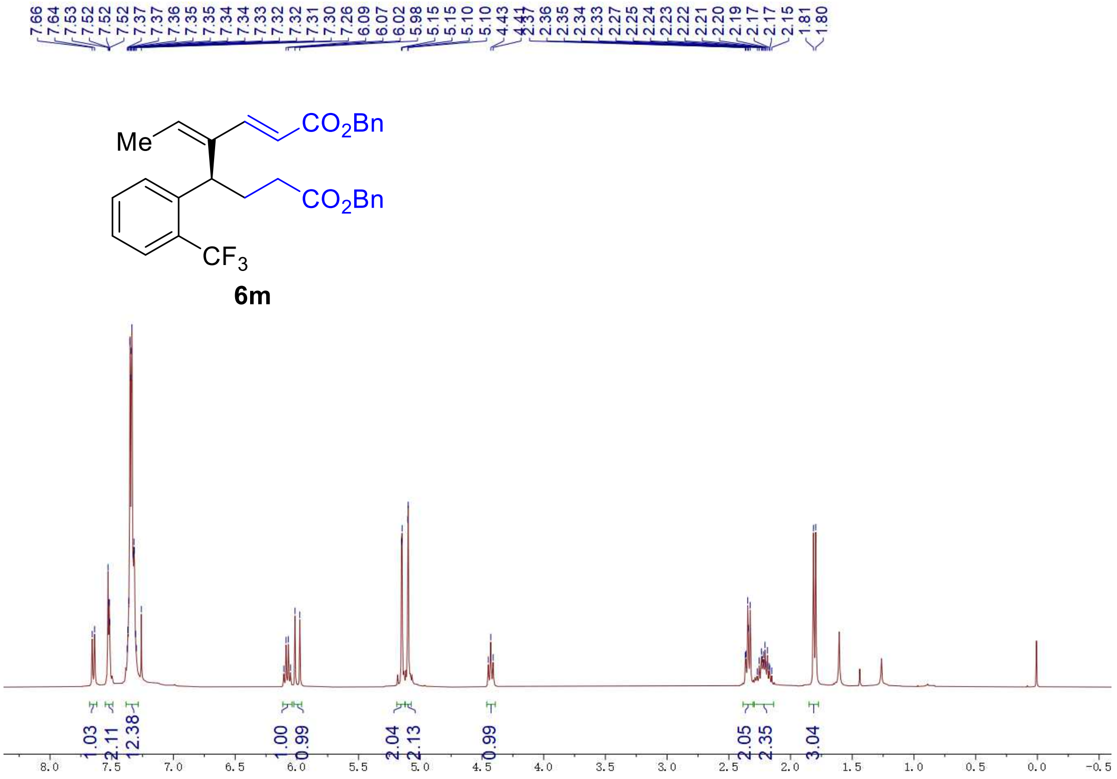

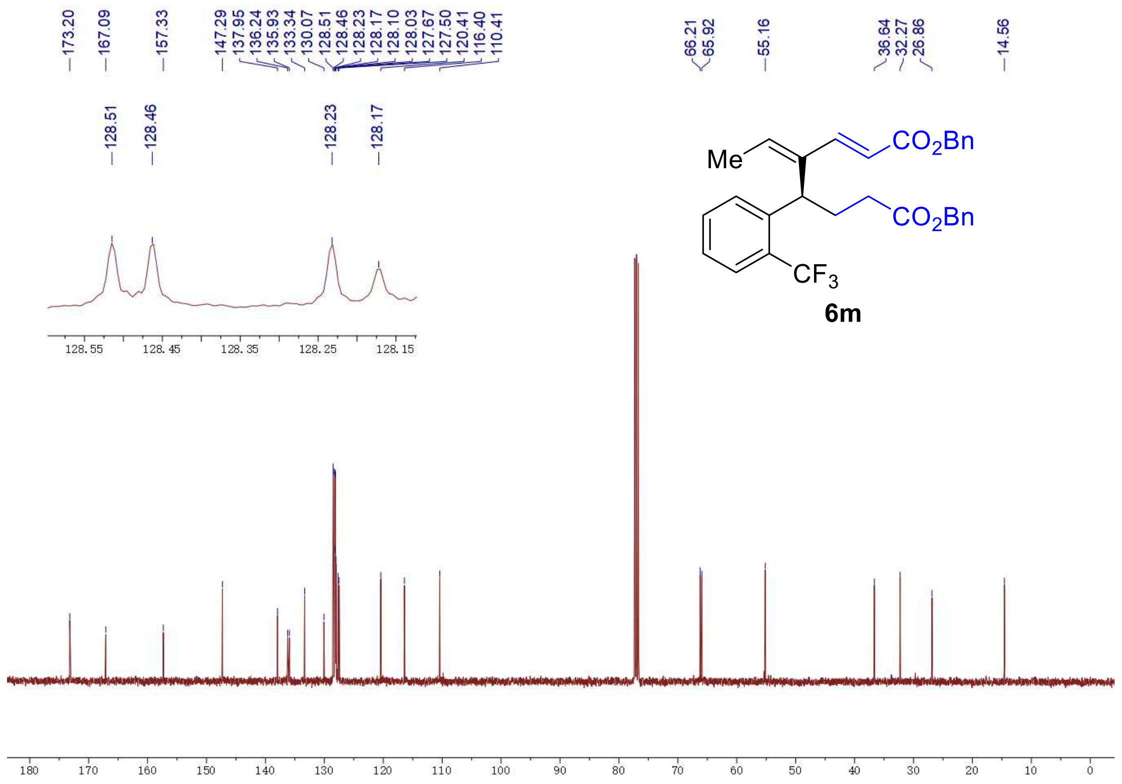

173.20
167.09
157.33
147.29
137.95
136.24
135.93
133.34
130.07
128.51
128.46
128.23
128.17
128.10
128.03
127.67
127.50
120.41
116.40
110.41
66.21
65.92
55.16
36.64
32.27
26.86
14.56
Me
CO_2Bn
CO_2Bn
CF_3
6m

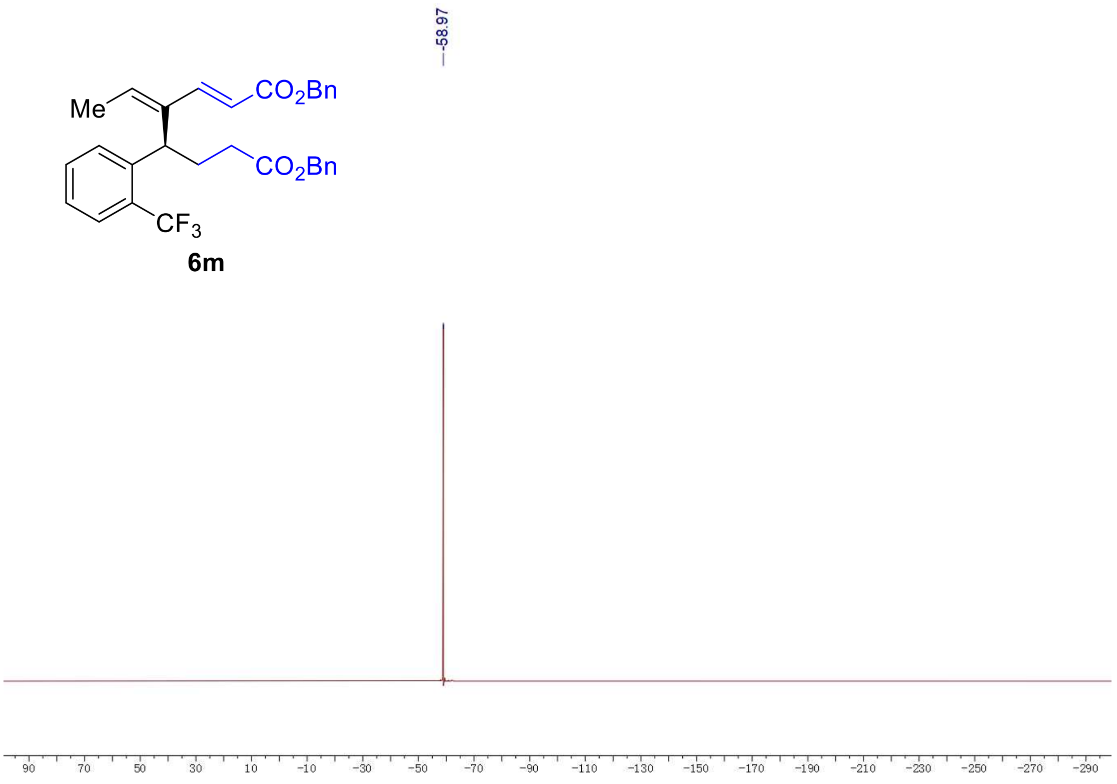

-58.97
Me
CO_2Bn
CO_2Bn
CF_3
6m

Supplementary Figure 54. ^{1}**H NMR and** ^{13}**C NMR** spectrum of compound of **6n**

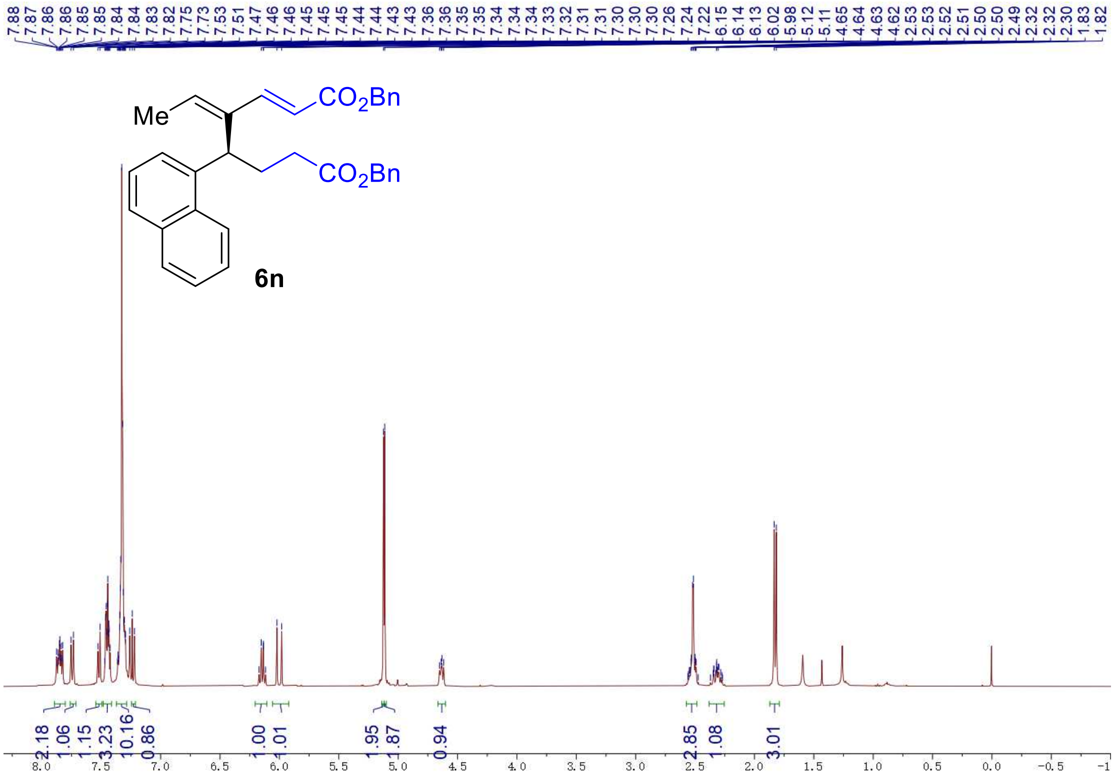

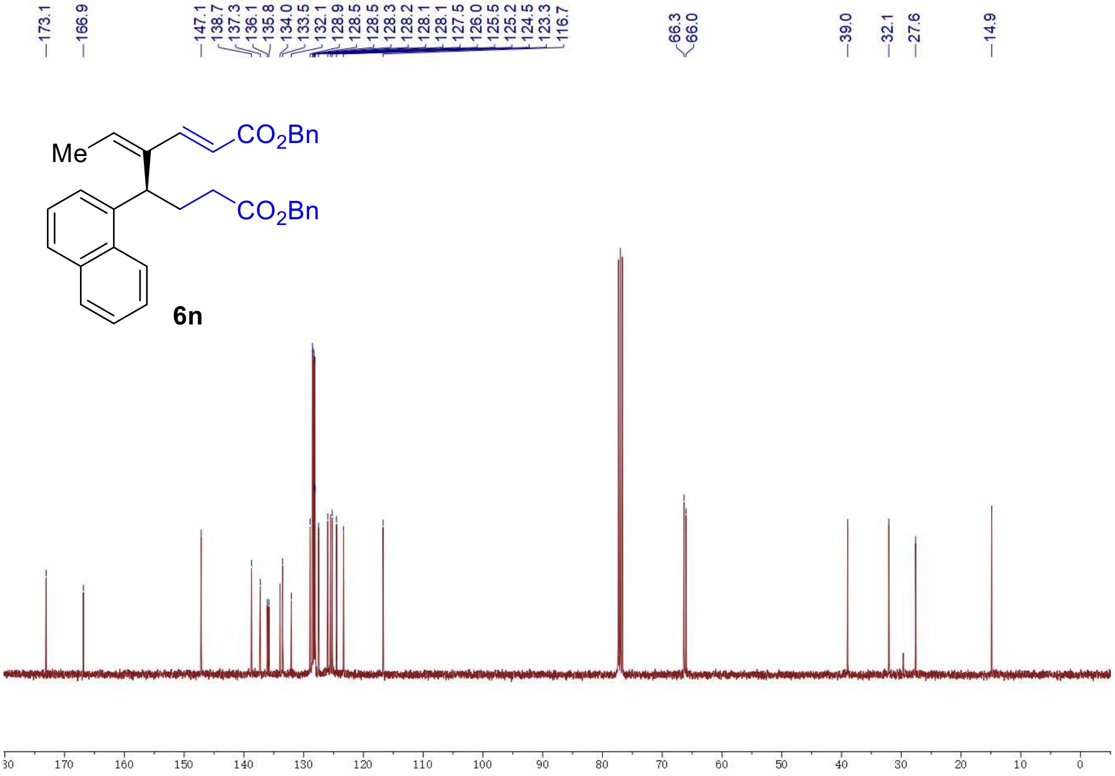

Supplementary Figure 55. ^{1}H NMR and ^{13}C NMR spectrum of compound of **7a**

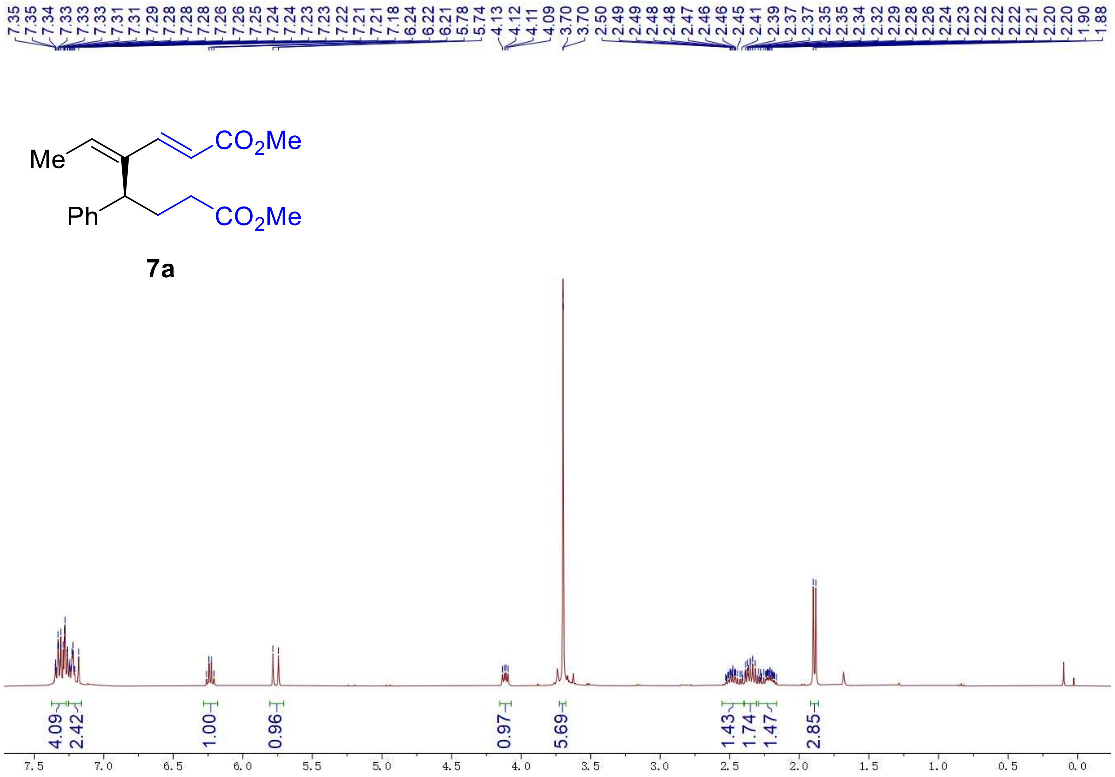

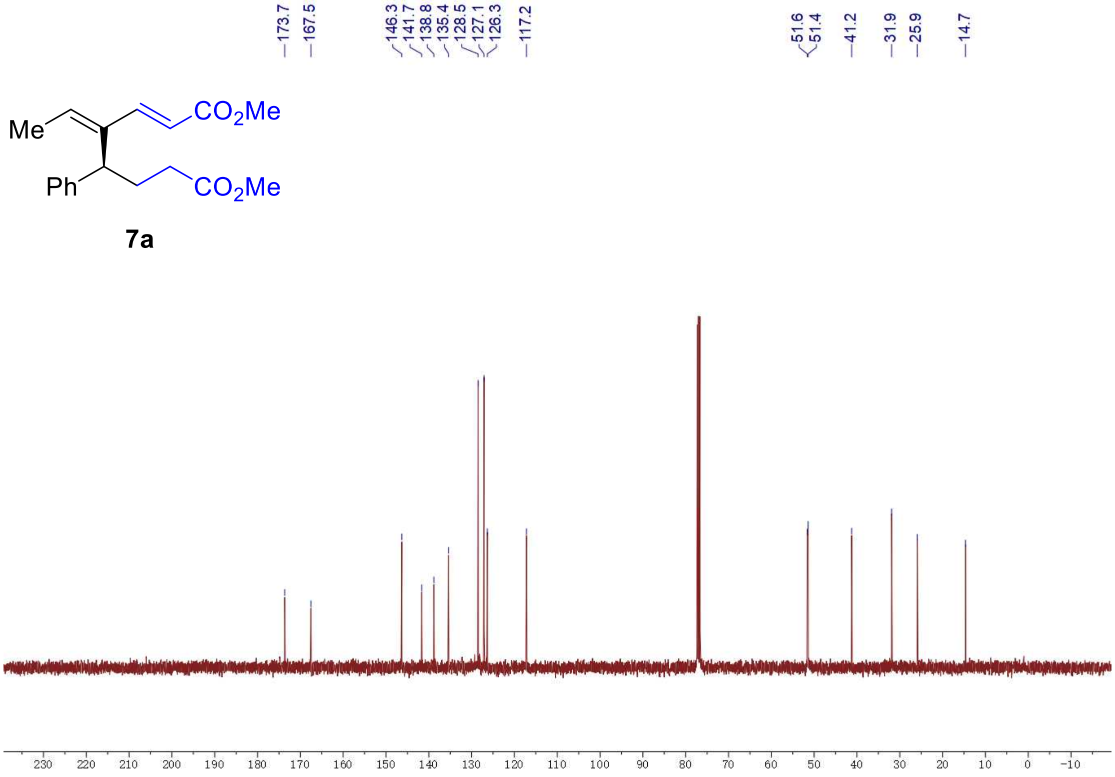

Supplementary Figure 56. ^{1}H NMR and ^{13}C NMR spectrum of compound of **7b**

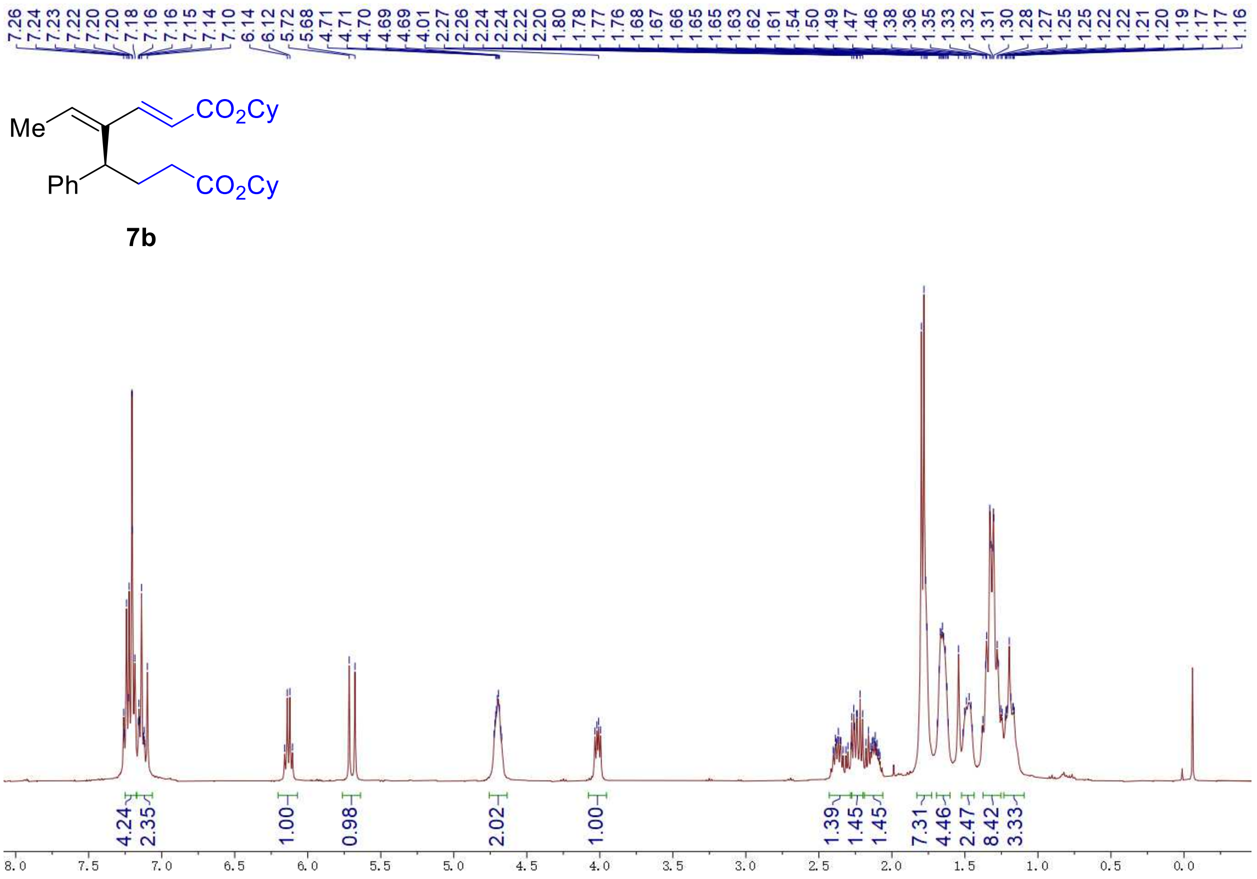

7b

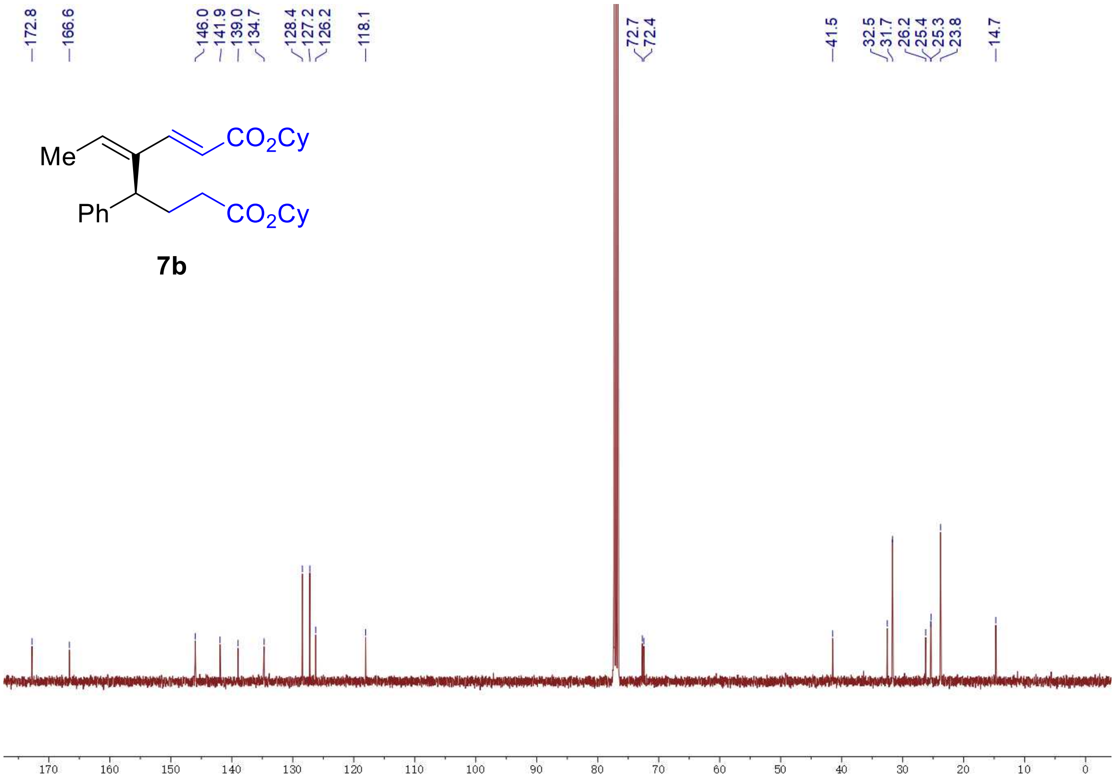

7b

Supplementary Figure 57. **^{1}H NMR and ^{13}C NMR** spectrum of compound of **8a**

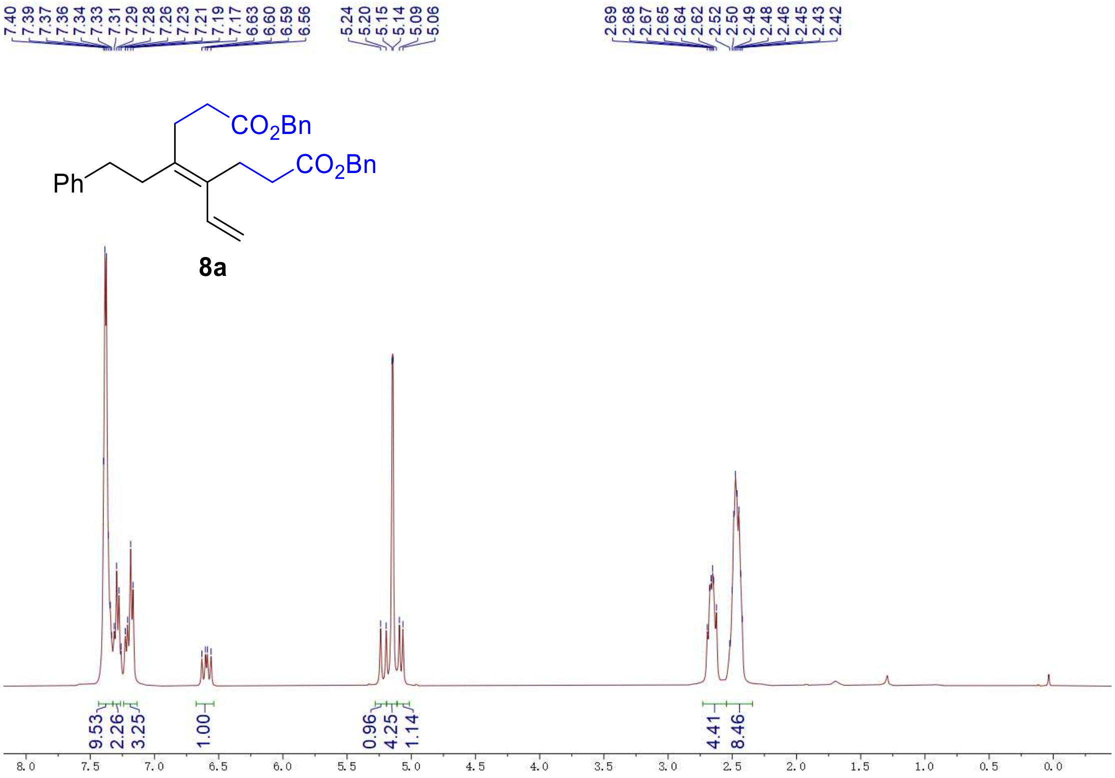

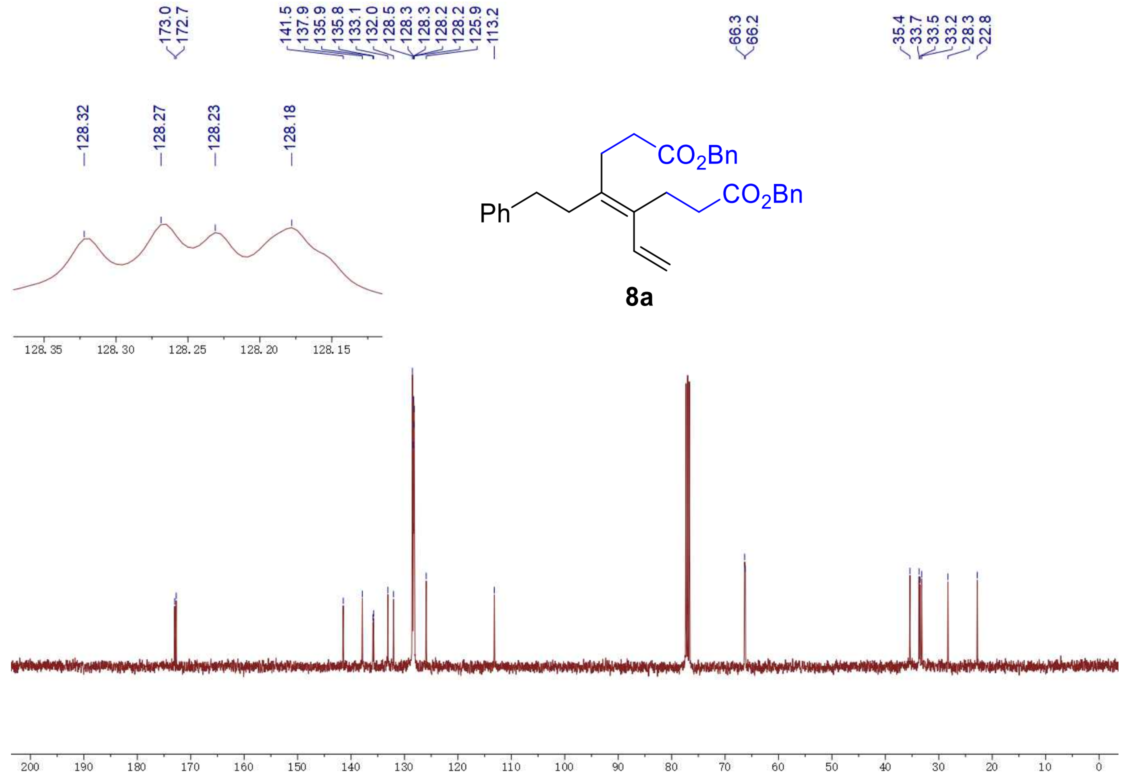

Supplementary Figure 58. ^{1}**H NMR and** ^{13}**C NMR** spectrum of compound of **8b**

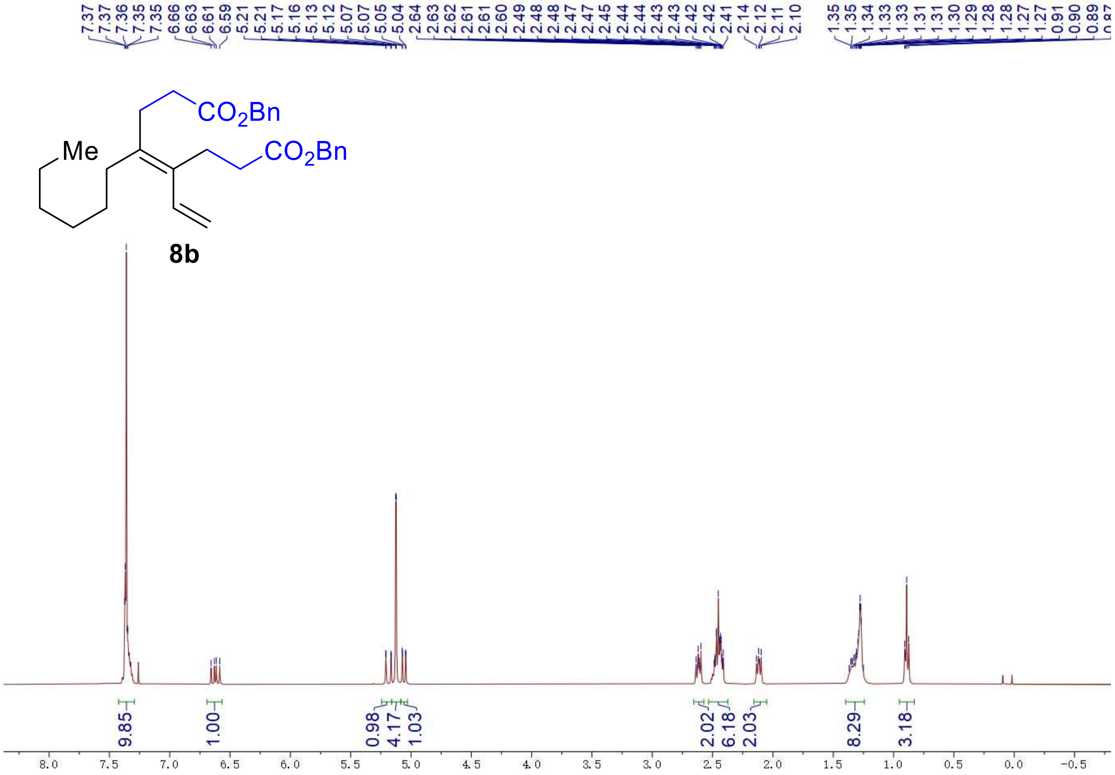

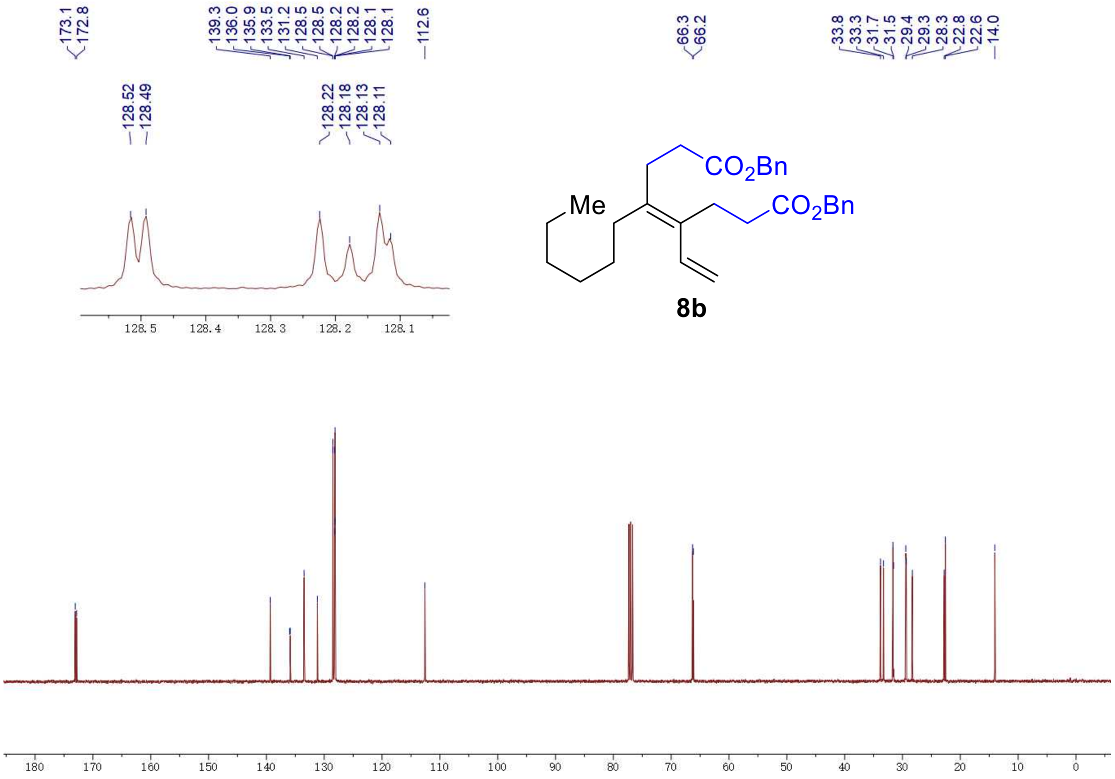

Supplementary Figure 59. ^{1}H NMR and ^{13}C NMR spectrum of compound of **8c**

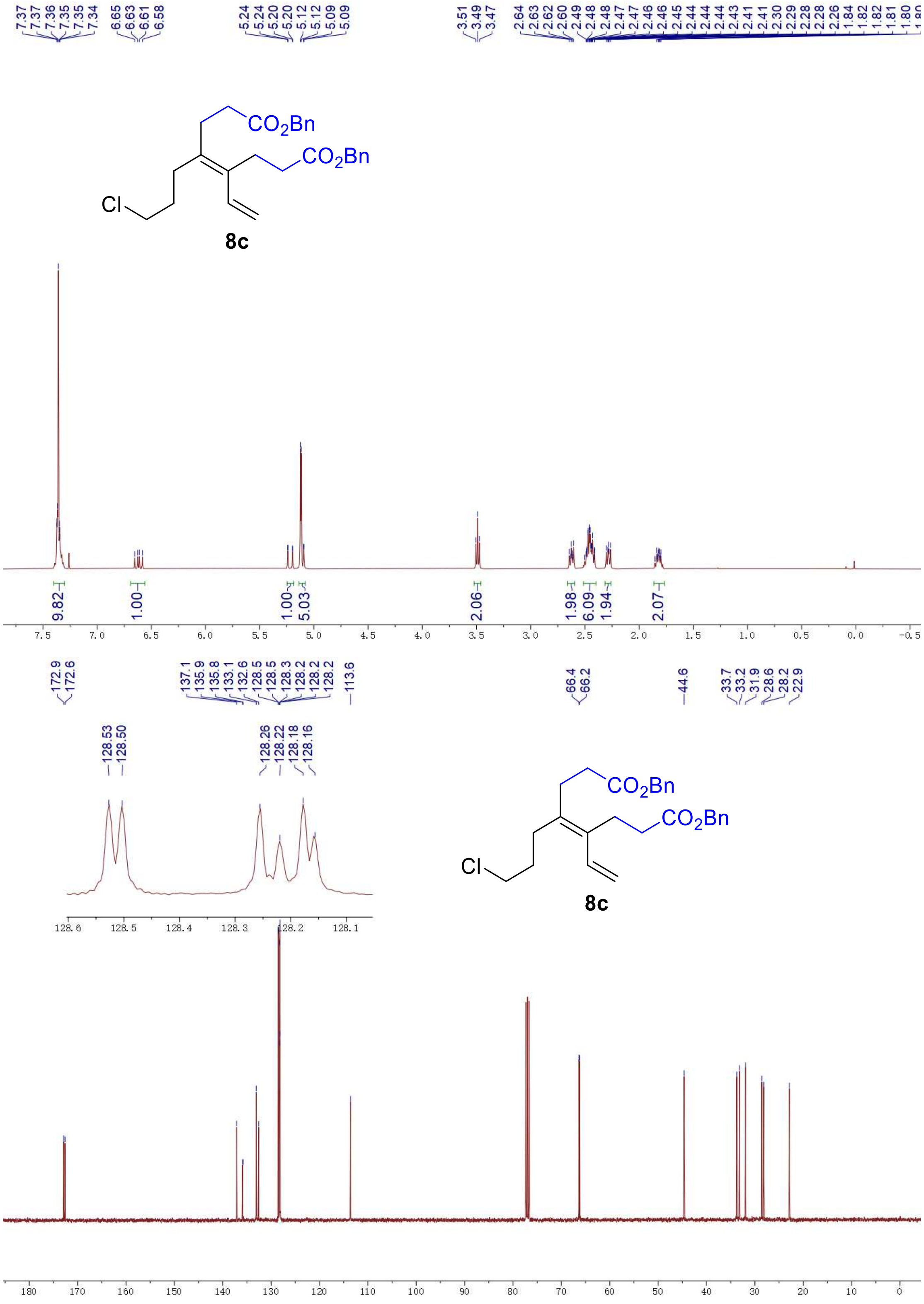

Supplementary Figure 60. ^{1}H NMR and ^{13}C NMR spectrum of compound of **8d**

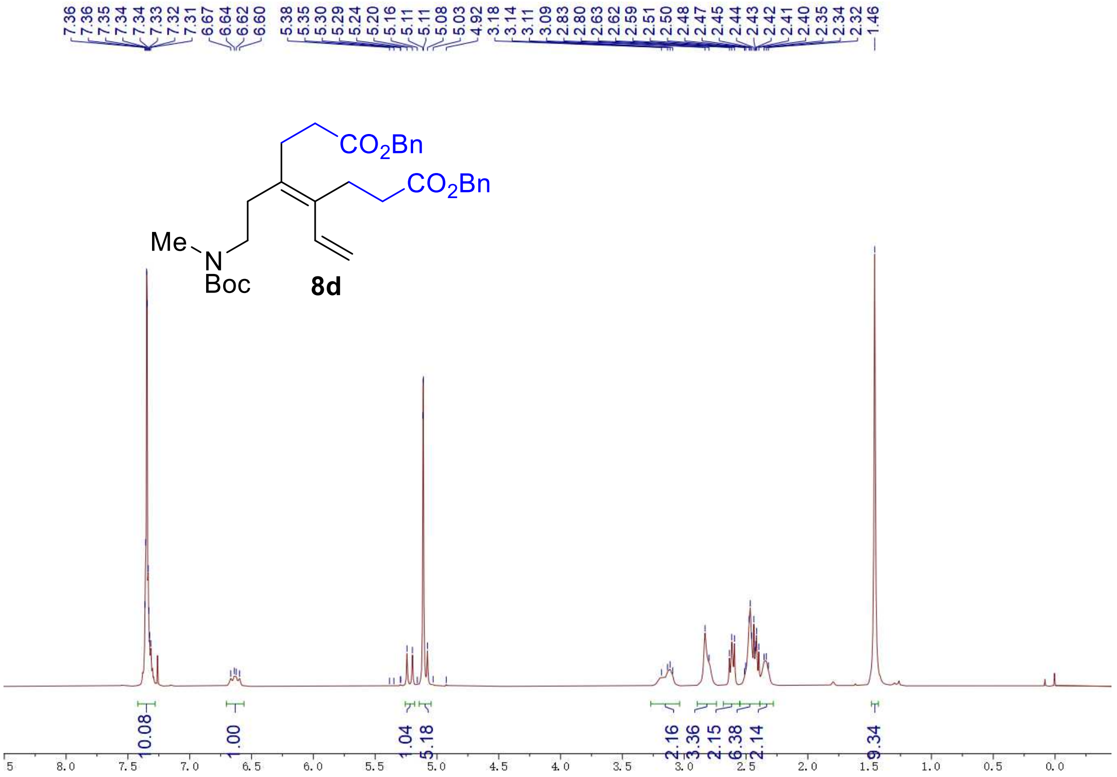

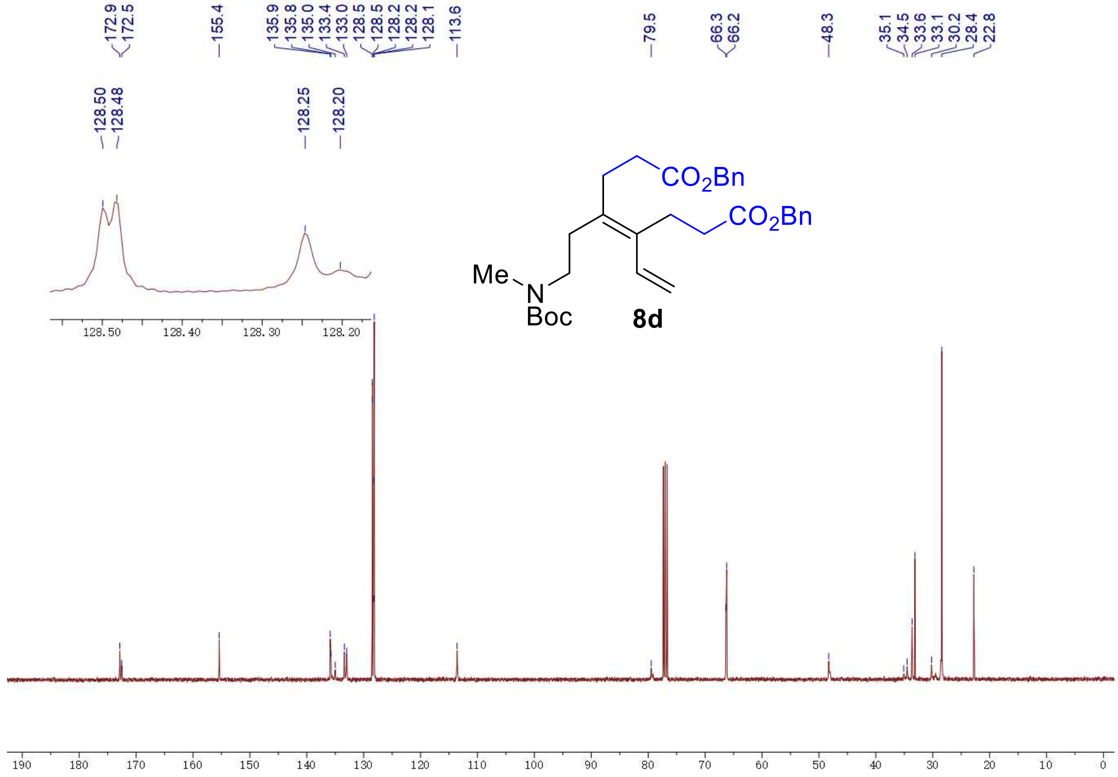

Supplementary Figure 61. **^{1}H NMR and ^{13}C NMR** spectrum of compound of **8e**

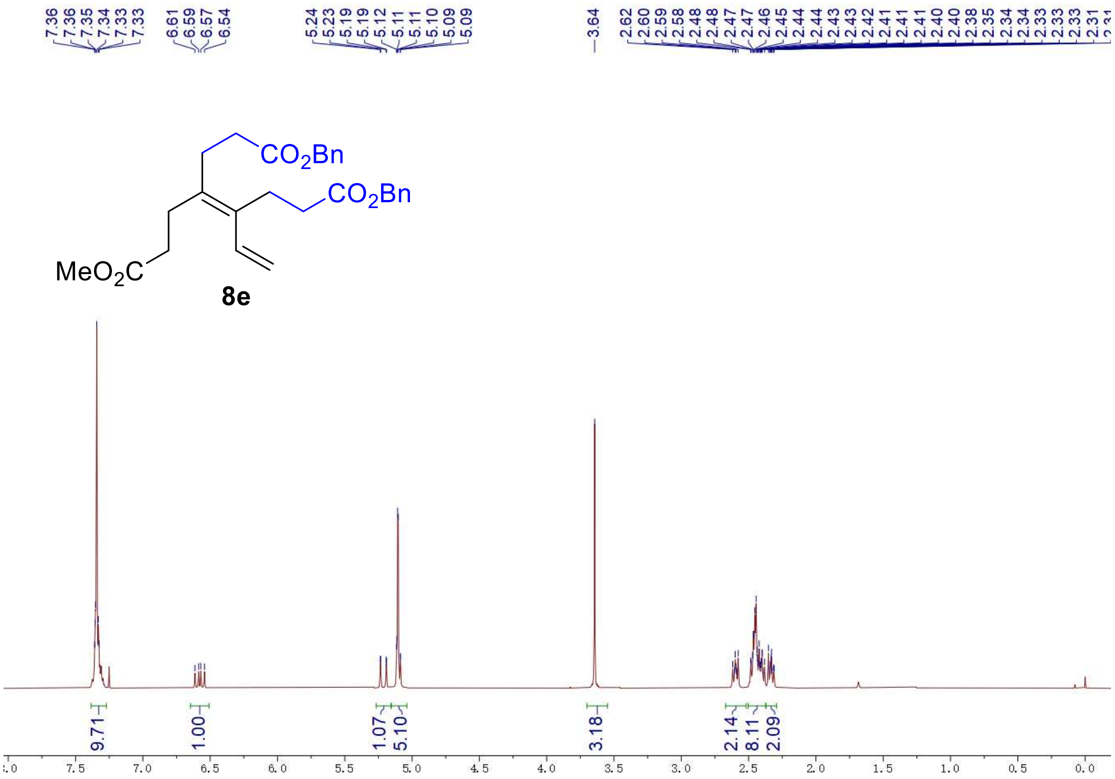

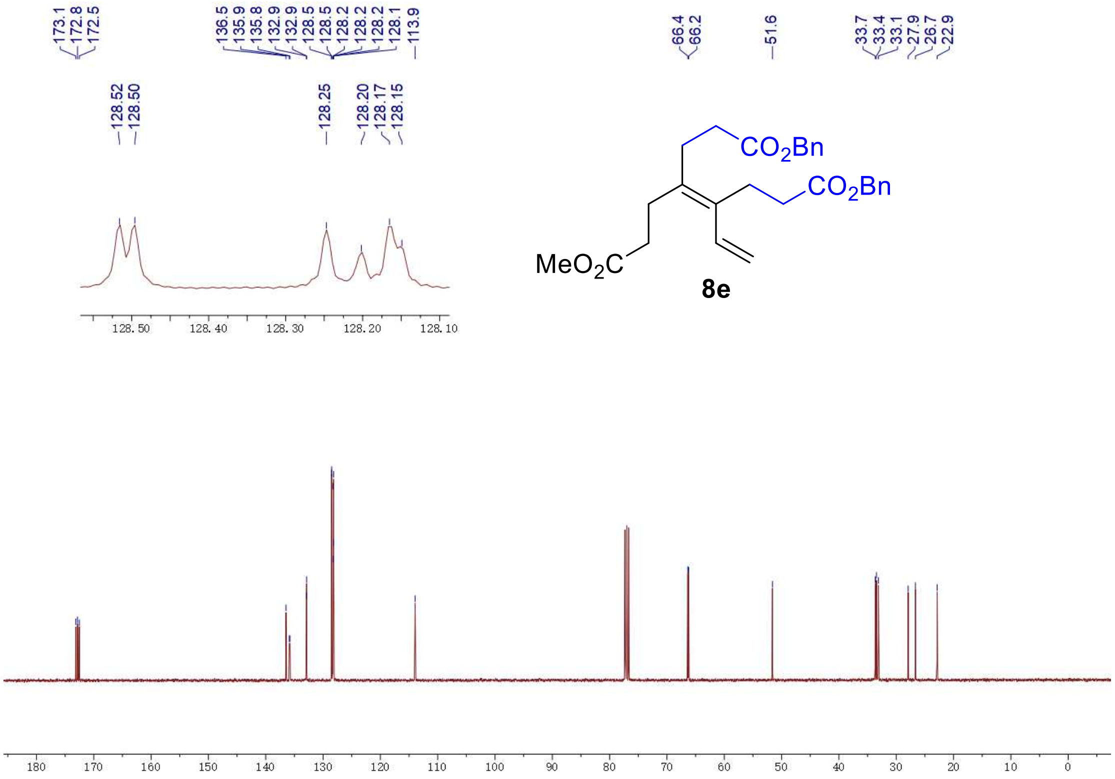

Supplementary Figure 62. ^{1}H NMR and ^{13}C NMR spectrum of compound of **8f**

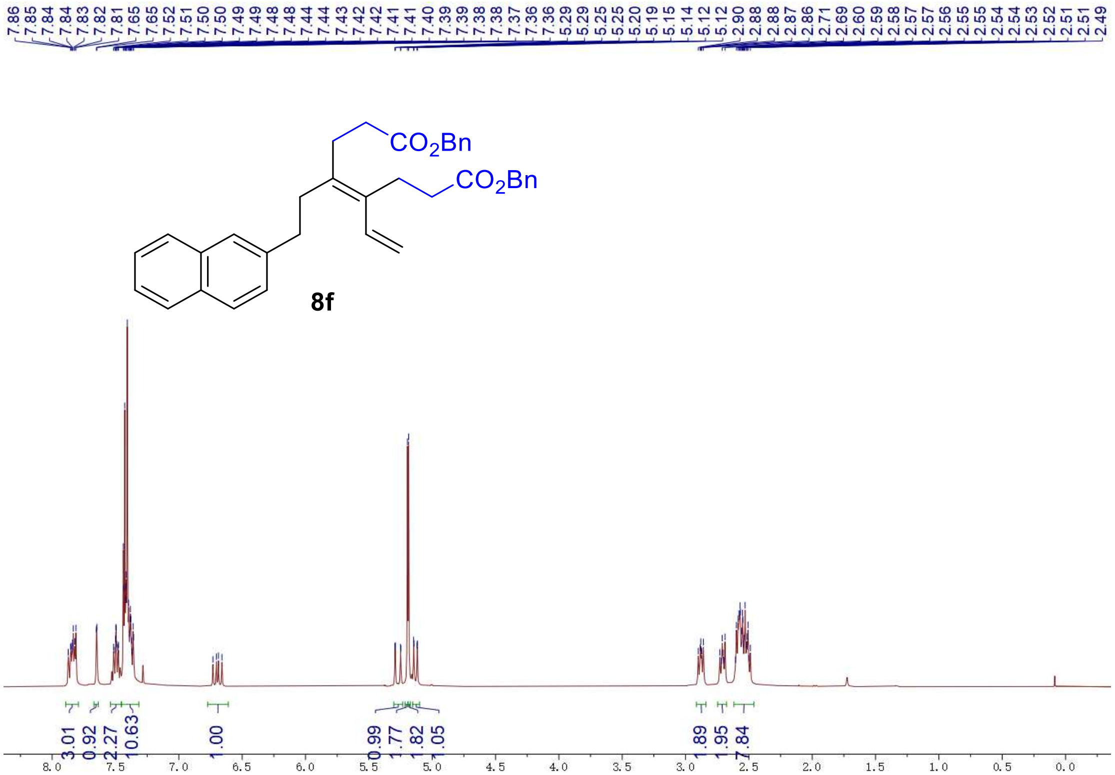

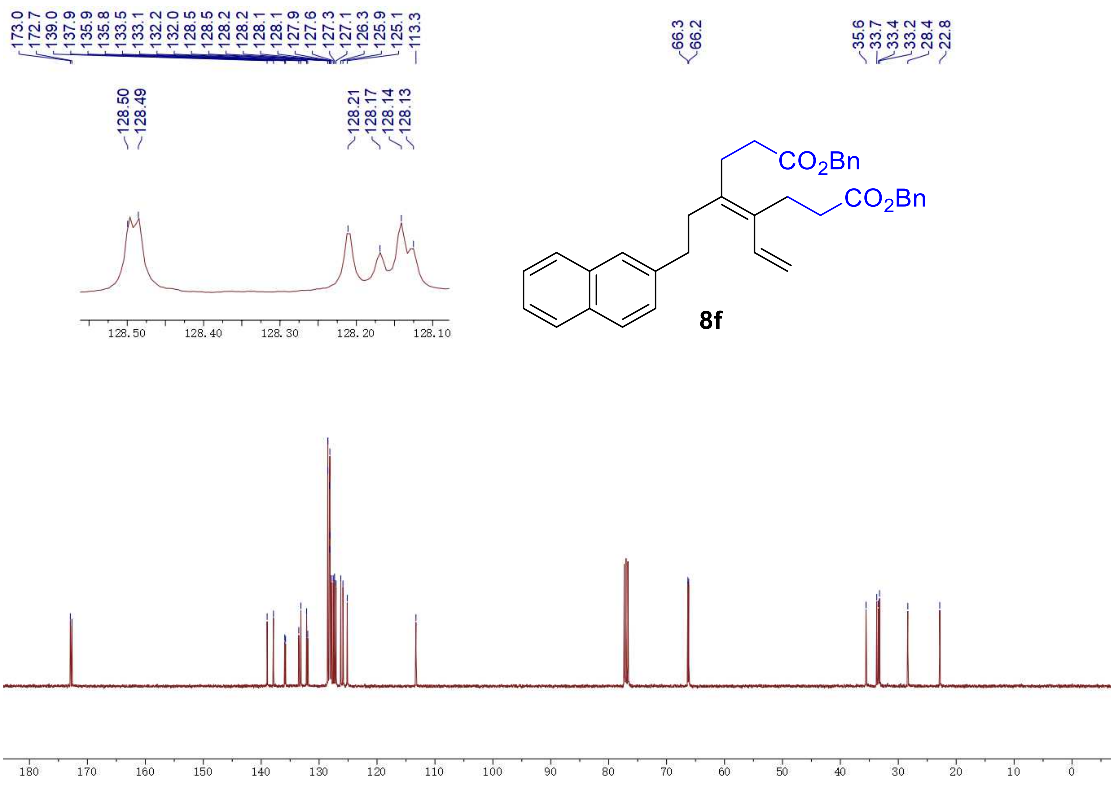

Supplementary Figure 63. ^{1}H NMR and ^{13}C NMR spectrum of compound of **8g**

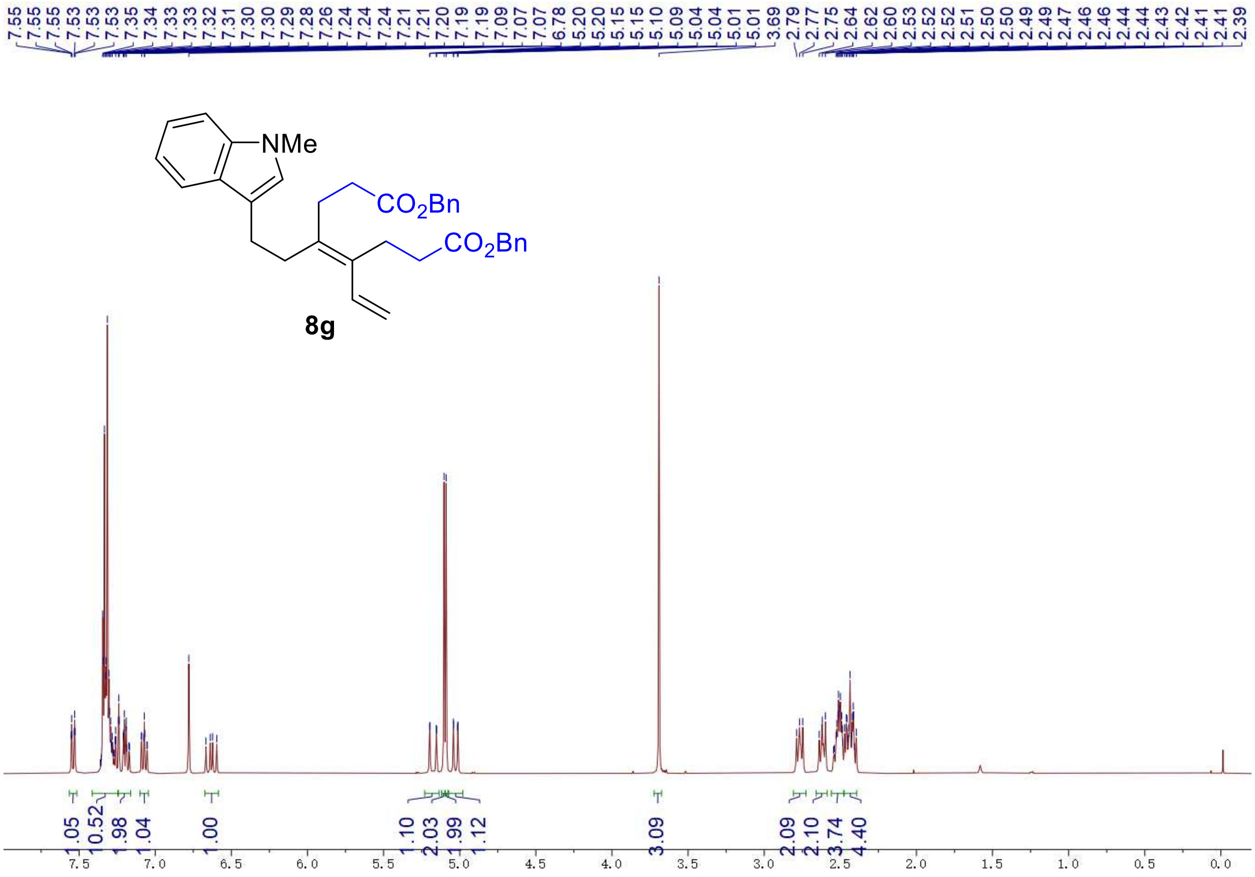

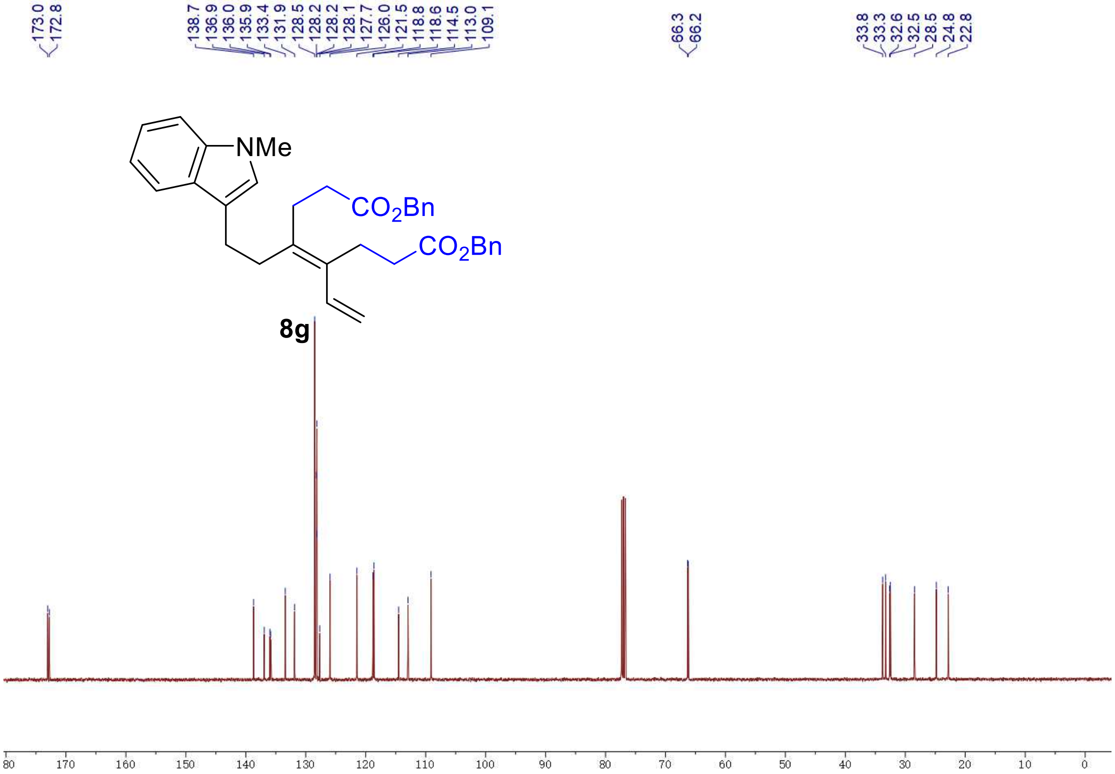

Supplementary Figure 64. ^{1}**H NMR and** ^{13}**C NMR** spectrum of compound of **8h**

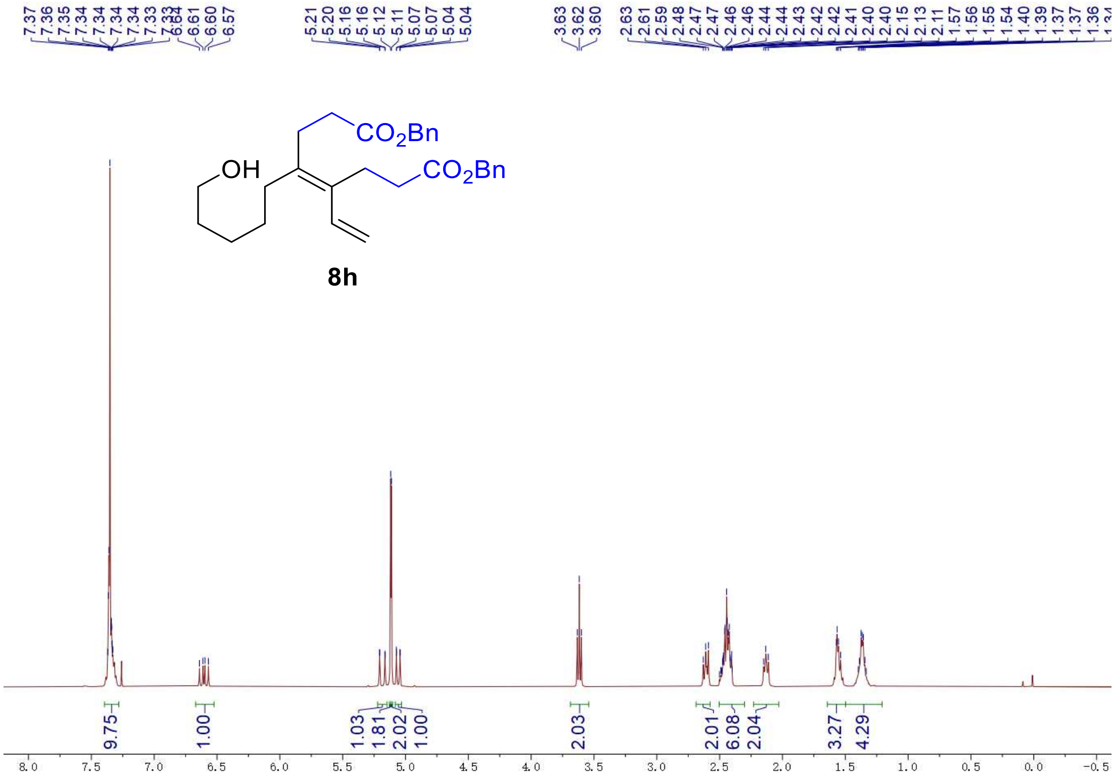

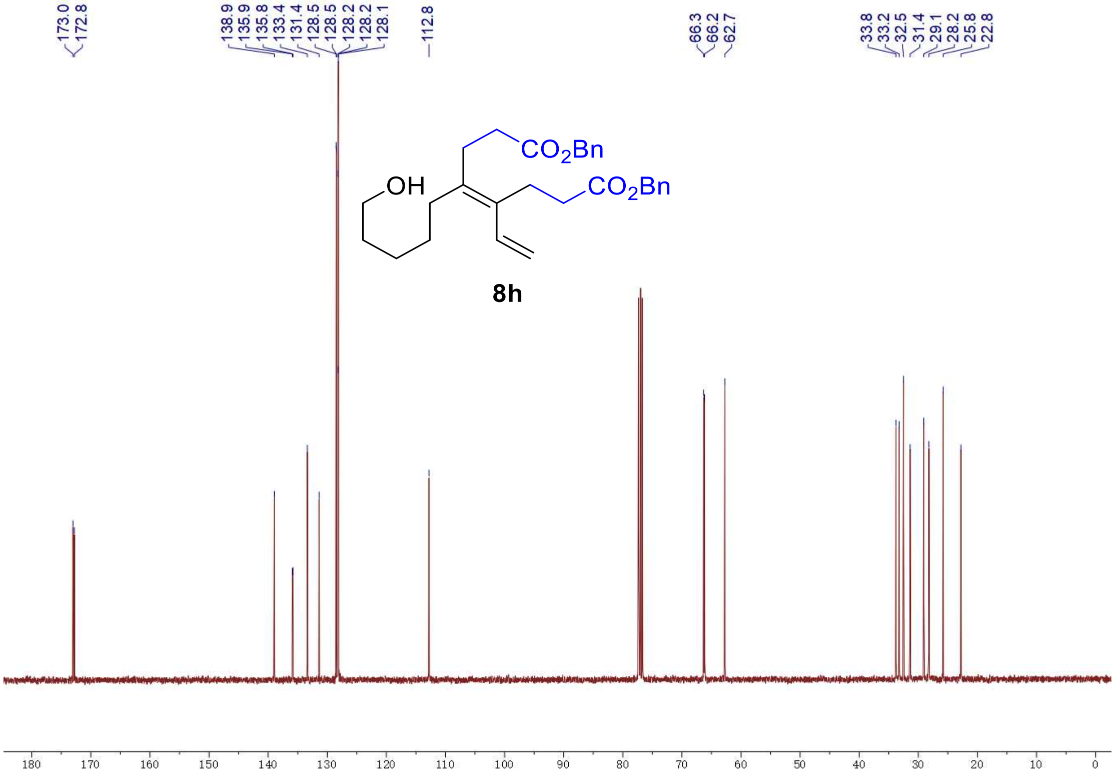

Supplementary Figure 65. ^{1}H NMR and ^{13}C NMR spectrum of compound of **8i**

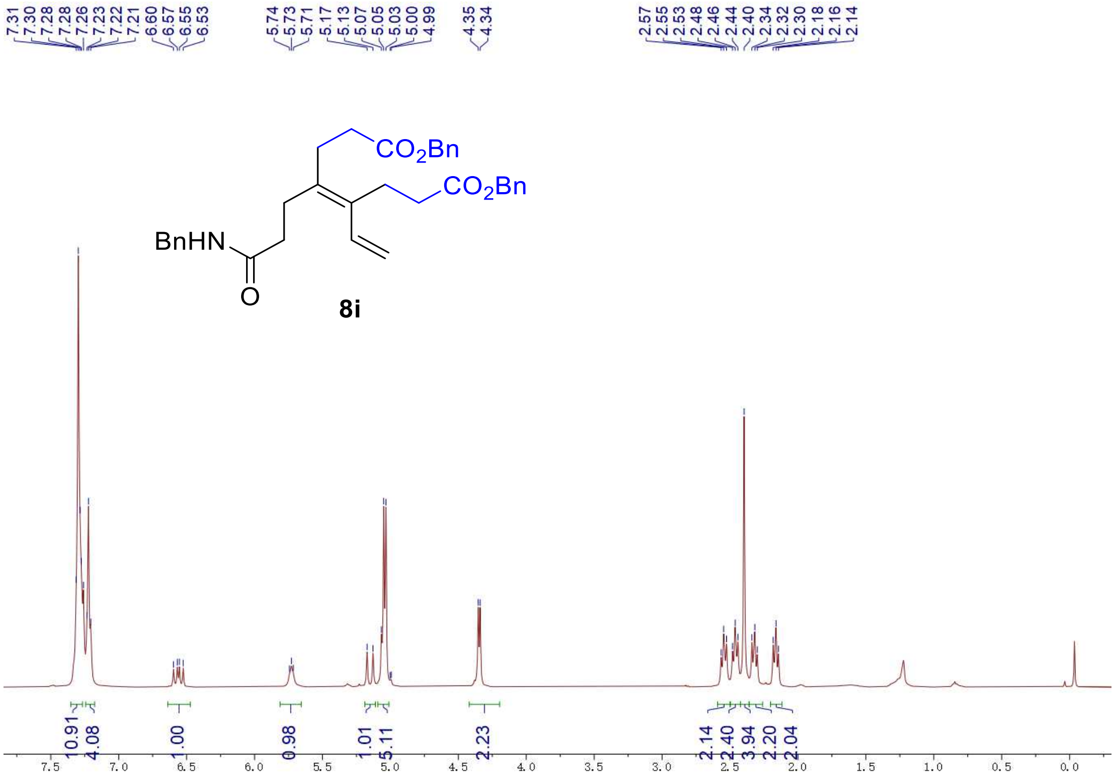

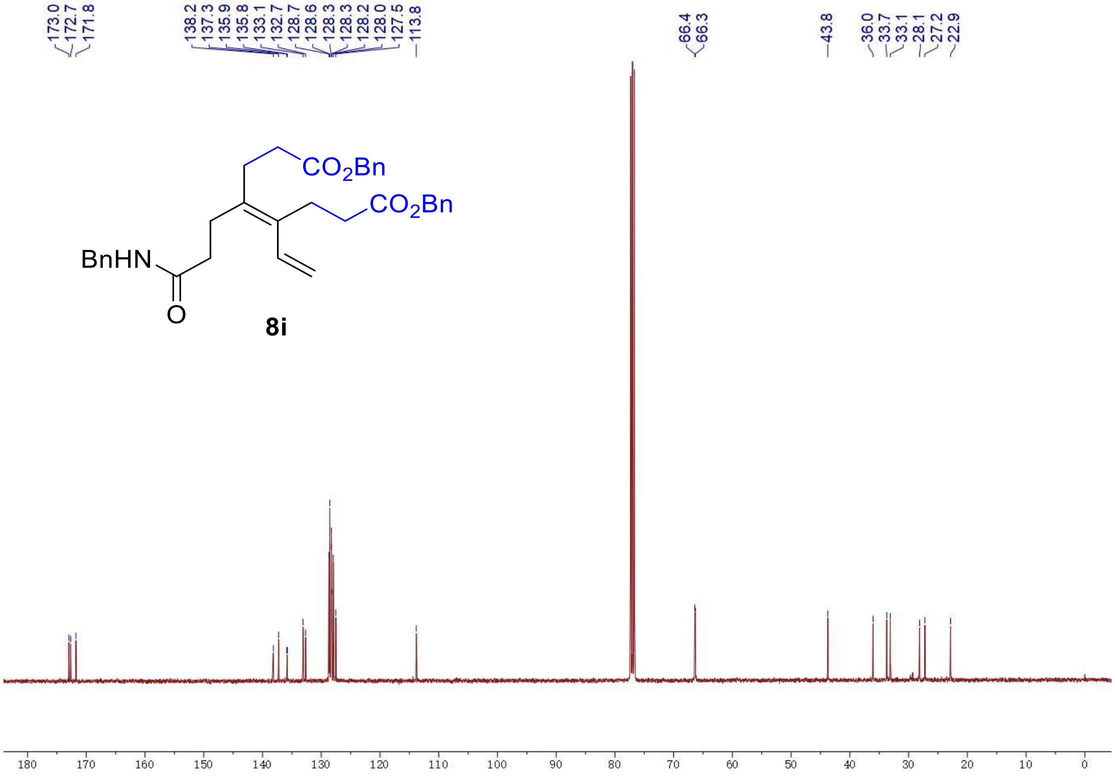

Supplementary Figure 66. ^{1}H NMR and ^{13}C NMR spectrum of compound of **8j**

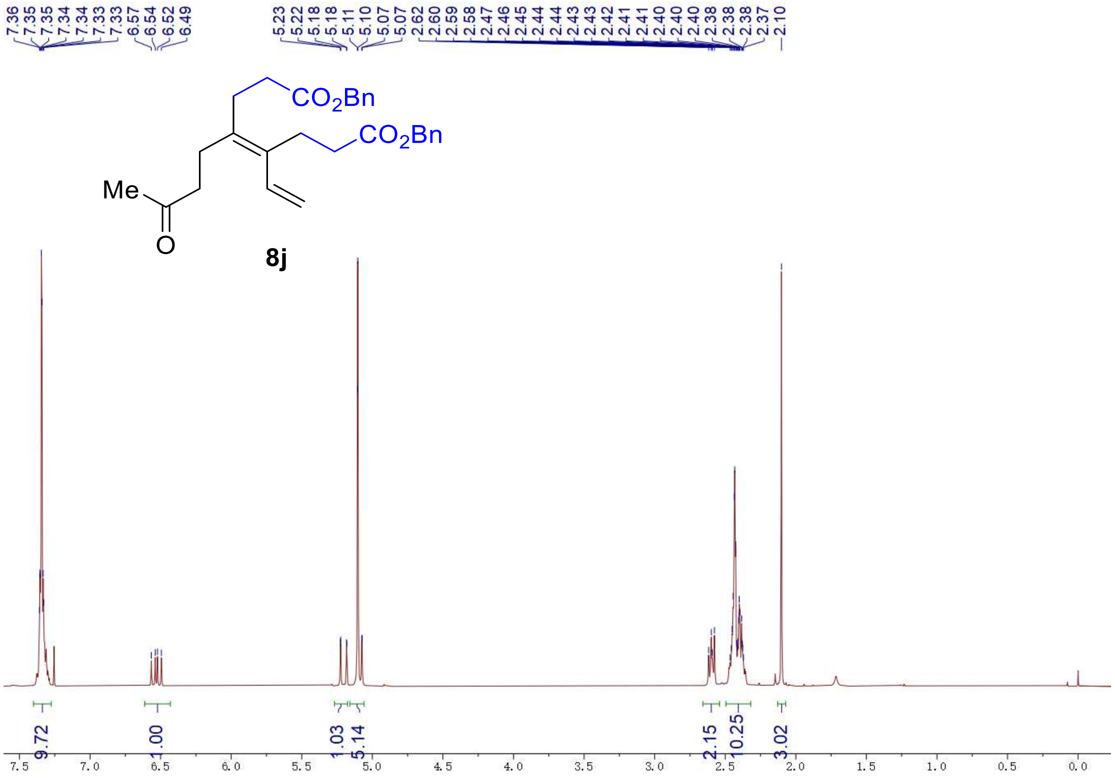

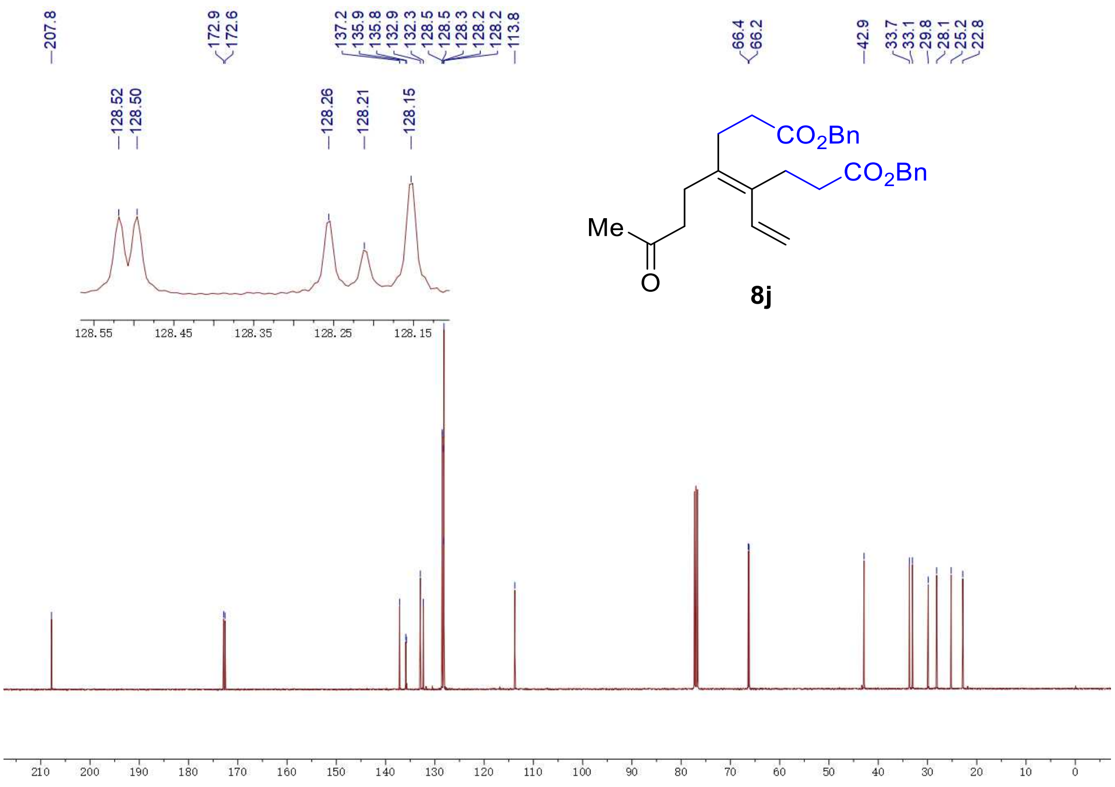

Supplementary Figure 67. ^{1}H NMR and ^{13}C NMR spectrum of compound of **8k**

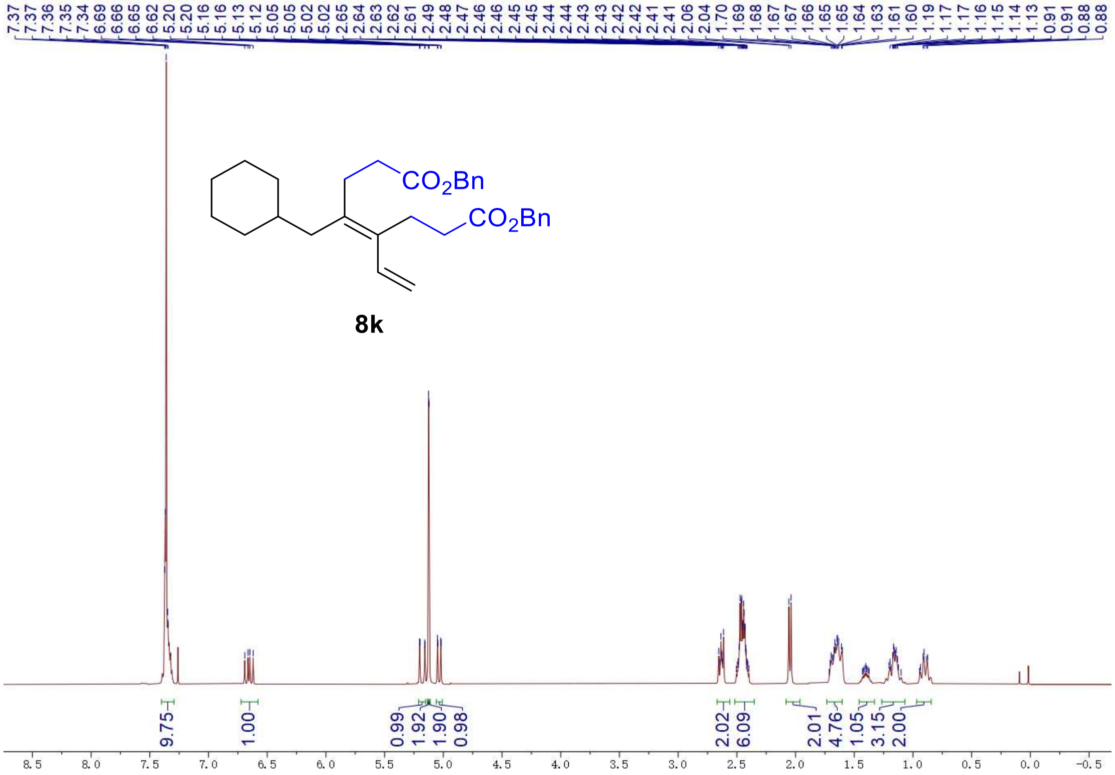

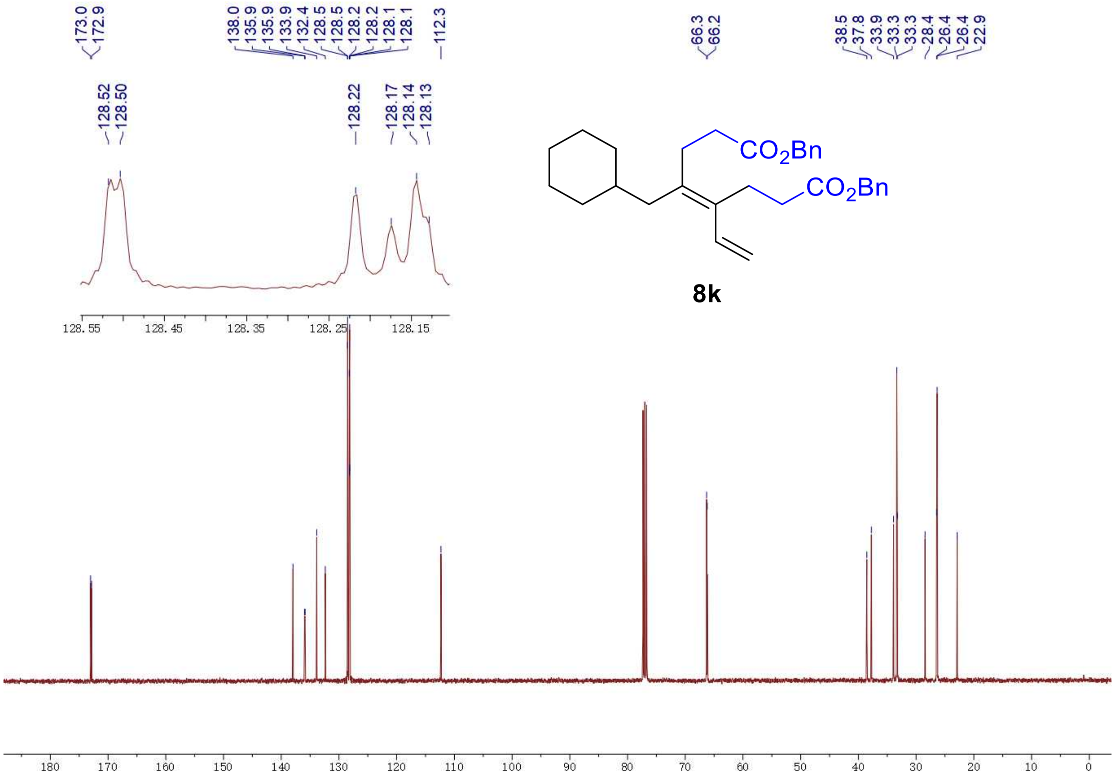

Supplementary Figure 68. ^{1}**H NMR and** ^{13}**C NMR** spectrum of compound of **8l**

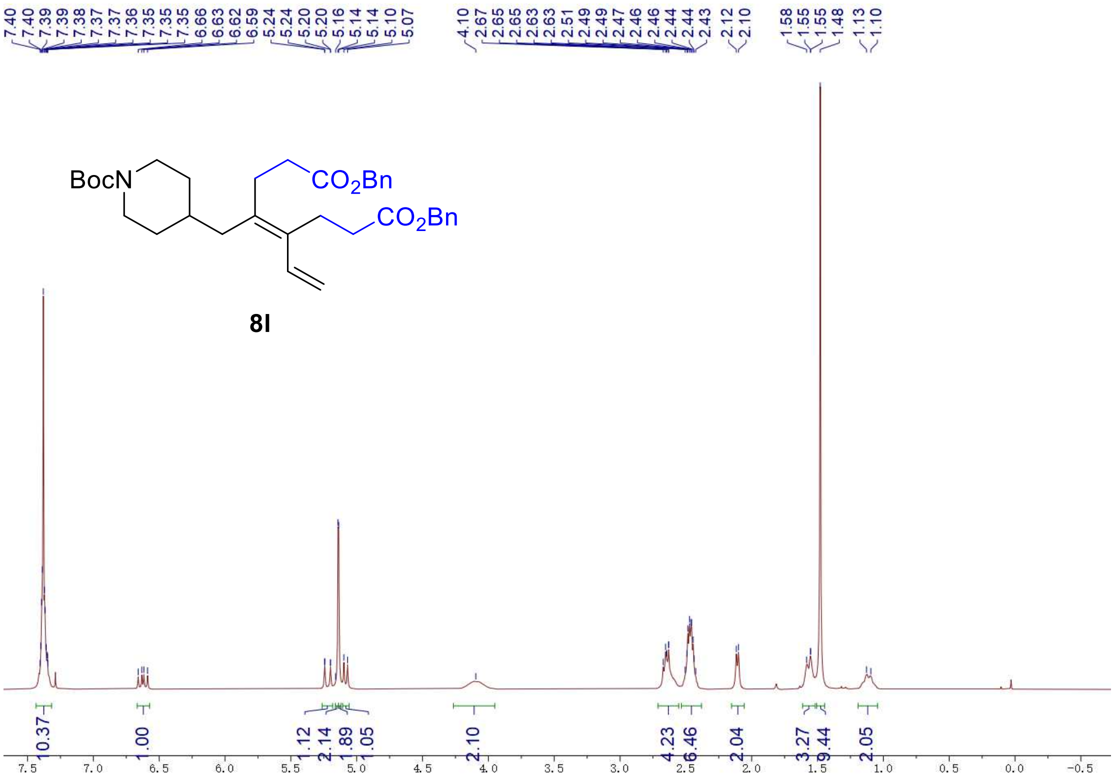

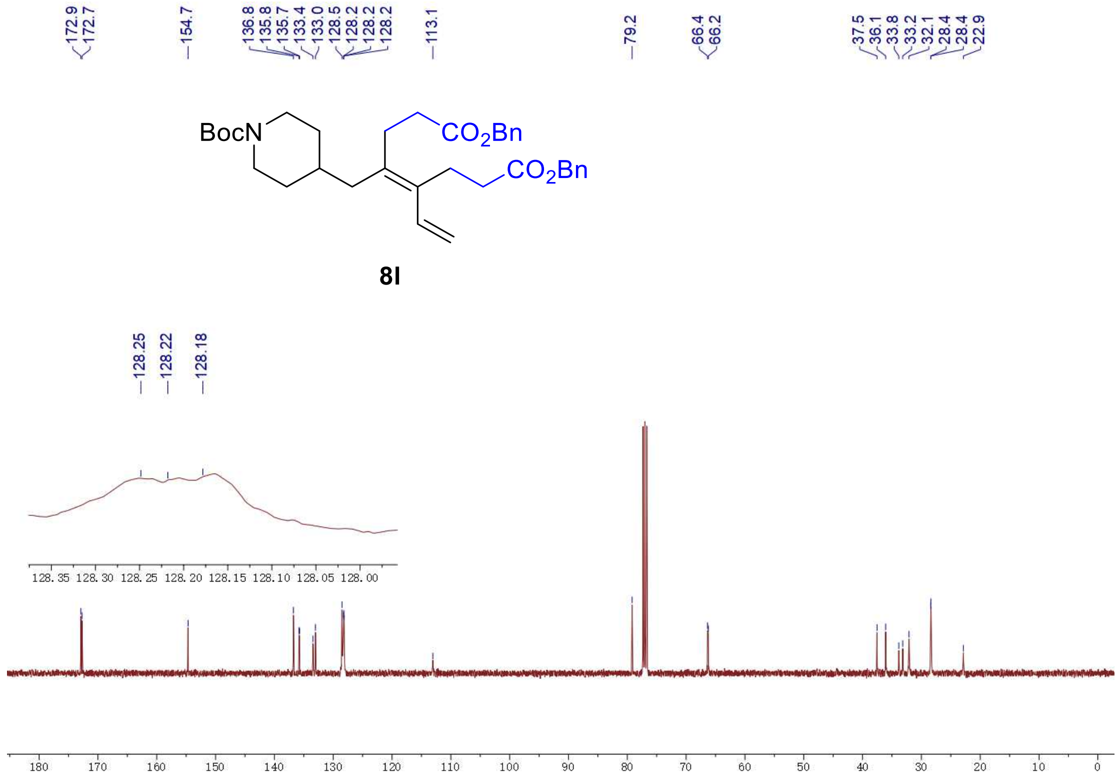

Supplementary Figure 69. ¹H NMR and ¹³C NMR spectrum of compound of **8m**

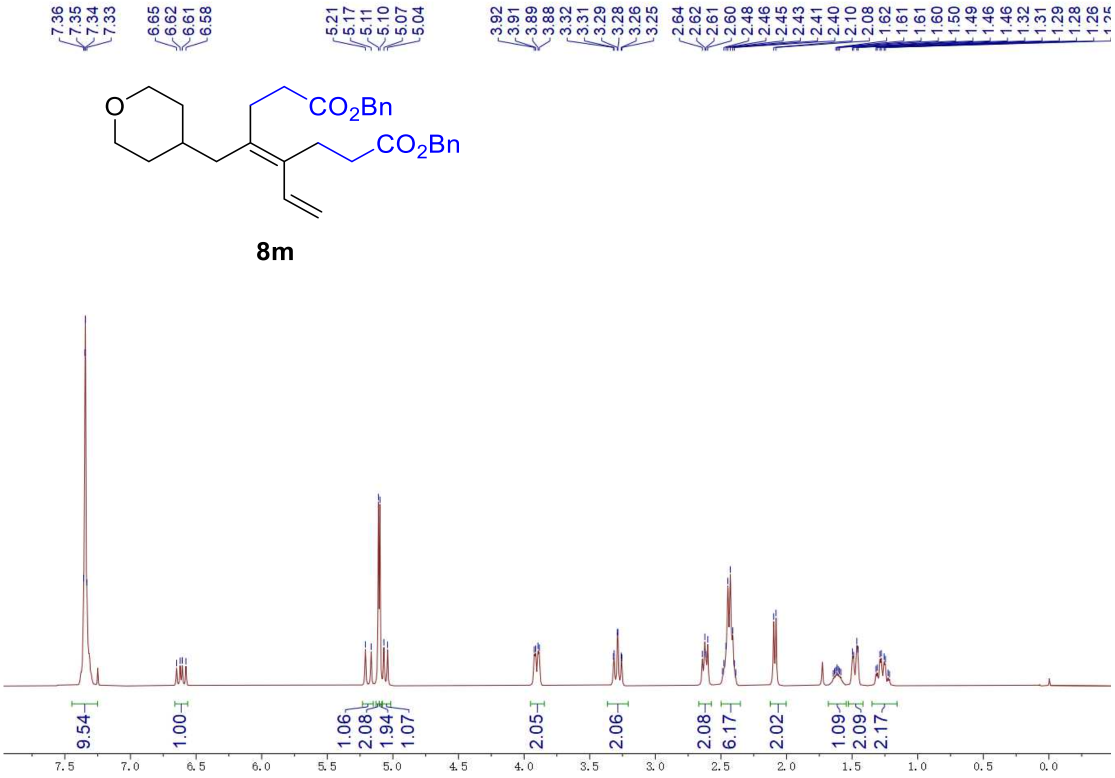

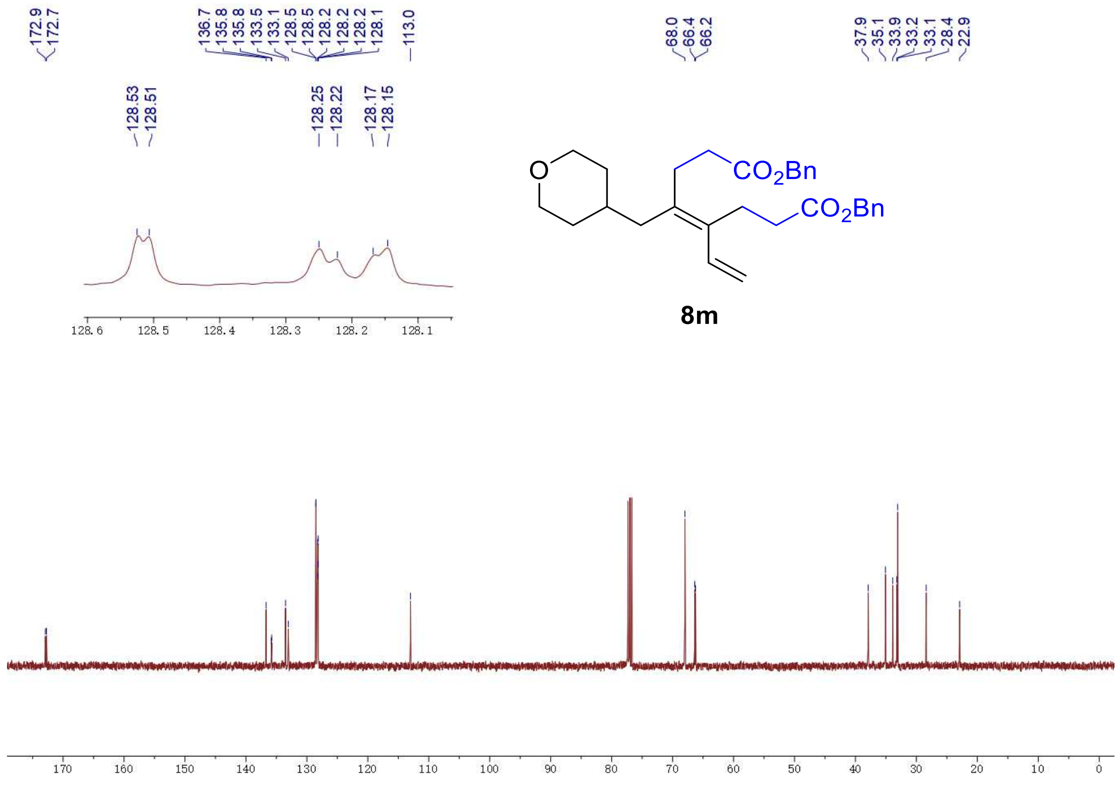

Supplementary Figure 70. ^{1}H NMR and ^{13}C NMR spectrum of compound of **8n**

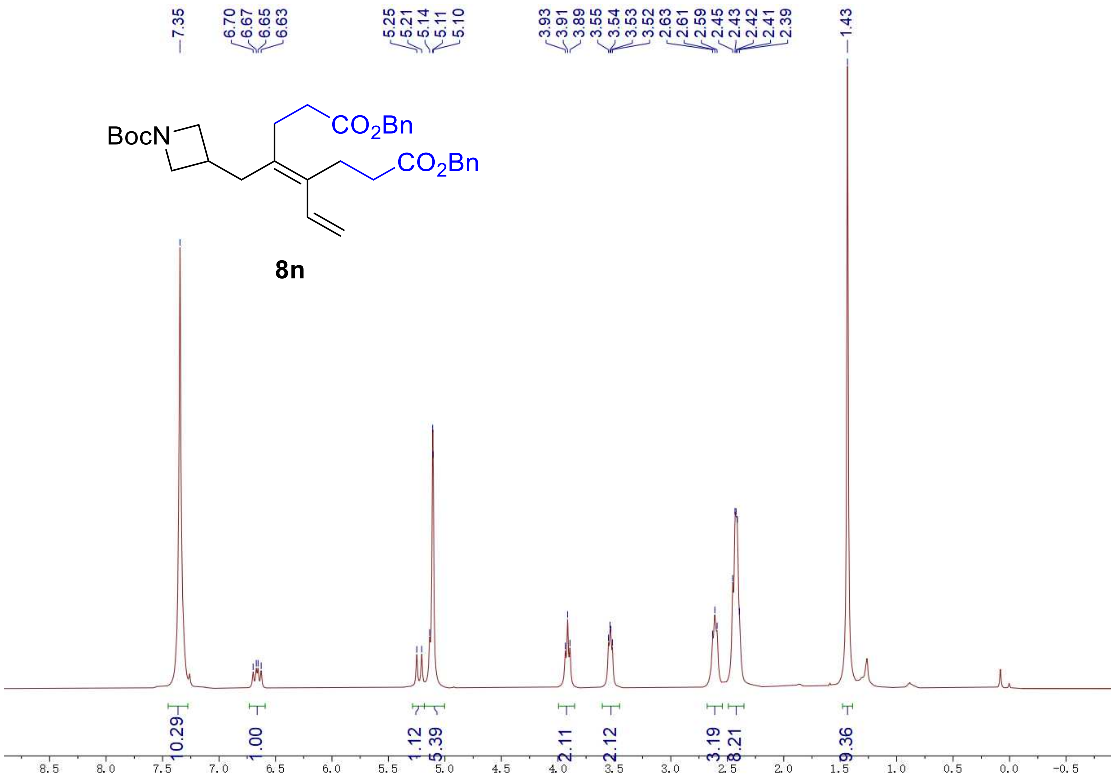

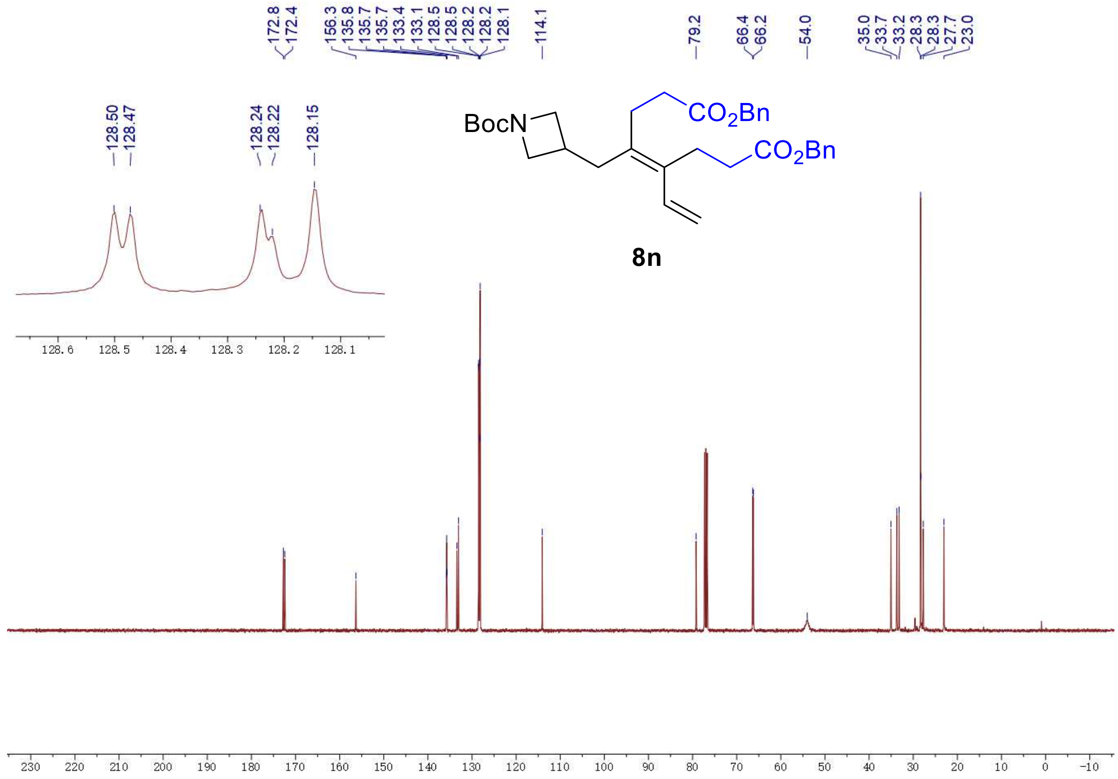

Supplementary Figure 71. **^{1}H NMR and ^{13}C NMR** spectrum of compound of **8o**

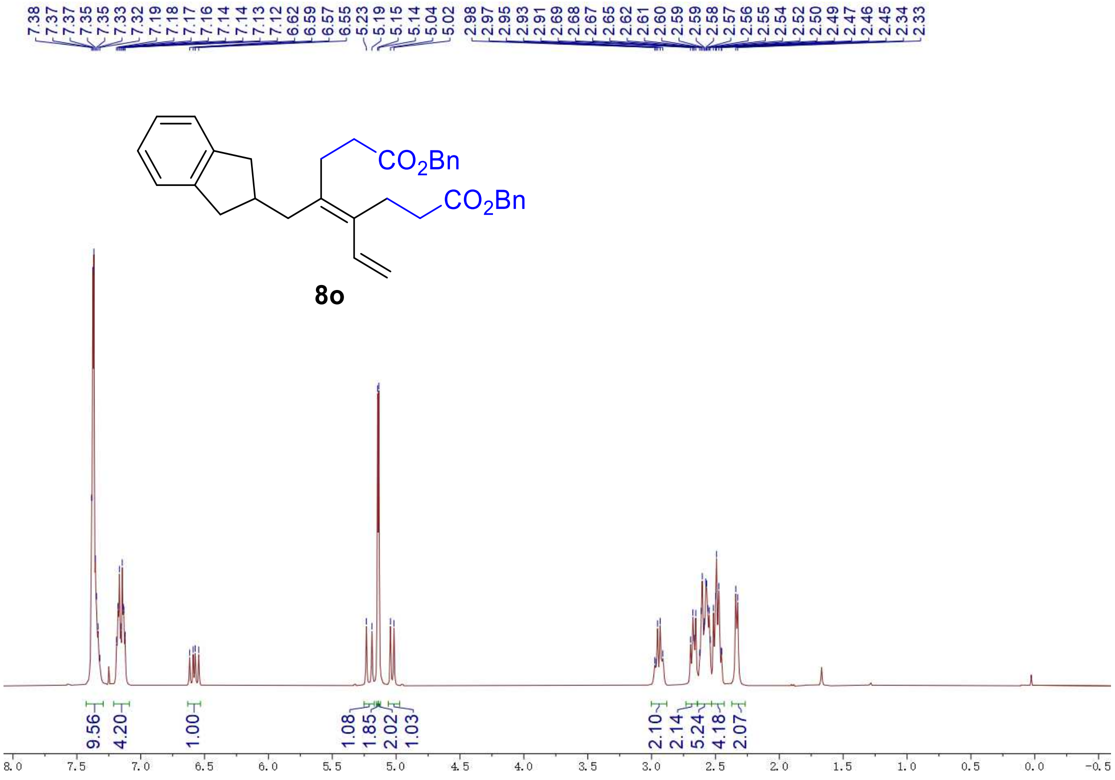

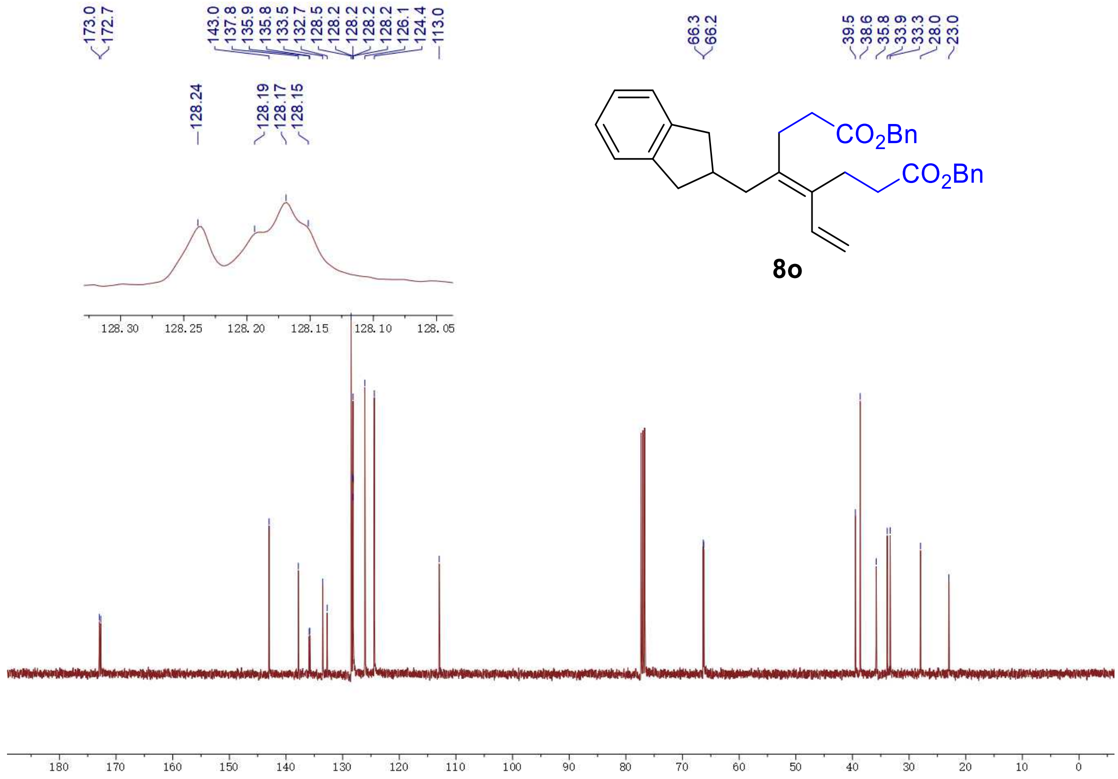

Supplementary Figure 72. ^{1}H NMR and ^{13}C NMR spectrum of compound of **8p**

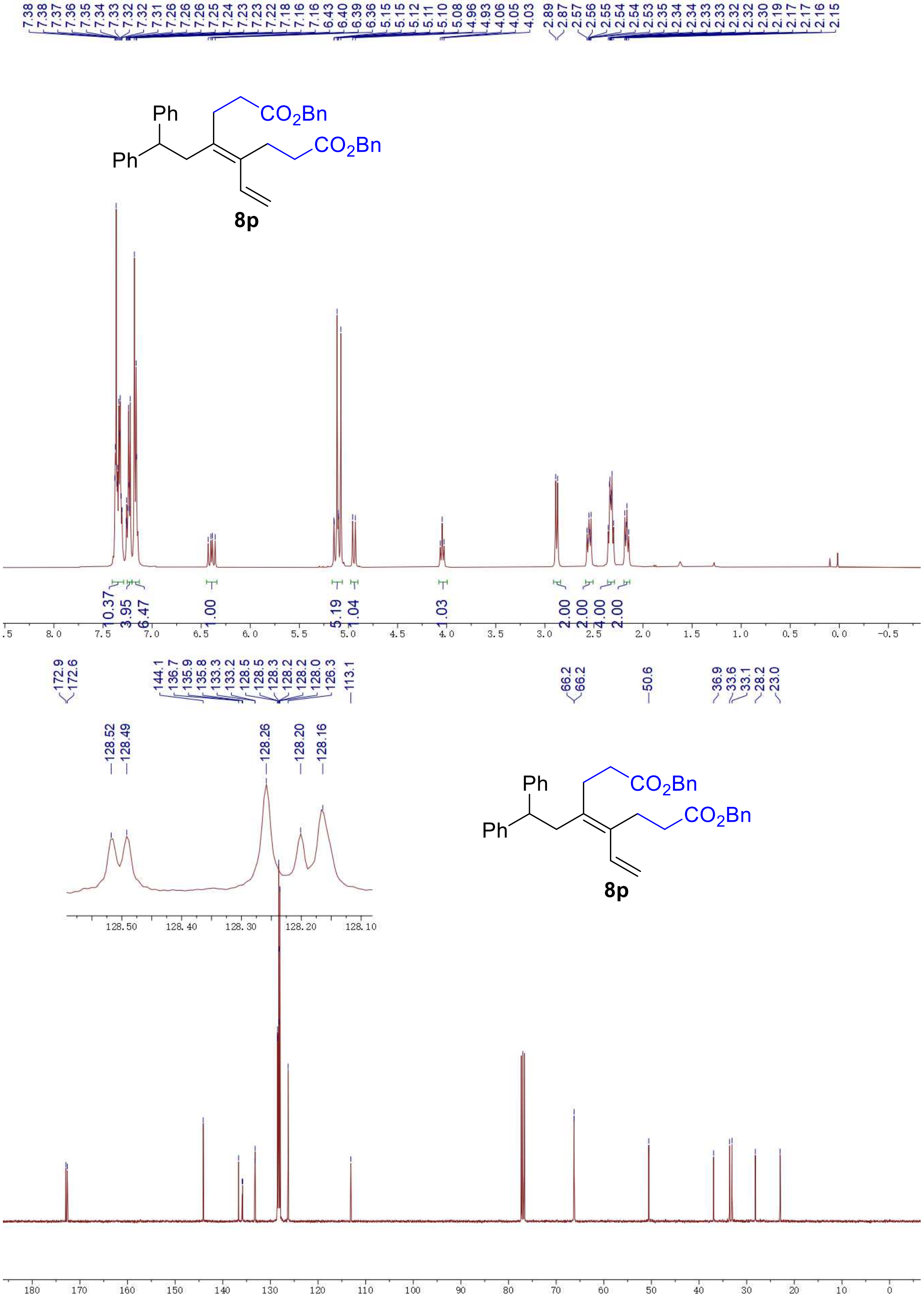

Supplementary Figure 73. H-H cosy spectrum of compound of **8p** in $CDCl_3$

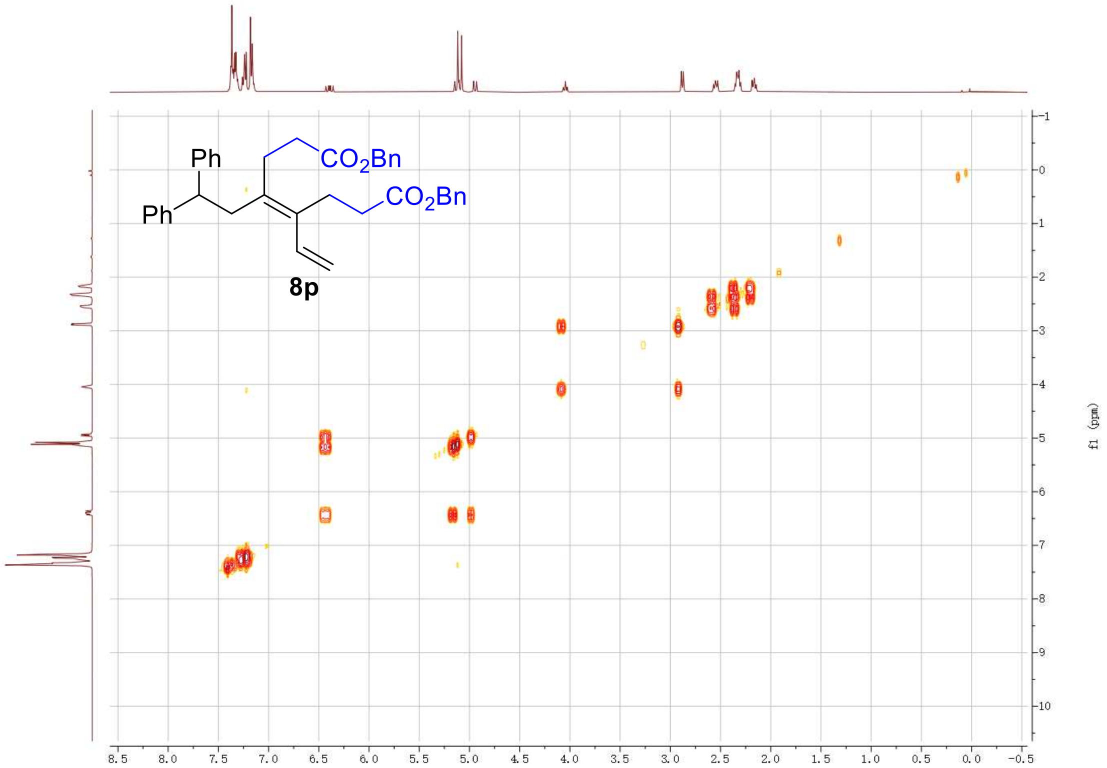

Supplementary Figure 74. HSQC spectrum of compound of **8p** in $CDCl_3$

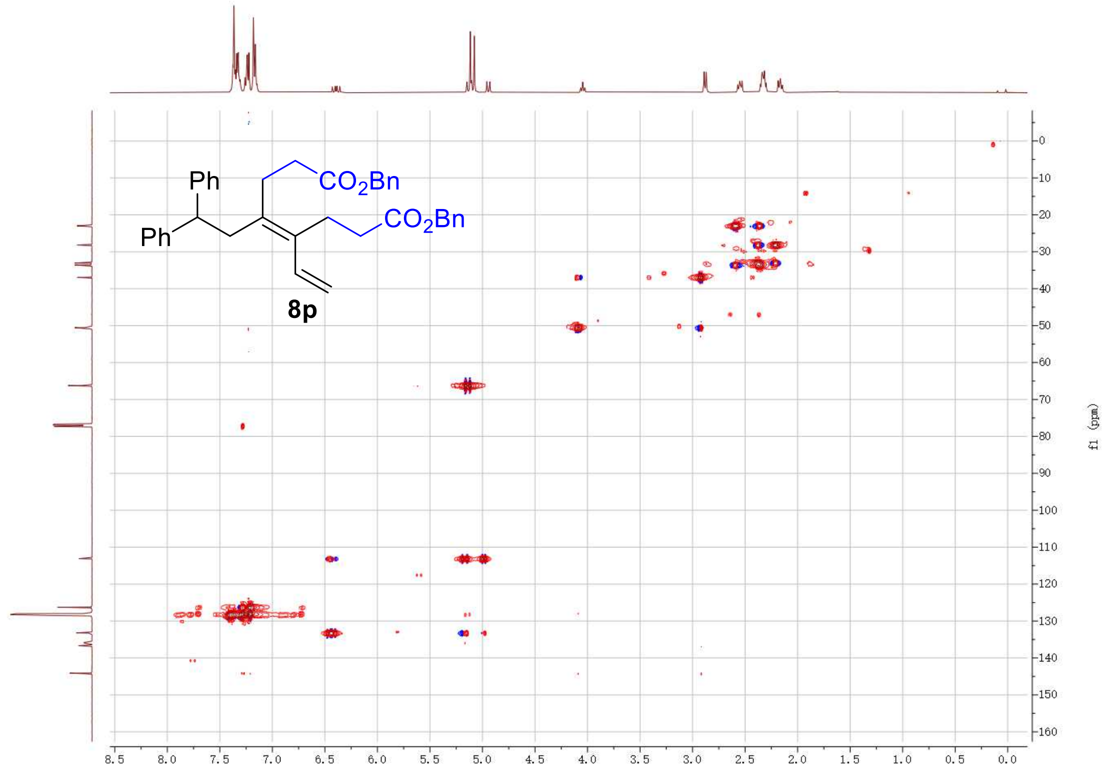

Supplementary Figure 75. NOESY spectrum of compound of **8p** in $CDCl_3$

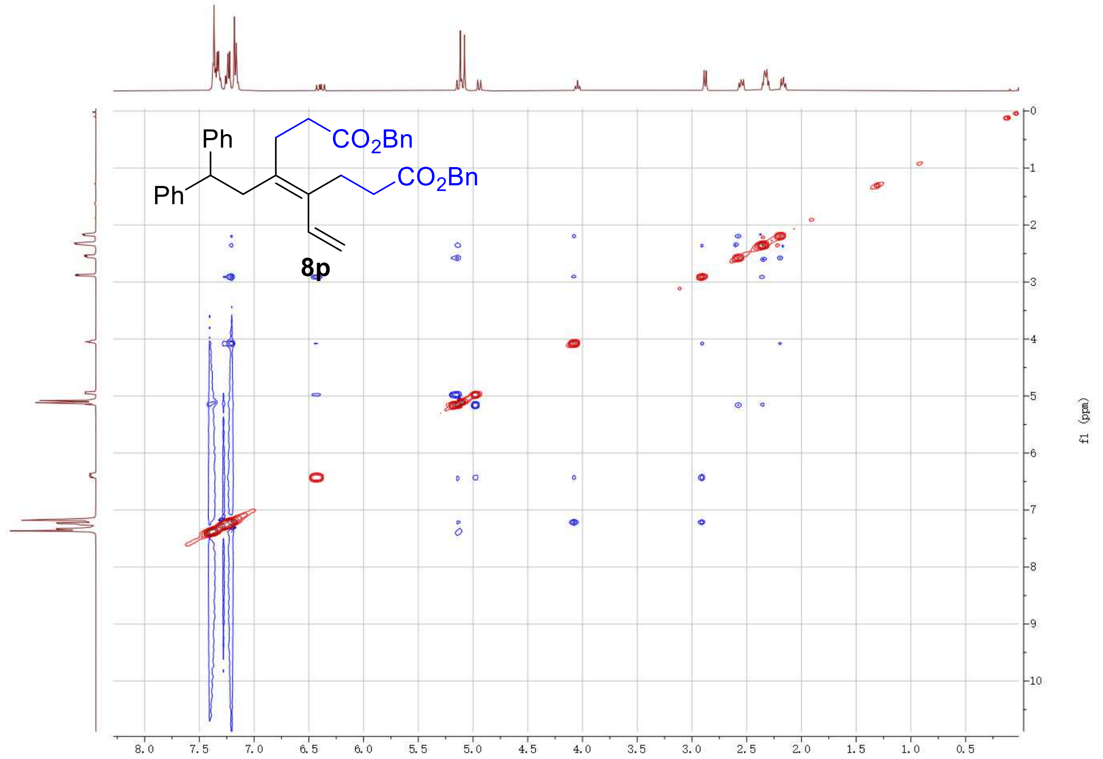

Supplementary Figure 76. ^{1}H NMR and ^{13}C NMR spectrum of compound of **8q**

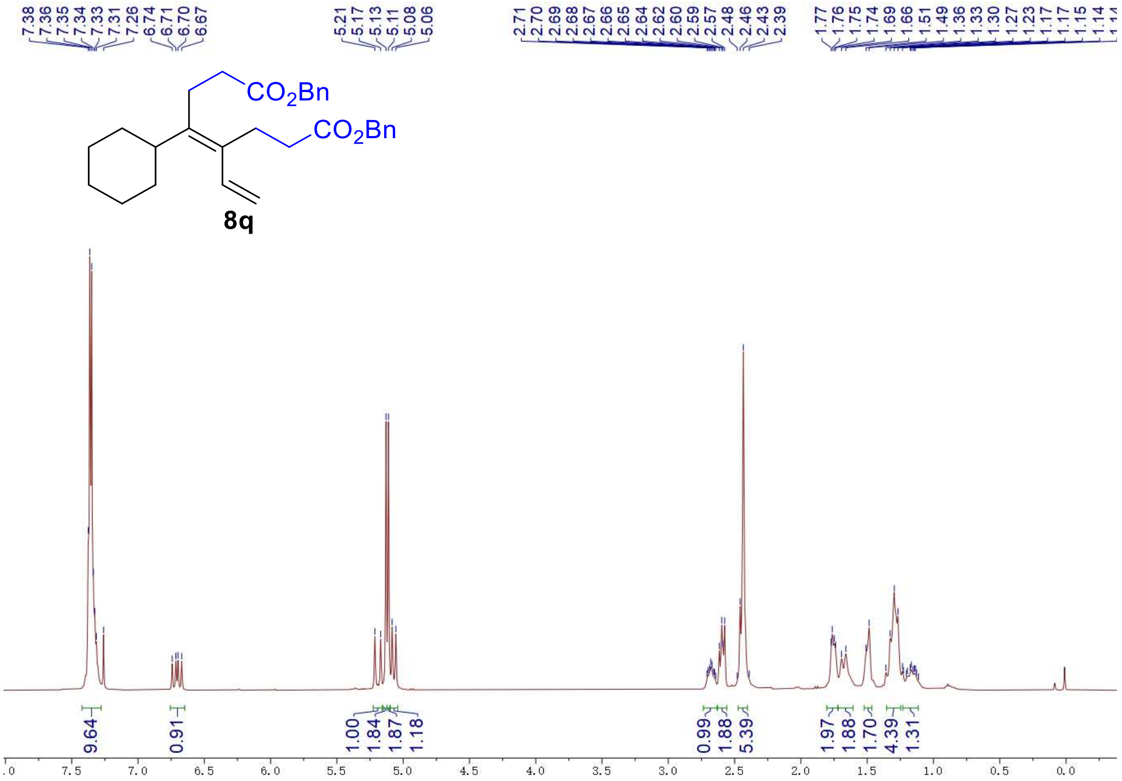

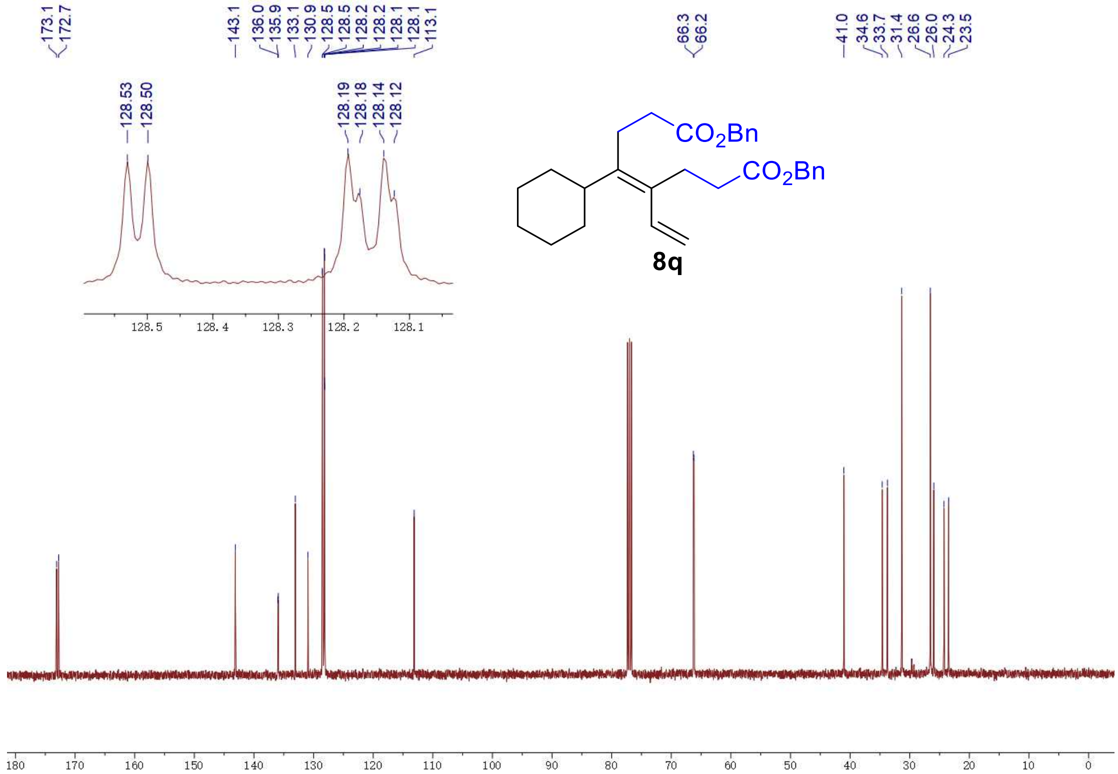

Supplementary Figure 77. ^{1}**H NMR and** ^{13}**C NMR** spectrum of compound of **8r**

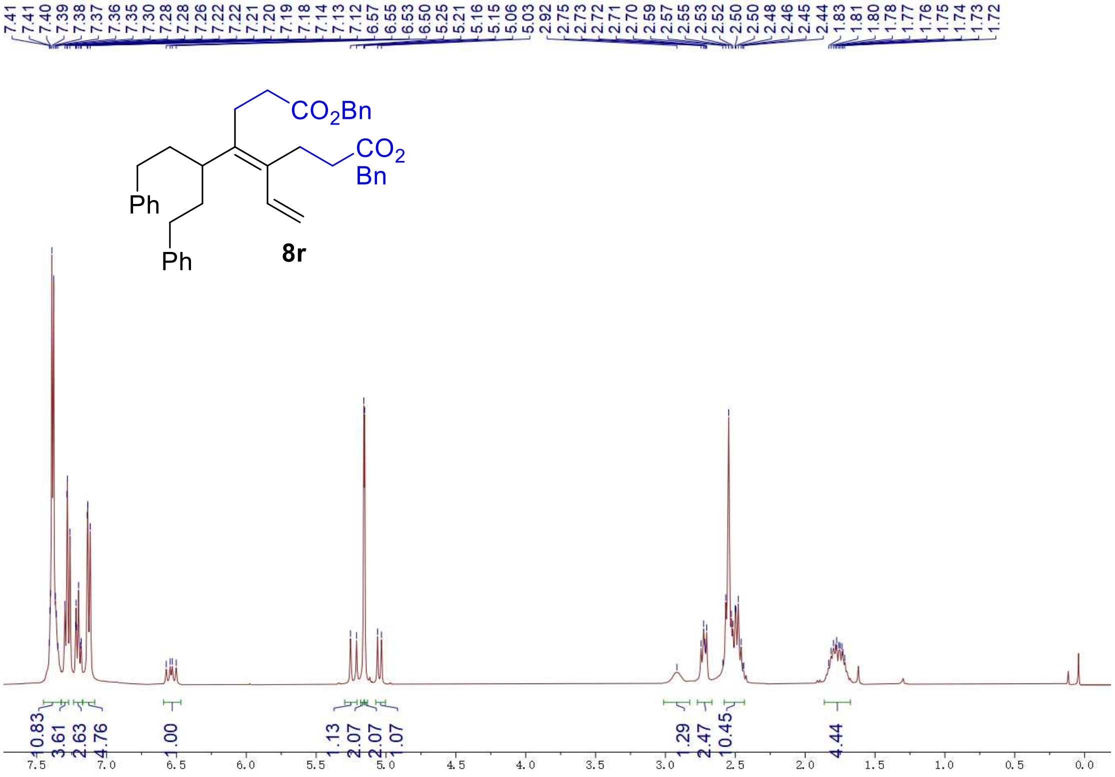

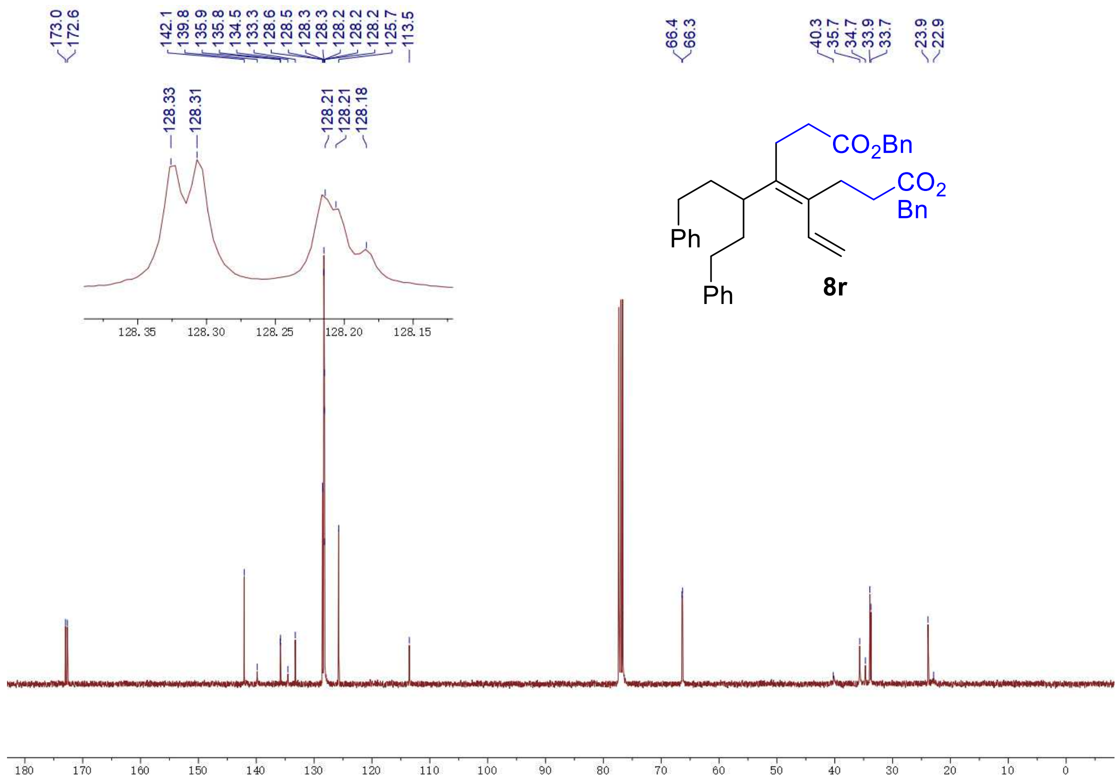

Supplementary Figure 78. ^{1}H NMR and ^{13}C NMR spectrum of compound of **8s**

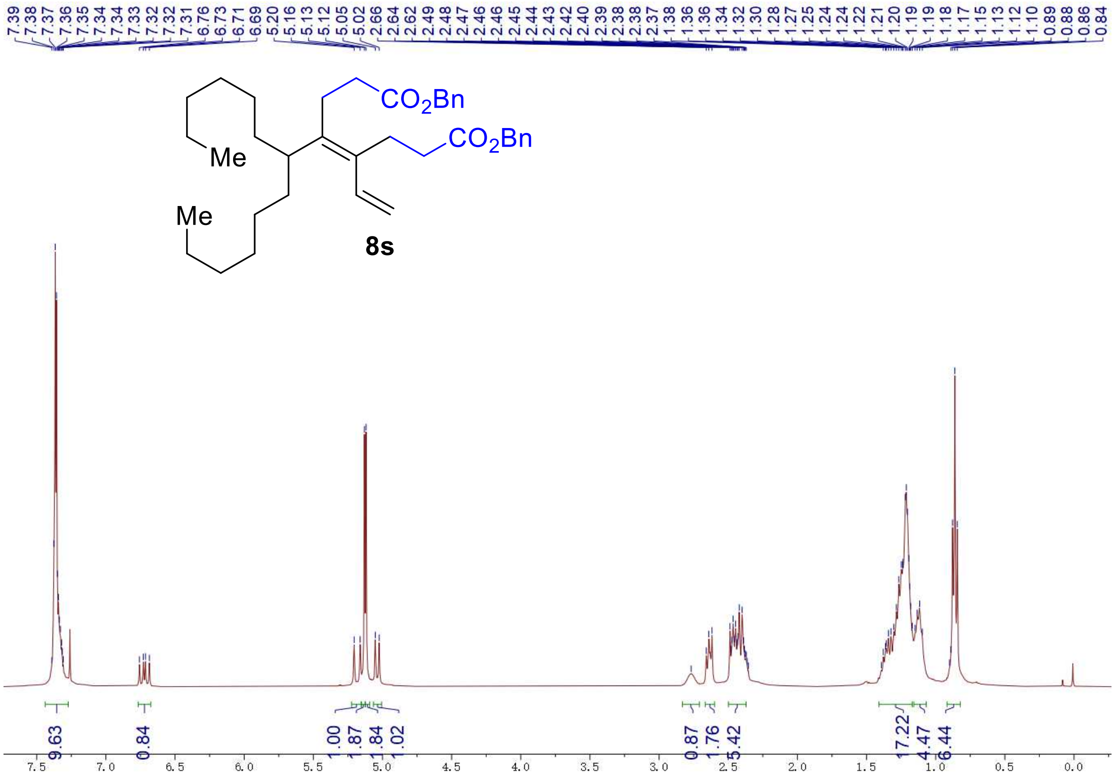

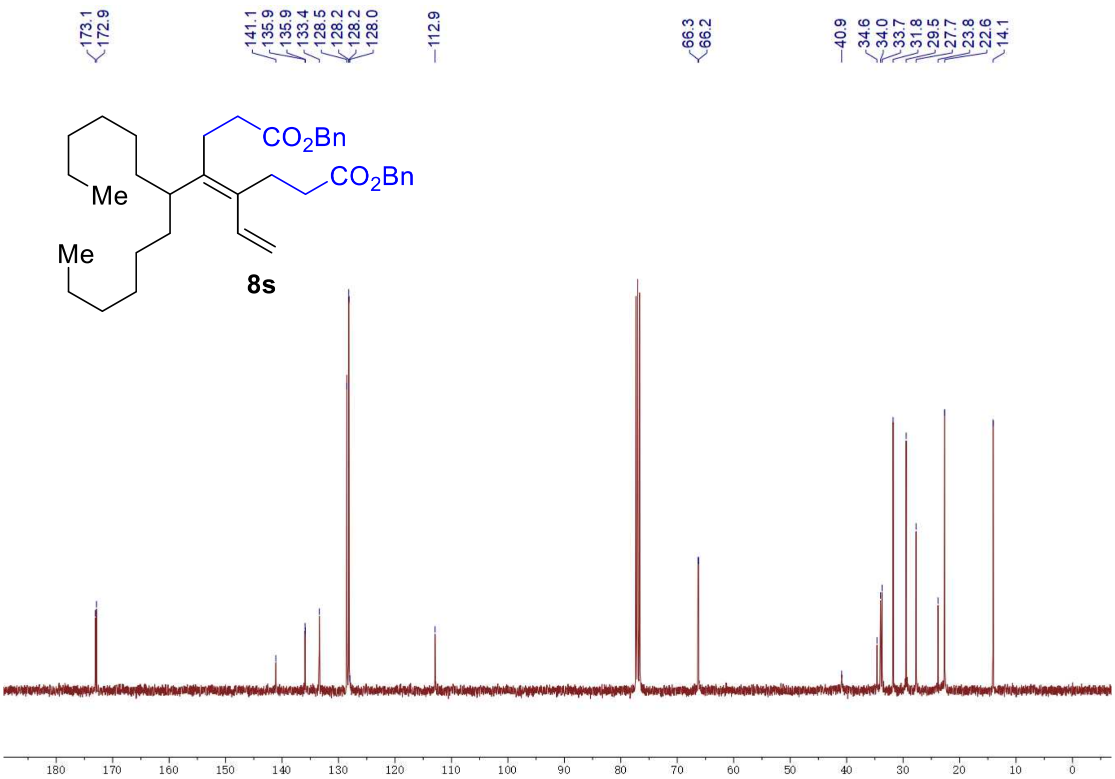

Supplementary Figure 79. ^{1}H NMR and ^{13}C NMR spectrum of compound of **8t**

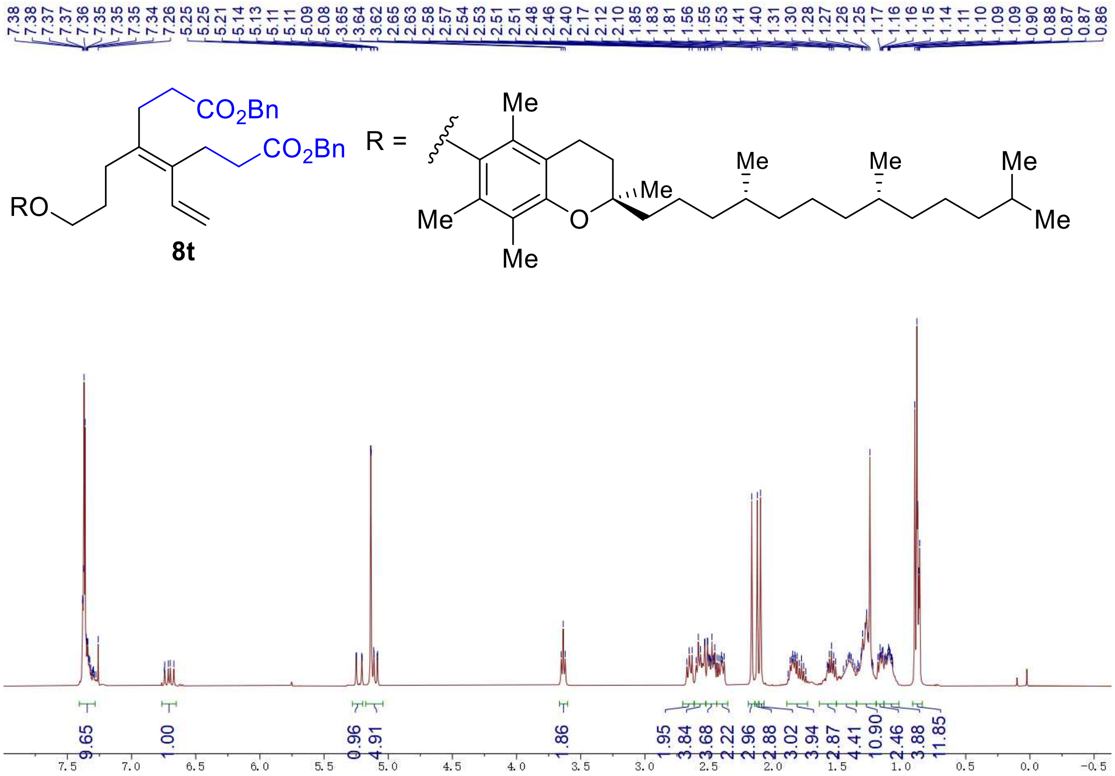

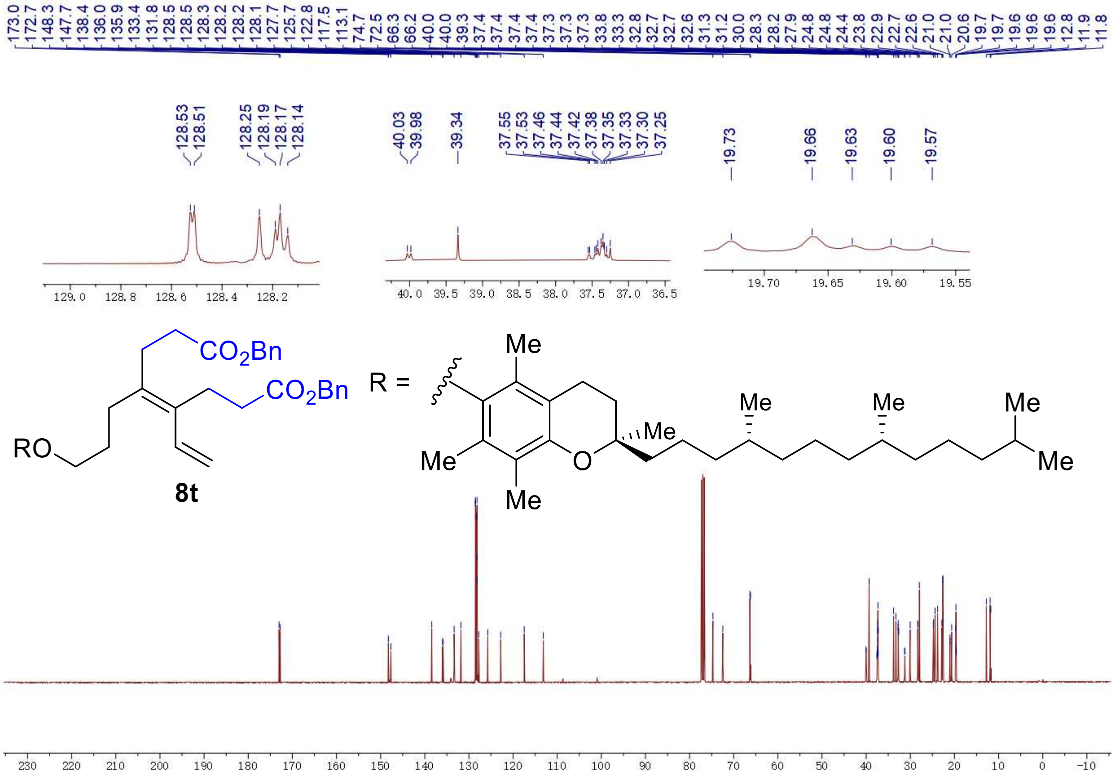

Supplementary Figure 80. ^{1}H NMR and ^{13}C NMR spectrum of compound of **9a**

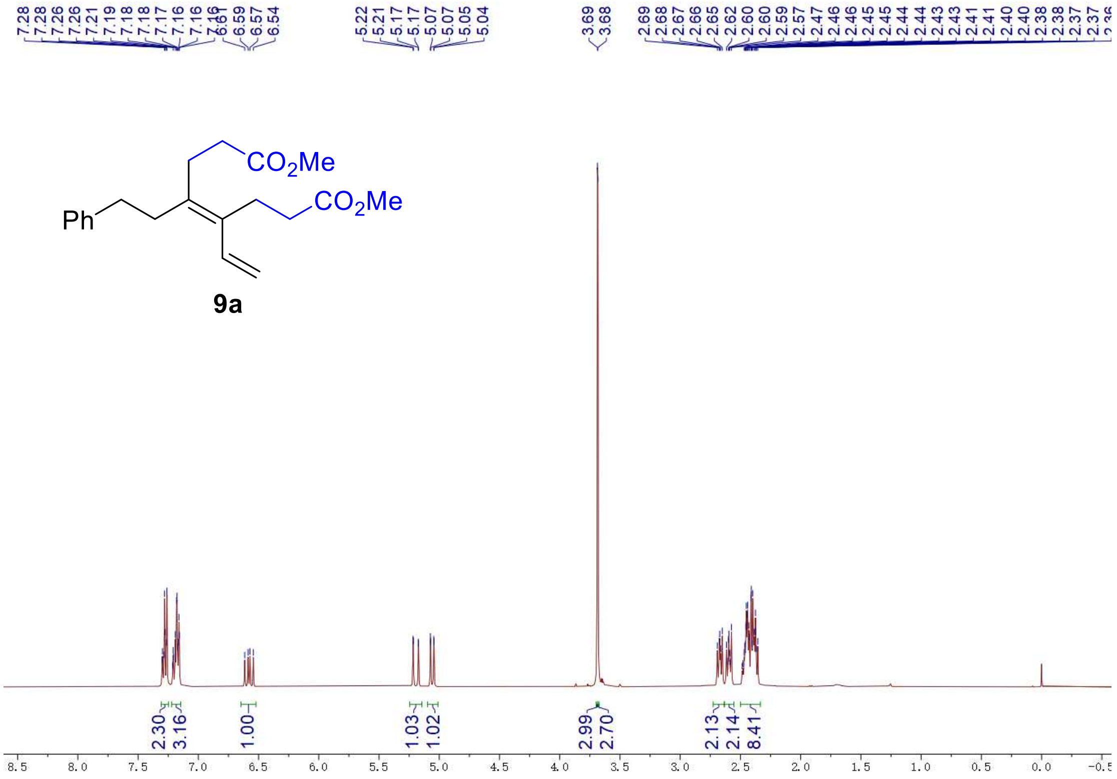

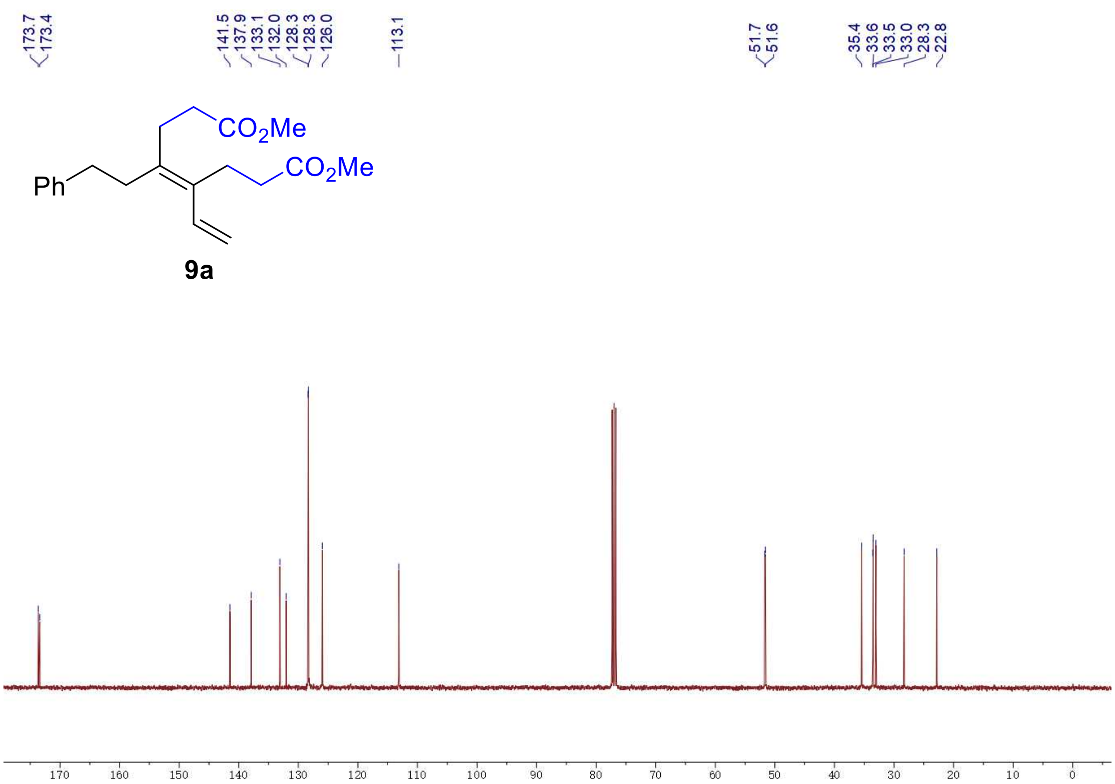

Supplementary Figure 81. ^{1}H NMR and ^{13}C NMR spectrum of compound of **9b**

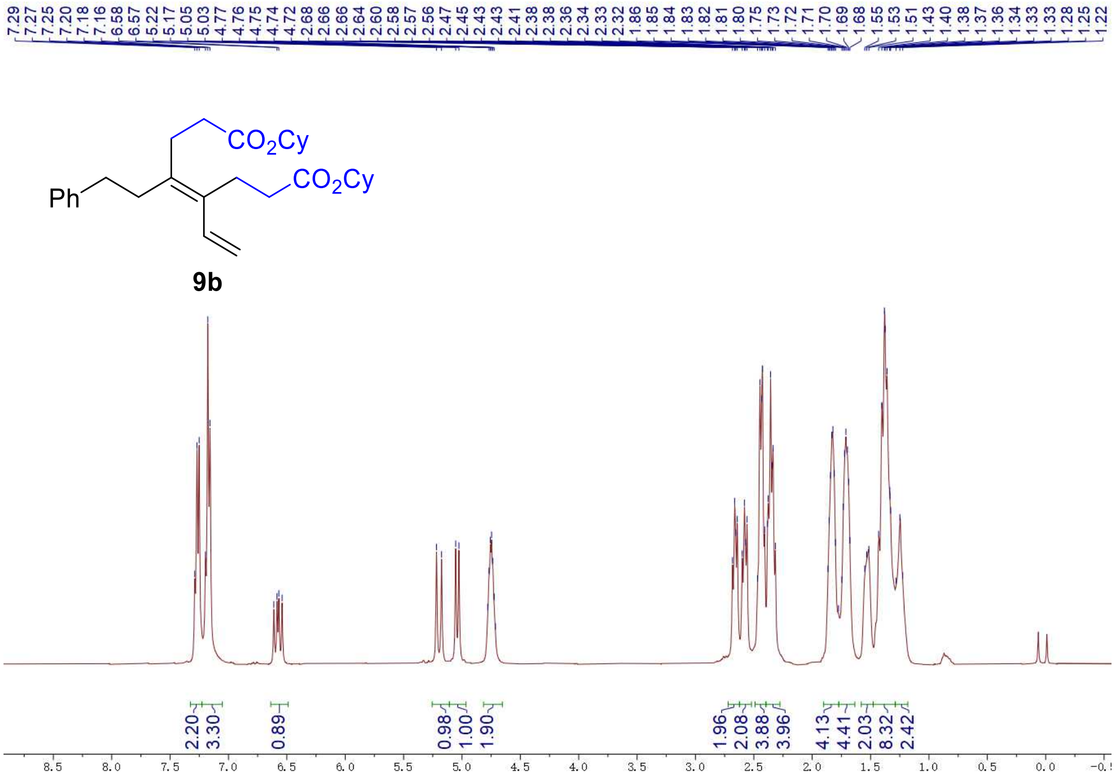

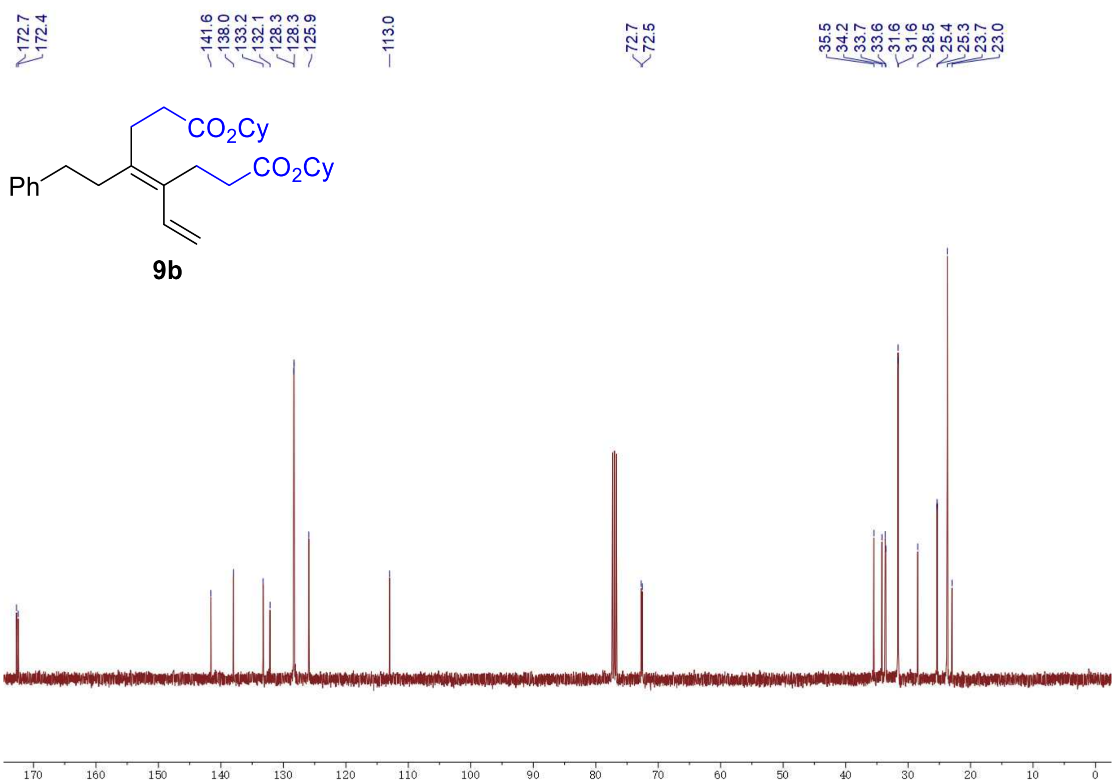

Supplementary Figure 82. ^{1}H NMR and ^{13}C NMR spectrum of compound of **10a**

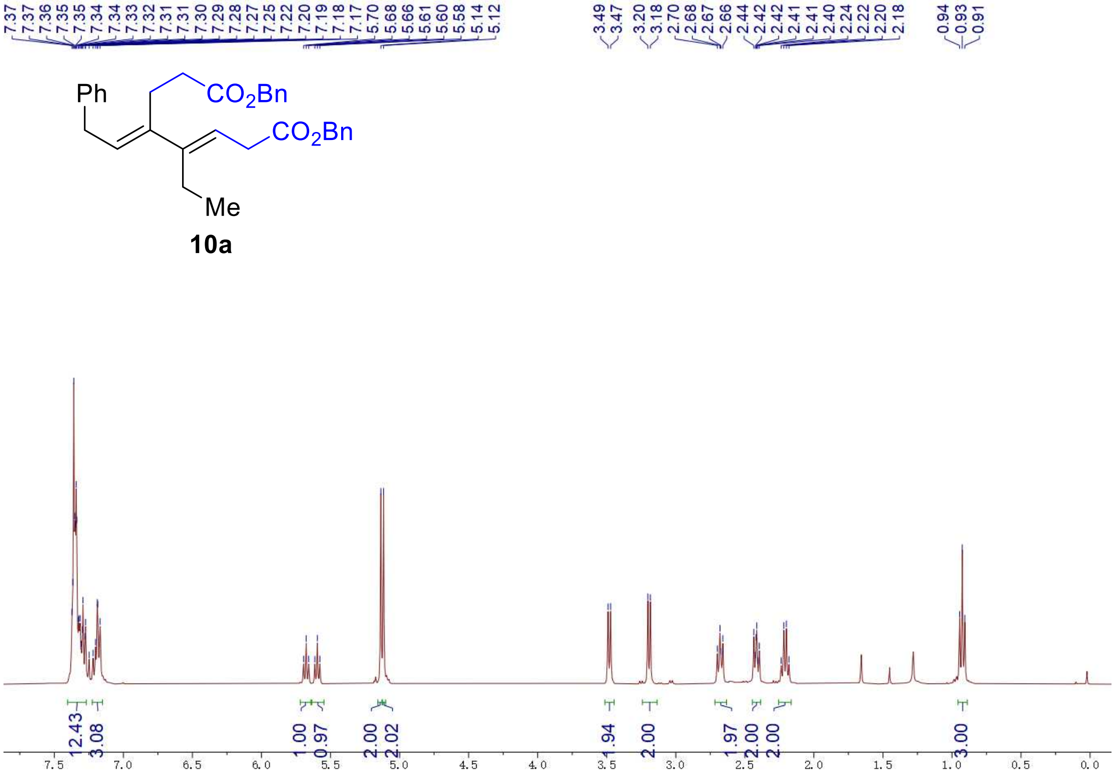

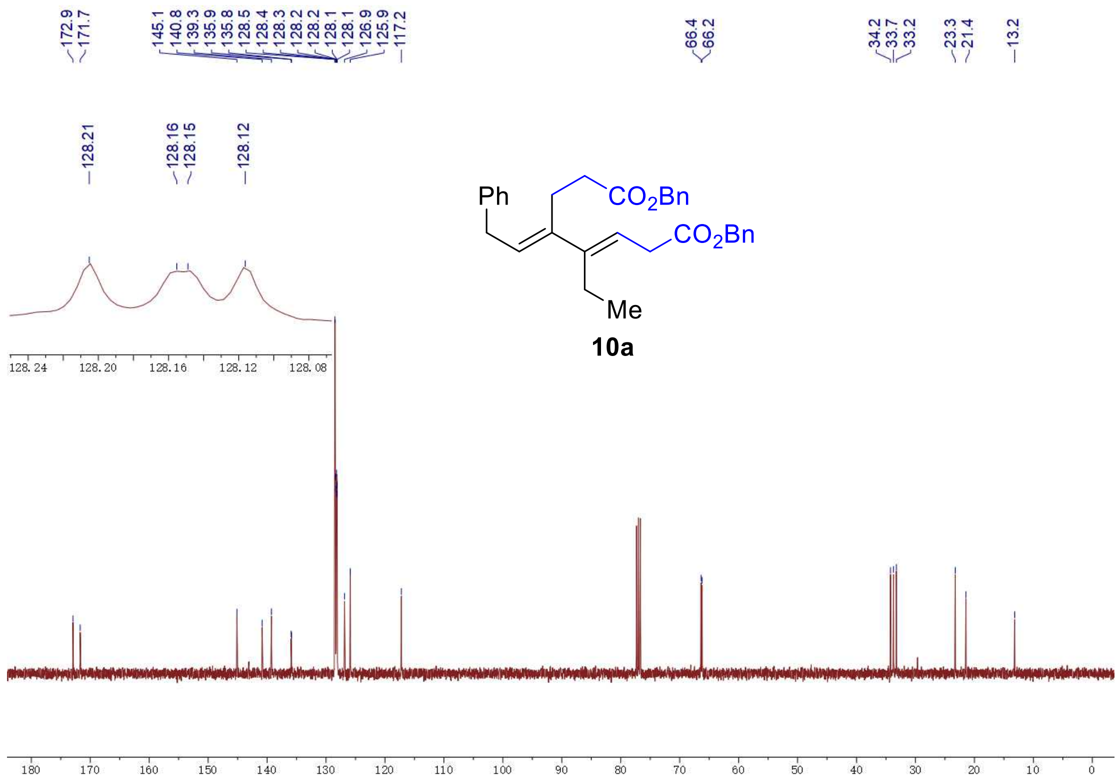

Supplementary Figure 83. ^{1}H NMR and ^{13}C NMR spectrum of compound of **10b**

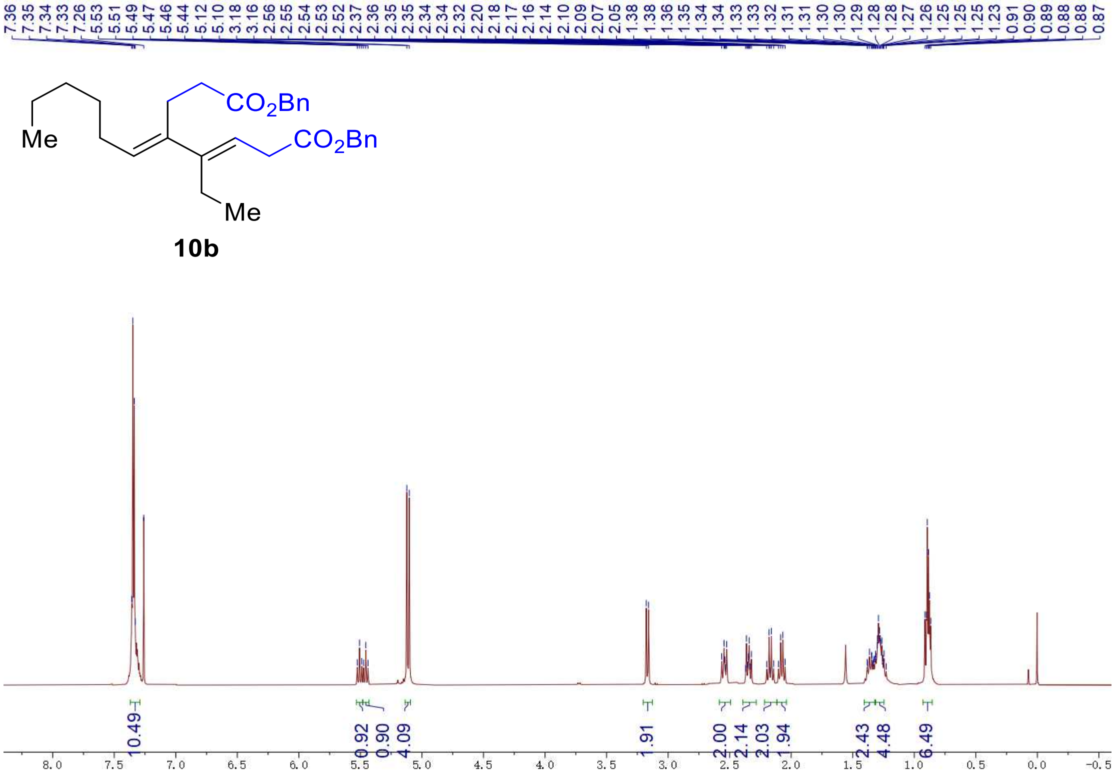

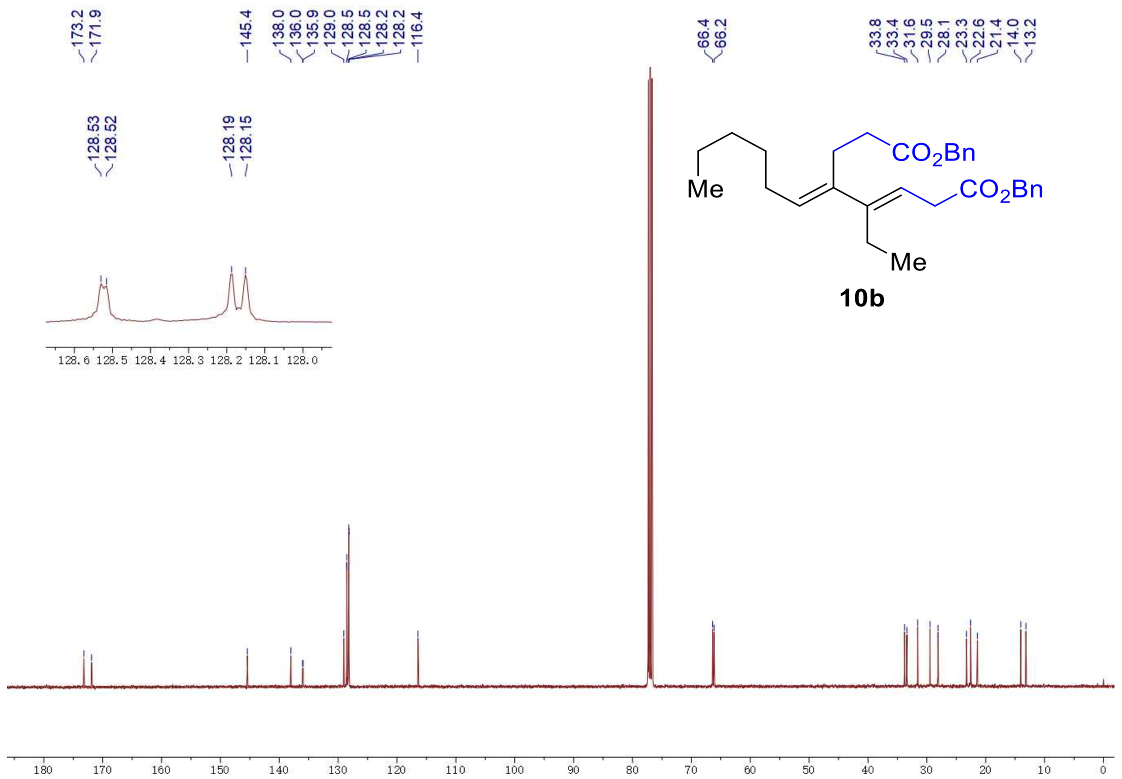

Supplementary Figure 84. ^{1}H NMR and ^{13}C NMR spectrum of compound of **10c**

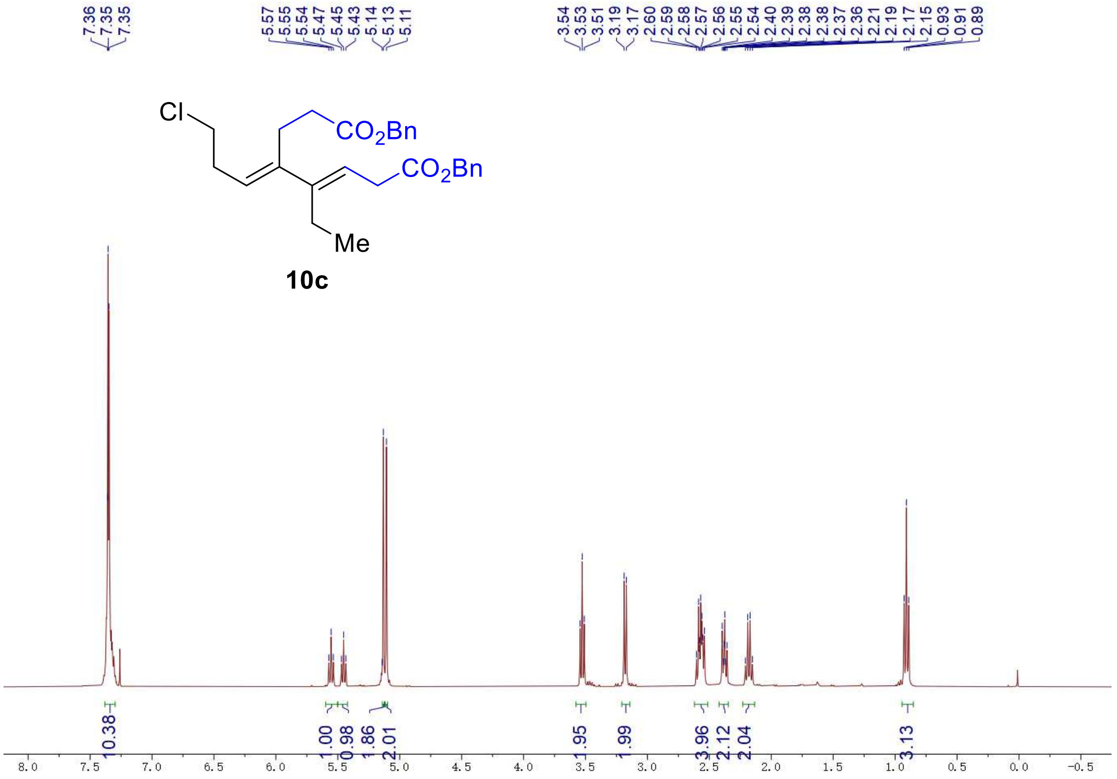

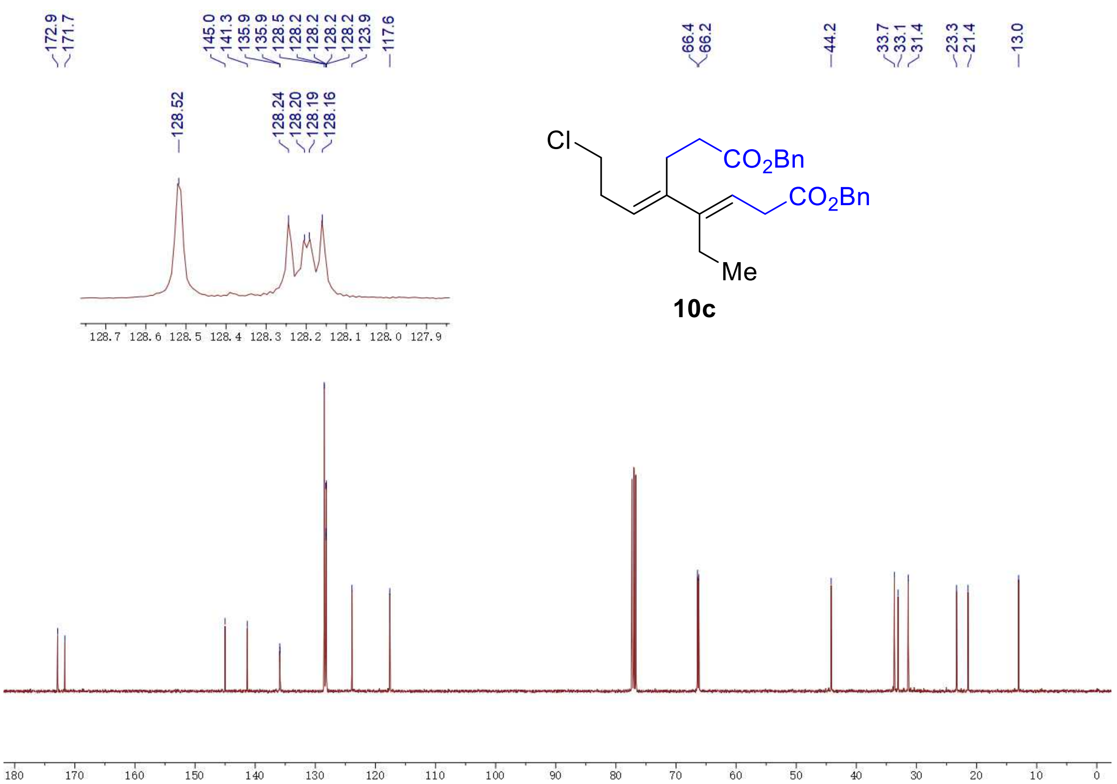

10c

Supplementary Figure 85. ^{1}H NMR and ^{13}C NMR spectrum of compound of **10d**

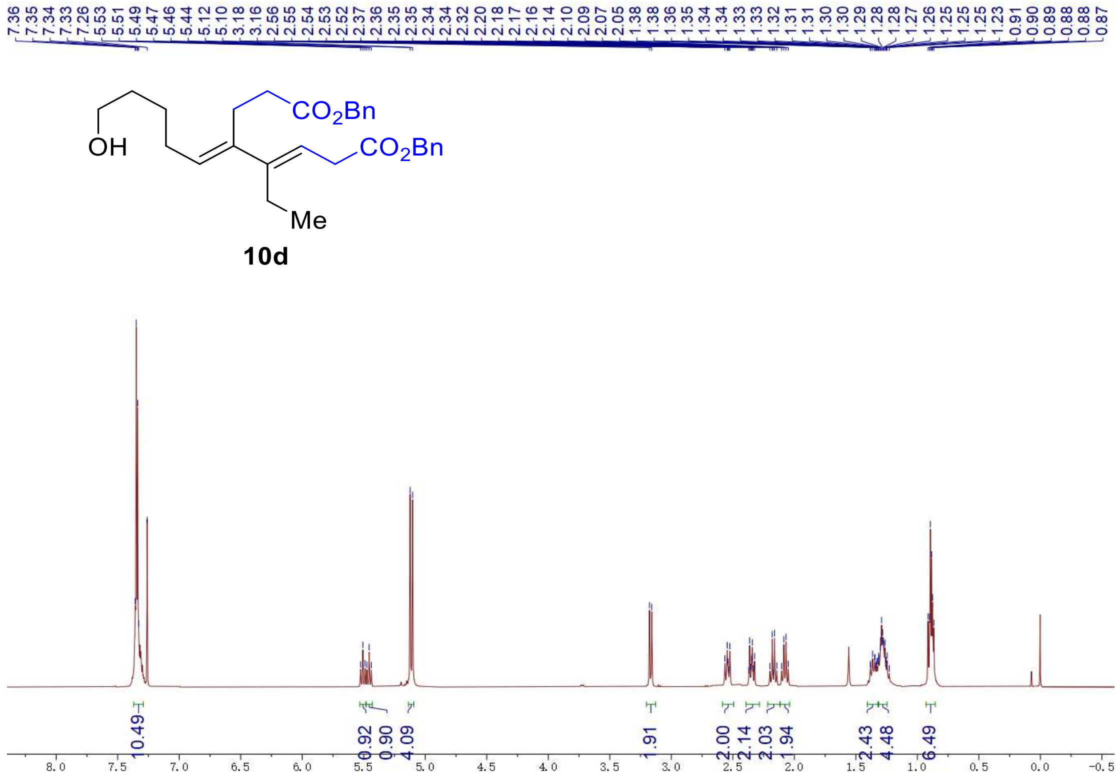

10d

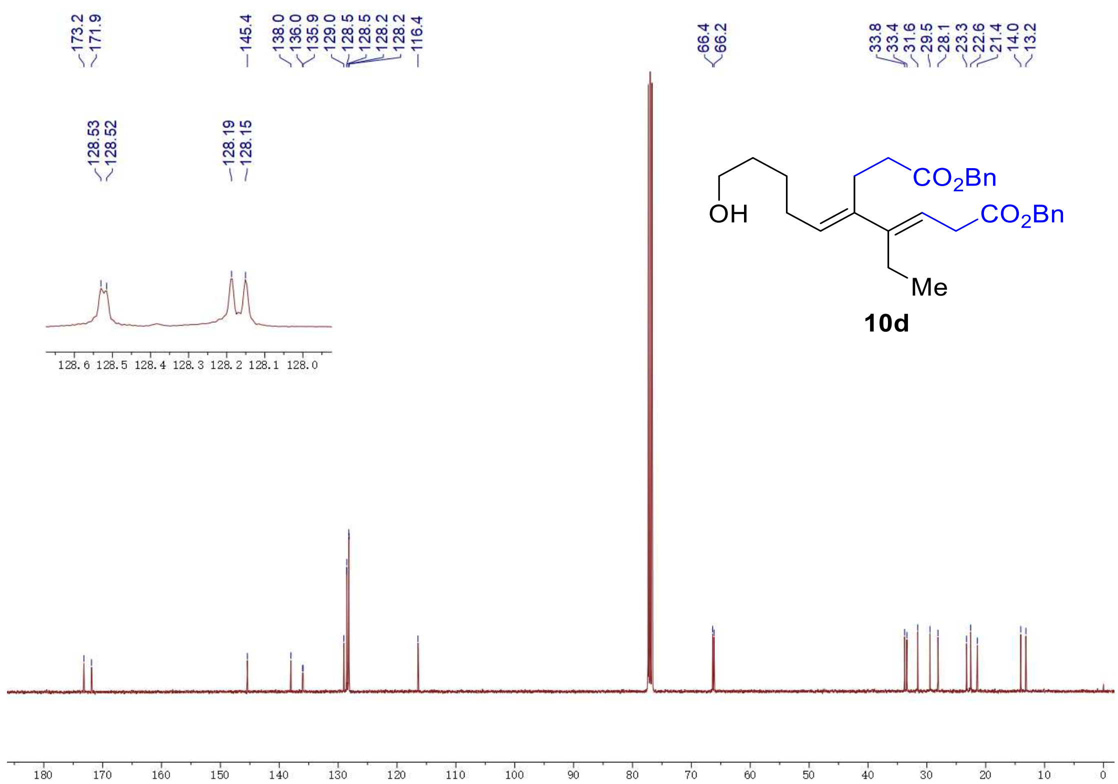

Supplementary Figure 86. ^{1}H NMR and ^{13}C NMR spectrum of compound of **10e**

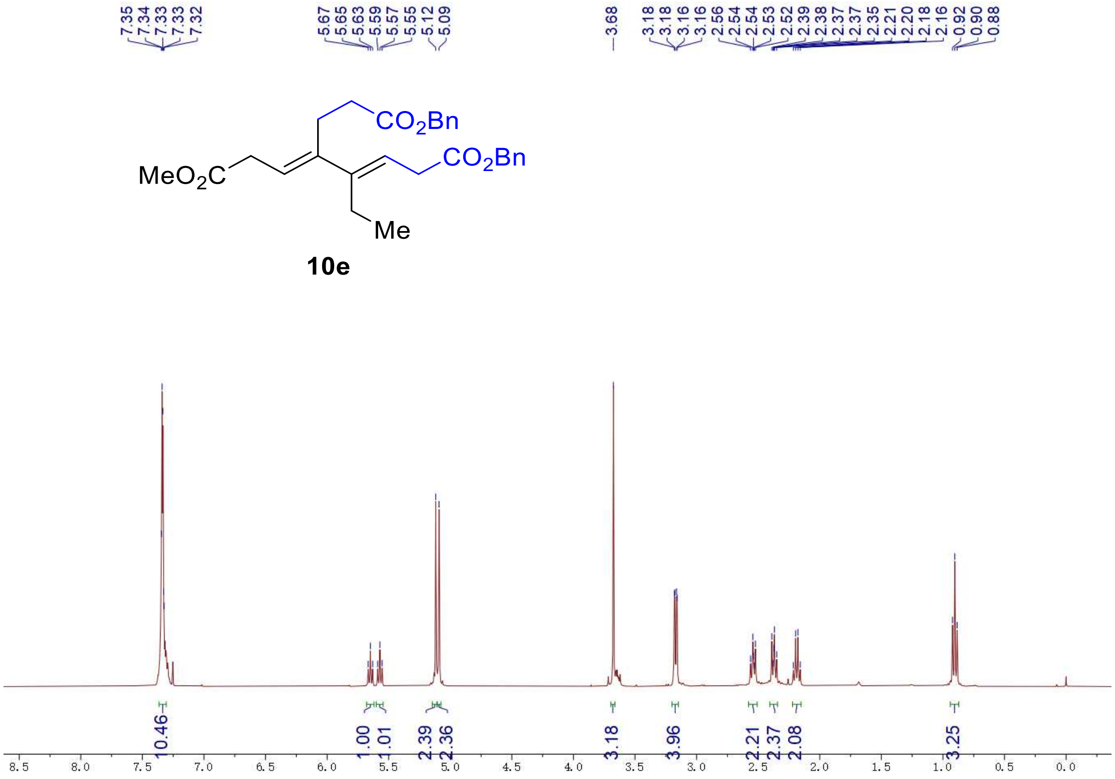

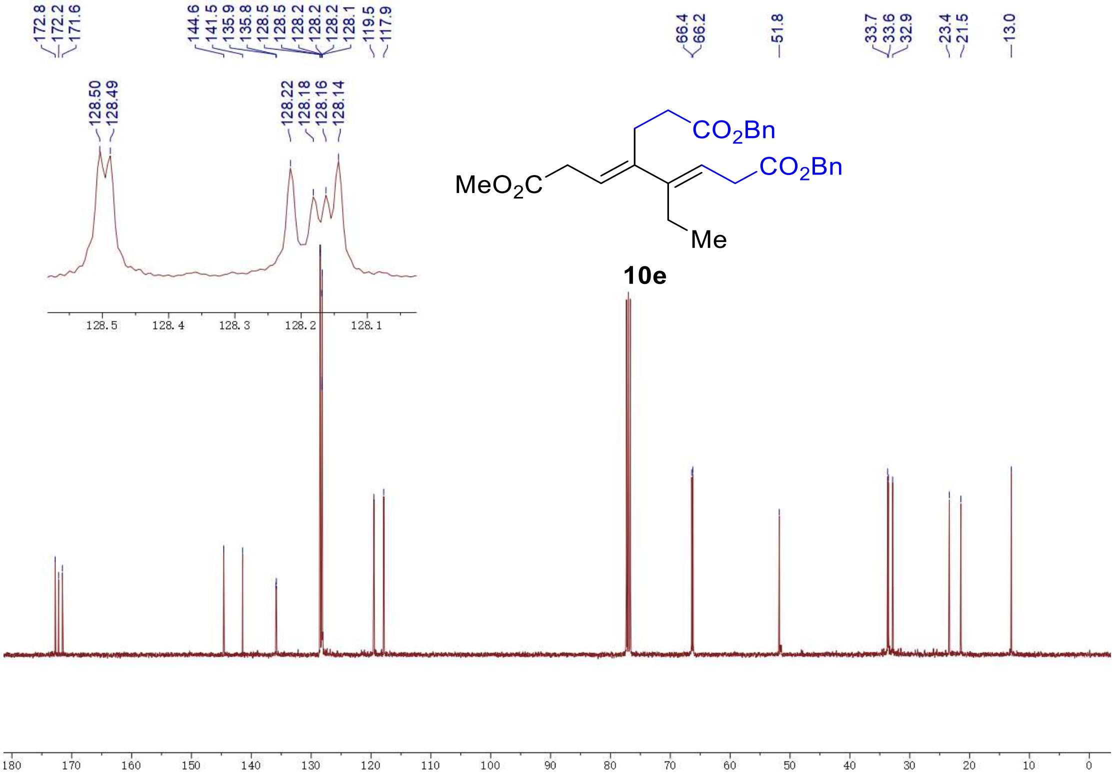

Supplementary Figure 87. ^{1}H NMR and ^{13}C NMR spectrum of compound of **10f**

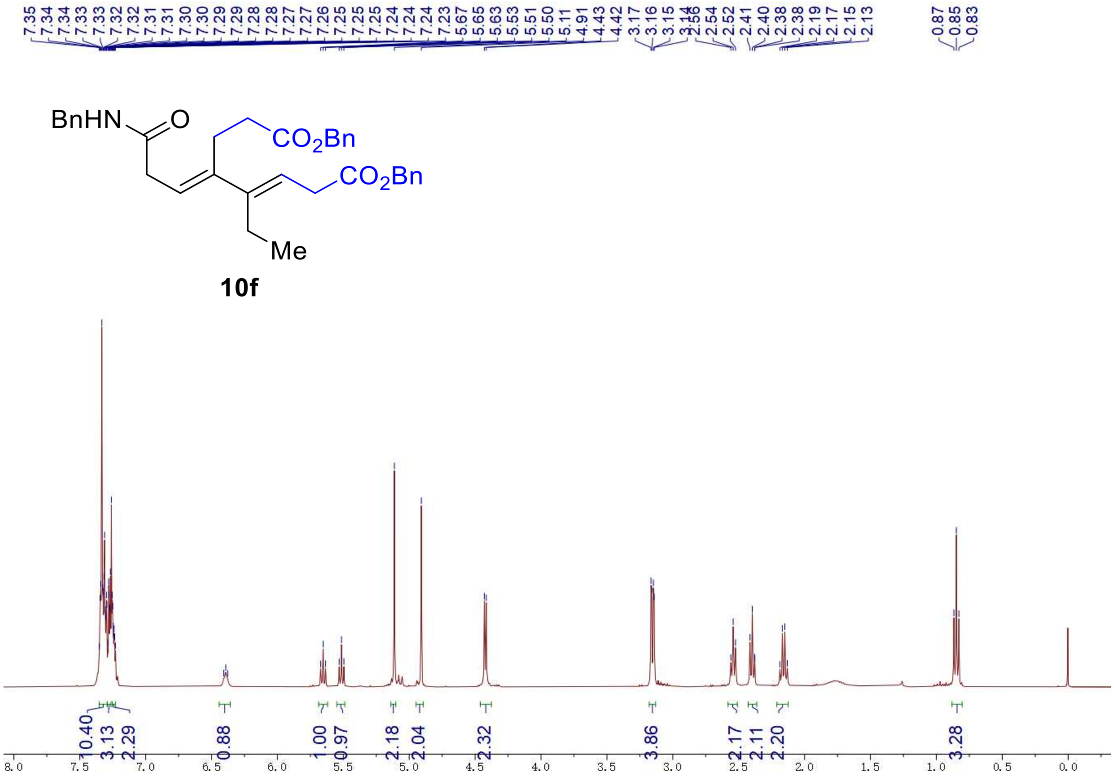

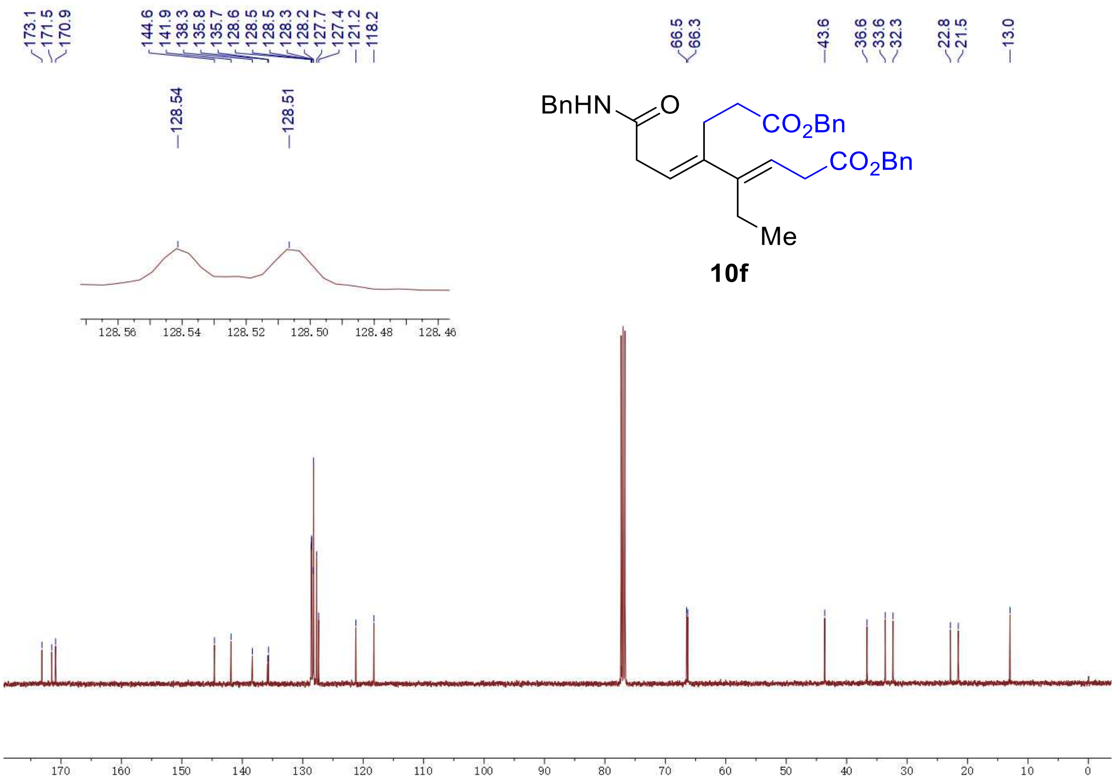

Supplementary Figure 88. ^{1}H NMR and ^{13}C NMR spectrum of compound of **10g**

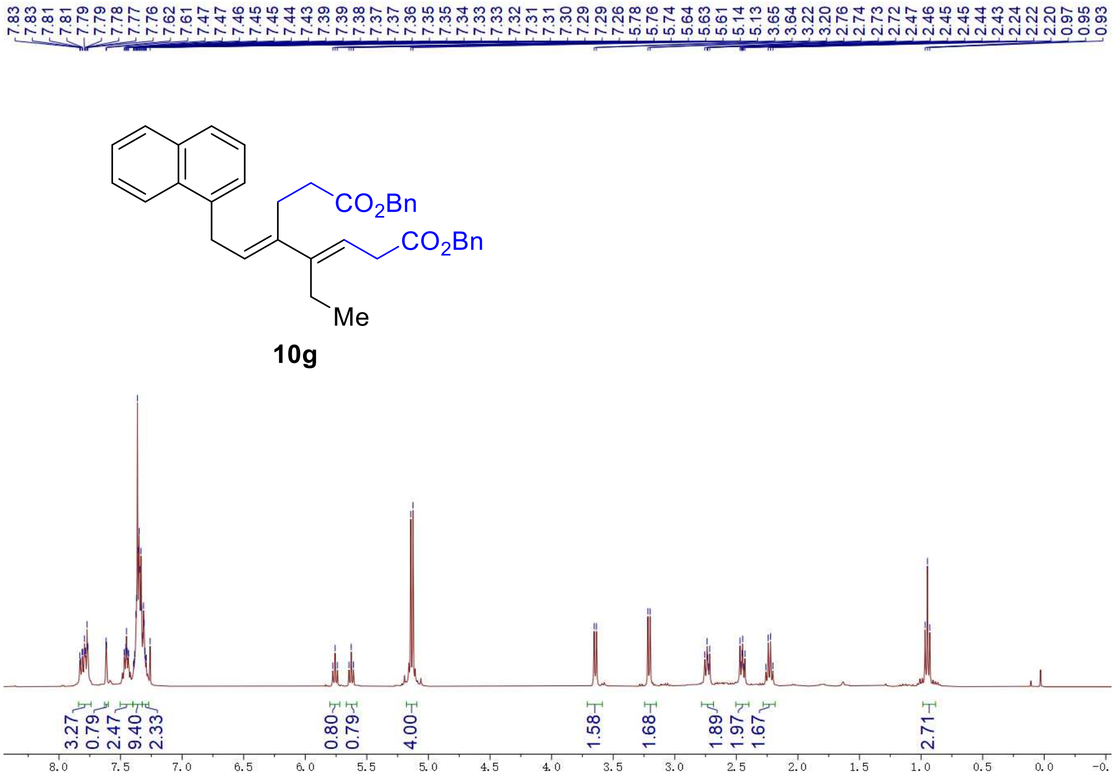

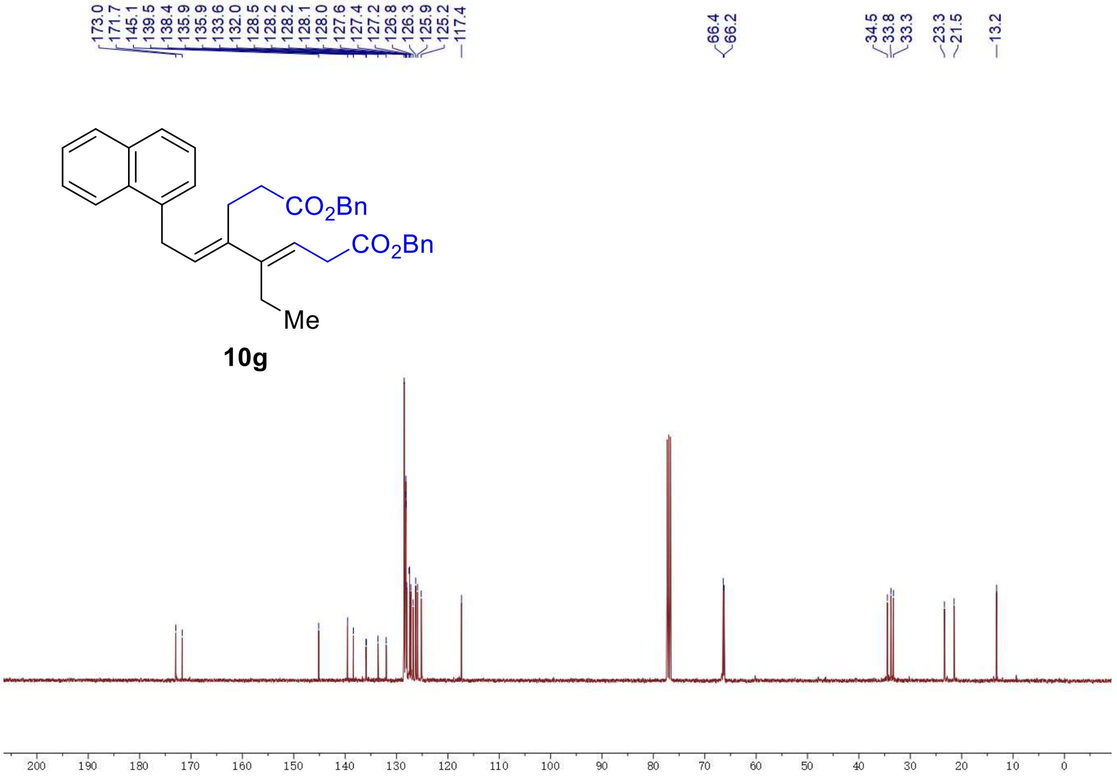

Supplementary Figure 89. ^{1}H NMR and ^{13}C NMR spectrum of compound of **10h**

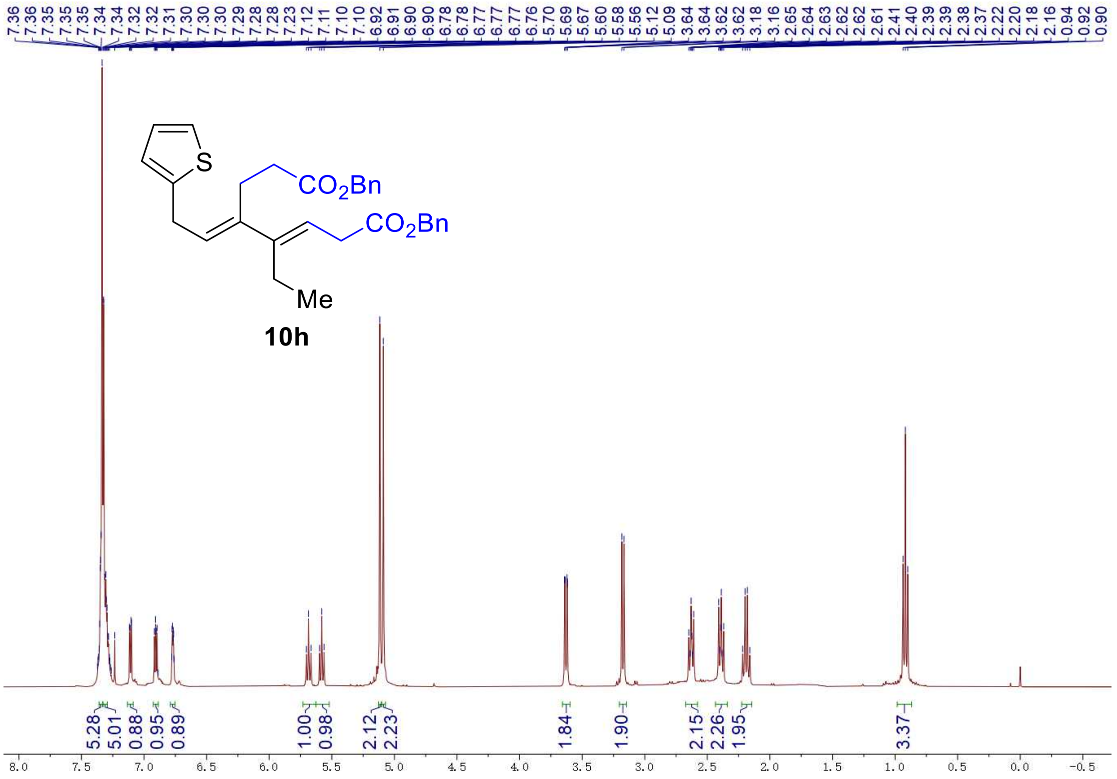

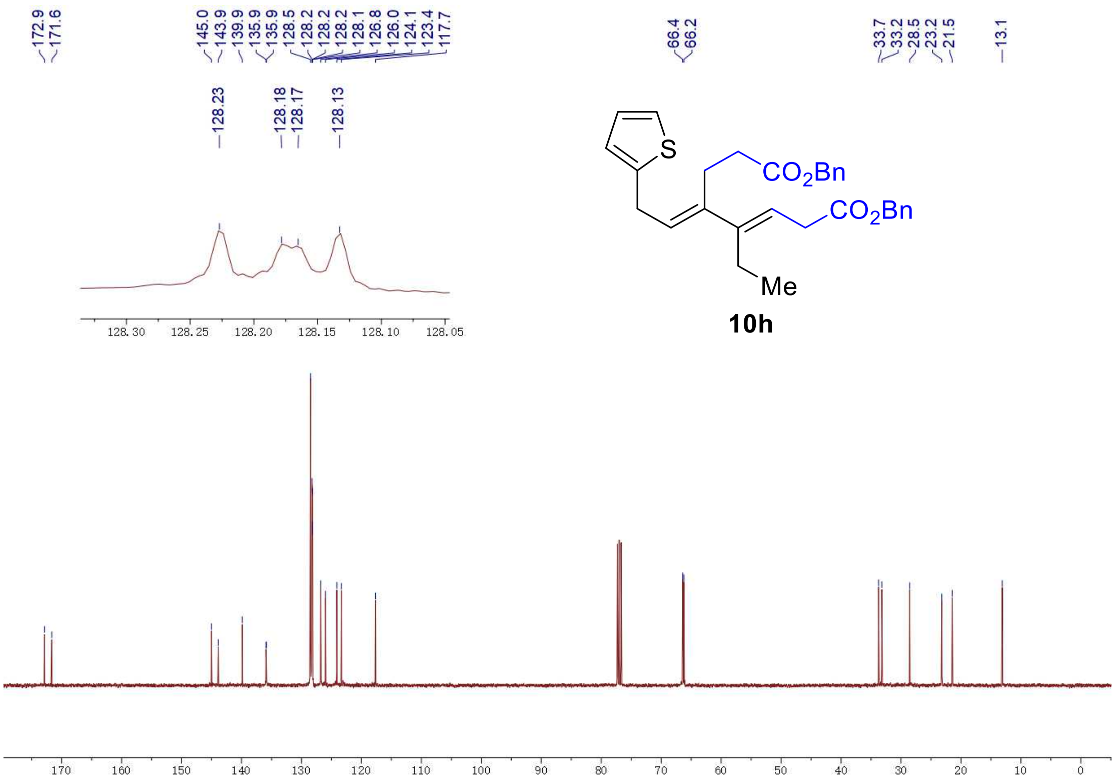
172.9
171.6
145.0
143.9
139.9
135.9
135.9
128.5
128.2
128.2
128.2
128.1
126.8
126.0
124.1
123.4
117.7
66.4
66.2
33.7
33.2
28.5
23.2
21.5
13.1
128.23
128.18
128.17
128.13
S
CO2Bn
CO2Bn
Me
10h

Supplementary Figure 90. ^{1}H NMR and ^{13}C NMR spectrum of compound of **10i**

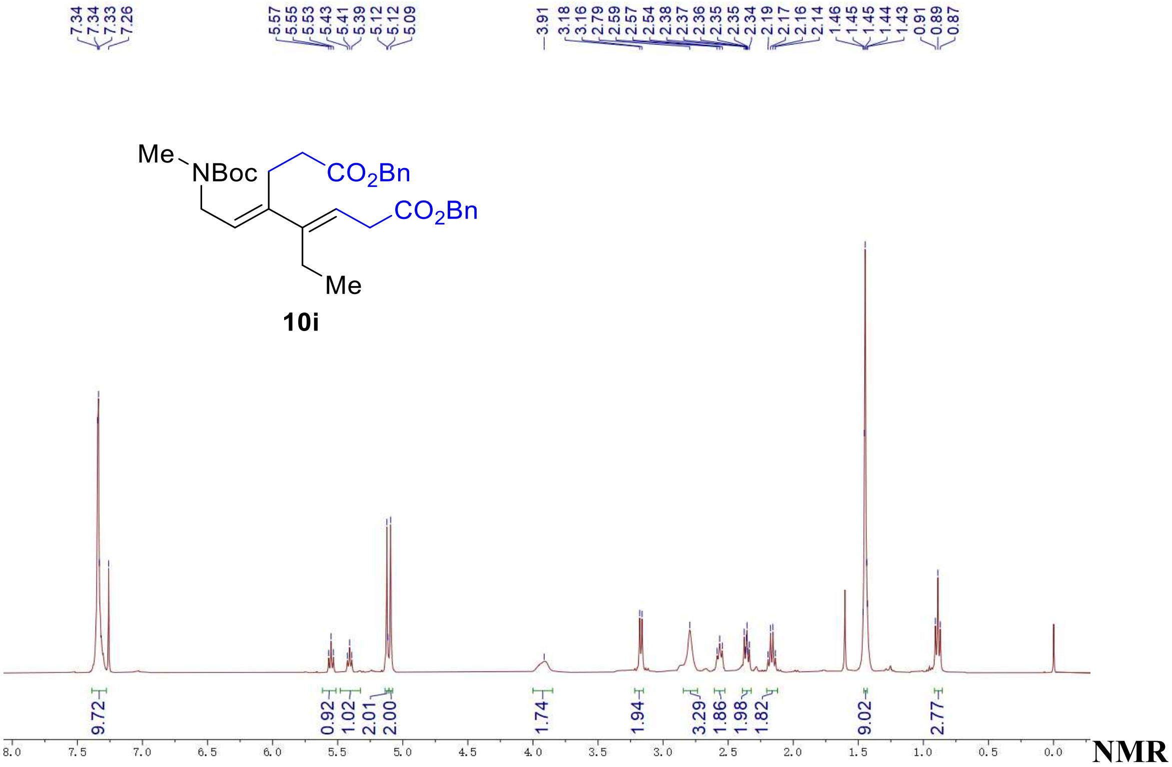

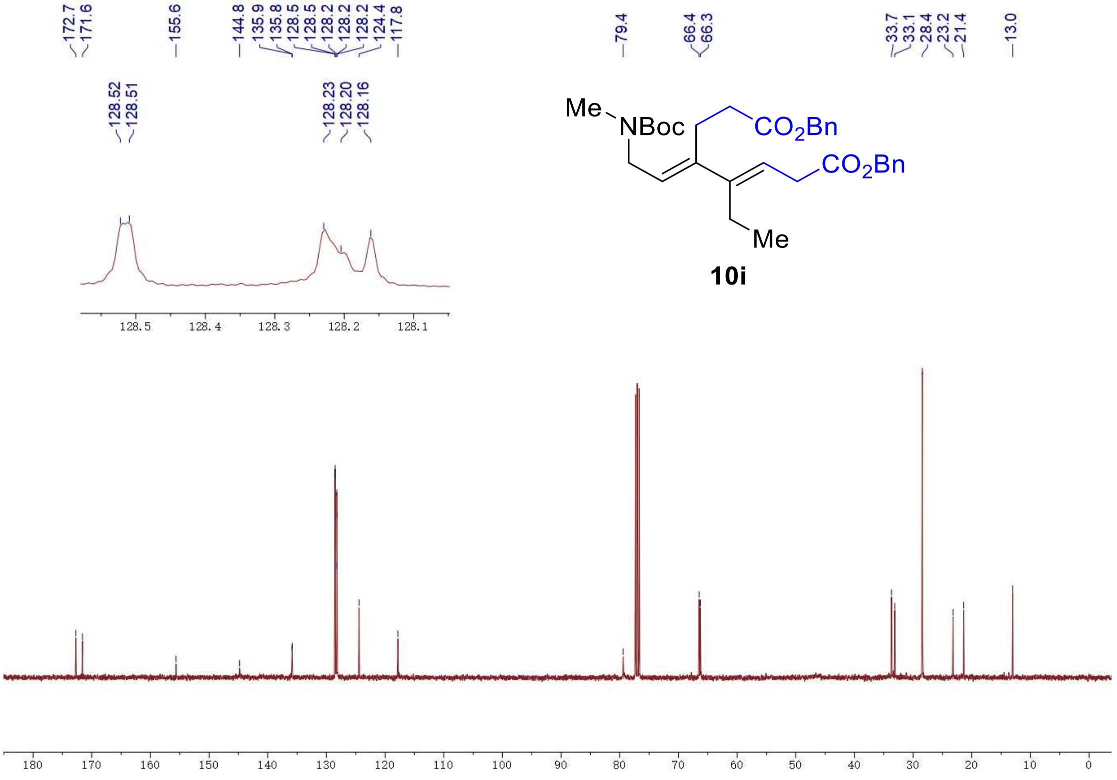

Supplementary Figure 91. ^{1}H NMR and ^{13}C NMR spectrum of compound of **10j**

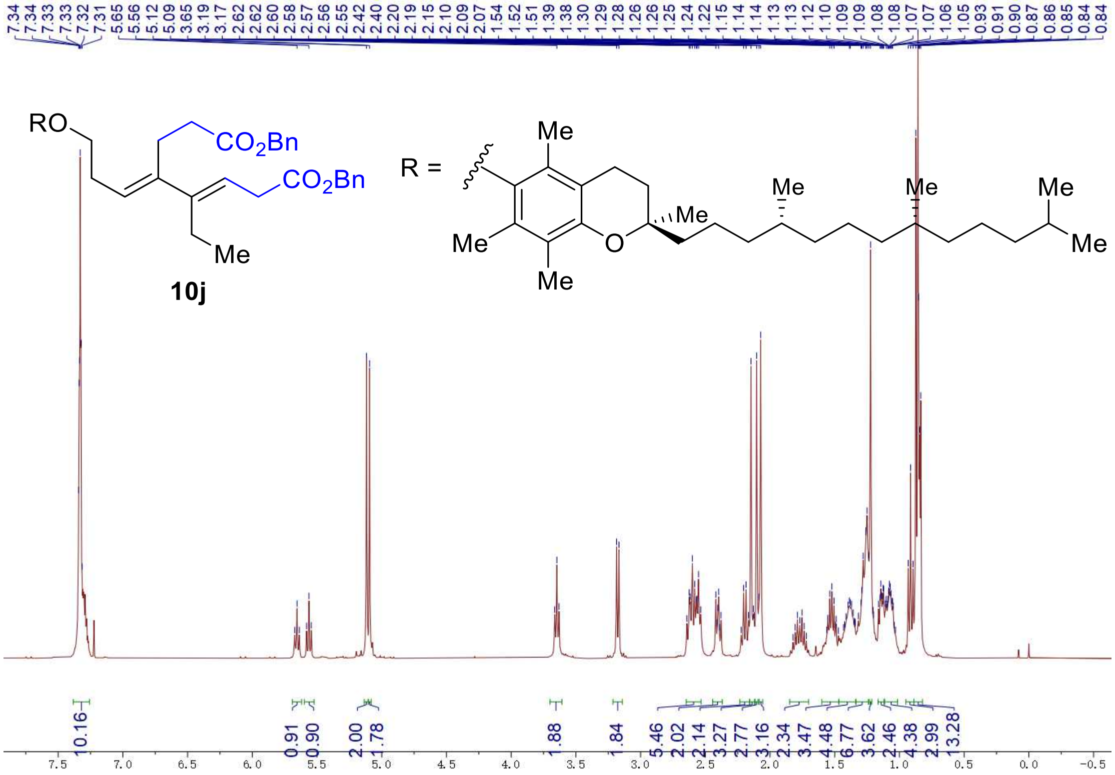

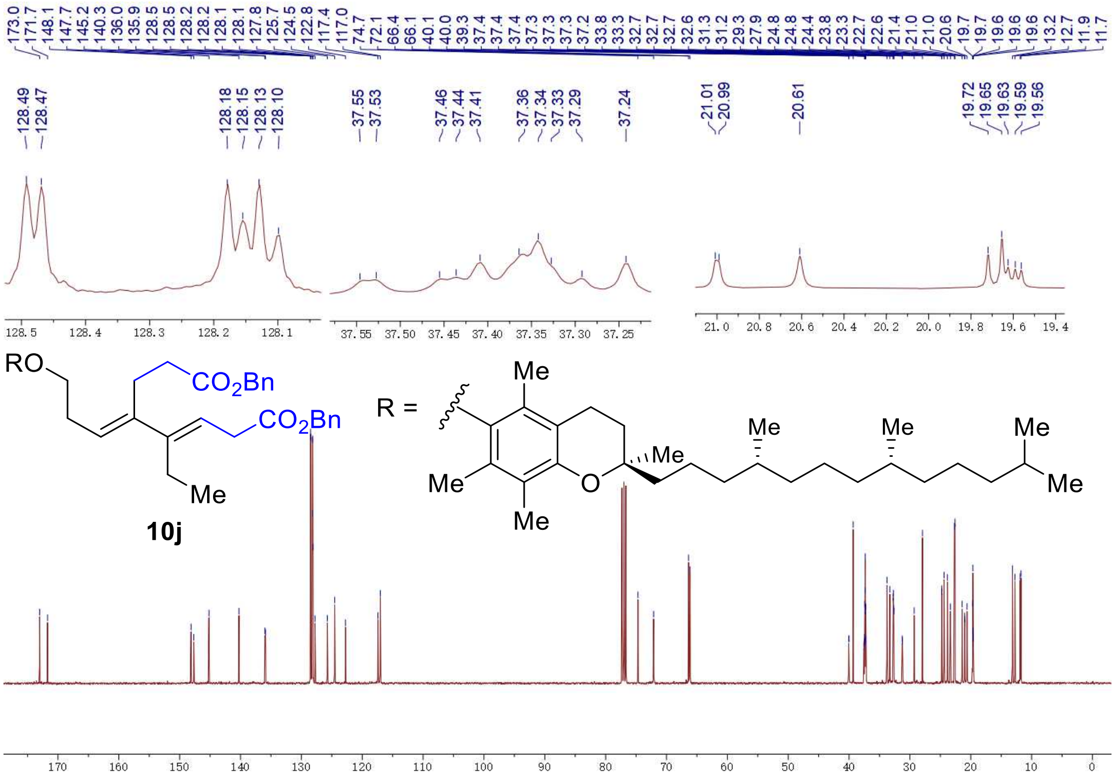

Supplementary Figure 92. ^{1}H NMR and ^{13}C NMR spectrum of compound of **10k**

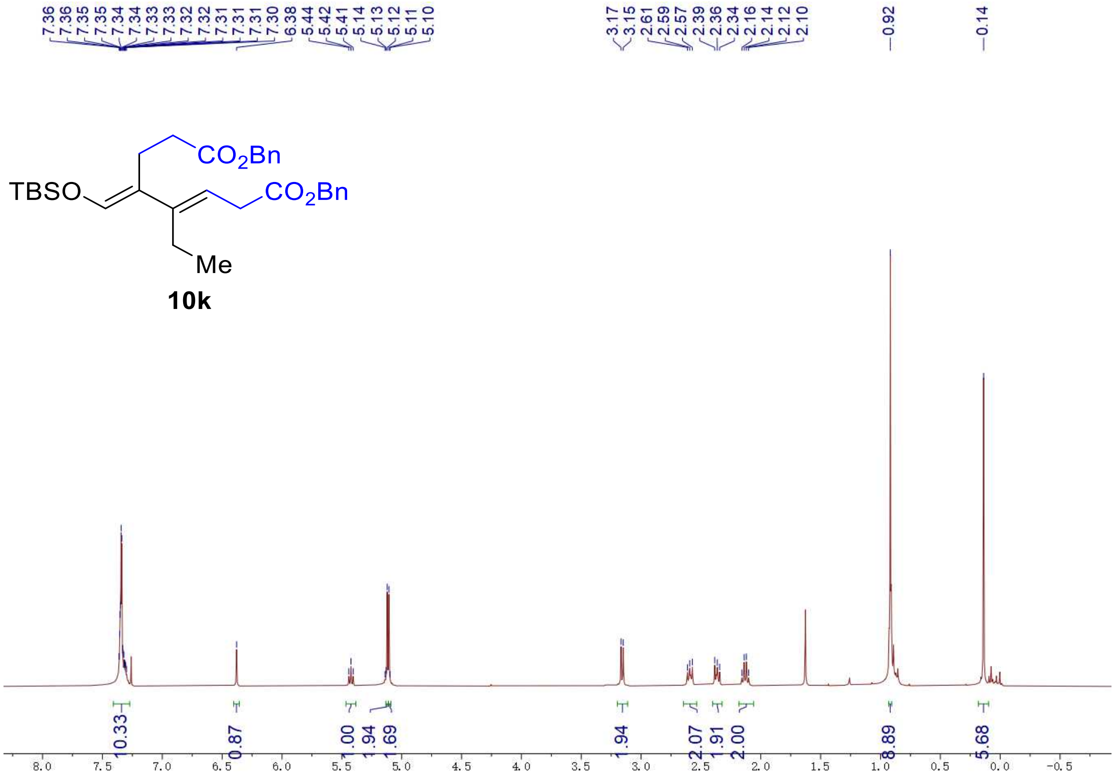

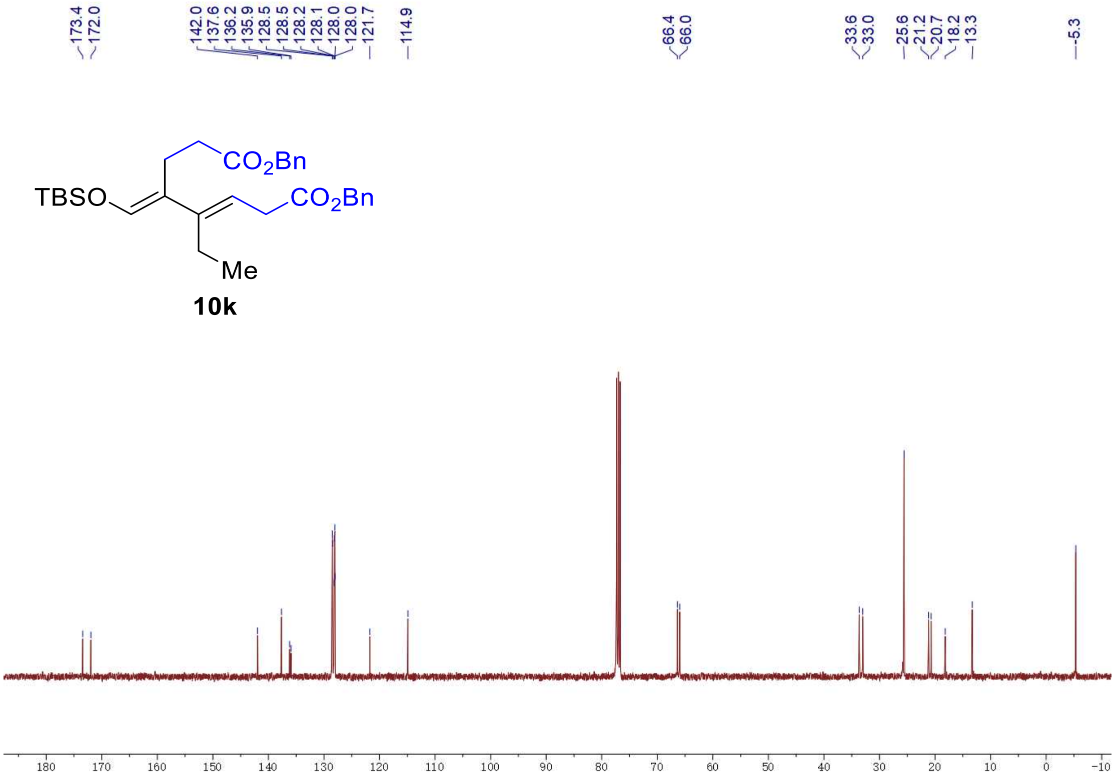

Supplementary Figure 93. ¹H NMR and ¹³C NMR spectrum of compound of **11a**

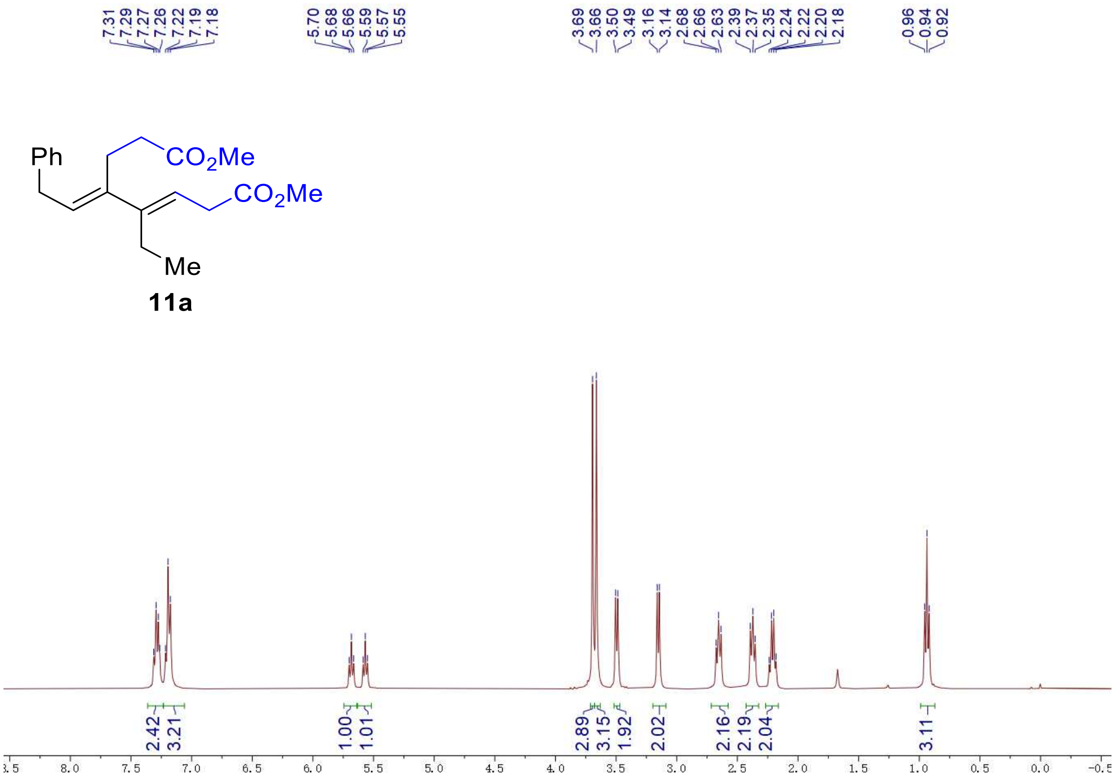

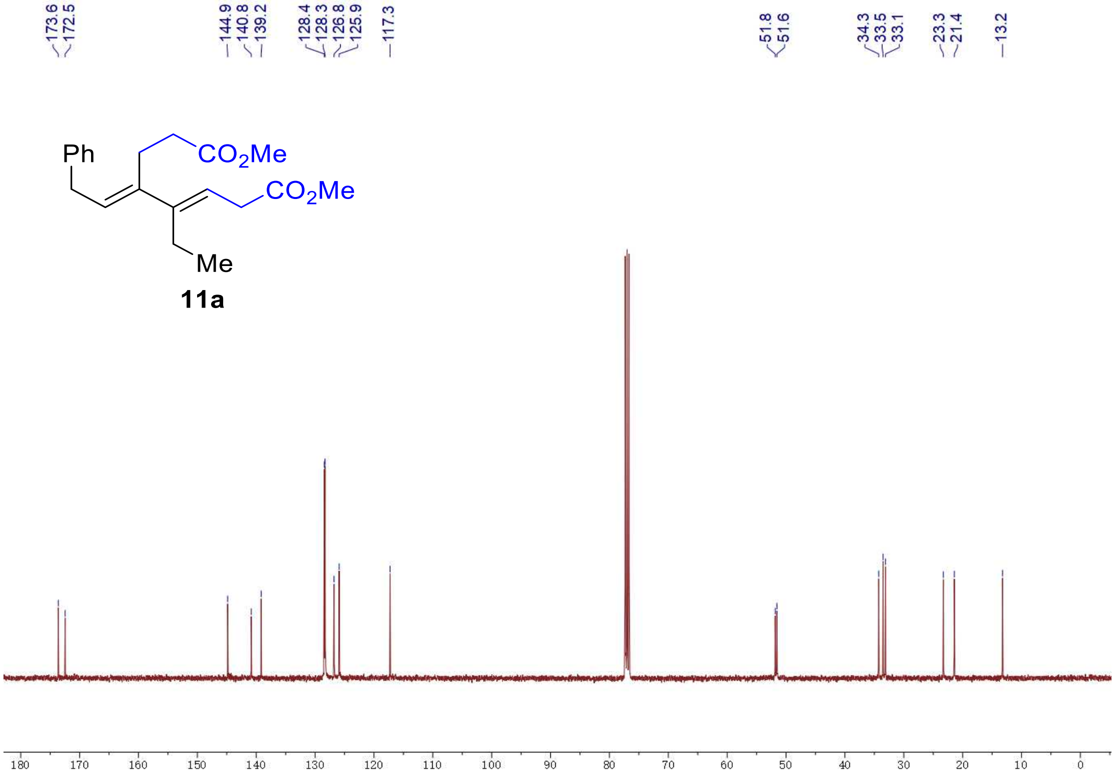

Supplementary Figure 94. ^{1}H NMR and ^{13}C NMR spectrum of compound of **11b**

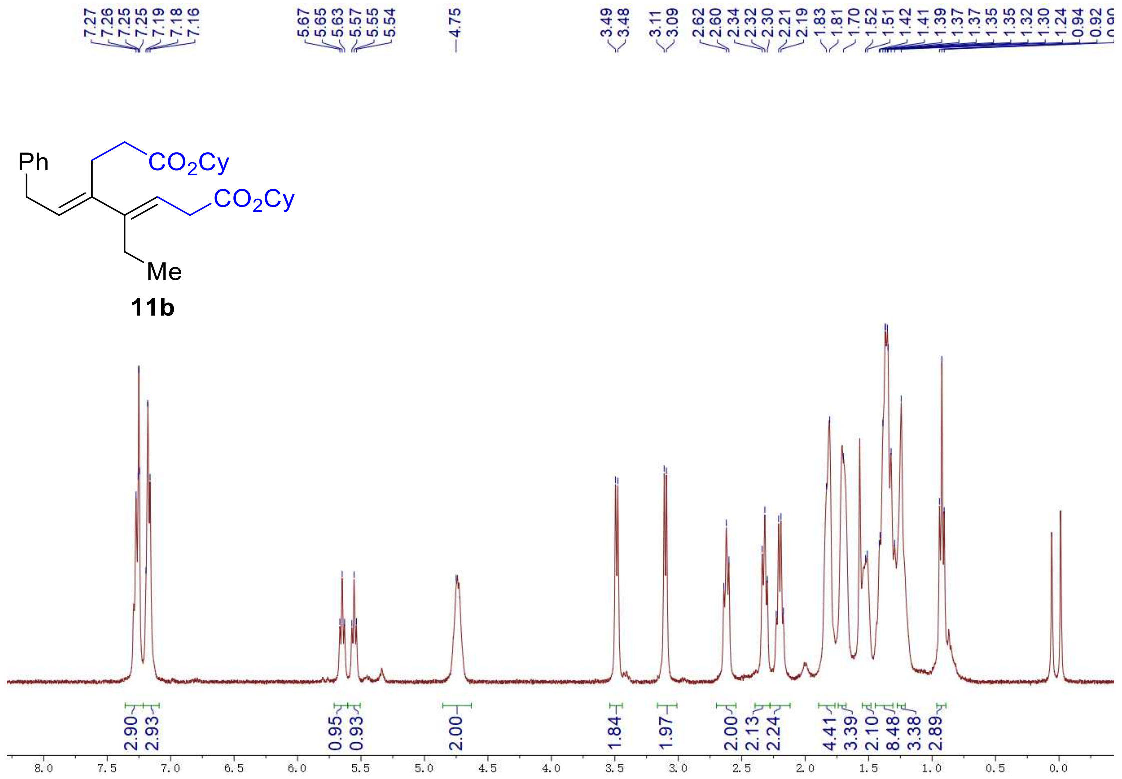

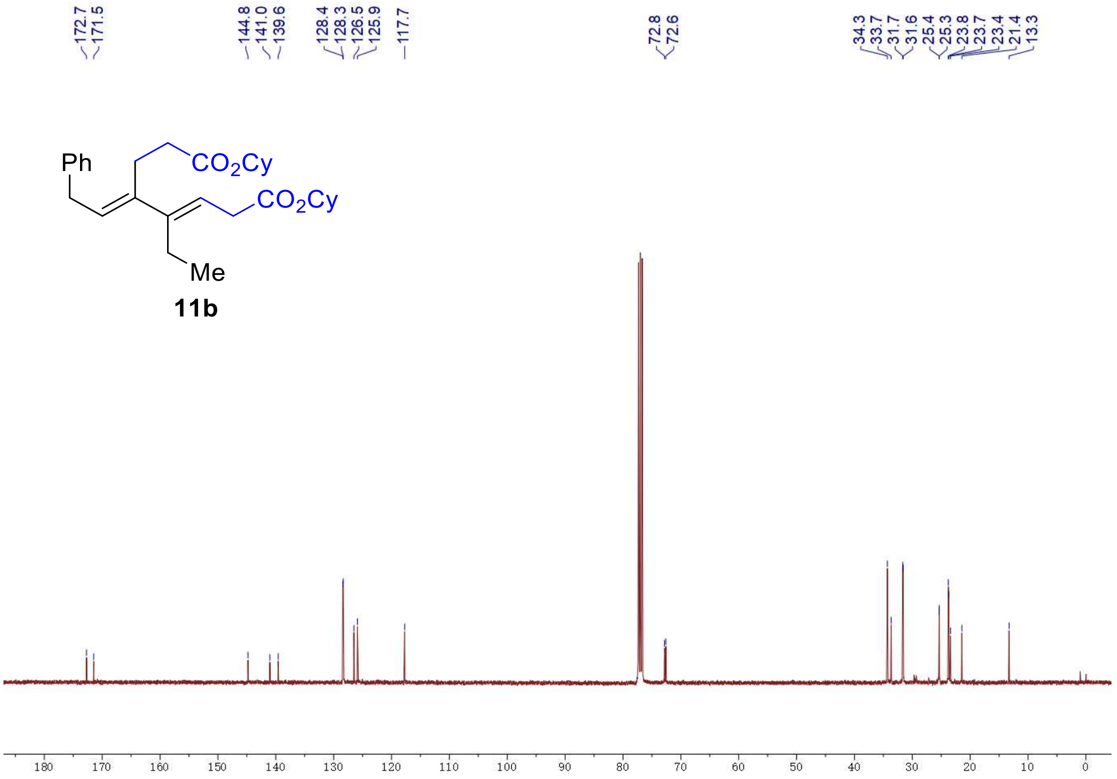

Supplementary Figure 95. ^{1}H NMR and ^{13}C NMR spectrum of compound of **11c**

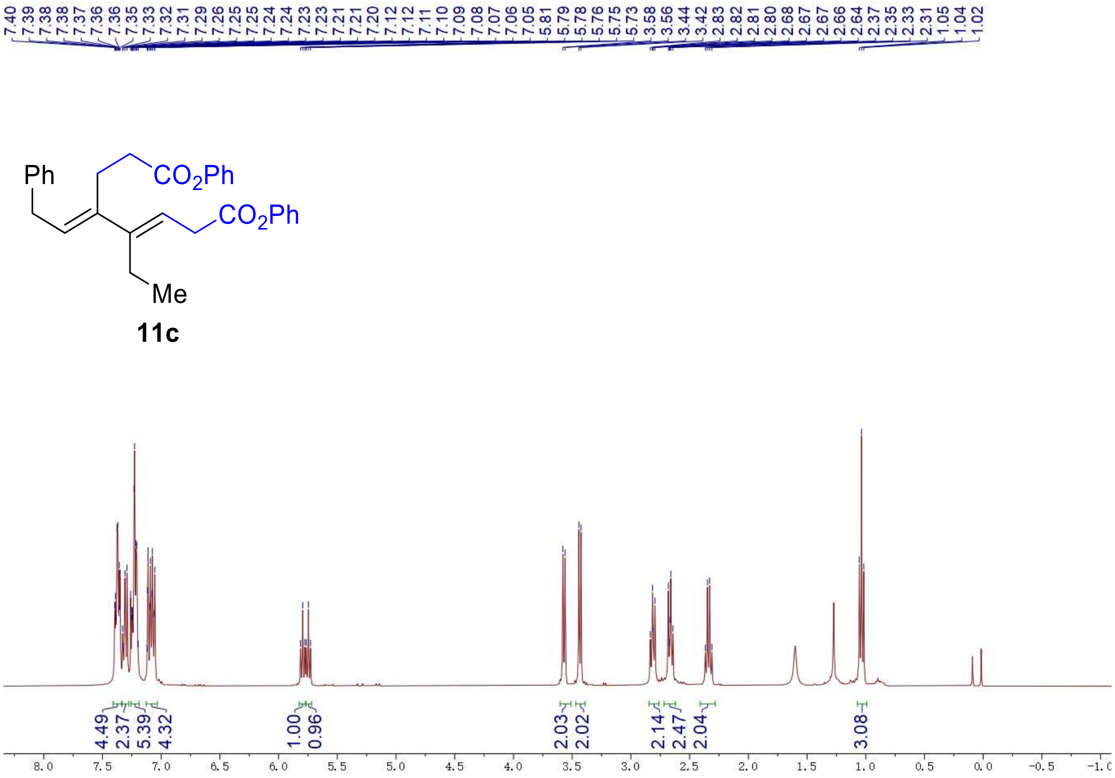

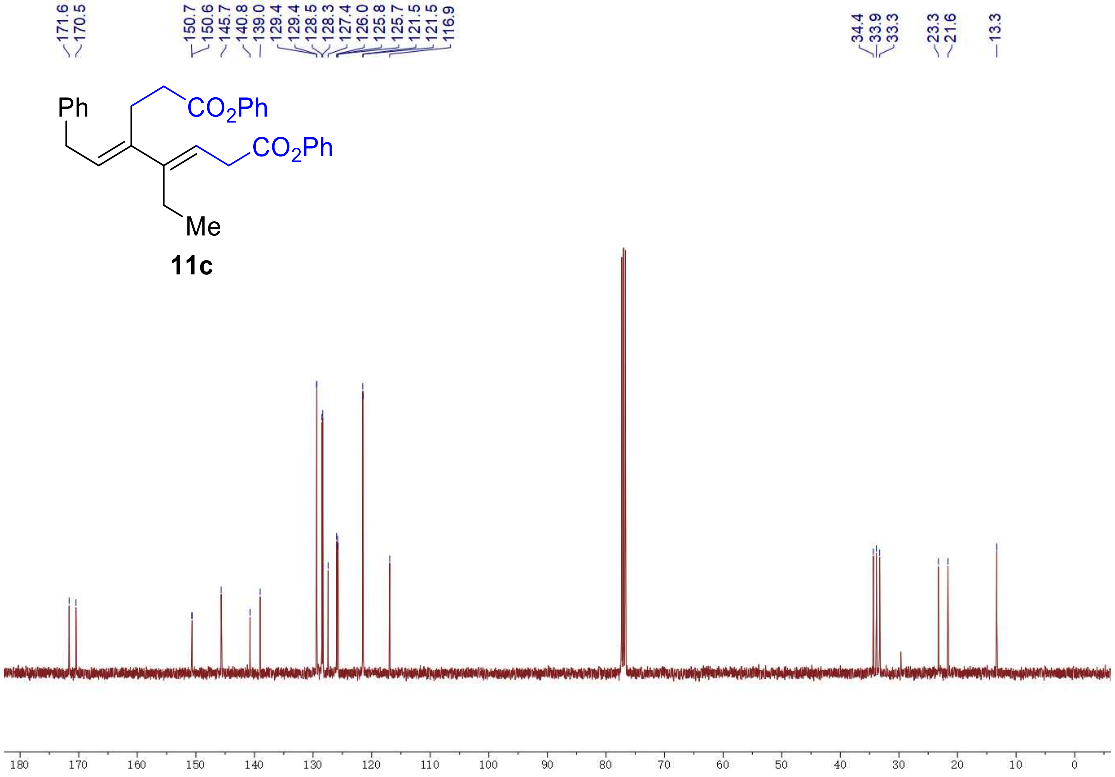

Supplementary Figure 96. ¹H NMR and ¹³C NMR spectrum of compound of **12**

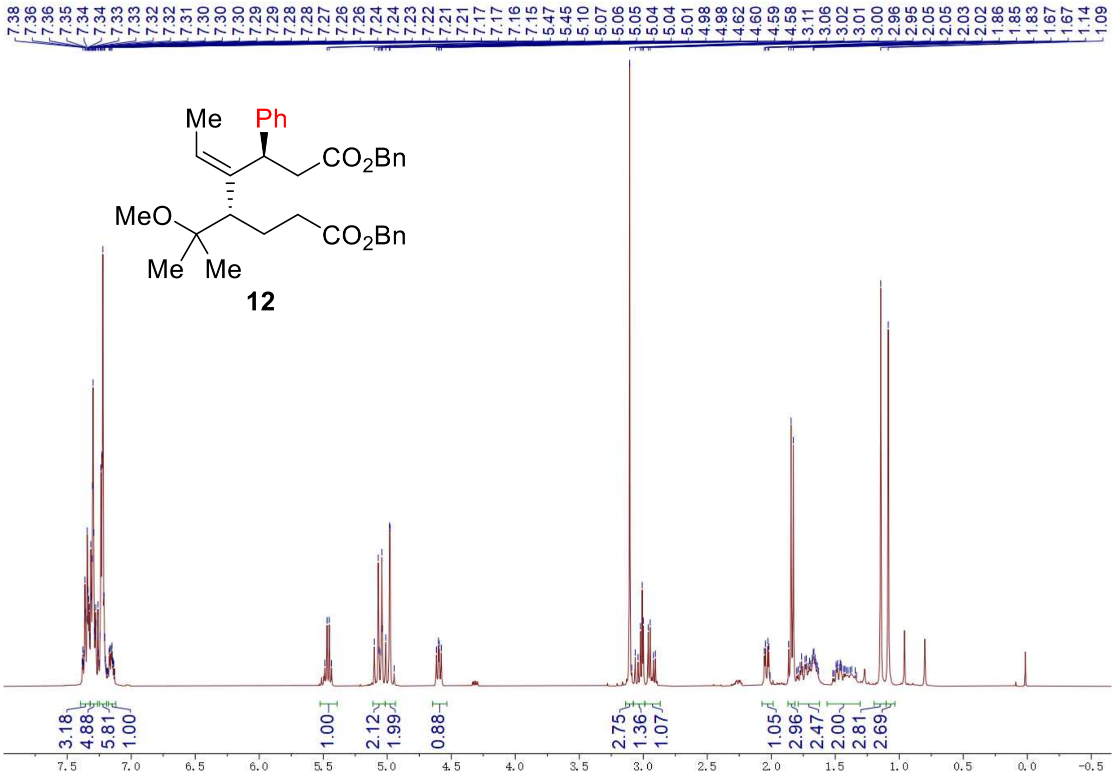

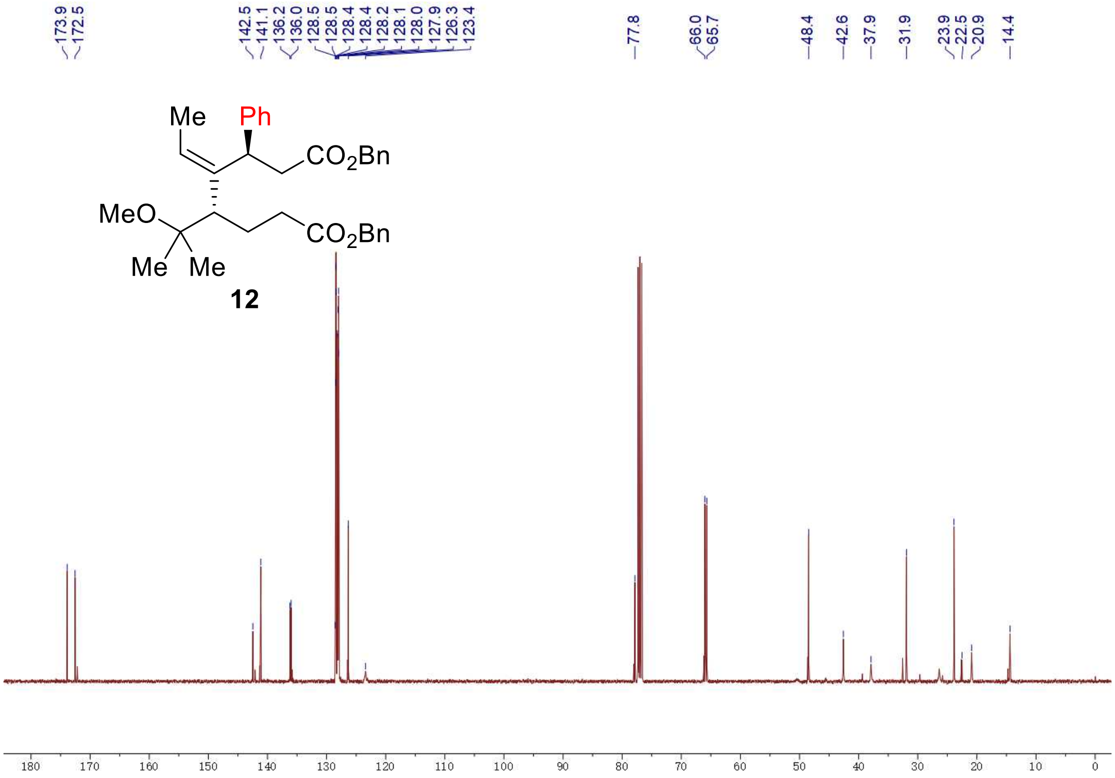

Supplementary Figure 97. ^{1}H NMR and ^{13}C NMR spectrum of compound of **13**

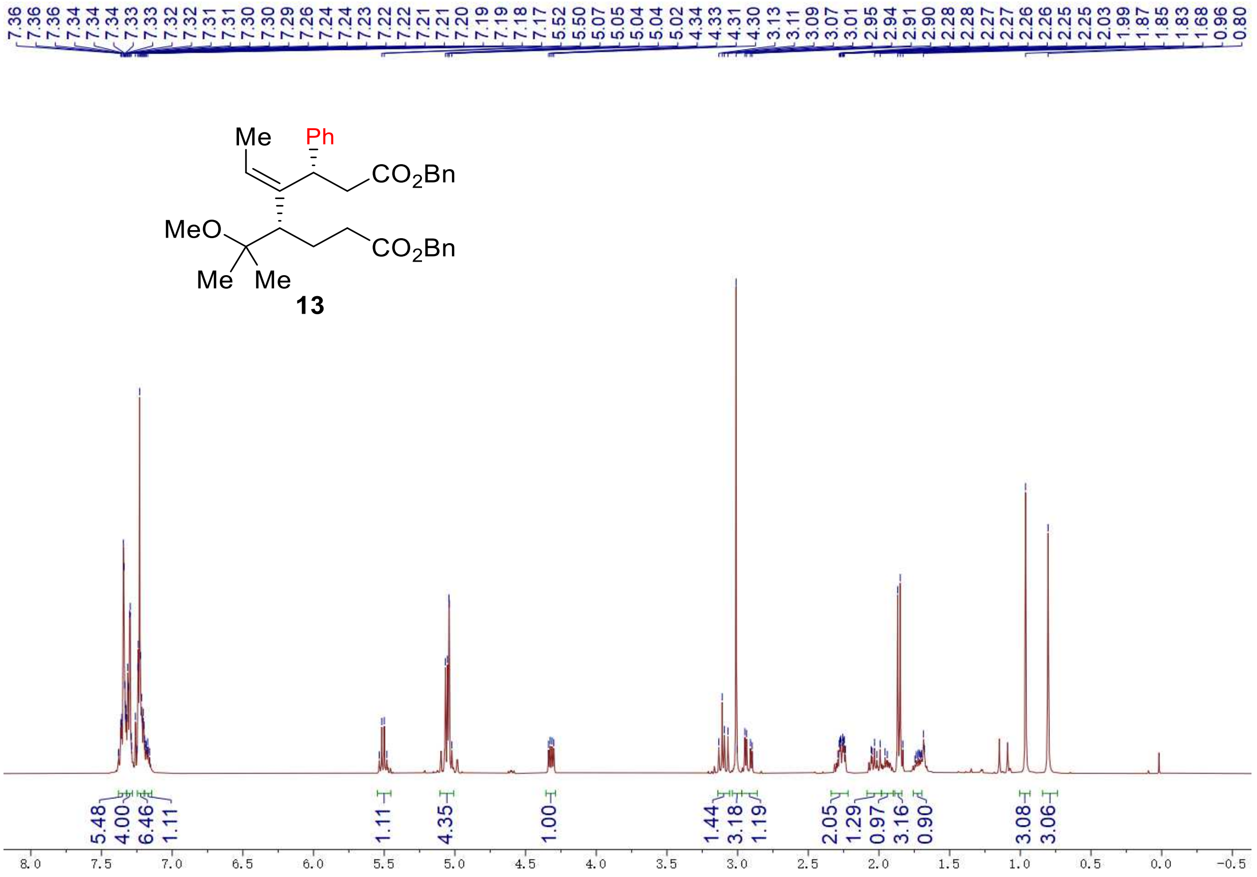

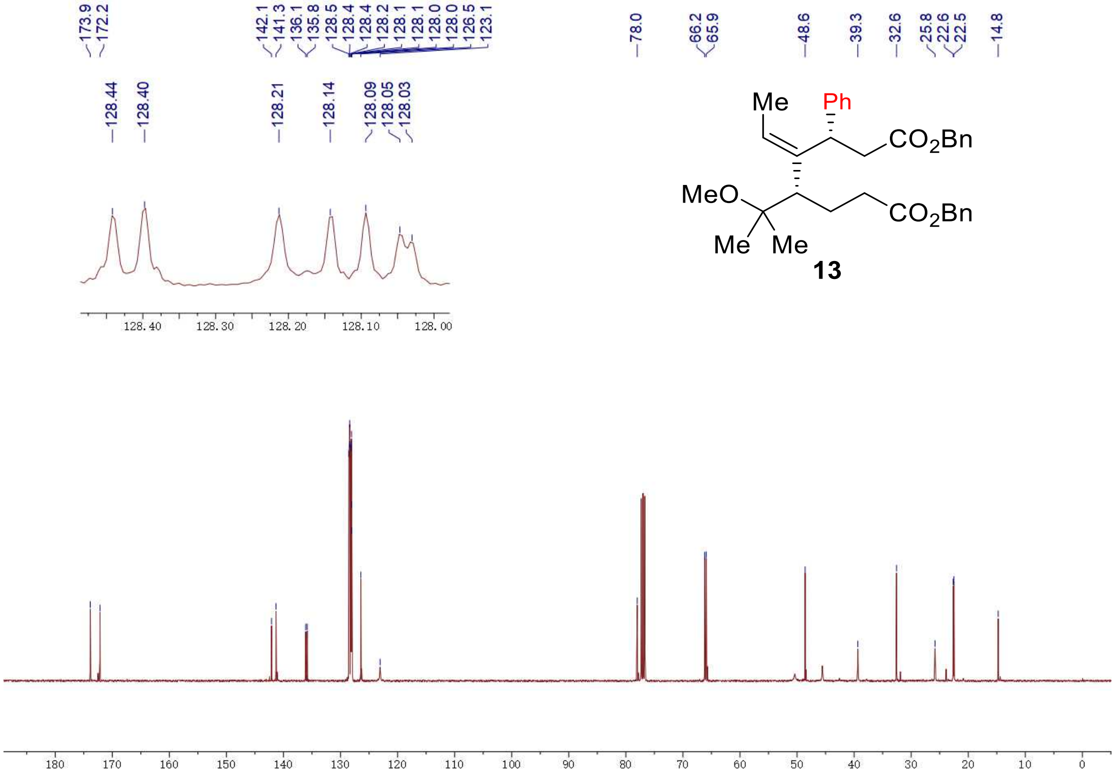

Supplementary Figure 98. ^{1}H NMR and ^{13}C NMR spectrum of compound of **15**

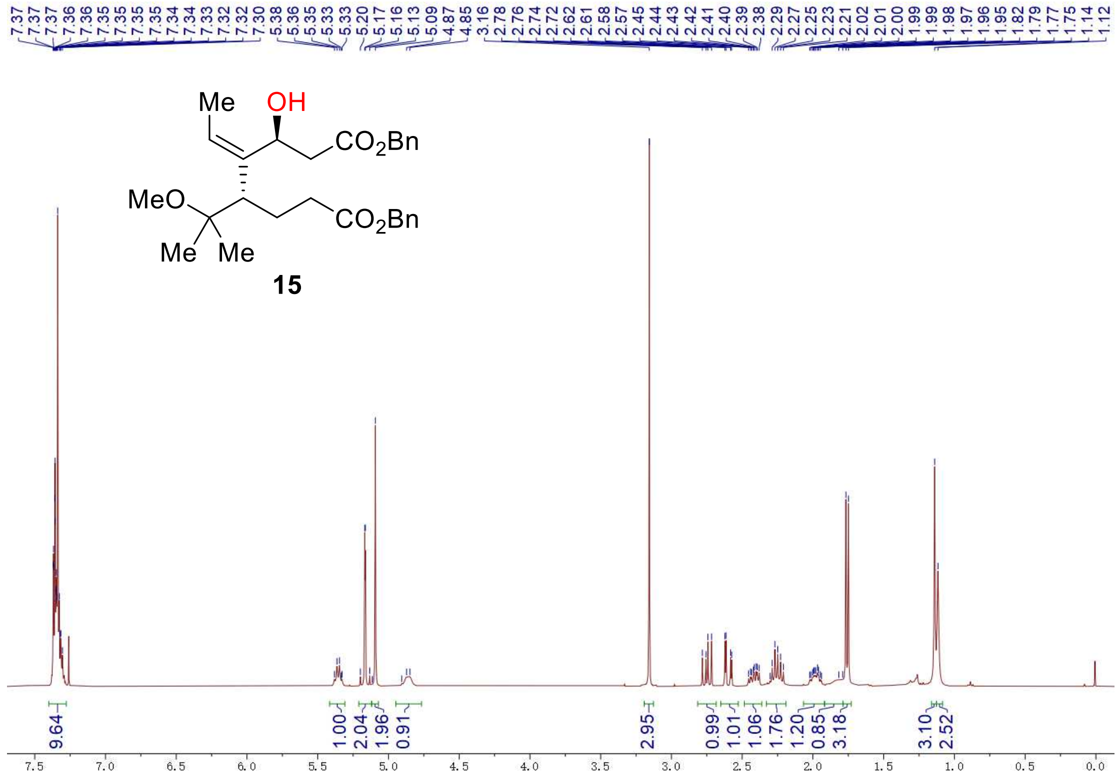

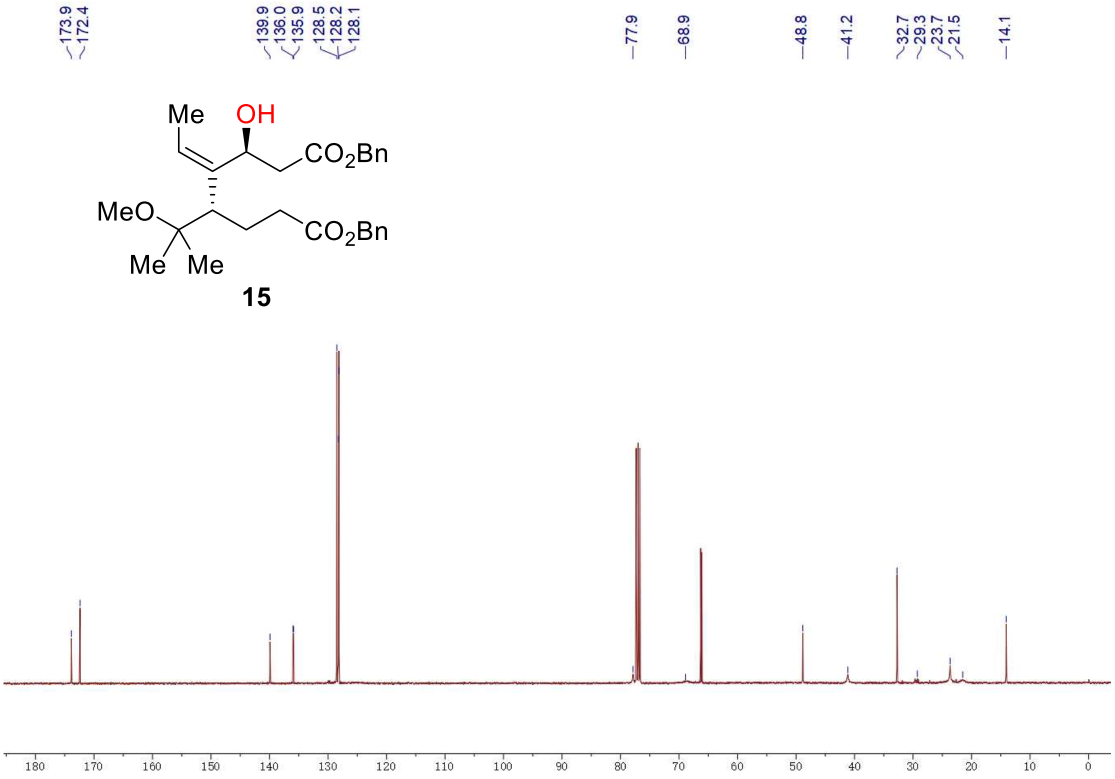

Supplementary Figure 99. ^{1}H NMR and ^{13}C NMR spectrum of compound of **16**

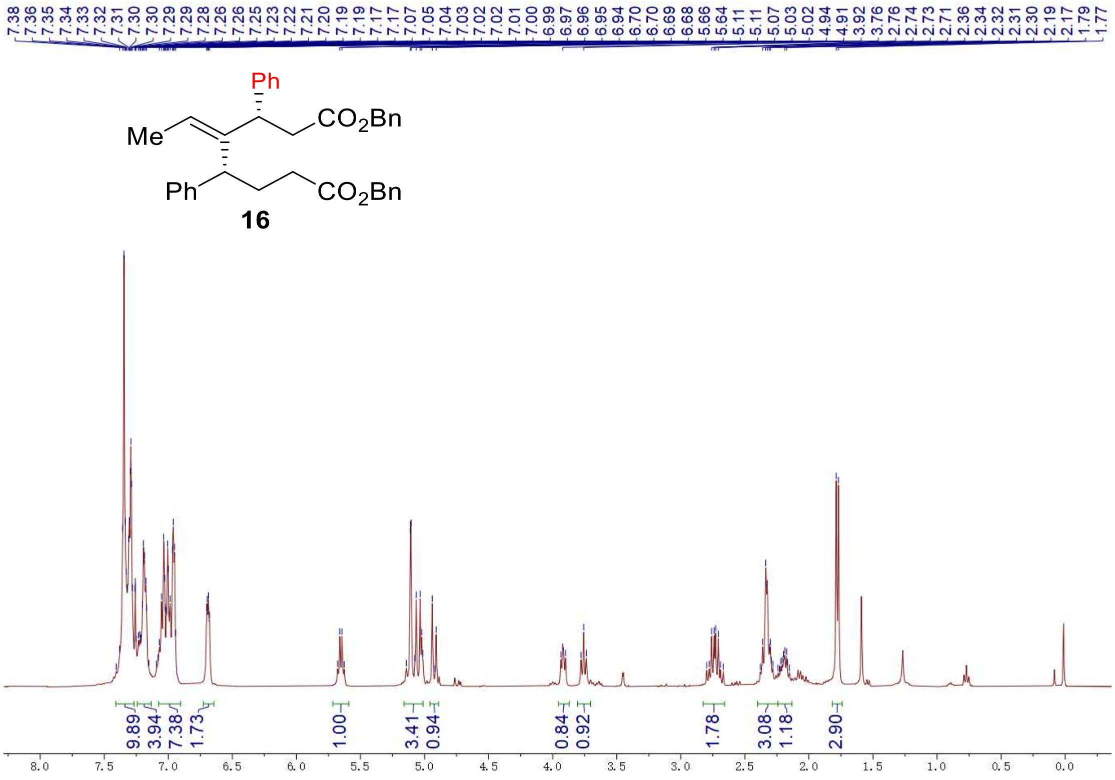

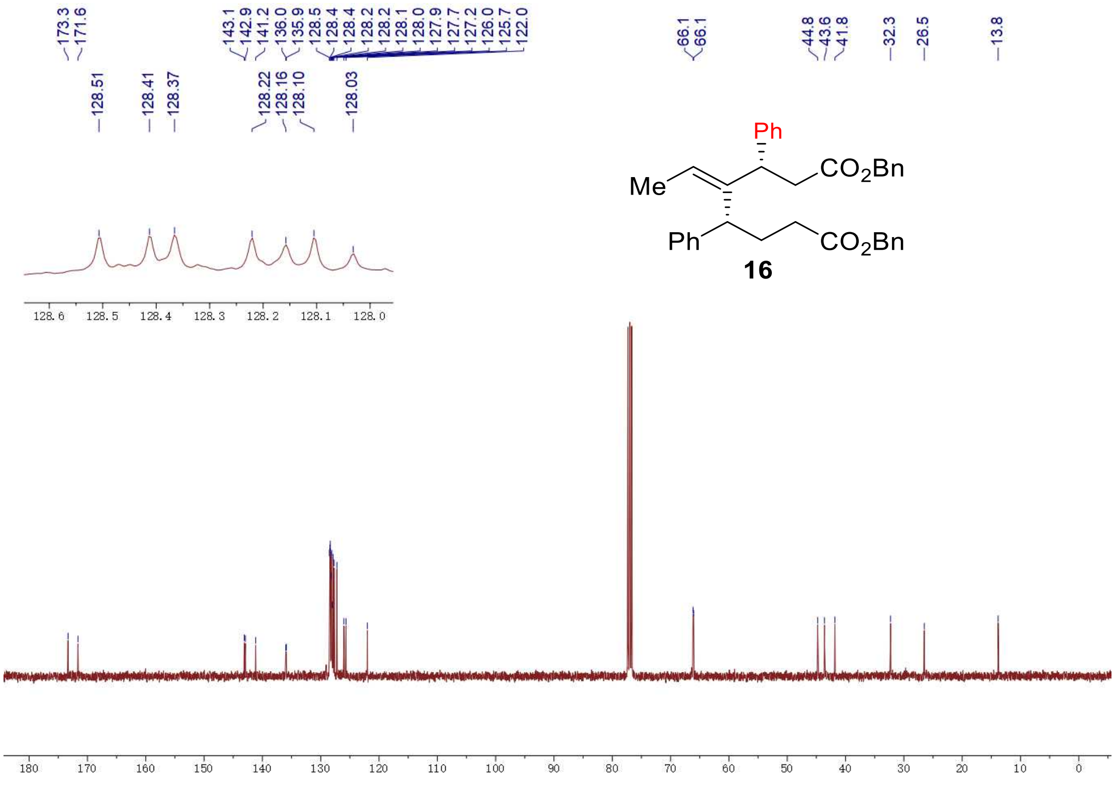

Supplementary Figure 100. ^{1}H NMR and ^{13}C NMR spectrum of compound of **17**

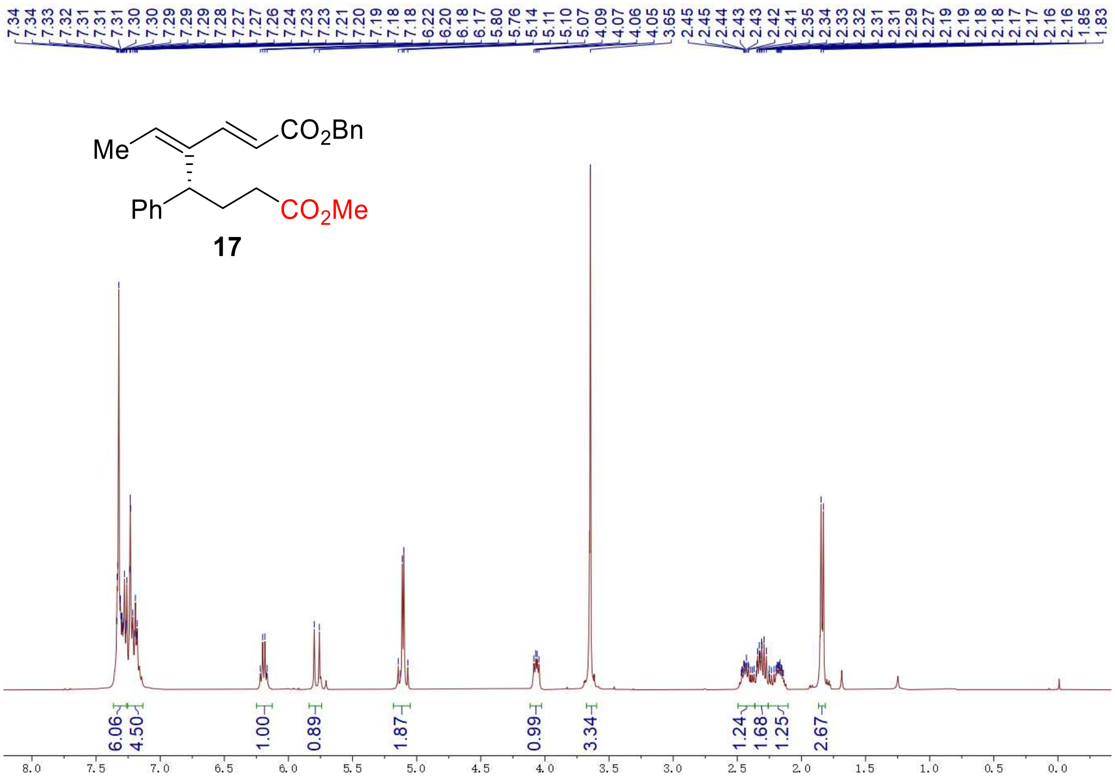

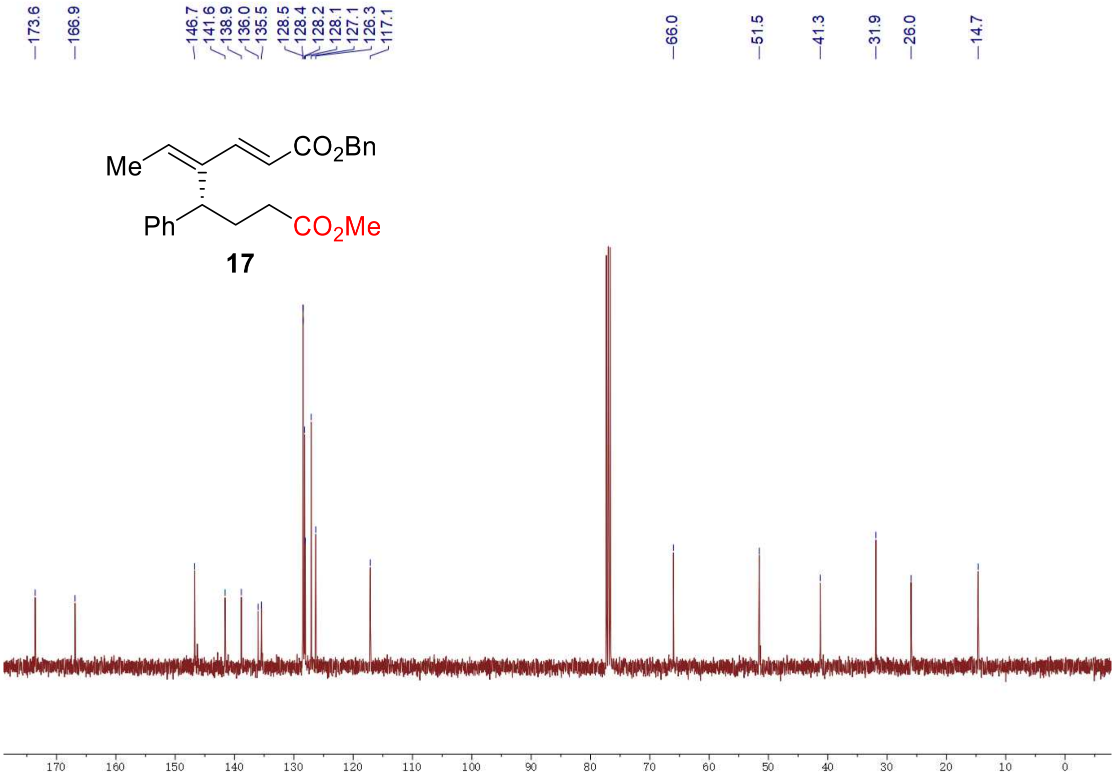

Supplementary Figure 101. ^{1}H NMR and ^{13}C NMR spectrum of compound of **18**

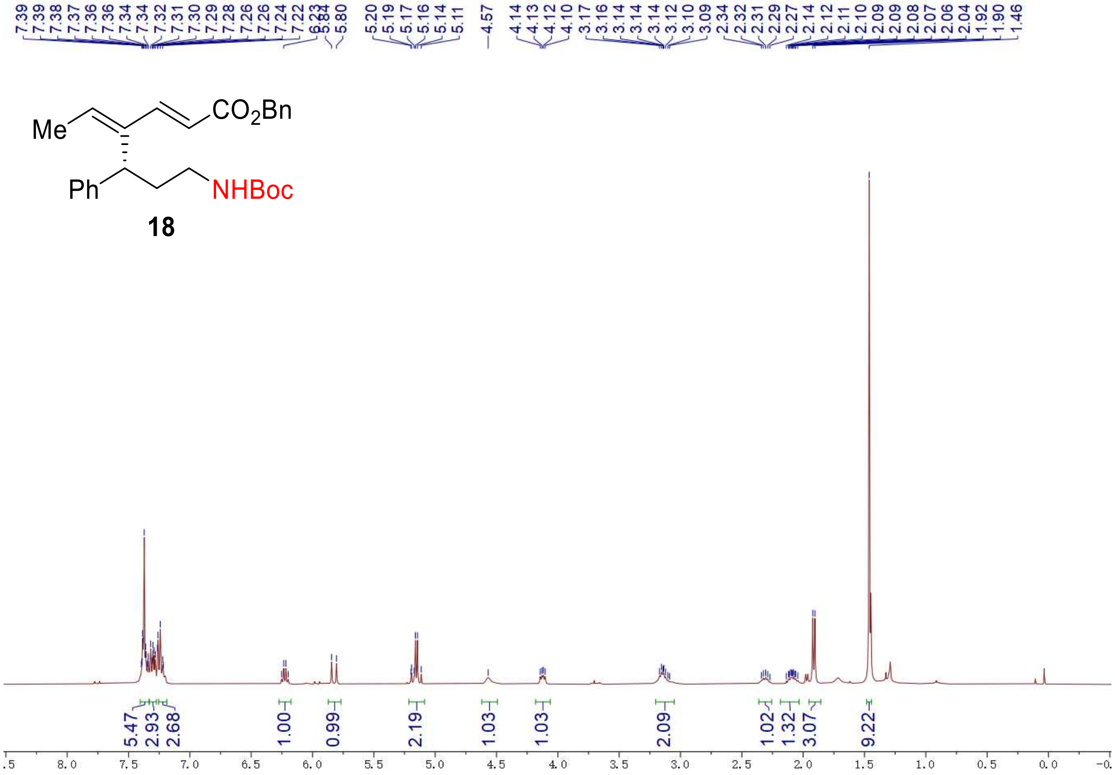

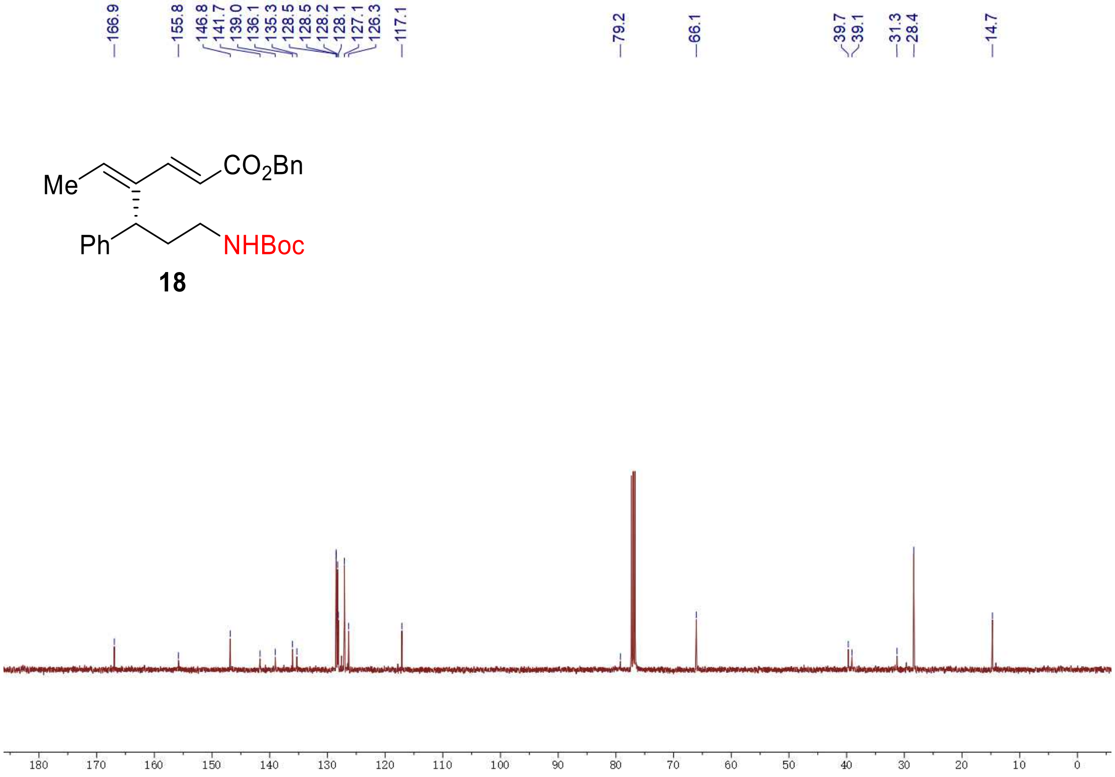

Supplementary Figure 102. ^{1}H NMR and ^{13}C NMR spectrum of compound of **19**

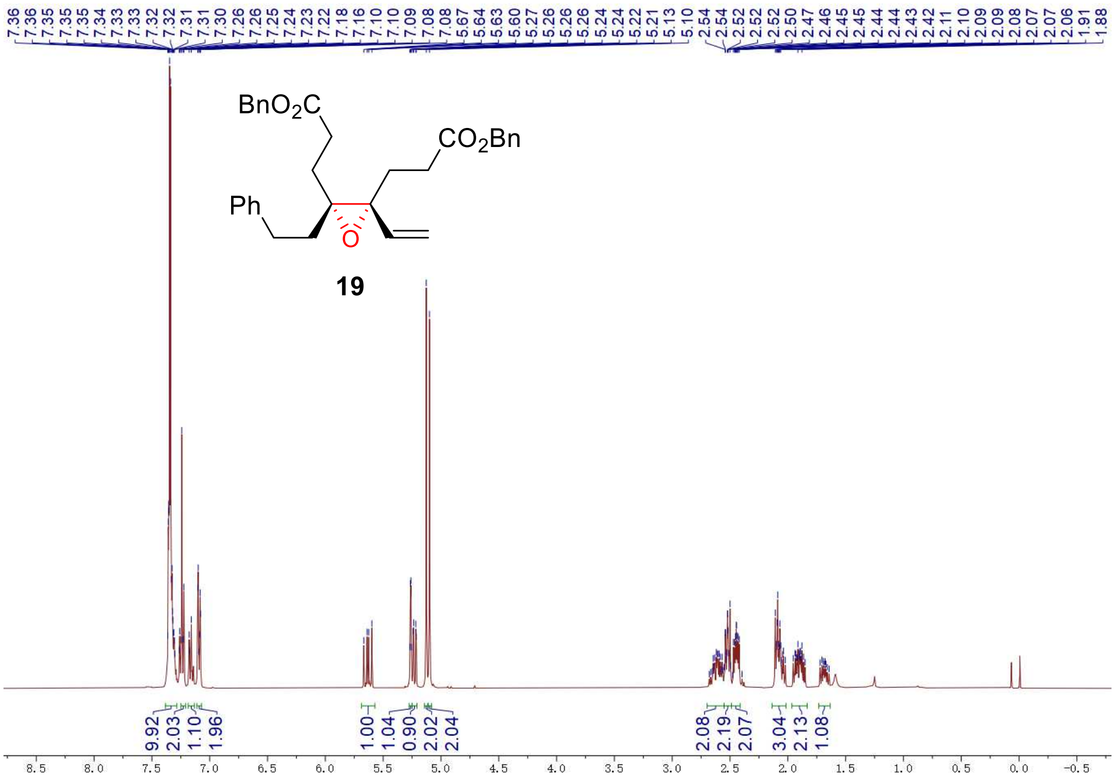

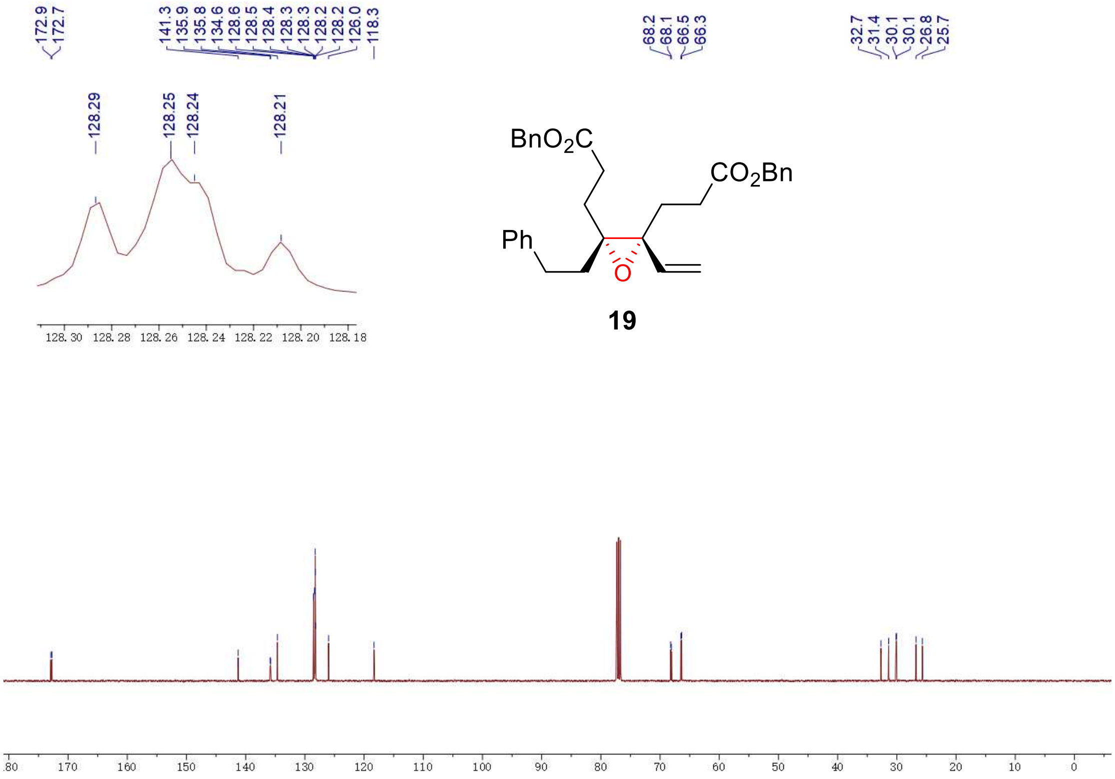

Supplementary Figure 103. ^{1}H NMR and ^{13}C NMR spectrum of compound of **20**

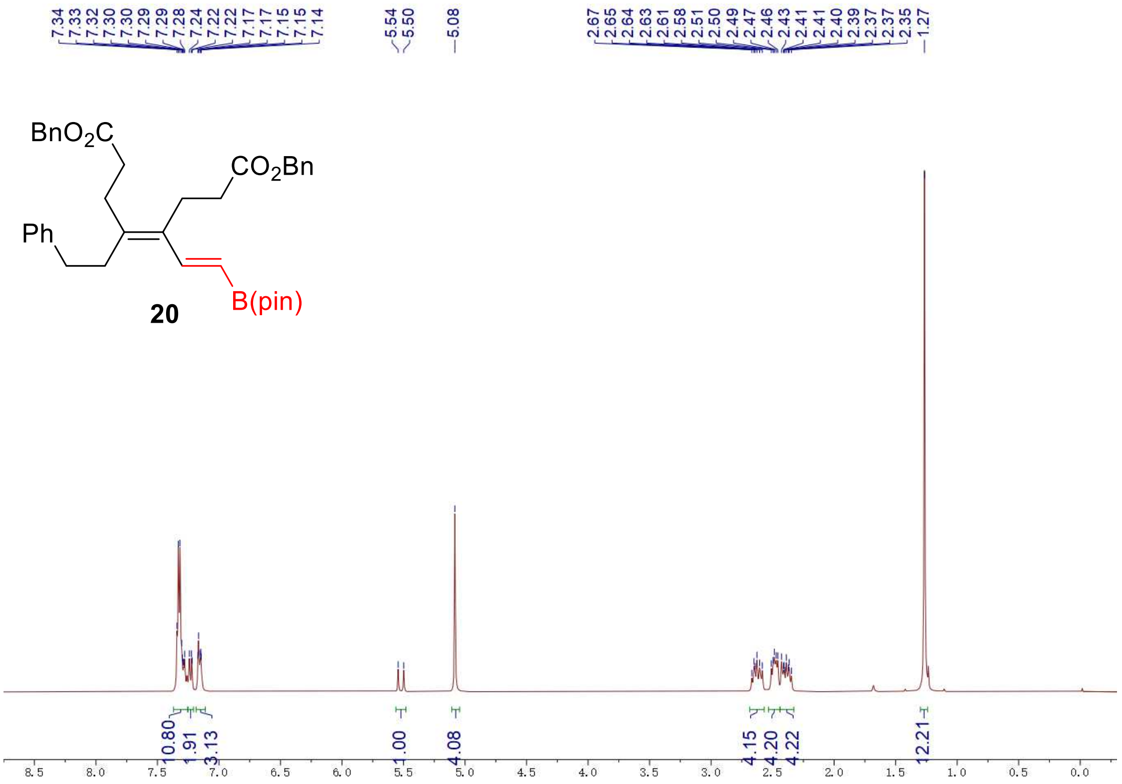

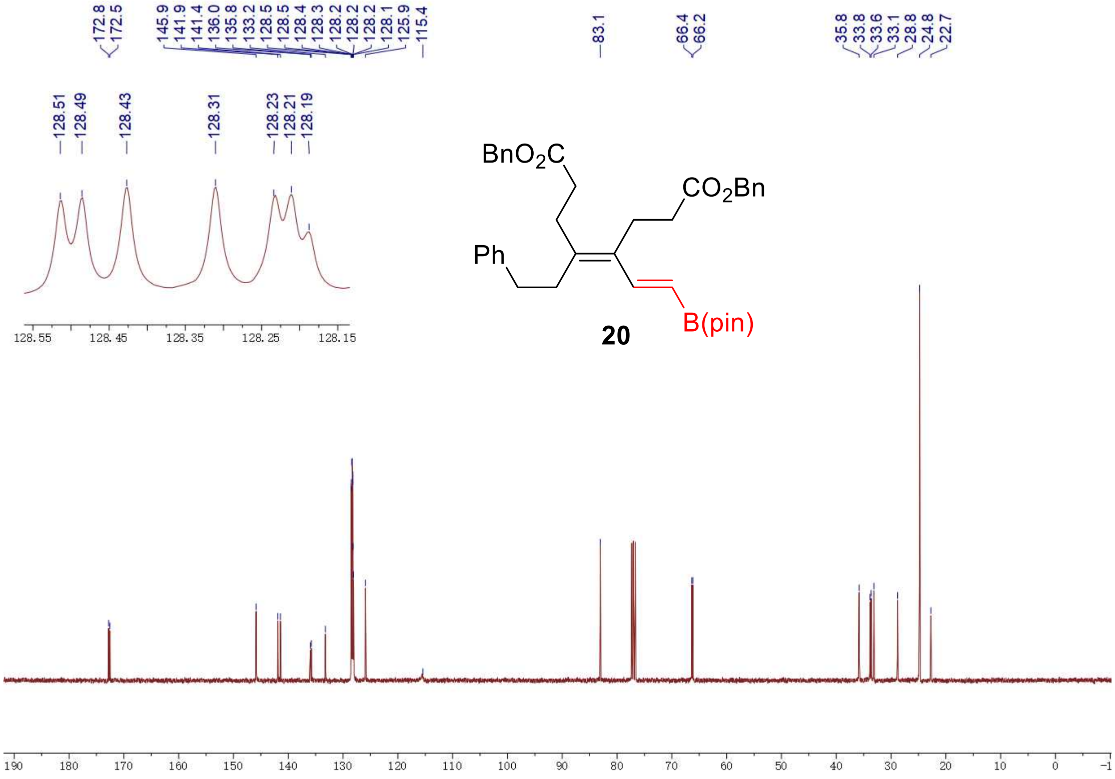

Supplementary Figure 104. ^{1}H NMR and ^{13}C NMR spectrum of compound of **21**

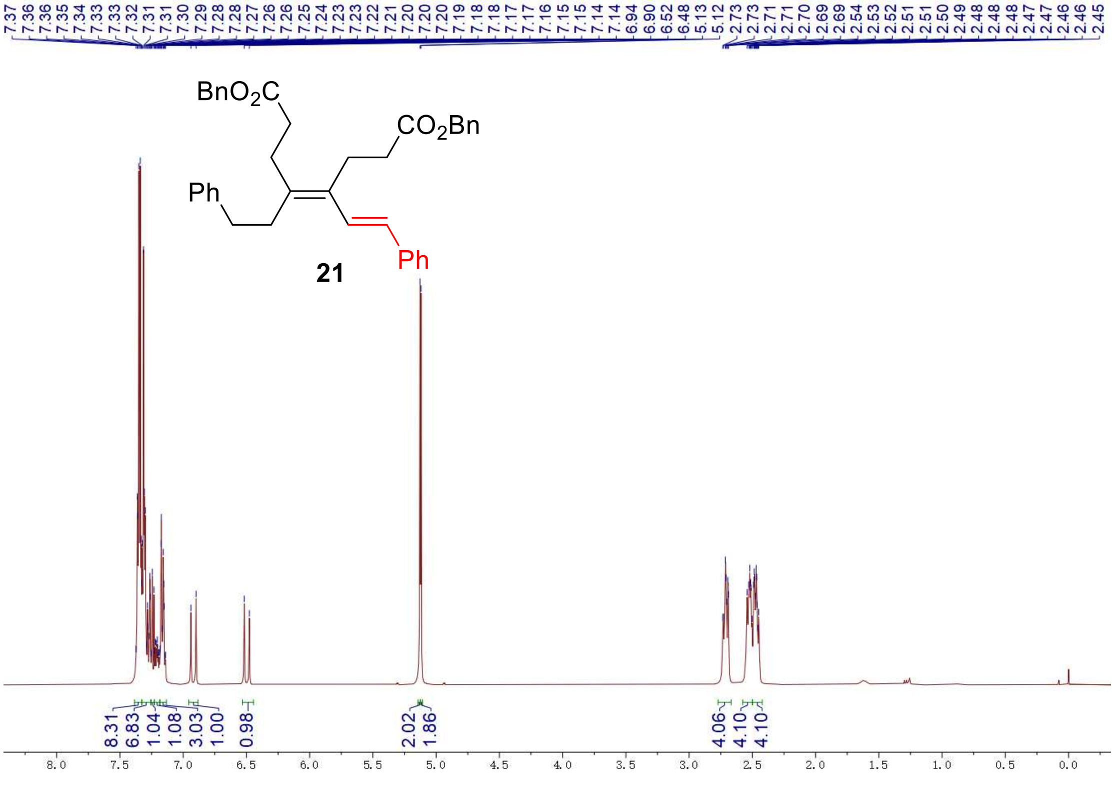

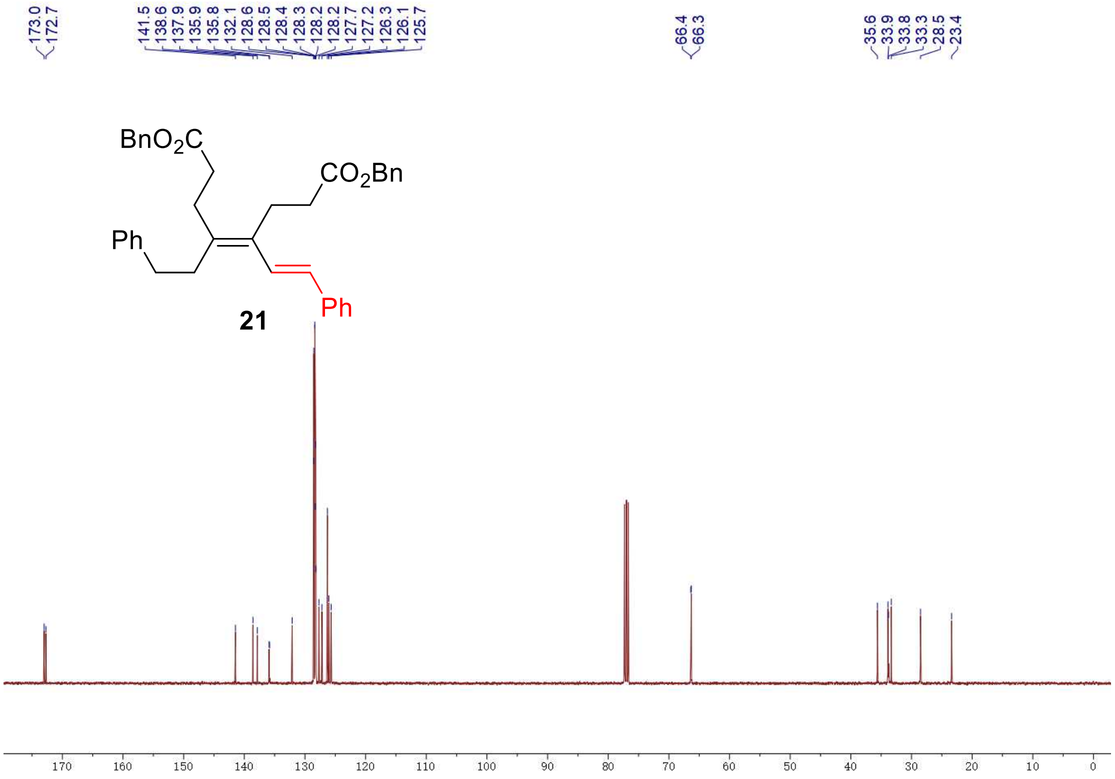

Supplementary Figure 105. ^{1}H NMR and ^{13}C NMR spectrum of compound of **22**

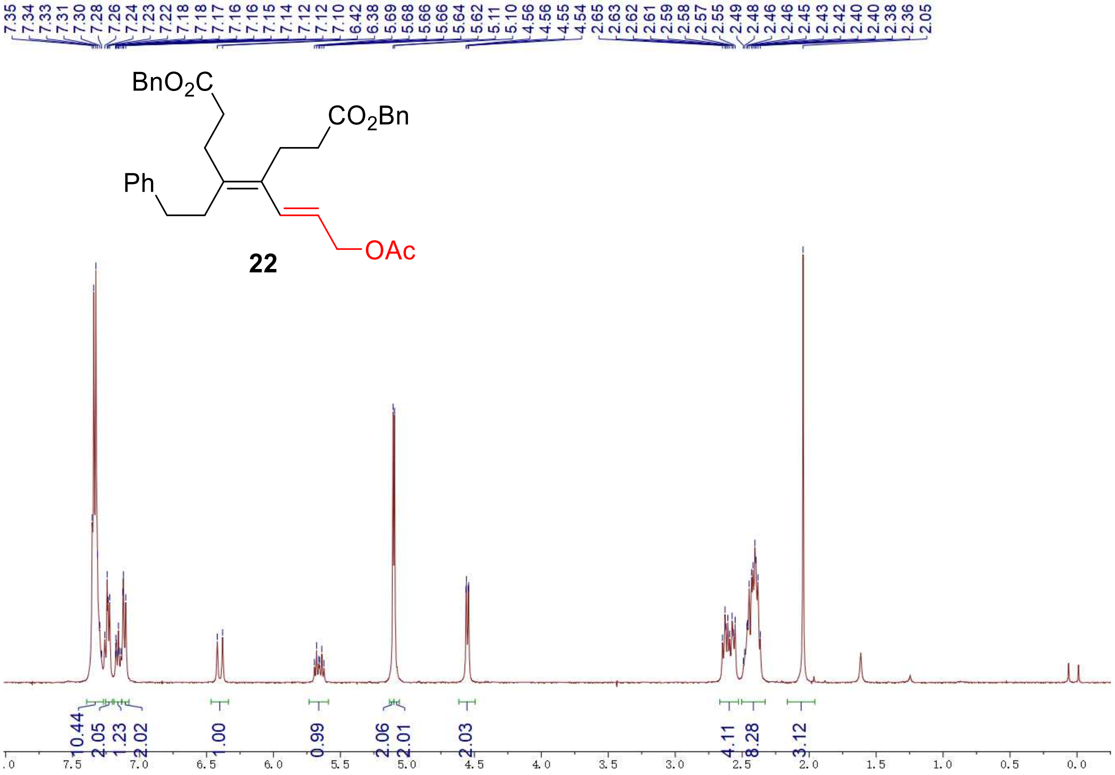

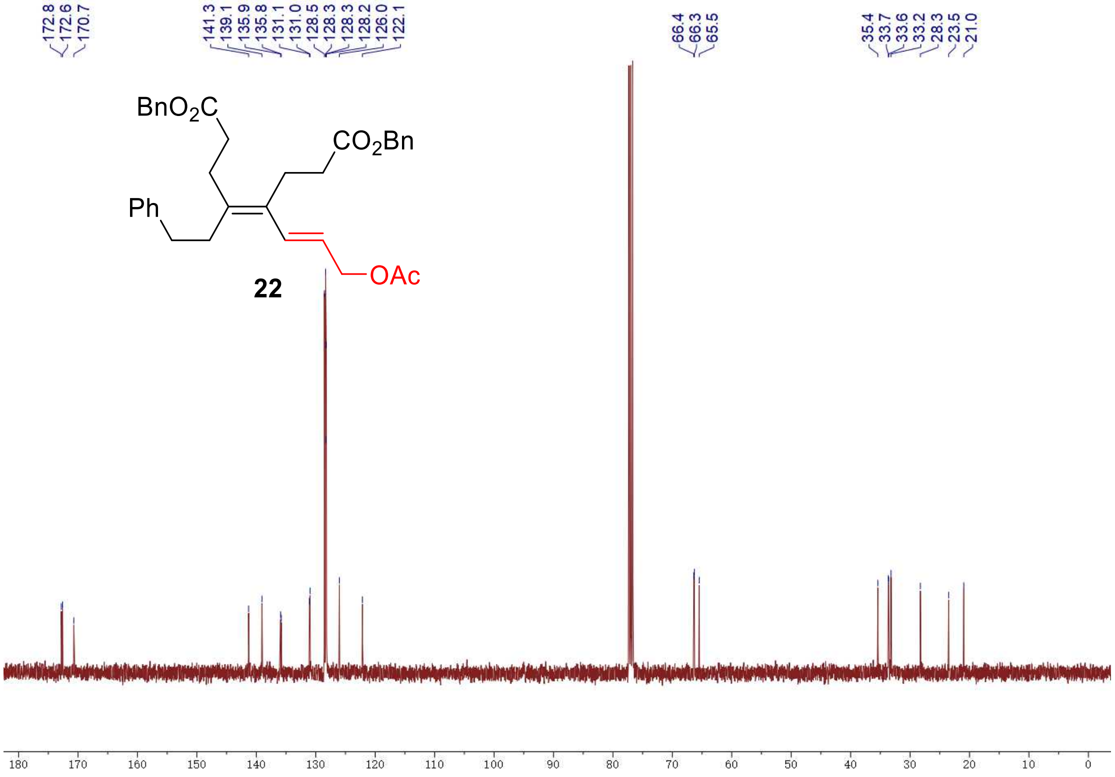

Supplementary Figure 106. ^{1}H NMR and ^{13}C NMR spectrum of compound of **24**

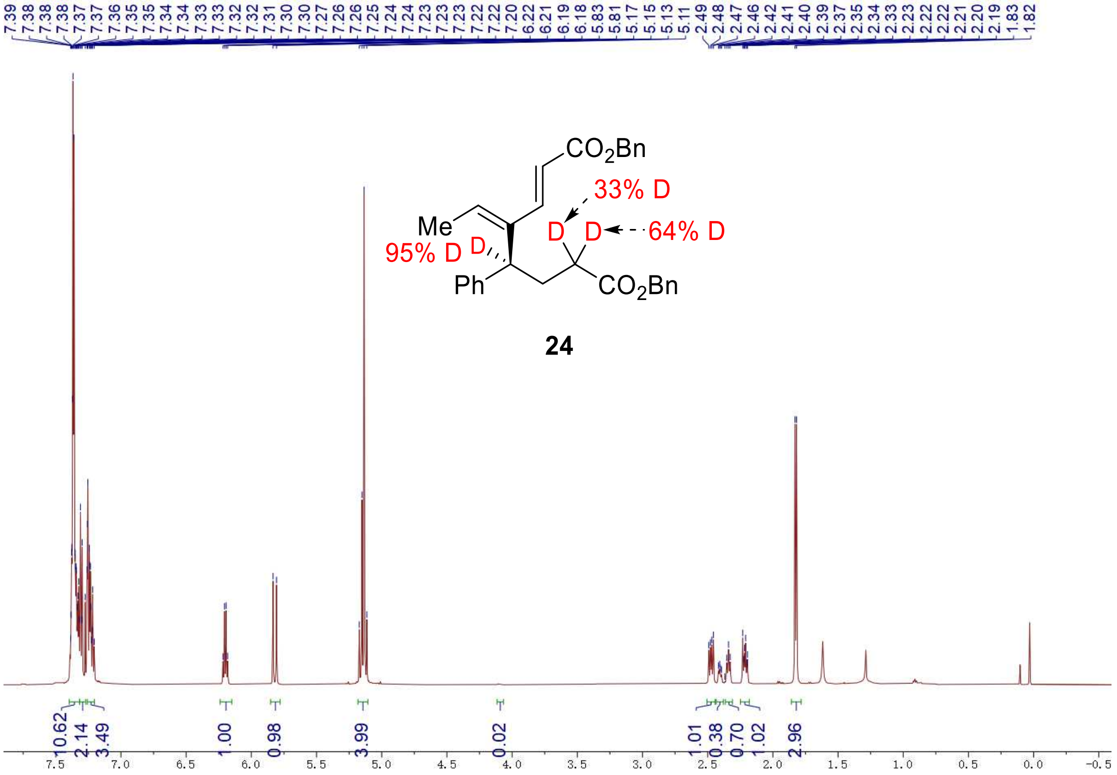

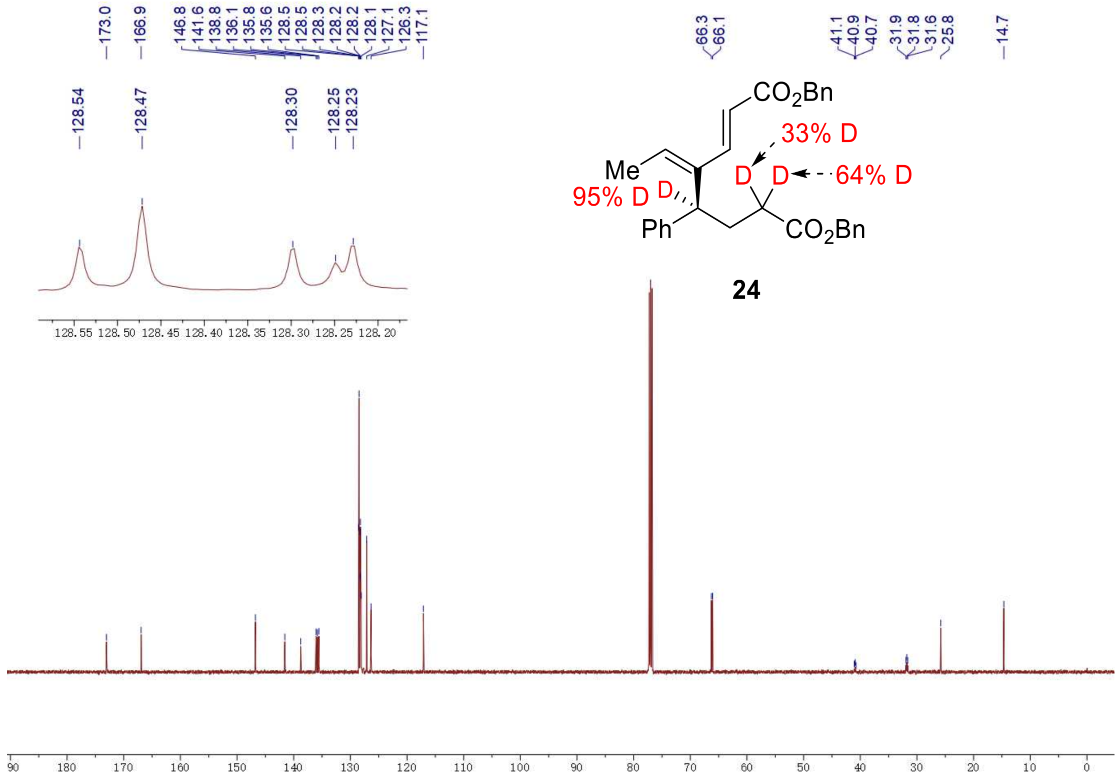

Supplementary Figure 107. ^{2}**H NMR** spectrum of compound of **24** in $CHCl_3$ ($CDCl_3$ as internal standard)

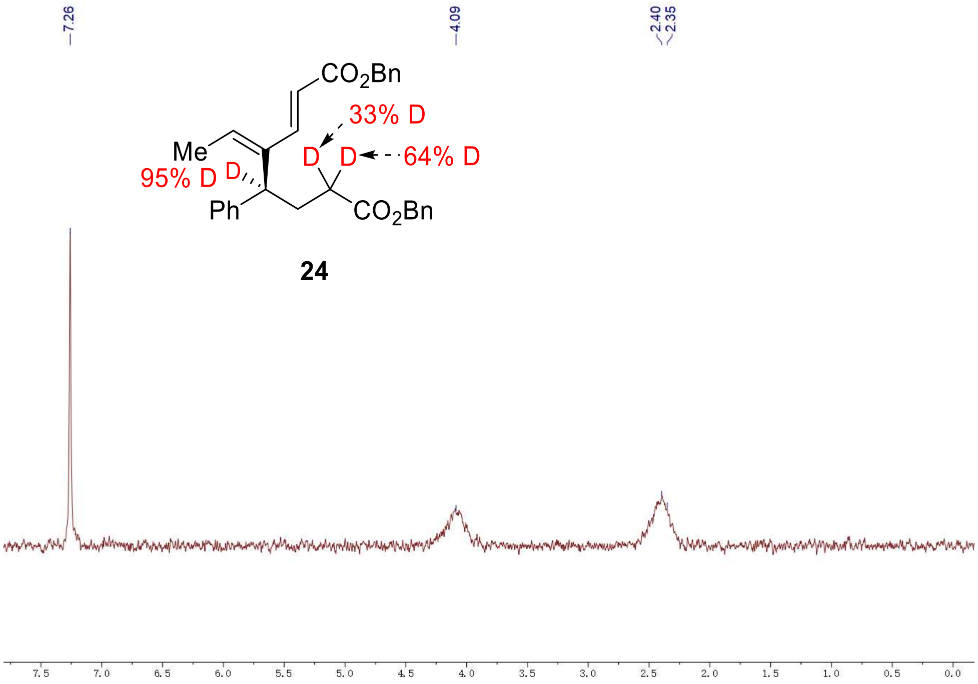

Supplementary Figure 108. ^{1}**H NMR** spectrum of compound of **25** in $CDCl_3$

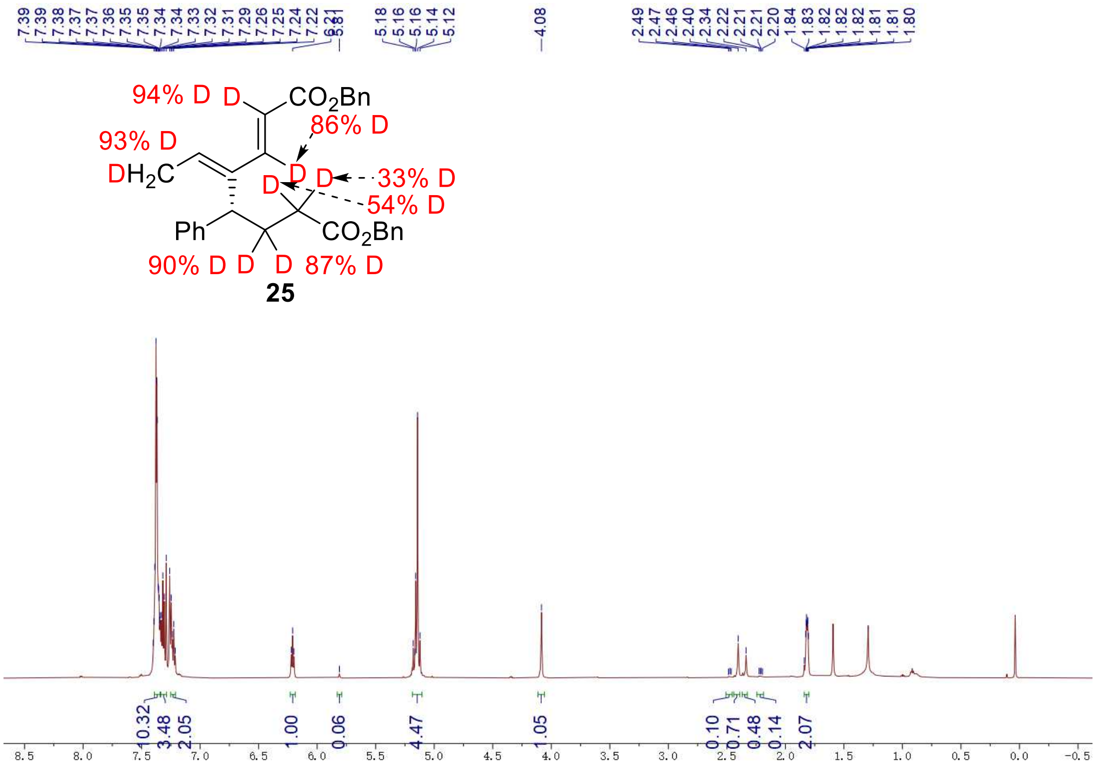

Supplementary Figure 109. ^{1}**H NMR** spectrum of compound of **25** in acetone-d_6

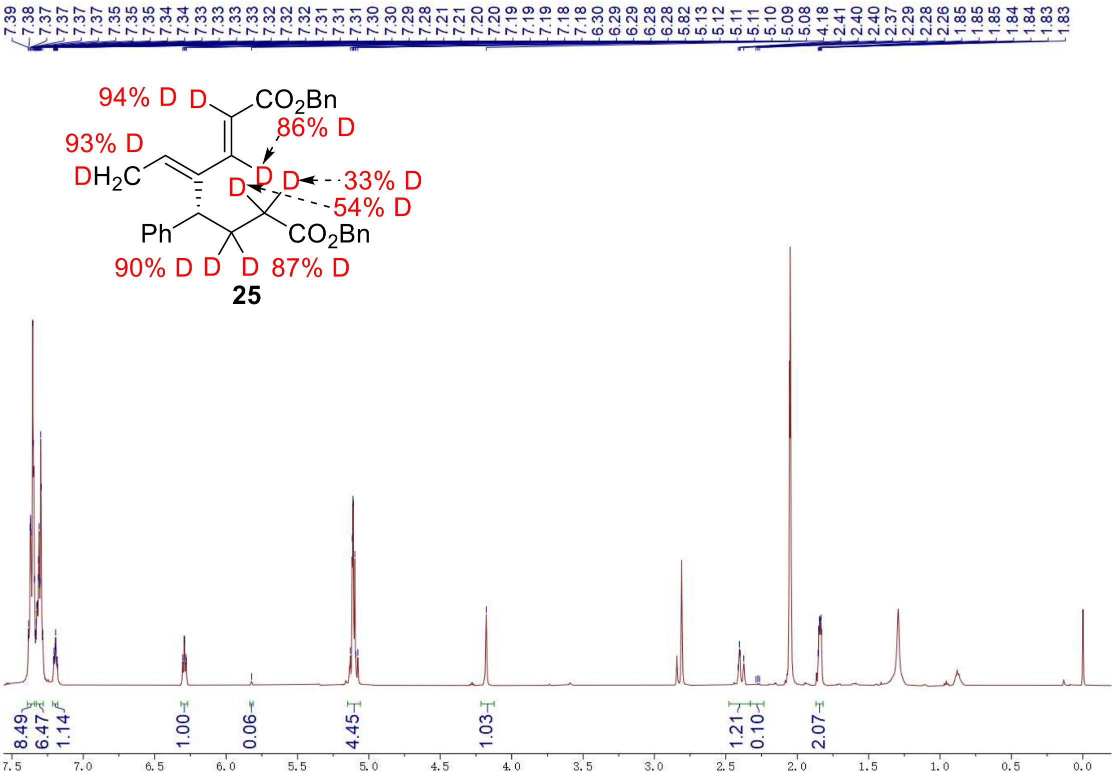

Supplementary Figure 110. ^{13}**C NMR** spectrum of compound of **25**

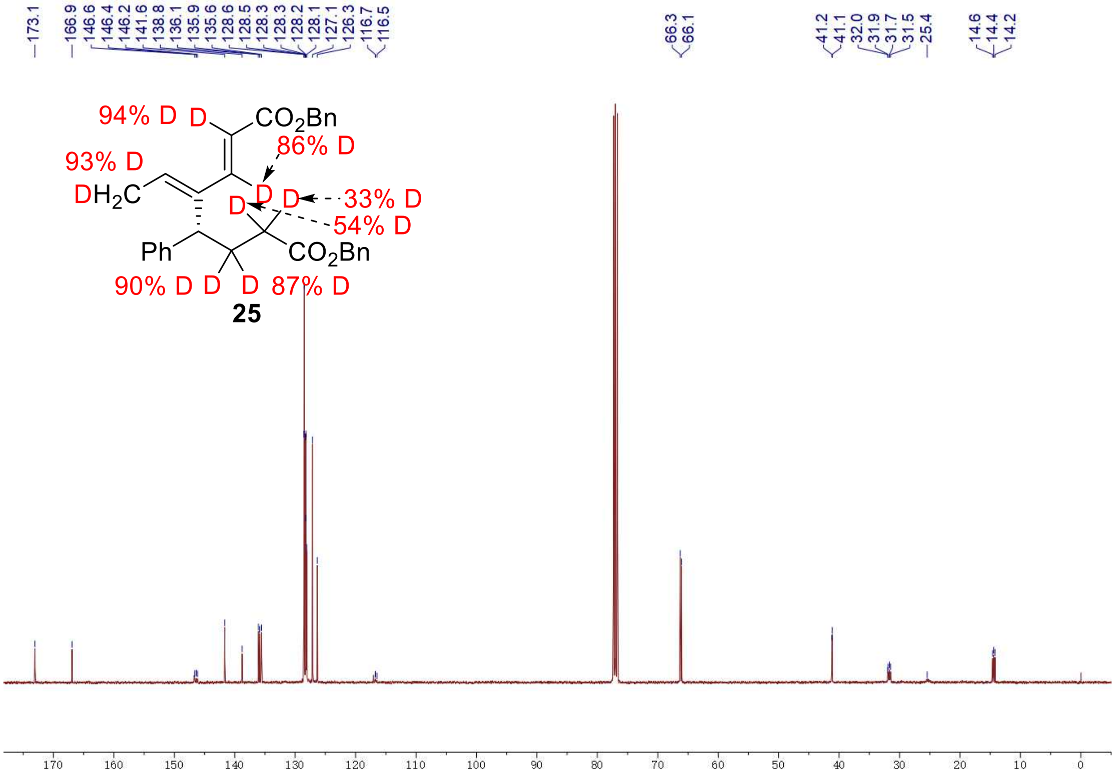

Supplementary Figure 111. ^{2}**H NMR** spectrum of compound of **25** in $CHCl_3$ ($CDCl_3$ as internal standard)

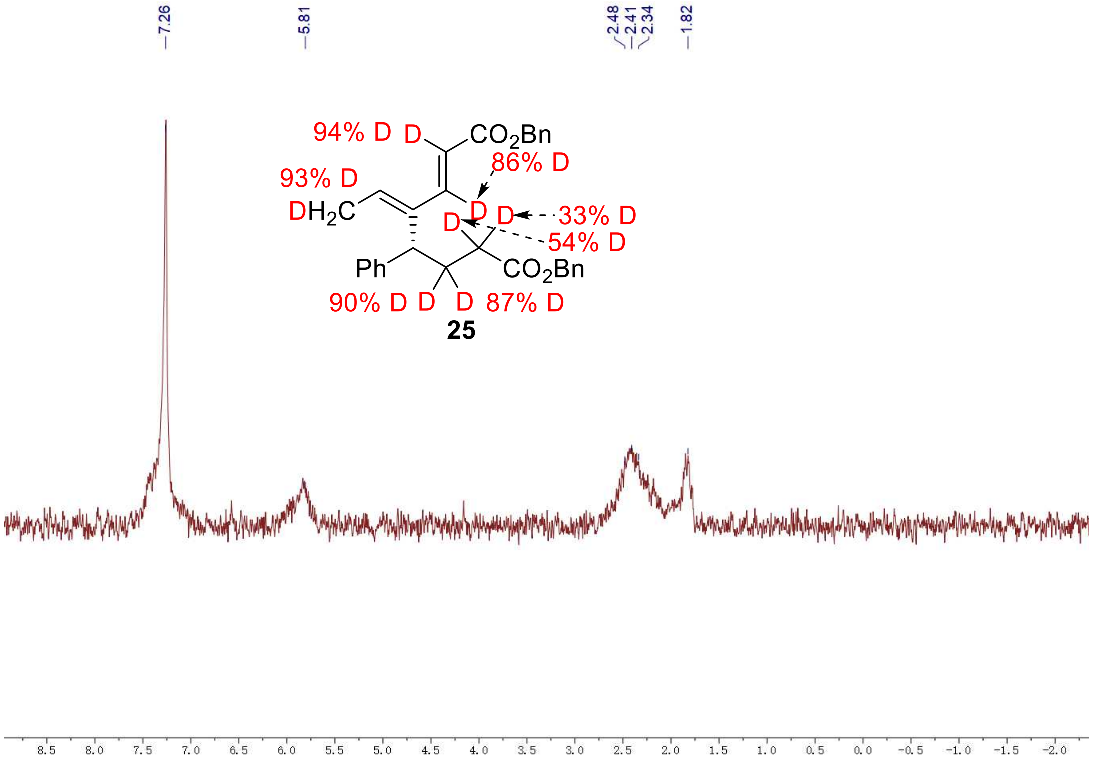

Supplementary Figure 112. ^{2}H NMR spectrum of compound of **25** in $CHCl_3$ (no internal standard)

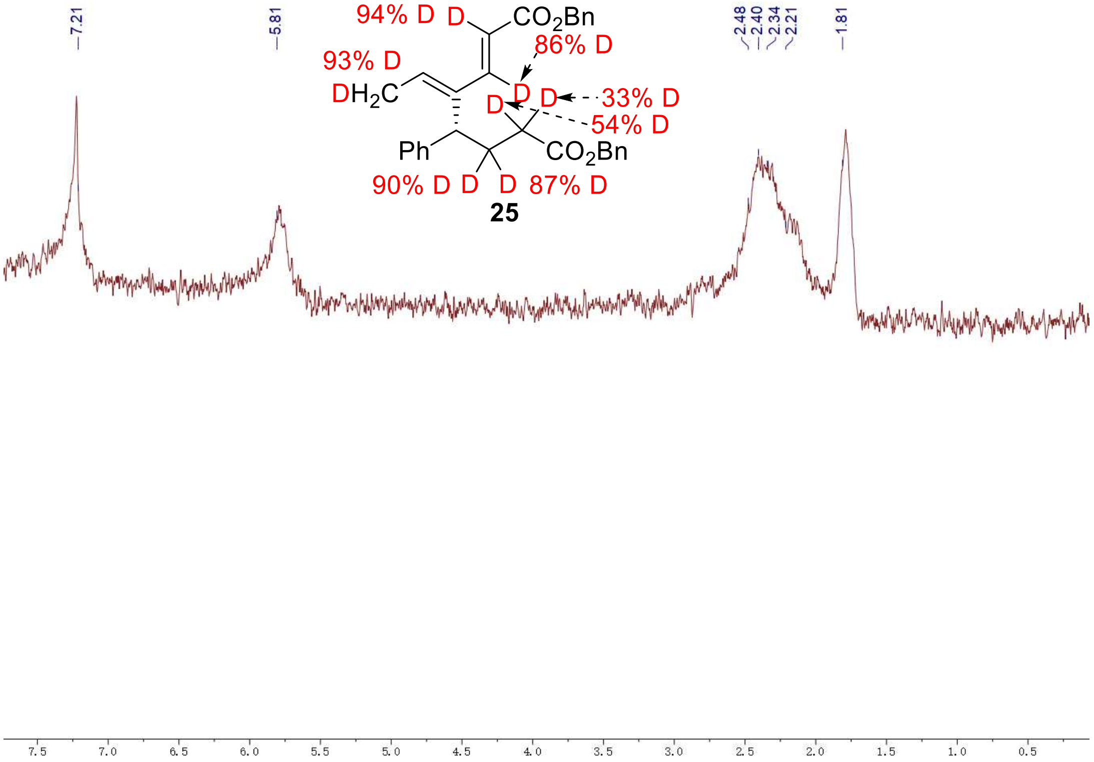

Supplementary Figure 113. ^{1}H NMR and ^{13}C NMR spectrum of compound of **26**

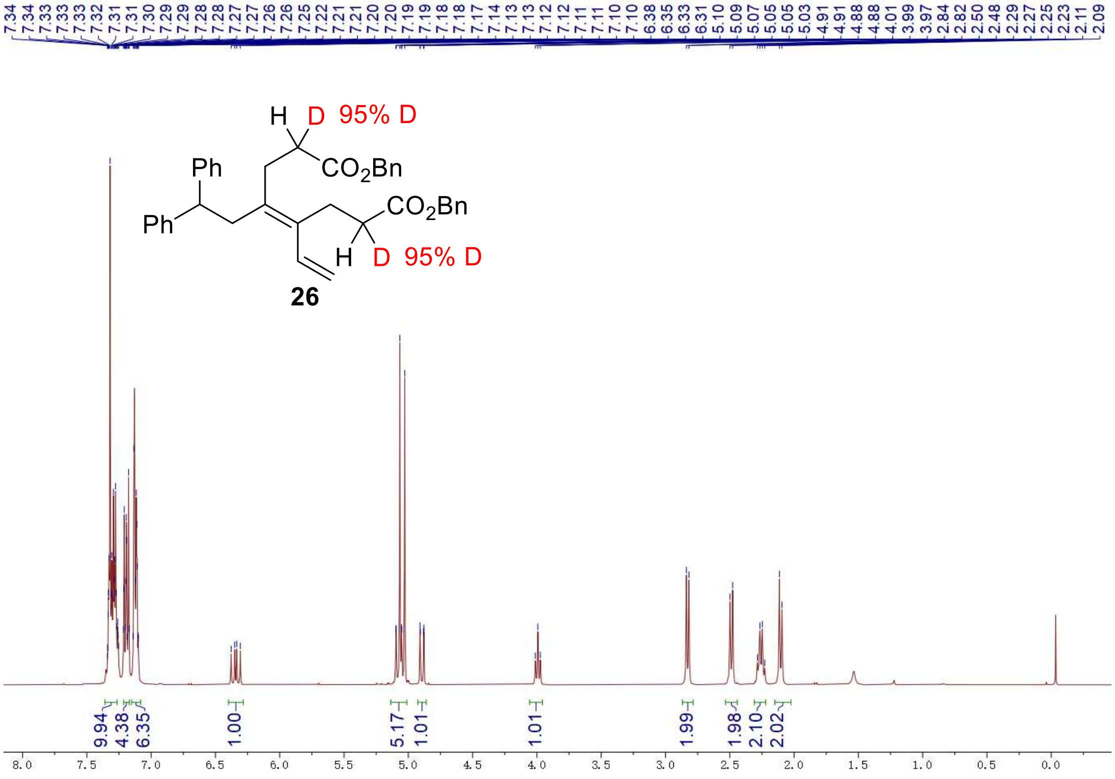

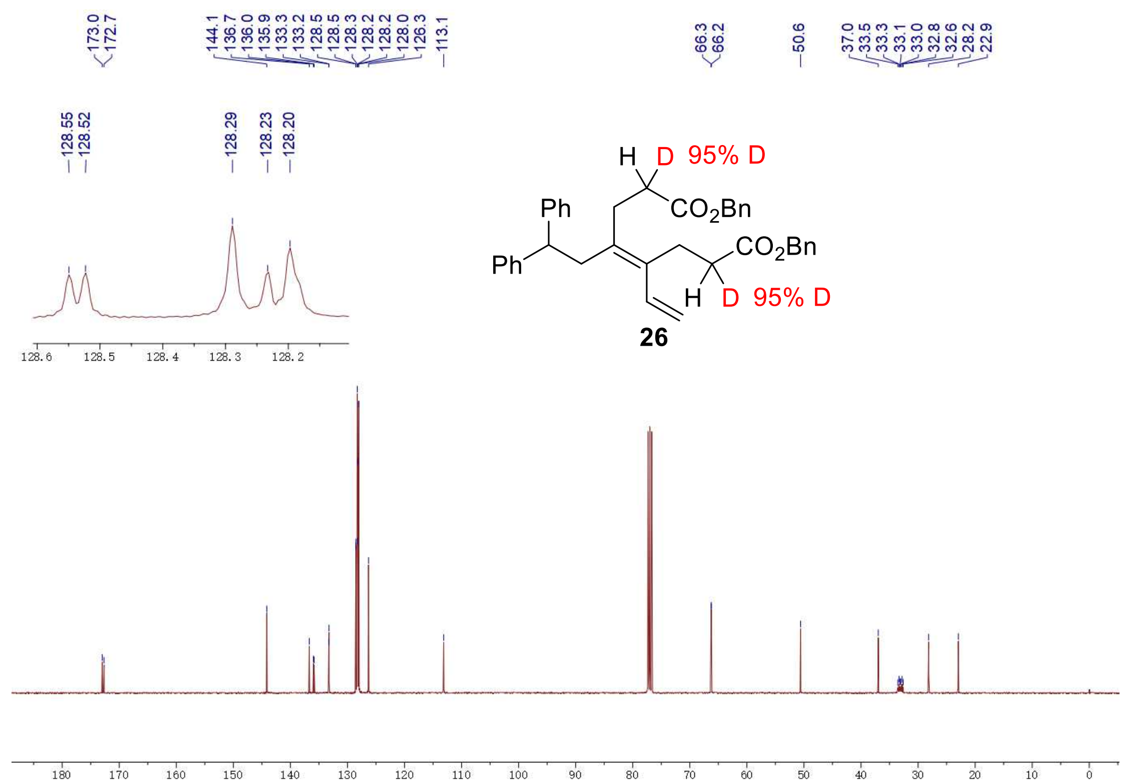

Supplementary Figure 114. ^{2}H NMR spectrum of compound of **26** in $CHCl_3$ ($CDCl_3$ as internal standard)

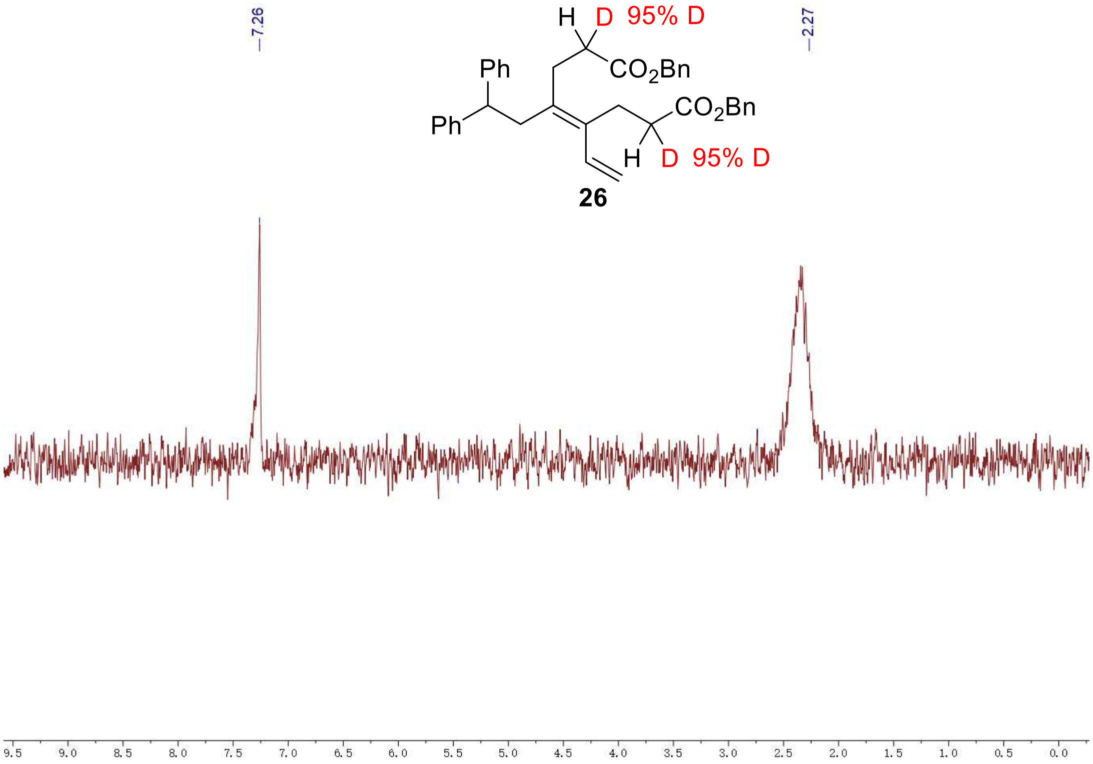

Supplementary Figure 115. ^{1}**H NMR and** ^{13}**C NMR** spectrum of compound of **27**

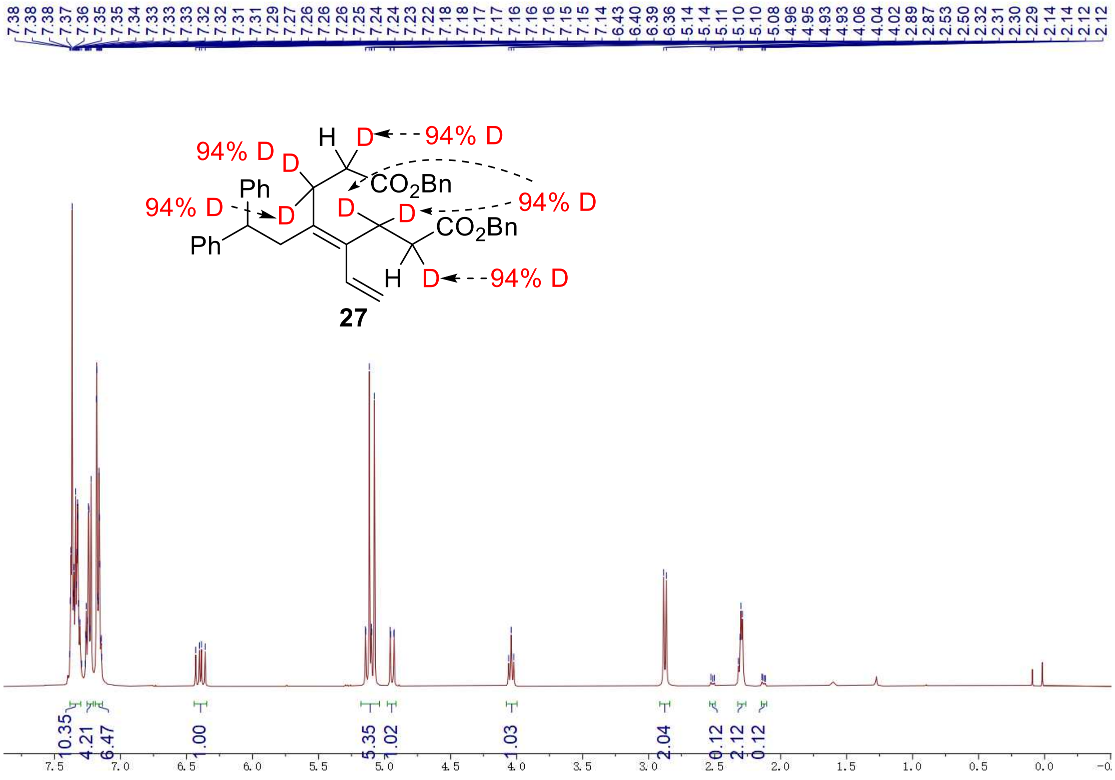

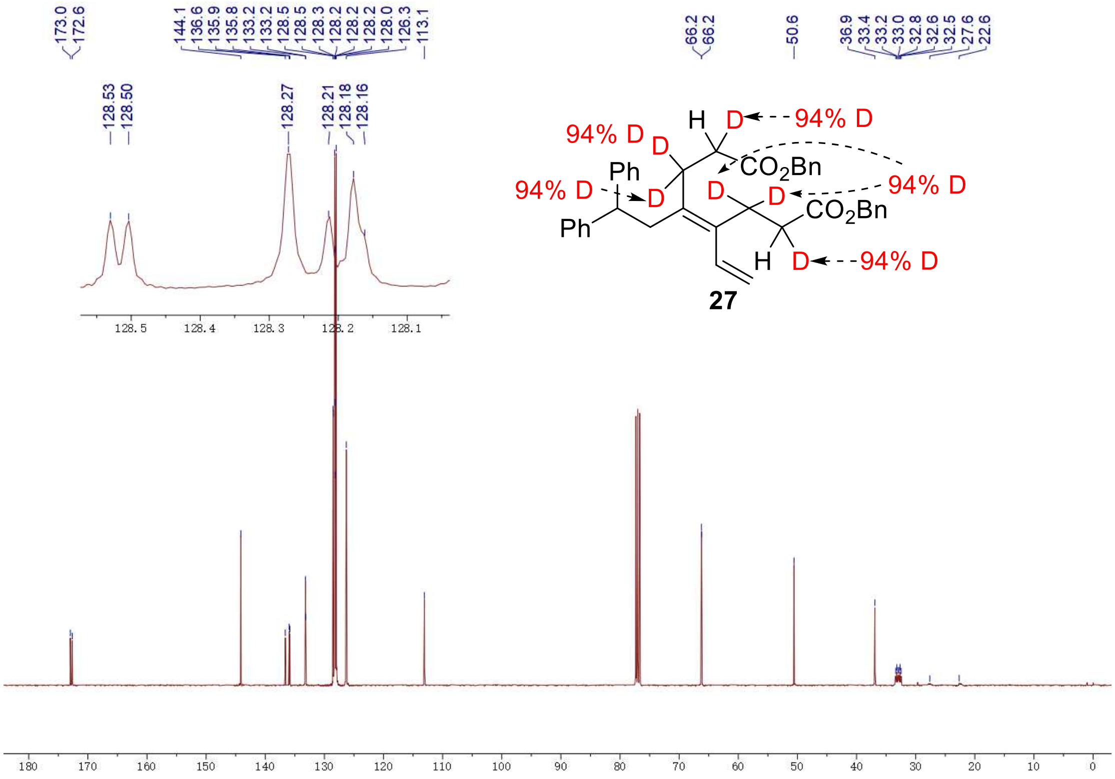

Supplementary Figure 116. ^{2}H NMR spectrum of compound of **27** in $CHCl_3$($CDCl_3$ as internal standard)

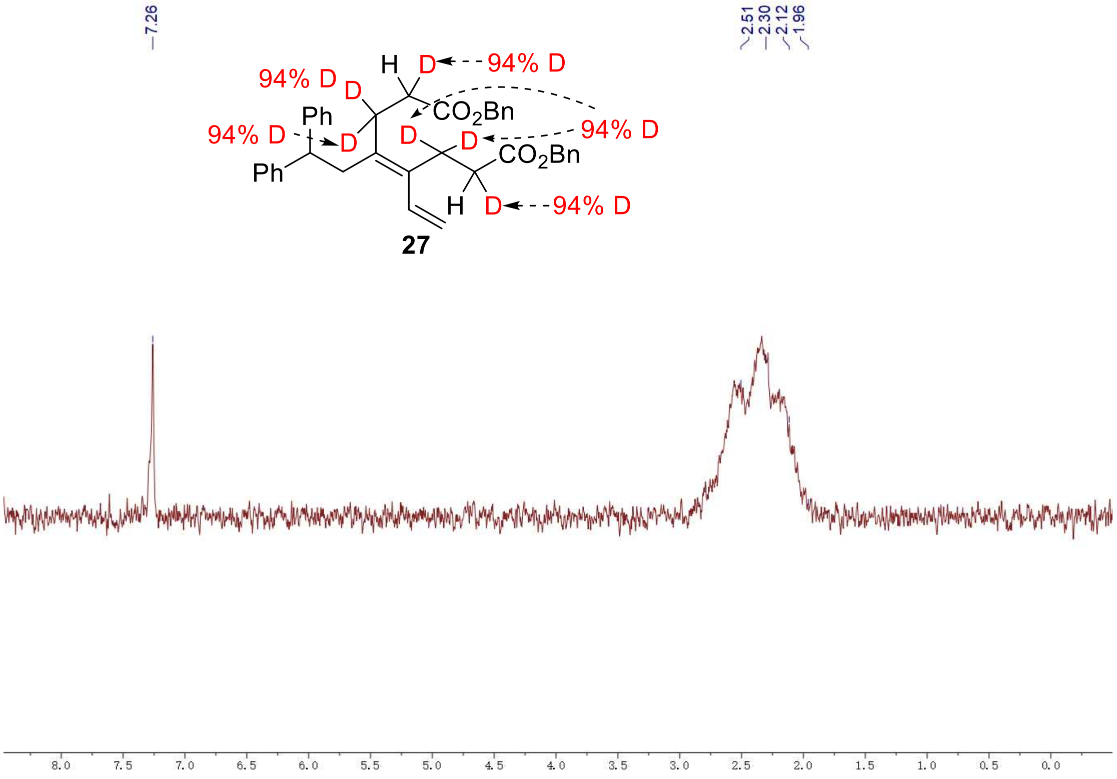

Supplementary Figure 117. ^{1}H NMR and ^{13}C NMR spectrum of compound of **28**

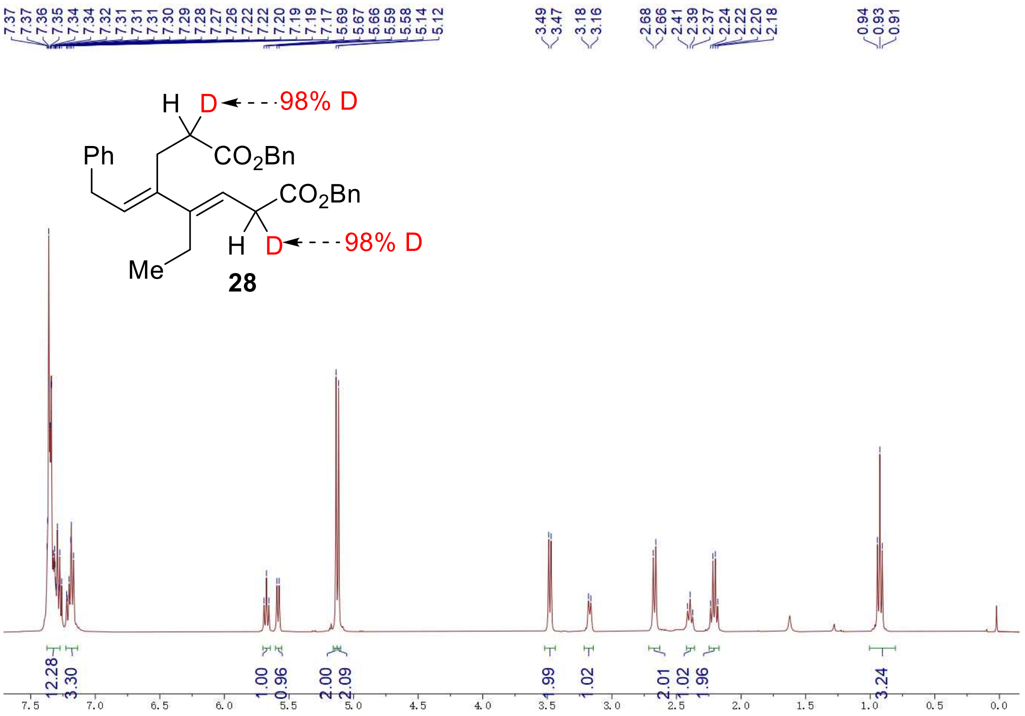

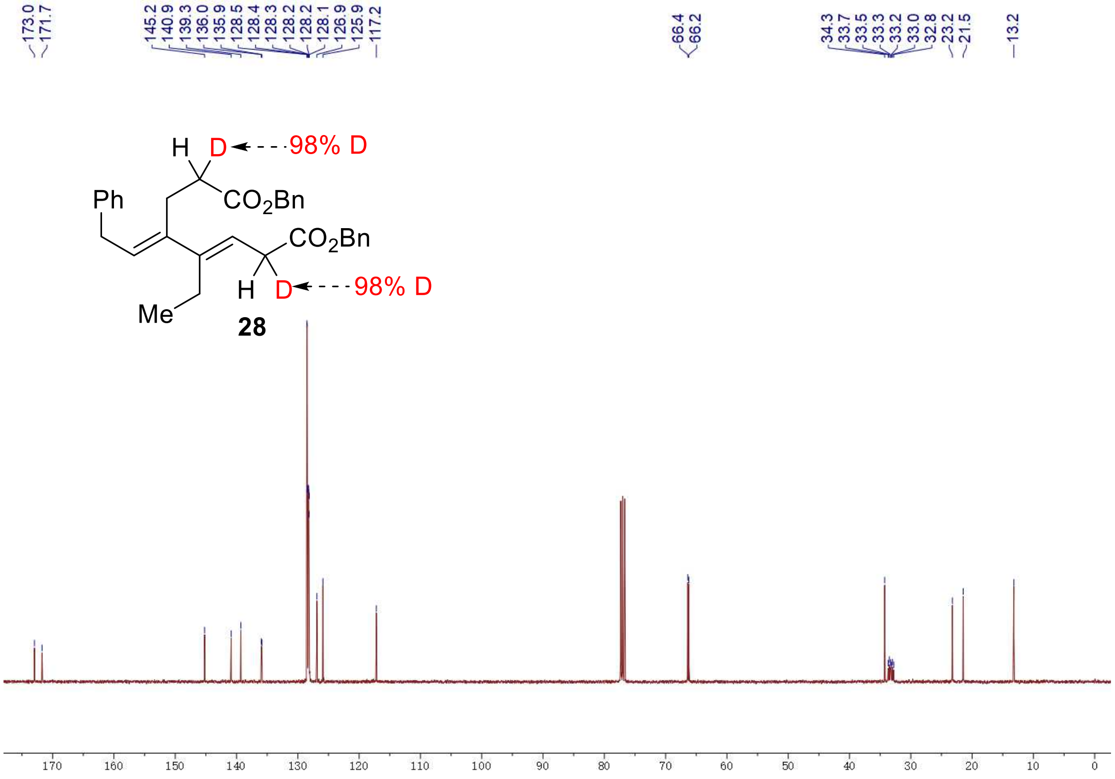

Supplementary Figure 118. ^{2}H NMR spectrum of compound of **28** in $CHCl_3$ ($CDCl_3$ as internal standard)

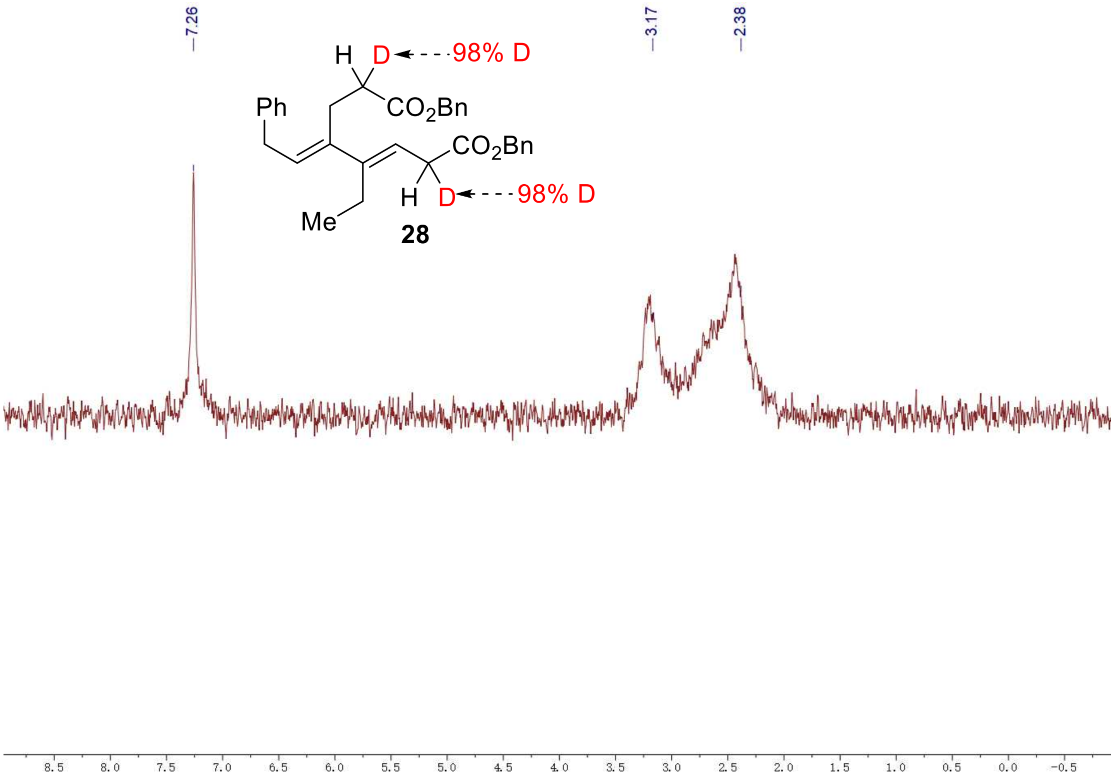

Supplementary Figure 119. ^{1}H NMR and ^{13}C NMR spectrum of compound of **29**

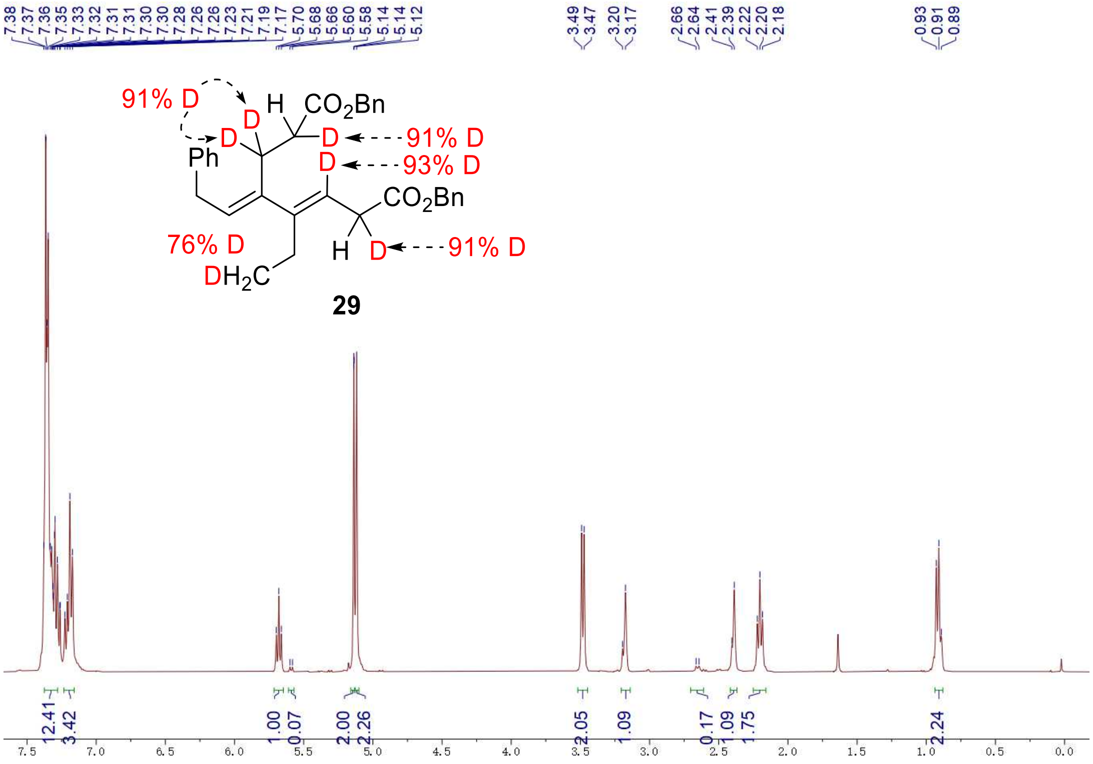

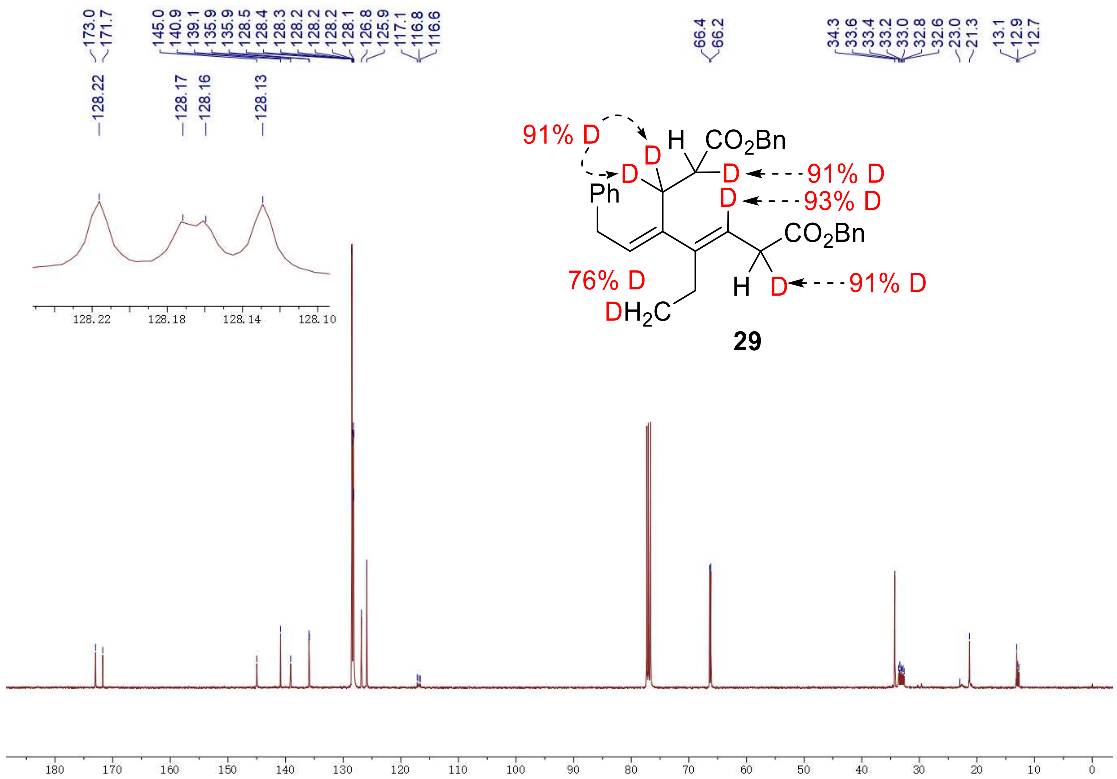

Supplementary Figure 120. ^{2}H NMR spectrum of compound of **29** in $CHCl_3$ ($CDCl_3$ as internal standard)

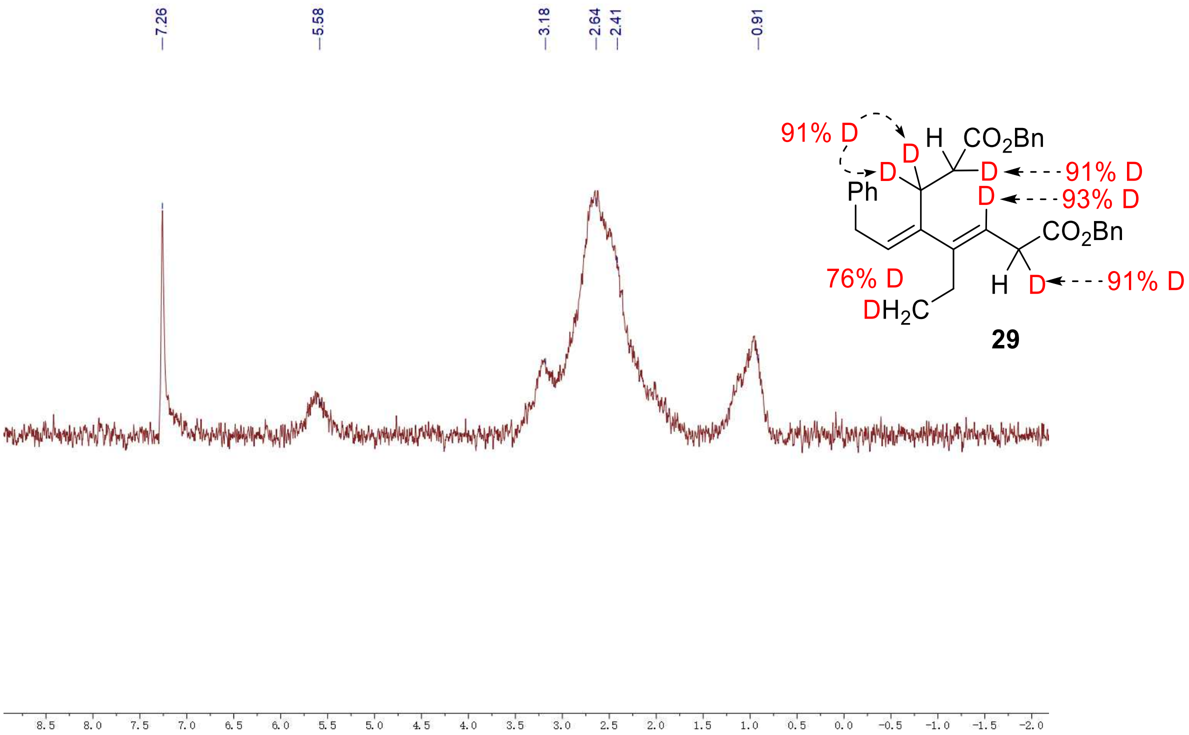

Supplementary Figure 121. ^{1}H NMR and ^{13}C NMR spectrum of compound of **30**

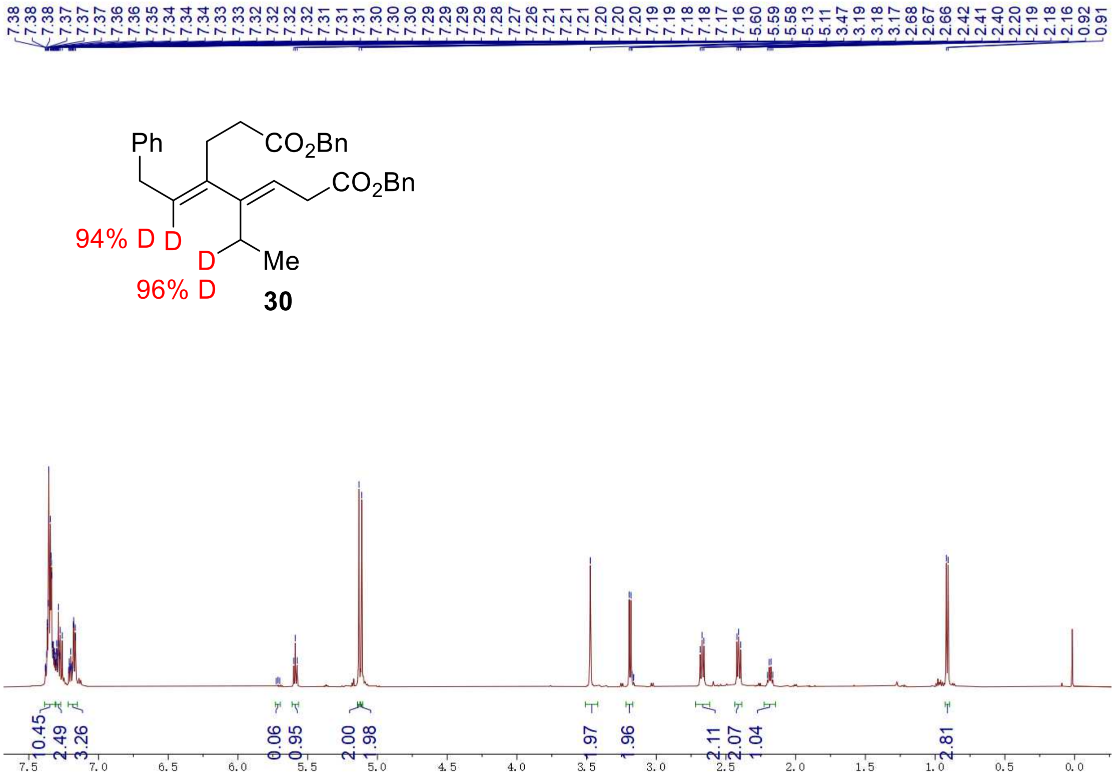

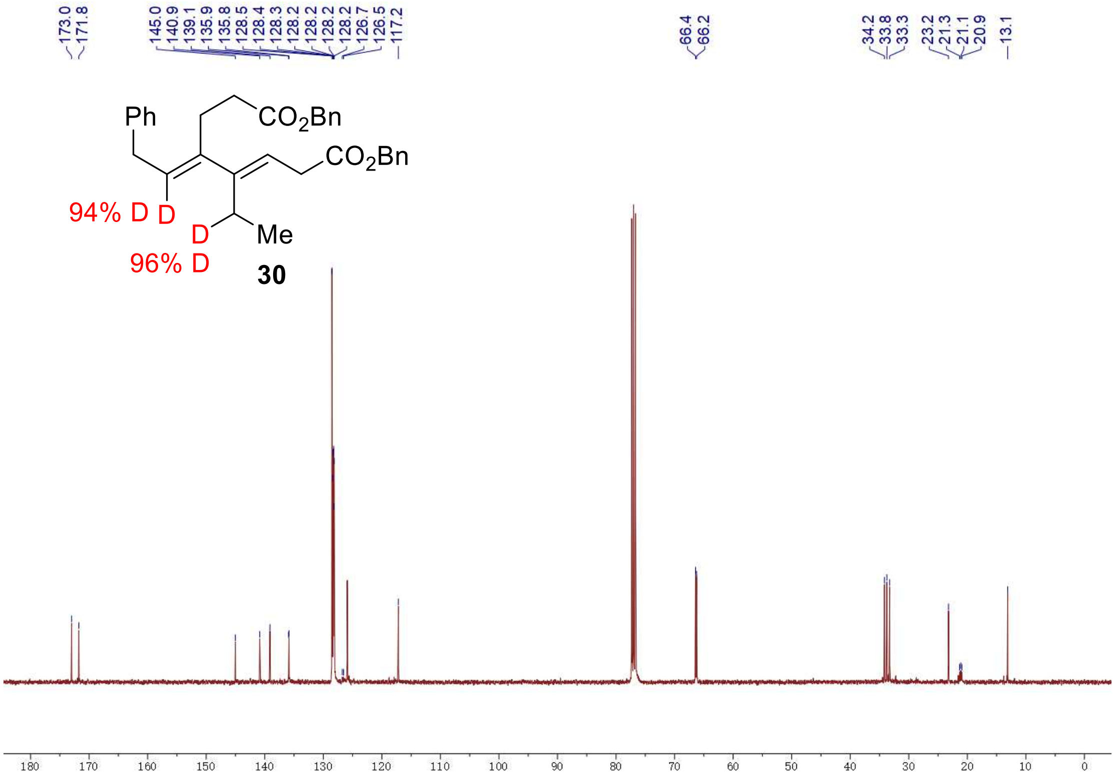

Supplementary Figure 122. ^{2}H NMR spectrum of compound of **30** in $CHCl_3$ ($CDCl_3$ as internal standard)

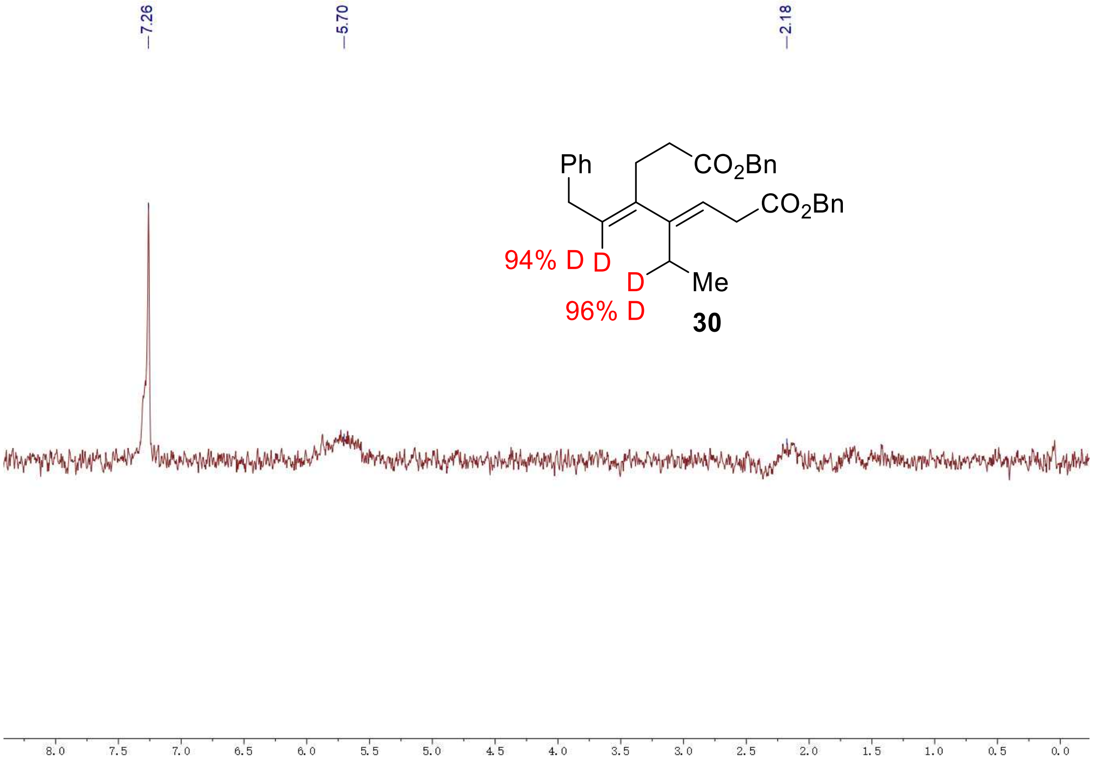

Supplementary Figure 123. ^{1}H NMR spectrum of compound of **31** in $CDCl_3$

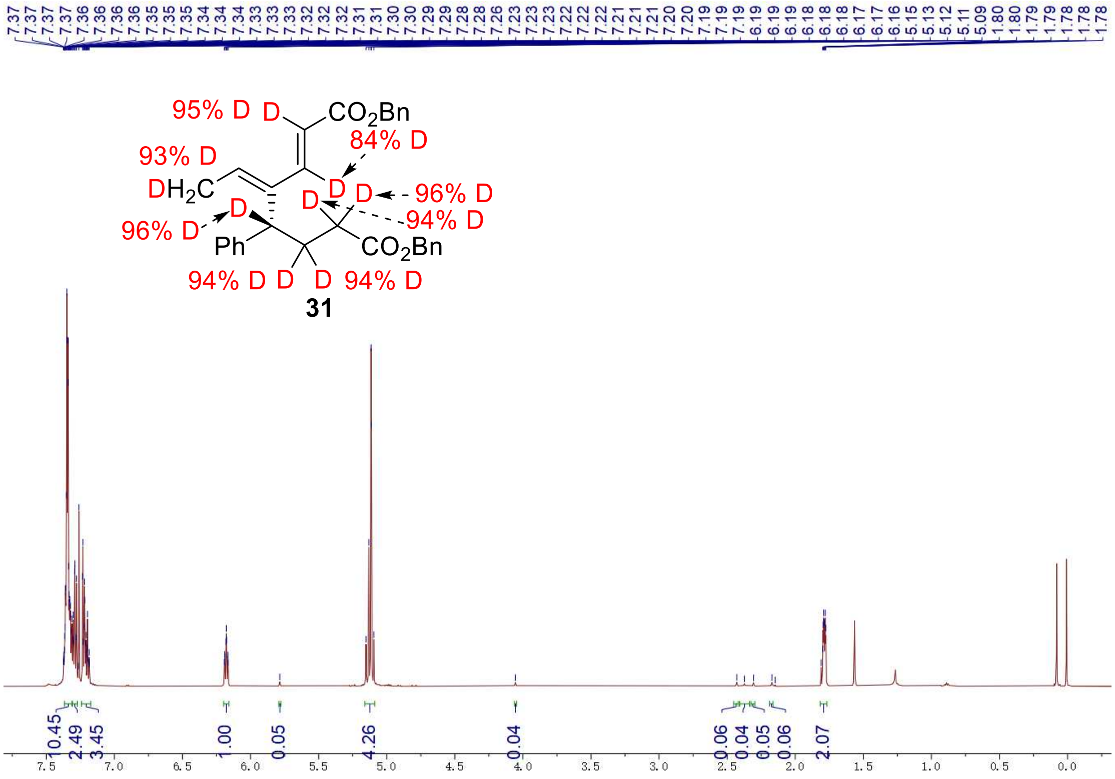

Supplementary Figure 124. ^{1}H NMR spectrum of compound of **31** in acetone-d_6

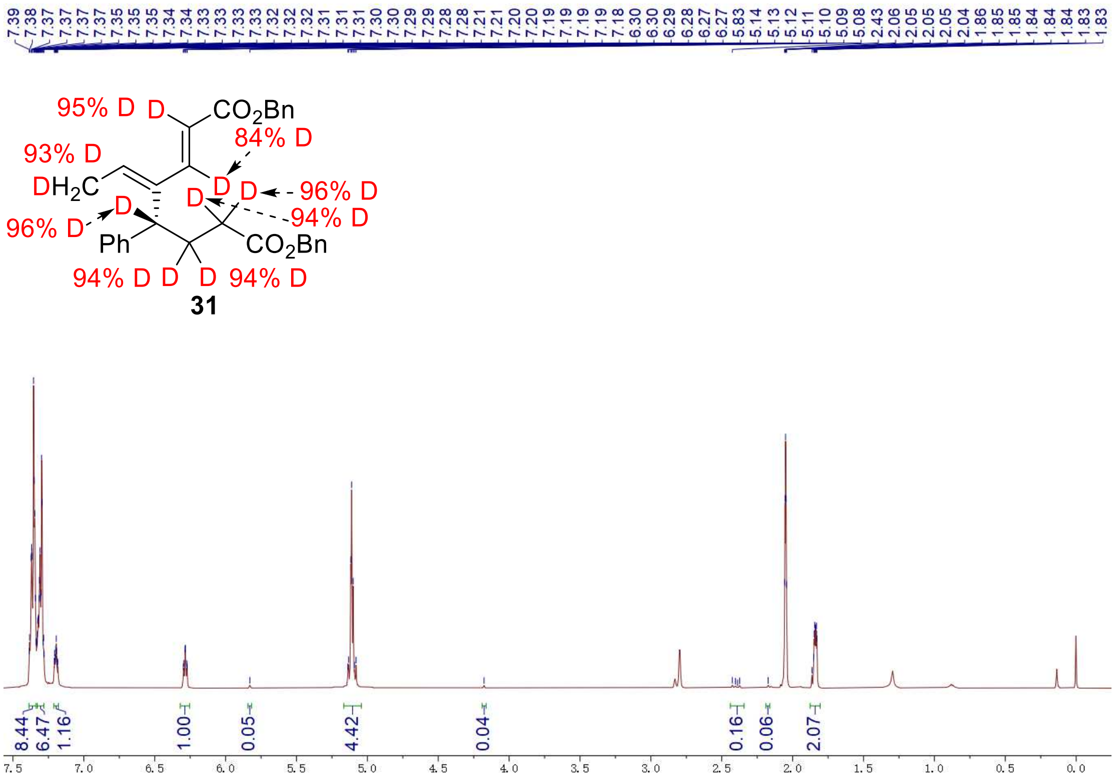

Supplementary Figure 125. ^{13}C NMR spectrum of compound of **31**

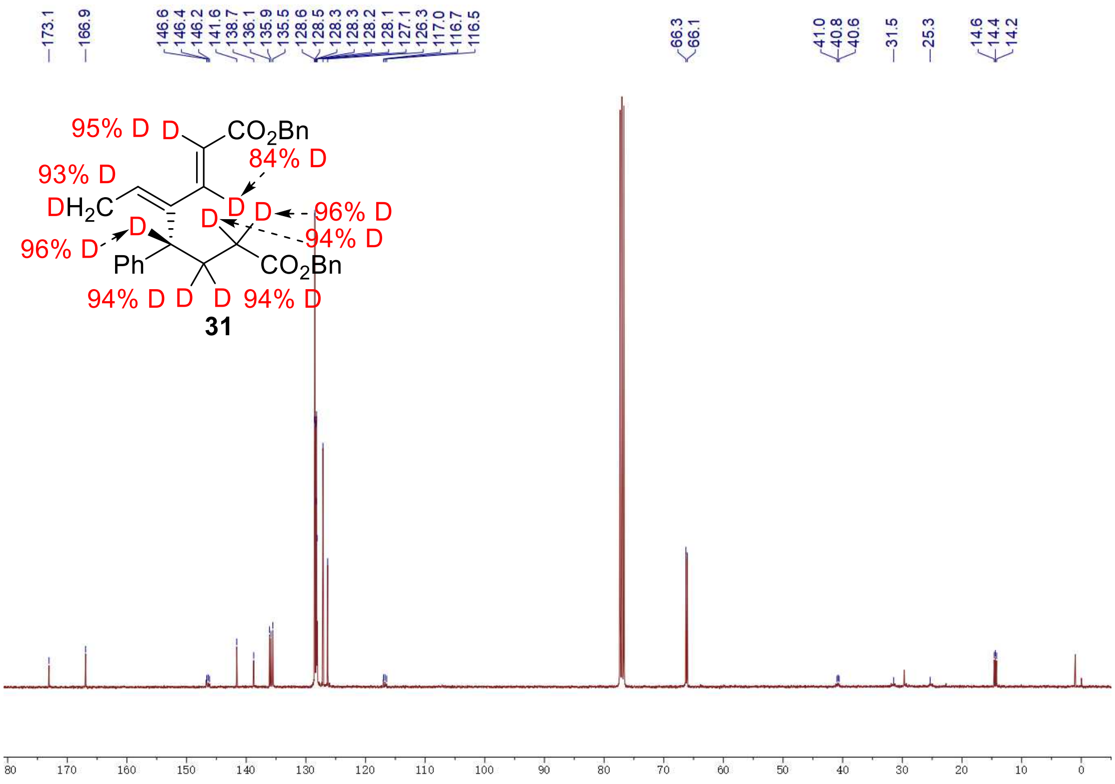

Supplementary Figure 126. ^{2}H NMR spectrum of compound of **31** in $CHCl_3$ ($CDCl_3$ as internal standard)

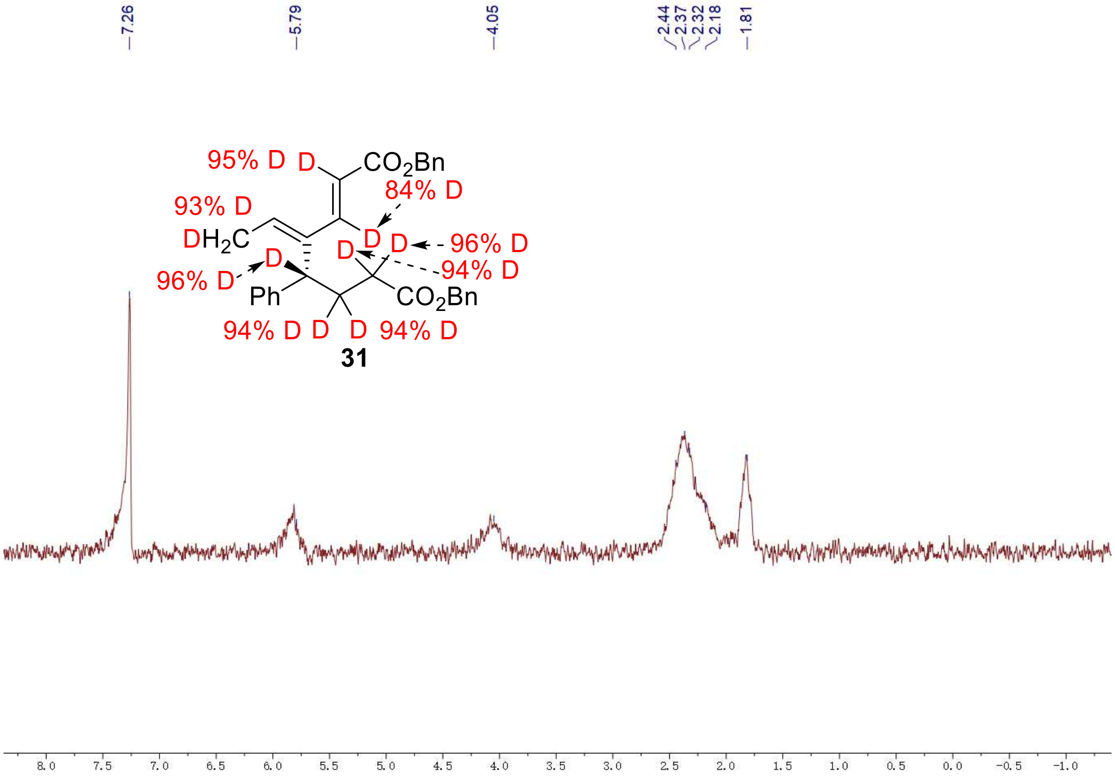

Supplementary Figure 127. ^{2}**H NMR** spectrum of compound of **31** in $CHCl_3$ (no internal standard)

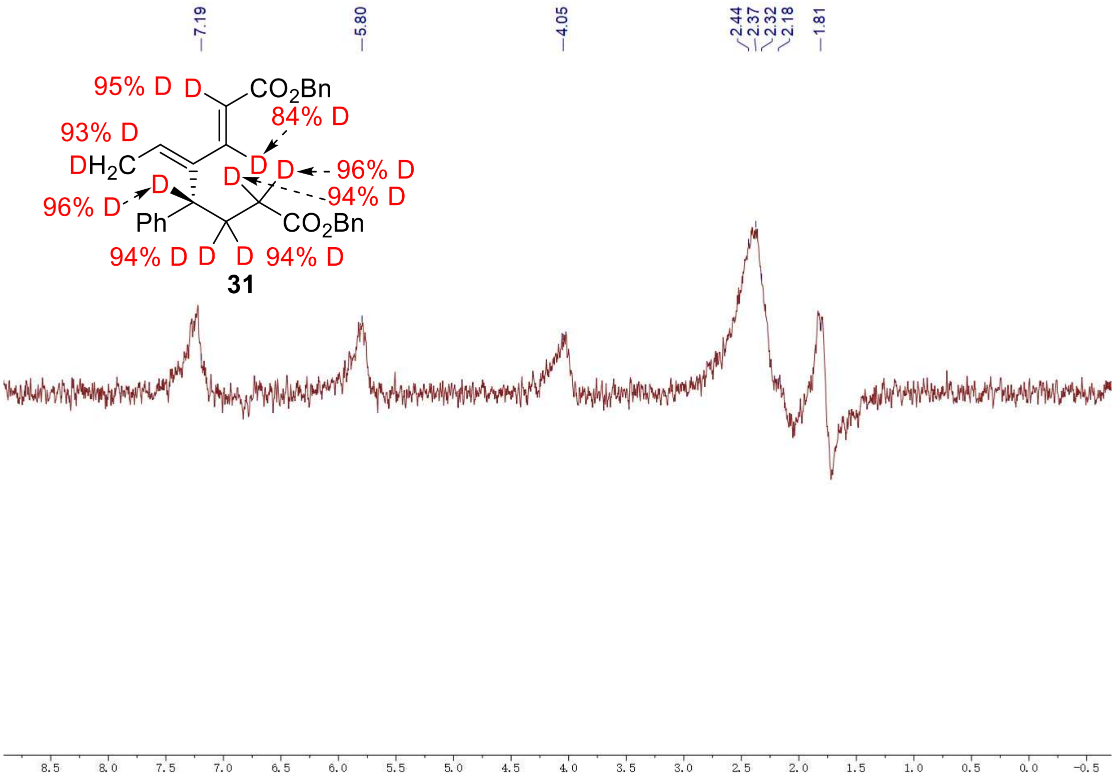

Supplementary Figure 128. ^{1}**H NMR and** ^{13}**C NMR** spectrum of compound of **32**

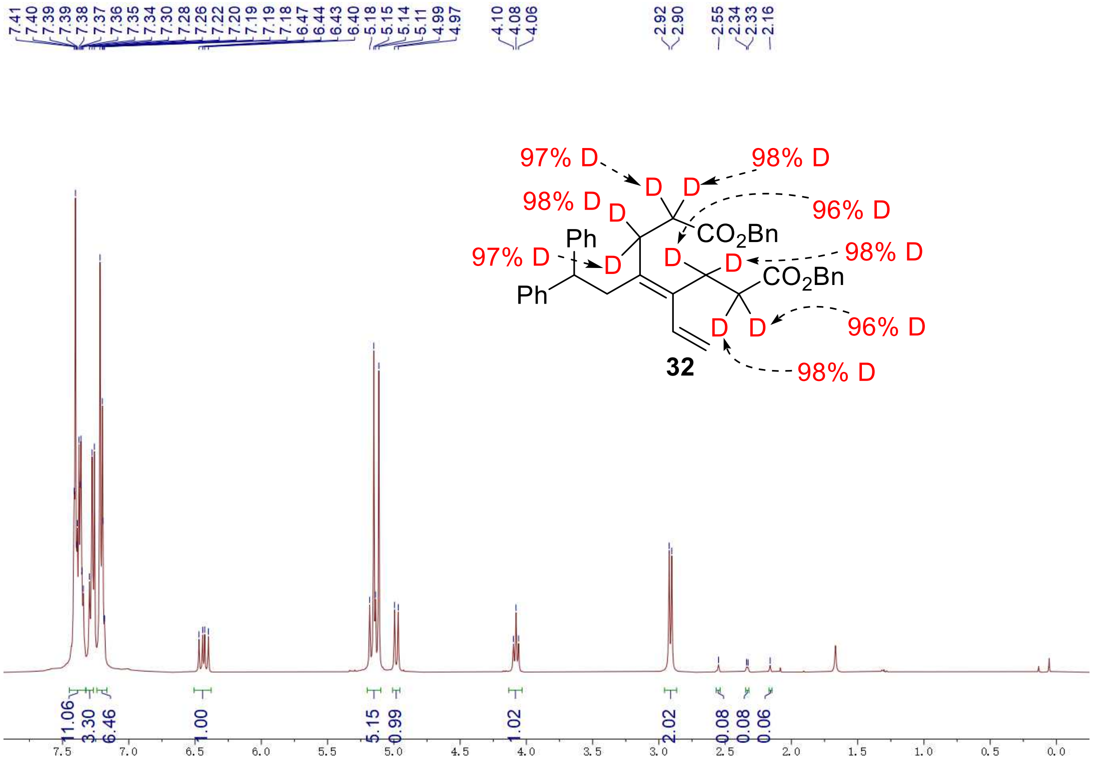

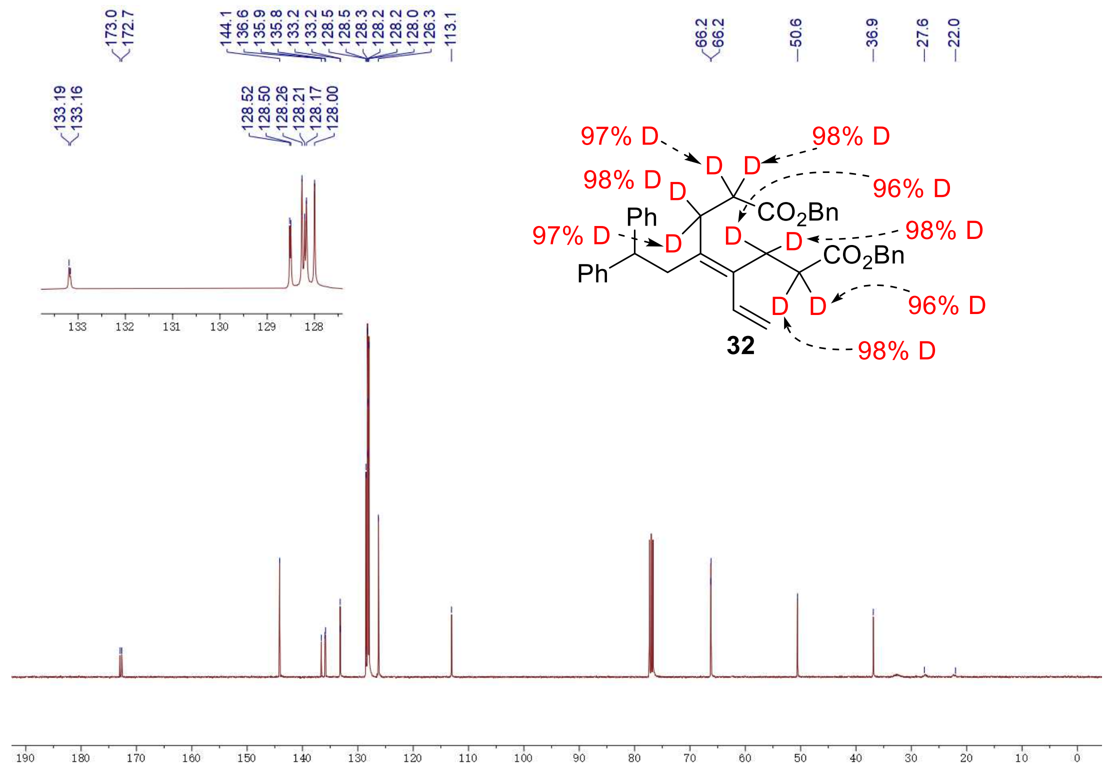

Supplementary Figure 129. ^{2}H NMR spectrum of compound of **32** in $CHCl_3$ ($CDCl_3$ as internal standard)

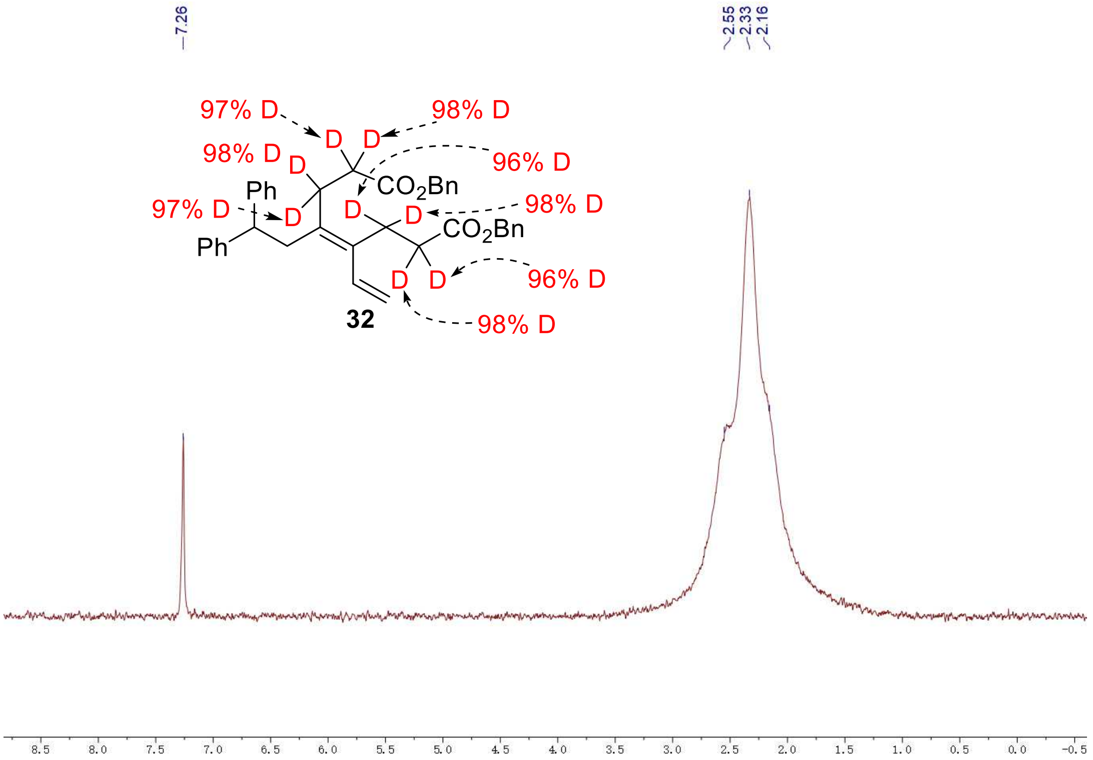

Supplementary Figure 130. ^{1}H NMR and ^{13}C NMR spectrum of compound of **33**

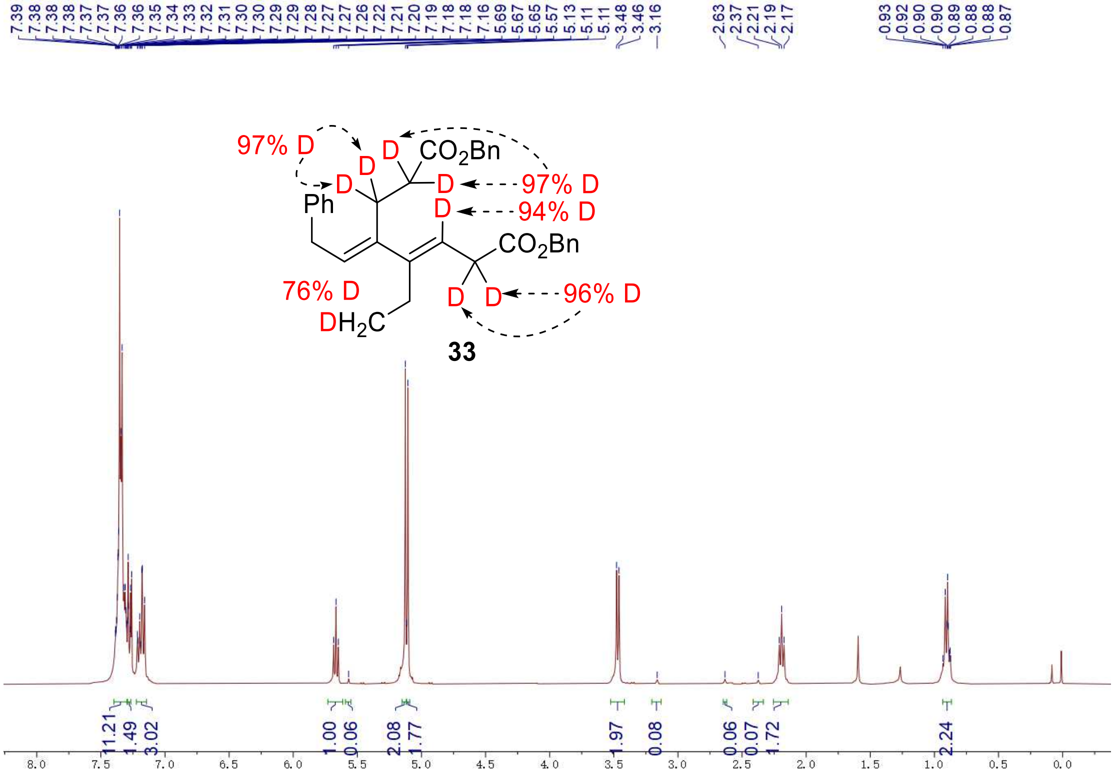

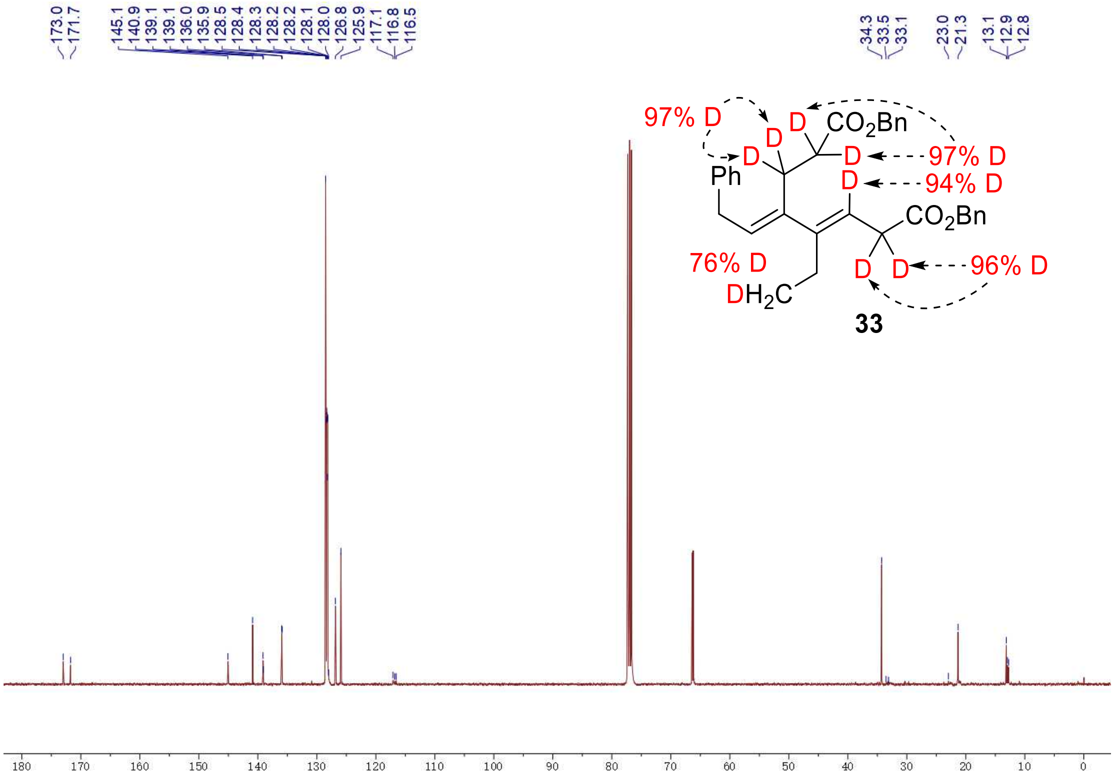

Supplementary Figure 131. ^{2}**H NMR** spectrum of compound of **33** in $CHCl_3$ ($CDCl_3$ as internal standard)

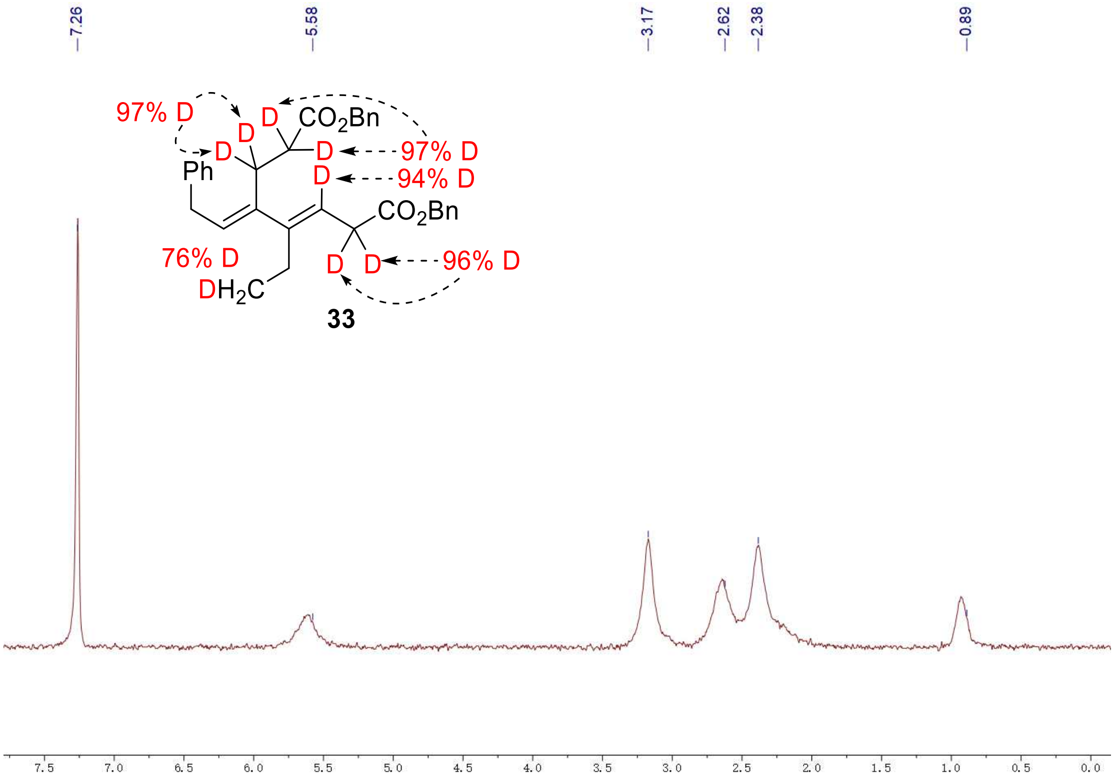

Supplementary Figure 132. ^{1}**H NMR and** ^{13}**C NMR** spectrum of compound of **36**

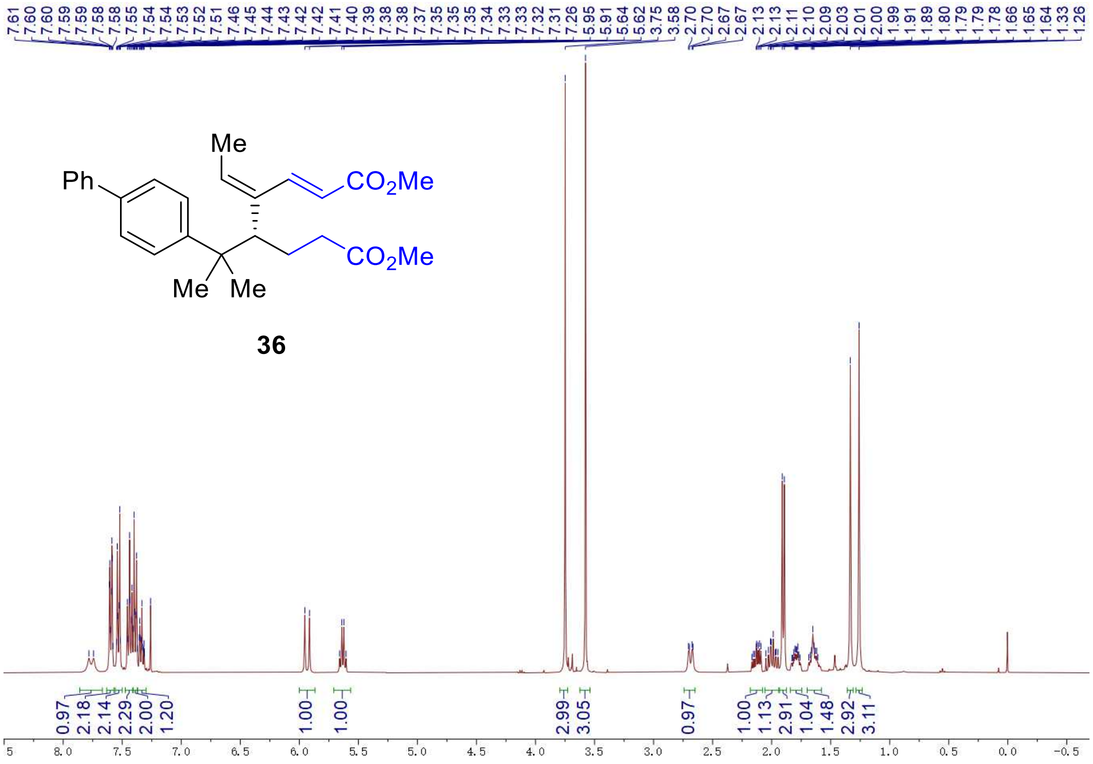

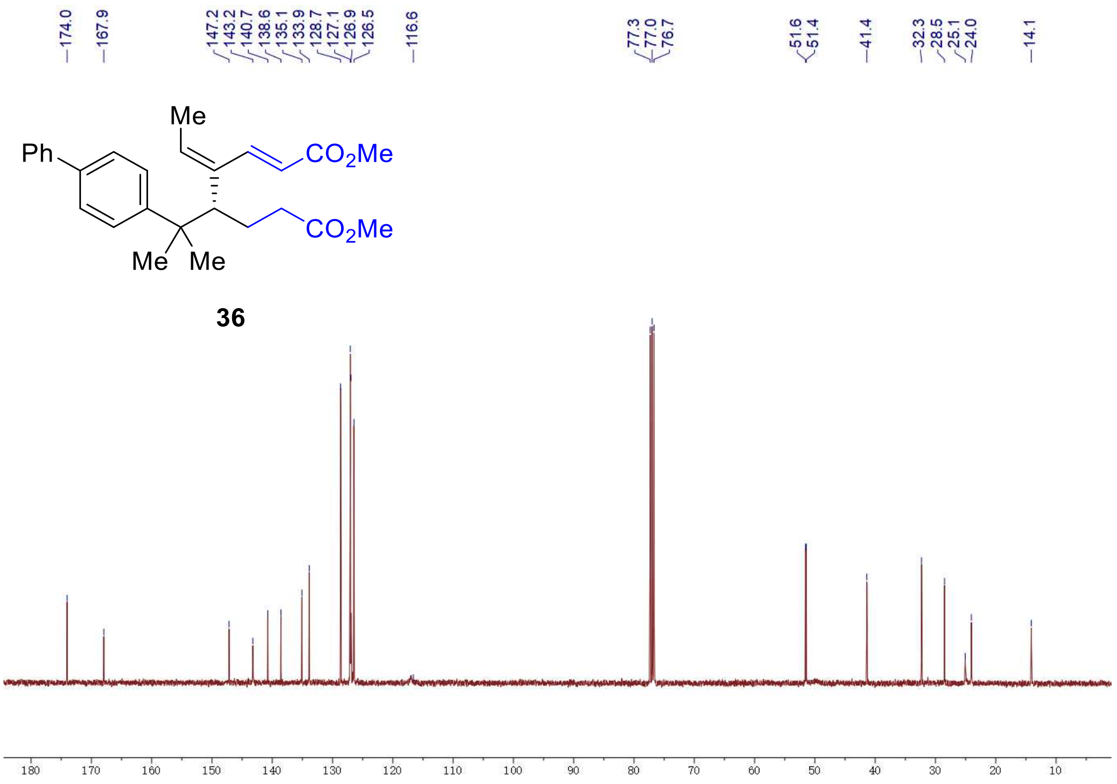

36

Supplementary Figure 133. ^{1}H NMR and ^{13}C NMR spectrum of compound of **37**

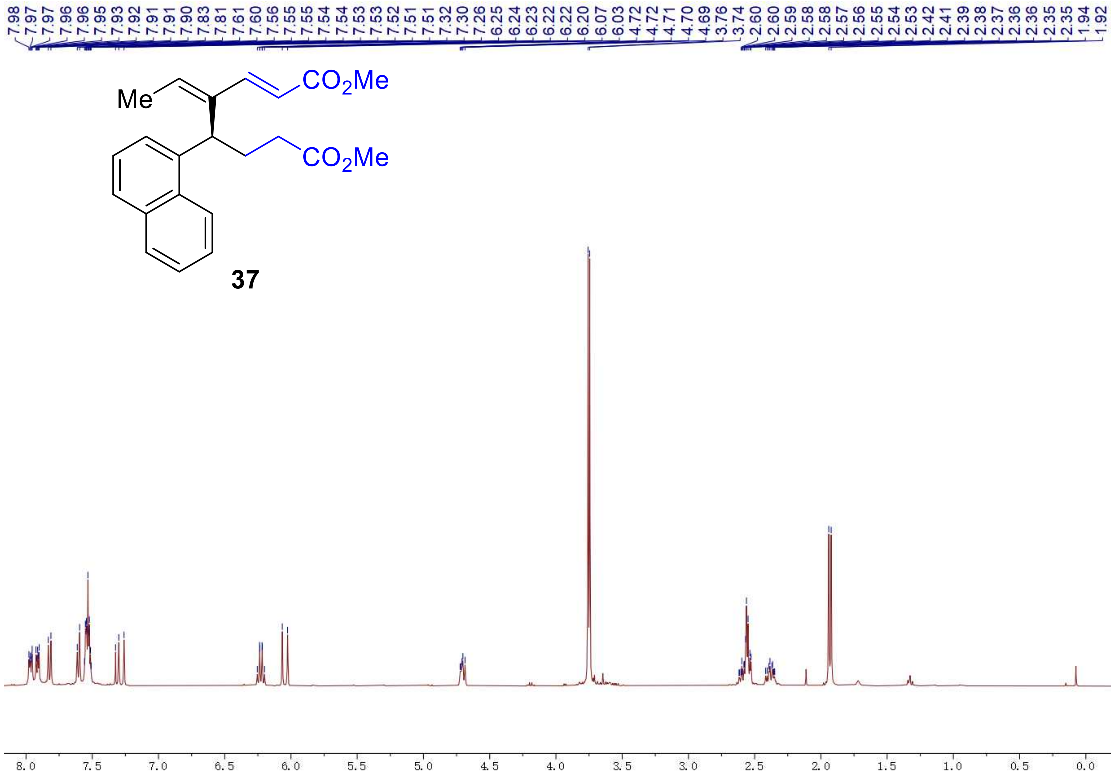

37

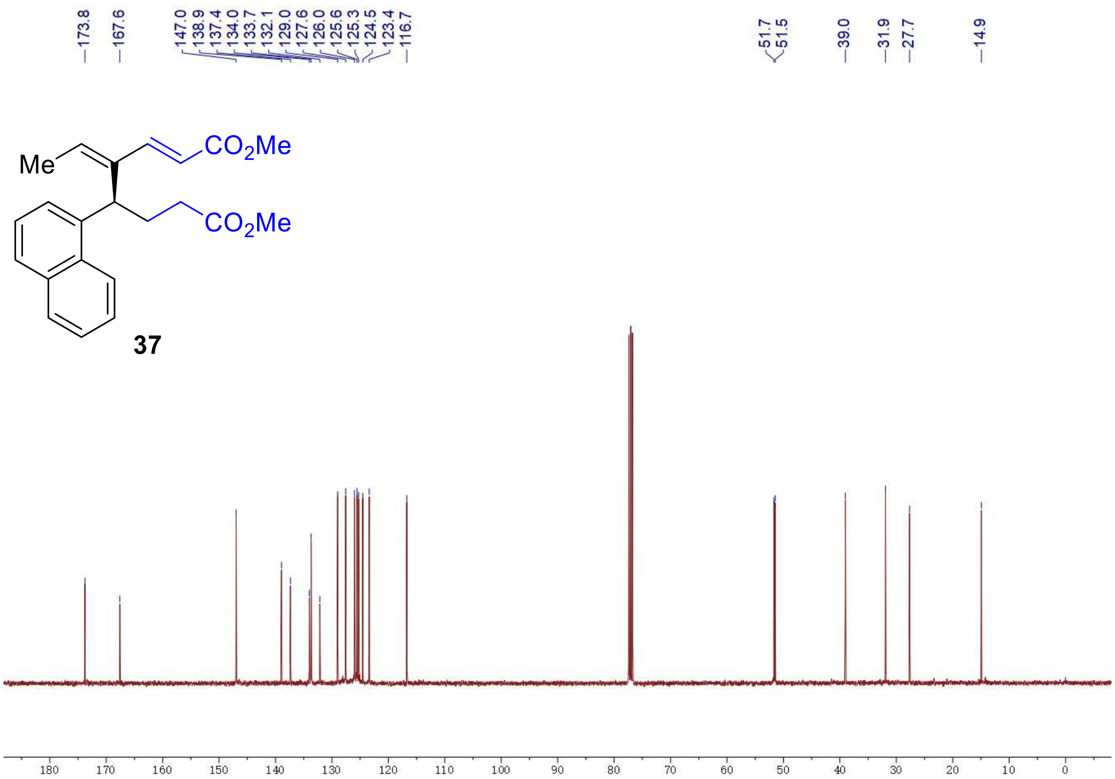

Supplementary Figure 134. ¹H NMR and ¹³C NMR spectrum of compound of **38**

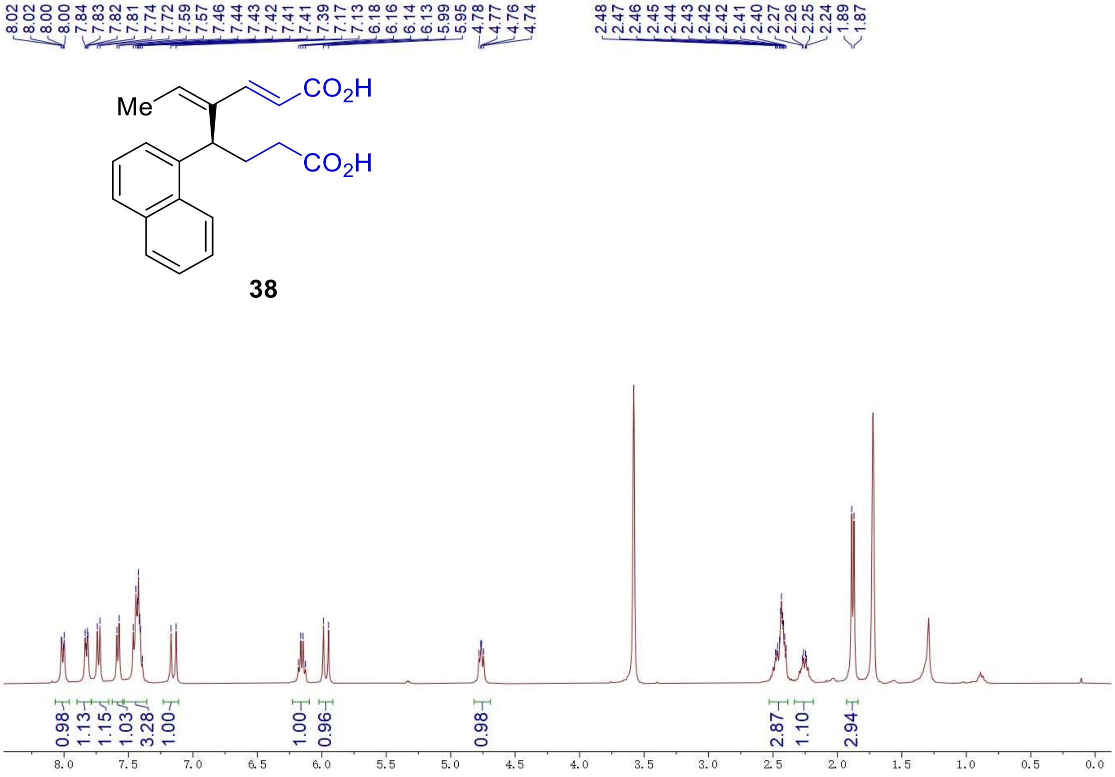

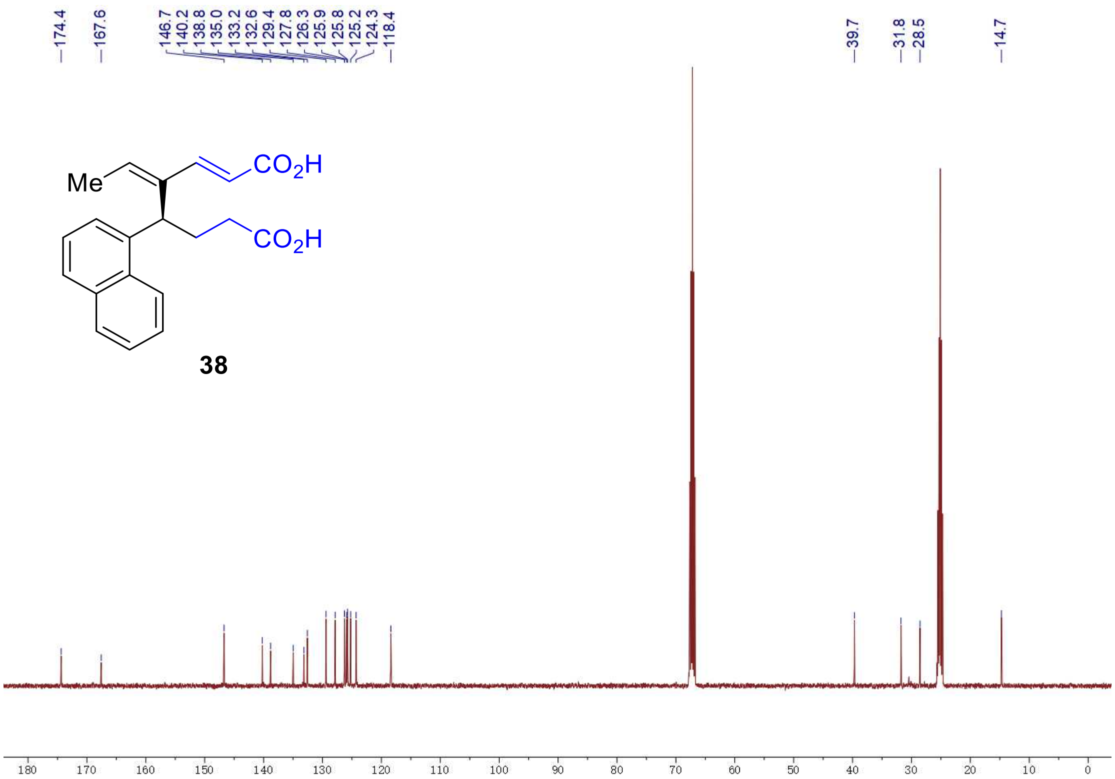

Supplementary Figure 135. ^{1}H NMR and ^{13}C NMR spectrum of compound of **39**

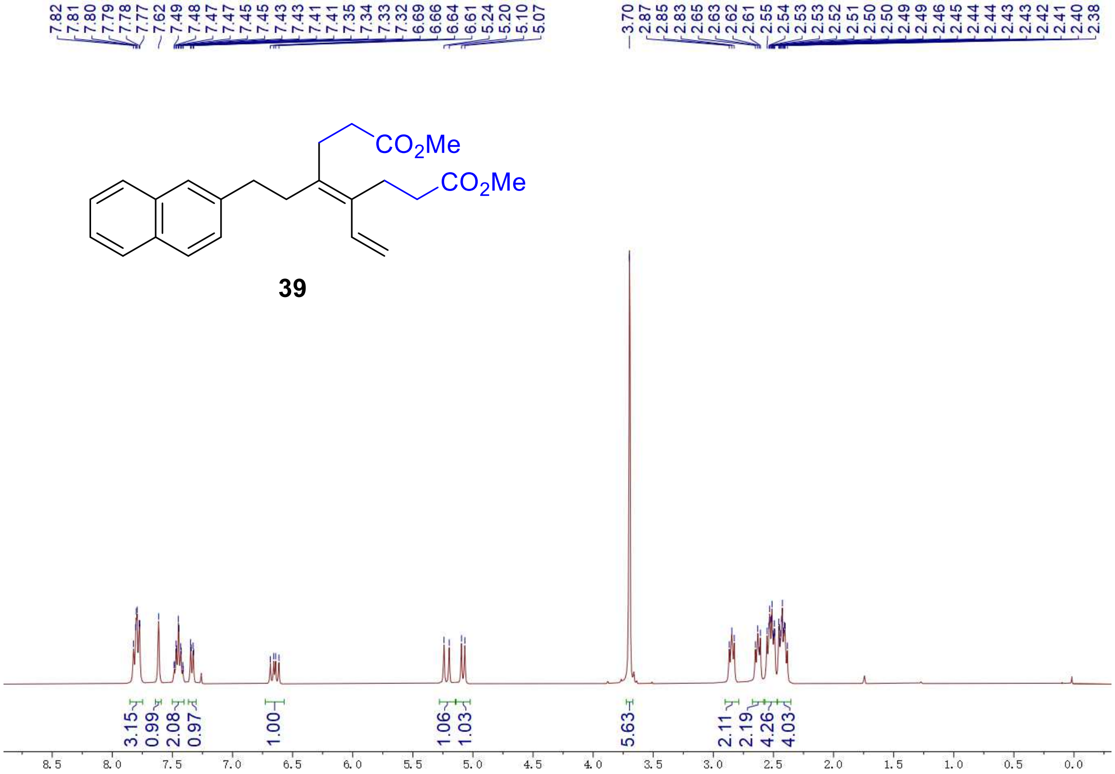

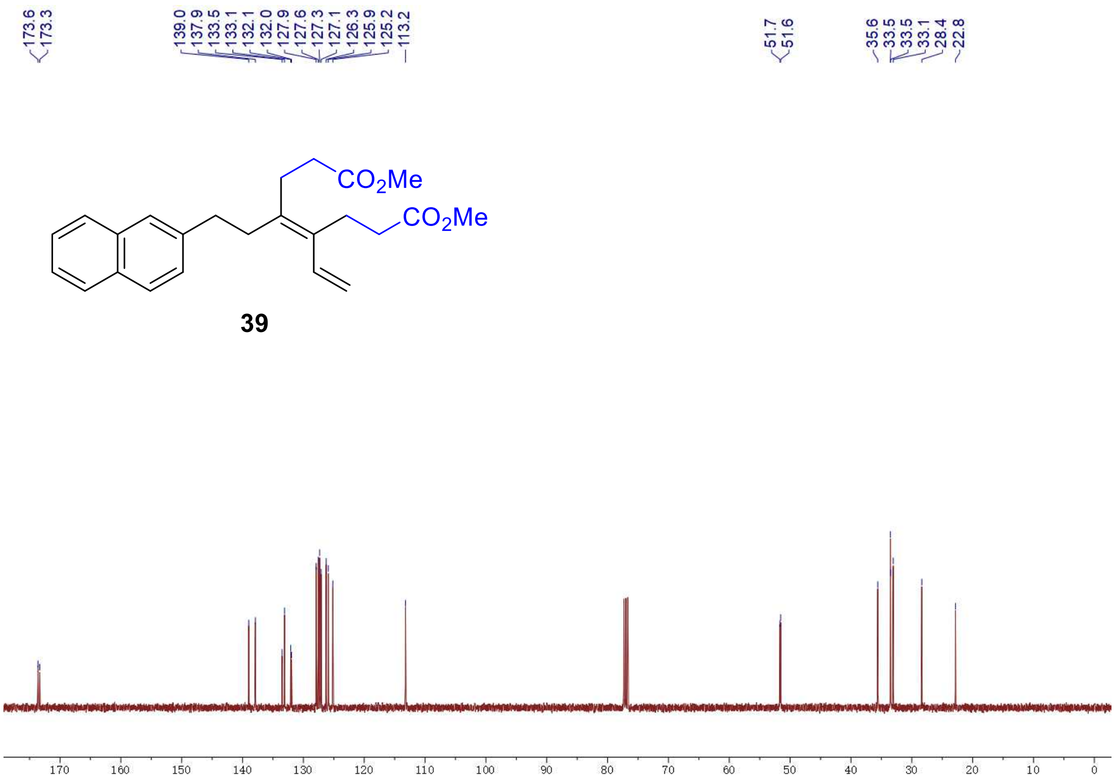

Supplementary Figure 136. ^{1}H NMR and ^{13}C NMR spectrum of compound of **40**

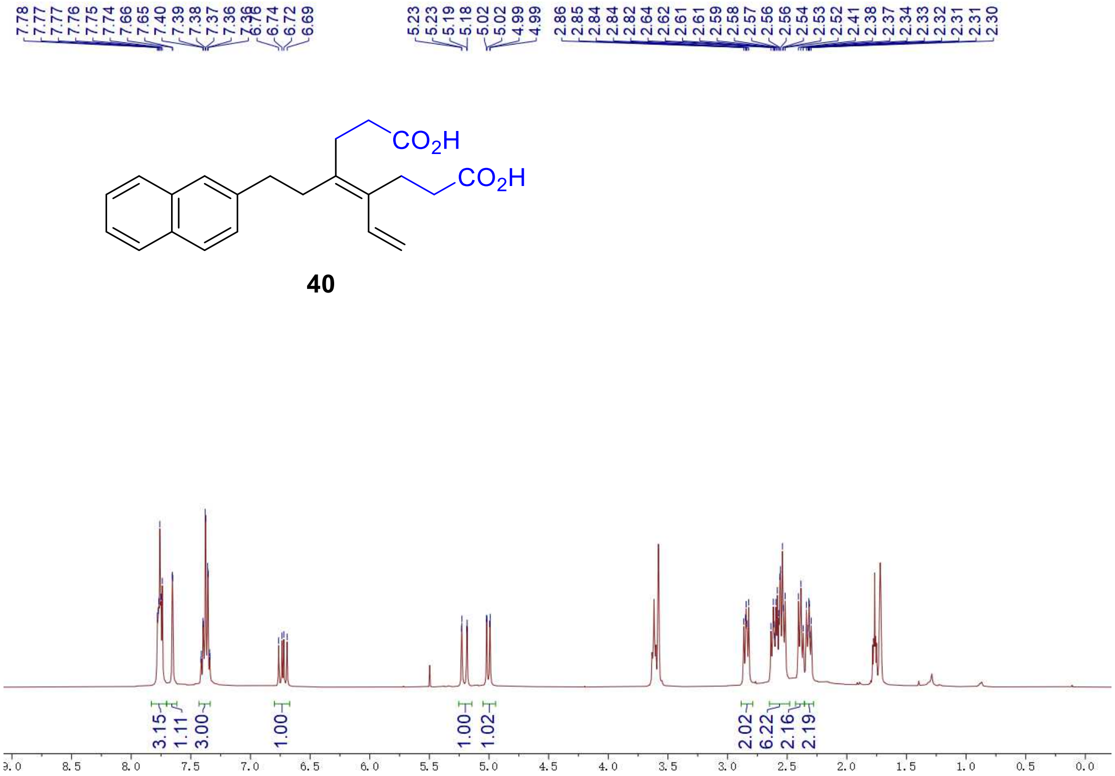

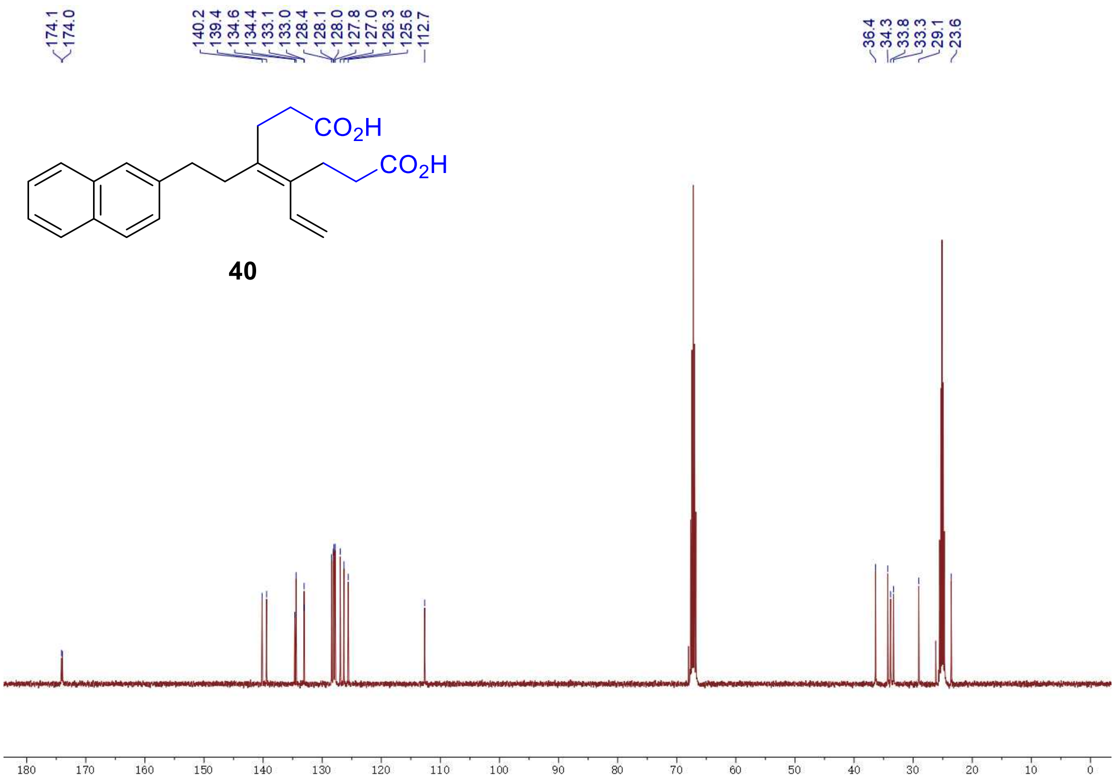

Supplementary Figure 137. ^{1}**H NMR and** ^{13}**C NMR** spectrum of compound of **41**

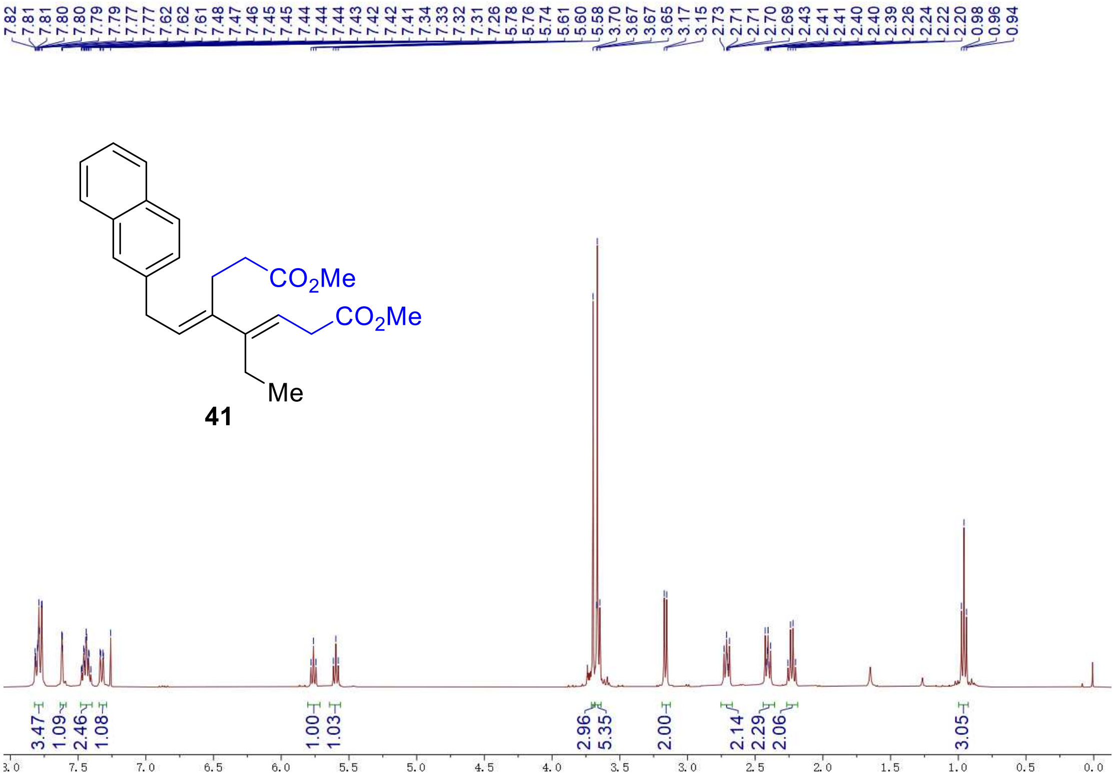

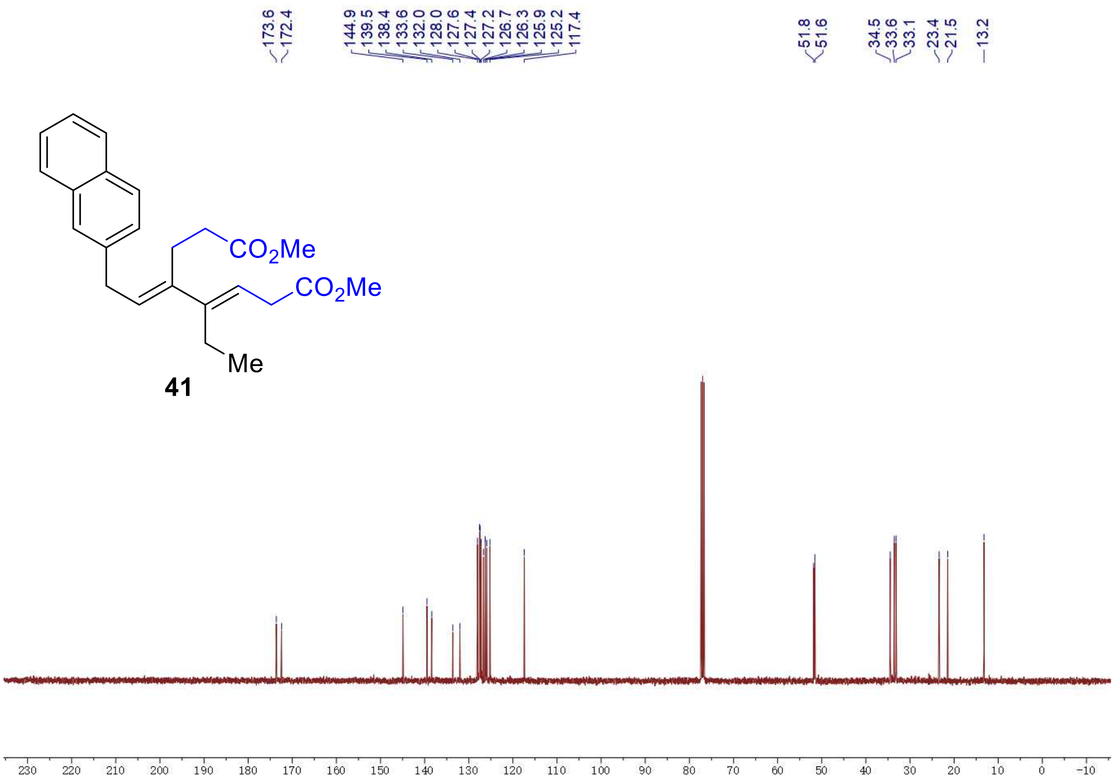

Supplementary Figure 138. ¹H NMR and ¹³C NMR spectrum of compound of **42**

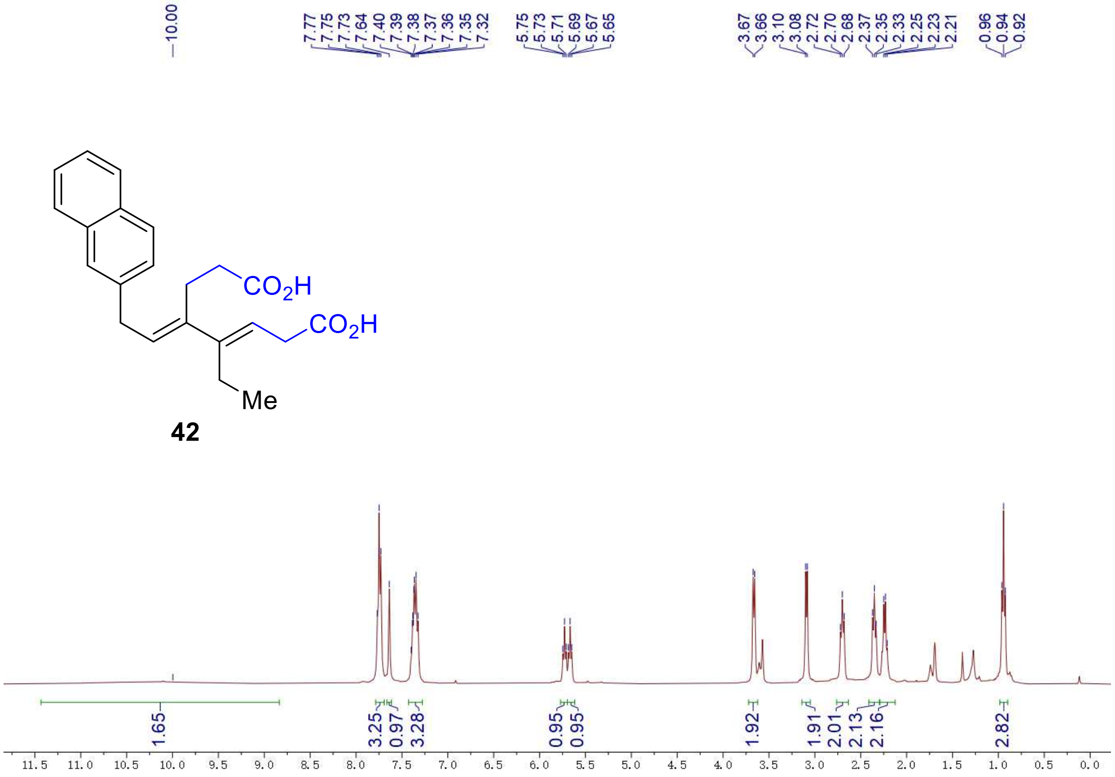

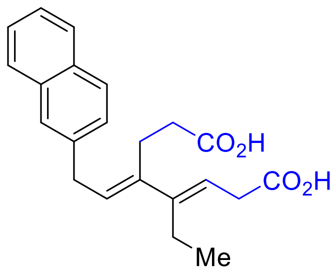

CO2H
CO2H
Me

42

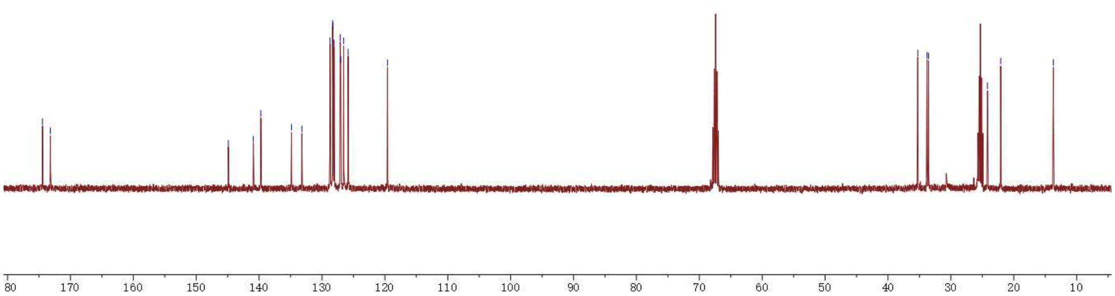

174.44
173.19
144.89
140.92
139.73
134.84
133.20
128.72
128.31
128.24
128.08
127.11
127.03
126.55
125.85
119.58
35.26
33.80
33.55
24.18
22.07
13.70

2.4 Copies of HPLC Spectra

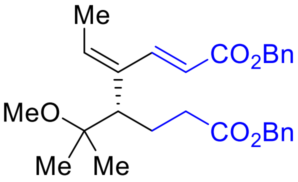

4a

Sample Name : WH-813-10-64-OZ-H-rac-7
Sample ID :
Data Filename : WH-813-10-64-OZ-H-rac-7.lcd
Method Filename : WH-5-9505-0.7-40MIN.lcm
Batch Filename : wh.lcb
Vial # : 1-61
Sample Type : Unknown
Injection Volume : 5 uL
Date Acquired : 9/21/2022 12:46:09 PM
Acquired by : System Administrator
Date Processed : 9/21/2022 1:26:11 PM
Processed by : System Administrator

<Chromatogram>

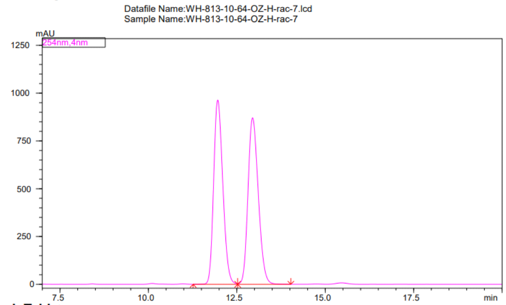

<Peak Table>

PDA Ch1 254nm

Peak#	Ret. Time	Area	Height	Area%
1	11.960	17405050	964065	49.635
2	12.945	17661308	871192	50.365
Total		35066358	1835257	100.000

Sample Name : WH-813-13-93-OZ-H-chi
Sample ID :
Data Filename : WH-813-13-93-OZ-H-chi.lcd
Method Filename : WH-5-9505-0.7-40MIN.lcm
Batch Filename : wh.lcb
Vial # : 1-62
Sample Type : Unknown
Injection Volume : 10 uL
Date Acquired : 9/21/2022 1:46:42 PM
Acquired by : System Administrator
Date Processed : 9/21/2022 2:18:37 PM
Processed by : System Administrator

<Chromatogram>

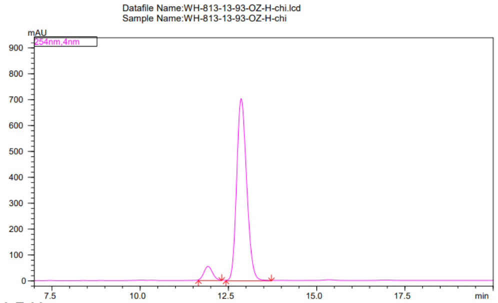

<Peak Table>

PDA Ch1 254nm

Peak#	Ret. Time	Area	Height	Area%
1	11.922	955056	54908	6.400
2	12.860	13967586	704080	93.600
Total		14922642	758988	100.000

Supplementary Figure 139. HPLC spectra of **4a**

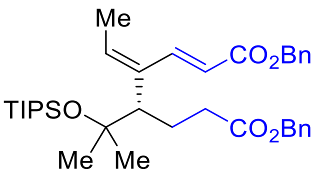

4b

Sample Name : WH-813-11-25-OZ-H-rac-4
Sample ID :
Data Filename : WH-813-11-25-OZ-H-rac-4.lcd
Method Filename : WH-5-9505-0.5-60MIN.lcm
Batch Filename : wh12.3.lcb
Vial # : 1-68
Sample Type : Unknown
Injection Volume : 5 uL
Date Acquired : 1/5/2023 10:05:43 PM
Acquired by : System Administrator
Date Processed : 1/5/2023 11:05:46 PM
Processed by : System Administrator

<Chromatogram>

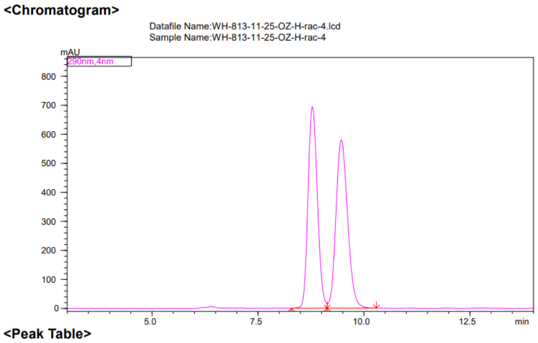

<Peak Table>

PDA Ch1 290nm

Peak#	Ret. Time	Area	Height	Area%
1	8.783	10673339	693514	49.028
2	9.465	11096758	579143	50.972
Total		21770098	1272658	100.000

Sample Name : WH-813-15-3A4-2-OZ-H-chi-1
Sample ID :
Data Filename : WH-813-15-3A4-2-OZ-H-chi-1.lcd
Method Filename : WH-5-9505-0.5-60MIN.lcm
Batch Filename : wh12.3.lcb
Vial # : 1-90
Sample Type : Unknown
Injection Volume : 5 uL
Date Acquired : 1/5/2023 11:06:18 PM
Acquired by : System Administrator
Date Processed : 1/6/2023 12:06:21 AM
Processed by : System Administrator

<Chromatogram>

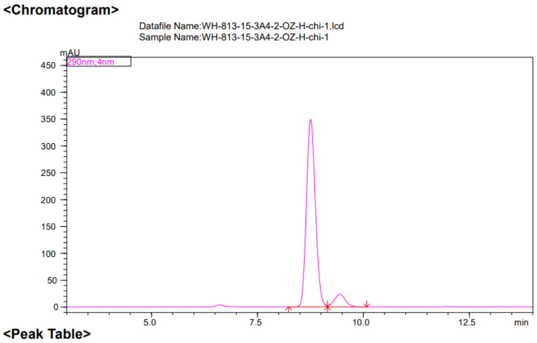

<Peak Table>

PDA Ch1 290nm

Peak#	Ret. Time	Area	Height	Area%
1	8.756	5133584	349668	92.192
2	9.438	434793	23295	7.808
Total		5568377	372963	100.000

Supplementary Figure 140. HPLC spectra of **4b**

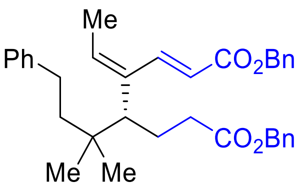

4c

Sample Name : WH-813-12-9-IG-rac-1
Sample ID :
Data Filename : WH-813-12-9-IG-rac-1.lcd
Method Filename : WH-4-9505-1.0-20MIN.lcm
Batch Filename : wh.lcb
Vial # : 1-91
Injection Volume : 10 uL
Date Acquired : 11/4/2022 5:44:25 PM
Date Processed : 11/4/2022 6:04:28 PM
Sample Type : Unknown
Acquired by : System Administrator
Processed by : System Administrator

<Chromatogram>

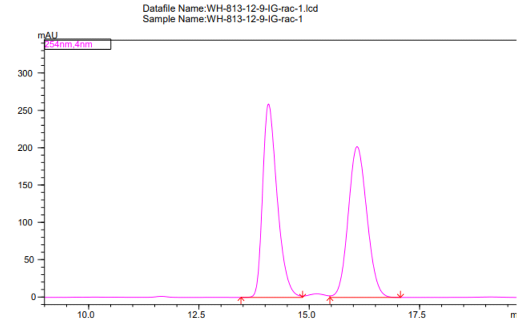

<Peak Table>

PDA Ch1 254nm

Peak#	Ret. Time	Area	Height	Area%
1	14.077	5947216	259072	50.014
2	16.084	5943986	201969	49.986
Total		11891203	461040	100.000

Sample Name : WH-813-14-28-1-IG-chi
Sample ID :
Data Filename : WH-813-14-28-1-IG-chi.lcd
Method Filename : WH-4-9505-1.0-20MIN.lcm
Batch Filename : wh.lcb
Vial # : 1-92
Injection Volume : 10 uL
Date Acquired : 11/4/2022 6:04:59 PM
Date Processed : 11/4/2022 6:25:03 PM
Sample Type : Unknown
Acquired by : System Administrator
Processed by : System Administrator

<Chromatogram>

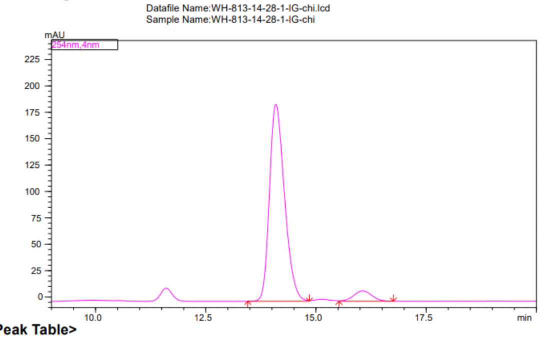

<Peak Table>

PDA Ch1 254nm

Peak#	Ret. Time	Area	Height	Area%
1	14.090	4440059	186658	94.004
2	16.058	283204	9863	5.996
Total		4723264	196520	100.000

Supplementary Figure 141. HPLC spectra of **4c**

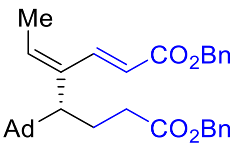

4d

Sample Name	: WH-813-11-92-3-OZ-H-rac-4		
Sample ID	:		
Data Filename	: WH-813-11-92-3-OZ-H-rac-4.lcd		
Method Filename	: WH-1-9505-0.7-40MIN.lcm		
Batch Filename	: wh.lcb		
Vial #	: 1-93	Sample Type	: Unknown
Injection Volume	: 10 uL		
Date Acquired	: 10/31/2022 6:08:46 PM	Acquired by	: System Administrator
Date Processed	: 10/31/2022 6:48:48 PM	Processed by	: System Administrator

<Chromatogram>

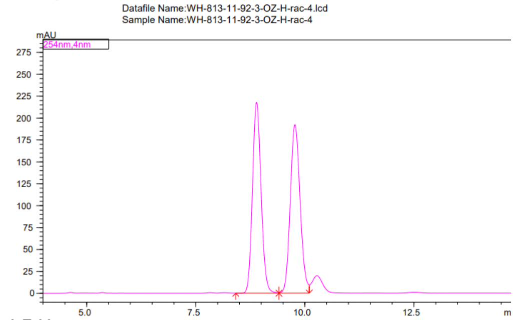

<Peak Table>

PDA Ch1 254nm

Peak#	Ret. Time	Area	Height	Area%
1	8.893	2997011	217749	49.835
2	9.773	3016802	192203	50.165
Total		6013813	409952	100.000

Sample Name	: WH-813-14-26-1-OZ-H-chi-1		
Sample ID	:		
Data Filename	: WH-813-14-26-1-OZ-H-chi-1.lcd		
Method Filename	: WH-1-9505-0.7-40MIN.lcm		
Batch Filename	: wh.lcb		
Vial #	: 1-94	Sample Type	: Unknown
Injection Volume	: 10 uL		
Date Acquired	: 10/31/2022 6:49:18 PM	Acquired by	: System Administrator
Date Processed	: 10/31/2022 7:19:37 PM	Processed by	: System Administrator

<Chromatogram>

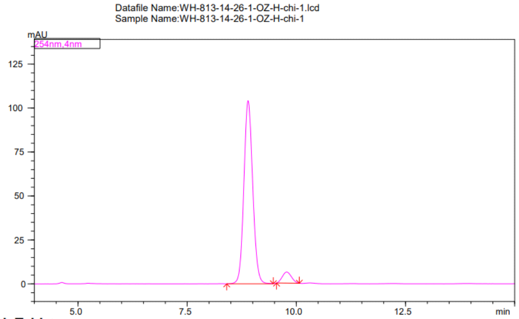

<Peak Table>

PDA Ch1 254nm

Peak#	Ret. Time	Area	Height	Area%
1	8.895	1473534	104135	94.165
2	9.778	91315	6292	5.835
Total		1564849	110427	100.000

Supplementary Figure 142. HPLC spectra of **4d**

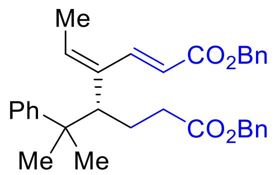

4e

Sample Name	: WH-813-11-23-OZ-H-rac-2		
Sample ID	:		
Data Filename	: WH-813-11-23-OZ-H-rac-2.lcd		
Method Filename	: WH-5-9505-0.7-30MIN.lcm		
Batch Filename	: wh12.3.lcb		
Vial #	: 1-67	Sample Type	: Unknown
Injection Volume	: 10 uL		
Date Acquired	: 12/4/2022 8:43:07 PM	Acquired by	: System Administrator
Date Processed	: 12/4/2022 9:13:11 PM	Processed by	: System Administrator

<Chromatogram>

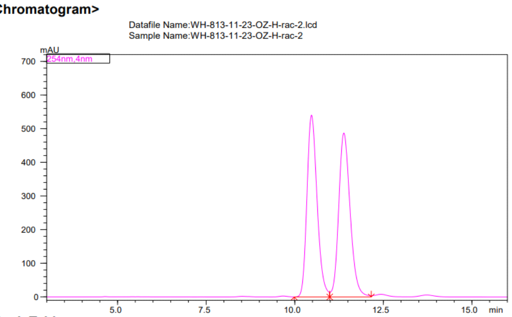

<Peak Table>

PDA Ch1 254nm

Peak#	Ret. Time	Area	Height	Area%
1	10.473	10785226	540582	49.686
2	11.386	10921531	487257	50.314
Total		21706756	1027840	100.000

Sample Name	: WH-813-14-96-OZ-H-CHI		
Sample ID	:		
Data Filename	: WH-813-14-96-OZ-H-CHI.lcd		
Method Filename	: WH-5-9505-0.7-30MIN.lcm		
Batch Filename	: wh12.3.lcb		
Vial #	: 1-68	Sample Type	: Unknown
Injection Volume	: 10 uL		
Date Acquired	: 12/4/2022 9:13:42 PM	Acquired by	: System Administrator
Date Processed	: 12/4/2022 9:43:44 PM	Processed by	: System Administrator

<Chromatogram>

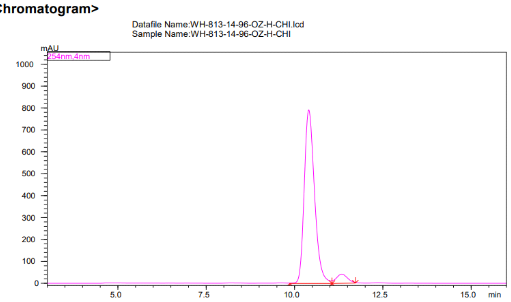

<Peak Table>

PDA Ch1 254nm

Peak#	Ret. Time	Area	Height	Area%
1	10.403	16070429	792228	94.517
2	11.338	932217	42050	5.483
Total		17002646	834278	100.000

Supplementary Figure 143. HPLC spectra of **4e**

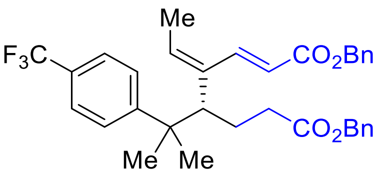

4f

Sample Name	: WH-813-12-16D2-OZ-H-rac-2
Sample ID	:
Data Filename	: WH-813-12-16D2-OZ-H-rac-2.lcd
Method Filename	: WH-5-9703-0.5-60MIN.lcm
Batch Filename	: wh12.3.lcb
Vial #	: 1-66
Sample Type	: Unknown
Injection Volume	: 10 uL
Date Acquired	: 1/5/2023 7:34:06 PM
Acquired by	: System Administrator
Date Processed	: 1/5/2023 8:34:09 PM
Processed by	: System Administrator

<Chromatogram>

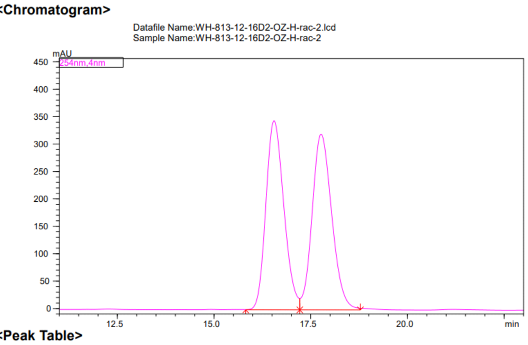

<Peak Table>

PDA Ch1 254nm

Peak#	Ret. Time	Area	Height	Area%
1	16.551	11157314	344879	49.607
2	17.767	11333901	320505	50.393
Total		22491215	665384	100.000

Sample Name	: WH-813-15-3A1-OZ-H-chi-1
Sample ID	:
Data Filename	: WH-813-15-3A1-OZ-H-chi-1.lcd
Method Filename	: WH-5-9703-0.5-60MIN.lcm
Batch Filename	: wh12.3.lcb
Vial #	: 1-74
Sample Type	: Unknown
Injection Volume	: 5 uL
Date Acquired	: 1/5/2023 8:34:41 PM
Acquired by	: System Administrator
Date Processed	: 1/5/2023 9:34:44 PM
Processed by	: System Administrator

<Chromatogram>

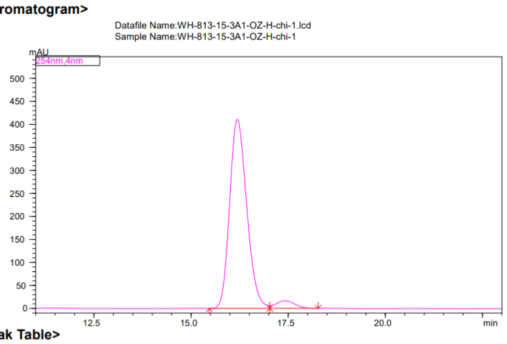

<Peak Table>

PDA Ch1 254nm

Peak#	Ret. Time	Area	Height	Area%
1	16.189	12590145	410818	95.792
2	17.419	553078	16504	4.208
Total		13143224	427323	100.000

Supplementary Figure 144. HPLC spectra of **4f**

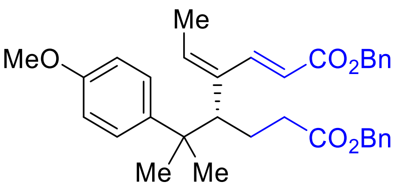

4g

Sample Name	: WH-813-12-14D2-2-OZ-H-rac-4
Sample ID	:
Data Filename	: WH-813-12-14D2-2-OZ-H-rac-4.lcd
Method Filename	: WH-2-9505-0.5-50MIN.lcm
Batch Filename	: wh12.3.lcb
Vial #	: 1-66
Sample Type	: Unknown
Injection Volume	: 10 uL
Date Acquired	: 12/22/2022 9:47:12 AM
Acquired by	: System Administrator
Date Processed	: 12/22/2022 10:37:15 AM
Processed by	: System Administrator

<Chromatogram>

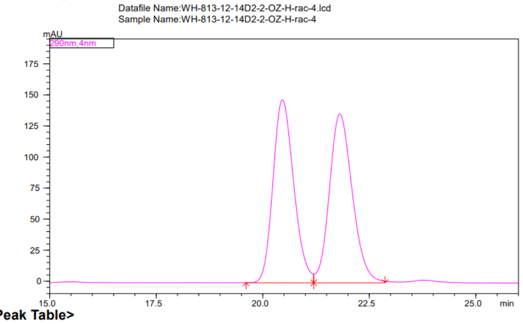

<Peak Table>

PDA Ch1 290nm

Peak#	Ret. Time	Area	Height	Area%
1	20.460	4991079	147420	49.521
2	21.808	5087723	135984	50.479
Total		10078802	283404	100.000

Sample Name	: WH-813-14-97A2-OZ-H-chi-2
Sample ID	:
Data Filename	: WH-813-14-97A2-OZ-H-chi-2.lcd
Method Filename	: WH-2-9505-0.5-50MIN.lcm
Batch Filename	: wh12.3.lcb
Vial #	: 1-67
Sample Type	: Unknown
Injection Volume	: 10 uL
Date Acquired	: 12/22/2022 10:37:47 AM
Acquired by	: System Administrator
Date Processed	: 12/22/2022 11:27:51 AM
Processed by	: System Administrator

<Chromatogram>

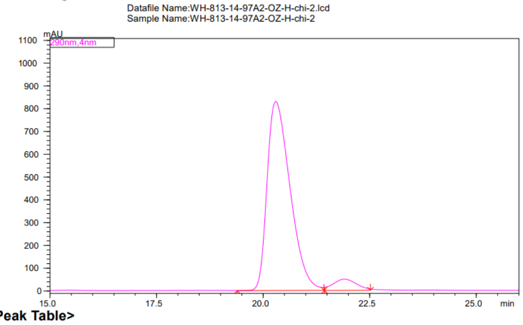

<Peak Table>

PDA Ch1 290nm

Peak#	Ret. Time	Area	Height	Area%
1	20.296	31130611	829715	94.267
2	21.909	1893105	49418	5.733
Total		33023716	879133	100.000

Supplementary Figure 145. HPLC spectra of **4g**

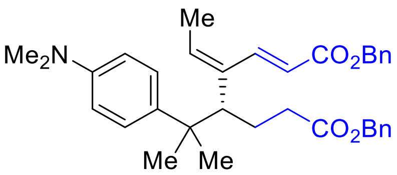

4h

Sample Name	: WH-813-12-17D2-2-IA-rac
Sample ID	:
Data Filename	: WH-813-12-17D2-2-IA-rac.lcd
Method Filename	: WH-2-9505-0.7-30MIN.lcm
Batch Filename	: wh.lcb
Vial #	: 1-69
Sample Type	: Unknown
Injection Volume	: 10 uL
Date Acquired	: 1/12/2022 5:26:18 PM
Acquired by	: System Administrator
Date Processed	: 1/12/2022 5:56:21 PM
Processed by	: System Administrator

<Chromatogram>

Datafile Name:WH-813-12-17D2-2-IA-rac.lcd
Sample Name:WH-813-12-17D2-2-IA-rac

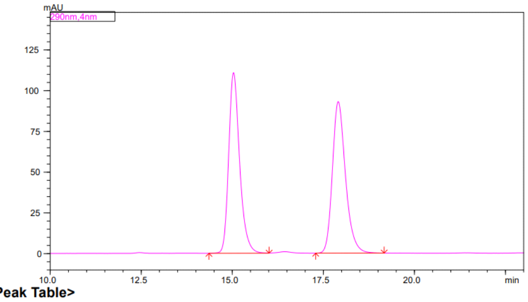

<Peak Table>

PDA Ch1 290nm

Peak#	Ret. Time	Area	Height	Area%
1	15.033	2316523	110869	49.862
2	17.908	2329340	93050	50.138
Total		4645863	203920	100.000

Sample Name	: WH-813-14-97A1-IA-CHI
Sample ID	:
Data Filename	: WH-813-14-97A1-IA-CHI.lcd
Method Filename	: WH-2-9505-0.7-30MIN.lcm
Batch Filename	: wh12.3.lcb
Vial #	: 1-65
Sample Type	: Unknown
Injection Volume	: 10 uL
Date Acquired	: 12/9/2022 9:00:55 PM
Acquired by	: System Administrator
Date Processed	: 12/9/2022 9:30:58 PM
Processed by	: System Administrator

<Chromatogram>

Datafile Name:WH-813-14-97A1-IA-CHI.lcd
Sample Name:WH-813-14-97A1-IA-CHI

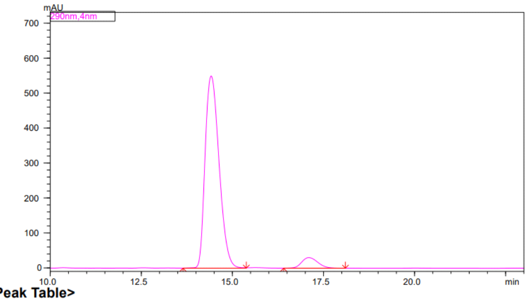

<Peak Table>

PDA Ch1 290nm

Peak#	Ret. Time	Area	Height	Area%
1	14.418	14654967	549647	94.022
2	17.097	931843	30147	5.978
Total		15586810	579794	100.000

Supplementary Figure 146. HPLC spectra of **4h**

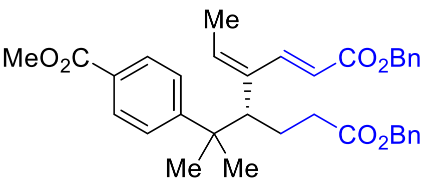

4i

Sample Name : WH-813-12-23D1-IA-rac-3
Sample ID :
Data Filename : WH-813-12-23D1-IA-rac-3.lcd
Method Filename : WH-2-9505-0.7-60MIN.lcm
Batch Filename : wh.lcb
Vial # : 1-91
Sample Type : Unknown
Injection Volume : 25 uL
Date Acquired : 11/12/2022 3:01:22 PM
Acquired by : System Administrator
Date Processed : 11/12/2022 4:01:24 PM
Processed by : System Administrator

<Chromatogram>

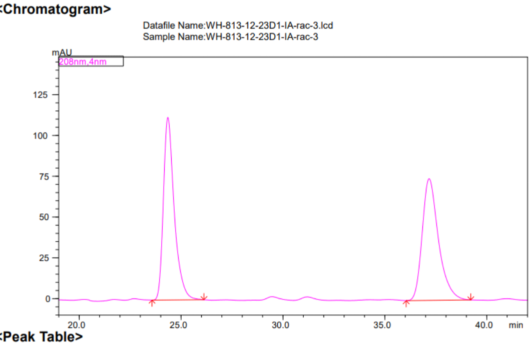

<Peak Table>

PDA Ch1 208nm

Peak#	Ret. Time	Area	Height	Area%
1	24.352	4155212	111840	50.073
2	37.166	4143096	74532	49.927
Total		8298308	186372	100.000

Sample Name : WH-813-14-69-IA-CHI-1
Sample ID :
Data Filename : WH-813-14-69-IA-CHI-1.lcd
Method Filename : WH-2-9505-0.7-60MIN.lcm
Batch Filename : wh.lcb
Vial # : 1-93
Sample Type : Unknown
Injection Volume : 25 uL
Date Acquired : 11/12/2022 4:01:55 PM
Acquired by : System Administrator
Date Processed : 11/12/2022 5:01:58 PM
Processed by : System Administrator

<Chromatogram>

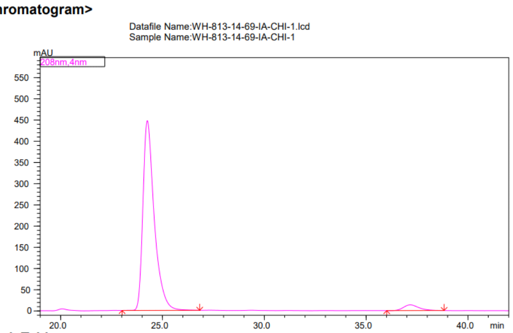

<Peak Table>

PDA Ch1 208nm

Peak#	Ret. Time	Area	Height	Area%
1	24.257	17329391	447150	96.015
2	37.160	719186	13510	3.985
Total		18048577	460660	100.000

Supplementary Figure 147. HPLC spectra of **4i**

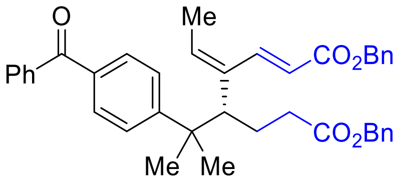

4j

Sample Name : WH-813-12-42D2-IB-rac-1
Sample ID :
Data Filename : WH-813-12-42D2-IB-rac-1.lcd
Method Filename : WH-3-9208-1.0-60MIN.lcm
Batch Filename : wh12.3.lcb
Vial # : 1-65
Injection Volume : 20 uL
Date Acquired : 1/4/2023 1:20:02 PM
Date Processed : 1/4/2023 2:20:05 PM
Sample Type : Unknown
Acquired by : System Administrator
Processed by : System Administrator

<Chromatogram>

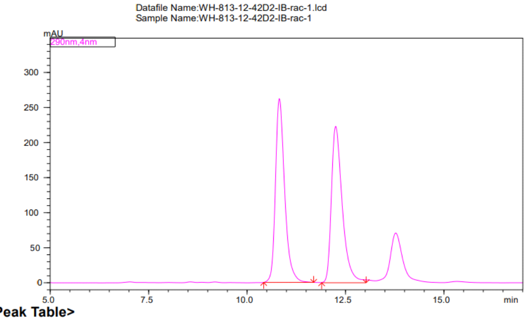

<Peak Table>

PDA Ch1 290nm

Peak#	Ret. Time	Area	Height	Area%
1	10.819	4273642	262163	49.822
2	12.250	4304159	223056	50.178
Total		8577801	485219	100.000

Sample Name : WH-813-15-1A4-IB-CHI
Sample ID :
Data Filename : WH-813-15-1A4-IB-CHI.lcd
Method Filename : WH-3-9208-1.0-60MIN.lcm
Batch Filename : wh12.3.lcb
Vial # : 1-73
Injection Volume : 20 uL
Date Acquired : 1/4/2023 2:20:37 PM
Date Processed : 1/4/2023 3:20:40 PM
Sample Type : Unknown
Acquired by : System Administrator
Processed by : System Administrator

<Chromatogram>

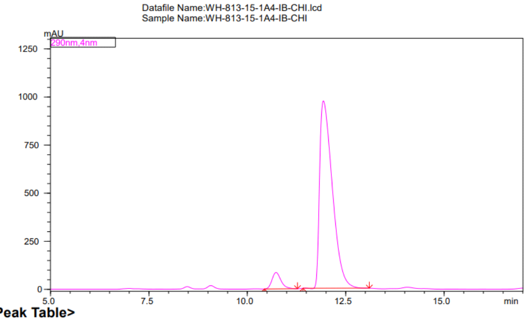

<Peak Table>

PDA Ch1 290nm

Peak#	Ret. Time	Area	Height	Area%
1	10.730	1358743	85389	5.847
2	11.933	21880707	973622	94.153
Total		23239450	1059011	100.000

Supplementary Figure 148. HPLC spectra of **4j**

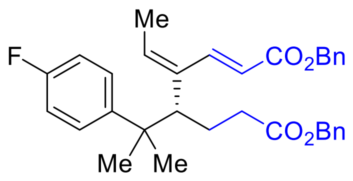

4k

Sample Name : WH-813-12-31D2-OZ-H-rac-1
Sample ID :
Data Filename : WH-813-12-31D2-OZ-H-rac-1.lcd
Method Filename : WH-5-9505-0.5-60MIN.lcm
Batch Filename : wh12.3.lcb
Vial # : 1-74
Injection Volume : 10 uL
Date Acquired : 1/18/2023 2:53:33 PM
Date Processed : 1/18/2023 3:53:36 PM
Sample Type : Unknown
Acquired by : System Administrator
Processed by : System Administrator

<Chromatogram>

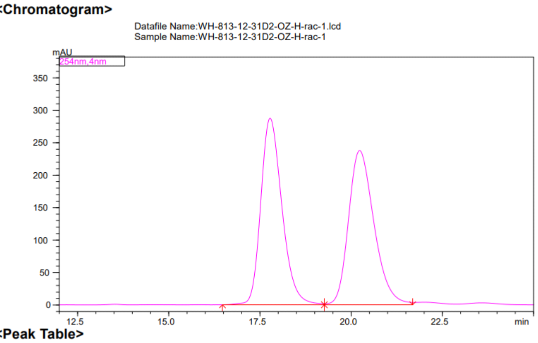

<Peak Table>

PDA Ch1 254nm

Peak#	Ret. Time	Area	Height	Area%
1	17.776	11597897	287326	50.104
2	20.232	11549609	237648	49.896
Total		23147506	524974	100.000

Sample Name : WH-813-14-92A5-2-OZ-H-chi
Sample ID :
Data Filename : WH-813-14-92A5-2-OZ-H-chi.lcd
Method Filename : WH-5-9505-0.5-60MIN.lcm
Batch Filename : wh12.3.lcb
Vial # : 1-75
Injection Volume : 10 uL
Date Acquired : 1/18/2023 4:54:43 PM
Date Processed : 1/18/2023 5:54:46 PM
Sample Type : Unknown
Acquired by : System Administrator
Processed by : System Administrator

<Chromatogram>

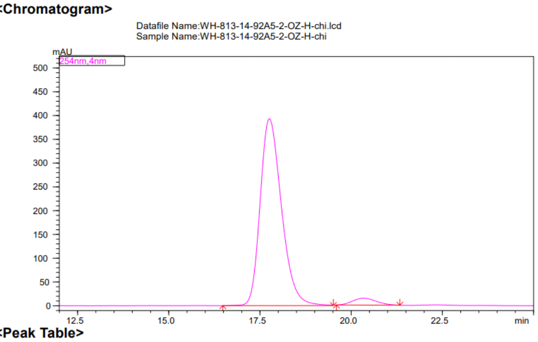

<Peak Table>

PDA Ch1 254nm

Peak#	Ret. Time	Area	Height	Area%
1	17.757	15982862	393154	96.050
2	20.338	657314	14453	3.950
Total		16640176	407607	100.000

Supplementary Figure 149. HPLC spectra of **4k**

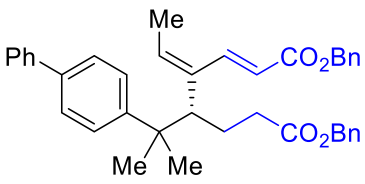

4l

Sample Name	: WH-813-12-43D2-IA-rac-1
Sample ID	:
Data Filename	: WH-813-12-43D2-IA-rac-1.lcd
Method Filename	: WH-2-9505-0.7-30MIN.lcm
Batch Filename	: wh12.3.lcb
Vial #	: 1-89
Sample Type	: Unknown
Injection Volume	: 10 uL
Date Acquired	: 1/18/2023 6:25:45 PM
Acquired by	: System Administrator
Date Processed	: 1/18/2023 6:55:48 PM
Processed by	: System Administrator

<Chromatogram>

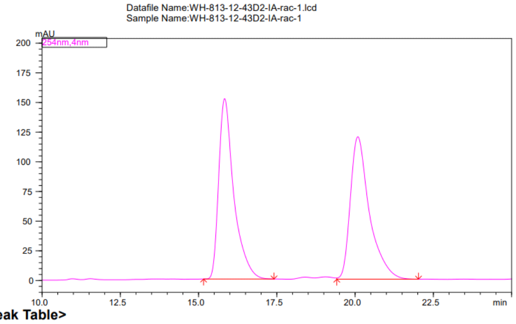

<Peak Table>

PDA Ch1 254nm

Peak#	Ret. Time	Area	Height	Area%
1	15.831	5101721	152058	50.097
2	20.087	5082018	120030	49.903
Total		10183739	272088	100.000

Sample Name	: WH-813-15-31A3-IA-CHI
Sample ID	:
Data Filename	: WH-813-15-31A3-IA-CHI.lcd
Method Filename	: WH-2-9505-0.7-30MIN.lcm
Batch Filename	: wh12.3.lcb
Vial #	: 1-90
Sample Type	: Unknown
Injection Volume	: 10 uL
Date Acquired	: 1/18/2023 7:16:21 PM
Acquired by	: System Administrator
Date Processed	: 1/18/2023 7:46:24 PM
Processed by	: System Administrator

<Chromatogram>

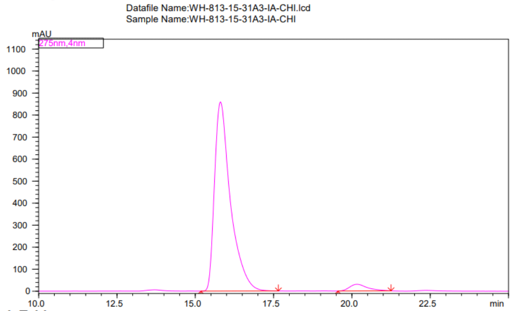

<Peak Table>

PDA Ch1 275nm

Peak#	Ret. Time	Area	Height	Area%
1	15.803	30396071	858869	96.191
2	20.161	1203740	30305	3.809
Total		31599810	889175	100.000

Supplementary Figure 150. HPLC spectra of **4l**

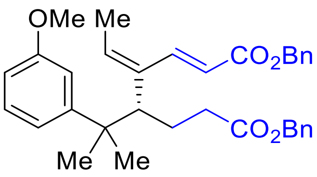

4m

Sample Name	: WH-813-12-34D2-OZ-H-rac-2
Sample ID	:
Data Filename	: WH-813-12-34D2-OZ-H-rac-2.lcd
Method Filename	: WH-2-9505-0.7-60MIN.lcm
Batch Filename	: wh12.3.lcb
Vial #	: 1-74
Injection Volume	: 10 uL
Date Acquired	: 12/10/2022 11:51:28 PM
Date Processed	: 12/26/2022 2:26:54 AM
Sample Type	: Unknown
Acquired by	: System Administrator
Processed by	: System Administrator

<Chromatogram>

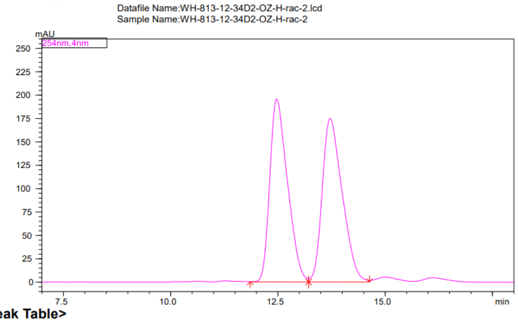

<Peak Table>

PDA Ch1 254nm

Peak#	Ret. Time	Area	Height	Area%
1	12.472	5622574	195649	49.772
2	13.718	5673997	174861	50.228
Total		11296571	370510	100.000

Sample Name	: WH-813-14-99A1-OZ-H-chi-1
Sample ID	:
Data Filename	: WH-813-14-99A1-OZ-H-chi-1.lcd
Method Filename	: WH-2-9505-0.7-60MIN.lcm
Batch Filename	: wh12.3.lcb
Vial #	: 1-90
Injection Volume	: 10 uL
Date Acquired	: 12/11/2022 12:52:02 AM
Date Processed	: 12/26/2022 2:26:52 AM
Sample Type	: Unknown
Acquired by	: System Administrator
Processed by	: System Administrator

<Chromatogram>

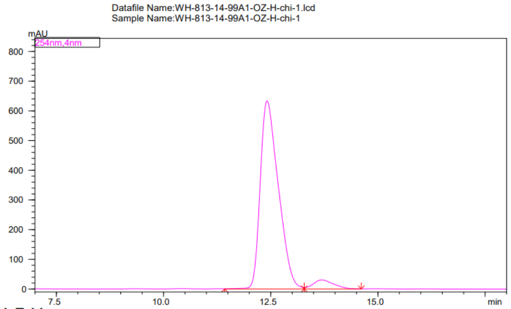

<Peak Table>

PDA Ch1 254nm

Peak#	Ret. Time	Area	Height	Area%
1	12.415	18617608	633525	94.829
2	13.689	1015240	30502	5.171
Total		19632848	664027	100.000

Supplementary Figure 151. HPLC spectra of **4m**

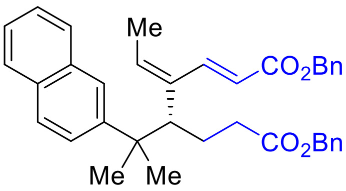

4n

Sample Name : WH-813-12-33D2-IA-rac-1
Sample ID :
Data Filename : WH-813-12-33D2-IA-rac-1.lcd
Method Filename : WH-2-9505-0.7-30MIN.lcm
Batch Filename : wh12.3.lcb
Vial # : 1-70
Injection Volume : 10 uL
Date Acquired : 12/10/2022 1:45:06 AM
Date Processed : 12/10/2022 2:15:09 AM
Sample Type : Unknown
Acquired by : System Administrator
Processed by : System Administrator

<Chromatogram>

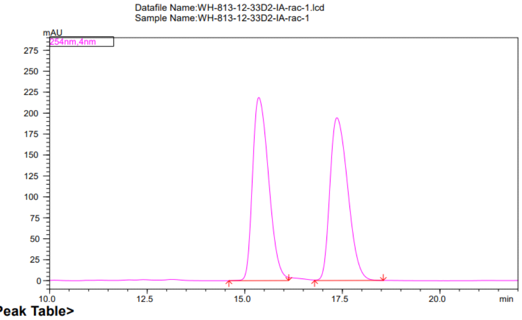

<Peak Table>

PDA Ch1 254nm

Peak#	Ret. Time	Area	Height	Area%
1	15.360	6284723	218680	50.305
2	17.363	6208554	194298	49.695
Total		12493277	412978	100.000

Sample Name : WH-813-14-97A4-IA-CHI
Sample ID :
Data Filename : WH-813-14-97A4-IA-CHI.lcd
Method Filename : WH-2-9505-0.7-30MIN.lcm
Batch Filename : wh12.3.lcb
Vial # : 1-71
Injection Volume : 10 uL
Date Acquired : 12/10/2022 2:15:40 AM
Date Processed : 12/10/2022 2:45:42 AM
Sample Type : Unknown
Acquired by : System Administrator
Processed by : System Administrator

<Chromatogram>

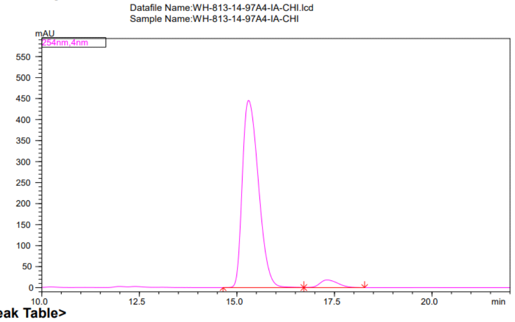

<Peak Table>

PDA Ch1 254nm

Peak#	Ret. Time	Area	Height	Area%
1	15.299	12836522	445430	95.652
2	17.311	583573	18189	4.348
Total		13420094	463620	100.000

Supplementary Figure 152. HPLC spectra of **4n**

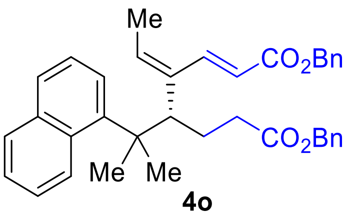

4o

Sample Name : WH-813-12-32D2-IC-rac-2
Sample ID :
Data Filename : WH-813-12-32D2-IC-rac-2.lcd
Method Filename : WH-4-9505-0.5-60MIN.lcm
Batch Filename : wh12.3.lcb
Vial # : 1-72
Injection Volume : 10 uL
Date Acquired : 12/11/2022 2:23:03 AM
Date Processed : 12/26/2022 2:26:50 AM
Sample Type : Unknown
Acquired by : System Administrator
Processed by : System Administrator

<Chromatogram>

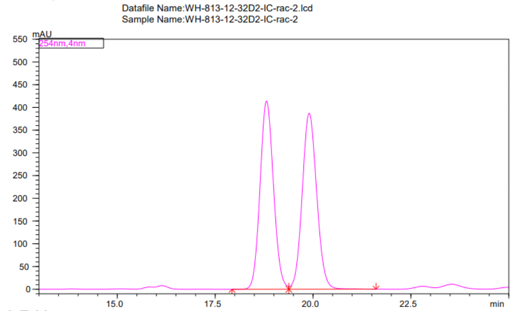

<Peak Table>

PDA Ch1 254nm

Peak#	Ret. Time	Area	Height	Area%
1	18.812	9918508	414027	49.741
2	19.901	10021894	387308	50.259
Total		19940403	801335	100.000

Sample Name : WH-813-14-97A5-IC-CHI-1
Sample ID :
Data Filename : WH-813-14-97A5-IC-CHI-1.lcd
Method Filename : WH-4-9505-0.5-60MIN.lcm
Batch Filename : wh12.3.lcb
Vial # : 1-73
Injection Volume : 10 uL
Date Acquired : 12/11/2022 3:23:39 AM
Date Processed : 12/26/2022 2:26:53 AM
Sample Type : Unknown
Acquired by : System Administrator
Processed by : System Administrator

<Chromatogram>

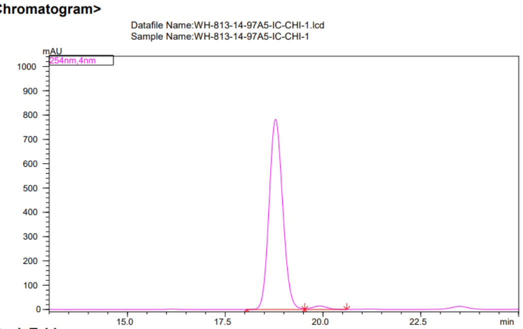

<Peak Table>

PDA Ch1 254nm

Peak#	Ret. Time	Area	Height	Area%
1	18.786	18949736	782410	97.989
2	19.908	388996	14220	2.011
Total		19338733	796630	100.000

Supplementary Figure 153. HPLC spectra of **4o**

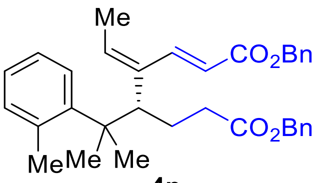

4p

Sample Name : WH-813-12-15D2-OZ-H-rac-1
Sample ID :
Data Filename : WH-813-12-15D2-OZ-H-rac-1.lcd
Method Filename : WH-5-9505-0.5-60MIN.lcm
Batch Filename : wh12.3.lcb
Vial # : 1-67
Injection Volume : 20 uL
Date Acquired : 1/4/2023 6:23:16 PM
Date Processed : 1/4/2023 7:23:19 PM
Sample Type : Unknown
Acquired by : System Administrator
Processed by : System Administrator

<Chromatogram>

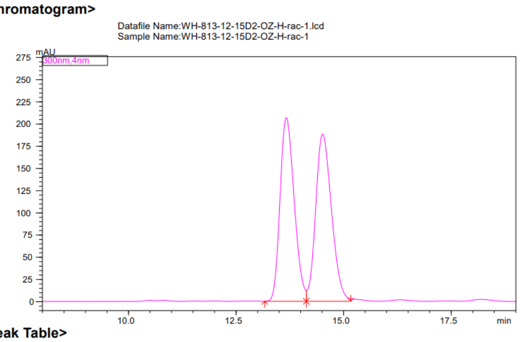

<Peak Table>

PDA Ch1 300nm

Peak#	Ret. Time	Area	Height	Area%
1	13.667	4724081	206857	49.523
2	14.512	4815150	188178	50.477
Total		9539231	395034	100.000

Sample Name : WH-813-15-3A2-1-OZ-H-chi
Sample ID :
Data Filename : WH-813-15-3A2-1-OZ-H-chi.lcd
Method Filename : WH-5-9505-0.5-60MIN.lcm
Batch Filename : wh12.3.lcb
Vial # : 1-75
Injection Volume : 20 uL
Date Acquired : 1/4/2023 7:23:50 PM
Date Processed : 1/4/2023 8:23:54 PM
Sample Type : Unknown
Acquired by : System Administrator
Processed by : System Administrator

<Chromatogram>

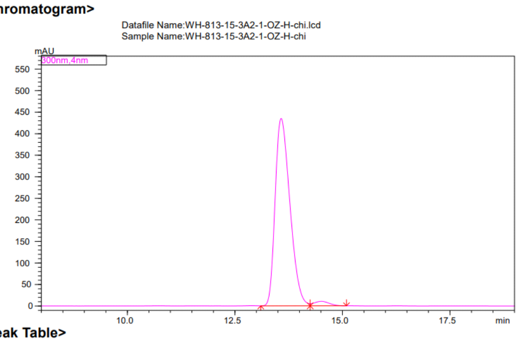

<Peak Table>

PDA Ch1 300nm

Peak#	Ret. Time	Area	Height	Area%
1	13.574	10398048	435015	97.599
2	14.510	255792	10418	2.401
Total		10653840	445433	100.000

Supplementary Figure 154. HPLC spectra of **4p**

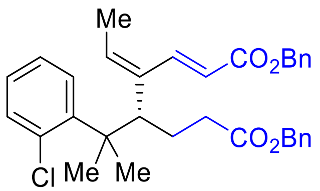

4q

Sample Name	: WH-813-12-70D2-OZ-H-rac-4		
Sample ID	:		
Data Filename	: WH-813-12-70D2-OZ-H-rac-4.lcd		
Method Filename	: WH-2-9505-0.5-50MIN.lcm		
Batch Filename	: wh12.3.lcb		
Vial #	: 1-68	Sample Type	: Unknown
Injection Volume	: 10 uL		
Date Acquired	: 12/22/2022 11:58:48 AM	Acquired by	: System Administrator
Date Processed	: 12/22/2022 12:48:51 PM	Processed by	: System Administrator

<Chromatogram>

Datafile Name:WH-813-12-70D2-OZ-H-rac-4.lcd
Sample Name:WH-813-12-70D2-OZ-H-rac-4

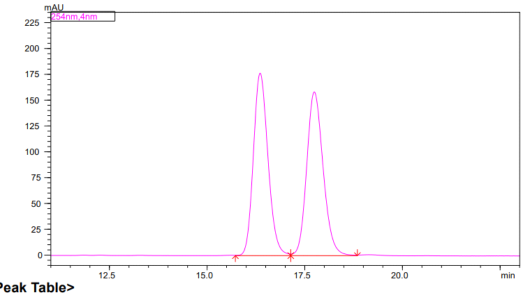

<Peak Table>

PDA Ch1 254nm

Peak#	Ret. Time	Area	Height	Area%
1	16.357	4601594	176627	49.792
2	17.745	4639982	158554	50.208
Total		9241576	335181	100.000

Sample Name	: WH-813-14-97A3-OZ-H-CHI-2		
Sample ID	:		
Data Filename	: WH-813-14-97A3-OZ-H-CHI-2.lcd		
Method Filename	: WH-2-9505-0.5-50MIN.lcm		
Batch Filename	: wh12.3.lcb		
Vial #	: 1-69	Sample Type	: Unknown
Injection Volume	: 10 uL		
Date Acquired	: 12/22/2022 12:49:22 PM	Acquired by	: System Administrator
Date Processed	: 12/22/2022 1:39:25 PM	Processed by	: System Administrator

<Chromatogram>

Datafile Name:WH-813-14-97A3-OZ-H-CHI-2.lcd
Sample Name:WH-813-14-97A3-OZ-H-CHI-2

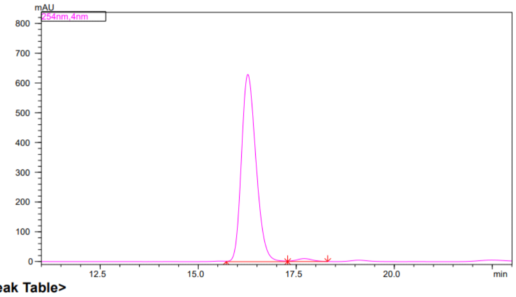

<Peak Table>

PDA Ch1 254nm

Peak#	Ret. Time	Area	Height	Area%
1	16.266	16252276	629253	98.240
2	17.704	291113	9876	1.760
Total		16543389	639129	100.000

Supplementary Figure 155. HPLC spectra of **4q**

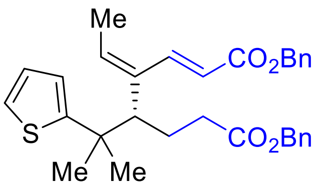

4r

Sample Name	: WH-813-12-45D2-OZ-H-rac-1		
Sample ID	:		
Data Filename	: WH-813-12-45D2-OZ-H-rac-1.lcd		
Method Filename	: WH-5-9505-0.5-60MIN.lcm		
Batch Filename	: wh12.3.lcb		
Vial #	: 1-72	Sample Type	: Unknown
Injection Volume	: 10 uL		
Date Acquired	: 1/18/2023 12:21:55 PM	Acquired by	: System Administrator
Date Processed	: 1/18/2023 1:21:57 PM	Processed by	: System Administrator

Sample Name	: WH-813-15-31A1-2-OZ-H-CHI		
Sample ID	:		
Data Filename	: WH-813-15-31A1-2-OZ-H-CHI.lcd		
Method Filename	: WH-5-9505-0.5-60MIN.lcm		
Batch Filename	: wh12.3.lcb		
Vial #	: 1-73	Sample Type	: Unknown
Injection Volume	: 10 uL		
Date Acquired	: 1/18/2023 1:22:29 PM	Acquired by	: System Administrator
Date Processed	: 1/18/2023 2:22:33 PM	Processed by	: System Administrator

<Chromatogram>

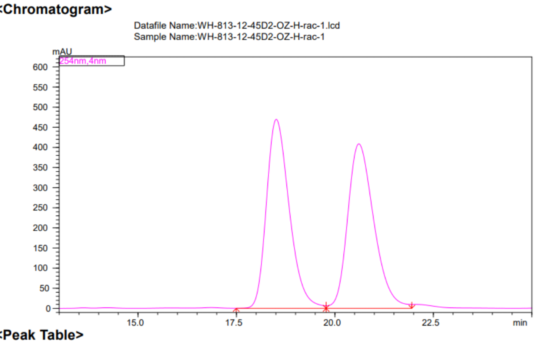

<Peak Table>

PDA Ch1 254nm

Peak#	Ret. Time	Area	Height	Area%
1	18.512	18974082	469652	50.016
2	20.608	18962052	408725	49.984
Total		37936134	878377	100.000

<Chromatogram>

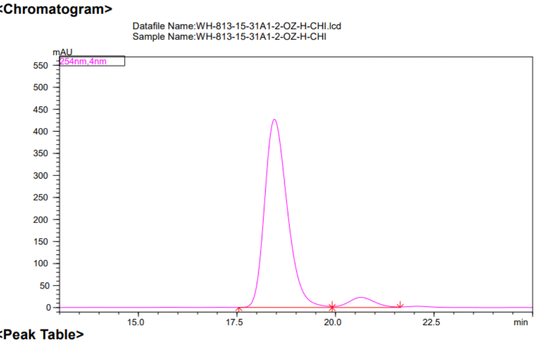

<Peak Table>

PDA Ch1 254nm

Peak#	Ret. Time	Area	Height	Area%
1	18.451	17117032	427236	94.101
2	20.650	1073096	22959	5.899
Total		18190128	450195	100.000

Supplementary Figure 156. HPLC spectra of **4r**

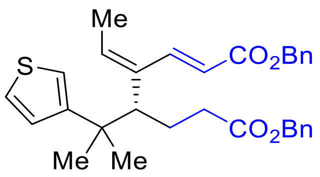

4s

Sample Name	: WH-813-12-44D2-OZ-H-rac-2		
Sample ID	:		
Data Filename	: WH-813-12-44D2-OZ-H-rac-2.lcd		
Method Filename	: WH-5-9505-0.5-60MIN.lcm		
Batch Filename	: wh12.3.lcb		
Vial #	: 1-64	Sample Type	: Unknown
Injection Volume	: 20 uL		
Date Acquired	: 1/4/2023 10:48:26 AM	Acquired by	: System Administrator
Date Processed	: 1/4/2023 11:48:29 AM	Processed by	: System Administrator

<Chromatogram>

Datafile Name:WH-813-12-44D2-OZ-H-rac-2.lcd
Sample Name:WH-813-12-44D2-OZ-H-rac-2

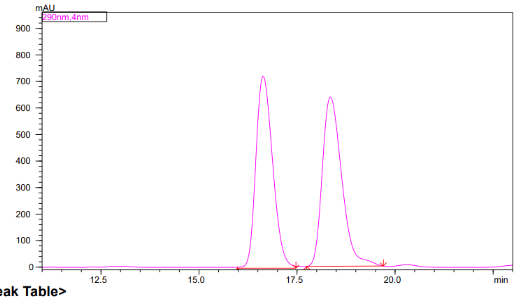

<Peak Table>

PDA Ch1 290nm

Peak#	Ret. Time	Area	Height	Area%
1	16.634	21597248	723729	49.563
2	18.351	21978057	637809	50.437
Total		43575305	1361538	100.000

Sample Name	: WH-813-15-1A3-OZ-H-CHI		
Sample ID	:		
Data Filename	: WH-813-15-1A3-OZ-H-CHI.lcd		
Method Filename	: WH-5-9505-0.5-60MIN.lcm		
Batch Filename	: wh12.3.lcb		
Vial #	: 1-72	Sample Type	: Unknown
Injection Volume	: 20 uL		
Date Acquired	: 1/4/2023 11:49:00 AM	Acquired by	: System Administrator
Date Processed	: 1/4/2023 12:49:03 PM	Processed by	: System Administrator

<Chromatogram>

Datafile Name:WH-813-15-1A3-OZ-H-CHI.lcd
Sample Name:WH-813-15-1A3-OZ-H-CHI

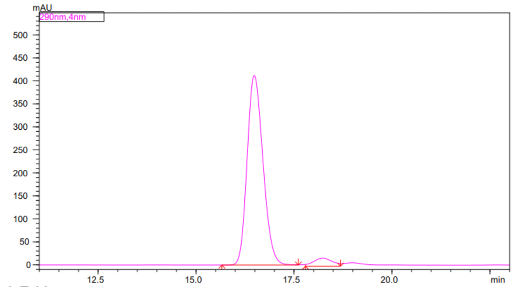

<Peak Table>

PDA Ch1 290nm

Peak#	Ret. Time	Area	Height	Area%
1	16.486	11606488	412069	95.133
2	18.230	593725	17567	4.867
Total		12200213	429636	100.000

Supplementary Figure 157. HPLC spectra of **4s**

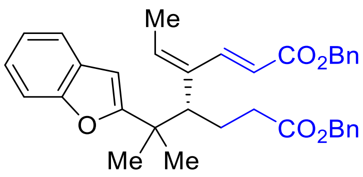

4t

Sample Name : WH-813-12-66D2-IA-rac
Sample ID :
Data Filename : WH-813-12-66D2-IA-rac.lcd
Method Filename : WH-2-9505-0.7-30MIN.lcm
Batch Filename : wh.lcb
Vial # : 1-65
Injection Volume : 10 uL
Date Acquired : 3/12/2022 5:39:19 PM
Date Processed : 3/12/2022 6:09:22 PM
Sample Type : Unknown
Acquired by : System Administrator
Processed by : System Administrator

<Chromatogram>

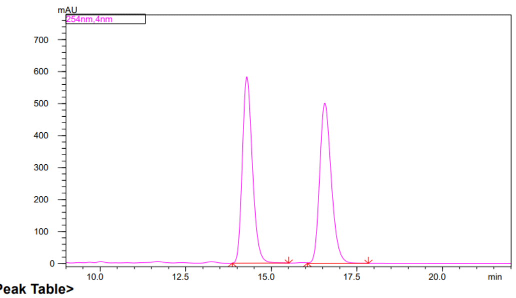

<Peak Table>

PDA Ch1 254nm

Peak#	Ret. Time	Area	Height	Area%
1	14.284	11951604	582800	50.186
2	16.562	11862807	501002	49.814
Total		23814411	1083803	100.000

Sample Name : WH-813-12-66D1-IA-CHI
Sample ID :
Data Filename : WH-813-12-66D1-IA-CHI.lcd
Method Filename : WH-2-9505-0.7-30MIN.lcm
Batch Filename : wh.lcb
Vial # : 1-66
Injection Volume : 10 uL
Date Acquired : 3/12/2022 9:18:18 PM
Date Processed : 3/12/2022 9:48:21 PM
Sample Type : Unknown
Acquired by : System Administrator
Processed by : System Administrator

<Chromatogram>

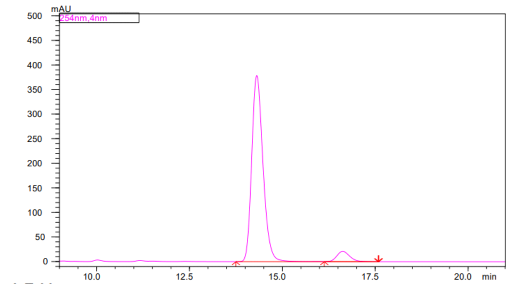

<Peak Table>

PDA Ch1 254nm

Peak#	Ret. Time	Area	Height	Area%
1	14.311	8010050	378853	94.029
2	16.626	508688	21082	5.971
Total		8518738	399936	100.000

Supplementary Figure 158. HPLC spectra of **4t**

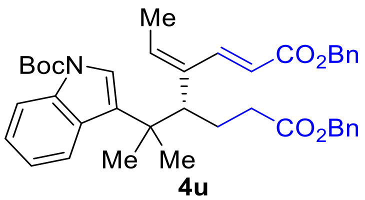

4u

Sample Name	: WH-813-12-88D2-IF-rac-1		
Sample ID	:		
Data Filename	: WH-813-12-88D2-IF-rac-1.lcd		
Method Filename	: WH-6-9505-0.5-60MIN.lcm		
Batch Filename	: wh12.3.lcb		
Vial #	: 1-63	Sample Type	: Unknown
Injection Volume	: 20 uL		
Date Acquired	: 1/4/2023 8:16:49 AM	Acquired by	: System Administrator
Date Processed	: 1/4/2023 9:16:53 AM	Processed by	: System Administrator

<Chromatogram>

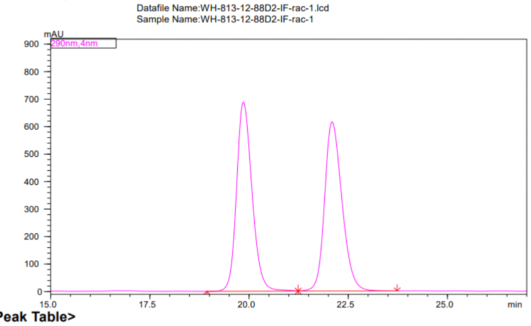

<Peak Table>

PDA Ch1 290nm

Peak#	Ret. Time	Area	Height	Area%
1	19.858	18682084	688246	49.822
2	22.092	18815735	615626	50.178
Total		37497819	1303872	100.000

Sample Name	: WH-813-15-1A2-IF-CHI		
Sample ID	:		
Data Filename	: WH-813-15-1A2-IF-CHI.lcd		
Method Filename	: WH-6-9505-0.5-60MIN.lcm		
Batch Filename	: wh12.3.lcb		
Vial #	: 1-71	Sample Type	: Unknown
Injection Volume	: 20 uL		
Date Acquired	: 1/4/2023 9:17:25 AM	Acquired by	: System Administrator
Date Processed	: 1/4/2023 10:17:28 AM	Processed by	: System Administrator

<Chromatogram>

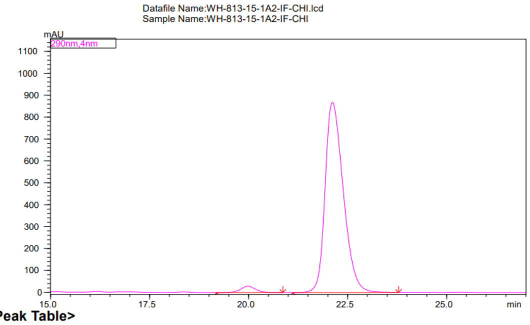

<Peak Table>

PDA Ch1 290nm

Peak#	Ret. Time	Area	Height	Area%
1	19.980	762024	28820	2.668
2	22.115	27800305	868696	97.332
Total		28562329	897516	100.000

Supplementary Figure 159. HPLC spectra of **4u**

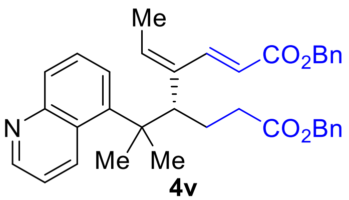

4v

Sample Name	: WH-813-12-89D2-OZ-H-rac-6
Sample ID	:
Data Filename	: WH-813-12-89D2-OZ-H-rac-6.lcd
Method Filename	: WH-5-9010-1.0-60MIN.lcm
Batch Filename	: wh12.3.lcb
Vial #	: 1-62
Injection Volume	: 20 uL
Date Acquired	: 1/4/2023 4:44:40 AM
Date Processed	: 1/4/2023 5:44:43 AM
Sample Type	: Unknown
Acquired by	: System Administrator
Processed by	: System Administrator

<Chromatogram>

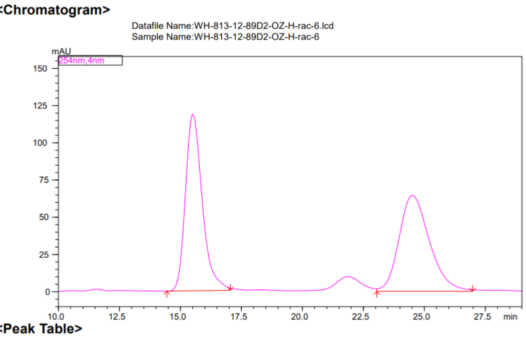

<Peak Table>

PDA Ch1 254nm

Peak#	Ret. Time	Area	Height	Area%
1	15.508	5694887	118528	50.429
2	24.506	5597916	64450	49.571
Total		11292803	182978	100.000

Sample Name	: WH-813-15-33A1-OZ-H-CHI
Sample ID	:
Data Filename	: WH-813-15-33A1-OZ-H-CHI.lcd
Method Filename	: WH-5-9010-1.0-60MIN.lcm
Batch Filename	: wh12.3.lcb
Vial #	: 1-70
Injection Volume	: 20 uL
Date Acquired	: 1/4/2023 6:45:49 AM
Date Processed	: 1/4/2023 7:45:52 AM
Sample Type	: Unknown
Acquired by	: System Administrator
Processed by	: System Administrator

<Chromatogram>

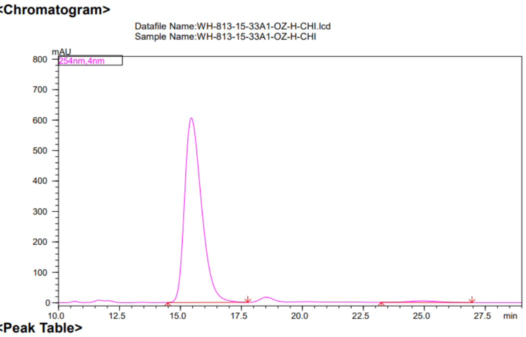

<Peak Table>

PDA Ch1 254nm

Peak#	Ret. Time	Area	Height	Area%
1	15.446	29416936	606079	98.588
2	24.905	421460	4689	1.412
Total		29838395	610767	100.000

Supplementary Figure 160. HPLC spectra of **4v**

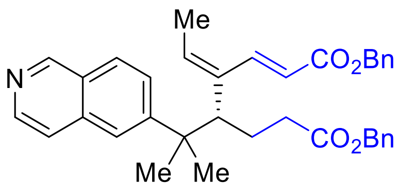

4w

Sample Name : WH-813-12-90D2-IF-rac-2
Sample ID :
Data Filename : WH-813-12-90D2-IF-rac-2.lcd
Method Filename : WH-6-9208-1.0-60MIN.lcm
Batch Filename : wh12.3.lcb
Vial # : 1-69
Injection Volume : 20 uL
Date Acquired : 1/4/2023 11:26:28 PM
Date Processed : 1/5/2023 12:26:31 AM
Sample Type : Unknown
Acquired by : System Administrator
Processed by : System Administrator

<Chromatogram>

Datafile Name:WH-813-12-90D2-IF-rac-2.lcd
Sample Name:WH-813-12-90D2-IF-rac-2

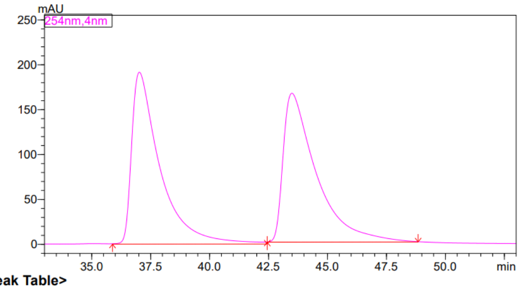

<Peak Table>

PDA Ch1 254nm

Peak#	Ret. Time	Area	Height	Area%
1	37.022	16013168	191391	49.643
2	43.488	16243611	165875	50.357
Total		32256779	357265	100.000

Sample Name : WH-813-15-33A2-IF-CHI
Sample ID :
Data Filename : WH-813-15-33A2-IF-CHI.lcd
Method Filename : WH-6-9208-1.0-60MIN.lcm
Batch Filename : wh12.3.lcb
Vial # : 1-89
Injection Volume : 20 uL
Date Acquired : 1/5/2023 12:27:03 AM
Date Processed : 1/5/2023 1:27:06 AM
Sample Type : Unknown
Acquired by : System Administrator
Processed by : System Administrator

<Chromatogram>

Datafile Name:WH-813-15-33A2-IF-CHI.lcd
Sample Name:WH-813-15-33A2-IF-CHI

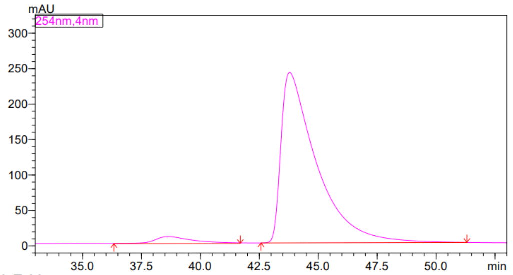

<Peak Table>

PDA Ch1 254nm

Peak#	Ret. Time	Area	Height	Area%
1	38.624	1119259	9869	4.254
2	43.788	25193175	240331	95.746
Total		26312434	250200	100.000

Supplementary Figure 161. HPLC spectra of **4w**

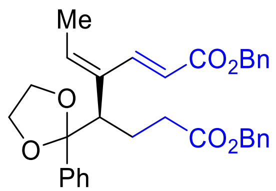

4x

Sample Name : WH-813-11-75-OZ-H-rac-3
Sample ID :
Data Filename : WH-813-11-75-OZ-H-rac-3.lcd
Method Filename : WH-5-9505-0.7-40MIN.lcm
Batch Filename : wh.lcb
Vial # : 1-61 Sample Type : Unknown
Injection Volume : 10 uL
Date Acquired : 12/15/2021 1:03:41 AM Acquired by : System Administrator
Date Processed : 12/15/2021 11:37:47 AM Processed by : System Administrator

<Chromatogram>

Datafile Name:WH-813-11-75-OZ-H-rac-3.lcd
Sample Name:WH-813-11-75-OZ-H-rac-3

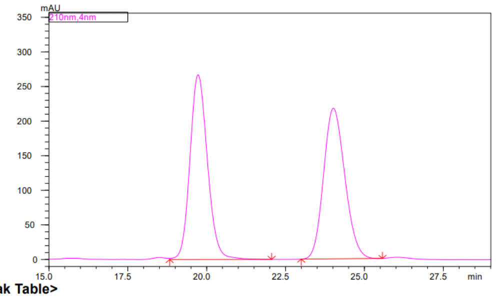

<Peak Table>

PDA Ch1 210nm

Peak#	Ret. Time	Area	Height	Area%
1	19.726	10306238	267016	50.228
2	24.016	10212771	217554	49.772
Total		20519009	484571	100.000

Sample Name : WH-813-11-89D1-2-OZ-H-CHI-1
Sample ID :
Data Filename : WH-813-11-89D1-2-OZ-H-CHI-1.lcd
Method Filename : WH-5-9505-0.7-40MIN.lcm
Batch Filename : wh.lcb
Vial # : 1-62 Sample Type : Unknown
Injection Volume : 10 uL
Date Acquired : 12/15/2021 1:44:14 AM Acquired by : System Administrator
Date Processed : 12/16/2021 10:44:14 AM Processed by : System Administrator

<Chromatogram>

Datafile Name:WH-813-11-89D1-2-OZ-H-CHI-1.lcd
Sample Name:WH-813-11-89D1-2-OZ-H-CHI-1

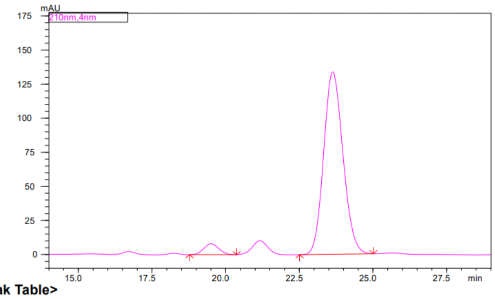

<Peak Table>

PDA Ch1 210nm

Peak#	Ret. Time	Area	Height	Area%
1	19.509	284403	7971	4.456
2	23.635	6098095	133772	95.544
Total		6382498	141744	100.000

Supplementary Figure 162. HPLC spectra of **4x**

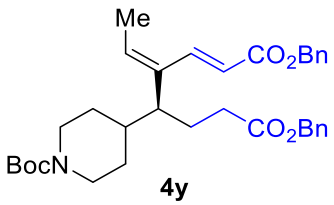

4y

Sample Name : WH-813-13-54D2-2-IB-rac-6
Sample ID :
Data Filename : WH-813-13-54D2-2-IB-rac-6.lcd
Method Filename : WH-6-9505-0.3-60MIN.lcm
Batch Filename : wh.lcb
Vial # : 1-62
Sample Type : Unknown
Injection Volume : 10 uL
Date Acquired : 11/28/2022 6:53:56 PM
Acquired by : System Administrator
Date Processed : 11/28/2022 7:53:59 PM
Processed by : System Administrator

<Chromatogram>

Datafile Name:WH-813-13-54D2-2-IB-rac-6.lcd
Sample Name:WH-813-13-54D2-2-IB-rac-6

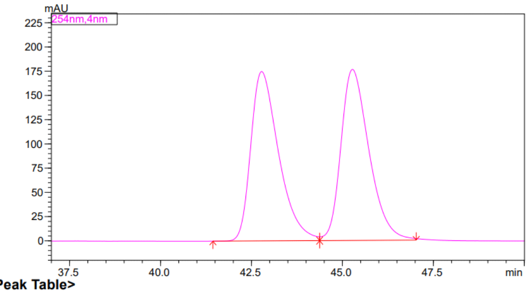

<Peak Table>

PDA Ch1 254nm

Peak#	Ret. Time	Area	Height	Area%
1	42.780	9325061	174800	49.529
2	45.276	9502361	176510	50.471
Total		18827422	351310	100.000

Sample Name : WH-813-14-71A1-2-IB-CHI
Sample ID :
Data Filename : WH-813-14-71A1-2-IB-CHI.lcd
Method Filename : WH-6-9505-0.3-60MIN.lcm
Batch Filename : wh.lcb
Vial # : 1-75
Sample Type : Unknown
Injection Volume : 10 uL
Date Acquired : 11/28/2022 8:14:31 PM
Acquired by : System Administrator
Date Processed : 11/28/2022 9:14:35 PM
Processed by : System Administrator

<Chromatogram>

Datafile Name:WH-813-14-71A1-2-IB-CHI.lcd
Sample Name:WH-813-14-71A1-2-IB-CHI

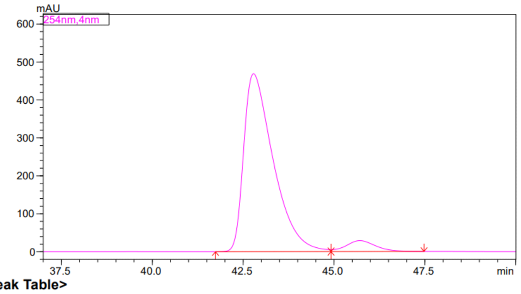

<Peak Table>

PDA Ch1 254nm

Peak#	Ret. Time	Area	Height	Area%
1	42.787	26541343	469026	94.268
2	45.711	1613787	28623	5.732
Total		28155130	497650	100.000

Supplementary Figure 163. HPLC spectra of **4y**

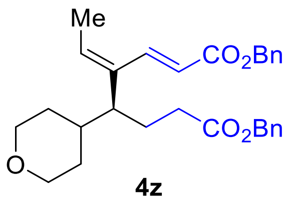

4z

Sample Name : WH-813-14-74-IB-rac-6
Sample ID :
Data Filename : WH-813-14-74-IB-rac-6.lcd
Method Filename : WH-6-9703-0.3-80MIN.lcm
Batch Filename : wh.lcb
Vial # : 1-71
Sample Type : Unknown
Injection Volume : 10 uL
Date Acquired : 11/28/2022 10:20:37 AM
Acquired by : System Administrator
Date Processed : 11/28/2022 11:40:40 AM
Processed by : System Administrator

<Chromatogram>

Datafile Name:WH-813-14-74-IB-rac-6.lcd
Sample Name:WH-813-14-74-IB-rac-6

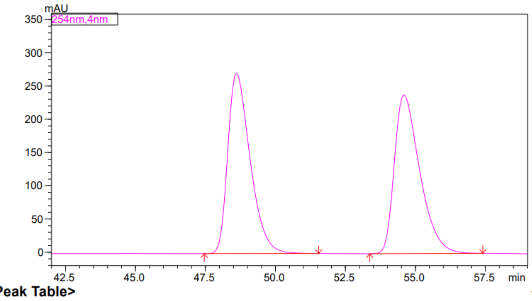

<Peak Table>

PDA Ch1 254nm

Peak#	Ret. Time	Area	Height	Area%
1	48.610	15090559	271164	50.025
2	54.594	15075503	238594	49.975
Total		30166062	509758	100.000

Sample Name : WH-813-14-71A3-2-IB-CHI-1
Sample ID :
Data Filename : WH-813-14-71A3-2-IB-CHI-1.lcd
Method Filename : WH-6-9703-0.3-80MIN.lcm
Batch Filename : wh.lcb
Vial # : 1-74
Sample Type : Unknown
Injection Volume : 10 uL
Date Acquired : 11/28/2022 12:01:11 PM
Acquired by : System Administrator
Date Processed : 11/28/2022 1:21:15 PM
Processed by : System Administrator

<Chromatogram>

Datafile Name:WH-813-14-71A3-2-IB-CHI-1.lcd
Sample Name:WH-813-14-71A3-2-IB-CHI-1

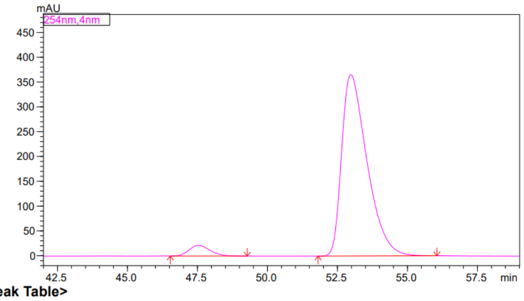

<Peak Table>

PDA Ch1 254nm

Peak#	Ret. Time	Area	Height	Area%
1	47.539	1153215	21764	4.766
2	52.979	23041525	365793	95.234
Total		24194740	387557	100.000

Supplementary Figure 164. HPLC spectra of **4z**

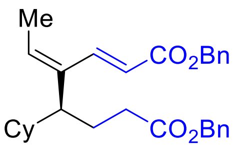

4aa

Sample Name : WH-813-14-64-OD-H-rac-1
Sample ID :
Data Filename : WH-813-14-64-OD-H-rac-1.lcd
Method Filename : WH-1-9505-0.5-60MIN.lcm
Batch Filename : wh.lcb
Vial # : 1-61
Sample Type : Unknown
Injection Volume : 10 uL
Date Acquired : 11/12/2022 5:42:32 AM
Acquired by : System Administrator
Date Processed : 11/12/2022 6:42:36 AM
Processed by : System Administrator

<Chromatogram>

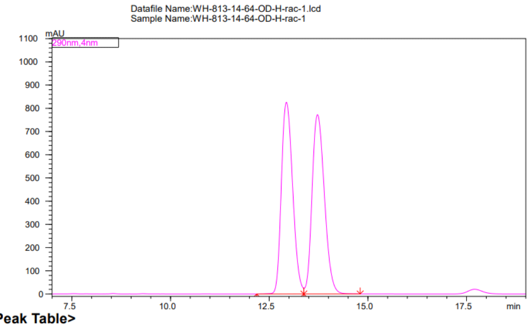

<Peak Table>

PDA Ch1 290nm

Peak#	Ret. Time	Area	Height	Area%
1	12.935	16446823	825694	49.404
2	13.724	16843446	772307	50.596
Total		33290269	1598001	100.000

Sample Name : WH-813-14-63-OD-H-CHI
Sample ID :
Data Filename : WH-813-14-63-OD-H-CHI.lcd
Method Filename : WH-1-9505-0.5-60MIN.lcm
Batch Filename : wh.lcb
Vial # : 1-62
Sample Type : Unknown
Injection Volume : 10 uL
Date Acquired : 11/12/2022 6:43:05 AM
Acquired by : System Administrator
Date Processed : 11/12/2022 7:43:08 AM
Processed by : System Administrator

<Chromatogram>

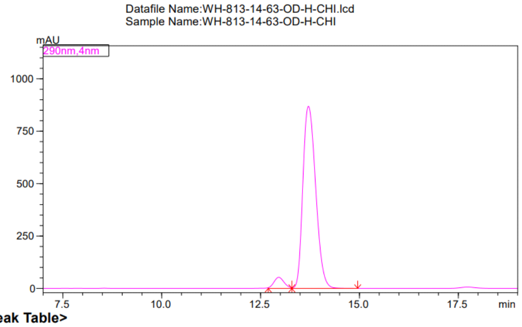

<Peak Table>

PDA Ch1 290nm

Peak#	Ret. Time	Area	Height	Area%
1	12.959	943911	52819	4.604
2	13.710	19556701	868351	95.396
Total		20500612	921170	100.000

Supplementary Figure 165. HPLC spectra of **4aa**

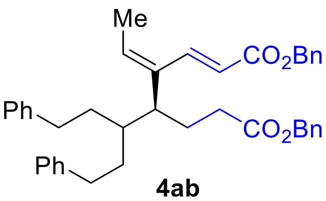

4ab

Sample Name : WH-813-14-75A1-AD-H-rac-3
Sample ID :
Data Filename : WH-813-14-75A1-AD-H-rac-3.lcd
Method Filename : WH-6-9505-0.5-60MIN.lcm
Batch Filename : wh.lcb
Vial # : 1-72
Sample Type : Unknown
Injection Volume : 10 uL
Date Acquired : 11/25/2022 9:12:23 PM
Acquired by : System Administrator
Date Processed : 11/25/2022 10:12:26 PM
Processed by : System Administrator

<Chromatogram>

Datafile Name:WH-813-14-75A1-AD-H-rac-3.lcd
Sample Name:WH-813-14-75A1-AD-H-rac-3

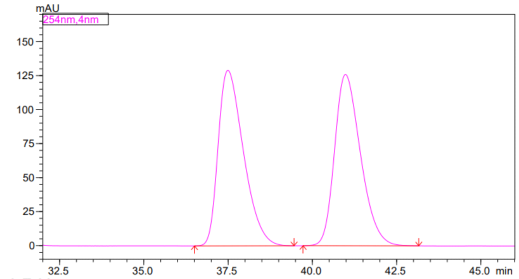

<Peak Table>

PDA Ch1 254nm

Peak#	Ret. Time	Area	Height	Area%
1	37.493	6744613	129049	49.945
2	40.981	6759346	125933	50.055
Total		13503960	254983	100.000

Sample Name : WH-813-14-71A4-2-AD-H-CHI-1
Sample ID :
Data Filename : WH-813-14-71A4-2-AD-H-CHI-1.lcd
Method Filename : WH-6-9505-0.5-60MIN.lcm
Batch Filename : wh.lcb
Vial # : 1-65
Sample Type : Unknown
Injection Volume : 10 uL
Date Acquired : 11/25/2022 10:32:57 PM
Acquired by : System Administrator
Date Processed : 11/25/2022 11:33:00 PM
Processed by : System Administrator

<Chromatogram>

Datafile Name:WH-813-14-71A4-2-AD-H-CHI-1.lcd
Sample Name:WH-813-14-71A4-2-AD-H-CHI-1

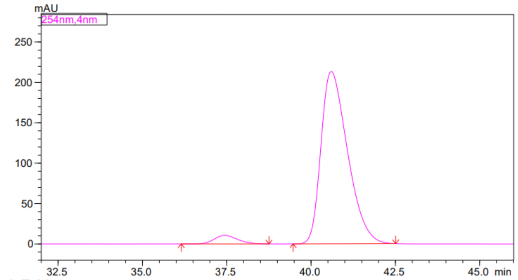

<Peak Table>

PDA Ch1 254nm

Peak#	Ret. Time	Area	Height	Area%
1	37.442	506130	10571	4.155
2	40.598	11675659	213282	95.845
Total		12181790	223853	100.000

Supplementary Figure 166. HPLC spectra of **4ab**

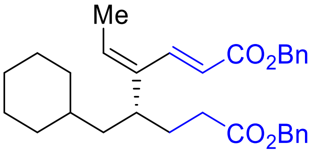

4ac

3733 WH-14-89-1+- IG+IF3 955 214 0.7

Sample Name:	WH-14-89-1+- IG+IF3 955 214 0.7	Injection Volume:	3.0
Vial Number:	BE7	Channel:	EXT270NM
Sample Type:	unknown	Wavelength:	270
Control Program:	test-dad2	Bandwidth:	0
Quantif. Method:	WXL	Dilution Factor:	1.0000
Recording Time:	2023-2-15 14:28	Sample Weight:	1.0000
Run Time (min):	45.82	Sample Amount:	1.0000

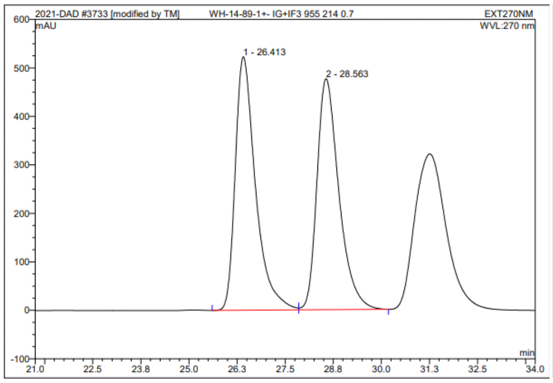

No.	Ret.Time min	Peak Name	Height mAU	Area mAU*min	Rel.Area %	Amount	Type
1	26.41	n.a.	522.837	318.943	50.32	n.a.	BM *
2	28.56	n.a.	476.738	314.899	49.68	n.a.	MB*
Total:			999.575	633.842	100.00	0.000	

3734 WH-14-91-1 IG+IF3 955 214 0.7

Sample Name:	WH-14-91-1 IG+IF3 955 214 0.7	Injection Volume:	3.0
Vial Number:	BE6	Channel:	EXT270NM
Sample Type:	unknown	Wavelength:	270
Control Program:	test-dad2	Bandwidth:	0
Quantif. Method:	WXL	Dilution Factor:	1.0000
Recording Time:	2023-2-15 15:16	Sample Weight:	1.0000
Run Time (min):	108.87	Sample Amount:	1.0000

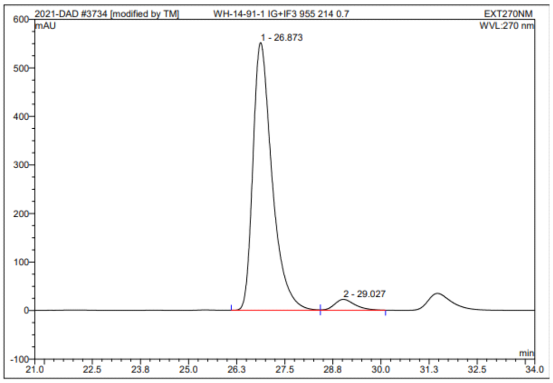

No.	Ret.Time min	Peak Name	Height mAU	Area mAU*min	Rel.Area %	Amount	Type
1	26.87	n.a.	551.590	318.346	95.87	n.a.	BM
2	29.03	n.a.	22.364	13.719	4.13	n.a.	MB
Total:			573.953	332.065	100.00	0.000	

Supplementary Figure 167. HPLC spectra of **4ac**

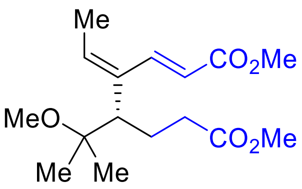

5a

Sample Name	: WH-813-10-91-OZ-H-rac-4		
Sample ID	:		
Data Filename	: WH-813-10-91-OZ-H-rac-4.lcd		
Method Filename	: WH-5-9901-0.5-60MIN.lcm		
Batch Filename	: wh12.3.lcb		
Vial #	: 1-68	Sample Type	: Unknown
Injection Volume	: 20 uL		
Date Acquired	: 1/18/2023 6:58:43 AM	Acquired by	: System Administrator
Date Processed	: 1/18/2023 7:58:47 AM	Processed by	: System Administrator

<Chromatogram>

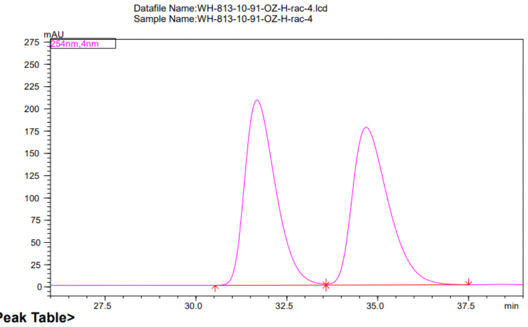

<Peak Table>

PDA Ch1 254nm

Peak#	Ret. Time	Area	Height	Area%
1	31.680	12218077	207951	50.087
2	34.683	12175426	177031	49.913
Total		24393503	384982	100.000

Sample Name	: WH-813-15-28B1-OZ-H-chi		
Sample ID	:		
Data Filename	: WH-813-15-28B1-OZ-H-chi.lcd		
Method Filename	: WH-5-9901-0.5-60MIN.lcm		
Batch Filename	: wh12.3.lcb		
Vial #	: 1-69	Sample Type	: Unknown
Injection Volume	: 10 uL		
Date Acquired	: 1/18/2023 7:59:18 AM	Acquired by	: System Administrator
Date Processed	: 1/18/2023 8:59:21 AM	Processed by	: System Administrator

<Chromatogram>

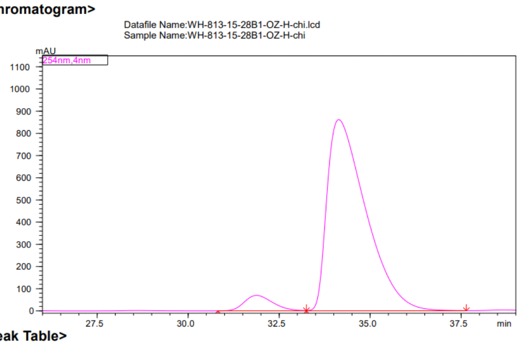

<Peak Table>

PDA Ch1 254nm

Peak#	Ret. Time	Area	Height	Area%
1	31.880	3787381	69617	5.603
2	34.137	63812261	861849	94.397
Total		67599642	931466	100.000

Supplementary Figure 168. HPLC spectra of **5a**

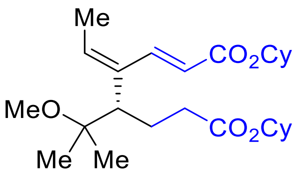

5b

Sample Name	: WH-813-12-22D1E1-IE-rac-1		
Sample ID	:		
Data Filename	: WH-813-12-22D1E1-IE-rac-1.lcd		
Method Filename	: WH-1-9505-0.5-60MIN.lcm		
Batch Filename	: wh12.3.lcb		
Vial #	: 1-70	Sample Type	: Unknown
Injection Volume	: 10 uL		
Date Acquired	: 1/18/2023 9:30:19 AM	Acquired by	: System Administrator
Date Processed	: 1/18/2023 10:30:23 AM	Processed by	: System Administrator

<Chromatogram>

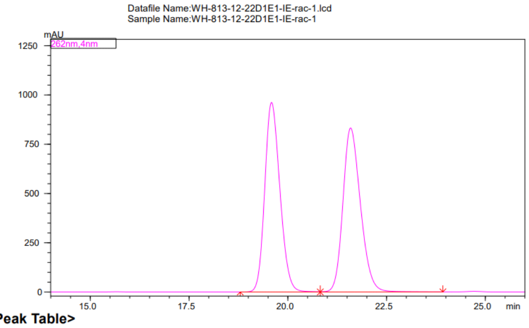

<Peak Table>

PDA Ch1 262nm

Peak#	Ret. Time	Area	Height	Area%
1	19.588	25670699	961776	49.934
2	21.589	25738237	832262	50.066
Total		51408936	1794038	100.000

Sample Name	: WH-813-15-28B2-2-IE-CHI		
Sample ID	:		
Data Filename	: WH-813-15-28B2-2-IE-CHI.lcd		
Method Filename	: WH-1-9505-0.5-60MIN.lcm		
Batch Filename	: wh12.3.lcb		
Vial #	: 1-71	Sample Type	: Unknown
Injection Volume	: 10 uL		
Date Acquired	: 1/18/2023 10:50:54 AM	Acquired by	: System Administrator
Date Processed	: 1/18/2023 11:50:57 AM	Processed by	: System Administrator

<Chromatogram>

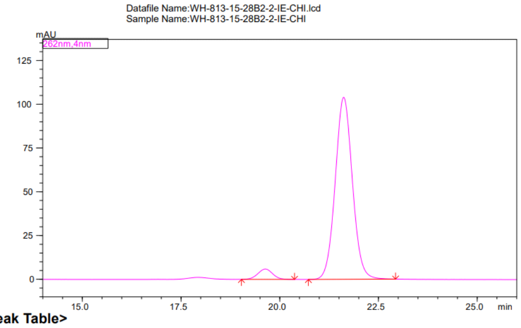

<Peak Table>

PDA Ch1 262nm

Peak#	Ret. Time	Area	Height	Area%
1	19.631	154720	5916	4.681
2	21.618	3150346	103979	95.319
Total		3305067	109896	100.000

Supplementary Figure 169. HPLC spectra of **5b**

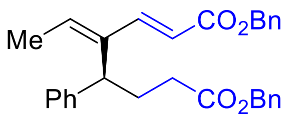

6a

Sample Name	: WH-813-13-56D2-3-IG-rac-1		
Sample ID	:		
Data Filename	: WH-813-13-56D2-3-IG-rac-1.lcd		
Method Filename	: WH-6-9505-1.0-60MIN.lcm		
Batch Filename	: wh.lcb		
Vial #	: 1-61	Sample Type	: Unknown
Injection Volume	: 20 uL		
Date Acquired	: 10/7/2022 2:03:19 PM	Acquired by	: System Administrator
Date Processed	: 10/14/2022 5:07:28 PM	Processed by	: System Administrator

<Chromatogram>

Datafile Name:WH-813-13-56D2-3-IG-rac-1.lcd
Sample Name:WH-813-13-56D2-3-IG-rac-1

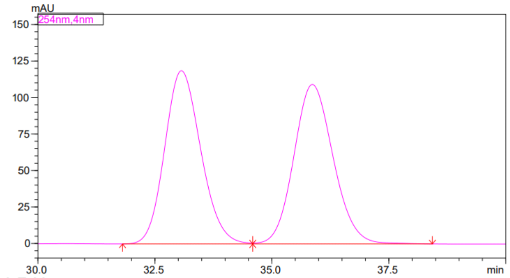

<Peak Table>

PDA Ch1 254nm

Peak#	Ret. Time	Area	Height	Area%
1	33.069	6369688	118668	49.894
2	35.866	6396742	109257	50.106
Total		12766430	227926	100.000

Sample Name	: WH-813-13-100E1-2-IG-CHI		
Sample ID	:		
Data Filename	: WH-813-13-100E1-2-IG-CHI.lcd		
Method Filename	: WH-6-9505-1.0-60MIN.lcm		
Batch Filename	: wh.lcb		
Vial #	: 1-62	Sample Type	: Unknown
Injection Volume	: 20 uL		
Date Acquired	: 10/7/2022 3:03:53 PM	Acquired by	: System Administrator
Date Processed	: 10/7/2022 4:03:57 PM	Processed by	: System Administrator

<Chromatogram>

Datafile Name:WH-813-13-100E1-2-IG-CHI.lcd
Sample Name:WH-813-13-100E1-2-IG-CHI

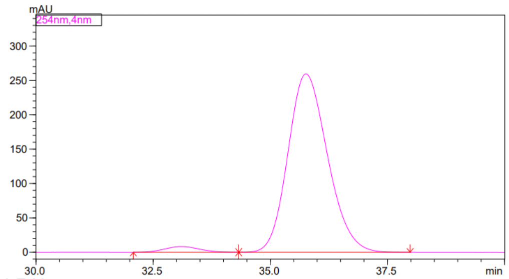

<Peak Table>

PDA Ch1 254nm

Peak#	Ret. Time	Area	Height	Area%
1	33.102	437339	8314	2.785
2	35.761	15266220	259508	97.215
Total		15703560	267822	100.000

Supplementary Figure 170. HPLC spectra of **6a**

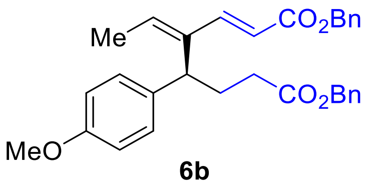

6b

Sample Name : WH-813-14-48A1-2-OD-H-rac-3
Sample ID :
Data Filename : WH-813-14-48A1-2-OD-H-rac-3.lcd
Method Filename : WH-2-9307-1.0-60MIN.lcm
Batch Filename : wh.lcb
Vial # : 1-71 Sample Type : Unknown
Injection Volume : 25 uL
Date Acquired : 11/4/2022 6:56:02 PM Acquired by : System Administrator
Date Processed : 11/4/2022 7:56:04 PM Processed by : System Administrator

<Chromatogram>

Datafile Name:WH-813-14-48A1-2-OD-H-rac-3.lcd
Sample Name:WH-813-14-48A1-2-OD-H-rac-3

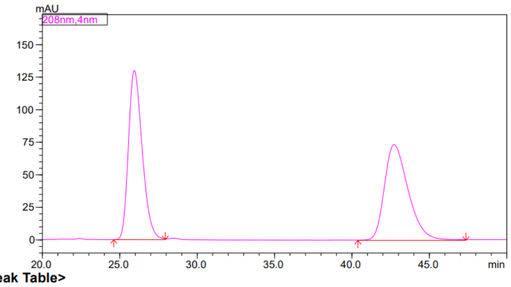

<Peak Table>

PDA Ch1 208nm

Peak#	Ret. Time	Area	Height	Area%
1	25.939	8040749	130018	50.377
2	42.721	7920448	73678	49.623
Total		15961197	203696	100.000

Sample Name : WH-813-14-10-2-OD-H-CHI-2
Sample ID :
Data Filename : WH-813-14-10-2-OD-H-CHI-2.lcd
Method Filename : WH-2-9307-1.0-60MIN.lcm
Batch Filename : wh.lcb
Vial # : 1-75 Sample Type : Unknown
Injection Volume : 25 uL
Date Acquired : 11/4/2022 7:56:37 PM Acquired by : System Administrator
Date Processed : 11/4/2022 8:56:41 PM Processed by : System Administrator

<Chromatogram>

Datafile Name:WH-813-14-10-2-OD-H-CHI-2.lcd
Sample Name:WH-813-14-10-2-OD-H-CHI-2

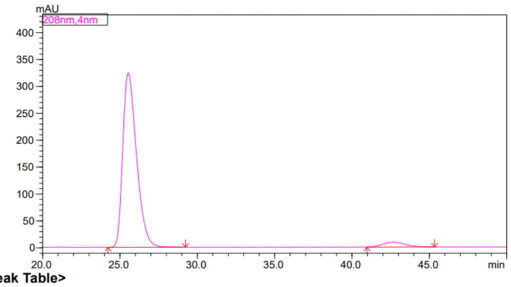

<Peak Table>

PDA Ch1 208nm

Peak#	Ret. Time	Area	Height	Area%
1	25.536	20059359	324052	95.512
2	42.642	942561	9165	4.488
Total		21001920	333217	100.000

Supplementary Figure 171. HPLC spectra of **6b**

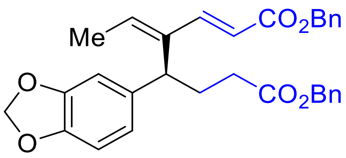

6c

Sample Name	: WH-813-14-60A3-IA-rac-1		
Sample ID	:		
Data Filename	: WH-813-14-60A3-IA-rac-1.lcd		
Method Filename	: WH-5-9505-1.0-60MIN.lcm		
Batch Filename	: wh.lcb		
Vial #	: 1-66	Sample Type	: Unknown
Injection Volume	: 10 uL		
Date Acquired	: 11/10/2022 11:44:56 AM	Acquired by	: System Administrator
Date Processed	: 11/10/2022 12:44:58 PM	Processed by	: System Administrator

<Chromatogram>

Datafile Name:WH-813-14-60A3-IA-rac-1.lcd
Sample Name:WH-813-14-60A3-IA-rac-1

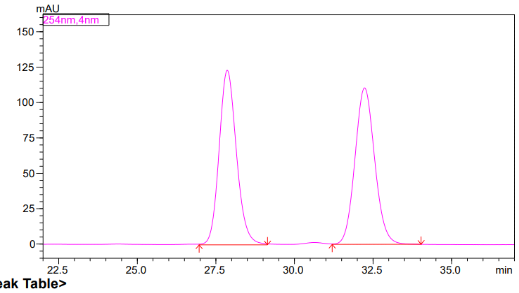

<Peak Table>

PDA Ch1 254nm

Peak#	Ret. Time	Area	Height	Area%
1	27.861	4741314	123353	49.565
2	32.229	4824517	110512	50.435
Total		9565832	233866	100.000

Sample Name	: WH-813-14-46A3-2-IA-CHI		
Sample ID	:		
Data Filename	: WH-813-14-46A3-2-IA-CHI.lcd		
Method Filename	: WH-5-9505-1.0-60MIN.lcm		
Batch Filename	: wh.lcb		
Vial #	: 1-71	Sample Type	: Unknown
Injection Volume	: 10 uL		
Date Acquired	: 11/10/2022 12:45:29 PM	Acquired by	: System Administrator
Date Processed	: 11/10/2022 1:45:33 PM	Processed by	: System Administrator

<Chromatogram>

Datafile Name:WH-813-14-46A3-2-IA-CHI.lcd
Sample Name:WH-813-14-46A3-2-IA-CHI

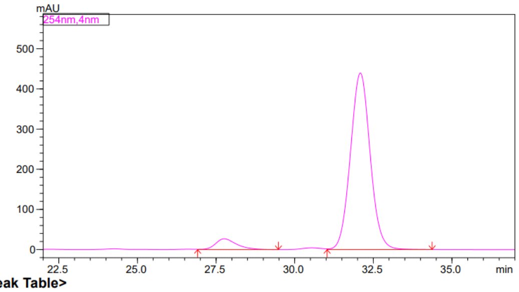

<Peak Table>

PDA Ch1 254nm

Peak#	Ret. Time	Area	Height	Area%
1	27.759	1249513	26894	6.116
2	32.087	19180690	439346	93.884
Total		20430202	466240	100.000

Supplementary Figure 172. HPLC spectra of **6c**

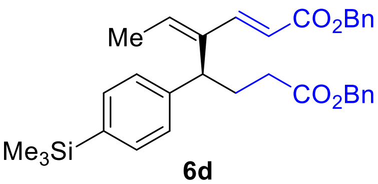

6d

Sample Name	: WH-813-14-60A2-IA-rac-2		
Sample ID	:		
Data Filename	: WH-813-14-60A2-IA-rac-2.lcd		
Method Filename	: WH-5-9505-0.5-60MIN.lcm		
Batch Filename	: wh.lcb		
Vial #	: 1-65	Sample Type	: Unknown
Injection Volume	: 10 uL		
Date Acquired	: 11/10/2022 11:27:37 PM	Acquired by	: System Administrator
Date Processed	: 11/11/2022 12:27:41 AM	Processed by	: System Administrator

<Chromatogram>

Datafile Name:WH-813-14-60A2-IA-rac-2.lcd
Sample Name:WH-813-14-60A2-IA-rac-2

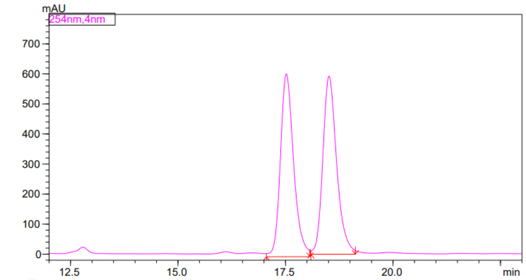

<Peak Table>

PDA Ch1 254nm

Peak#	Ret. Time	Area	Height	Area%
1	17.519	13063013	608455	49.564
2	18.512	13293056	592908	50.436
Total		26356069	1201363	100.000

Sample Name	: WH-813-14-46A2-2-IA-CHI		
Sample ID	:		
Data Filename	: WH-813-14-46A2-2-IA-CHI.lcd		
Method Filename	: WH-5-9505-0.5-60MIN.lcm		
Batch Filename	: wh.lcb		
Vial #	: 1-70	Sample Type	: Unknown
Injection Volume	: 10 uL		
Date Acquired	: 11/11/2022 12:28:12 AM	Acquired by	: System Administrator
Date Processed	: 11/11/2022 1:28:16 AM	Processed by	: System Administrator

<Chromatogram>

Datafile Name:WH-813-14-46A2-2-IA-CHI.lcd
Sample Name:WH-813-14-46A2-2-IA-CHI

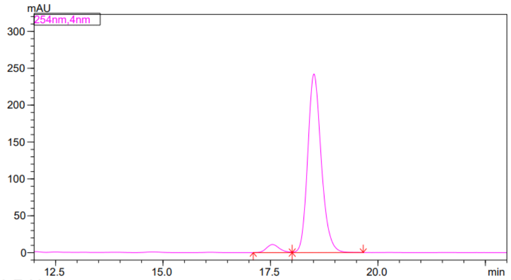

<Peak Table>

PDA Ch1 254nm

Peak#	Ret. Time	Area	Height	Area%
1	17.544	229987	10928	4.154
2	18.511	5306193	241942	95.846
Total		5536180	252870	100.000

Supplementary Figure 173. HPLC spectra of **6d**

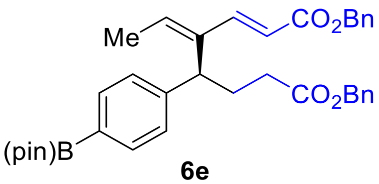

6e

Sample Name	: WH-813-14-47A4-2-OD-H-rac-1		
Sample ID	:		
Data Filename	: WH-813-14-47A4-2-OD-H-rac-1.lcd		
Method Filename	: WH-2-9505-0.8-60MIN.lcm		
Batch Filename	: wh.lcb		
Vial #	: 1-69	Sample Type	: Unknown
Injection Volume	: 10 uL		
Date Acquired	: 11/1/2022 11:30:14 PM	Acquired by	: System Administrator
Date Processed	: 11/2/2022 12:30:18 AM	Processed by	: System Administrator

<Chromatogram>

Datafile Name:WH-813-14-47A4-2-OD-H-rac-1.lcd
Sample Name:WH-813-14-47A4-2-OD-H-rac-1

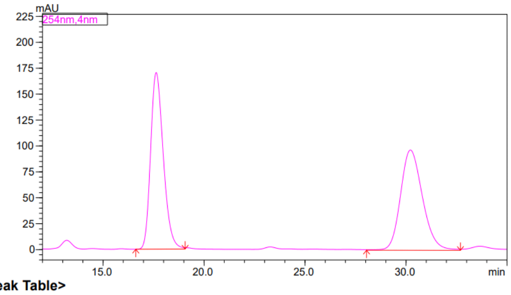

<Peak Table>

PDA Ch1 254nm

Peak#	Ret. Time	Area	Height	Area%
1	17.630	7457772	170431	50.453
2	30.217	7323721	96784	49.547
Total		14781493	267216	100.000

Sample Name	: WH-813-14-36A2-2-OD-H-CHI		
Sample ID	:		
Data Filename	: WH-813-14-36A2-2-OD-H-CHI.lcd		
Method Filename	: WH-2-9505-0.8-60MIN.lcm		
Batch Filename	: wh.lcb		
Vial #	: 1-46	Sample Type	: Unknown
Injection Volume	: 25 uL		
Date Acquired	: 11/2/2022 12:30:50 AM	Acquired by	: System Administrator
Date Processed	: 11/2/2022 1:30:53 AM	Processed by	: System Administrator

<Chromatogram>

Datafile Name:WH-813-14-36A2-2-OD-H-CHI.lcd
Sample Name:WH-813-14-36A2-2-OD-H-CHI

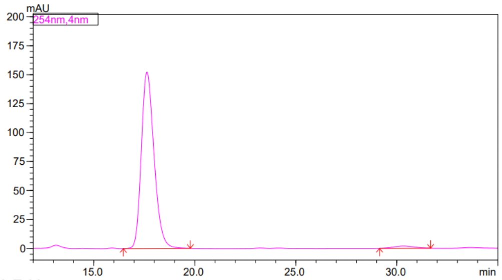

<Peak Table>

PDA Ch1 254nm

Peak#	Ret. Time	Area	Height	Area%
1	17.630	6680268	152307	97.957
2	30.317	139322	1984	2.043
Total		6819590	154291	100.000

Supplementary Figure 174. HPLC spectra of **6e**

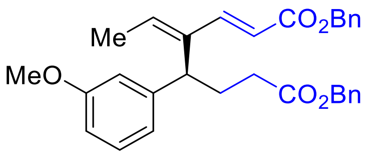

6f

Sample Name	: WH-813-14-60A1-IA-rac-1		
Sample ID	:		
Data Filename	: WH-813-14-60A1-IA-rac-1.lcd		
Method Filename	: WH-2-9505-1.0-40MIN.lcm		
Batch Filename	: wh.lcb		
Vial #	: 1-64	Sample Type	: Unknown
Injection Volume	: 10 uL		
Date Acquired	: 11/14/2022 3:50:13 AM	Acquired by	: System Administrator
Date Processed	: 11/14/2022 4:30:16 AM	Processed by	: System Administrator

<Chromatogram>

Datafile Name:WH-813-14-60A1-IA-rac-1.lcd
Sample Name:WH-813-14-60A1-IA-rac-1

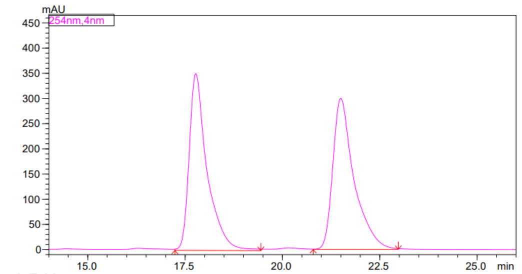

<Peak Table>

PDA Ch1 254nm

Peak#	Ret. Time	Area	Height	Area%
1	17.774	11238478	351050	49.754
2	21.498	11349801	300161	50.246
Total		22588279	651211	100.000

Sample Name	: WH-813-14-14-IA-CHI		
Sample ID	:		
Data Filename	: WH-813-14-14-IA-CHI.lcd		
Method Filename	: WH-2-9505-1.0-40MIN.lcm		
Batch Filename	: wh.lcb		
Vial #	: 1-67	Sample Type	: Unknown
Injection Volume	: 10 uL		
Date Acquired	: 11/14/2022 4:50:47 AM	Acquired by	: System Administrator
Date Processed	: 11/14/2022 5:30:49 AM	Processed by	: System Administrator

<Chromatogram>

Datafile Name:WH-813-14-14-IA-CHI.lcd
Sample Name:WH-813-14-14-IA-CHI

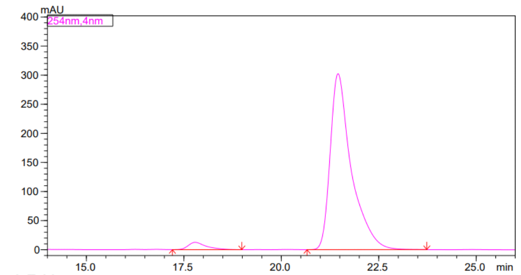

<Peak Table>

PDA Ch1 254nm

Peak#	Ret. Time	Area	Height	Area%
1	17.789	405307	12555	3.438
2	21.456	11384695	302401	96.562
Total		11790002	314956	100.000

Supplementary Figure 175. HPLC spectra of **6f**

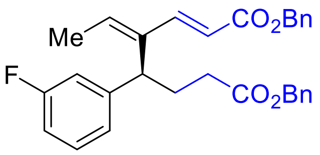

6g

Sample Name : WH-813-14-16A4-2-OD-H-rac-1
Sample ID :
Data Filename : WH-813-14-16A4-2-OD-H-rac-1.lcd
Method Filename : WH-2-9505-1.0-40MIN.lcm
Batch Filename : wh.lcb
Vial # : 1-67 Sample Type : Unknown
Injection Volume : 20 uL
Date Acquired : 10/15/2022 1:34:09 AM Acquired by : System Administrator
Date Processed : 10/15/2022 12:19:22 PM Processed by : System Administrator

<Chromatogram>

Datafile Name:WH-813-14-16A4-2-OD-H-rac-1.lcd
Sample Name:WH-813-14-16A4-2-OD-H-rac-1

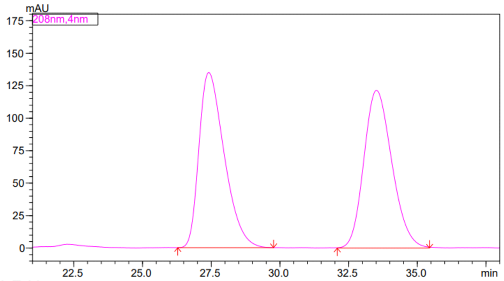

<Peak Table>

PDA Ch1 208nm

Peak#	Ret. Time	Area	Height	Area%
1	27.409	8627411	134849	50.015
2	33.507	8622352	121402	49.985
Total		17249763	256251	100.000

Sample Name : WH-813-14-15-2-OD-H-CHI
Sample ID :
Data Filename : WH-813-14-15-2-OD-H-CHI.lcd
Method Filename : WH-2-9505-1.0-40MIN.lcm
Batch Filename : wh.lcb
Vial # : 1-70 Sample Type : Unknown
Injection Volume : 20 uL
Date Acquired : 10/15/2022 2:14:43 AM Acquired by : System Administrator
Date Processed : 10/15/2022 2:54:46 AM Processed by : System Administrator

<Chromatogram>

Datafile Name:WH-813-14-15-2-OD-H-CHI.lcd
Sample Name:WH-813-14-15-2-OD-H-CHI

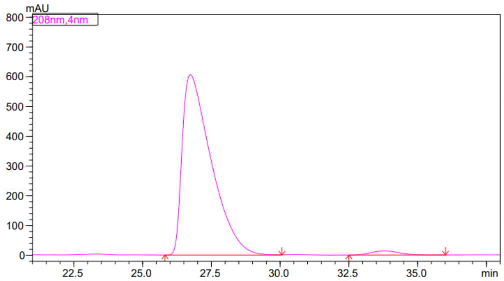

<Peak Table>

PDA Ch1 208nm

Peak#	Ret. Time	Area	Height	Area%
1	26.738	44448356	606314	97.838
2	33.787	982064	13994	2.162
Total		45430420	620308	100.000

Supplementary Figure 176. HPLC spectra of **6g**

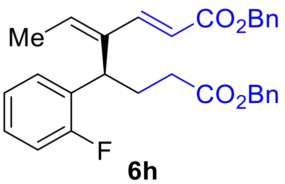

6h

Sample Name : WH-813-14-42A2-2-OD-H-rac-1
Sample ID :
Data Filename : WH-813-14-42A2-2-OD-H-rac-1.lcd
Method Filename : WH-2-9505-1.0-60MIN.lcm
Batch Filename : wh.lcb
Vial # : 1-64 Sample Type : Unknown
Injection Volume : 20 uL
Date Acquired : 11/1/2022 8:58:38 PM Acquired by : System Administrator
Date Processed : 11/1/2022 9:58:42 PM Processed by : System Administrator

<Chromatogram>

Datafile Name:WH-813-14-42A2-2-OD-H-rac-1.lcd
Sample Name:WH-813-14-42A2-2-OD-H-rac-1

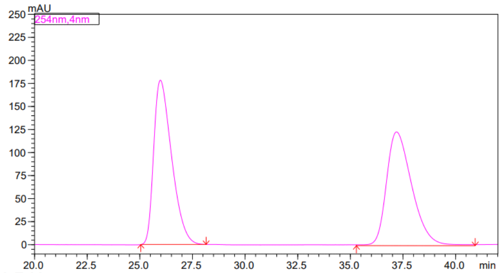

<Peak Table>

PDA Ch1 254nm

Peak#	Ret. Time	Area	Height	Area%
1	25.974	10566532	178249	50.384
2	37.194	10405593	123431	49.616
Total		20972125	301679	100.000

Sample Name : WH-813-14-38A2-2-OD-H-CHI
Sample ID :
Data Filename : WH-813-14-38A2-2-OD-H-CHI.lcd
Method Filename : WH-2-9505-1.0-60MIN.lcm
Batch Filename : wh.lcb
Vial # : 1-68 Sample Type : Unknown
Injection Volume : 25 uL
Date Acquired : 11/1/2022 9:59:13 PM Acquired by : System Administrator
Date Processed : 11/1/2022 10:59:15 PM Processed by : System Administrator

<Chromatogram>

Datafile Name:WH-813-14-38A2-2-OD-H-CHI.lcd
Sample Name:WH-813-14-38A2-2-OD-H-CHI

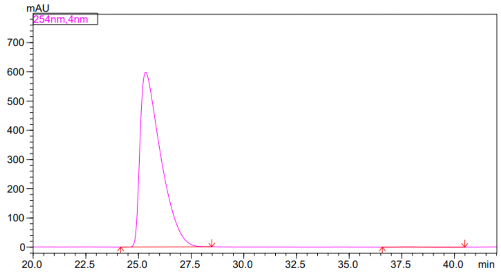

<Peak Table>

PDA Ch1 254nm

Peak#	Ret. Time	Area	Height	Area%
1	25.340	40773568	598091	99.803
2	37.756	80649	1101	0.197
Total		40854217	599192	100.000

Supplementary Figure 177. HPLC spectra of **6h**

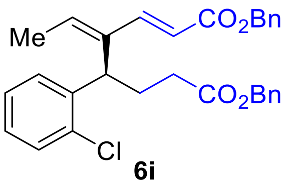

6i

Sample Name	: WH-813-13-106A1-2-rac-1-IC-9307-1.0-60MIN
Sample ID	:
Data Filename	: WH-813-13-106A1-2-rac-1-IC-9307-1.0-60MIN.lcd
Method Filename	: HW-5-9307-1.0-60MIN.lcm
Batch Filename	: hw.lcb
Vial #	: 1-66
Sample Type	: Unknown
Injection Volume	: 8 uL
Date Acquired	: 10/13/2022 6:12:16 AM
Acquired by	: System Administrator
Date Processed	: 10/13/2022 7:12:19 AM
Processed by	: System Administrator

<Chromatogram>

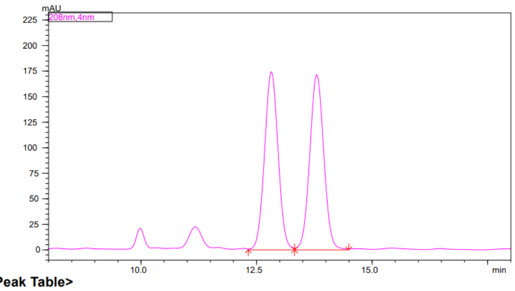

<Peak Table>

PDA Ch1 208nm

Peak#	Ret. Time	Area	Height	Area%
1	12.819	3566599	174367	49.590
2	13.802	3625637	171491	50.410
Total		7192235	345859	100.000

Sample Name	: WH-813-14-13-2-CHI-IC-9307-1.0-60MIN
Sample ID	:
Data Filename	: WH-813-14-13-2-CHI9307-1.0-60MIN.lcd
Method Filename	: HW-5-9307-1.0-60MIN.lcm
Batch Filename	: hw.lcb
Vial #	: 1-69
Sample Type	: Unknown
Injection Volume	: 8 uL
Date Acquired	: 10/13/2022 7:12:49 AM
Acquired by	: System Administrator
Date Processed	: 10/13/2022 8:12:52 AM
Processed by	: System Administrator

<Chromatogram>

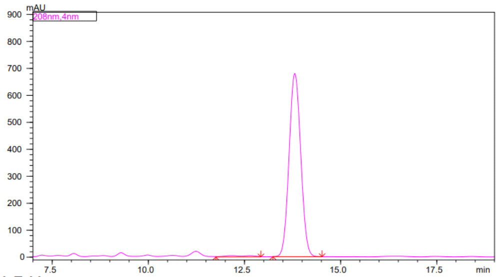

<Peak Table>

PDA Ch1 208nm

Peak#	Ret. Time	Area	Height	Area%
1	12.163	239560	4819	1.644
2	13.809	14333215	679174	98.356
Total		14572776	683993	100.000

Supplementary Figure 178. HPLC spectra of **6i**

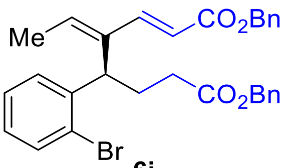

6j

Sample Name	: WH-813-14-48A3-2-OD-H-rac-1		
Sample ID	:		
Data Filename	: WH-813-14-48A3-2-OD-H-rac-1.lcd		
Method Filename	: WH-2-9505-1.0-60MIN.lcm		
Batch Filename	: wh.lcb		
Vial #	: 1-72	Sample Type	: Unknown
Injection Volume	: 25 uL		
Date Acquired	: 11/2/2022 7:05:03 AM	Acquired by	: System Administrator
Date Processed	: 11/2/2022 8:05:06 AM	Processed by	: System Administrator

<Chromatogram>

Datafile Name:WH-813-14-48A3-2-OD-H-rac-1.lcd
Sample Name:WH-813-14-48A3-2-OD-H-rac-1

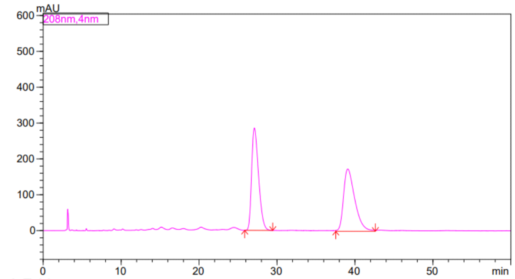

<Peak Table>

PDA Ch1 208nm

Peak#	Ret. Time	Area	Height	Area%
1	27.097	17702183	285658	50.266
2	39.093	17515138	173843	49.734
Total		35217321	459501	100.000

Sample Name	: WH-813-14-38A4-2-OD-H-CHI		
Sample ID	:		
Data Filename	: WH-813-14-38A4-2-OD-H-CHI.lcd		
Method Filename	: WH-2-9505-1.0-60MIN.lcm		
Batch Filename	: wh.lcb		
Vial #	: 1-74	Sample Type	: Unknown
Injection Volume	: 25 uL		
Date Acquired	: 11/2/2022 8:05:38 AM	Acquired by	: System Administrator
Date Processed	: 11/2/2022 9:05:40 AM	Processed by	: System Administrator

<Chromatogram>

Datafile Name:WH-813-14-38A4-2-OD-H-CHI.lcd
Sample Name:WH-813-14-38A4-2-OD-H-CHI

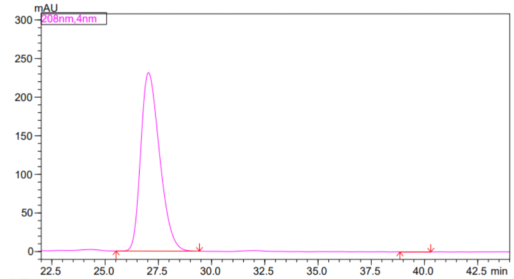

<Peak Table>

PDA Ch1 208nm

Peak#	Ret. Time	Area	Height	Area%
1	27.037	14253495	231208	99.860
2	39.809	19944	340	0.140
Total		14273439	231548	100.000

Supplementary Figure 179. HPLC spectra of **6j**

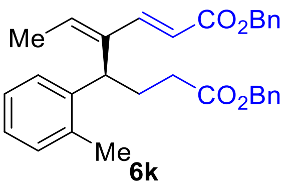

6k

Sample Name	: WH-813-13-78D2-3-OD-H-rac-1		
Sample ID	:		
Data Filename	: WH-813-13-78D2-3-OD-H-rac-1.lcd		
Method Filename	: WH-2-9505-1.0-40MIN.lcm		
Batch Filename	: wh.lcb		
Vial #	: 1-65	Sample Type	: Unknown
Injection Volume	: 20 uL		
Date Acquired	: 10/14/2022 9:31:00 PM	Acquired by	: System Administrator
Date Processed	: 10/14/2022 10:11:02 PM	Processed by	: System Administrator

<Chromatogram>

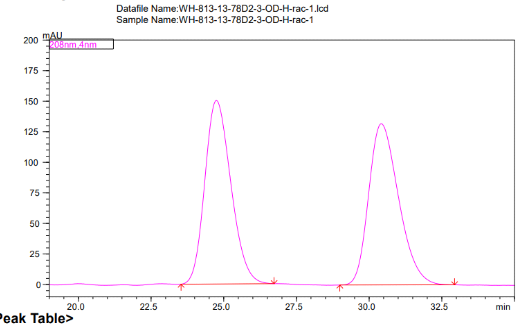

<Peak Table>

PDA Ch1 208nm

Peak#	Ret. Time	Area	Height	Area%
1	24.740	9383416	150147	49.848
2	30.420	9440825	131900	50.152
Total		18824241	282047	100.000

Sample Name	: WH-813-14-12-2-OD-H-CHI		
Sample ID	:		
Data Filename	: WH-813-14-12-2-OD-H-CHI.lcd		
Method Filename	: WH-2-9505-1.0-40MIN.lcm		
Batch Filename	: wh.lcb		
Vial #	: 1-68	Sample Type	: Unknown
Injection Volume	: 20 uL		
Date Acquired	: 10/14/2022 10:11:34 PM	Acquired by	: System Administrator
Date Processed	: 10/15/2022 12:16:03 PM	Processed by	: System Administrator

<Chromatogram>

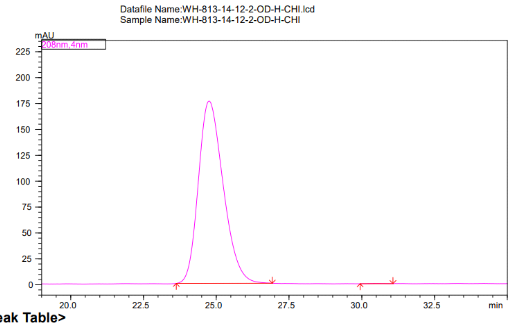

<Peak Table>

PDA Ch1 208nm

Peak#	Ret. Time	Area	Height	Area%
1	24.751	10438002	176017	99.922
2	30.457	8198	277	0.078
Total		10446200	176294	100.000

Supplementary Figure 180. HPLC spectra of **6k**

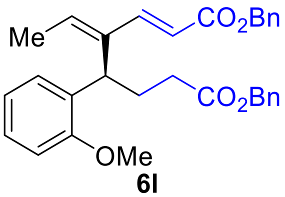

6l

Sample Name	: WH-813-14-42A1-2-IC-rac-2		
Sample ID	:		
Data Filename	: WH-813-14-42A1-2-IC-rac-2.lcd		
Method Filename	: WH-3-9505-1.0-40MIN.lcm		
Batch Filename	: wh.lcb		
Vial #	: 1-63	Sample Type	: Unknown
Injection Volume	: 10 uL		
Date Acquired	: 11/6/2022 7:36:26 PM	Acquired by	: System Administrator
Date Processed	: 11/6/2022 8:16:28 PM	Processed by	: System Administrator

<Chromatogram>

Datafile Name:WH-813-14-42A1-2-IC-rac-2.lcd
Sample Name:WH-813-14-42A1-2-IC-rac-2

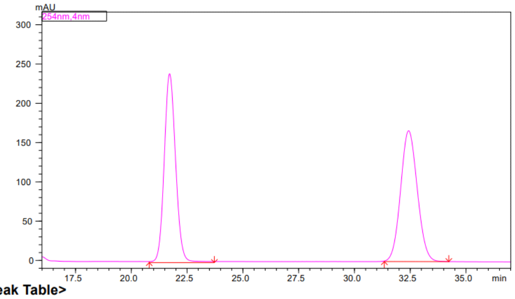

<Peak Table>

PDA Ch1 254nm

Peak#	Ret. Time	Area	Height	Area%
1	21.716	8575329	240403	49.698
2	32.429	8679600	166559	50.302
Total		17254930	406961	100.000

Sample Name	: WH-813-14-38A1-2-IC-CHI-1		
Sample ID	:		
Data Filename	: WH-813-14-38A1-2-IC-CHI-1.lcd		
Method Filename	: WH-3-9505-1.0-40MIN.lcm		
Batch Filename	: wh.lcb		
Vial #	: 1-67	Sample Type	: Unknown
Injection Volume	: 25 uL		
Date Acquired	: 11/6/2022 8:17:00 PM	Acquired by	: System Administrator
Date Processed	: 11/6/2022 8:57:02 PM	Processed by	: System Administrator

<Chromatogram>

Datafile Name:WH-813-14-38A1-2-IC-CHI-1.lcd
Sample Name:WH-813-14-38A1-2-IC-CHI-1

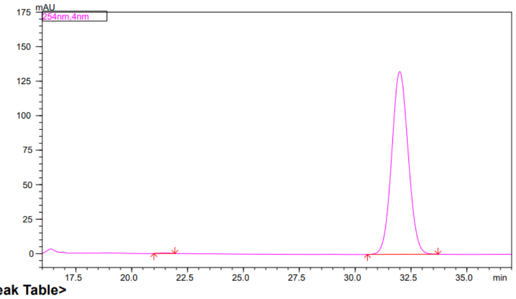

<Peak Table>

PDA Ch1 254nm

Peak#	Ret. Time	Area	Height	Area%
1	21.496	13980	469	0.205
2	31.995	6807995	132412	99.795
Total		6821975	132881	100.000

Supplementary Figure 181. HPLC spectra of **6l**

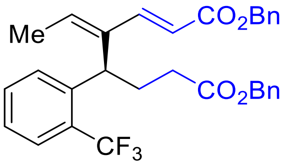

6m

Sample Name : WH-813-14-33A3-2-OZ-H-rac-3
Sample ID :
Data Filename : WH-813-14-33A3-2-OZ-H-rac-3.lcd
Method Filename : WH-1-9505-1.0-60MIN.lcm
Batch Filename : wh.lcb
Vial # : 1-61 Sample Type : Unknown
Injection Volume : 10 uL
Date Acquired : 11/1/2022 4:56:27 AM Acquired by : System Administrator
Date Processed : 11/1/2022 5:56:30 AM Processed by : System Administrator

<Chromatogram>

Datafile Name:WH-813-14-33A3-2-OZ-H-rac-3.lcd
Sample Name:WH-813-14-33A3-2-OZ-H-rac-3

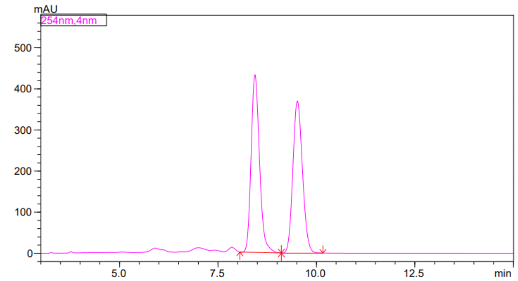

<Peak Table>

PDA Ch1 254nm

Peak#	Ret. Time	Area	Height	Area%
1	8.438	6407024	432310	50.445
2	9.516	6294079	370254	49.555
Total		12701104	802564	100.000

Sample Name : WH-813-14-38A3-2-OZ-H-chi
Sample ID :
Data Filename : WH-813-14-38A3-2-OZ-H-chi.lcd
Method Filename : WH-1-9505-1.0-60MIN.lcm
Batch Filename : wh.lcb
Vial # : 1-65 Sample Type : Unknown
Injection Volume : 10 uL
Date Acquired : 11/1/2022 5:57:00 AM Acquired by : System Administrator
Date Processed : 11/1/2022 6:57:03 AM Processed by : System Administrator

<Chromatogram>

Datafile Name:WH-813-14-38A3-2-OZ-H-chi.lcd
Sample Name:WH-813-14-38A3-2-OZ-H-chi

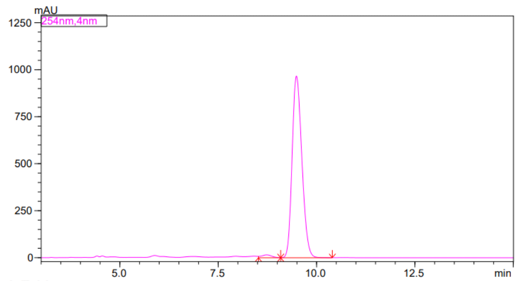

<Peak Table>

PDA Ch1 254nm

Peak#	Ret. Time	Area	Height	Area%
1	8.735	275962	15075	1.626
2	9.488	16700294	966045	98.374
Total		16976255	981120	100.000

Supplementary Figure 182. HPLC spectra of **6m**

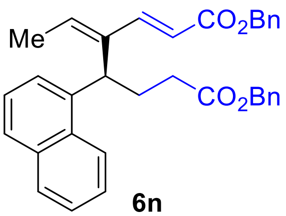

6n

Sample Name	: WH-813-14-48A2-2-OD-H-rac-1		
Sample ID	:		
Data Filename	: WH-813-14-48A2-2-OD-H-rac-1.lcd		
Method Filename	: WH-2-9505-1.0-60MIN.lcm		
Batch Filename	: wh.lcb		
Vial #	: 1-70	Sample Type	: Unknown
Injection Volume	: 10 uL		
Date Acquired	: 11/2/2022 2:01:49 AM	Acquired by	: System Administrator
Date Processed	: 11/2/2022 3:01:52 AM	Processed by	: System Administrator

<Chromatogram>

Datafile Name:WH-813-14-48A2-2-OD-H-rac-1.lcd
Sample Name:WH-813-14-48A2-2-OD-H-rac-1

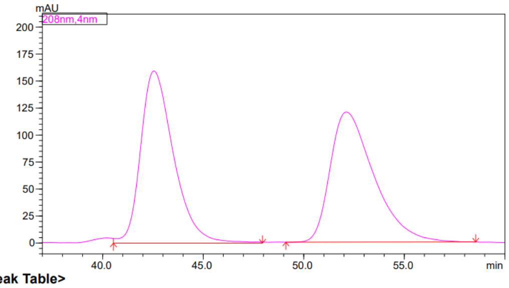

<Peak Table>

PDA Ch1 208nm

Peak#	Ret. Time	Area	Height	Area%
1	42.558	18222997	159461	49.963
2	52.119	18249969	120478	50.037
Total		36472966	279938	100.000

Sample Name	: WH-813-14-38A5-2-OD-H-CHI		
Sample ID	:		
Data Filename	: WH-813-14-38A5-2-OD-H-CHI.lcd		
Method Filename	: WH-2-9505-1.0-60MIN.lcm		
Batch Filename	: wh.lcb		
Vial #	: 1-73	Sample Type	: Unknown
Injection Volume	: 25 uL		
Date Acquired	: 11/2/2022 3:02:23 AM	Acquired by	: System Administrator
Date Processed	: 11/2/2022 4:02:26 AM	Processed by	: System Administrator

<Chromatogram>

Datafile Name:WH-813-14-38A5-2-OD-H-CHI.lcd
Sample Name:WH-813-14-38A5-2-OD-H-CHI

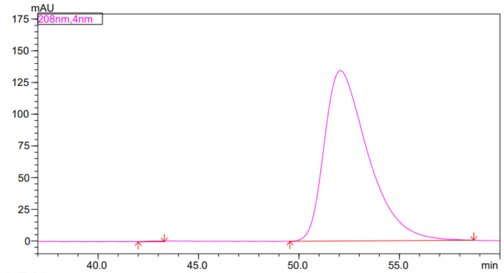

<Peak Table>

PDA Ch1 208nm

Peak#	Ret. Time	Area	Height	Area%
1	43.283	31282	566	0.154
2	52.073	20341730	134398	99.846
Total		20373012	134964	100.000

Supplementary Figure 183. HPLC spectra of **6n**

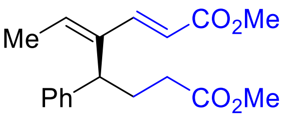

7a

Sample Name	: WH-813-14-57D2-IC-rac		
Sample ID	:		
Data Filename	: WH-813-14-57D2-IC-rac.lcd		
Method Filename	: WH-3-9505-1.0-60MIN.lcm		
Batch Filename	: wh.lcb		
Vial #	: 1-61	Sample Type	: Unknown
Injection Volume	: 10 uL		
Date Acquired	: 11/7/2022 3:01:10 AM	Acquired by	: System Administrator
Date Processed	: 11/7/2022 4:01:14 AM	Processed by	: System Administrator

<Chromatogram>

Datafile Name:WH-813-14-57D2-IC-rac.lcd
Sample Name:WH-813-14-57D2-IC-rac

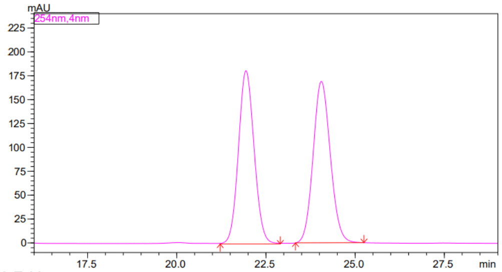

<Peak Table>

PDA Ch1 254nm

Peak#	Ret. Time	Area	Height	Area%
1	21.939	5571706	181433	49.551
2	24.052	5672591	169099	50.449
Total		11244297	350532	100.000

Sample Name	: WH-813-14-57D1-2-IC-CHI		
Sample ID	:		
Data Filename	: WH-813-14-57D1-2-IC-CHI.lcd		
Method Filename	: WH-3-9505-1.0-40MIN.lcm		
Batch Filename	: wh.lcb		
Vial #	: 1-67	Sample Type	: Unknown
Injection Volume	: 20 uL		
Date Acquired	: 11/8/2022 2:51:02 AM	Acquired by	: System Administrator
Date Processed	: 11/9/2022 12:57:19 AM	Processed by	: System Administrator

<Chromatogram>

Datafile Name:WH-813-14-57D1-2-IC-CHI.lcd
Sample Name:WH-813-14-57D1-2-IC-CHI

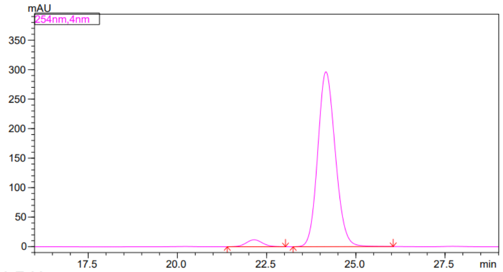

<Peak Table>

PDA Ch1 254nm

Peak#	Ret. Time	Area	Height	Area%
1	22.151	373677	11762	3.562
2	24.162	10117039	296476	96.438
Total		10490716	308238	100.000

Supplementary Figure 184. HPLC spectra of **7a**

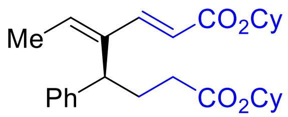

7b

Sample Name : WH-813-14-58D2-2-IC-rac-1
Sample ID :
Data Filename : WH-813-14-58D2-2-IC-rac-1.lcd
Method Filename : WH-3-9505-0.8-40MIN.lcm
Batch Filename : wh.lcb
Vial # : 1-62
Sample Type : Unknown
Injection Volume : 10 uL
Date Acquired : 11/8/2022 4:02:02 AM
Acquired by : System Administrator
Date Processed : 11/9/2022 12:57:21 AM
Processed by : System Administrator

<Chromatogram>

Datafile Name:WH-813-14-58D2-2-IC-rac-1.lcd
Sample Name:WH-813-14-58D2-2-IC-rac-1

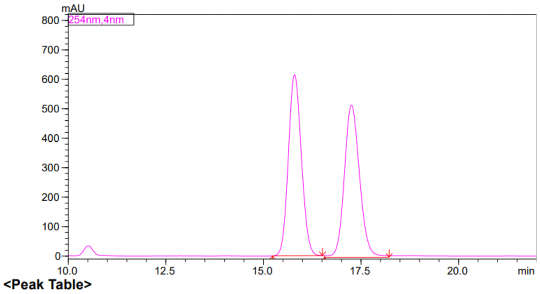

<Peak Table>

PDA Ch1 254nm

Peak#	Ret. Time	Area	Height	Area%
1	15.796	14243669	614908	50.388
2	17.255	14024395	516913	49.612
Total		28268064	1131820	100.000

Sample Name : WH-813-14-58D1-2-IC-CHI
Sample ID :
Data Filename : WH-813-14-58D1-2-IC-CHI.lcd
Method Filename : WH-3-9505-0.8-40MIN.lcm
Batch Filename : wh.lcb
Vial # : 1-68
Sample Type : Unknown
Injection Volume : 25 uL
Date Acquired : 11/8/2022 4:42:35 AM
Acquired by : System Administrator
Date Processed : 11/9/2022 12:57:23 AM
Processed by : System Administrator

<Chromatogram>

Datafile Name:WH-813-14-58D1-2-IC-CHI.lcd
Sample Name:WH-813-14-58D1-2-IC-CHI

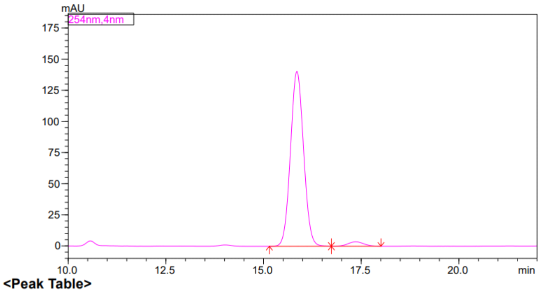

<Peak Table>

PDA Ch1 254nm

Peak#	Ret. Time	Area	Height	Area%
1	15.854	3284048	140414	97.046
2	17.356	99981	3537	2.954
Total		3384028	143951	100.000

Supplementary Figure 185. HPLC spectra of **7b**

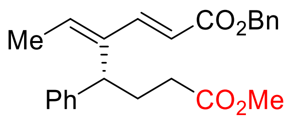

17

Sample Name	: WH-813-16-72-IG-rac-1
Sample ID	:
Data Filename	: WH-813-16-72-IG-rac-1.lcd
Method Filename	: WH-6-9505-0.7-60MIN.lcm
Batch Filename	: wh12.3.lcb
Vial #	: 1-66
Injection Volume	: 10 uL
Date Acquired	: 5/26/2023 9:56:30 AM
Date Processed	: 5/31/2023 10:44:46 AM
Sample Type	: Unknown
Acquired by	: System Administrator
Processed by	: System Administrator

<Chromatogram>

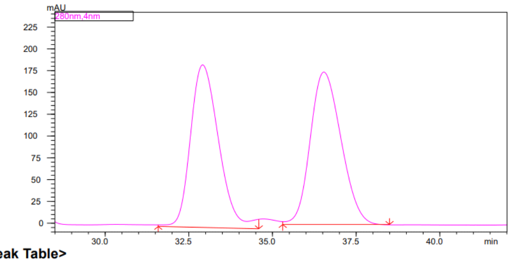

<Peak Table>

PDA Ch1 280nm

Peak#	Ret. Time	Area	Area%
1	32.913	10992863	49.504
2	36.534	11212972	50.496
Total		22205835	100.000

Sample Name	: WH-813-16-78-2-IG-CHI
Sample ID	:
Data Filename	: WH-813-16-78-2-IG-CHI.lcd
Method Filename	: WH-6-9505-0.7-60MIN.lcm
Batch Filename	: wh12.3.lcb
Vial #	: 1-67
Injection Volume	: 10 uL
Date Acquired	: 5/26/2023 11:17:04 AM
Date Processed	: 5/26/2023 12:02:43 PM
Sample Type	: Unknown
Acquired by	: System Administrator
Processed by	: System Administrator

<Chromatogram>

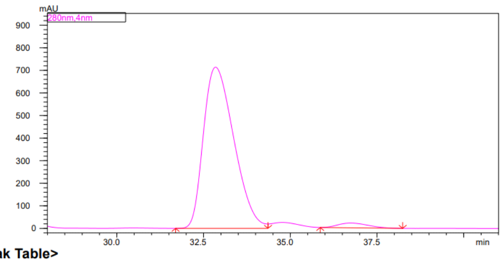

<Peak Table>

PDA Ch1 280nm

Peak#	Ret. Time	Area	Area%
1	32.848	43541645	97.164
2	36.740	1270961	2.836
Total		44812606	100.000

Supplementary Figure 186. HPLC spectra of **17**

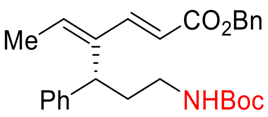

18

Sample Name : WH-813-16-87-OD-H-rac-2
Sample ID :
Data Filename : WH-813-16-87-OD-H-rac-2.lcd
Method Filename : WH-4-9505-1.0-60MIN.lcm
Batch Filename : wh12.3.lcb
Vial # : 1-63
Sample Type : Unknown
Injection Volume : 20 uL
Date Acquired : 6/3/2023 11:54:38 PM
Acquired by : System Administrator
Date Processed : 6/4/2023 12:54:41 AM
Processed by : System Administrator

<Chromatogram>

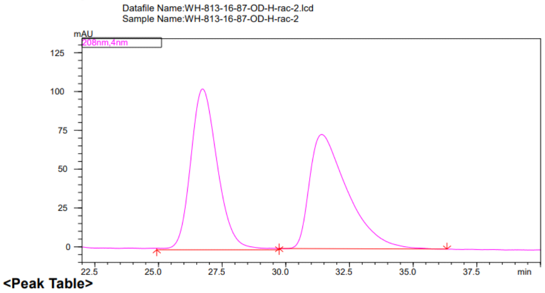

<Peak Table>

PDA Ch1 208nm

Peak#	Ret. Time	Area	Area%
1	26.733	7612913	49.961
2	31.416	7624684	50.039
Total		15237598	100.000

Sample Name : WH-813-16-89-OD-H-chi
Sample ID :
Data Filename : WH-813-16-89-OD-H-chi.lcd
Method Filename : WH-4-9505-1.0-60MIN.lcm
Batch Filename : wh12.3.lcb
Vial # : 1-64
Sample Type : Unknown
Injection Volume : 10 uL
Date Acquired : 6/4/2023 12:55:12 AM
Acquired by : System Administrator
Date Processed : 6/4/2023 1:55:16 AM
Processed by : System Administrator

<Chromatogram>

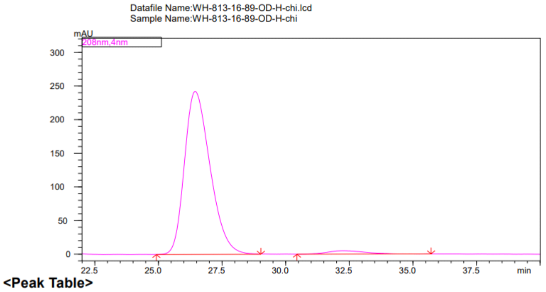

<Peak Table>

PDA Ch1 208nm

Peak#	Ret. Time	Area	Area%
1	26.445	16986297	97.080
2	32.306	510894	2.920
Total		17497191	100.000

Supplementary Figure 187. HPLC spectra of **18**

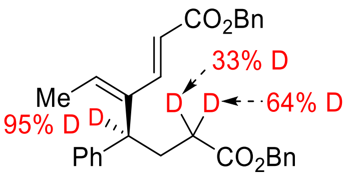

24

Sample Name : WH-813-14-65-2-IB-rac-1
Sample ID :
Data Filename : WH-813-14-65-2-IB-rac-1.lcd
Method Filename : WH-4-9505-1.0-25MIN.lcm
Batch Filename : wh.lcb
Vial # : 1-68
Injection Volume : 10 uL
Date Acquired : 11/17/2022 7:29:36 PM
Date Processed : 11/17/2022 7:54:38 PM
Sample Type : Unknown
Acquired by : System Administrator
Processed by : System Administrator

<Chromatogram>

Datafile Name:WH-813-14-65-2-IB-rac-1.lcd
Sample Name:WH-813-14-65-2-IB-rac-1

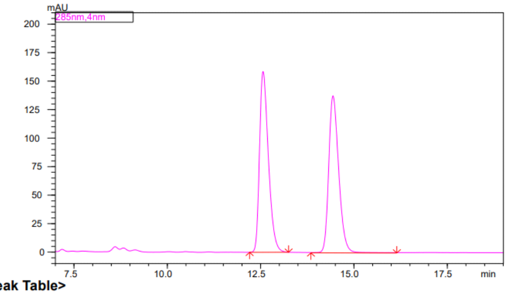

<Peak Table>

PDA Ch1 285nm

Peak#	Ret. Time	Area	Height	Area%
1	12.565	2623288	158629	50.401
2	14.439	2581541	137800	49.599
Total		5204829	296429	100.000

Sample Name : WH-813-14-62-2-IB-CHI
Sample ID :
Data Filename : WH-813-14-62-2-IB-CHI.lcd
Method Filename : WH-4-9505-1.0-25MIN.lcm
Batch Filename : wh.lcb
Vial # : 1-69
Injection Volume : 20 uL
Date Acquired : 11/17/2022 8:15:09 PM
Date Processed : 11/17/2022 8:40:13 PM
Sample Type : Unknown
Acquired by : System Administrator
Processed by : System Administrator

<Chromatogram>

Datafile Name:WH-813-14-62-2-IB-CHI.lcd
Sample Name:WH-813-14-62-2-IB-CHI

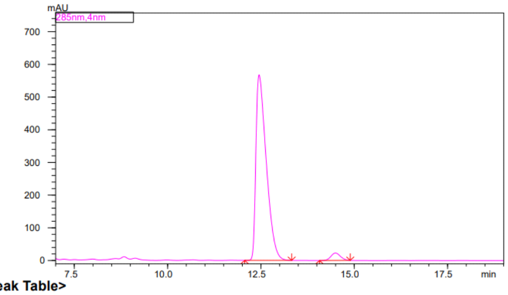

<Peak Table>

PDA Ch1 285nm

Peak#	Ret. Time	Area	Height	Area%
1	12.451	10965750	567704	96.492
2	14.495	398693	22646	3.508
Total		11364442	590350	100.000

Supplementary Figure 188. HPLC spectra of **24**

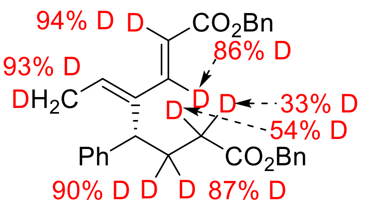

25

Sample Name : WH-813-13-56D2-3-IG-rac-2
Sample ID :
Data Filename : WH-813-13-56D2-3-IG-rac-2.lcd
Method Filename : WH-1-9505-1.0-60MIN.lcm
Batch Filename : wh12.3.lcb
Vial # : 1-64
Injection Volume : 20 uL
Date Acquired : 2/4/2023 11:34:50 AM
Date Processed : 2/4/2023 12:34:54 PM
Sample Type : Unknown
Acquired by : System Administrator
Processed by : System Administrator

<Chromatogram>

Datafile Name:WH-813-13-56D2-3-IG-rac-2.lcd
Sample Name:WH-813-13-56D2-3-IG-rac-2

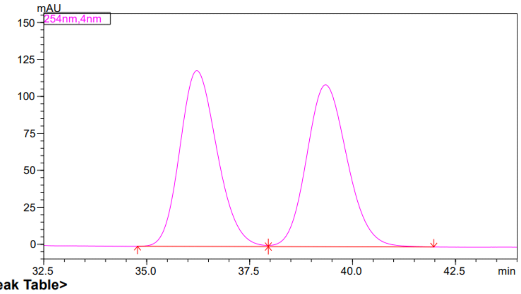

<Peak Table>

PDA Ch1 254nm

Peak#	Ret. Time	Area	Height	Area%
1	36.215	7495219	118906	49.946
2	39.346	7511551	109493	50.054
Total		15006770	228399	100.000

Sample Name : WH-813-15-50-IG-CHI
Sample ID :
Data Filename : WH-813-15-50-IG-CHI.lcd
Method Filename : WH-1-9505-1.0-60MIN.lcm
Batch Filename : wh12.3.lcb
Vial # : 1-65
Injection Volume : 20 uL
Date Acquired : 2/4/2023 12:35:26 PM
Date Processed : 2/4/2023 1:35:30 PM
Sample Type : Unknown
Acquired by : System Administrator
Processed by : System Administrator

<Chromatogram>

Datafile Name:WH-813-15-50-IG-CHI.lcd
Sample Name:WH-813-15-50-IG-CHI

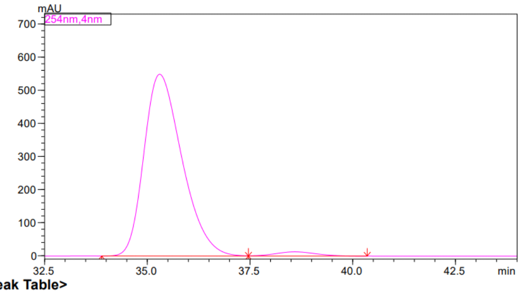

<Peak Table>

PDA Ch1 254nm

Peak#	Ret. Time	Area	Height	Area%
1	35.302	35456431	549364	97.581
2	38.593	879114	12777	2.419
Total		36335545	562141	100.000

Supplementary Figure 189. HPLC spectra of **25**

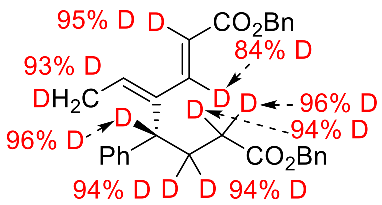

31

Sample Name : WH-813-13-56D2-3-IBN-5-rac-6
Sample ID :
Data Filename : WH-813-13-56D2-3-IBN-5-rac-6.lcd
Method Filename : WH-4-9505-1.0-40MIN.lcm
Batch Filename : wh12.3.lcb
Vial # : 1-64
Sample Type : Unknown
Injection Volume : 20 uL
Date Acquired : 3/2/2023 1:13:11 PM
Acquired by : System Administrator
Date Processed : 3/2/2023 1:53:14 PM
Processed by : System Administrator

<Chromatogram>

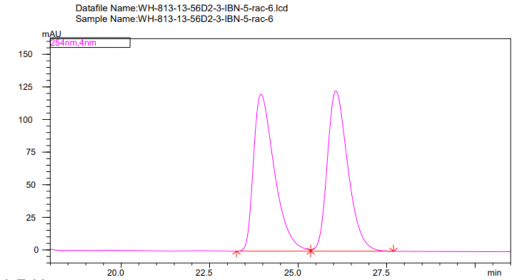

<Peak Table>

PDA Ch1 254nm

Peak#	Ret. Time	Area	Area%
1	23.950	4943780	49.878
2	26.063	4967947	50.122
Total		9911727	100.000

Sample Name : WH-813-15-75-3-IBN-5-CHI-2
Sample ID :
Data Filename : WH-813-15-75-3-IBN-5-CHI-2.lcd
Method Filename : WH-4-9505-1.0-40MIN.lcm
Batch Filename : wh12.3.lcb
Vial # : 1-65
Sample Type : Unknown
Injection Volume : 10 uL
Date Acquired : 3/2/2023 2:13:45 PM
Acquired by : System Administrator
Date Processed : 3/2/2023 2:53:49 PM
Processed by : System Administrator

<Chromatogram>

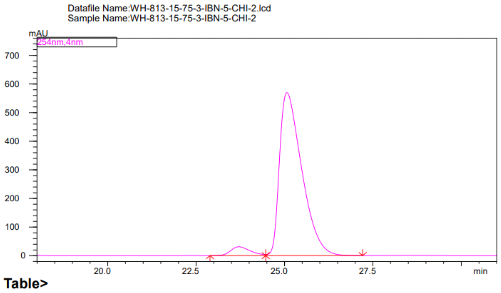

<Peak Table>

PDA Ch1 254nm

Peak#	Ret. Time	Area	Area%
1	23.709	1192268	4.559
2	25.067	24961345	95.441
Total		26153613	100.000

Supplementary Figure 190. HPLC spectra of **31**

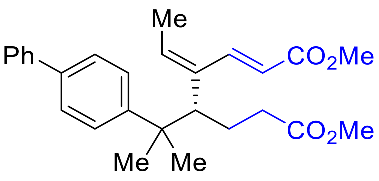

36

Sample Name	: WH-813-13-2D2-IBN-5-rac-1		
Sample ID	:		
Data Filename	: WH-813-13-2D2-IBN-5-rac-1.lcd		
Method Filename	: WH-4-9505-0.5-60MIN.lcm		
Batch Filename	: wh12.3.lcb		
Vial #	: 1-87	Sample Type	: Unknown
Injection Volume	: 20 uL		
Date Acquired	: 1/20/2023 1:04:39 AM	Acquired by	: System Administrator
Date Processed	: 1/20/2023 2:04:42 AM	Processed by	: System Administrator

<Chromatogram>

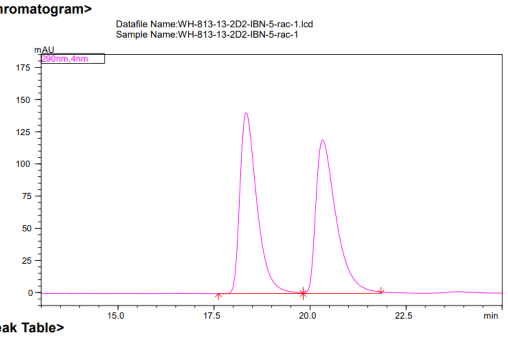

<Peak Table>

PDA Ch1 290nm

Peak#	Ret. Time	Area	Height	Area%
1	18.337	4344523	140540	49.731
2	20.329	4391576	119354	50.269
Total		8736099	259893	100.000

Sample Name	: WH-813-15-32-IBN-5-CHI		
Sample ID	:		
Data Filename	: WH-813-15-32-IBN-5-CHI.lcd		
Method Filename	: WH-4-9505-0.5-60MIN.lcm		
Batch Filename	: wh12.3.lcb		
Vial #	: 1-88	Sample Type	: Unknown
Injection Volume	: 10 uL		
Date Acquired	: 1/20/2023 2:25:14 AM	Acquired by	: System Administrator
Date Processed	: 1/20/2023 3:25:17 AM	Processed by	: System Administrator

<Chromatogram>

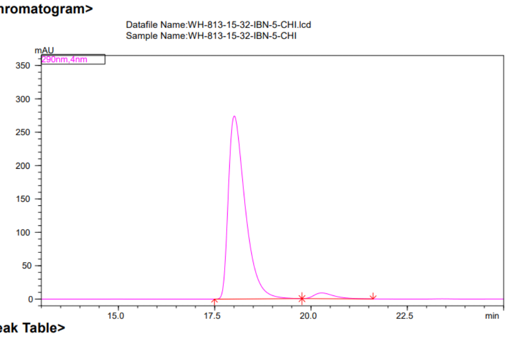

<Peak Table>

PDA Ch1 290nm

Peak#	Ret. Time	Area	Height	Area%
1	18.008	8378308	274223	96.380
2	20.285	314714	8654	3.620
Total		8693022	282876	100.000

Supplementary Figure 191. HPLC spectra of **36**

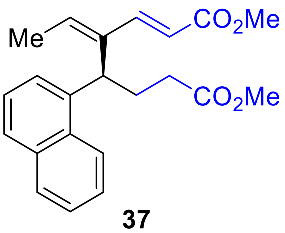

37

Sample Name : WH-813-15-27-2-IBN-5-rac-1
Sample ID :
Data Filename : WH-813-15-27-2-IBN-5-rac-1.lcd
Method Filename : WH-4-9505-1.0-60MIN.lcm
Batch Filename : wh12.3.lcb
Vial # : 1-61
Injection Volume : 10 uL
Date Acquired : 1/4/2023 2:13:05 AM
Date Processed : 1/4/2023 3:13:08 AM
Sample Type : Unknown
Acquired by : System Administrator
Processed by : System Administrator

<Chromatogram>

Datafile Name:WH-813-15-27-2-IBN-5-rac-1.lcd
Sample Name:WH-813-15-27-2-IBN-5-rac-1

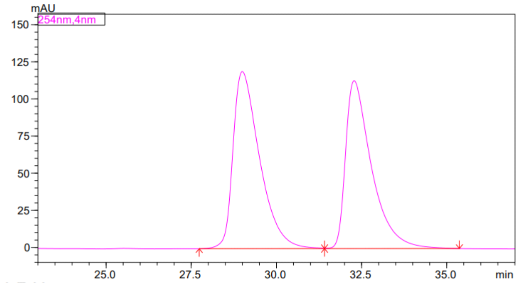

<Peak Table>

PDA Ch1 254nm

Peak#	Ret. Time	Area	Height	Area%
1	28.998	6481564	119252	51.968
2	32.282	5990745	113023	48.032
Total		12472310	232275	100.000

Sample Name : WH-813-15-26-2-IBN-5-CHI
Sample ID :
Data Filename : WH-813-15-26-2-IBN-5-CHI.lcd
Method Filename : WH-4-9505-1.0-60MIN.lcm
Batch Filename : wh12.3.lcb
Vial # : 1-88
Injection Volume : 10 uL
Date Acquired : 1/4/2023 3:13:39 AM
Date Processed : 1/4/2023 4:13:41 AM
Sample Type : Unknown
Acquired by : System Administrator
Processed by : System Administrator

<Chromatogram>

Datafile Name:WH-813-15-26-2-IBN-5-CHI.lcd
Sample Name:WH-813-15-26-2-IBN-5-CHI

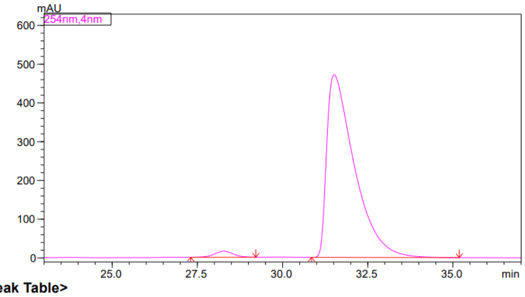

<Peak Table>

PDA Ch1 254nm

Peak#	Ret. Time	Area	Height	Area%
1	28.267	599624	15702	2.200
2	31.517	26650080	471535	97.800
Total		27249704	487236	100.000

Supplementary Figure 192. HPLC spectra of **37**

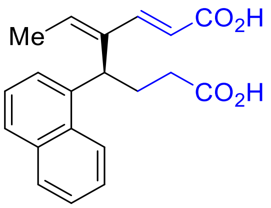

38

Sample Name : WH-813-15-52-2-AD-H-RAC-4
Sample ID :
Data Filename : WH-813-15-52-2-AD-H-RAC-4.lcd
Method Filename : WH-4-9010-3.0-60min.lcm
Batch Filename : wh-1.lcb
Vial # : 1-63
Sample Type : Unknown
Injection Volume : 20 uL
Date Acquired : 2/8/2023 2:05:20 AM
Acquired by : System Administrator
Date Processed : 2/8/2023 3:05:38 AM
Processed by : System Administrator

<Chromatogram>

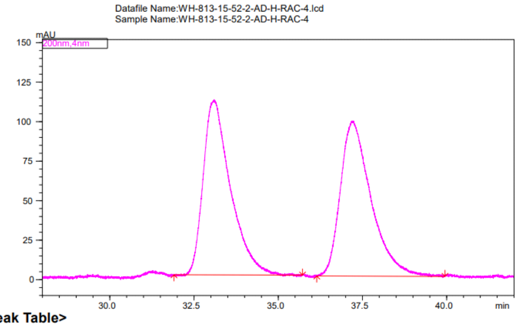

<Peak Table>

PDA Ch1 200nm

Peak#	Ret. Time	Area	Height	Area%
1	33.095	6217905	110310	49.997
2	37.197	6218637	97917	50.003
Total		12436542	208228	100.000

Sample Name : WH-813-15-36-AD-H-chi-2
Sample ID :
Data Filename : WH-813-15-36-AD-H-chi-2.lcd
Method Filename : WH-4-9010-3.0-60min.lcm
Batch Filename : wh-1.lcb
Vial # : 1-69
Sample Type : Unknown
Injection Volume : 20 uL
Date Acquired : 2/8/2023 3:37:19 AM
Acquired by : System Administrator
Date Processed : 2/8/2023 4:37:33 AM
Processed by : System Administrator

<Chromatogram>

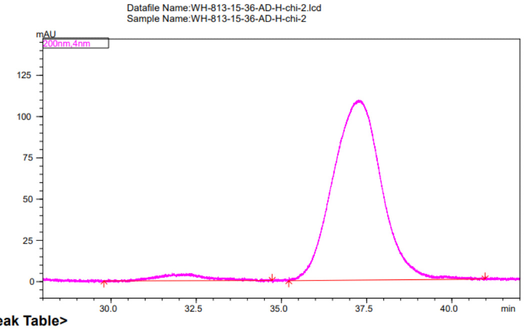

<Peak Table>

PDA Ch1 200nm

Peak#	Ret. Time	Area	Height	Area%
1	32.205	393361	4104	3.520
2	37.255	10782945	108495	96.480
Total		11176306	112600	100.000

Supplementary Figure 193. HPLC spectra of **38**

3. Supplementary References

1. J. H. Kim, E.-S. Park, J. H. Shim, M.-N. Kim, W.-S. Moon, K.-H. Chung, J.-S. Yoon, *J. Agric. Food Chem.* **52**, 7480-7483 (2004).
2. P. Keller, D. L. Thomsen, M. H. Li, *Macromolecules*. **35**, 581-584 (2002).
3. S. Lee, T. Rovis, *ACS Catal.* **11**, 8585-8590 (2021).
4. S. Rej, N. Chatani, *Chem. Eur. J.* **26**, 11093-11098 (2020).
5. Y. Luo, I. D. Roy, A. G. E. Madec, H. W. Lam, *Angew. Chem. Int. Ed. 53*, 4186-4190 (2014).
6. J.-K. Cheng, T.-P. Loh, *J. Am. Chem. Soc.* **137**, 42-45 (2015).
7. Y. Liao, X. Yin, X. Wang, W. Yu, D. Fang, L. Hu, M. Wang, J. Liao, *Angew. Chem. Int. Ed.* **59**, 1176-1180 (2020).
8. S.-Q. Yang, Y.-F. Wang, W.-C. Zhao, G.-Q. Lin, Z.-T. He, *J. Am. Chem. Soc.* **143**, 7285-7291 (2021).
9. M.-I. Picher, B. Plietker, *Org. Lett. 22*, 340-344 (2020).
10. M. Rivara, M. K. Patel, L. Amori, V. Zuliani, *Bioorganic Med. Chem. Lett.* **22**, 6401-6404 (2012).
11. J. Li, D. Smith, S. Krishnananthan, D.-R. Wu, D. Sun, P. Li, K. Ryan, M. Hu, W. Cui, J. Naginskaya, S. Liu, P. C. Lobben, A. T. Ng, R. Olson, A. Mathur, *Tetrahedron: Asymmetry* **28**, 196–202 (2017).
12. J. K. Park, H. H. Lackey, M. D. Rexford, K. Kovnir, M. Shatruk, D. T. McQuade, *Org. Lett.* **12**, 5008–5011 (2010).
13. T. Shioiri, K. Ninomiya, S. Yamada, *J. Am. Chem. Soc.* **94**, 6203–6205 (1972).
14. C. Wu, P. A. Miller, M. J. Miller, *Bioorganic Med. Chem. Lett.* **21**, 2611-2615 (2011).
15. Y. Li, W.-S. Zhang, S.-N. Yang, X.-Y. Wang, Y. Liu, D.-W. Ji, Q.-A. Chen, *Angew. Chem. Int. Ed.* **62**, e202300036 (2023).
16. T. W. Funk, J. Efskind, R. H. Grubbs, *Org. Lett.* 7, 187–190 (2005).
17. J.-T. Zhang, H.-Y. Wang, W. Zhu, T.-T. Cai, Y.-L. Guo, *Anal. Chem.* 86, 8937-8942 (2014).
